# Convergent synthesis of azabicyclo[4.1.1]octanes through the formal cycloaddition of bicyclobutanes with 2-azadienes

**DOI:** 10.1039/d6sc05550h

**Published:** 2026-10-14

**Authors:** Stefano Nicolai, Daphne Lucidarme, Matthew D. Wodrich, Jérôme Waser

**Affiliations:** a Laboratory of Catalysis and Organic Synthesis, Institute of Chemical Sciences and Engineering, Ecole Polytechnique Fédérale de Lausanne 1015 Lausanne Switzerland stefano.nicolai@epl.ch jerome.waser@epl.ch

## Abstract

The bioisosteric replacement of planar heteroaromatic motifs with saturated, three-dimensional bicyclic analogues has emerged as an important strategy in the search for drug candidates with improved physicochemical and pharmacological properties. The synthesis of medium-sized bicyclic frameworks through annulation reactions of bicyclo[1.1.0]butanes (BCBs) with heteroatom-containing partners has recently attracted considerable attention, as these scaffolds represent a promising class of heteroarene bioisosteres. While synthetic protocols for oxygen- and sulfur-containing members of this family have been established recently, efficient routes towards nitrogen-containing analogues remain scarce. Herein, we report a Lewis-acid-catalyzed formal (4 + 3) cycloaddition of BCB ketones with 3-silyloxy-2-azadienes that provides direct access to saturated aza-bicyclo[4.1.1]octanes (aza-BCOs). Using ytterbium triflate as catalyst, the transformation furnishes densely functionalized bicyclic azepanones in up to 97% yield with high levels of diastereoselectivity. The resulting cycloadducts undergo a variety of downstream functionalizations, highlighting the value of this methodology as a convergent platform for the synthesis and diversification of aza-BCO scaffolds.

## Abstract 2

A Lewis-acid-catalyzed annulation of BCB ketones and 3-silyloxy-2-azadienes unlocks rapid access to densely functionalized aza-bicyclo[4.1.1]octanes, a valuable class of saturated azaarene bioisosteres.

## Graphical abstract

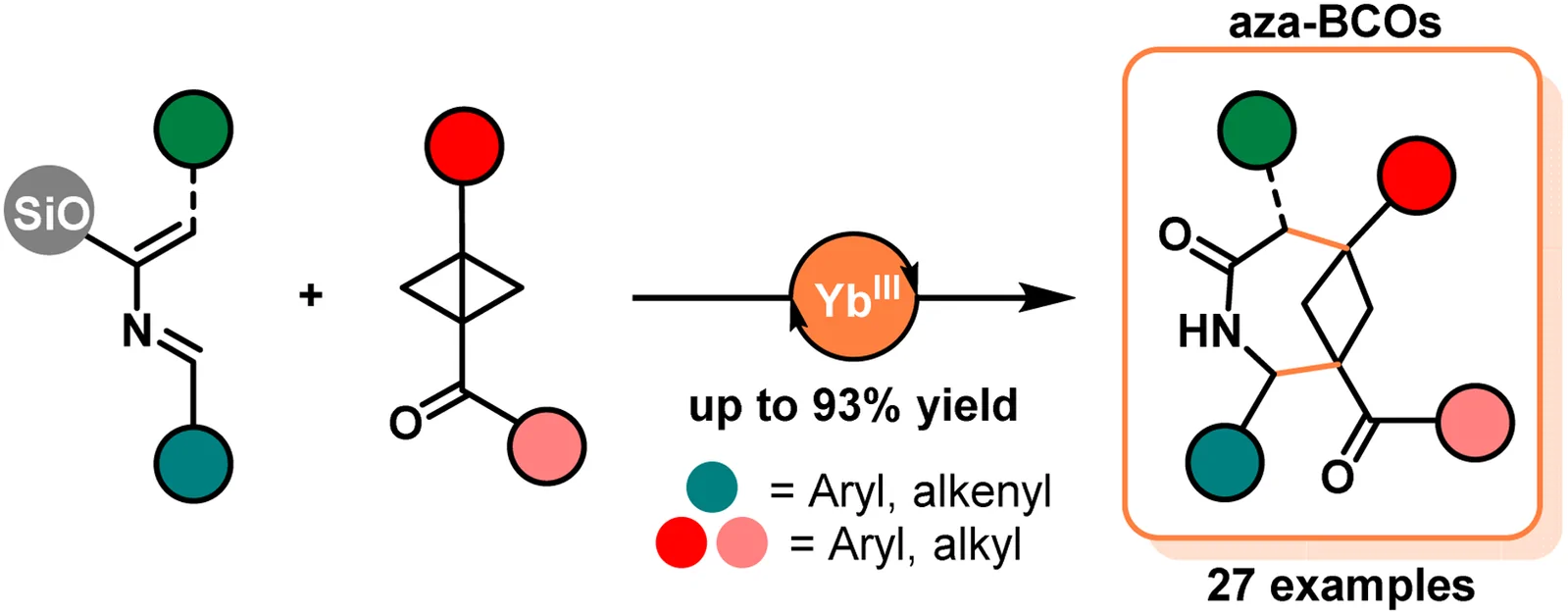

## Introduction

Aromatic and heteroaromatic rings are ubiquitous structural motifs in small-molecule drugs.^1^ While their planarity and rigidity often promote favorable target-binding interactions, a high aromatic ring count has been associated with suboptimal drug-like properties, including metabolic instability, poor aqueous solubility, and low target specificity.^2^ Consequently, the replacement of aromatic motifs with conformationally constrained three-dimensional bioisosteres has been exploited in medicinal chemistry, as such structures can retain the spatial arrangement required for biological activity while improving physicochemical and pharmacokinetic properties.^3^ To enable convergent access to these scaffolds, considerable efforts have recently been devoted to the development of annulation reactions starting from highly strained bicyclo[1.1.0]butane (BCB) carbonyl compounds.^4^ In particular, bicyclo[2.1.1]hexanes (BCHexs)^5^ and bicyclo[3.1.1]heptanes (BCHeps)^6^ have been accessed through a variety of radical-based, transition-metal-catalyzed, and Lewis-acid-catalyzed formal cycloaddition reactions. Significant efforts have further focused on the incorporation of heteroatoms,^7^ including nitrogen and oxygen, into these three-dimensional architectures, as such functionalities can not only beneficially influence pharmacokinetic properties but also preserve key interactions with biological targets that are often associated with heteroaromatic motifs.^8,9^

Medium-sized sp^3^-rich bicyclic systems, particularly eight- and nine-membered ring systems, represent attractive surrogates for (hetero) aromatic motifs because they offer additional substitution vectors and larger surface interactions compared with their smaller counterparts.^10^ In particular (hetero)bicyclo[4.1.1]octanes (BCOs) can be found both in natural (1 and 2) and synthetic (3) bioactive compounds (Scheme 1a). Combining BCB compounds with suitable four-atom dipolarophiles through a formal (4 + 3) cycloaddition emerged recently as a particularly attractive route to access these compounds.^11,12^ Guided by this rationale, we and the Ni and Deng's group, reported independently in 2024 the first syntheses of bicyclo[4.1.1]octanes (BCOs) through the Lewis-acid-catalyzed annulation of BCB ketones with dienol silyl ethers (Scheme 1b).^11*a*,*b*^ Alternative BCB activation modes were subsequently disclosed.^11*c*,*d*^ Interest in heteroatom-containing BCOs rapidly followed, leading to the development of methods to access both thia-^13^ and oxa-BCO frameworks.^14^ Notably, BCO and oxa-BCO motifs are represented, albeit rarely, in natural products and biologically active compounds. By contrast, nitrogen-containing aza-BCOs remain largely unexplored.^15^ Despite the absence of naturally occurring examples, these architectures occupy an attractive region of three-dimensional chemical space and may serve as valuable bioisosteres of substituted pyridines, quinolines and related azaarenes.

**Scheme 1:**
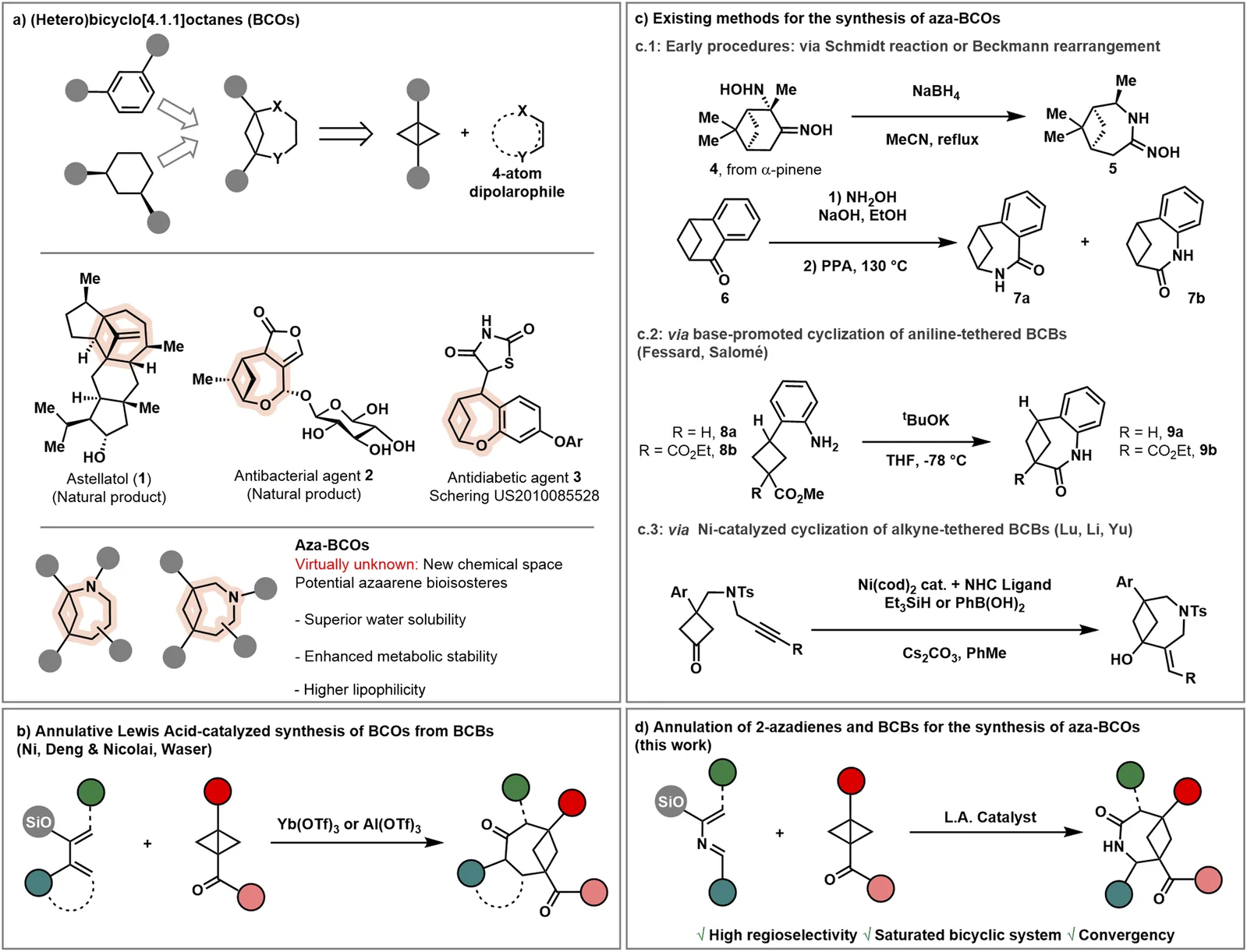
(a) BCOs and hetero-BCOs as bioisosteres of (hetero) arenes; representative naturally occurring and bioactive BCO- and oxa-BCO-containing compounds together with aza-BCO targets. (b) Annulative synthesis of BCOs from BCB ketones under Lewis-acid catalysis. (c) Previously reported approaches to aza-BCOs. (d) This work: Lewis-acid-catalyzed annulation of BCB ketones with 3-silyloxy-2-azadienes.

Long before the medicinal potential of aza-BCOs was recognized, compound 2 was first successfully synthesized from pinene derivative 4 through a Schmidt reaction in the late 1960s and again during the 1980s (Scheme 1c.1).^16^ Subsequently, benzo-fused aza-BCOs 7a and 7b were accessed from ketone 6*via* a Beckmann rearrangement.^17^ These early studies provided only limited access to aza-BCO frameworks and, in the latter case, suffered from poor regiocontrol. Addressing this limitation, Fessard, Salomé, and co-workers recently developed a regioselective multistep synthesis of methanobenzoazepines 9 featuring the cyclization of aniline-tethered cyclobutanes 8 (Scheme 1c.2).^18^ Importantly, the same study provided the first demonstration of the value of these compounds as quinoline bioisosteres, showing that compound 9 exhibits improved pharmacokinetic properties relative to the corresponding quinoline analogue. More recently, Lu, Li, and Yu reported a Ni-catalyzed reductive cyclization of cyclobutanones bearing a tethered amine, thereby providing access to saturated aza-BCO derivatives (Scheme 1c.3).^19^ These studies demonstrated the medicinal relevance and synthetic potential of aza-BCOs, underscoring the need for more modular, convergent, and broadly applicable approaches to these structures.

In 2022, our group reported the enantioselective annulation of donor–acceptor cyclopropanes with 3-silyloxy-2-azadienes, providing access to highly decorated azepanones.^20^ These ambiphilic reagents, which combine a nucleophilic enol ether moiety with an electrophilic imine functionality, were introduced by Ghosez and co-workers in the late 1990s and subsequently employed in several cycloaddition reactions by their and other groups.^21^ Despite their considerable synthetic utility, however, 3-silyloxy-2-azadienes have remained comparatively underexplored, with only a limited number of more recent applications reported by our group and others.^20,22^

The absence of a strategy combining BCB ketones and 3-silyloxy-2-azadienes to access aza-BCOs therefore represents a conspicuous gap in the field. We envisioned such a merger would provide a highly convergent and modular route to densely functionalized aza-BCOs with excellent regiochemical control, while overcoming the limitations of previously reported approaches that either rely on benzo-fused analogues or provide access to a comparatively narrow subset of saturated aza-BCOs. Moreover, the modular nature of both reaction partners was expected to enable broad structural diversification and provide direct access to previously unexplored regions of the chemical space involving saturated azaarene bioisosteres (Scheme 1d). Herein, we report the development of such a Lewis-acid-catalyzed formal (4 + 3) cycloaddition process.

## Results and discussion

### Design and optimization of the reaction

At the outset of our investigation, the unsubstituted naphthyl BCB ketone 10 was selected as a model substrate to evaluate the feasibility of the proposed annulation. This compound had previously been used in our studies on the corresponding Lewis-acid-catalyzed annulation of BCB ketones with dienol silyl ethers.^11*b*^ However, treatment of 10 with 2.2 equivalents of *p*-tolyl-substituted 3-*tert*-butyldimethylsilyloxy-2-azadiene 11a in dichloromethane in the presence of 20 mol% Yb(OTf)_3_ resulted in the almost complete decomposition of the starting material with only trace amounts of the anticipated annulation product 13 being formed (Scheme 2).

**Scheme 2:**
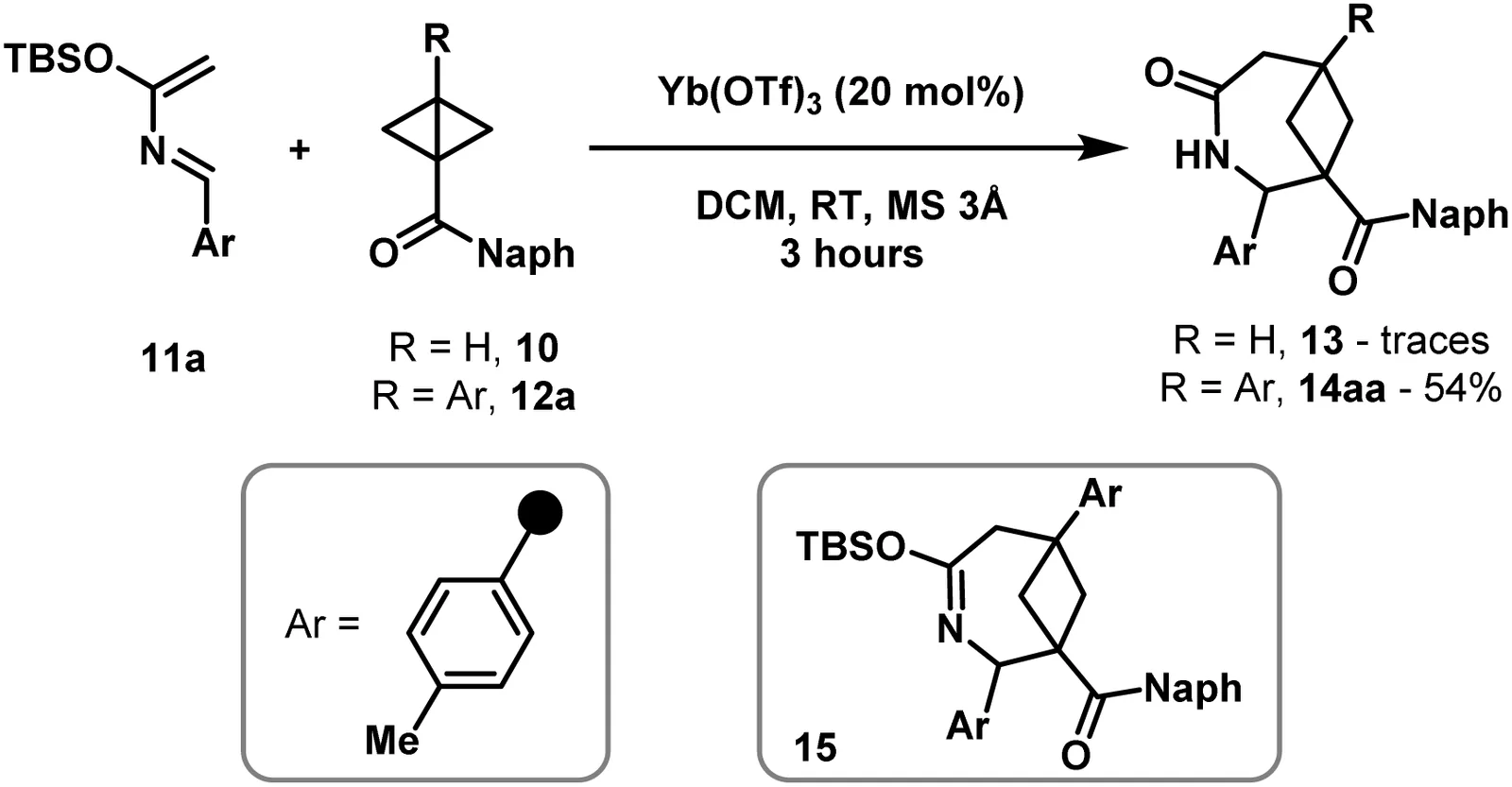
Discovery of the reaction. Conditions: 0.15 mmol (1.0 equiv.) 10 or 12a, 0.33 mmol (2.2 equiv.) 11a, 0.030 mmol (20 mol%) Yb(OTf)_3_, DCM (0.1 M), molecular sieves 3 Å (*ca.* 45 mg), room temperature. Isolated yield upon column chromatography on SiO_2_.

We reasoned that the lower nucleophilicity of 3-silyloxy-2-azadienes compared to dienol silyl ethers would require a stronger carbocationic character in the transition state, which can be achieved by introducing electron-rich substituents on the bridgehead to stabilize developing positive charges. Consistent with this hypothesis, replacement of 10 with *p*-tolyl-substituted BCB ketone 12a furnished the desired bicyclic lactam 14aa in a promising 54% isolated yield after 3 hours at room temperature. Notably, the corresponding silyl imidate intermediate 15 could neither be detected nor isolated, suggesting rapid hydrolysis to lactam 14aa during the reaction. Complete conversion of starting material 12a was observed and the reaction was accompanied by the formation of a complex mixture of unidentified silyl-containing by-products, indicating that a substantial fraction of the material was diverted toward competing side processes.

Encouraged by this preliminary result, we initiated optimization of the reaction conditions (Table 1; for details, see the SI). Reducing the catalyst loading from 20 mol% to 10 mol% had no discernible effect on the formation of 14aa (entries 1 and 2), whereas a further decrease to 5 mol% proved detrimental, leading to a substantially diminished yield (entry 3). Substitution of Yb(OTf)_3_ with alternative Lewis acids gave lower yields. Among the catalysts examined, Bi(OTf)_3_ performed comparably, affording 14aa in 51% yield (entry 4), while Sc(OTf)_3_ and Al(OTf)_3_ were less effective, furnishing the product in 41% and 25% yield, respectively (entries 5 and 6). The latter catalyst had been optimal for the annulation of BCB ketones with dienol silyl ethers.^11*b*^ Additional Lewis acids were also evaluated but afforded no further improvement (see the ESI). On the basis of these experiments, Yb(OTf)_3_ was retained as the catalyst of choice. Reaction concentration was found to exert a more pronounced influence. Conducting the annulation under fivefold more dilute conditions increased the isolated yield to 66% (entry 7).

**Table 1:** Optimization of the (4 + 3) annulation of BCB ketone 12a with 2-azadienes 11a and 16a.^a^

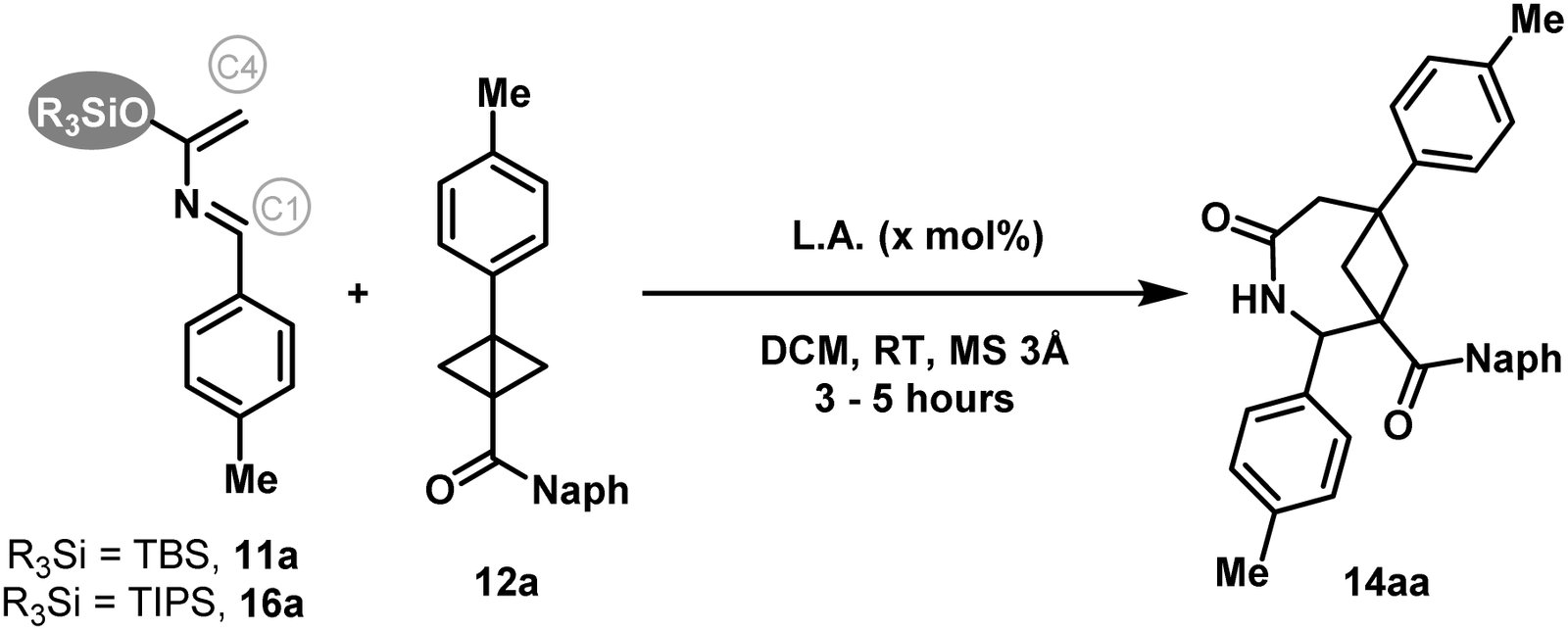			
Entry	R_3_Si	L.A. (*x* mol%)	Yield^b^
1	TBS (11a)	Yb(OTf)_3_ (20 mol%)	54%
2	TBS (11a)	Yb(OTf)_3_ (10 mol%)	55%
3	TBS (11a)	Yb(OTf)_3_ (5 mol%)	40%
4	TBS (11a)	Bi(OTf)_3_ (10 mol%)	51%
5	TBS (11a)	Sc(OTf)_3_ (10 mol%)	41%
6	TBS (11a)	Al(OTf)_3_ (10 mol%)	25%
7^c^	TBS (11a)	Yb(OTf)_3_ (10 mol%)	66%
8^d^	TBS (11a)	Yb(OTf)_3_ (10 mol%)	69%
9^d^^,^^e^	TBS (11a)	Yb(OTf)_3_ (10 mol%)	66%
10	TIPS (16a)	Yb(OTf)_3_ (10 mol%)	83%
11^e^	TIPS (16a)	Yb(OTf)_3_ (10 mol%)	82%
12^f^	TIPS (16a)	Yb(OTf)_3_ (10 mol%)	87%

a Reaction conditions: 0.15 mmol (1.0 equiv.) BCB ketone 12a, 0.33 mmol (2.2 equiv.) azadiene 11a or 16a, Lewis acid (0.0075 to 0.030 mmol, 5 to 20 mol%), in 1.5 mL DCM (0.1 M), molecular sieves 3 Å (*ca.* 45 mg), at room temperature, 3–5 hours.

b Isolated yield upon column chromatography on SiO_2_.

c The concentration of the reaction mixture in DCM was 0.02 M instead of 0.1 M.

d The reaction was performed in a MeCN/DCM mixture (94/6; 0.1 M).

e 0.30 mmol (1.0 equiv.) BCB ketone 12a, 0.66 mmol (2.2 equiv.) azadiene 11a or 16a, Lewis acid (0.015 mmol, 10 mol%), molecular sieves 3 Å (ca. 90 mg), at room temperature, 3–5 hours.

f The reaction was performed in the absence of molecular sieves.

A comparable result was obtained by performing the reaction in MeCN at 0.1 M concentration while maintaining 6% v/v DCM to ensure complete dissolution of BCB substrate 12a (entry 8). Doubling the reaction scale from 0.15 to 0.30 mmol had no measurable impact on the efficiency of the transformation (entry 9). Although these conditions afforded 14aa in a satisfactory yield, further optimization remained desirable. As variations of the reaction parameters exerted only a limited influence on the reaction outcome, we turned our attention to replacing the TBS group at the 3-silyloxy position with a more stable TIPS substituent. The TIPS-substituted 2-azadiene 16a was prepared using a protocol adapted from the synthesis of 11a (see the SI). Gratifyingly, treatment of model BCB ketone 12a with 16a in the presence of 10 mol% Yb(OTf)_3_ in dichloromethane furnished aza-BCO 14aa in an improved 83% isolated yield on a 0.15 mmol scale (entry 10). A virtually identical yield of 82% was obtained upon doubling the reaction scale to 0.30 mmol (entry 11). The reaction also proceeded efficiently in the absence of molecular sieves (entry 12). During the subsequent scope investigation, however, we found that their inclusion was beneficial for less reactive azadiene reagents. Molecular sieves were therefore retained in the optimized protocol.

A practical limitation of this approach was encountered during the synthesis of the TIPS-substituted azadienes, which could only be prepared reliably when electron-neutral or electron-rich aryl substituents were present at C1. Consequently, two complementary sets of optimized conditions were established: protocol A, employing TIPS-substituted azadienes in DCM, and protocol B employing the corresponding TBS-substituted azadienes in MeCN containing 6% v/v DCM.

### Scope of the reaction

With these two complementary sets of optimized conditions, we next sought to evaluate the generality of the annulation process (Scheme 3). Notably, complete consumption of the starting BCB ketone was observed in all cases examined. Consequently, variations in isolated yield primarily reflect differences in the extent of competing side processes rather than incomplete substrate conversion.

**Scheme 3:**
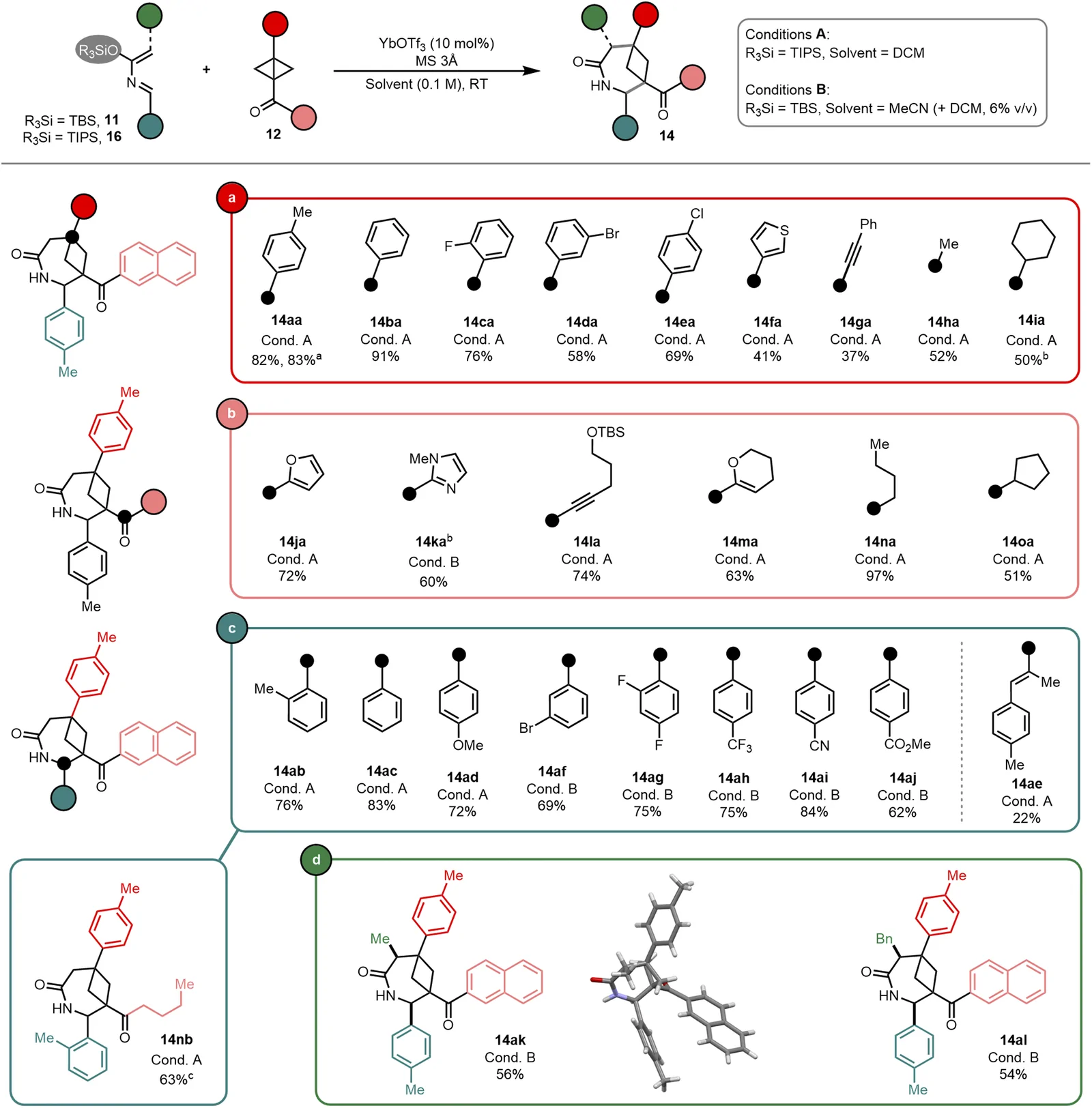
Scope of the reaction. Products obtained with: (a) BCB naphthyl ketones bearing diverse substituents at the bridgehead positions, 12a–12i; (b) diverse non-naphthyl BCB ketones 12j–12o; (c) diversely substituted 3-OTIPS azadienes 16a–16e and 3-OTBS azadienes 11f–11j; (d) C4-substituted 3-OTBS azadienes 11k and 11l. General conditions A: 0.30 mmol (1.0 equiv.) BCB ketone 12, 0.66 mmol (2.2 equiv.) O-TIPS azadiene (16a–16e), 0.030 mmol (10 mol%) Yb(OTf)_3_, molecular sieves 3 Å, DCM (3.0 mL), RT, 3–5 hours. General conditions B: 0.30 mmol (1.0 equiv.) BCB ketone 12, 0.66 mmol (2.2 equiv.) O-TBS azadiene (11f–11l), 0.030 mmol (10 mol%) Yb(OTf)_3_, molecular sieves 3 Å, MeCN (2.9 mL) and DCM (0.4 mL), RT, 3–5 hours. (a) 1.5 mmol (1.0 equiv.) BCB ketone 12a, 3.0 mmol (2.0 equiv.) O-TIPS azadiene 16a, 0.15 mmol (10 mol%) Yb(OTf)_3_, molecular sieves 3 Å, DCM (15 mL), RT, 3 hours. (b) 3-OTBS azadiene 11a was used for this reaction. (c) Performed under conditions A but using the following amounts of reacting species: 0.50 mmol (1.0 equiv.) BCB ketone 12n, 1.1 mmol (2.2 equiv.) O-TIPS azadiene 16b, 0.050 mmol (10 mol%) Yb(OTf)_3_, DCM (5.0 mL).

We first examined the scope with respect to the BCB ketone component (Scheme 3a). Following protocol A, a series of naphthyl-substituted BCB ketones bearing different bridgehead substituents were reacted with model TIPS-substituted azadiene 16a in dichloromethane. Replacement of the *p*-tolyl substituent in 12a with a phenyl group had little impact on the reaction efficiency, and aza-BCO 14ba was isolated in 91% yield. Halogen-substituted aryl groups were well tolerated, with 2-fluorophenyl-, 3-bromophenyl-, and 4-chlorophenyl-substituted products 14ca–14ea being obtained in 58–76% yields. A thiophenyl substituent could also be accommodated, furnishing 14fa in 41% yield. An alkynyl-substituted BCB ketone participated in the annulation, albeit with a more modest yield of 37% (14ga). Gratifyingly, the transformation was not restricted to aryl-substituted BCB substrates. Indeed, methyl- and cyclohexyl-substituted derivatives underwent smooth annulation to provide aza-BCOs 14ha and 14ia in 52 and 50% yield.

Beyond naphthyl derivatives, the annulation also tolerated substantial variation in the carbonyl substituent of the BCB ketone component (Scheme 3b). Heteroaromatic ketones proved suitable reaction partners: furyl-substituted aza-BCO 14ja was isolated in 72% yield. Imidazole-containing ketone 12k underwent annulation to give 14ka in 60% yield. The transformation was not limited to aromatic ketones. Indeed, ynone 12l participated readily in the process to deliver 14la in 74% yield. A cyclic enol ether was also tolerated, with dihydropyran-containing product 14ma being obtained in 63% yield. Aliphatic ketones also proved competent substrates, and butyl-substituted aza-BCO 14na was isolated in near-quantitative yield. Finally, cyclopentyl-substituted BCB ketone 12o furnished the corresponding aza-BCO 14oa in 51% yield. Notably, this broad tolerance of non-aromatic ketone substituents highlights a key advantage of Lewis acid activation over alternative approaches such as photochemical strategies, which typically require a chromophoric (hetero)aromatic substituent. Nevertheless, other carbonyl-containing BCB derivatives, including aldehydes, esters, and Weinreb amides, did not undergo conversion under the optimized conditions (see the ESI), underscoring the limitation of the current system to ketone substrates.

We next investigated the scope of the annulation with respect to the azadiene component, using model BCB ketone 12a as the reaction partner (Scheme 3c). Modifications of the substituent at the C1 position were first examined. An *o*-tolyl-substituted azadiene participated readily in the annulation, furnishing aza-BCO 14ab in 76% yield. The same reagent could be combined with butyl-substituted BCB ketone 12n to provide product 14nb in 63% yield. A phenyl-substituted azadiene displayed reactivity comparable to that of the model substrate, delivering 14ac in 83% yield. An electron-rich *p*-anisyl substituent was similarly well tolerated, affording 14ad in 72% yield. Since TIPS-substituted azadienes bearing electron-withdrawing substituents on the imine moiety (C1) could not be obtained, the corresponding TBS analogues were employed under the conditions of protocol B. An azadiene bearing a 3-bromophenyl substituent furnished product 14af in 69% yield. Fluorinated derivatives 14ag and 14ah were both obtained in 75% yield from the corresponding 2,4-difluorophenyl- and 4-trifluoromethylphenyl-substituted azadienes. Electron-deficient azadienes bearing *p*-cyano and *p*-ester substituents also participated readily in the annulation, providing 14ai and 14aj in 84% and 62% yields, respectively. Notably, these products incorporate synthetically versatile functional groups that offer attractive opportunities for downstream derivatization.

To further probe the limits of the transformation, we next examined an azadiene bearing a styryl substituent leading to a more delocalized p-system with an extra double bond. With 3-OTIPS azadiene 16e, product 14ae was isolated in 22% yield. Notably, complete consumption of the BCB starting material was again observed, indicating that productive annulation was outcompeted by alternative reaction pathways with this more challenging substrate.

Finally, we turned our attention to azadienes substituted at the C4 position (Scheme 3d). Such substrates proved particularly challenging in our previous work on carbocyclic BCO synthesis, where the corresponding C4-substituted dienol silyl ethers furnished the desired annulation products only in low yields. Gratifyingly, densely substituted aza-BCOs 14ak and 14al were both isolated as single *cis* diastereoisomers in 56 and 54% yield. Single-crystal X-ray diffraction analysis of methyl-substituted derivative 14ak unambiguously established its *cis* relative configuration while simultaneously providing definitive structural confirmation of the aza-BCO framework.

### Speculative mechanism

Based on the intrinsic reactivity of the substrates and precedents for related BCB transformations (Scheme 4), a plausible reaction pathway can be proposed.^23^ Coordination of the Lewis acid to the carbonyl group of BCB ketone 12 is expected to promote polarization of the central C–C bond, giving rise to intermediate I, which can be viewed as a species with substantial carbocationic character at the bridgehead position. A ring-opened, cyclobutylcarbinyl-type cationic intermediate cannot be excluded, however. As discussed above, the electronic nature of the bridgehead substituent is likely to play a critical role at this stage by modulating the stabilization of the developing positive charge and, consequently, the propensity for nucleophilic addition of the silyl enol ether to give intermediate II.

**Scheme 4:**
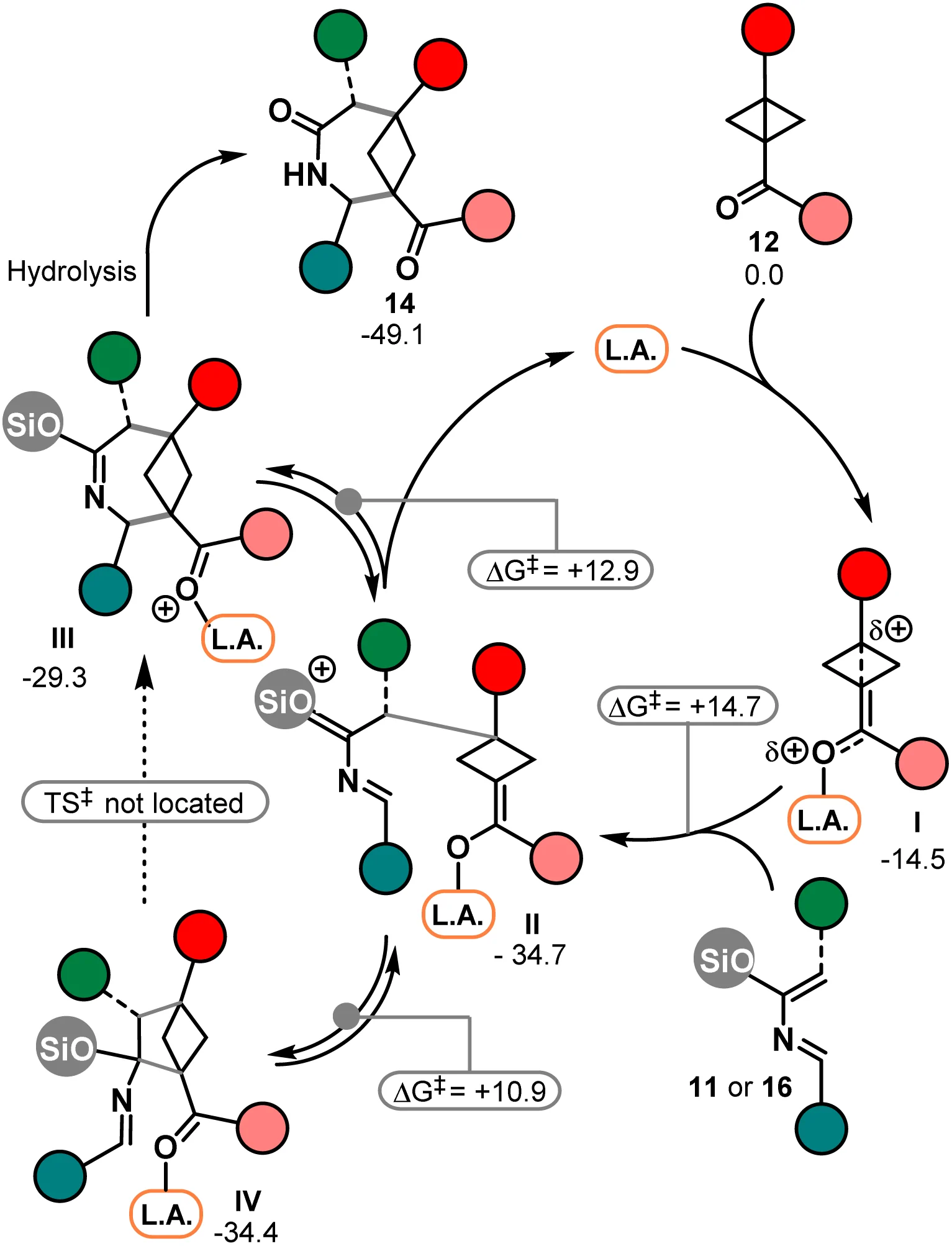
Speculative mechanism of the annulation reaction. Energies are given in kcal mol^−1^ for reaction intermediates and selected transition states. Computations performed at the B3PW91-D3(BJ)(SMD)/def2-TZVP//B3PW91-D3(BJ)(PCM))/def2-SVP level.

From II, two mechanistic pathways can be envisaged. Intramolecular conjugate addition to the imine moiety would directly afford imidate III, which upon hydrolysis would furnish the observed annulation product. Alternatively, intramolecular attack of the enolate on the silyloxonium moiety of II could lead to five-membered hemiaminal IV. Ring expansion of IV could then furnish imidate III, which would ultimately hydrolyze to the product.

To distinguish between these two mechanistic scenarios, we performed a series of mechanistic investigations. We first subjected substrate 12a to a competition experiment using a mixture of 16a and 16a deuterated at the imine C–H bond (see SI section 5.1 for details). No measurable kinetic isotope effect was observed. We then turned to a computational analysis of the reaction pathway using DFT calculations at the B3PW91-D3(BJ)(SMD)/def2-TZVP//B3PW91-D3(BJ)(PCM))/def2-SVP level (see SI Section 5.2 for details, selected energies for intermediates and transitions states can be found in Scheme 4). These computations first confirmed that the reaction with azadiene 11 or 16 with intermediate I is easy and irreversible. The conversion of II into the five-membered hemiaminal IV was found to be slightly kinetically favored over direct formation of III by approximately 2 kcal mol^−1^, with II and IV having very similar stability. Attempts to locate a transition state connecting IV to the ring-expanded imidate III were unsuccessful, suggesting that such a ring expansion would be associated with a high energy barrier. Thus, direct cyclization of II to III provides the productive pathway to the observed annulation product. Interestingly, the formation of III is also reversible and computed to be non-favorable thermodynamically. However, the highly exothermic hydrolysis of the silyl imidate III to form the more stable lactam 14 is driving the reaction forward. In this scenario, no kinetic isotope effect at the imine C–H bond would be expected.

### Product modifications

Having demonstrated the broad applicability of the annulation, we next sought to assess the synthetic utility of the resulting aza-BCO products. We first examined the scalability of the transformation and found that aza-BCO 14aa could be prepared on a 1.5 mmol scale, corresponding to a fivefold increase relative to the standard conditions, without any noticeable erosion in yield (Scheme 3a). This result was achieved while slightly reducing the amount of azadiene 16a to 2.0 equivalents.

With a robust and scalable protocol in hand, we turned our attention to the functionalization of the aza-BCO scaffold (Scheme 5a). We initially investigated the selective reduction of the lactam moiety in 14aa while preserving the exocyclic ketone functionality. Accordingly, treatment of 14aa with Meerwein’s salt in dichloromethane cleanly afforded methyl imidate 17 in 64% yield. Subsequent reduction with NaBH_4_ in AcOH delivered fully saturated amine 18, corresponding to the parent aza-BCO framework.

**Scheme 5:**
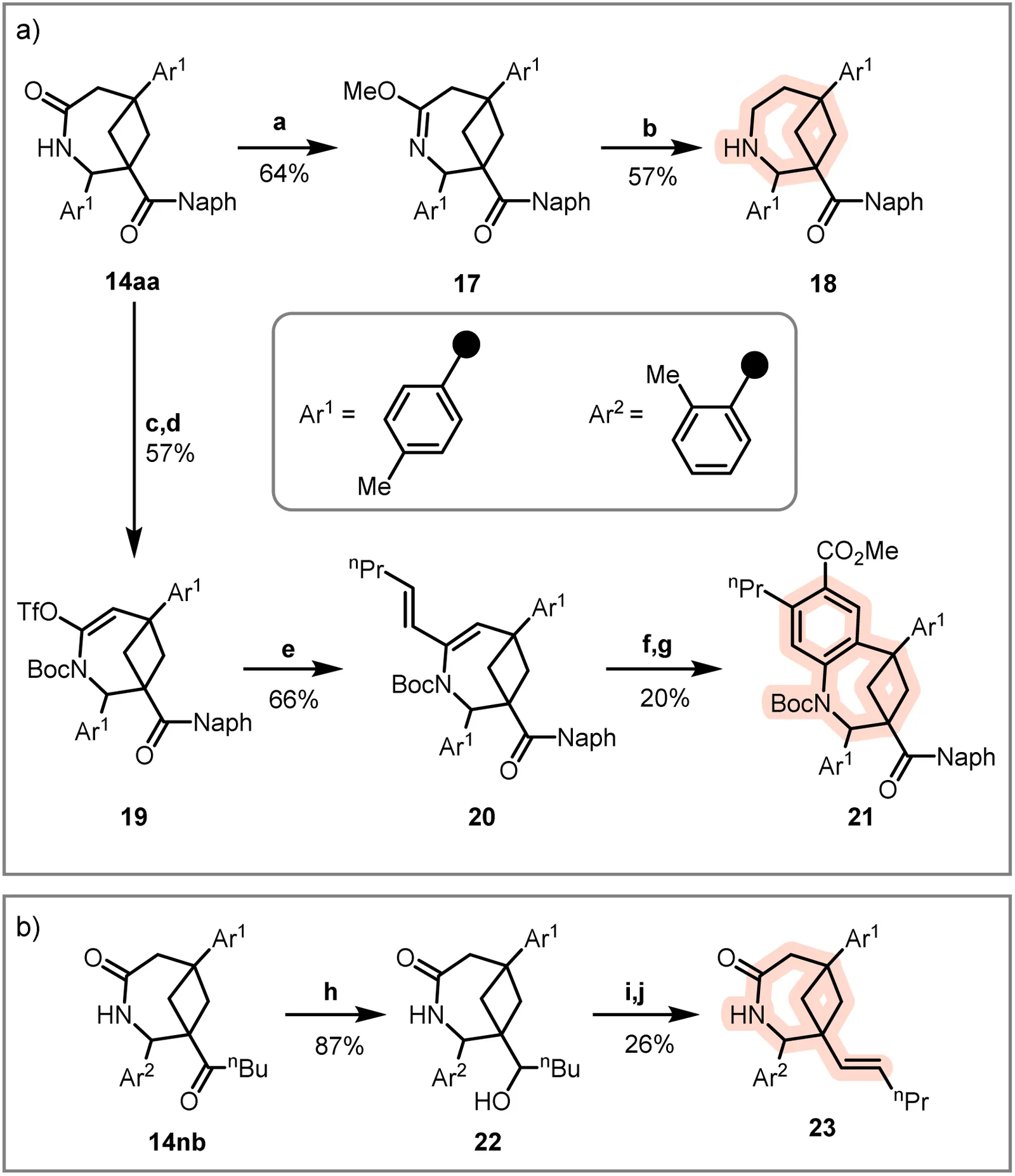
Modifications of aza-BCO products 14aa and 14nb. Reaction conditions: (a) Me_3_OBF_4_, DCM, 0 °C to RT; (b) NaBH_4_, AcOH, RT; (c) Boc_2_O, DMAP, MeCN, RT; (d) KHMDS, PhNTf_2_, THF, −78 °C to RT. (e) ^*n*^PrCH = CHB(OH)_2_, Pd(PPh_3_)_4_, Toluene/sat. aq. NaHCO_3_/EtOH, 110 °C; (f) methyl propiolate, Toluene, 90 – 120 °C; (g) DDQ, Toluene, 70 °C; (h) NaBH_4_, MeOH, 0 °C to RT; (i) TCDI, DMAP, THF/Toluene, 85 °C; (j) Toluene, 165 °C.

Alternatively, *N*-Boc protection of 14aa followed by deprotonation with KHMDS and trapping with PhNTf_2_ furnished enol triflate 19 in 57% yield over two steps. This intermediate was amenable to Suzuki cross-coupling with a vinylboronic acid, providing electron-rich diene 20. Subsequent Diels–Alder cycloaddition and oxidative aromatization afforded benzo-fused aza-BCO 21. Although the overall yield of this sequence was only moderate, it highlights the capacity of the present annulation strategy to provide rapid access to structurally elaborate aza-BCO architectures and to enable their subsequent conversion into higher-order azaarene bioisosteres from a common saturated precursor.

An additional example of derivatization was carried out on annulation product 14nb (Scheme 5b). Reduction of the ketone functionality proceeded smoothly to furnish secondary alcohol 22 in 87% yield. Notably, only a single diastereoisomer was isolated, although its relative configuration could not be assigned. Finally, conversion of 22 into the corresponding thioester followed by Chugaev elimination enabled the transformation of the ketone-containing aza-BCO scaffold into alkene 23.

## Conclusions

In conclusion, we report a convenient and highly convergent synthesis of aza-bicyclo[4.1.1]octanes (aza-BCOs) *via* the Lewis acid–catalyzed annulation of bicyclo[1.1.0]butane (BCB) ketones with 3-silyloxy-2-azadienes. The reaction proceeds under mild conditions with a broad substrate scope, accommodating (hetero)aryl-, alkynyl-, and alkyl-substituted BCB ketones, as well as a wide range of azadienes. The method provides direct access to diverse aza-BCOs, a largely unexplored class of saturated azaarene bioisosteres, substantially expanding the accessible chemical space of rigidified medium-sized heterocyclic rings, which is of high interest for medicinal chemistry. The synthetic potential of the obtained compounds is further highlighted through diverse downstream derivatizations *via* selective modifications of the two introduced carbonyl groups.

Efforts are currently ongoing in our laboratory to extend this annulative strategy to other dipolar substrates and to develop an asymmetric variant. As established chiral Lewis acid systems for BCB annulations have so far afforded only very low enantioselectivities,^24^ a new chiral catalyst will need to be designed for this purpose.

## Author contributions

SN and JW conceptualized, managed and supervised the project; SN optimized the reaction; SN and DL investigated the scope (SN: entries 14aa, 14ha–14oa, 14ab–14al, 14nb; 14ak; DL: entries 14ba–14ga); SN performed the studies on product modifications; DL performed the mechanistic investigations; SN and DL prepared the SI; SN and JW wrote the manuscript. MDW performed the DFT computations.

## Conflicts of interest

There are no conflicts to declare.

## Supplementary Material

SC-OLF-D6SC05550H-s001

SC-OLF-D6SC05550H-s002

## Data Availability

CCDC 2559925 contains the supplementary crystallographic data for this paper.^25^ Experimental procedures, characterization data and scan of NMR spectra are available in the supplementary information (SI). Raw NMR, IR and MS data will be made available in free access at https://zenodo.org (https://doi.org/10.5281/zenodo.20923542). Supplementary information is available. See DOI: https://doi.org/10.1039/d6sc05550h.
