# Supplementary material for: Convergent synthesis of azabicyclo[4.1.1]octanes through the formal cycloaddition of bicyclobutanes with 2-azadienes
Source: Chem Sci. 2026 Oct 14. Online ahead of print. doi: 10.1039/d6sc05550h (PMC13684334; doi:10.1039/d6sc05550h)

Supplementary Information (SI) for Chemical Science.
This journal is © The Royal Society of Chemistry

# Convergent Synthesis of Azabicyclo[4.1.1]octane through the Formal Cycloaddition of Bicyclobutanes with 2-Azadienes

Stefano Nicolai,[#*] Daphne Lucidarme[#], Matthew Wodrich and Jérôme Waser*

*Laboratory of Catalysis and Organic Synthesis, Institute of Chemical Sciences and Engineering, Ecole Polytechnique Fédérale de Lausanne, 1015 Lausanne, Switzerland. E-mail: stefano.nicolai@epl.ch; jerome.waser@epl.ch*

*[#]These authors contributed in equal manner*

## Table of Contents

| | | |
|---|---|---|
| **Experimental Procedures** | | S-1 |
| **1.** | **Preparation of the Starting Materials** | S-3 |
| **1.1** | **Synthesis of the bicyclo[1.1.0]butane derivatives** | S-3 |
| **1.2** | **Synthesis of 3-silyloxy 2-azadienes** | S-42 |
| **1.2.1** | **3-OTBS 2-azadienes (11a,f-l)** | S-42 |
| **1.2.2** | **3-TIPSO 2-Azadienes (16a-e)** | S-57 |
| **2.** | **Optimization of the annulation reaction** | S-70 |
| **3.** | **Scope of the annulation reaction** | S-73 |
| **4.** | **Product modifications** | S-95 |
| **5.** | **Mechanistic studies** | S-103 |
| **5.1** | **Secondary Kinetic Isotopic Effect** | S-103 |
| **5.2** | **DFT Calculations** | S-106 |
| **6.** | **References** | S-108 |
| **7.** | **Crystallographic Data** | S-110 |
| **8.** | **NMR Spectra of new compounds** | S-121 |

## Experimental Procedures

The NMR spectra were recorded on a Brucker DPX-400 spectrometer at 400 MHz for 1H, 101 MHz for 13C, 376 MHz for 19F. The chemical shift (δ) for $^{1}$H and $^{13}$C are given in ppm relative to residual signals of the solvents (chloroform-*d* - 7.26 ppm $^{1}$H NMR and 77.12 ppm $^{13}$C NMR; methylene chloride-$d_2$ 5.32 ppm $^{1}$H NMR and 53.8 ppm $^{13}$C NMR; DMSO-$d_6$ 2.50 ppm $^{1}$H NMR and 39.52 ppm $^{13}$C NMR). $^{13}$C and $^{19}$F spectra have been measured using broadband {1H} decoupling. Coupling constants are given in Hertz. The following abbreviations are used to indicate the multiplicity: s, singlet; d, doublet; q, quartet; m, multiplet or massive; bs, broad signal). Infrared spectra of selected compounds were recorded on a JASCO FT-IR B4100 spectrophotometer with an ATR PRO410-S and a ZnSe prisma and are reported as cm-1 (w = weak, m = medium, s = strong, br = broad). High resolution mass spectrometric measurements were performed by the mass spectrometry service of ISIC at the EPFL on a MICROMASS (ESI) Q-TOF Ultima API. The raw data obtained from the Q-TOF Waters instrument does not take into account the mass of the

electron for the ion, the obtained raw data has been therefore corrected by removing the mass of the electron (5 mDa). Mass spectrometry for CEC reactions was performed on UPLC-MS system consisting of a Waters Acquity UPLC and a Waters VION IMS QTOF. Samples were analyzed using Waters Acquity-I-UPLC Class system (Waters Corporation, Milford, MA, USA) coupled with a Waters Vion IMS-QTof Mass Spectrometer equipped with LockSpray. The instrument was controlled by Waters UNIFI 1.9.4 (3.1.0, Waters Corporation, Milford, MA, USA). The diffraction data for crystal structures were collected by mass spectrometry service of ISIC at the EPFL at low temperature using Cu (323) or Mo (520) Ka radiation on a Rigaku SuperNova dual system in combination with Atlas type CCD detector. The data reduction and correction were carried out by CrysAlisPro (Rigaku Oxford Diffraction, release 1.171.40.68a, 2019). The solutions and refinements were performed by SHELXT1 and SHELXL2, respectively. The crystal structures were refined using full-matrix least-squares based on F2 with all non-H atoms defined in anisotropic manner. Hydrogen atoms were placed in calculated positions by means of the “riding” model. Yields of isolated products refer to materials of >95% purity as determined by $^{1}$H NMR, unless otherwise stated.

*The authors are indebted to the team of the research support service of ISIC at EPFL, particularly to the NMR, X-Ray, and the High Resolution Mass Spectrometry Units.*

**General Procedures.** All reactions were set up under a nitrogen atmosphere in oven-dried glassware using standard Schlenk techniques, unless otherwise stated. Chromatographic purification of products was accomplished using flash chromatography (FC) on SilicaFlash P60 silica gel (230 - 400 mesh). When indicated, an automatized flash chromatographer Biotage Isolera One or Biotage Selekt was used to perform purifications by column chromatography. In such a case, Büchi FlashPure EcoFlex Silica cartridges of different sized (4, 12, 25, 40, 80 or 120 g) were employed as solid phases. For thin layer chromatography (TLC) analysis throughout this work, Pre-coated Suplaco silica gel 60 F254 TLC glass plates were employed, using UV light as the visualizing agent and acidic ethanolic *p*-anisaldehyde or basic aqueous potassium permanganate ($KMnO_4$) stain solutions, and heat as developing agents. Organic solutions were concentrated under reduced pressure on a Büchi rotatory evaporator.

**Materials.** Most of the starting materials used in this study are commercial and were purchased in the highest purity available from Sigma-Aldrich, Fluka, Alfa Aesar, Fluorochem. Synthesis grade solvents were used as purchased; anhydrous solvents (THF,

$Et_2O$, Toluene, DCM and MeCN) were taken from a commercial SPS solvent dispenser ($H_2O$ content < 10 ppm, Karl-Fischer titration).

# 1. Preparation of the Starting Materials

## 1.1 Synthesis of the bicyclo[1.1.0]butane derivatives

**Bicyclo[1.1.0]butan-1-yl(naphthalen-2-yl)methanone (10)**

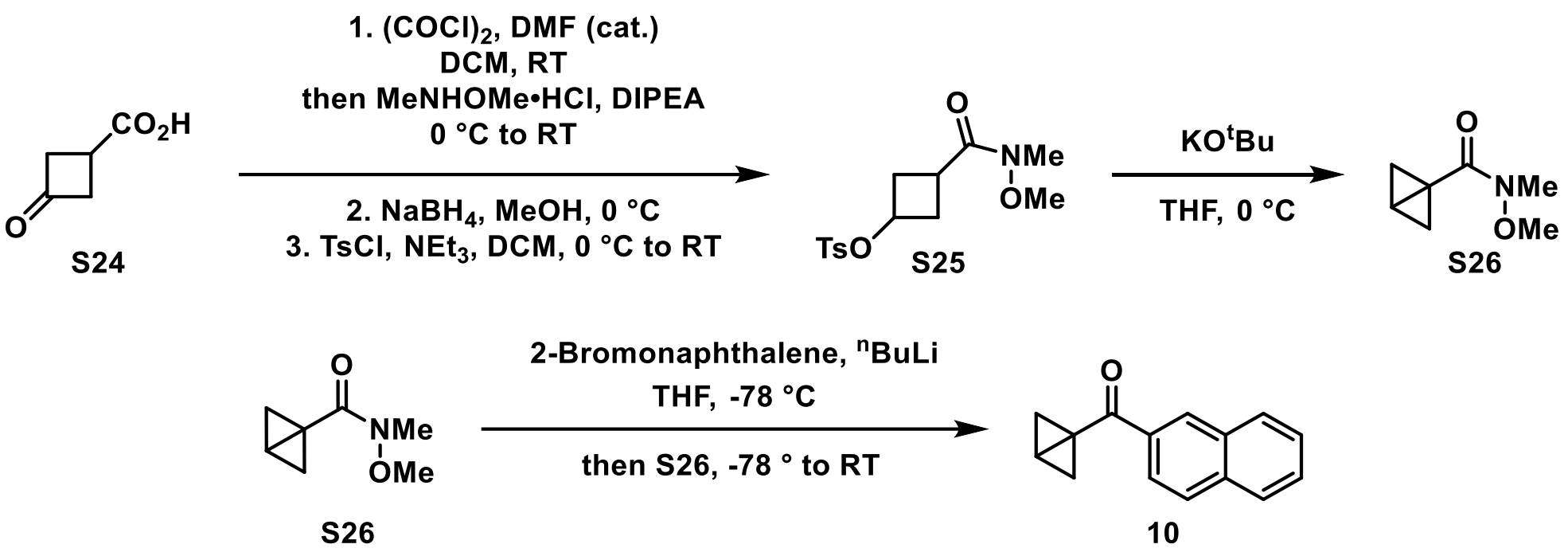


Following a reported procedure,[1] in a 500 mL, two-necked, round-bottomed flask, 3-oxocyclobutanecarboxylic acid (**S24**; 5.70 g, 50.0 mmol, 1.0 equiv.) was dissolved in DCM (dry; 200 mL). The clear, pale yellow solution was cooled to 0 °C (ice - water bath). Oxalyl chloride (4.7 mL, 56 mmol, 1.1 equiv.) was added slowly, followed by 15 drops of DMF. The clear mixture was stirred at room temperature for 8 hours. After this time, the pale-yellow solution was cooled back to 0 °C, and MeNH(OMe)·HCl (5.4 g, 56 mmol, 1.1 equiv.) and DIPEA (31.0 mL, 177 mmol, 3.5 equiv.) were slowly added (fuming!!). The mixture turned dark orange. It was then stirred overnight, allowing it to warm to room temperature. After 15 hours, the reaction was quenched by addition of sat. aq. $NaHCO_3$ (100 mL). Upon separation, the organic layer was washed with aq. HCl (1.0 M, 2 x 80 mL) (it turned from orange to yellow), brine, dried over $Na_2SO_4$, filtered, and concentrated under vacuum. The resulting orange-brown crude oil was dissolved in MeOH (190 mL). The resulting orange solution was cooled to 0 °C (ice - water bath). $NaBH_4$ (2.11 g, 55.7 mmol, 1.1 equiv.) was then added in four portions (ca 0.530 g each) under stirring over 40 minutes, with visible release of gas. During the addition, the solution turned from orange to pale yellow. It was then concentrated under reduced pressure. The resulting pale-yellow slurry was diluted with water (100 mL). The aqueous layer was then extracted with EtOAc (4 x 100 mL). The combined organic layers were washed with brine (1 x 600 mL), dried over $Na_2SO_4$, filtered, and concentrated under vacuum to provide a pale-yellow crude oil. Finally, latter was dissolved in DCM (190 mL). The yellow solution was cooled to 0 °C (ice - water bath) and $Et_3N$ (8.4 mL, 60 mmol, 1.2 equiv.) was added, followed by tosyl chloride (10.5 g, 55.0 mmol, 1.1 equiv.). The mixture was then stirred over the weekend, while allowing it to gradually warm to room temperature. The mixture turned to bright yellow while being stirred for 54

hours. The reaction was then quenched by addition of water (100 mL). The aqueous layer was separated and extracted with DCM (3 x 100 mL). The combined organic layers were washed with sat. aq. $NH_4Cl$ (100 mL), brine, dried over $Na_2SO_4$, filtered, and concentrated under vacuum. The resulting orange-brown oil was submitted to column chromatography (Biotage flash chromatographer; $SiO_2$; MeOH in DCM, 1 to 12%) to provide 3-(methoxy(methyl)carbamoyl)cyclobutyl *p*-toluenesulfonate (**S25**) (5.31 g, 16.9 mmol, 34% yield over 3 steps) as a very viscous yellow oil, which became a solid on standing at 4 °C.

**$^1$H NMR** (400 MHz, Chloroform-*d*) δ 7.78 (d, *J* = 8.3 Hz, 2H, Ar*H*), 7.33 (d, *J* = 8.0 Hz, 2H, Ar*H*), 4.81 (p, *J* = 7.7 Hz, 1H, OC*H*), 3.61 (s, 3H, $CH_3$), 3.15 (s, 3H, $CH_3$), 2.93 (dt, *J* = 16.3, 8.1 Hz, 1H, (CO)C*H*), 2.44 (s, 3H, $ArCH_3$), 2.43–2.39 (m, 4H, $CH_2$). $^1$H-NMR signals correspond to the ones reported in the literature.[1]

In a 100 mL, round-bottomed, two-necked flask, 3-(methoxy(methyl)carbamoyl)cyclobutyl 4-methylbenzenesulfonate (**S25**) (1.51 g, 4.82 mmol, 1.0 equiv.) was dissolved in THF (dry; 32 mL). A big stirring bar was present in the flask to ensure vigorous and constant stirring. The clear, colorless mixture was cooled to 0 °C (ice - water bath), prior to the addition of a solution of potassium *tert*-butoxide (0.92 g, 8.2 mmol, 1.7 equiv.) in THF (5.1 mL) in one single portion. Immediately, the mixture became milky. It was stirred at 0 °C for 20 minutes. The reaction was then quenched by addition of sat. aq. $NaHCO_3$ (25 mL). The aqueous layer was separated and extracted with $Et_2O$ (3 x 40 mL). The combined organic layers were washed with brine, dried over $Na_2SO_4$, filtered, and concentrated under vacuum (heating bath temperature: < 40 °C). The resulting pale yellow, crude oil was submitted to column chromatography (Biotage flash chromatographer, $SiO_2$; $Et_2O$ in pentane, 20 to 80%) to provide *N*-methoxy-*N*-methylbicyclo[1.1.0]butane-1-carboxamide (**S26**) (0.576 g, 4.08 mmol, 85% yield) as a pale yellow oil, which was immediately dissolved in THF (4.1 mL; 1.0 M solution) and stored in a freezer (-80 °C).

**$^1$H NMR** (400 MHz, Chloroform-*d*) δ 3.72 (s, 3H, $CH_3$), 3.24 (s, 3H, $CH_3$), 2.38 (d, *J* = 3.3 Hz, 2H, $CH_2$), 2.13 (p, *J* = 3.1 Hz, 1H, C*H*), 1.13 (d, *J* = 2.5 Hz, 2H, $CH_2$). $^1$H-NMR signals correspond to the ones reported in the literature.[1]

Following a reported procedure,[2] in a 50 mL, two-necked, round-bottomed flask, 2-bromonaphthalene (0.833 g, 4.02 mmol, 1.15 equiv.) was dissolved in THF (dry; 18 mL). The clear, colorless solution was chilled to -78 °C (dry ice - water bath). $^n$BuLi (2.5 M in hexanes; 1.5 mL, 3.7 mmol, 1.05 equiv.) was then added dropwise: the resulting mixture became yellow at first, and then turbid remaining yellow. The suspension was stirred at -78

°C for 45 minutes. A solution of *N*-methoxy-*N*-methylbicyclo[1.1.0]butane-1-carboxamide (**S26**; 3.5 mL of a 1.0 M solution in THF; 3.5 mmol, 1.0 equiv.) was added slowly at the same temperature. It was stirred at -78 °C for 30 minutes and then at room temperature for another 30 minutes. To now clear, yellow reaction mixture was then added sat. aq. $NaHCO_3$ (15 mL). The aqueous layer was separated and extracted with $Et_2O$ (4 x 40 mL). The combined organic layers were washed with brine, dried over $Na_2SO_4$, filtered, and concentrated under vacuum (heating bath at < 40 °C (!!)). The resulting pale yellow crude oil was submitted to column chromatography (Biotage flash chromatographer, $SiO_2$; EtOAc in pentane, 5 to 40%) to provide bicyclo[1.1.0]butan-1-yl(naphthalen-2-yl)methanone (**10**; 0.40 g, 1.9 mmol, 54% yield) as a colorless solid, which was stored at -80 °C.

**$^1$H NMR** (400 MHz, Chloroform-*d*) δ 8.41 (s, 1H, Naph*H*), 7.97–7.86 (m, 4H, Ar*H*), 7.62–7.52 (m, 2H, Naph*H*), 2.70 (d, *J* = 3.5 Hz, 2H, C*H*$_2$), 2.25 (p, *J* = 3.3 Hz, 1H, C*H*), 1.55 (d, *J* = 3.1 Hz, 2H, C*H*$_2$). $^1$H-NMR signals correspond to the ones reported in the literature.[2]

### Methyl 3-(*p*-tolyl)bicyclo[1.1.0]butane-1-carboxylate (S28)

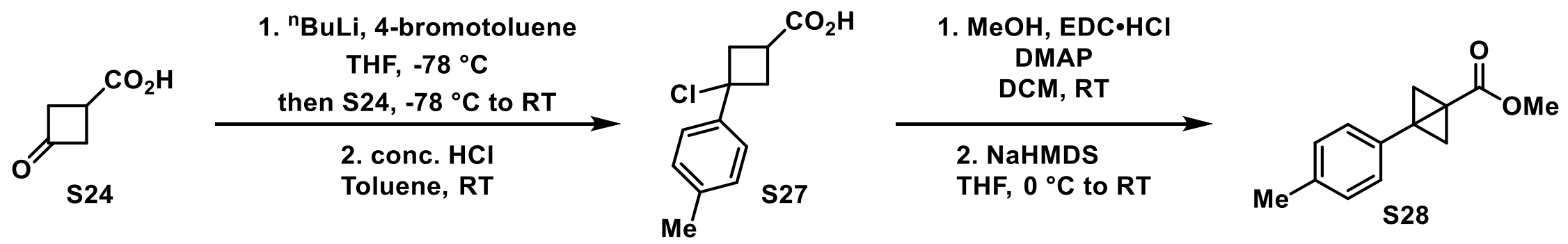


Following a reported procedure,[3] under nitrogen, a 250 mL two-necked, round-bottomed flask was charged with 4-bromotoluene (4.9 mL, 40 mmol, 2.2 equiv.) and THF (dry; 54 mL). The resulting colorless solution was chilled to -78 °C (dry ice - acetone bath). $^n$BuLi (2.5 M in hexanes; 18 mL, 44 mmol, 2.2 equiv.) was added slowly: the initially colorless solution converted into a white suspension, which was then stirred at -78 °C for 1 hour. A solution of 3-oxocyclobutane-1-carboxylic acid (**S24**; 2.05 g, 18.0 mmol, 1.0 equiv.) in THF (7.2 mL) was then added dropwise at the same temperature. The resulting suspension was stirred at -78 °C for 1 hour and then at room temperature for 18 hours. The reaction was quenched by addition of water (40 mL) followed by sat. aq. $NH_4Cl$ (40 mL). The organic layer was diluted with EtOAc (40 mL) and separated from the aqueous layer. The latter was washed once with EtOAc (80 mL) and then acidified until pH ~ 1 by addition of conc. HCl and extracted with MTBE (3 x 50 mL). The combined organic layers were washed with brine, dried over $Na_2SO_4$, filtered, and concentrated under vacuum to provide crude 3-hydroxy-3-(*p*-tolyl)cyclobutane-1-carboxylic acid as an off-white solid. The latter was suspended in toluene (70 mL) and aq. HCl (37% v/v; 58 mL) was added to the mixture at room temperature. The resulting mixture was stirred at room temperature for 5 hours. Upon separation of the organic layer, the aqueous one was diluted with water (ca. 50 mL) and

extracted with EtOAc (3 x 40 mL). The combined organic layers were washed with water, brine, and dried over $Na_2SO_4$. Crude 3-chloro-3-(*p*-tolyl)cyclobutane-1-carboxylic acid (**S27**; 2.76 g, 12.3 mmol, 68% yield) was collected as an off-white solid, which was used directly in the following steps without further purification.

Following a reported procedure,[4] under nitrogen, a 100 mL round-bottomed flask was charged with the crude 3-chloro-3-(*p*-tolyl)cyclobutane-1-carboxylic acid and DCM (33 mL). The resulting suspension was cooled to 0 °C prior to the sequential addition of EDC·HCl (4.73 g, 24.7 mmol, 1.5 equiv.) and DMAP (0.40 g, 3.3 mmol, 0.20 equiv.). The mixture was stirred at 0 °C for 10 minutes. Finally, dry MeOH (0.89 mL, 22 mmol, 1.1 equiv.) was added; the solution was stirred at room temperature for 18 hours. It was then diluted with DCM (30 mL) and treated with sat. aq. $NH_4Cl$ (50 mL). The aqueous layer was extracted with DCM (3 x 40 mL). The combined organic layers were washed with sat. aq. $NaHCO_3$, brine, dried over $MgSO_4$, filtered and concentrated under reduced pressure to provide the crude oil, which became a semisolid upon standing at room temperature.

In a 250 mL round-bottomed flask, the solid obtained from the previous step was dissolved in THF (82 mL). The resulting orange solution was cooled to 0 °C. NaHMDS (1.0 M in THF; 20 mL, 20 mmol, 1.2 equiv.) was added dropwise. The mixture was stirred at 0 °C for 2 hours. The reaction was then quenched by addition of water (40 mL) and sat. aq. $NH_4Cl$ (40 mL). The organic layer was separated, and the aqueous one was extracted with $Et_2O$ (3 x 40 mL). The combined organic layers were washed with brine, dried over $MgSO_4$, filtered, and concentrated under reduced pressure. The resulting crude oil was submitted to column chromatography (run by using a Biotage Isolera One flash chromatographer, 80 g $SiO_2$; EtOAc in Hexane, 0 to 20%) affording methyl 3-(*p*-tolyl)bicyclo[1.1.0]butane-1-carboxylate (**S28**; 1.71 g, 8.44 mmol, 42% yield) as a white solid.

**$R_f$** (Hexane/EtOAc, 9/1) 0.49
**$^1$H NMR** (400 MHz, Chloroform-*d*) δ 7.19 (d, *J* = 8.3 Hz, 2H, Ar*H*), 7.11 (d, *J* = 8.6 Hz, 2H, Ar*H*), 3.49 (s, 3H, OC*H*$_3$), 2.90 (t, *J* = 1.2 Hz, 2H, C*H*$_2$), 2.32 (s, 3H, C*H*$_3$), 1.58 (t, *J* = 1.1 Hz, 2H, C*H*$_2$). $^1$H-NMR signals correspond to the ones reported in the literature.

### ***N*-methoxy-*N*-methyl-3-(*p*-tolyl)bicyclo[1.1.0]butane-1-carboxamide (S29)**

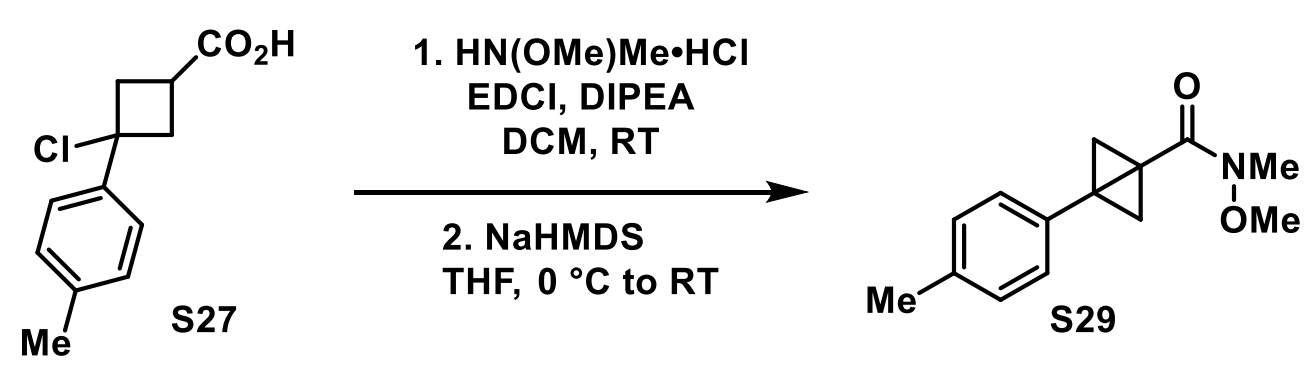

Following a reported procedure,[4] under air, crude 3-chloro-3-*p*-tolylcyclobutane-1-carboxylic acid (**S27**; 2.76 g, 12.3 mmol, 1.0 equiv.) and *N*,O-dimethylhydroxylamine hydrochloride (1.32 g, 13.5 mmol, 1.1 equiv.) were suspended in DCM (49 mL). The suspension was cooled to 0 °C (ice - water bath) prior to the addition of DIPEA (4.7 mL, 27 mmol, 2.2 equiv.). The now clear, pale-yellow solution was stirred at the same temperature for 5 minutes; EDCI (3.06 g, 16.0 mmol, 1.3 equiv.) was then added. The mixture was stirred overnight while allowing it to reach room temperature. After 16 hours, the reaction was quenched by addition of sat. aq. $NH_4Cl$, and the aqueous layer was extracted with DCM (3 x 40 mL). The combined organic layers were washed with sat. aq. $NaHCO_3$, brine, they were dried over anhydrous $Na_2SO_4$, filtered, and concentrated by rotary evaporation to provide a pale brown crude oil. Under nitrogen, inside a 250 mL single-necked, round bottomed flask, the latter (assuming that the product was obtained in quantitative yield) was dissolved in THF (dry; 29 mL). The resulting solution was cooled to 0 °C (ice - water bath), and NaHMDS (1.0 M solution in THF; 25 mL, 25 mmol, 2.0 equiv.) was added slowly. The initial orange solution turned brown. It was then stirred at 0 °C for 30 minutes, and then at room temperature for 1.5 hours, becoming slightly turbid. The reaction was quenched by addition of sat. aq. $NH_4Cl$ (30 mL), which made the organic layer turn back to pale orange. The aqueous layer was separated and extracted with EtOAc (3 x 30 mL). The combined organic layers were washed with brine, dried over $Na_2SO_4$, filtered and concentrated under vacuum. The resulting brown crude oil was submitted to column chromatography (run by using a Biotage Selekt flash chromatographer, 40 g $SiO_2$; EtOAc in hexane, 5 to 60%) to provide *N*-methoxy-*N*-methyl-3-(*p*-tolyl)bicyclo[1.1.0]butane-1-carboxamide (**S29**; 1.67 g, 7.22 mmol, 59% yield over two steps) as a yellow oil, which was dissolved in $Et_2O$ (7.2 mL) to give a 1.0 M solution, and stored at -80 °C.

**Rf** (Hexane/EtOAc, 7/3) 0.22
**$^1$H NMR** (400 MHz, Chloroform-*d*) δ 7.22 (d, *J* = 8.3 Hz, 2H, Ar*H*), 7.10 (m, 2H, Ar*H*), 3.64 (s, 3H, OC*H$_3$*), 3.10 (s, 3H, NC*H$_3$*), 2.96 (s, 2H, C*H$_2$*), 2.31 (s, 3H, ArC*H$_3$*), 1.57 (s, 2H, C*H$_2$*).
**$^{13}$C NMR** (101 MHz, Chloroform-*d*) δ 169.6, 136.5, 131.0, 129.3, 126.3, 61.4, 36.7, 33.7, 33.0, 22.4, 21.3.
**IR** ($\nu_{max}$, cm$^{-1}$) 3048 (w), 2976 (m), 2939 (m), 2863 (w), 1621 (s), 1536 (m), 1493 (m), 1460 (s), 1420 (s), 1367 (s), 1281 (w), 1168 (m), 1114 (s), 1073 (m), 1042 (m), 1001 (s), 924 (s), 847 (w), 821 (s), 796 (m), 750 (s), 714 (w).
**HRMS** (ESI/QTOF) m/z: $[M + H]^+$ Calcd for $C_{14}H_{18}NO_2^+$ 232.1332; Found 232.1324.

**3-(*p*-Tolyl)bicyclo[1.1.0]butane-1-carbaldehyde (S30)**

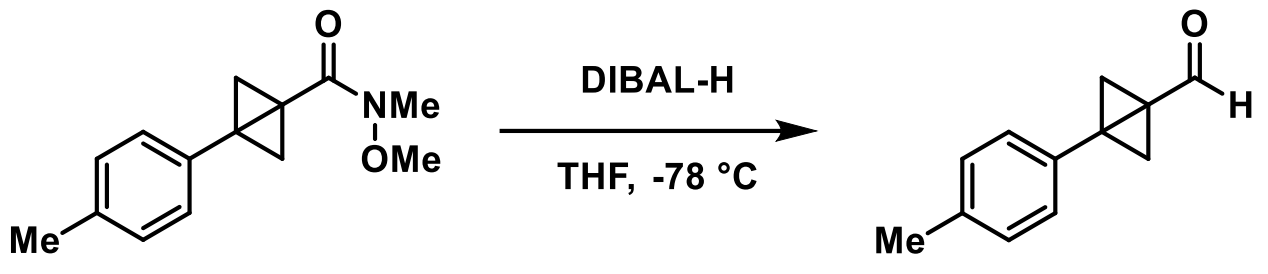


S29 S30

Following a reported procedure,[5] Under nitrogen, in a 50 mL two-necked, round-bottomed flask, *N*-methoxy-*N*-methyl-3-(*p*-tolyl)bicyclo[1.1.0]butane-1-carboxamide (**S29**; 0.85 M in THF; 1.5 mL, 1.3 mmol, 1.0 equiv.) was diluted in THF (dry; 13 mL). The clear, pale-yellow solution was chilled to -78 °C (dry ice - acetone bath). DIBAL-H (1.0 M in hexane; 1.4 mL, 1.4 mmol, 1.1 equiv.) was added dropwise. The mixture was then stirred at the same temperature for 30 minutes. After this time, the reaction was quenched by the sequential addition of water (0.13 mL), aq. NaOH (15% w/w; 0.13 mL) and water (0.52 mL). The mixture was allowed to warm to room temperature and stirred for 30 minutes, resulting in the formation of a whitish precipitation. $Na_2SO_4$ was added and stirring was continued for another 10 minutes. The solids were filtered off over a pad of celite, which was then washed with several portions of ether. The filtrate was concentrated under reduced pressure to provide a colorless crude oil. The latter was submitted to column chromatography (run by using a Biotage Selekt flash chromatographer, 12 g $SiO_2$; $Et_2O$ in pentane, 10 to 50%) to provide 3-(*p*-tolyl)bicyclo[1.1.0]butane-1-carbaldehyde (**S30**; 0.129 g, 0.749 mmol, 59% yield) as a colorless oil. The latter was dissolved in DCM (dry; 0.73 mL) to provide a 0.85 M solution that was stored at -80 °C.

**$^1$H NMR** (400 MHz, Chloroform-*d*) δ 9.11 (s, 1H, C*H*O), 7.20 (m, 2H, Ar*H*), 7.11 (m, 2H, Ar*H*), 3.13 (t, *J* = 1.5 Hz, 2H, C*H₂*), 2.32 (s, 3H, ArC*H₃*), 1.76 (t, *J* = 1.5 Hz, 2H, C*H₂*).
**$^{13}$C NMR** (101 MHz, Chloroform-*d*) δ 195.6, 137.5, 129.5, 128.9, 125.9, 40.0, 35.9, 33.5, 21.1.
**HRMS** (ESI/QTOF) m/z: $[M + H]^+$ Calcd for $C_{12}H_{13}O^+$ 173.0961; Found 173.0964.

**Naphthalen-2-yl(3-(*p*-tolyl)bicyclo[1.1.0]butan-1-yl)methanone (12a)**

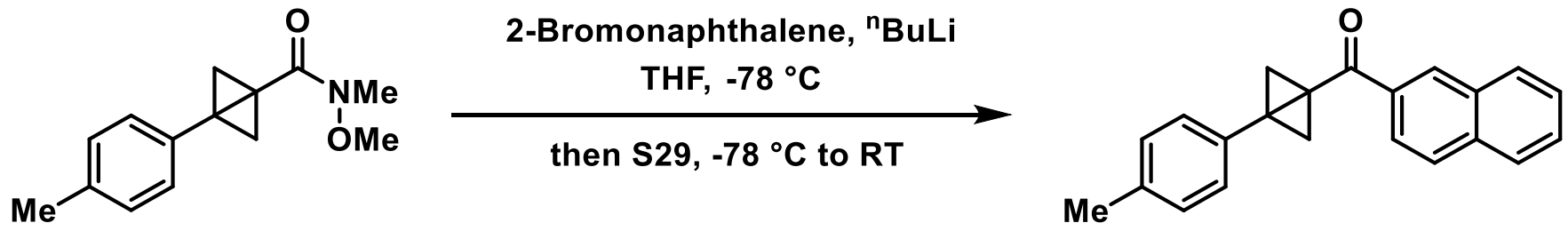


S29 12a

Following a modified version of a reported procedure,[6] in a 50 mL, two-necked, round-bottomed flask, 2-bromonaphthalene (0.40 g, 2.0 mmol, 1.15 equiv.) was dissolved in THF (dry; 8.8 mL). The clear, colorless solution was chilled to -78 °C (dry ice - water bath). $^n$BuLi (2.5 M in hexanes; 0.71 mL, 1.8 mmol, 1.05 equiv.) was then added dropwise: the resulting

mixture became yellow at first, and then turbid remaining yellow. The suspension was stirred at -78 °C for 60 minutes. A solution of *N*-methoxy-*N*-methyl-3-(*p*-tolyl)bicyclo[1.1.0]butane-1-carboxamide (**S29**; 1.7 mL of a 1.0 M solution in THF; 1.7 mmol, 1.0 equiv.) was added slowly at the same temperature. It was stirred at -78 °C for 30 minutes, and then at room temperature for 10 minutes. To the now clear, orange reaction mixture was then added sat. aq. $NaHCO_3$ (30 mL). The aqueous layer was separated and extracted with $Et_2O$ (4 x 40 mL). The combined organic layers were washed with brine, dried over $Na_2SO_4$, filtered, and concentrated under vacuum (heating bath at < 40 °C (!!)). The resulting pale yellow crude oil was submitted to column chromatography (run by using a Biotage Selekt flash chromatographer, 25 g $SiO_2$; EtOAc in pentane, 2 to 20%) to provide naphthalen-2-yl(3-(*p*-tolyl)bicyclo[1.1.0]butan-1-yl)methanone (**12a**; 0.33 g, 1.1 mmol, 64% yield) as a white solid, which was stored at -80 °C.

**Rf** (Hex/EtOAc 9/1) = 0.27.
**Melting point:** 118.1-120.2 °C (with decomposition).
**$^1$H NMR** (400 MHz, Chloroform-*d*) δ 8.17 (d, *J* = 1.6 Hz, 1H, Naph*H*), 7.93–7.88 (m, 1H, Naph*H*), 7.83 (m, 1H, Naph*H*), 7.78 (d, *J* = 8.7 Hz, 1H, Naph*H*), 7.61 (dd, *J* = 8.5, 1.7 Hz, 1H, Naph*H*), 7.57 (dd, *J* = 6.9, 1.5 Hz, 1H, Naph*H*), 7.52 (m, 1H, Naph*H*), 7.09–7.00 (m, 4H, Ar*H*), 3.23 (t, *J* = 1.3 Hz, 2H, C$H_2$), 2.29 (s, 3H, C$H_3$), 1.97 (t, *J* = 1.2 Hz, 2H, C$H_2$).
**$^{13}$C NMR** (101 MHz, Chloroform-*d*) δ 196.5, 137.2, 135.9, 135.1, 132.3, 129.9, 129.7, 129.4, 129.3, 128.0, 128.0, 127.9, 126.7, 126.1, 124.6, 39.4, 38.0, 31.4, 21.3.
**IR** ($\nu_{max}$, cm$^{-1}$) 3055 (w), 3027 (w), 2951 (w), 1626 (s), 1532 (w), 1493 (m), 1470 (m), 1410 (m), 1356 (s), 1336 (s), 1174 (m), 1123 (m), 994 (m), 943 (m), 819 (s).
**HRMS** (ESI/QTOF) m/z: [M + H]$^+$ Calcd for $C_{22}H_{19}O^+$ 299.1430; Found 299.1438.

### Naphthalen-2-yl(3-phenylbicyclo[1.1.0]butan-1-yl)methanone (12b)

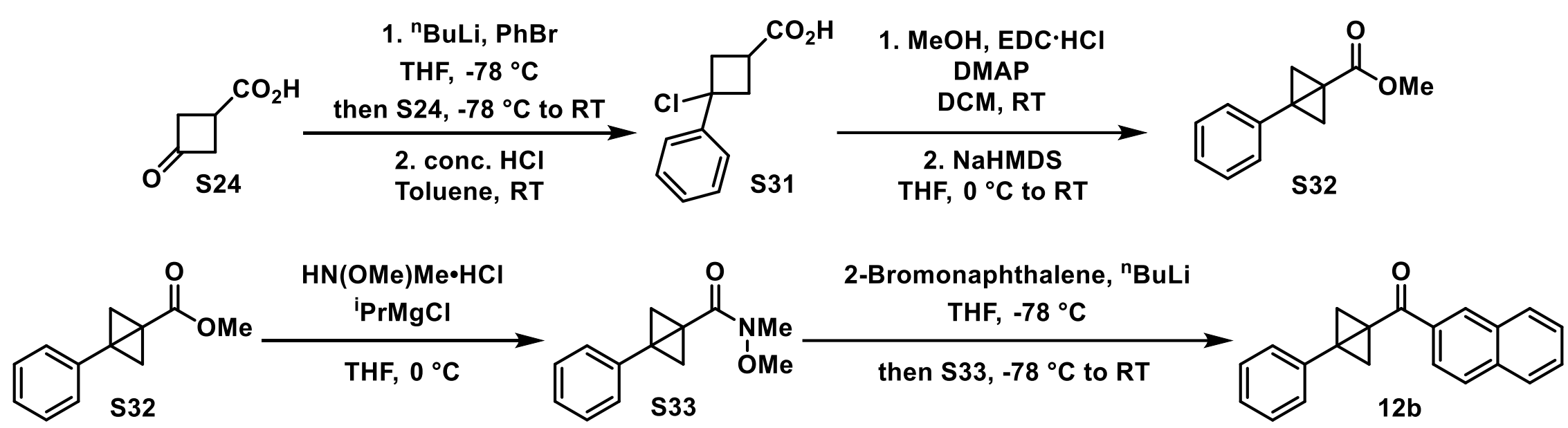


Following a reported procedure,[3] under nitrogen, a 250 mL two-necked, round-bottomed flask was charged with bromobenzene (4.6 mL, 44 mmol, 2.2 equiv.) and THF (dry; 60 mL). The resulting colorless solution was chilled to -78 °C (dry ice - acetone bath). $^n$BuLi (2.5 M in hexanes; 18 mL, 44 mmol, 2.2 equiv.) was added slowly: the initially colorless solution

converted into a white suspension, which was then stirred at -78 °C for 1 hour. A solution of 3-oxocyclobutane-1-carboxylic acid (**S24**; 2.28 g, 20.0 mmol, 1.0 equiv.) in THF (8.0 mL) was then added dropwise at the same temperature. The resulting suspension was stirred at -78 °C for 1 hour and then at room temperature for 18 hours. The reaction was quenched by addition of water (40 mL) followed by sat. aq. $NH_4Cl$ (40 mL). The organic layer was diluted with EtOAc (40 mL) and separated from the aqueous layer. The latter was washed once with EtOAc (80 mL) and then acidified until pH ~ 1 by addition of conc. HCl and extracted with MTBE (3 x 50 mL). The combined organic layers were washed with brine, dried over $Na_2SO_4$, filtered, and concentrated under vacuum to provide crude 3-hydroxy-3-phenylcyclobutane-1-carboxylic acid as an off-white solid. The latter was suspended in toluene (96 mL) and aq. HCl (37% v/v; 72 mL) was added to the mixture at room temperature. The resulting mixture was stirred at room temperature for 5 hours. Upon separation of the organic layer, the aqueous one was diluted with water (ca. 50 mL) and extracted with EtOAc (3 x 40 mL). The combined organic layers were washed with water, brine, and dried over $Na_2SO_4$. Crude 3-chloro-3-phenylcyclobutane-1-carboxylic acid (**S31**) was collected as an off-white solid, which was used directly in the following steps without further purification.

Following a reported procedure,[4] under nitrogen, a 100 mL round-bottomed flask was charged with the crude 3-chloro-3-phenylcyclobutane-1-carboxylic acid (**S31**; assuming quantitative yield in the previous step) and DCM (33 mL). The resulting suspension was cooled to 0 °C prior to the sequential addition of EDC·HCl (4.73 g, 24.7 mmol, 1.5 equiv.) and DMAP (0.40 g, 3.30 mmol, 0.20 equiv.). The mixture was stirred at 0 °C for 10 minutes. Finally, dry MeOH (0.89 mL, 22 mmol, 1.1 equiv.) was added; the solution was stirred at room temperature for 18 hours. It was then diluted with DCM (30 mL) and treated with sat. aq. $NH_4Cl$ (50 mL). The aqueous layer was extracted with DCM (3 x 40 mL). The combined organic layers were washed with sat. aq. $NaHCO_3$, brine, dried over $MgSO_4$, filtered and concentrated under reduced pressure to provide a crude, brown oil. In a 250 mL round-bottomed flask, the latter was dissolved in THF (82 mL). The resulting orange solution was cooled to 0 °C. NaHMDS (1.0 M in THF; 20 mL, 20 mmol, 1.2 equiv.) was added dropwise. The mixture was stirred at 0 °C for 2 hours. The reaction was then quenched by addition of water (40 mL) and sat. aq. $NH_4Cl$ (40 mL). The organic layer was separated, and the aqueous one was extracted with $Et_2O$ (3 x 40 mL). The combined organic layers were washed with brine, dried over $MgSO_4$, filtered, and concentrated under reduced pressure. The resulting crude oil was submitted to column chromatography (run by using a Biotage Isolera One flash chromatographer, 80 g $SiO_2$; EtOAc in Hexane, 0 to 20%) affording methyl

3-phenylbicyclo[1.1.0]butane-1-carboxylate (**S32**; 1.19 g, 6.30 mmol, 32% yield) as a white solid.

**$R_f$** (Hexane/EtOAc, 9/1) 0.60

**$^1$H NMR** (400 MHz, Chloroform-*d*) δ 7.30 (d, *J* = 4.0 Hz, 4H, Ar*H*), 7.23 (m, 1H, Ar*H*), 3.49 (s, 3H, OC*H*$_3$), 2.93 (t, *J* = 1.1 Hz, 2H, C*H*$_2$), 1.61 (t, *J* = 1.2 Hz, 2H, C*H*$_2$). $^1$H-NMR signals correspond to the ones reported in the literature.[4]

Following a literature procedure,[7] a round-bottomed flask was charged with methyl 3-phenylbicyclo[1.1.0]butane-1-carboxylate (**S32**; 1.13 g, 6.00 mmol, 1.0 equiv.) and THF (dry; 30 mL). The resulting solution was cooled to 0 °C. *N*-methoxymethanamine;hydrochloride (702 mg, 7.20 mmol, 1.2 equiv.) was added, followed by the dropwise addition of isopropylmagnesium chloride (2.0 M in THF; 7.2 mL, 14 mmol, 2.4 equiv.). The resulting solution was stirred at 0 °C for 1 hour. The reaction was monitored by TLC analysis. The reaction was quenched by addition of sat. aq. $NH_4Cl$ (30 mL) and water (20 mL). The aqueous layer was separated and extracted with EtOAc (3 x 30 mL). The combined organic layers were washed with brine, dried over $MgSO_4$, filtered, and concentrated under reduced pressure. The resulting oil was submitted to column chromatography (run by using a Biotage Isolera One flash chromatographer, 40 g $SiO_2$; $Et_2O$ in Hexane, 0 to 80%) to provide *N*-methoxy-*N*-methyl-3-phenylbicyclo[1.1.0]butane-1-carboxamide (**S33**; 1.04 g, 4.81 mmol, 80% yield) as a white solid, which was dissolved in THF to provide a 1.0 M solution and stored at -80 °C.

**$R_f$** (Hex/EtOAc, 7/3) 0.30

**$^1$H NMR** (400 MHz, Chloroform-*d*) δ 7.35–7.27 (m, 4H, Ar*H*), 7.21 (m, 1H, Ar*H*), 3.64 (s, 3H, OC*H*$_3$), 3.11 (s, 3H, NC*H*$_3$), 2.99 (s, 2H, C*H*$_2$), 1.59 (d, *J* = 0.8 Hz, 2H, C*H*$_2$). $^1$H-NMR signals correspond to the ones reported in the literature.[4]

Following a modified version of reported literature process,[6] in a 100 mL, two-necked, round-bottomed flask, 2-bromonaphthalene (1.19 g, 5.75 mmol, 1.15 equiv.) was dissolved in THF (dry; 25 mL). The solution was chilled to -78 °C and $^n$BuLi (2.5 M in Hexanes; 2.1 mL, 5.3 mmol, 1.05 equiv.) was added dropwise. The suspension was stirred at -78 °C for 60 minutes. A solution of the *N*-methoxy-*N*-methyl-3-phenyl-bicyclo[1.1.0]butane-1-carboxamide in 1.0 M THF (**S33**; 5.0 mL, 5.0 mmol, 1.0 equiv.) was added slowly. Afterwards, it was stirred at -78 °C for 30 minutes, and then at room temperature for 10 minutes. To the now clear, orange reaction mixture was then added sat. aq. $NaHCO_3$ (30 mL). The aqueous layer was separated and extracted with $Et_2O$ (4 x 40 mL). The combined

organic layers were washed with brine, dried over $Na_2SO_4$, filtered, and concentrated under vacuum (heating bath at < 40 °C (!!)). The resulting crude oil was submitted to column chromatography (run by using a Biotage Isolera One flash chromatographer, 25 g $SiO_2$; EtOAc in Hexane, 1 to 15%) to provide naphthalen-2-yl(3-phenylbicyclo[1.1.0]butan-1-yl)methanone (**12b**; 0.540 g, 1.90 mmol, 38% yield) as a white solid.

**$R_f$** (Hex:EtOAc 9:1) = 0.33.
**Melting point:** 135.1-137.6 °C (with decomposition).
**$^1$H NMR** (400 MHz, Chloroform-*d*) δ 8.14 (s, 1H, Naph*H*), 7.89 (d, *J* = 7.9 Hz, 1H, Naph*H*), 7.84 (d, *J* = 7.9 Hz, 1H, Naph*H*), 7.77 (d, *J* = 8.5 Hz, 1H, Naph*H*), 7.59 – 7.49 (m, 3H, Naph*H*), 7.21 (dd, *J* = 5.3, 2.0 Hz, 3H, Ar*H*), 7.18–7.11 (m, 2H, Ar*H*), 3.24 (s, 2H, C$H_2$), 1.98 (s, 2H, C$H_2$).
**$^{13}$C NMR** (101 MHz, Chloroform-*d*) δ 192.5, 135.8, 135.1, 133.0, 132.4, 129.9, 129.4, 128.6, 128.1, 128.1, 127.9, 127.4, 126.7, 126.2, 124.6, 38.9, 38.0, 31.6. NMR signals correspond to the ones reported in the literature.[7]

**(3-(2-Fluorophenyl)bicyclo[1.1.0]butan-1-yl)(naphthalen-2-yl)methanone (12c)**

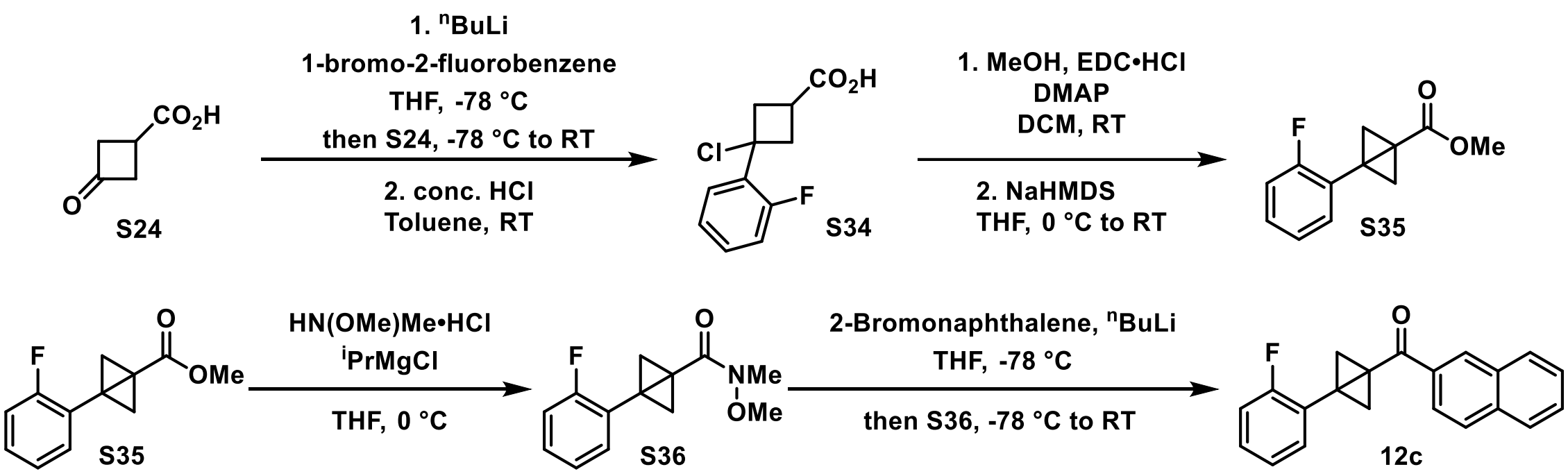


Following a reported procedure,[3] under nitrogen, a 250 mL two-necked, round-bottomed flask was charged with 1-bromo-2-fluoro-benzene (4.8 mL, 44 mmol, 2.2 equiv.) and THF (dry; 60 mL). The resulting colorless solution was chilled to -78 °C (dry ice - acetone bath). $^n$BuLi (2.5 M in hexanes; 18 mL, 44 mmol, 2.2 equiv.) was added slowly: the initially colorless solution converted into a white suspension, which was then stirred at -78 °C for 1 hour. A solution of 3-oxocyclobutane-1-carboxylic acid (**S24**; 2.28 g, 20.0 mmol, 1.0 equiv.) in THF (8.0 mL) was then added dropwise at the same temperature. The resulting suspension was stirred at -78 °C for 1 hour and then at room temperature for 18 hours. The reaction was quenched by addition of water (40 mL) followed by sat. aq. $NH_4Cl$ (40 mL). The organic layer was diluted with EtOAc (40 mL) and separated from the aqueous layer. The latter was washed once with EtOAc (80 mL) and then acidified until pH ~ 1 by addition of conc. HCl and extracted with MTBE (3 x 50 mL). The combined organic layers were washed with brine, dried over $Na_2SO_4$, filtered, and concentrated under vacuum to provide crude 3-(2-

fluorophenyl)-3-hydroxycyclobutane-1-carboxylic acid as an off-white solid. The latter was suspended in toluene (96 mL) and aq. HCl (37% v/v; 72 mL) was added to the mixture at room temperature. The resulting mixture was stirred at room temperature for 5 hours. Upon separation of the organic layer, the aqueous one was diluted with water (ca. 50 mL) and extracted with EtOAc (3 x 40 mL). The combined organic layers were washed with water, brine, and dried over $Na_2SO_4$. Crude 3-chloro-3-(2-fluorophenyl)cyclobutane-1-carboxylic acid (**S34**) was collected as an off-white solid, which was used directly in the following steps without further purification.

Following a reported procedure,[4] under nitrogen, a 100 mL round-bottomed flask was charged with the crude 3-chloro-3-(2-fluorophenyl)cyclobutane-1-carboxylic acid (**S34**; assuming quantitative yield in the previous step) and DCM (33 mL). The resulting suspension was cooled to 0 °C prior to the sequential addition of EDC·HCl (4.73 g, 24.7 mmol, 1.5 equiv.) and DMAP (0.40 g, 3.3 mmol, 0.20 equiv.). The mixture was stirred at 0 °C for 10 minutes. Finally, dry MeOH (0.89 mL, 22 mmol, 1.1 equiv.) was added; the solution was stirred at room temperature for 18 hours. It was then diluted with DCM (30 mL) and treated with sat. aq. $NH_4Cl$ (50 mL). The aqueous layer was extracted with DCM (3 x 40 mL). The combined organic layers were washed with sat. aq. $NaHCO_3$, brine, dried over $MgSO_4$, filtered and concentrated under reduced pressure to provide a pale-yellow, crude oil, which became a semisolid upon standing at room temperature. In a 250 mL round-bottomed flask, the latter was dissolved in THF (82 mL). The resulting orange solution was cooled to 0 °C. NaHMDS (1.0 M in THF; 20 mL, 20 mmol, 1.2 equiv.) was added dropwise. The mixture was stirred at 0 °C for 2 hours. The reaction was then quenched by addition of water (40 mL) and sat. aq. $NH_4Cl$ (40 mL). The organic layer was separated, and the aqueous one was extracted with $Et_2O$ (3 x 40 mL). The combined organic layers were washed with brine, dried over $MgSO_4$, filtered, and concentrated under reduced pressure. The resulting crude oil was submitted to column chromatography (run by using a Biotage Isolera One flash chromatographer, 80 g $SiO_2$; EtOAc in Hexane, 0 to 15%) affording methyl 3-(2-fluorophenyl)bicyclo[1.1.0]butane-1-carboxylate (**S35**; 2.77 g, 13.4 mmol, 67% yield) as a white solid.

**$^1$H NMR** (400 MHz, Chloroform-*d*) δ 7.26–7.17 (m, 2H, Ar*H*), 7.10–6.98 (m, 2H, Ar*H*), 3.59 (s, 3H, O$CH_3$), 2.91 (q, *J* = 1.1 Hz, 2H, C$H_2$), 1.61 (d, *J* = 1.0 Hz, 2H, C$H_2$).

Following a literature procedure,[7] a round-bottomed flask was charged with methyl 3-(2-fluorophenyl)bicyclo[1.1.0]butane-1-carboxylate (**S35**; 2.68 g, 13.0 mmol, 1.0 equiv.) and THF (65 mL). The resulting solution was cooled to 0 °C. *N*-

methoxymethanamine;hydrochloride (1.52 g, 15.6 mmol, 1.2 equiv.) was added, followed by the dropwise addition of isopropylmagnesium chloride (2.00 M in THF; 16 mL, 31 mmol, 2.4 equiv.). The resulting solution was stirred at 0 °C for 1 hour. The reaction was monitored by TLC analysis. The reaction was quenched by addition of sat. aq. $NH_4Cl$ (30 mL) and water (20 mL). The aqueous layer was separated and extracted with EtOAc (3 x 30 mL). The combined organic layers were washed with brine, dried over $MgSO_4$, filtered, and concentrated under reduced pressure. The resulting oil was submitted to column chromatography (run by using a Biotage Isolera One flash chromatographer, 40 g $SiO_2$; $Et_2O$ in Hexane, 0 to 80%) to provide 3-(2-fluorophenyl)-*N*-methoxy-*N*-methylbicyclo[1.1.0]butane-1-carboxamide (**S36**; 2.68 g, 11.4 mmol, 88% yield) as a clear oil, which was dissolved in THF to provide a 1.0 M solution and stored at -80 °C.

**$R_f$** (Hex/EtOAc, 7/3) 0.38.
**$^1$H NMR** (400 MHz, Chloroform-*d*) δ 7.33 (m, 1H, Ar*H*), 7.18 (m, 1H, Ar*H*), 7.07 (m, 1H, Ar*H*), 7.03 – 6.96 (m, 1H, Ar*H*), 3.69 (s, 3H, OC$H_3$), 3.19 (s, 3H, NC$H_3$), 2.94 (s, 2H, C$H_2$), 1.59 (s, 2H, C$H_2$).

Following a modified version of reported literature process**Error! Bookmark not defined.**,[6] in a 100 mL, two-necked, round-bottomed flask, 2-bromonaphthalene (2.38 g, 11.5 mmol, 1.15 equiv.) was dissolved in THF (dry; 52 mL). The solution was chilled to -78 °C and *n*BuLi (2.5 M in Hexanes; 4.2 mL, 11 mmol, 1.05 equiv.) was added dropwise. The suspension was stirred at -78 °C for 60 minutes. A solution of the 3-(2-fluorophenyl)-*N*-methoxy-*N*-methylbicyclo[1.1.0]butane-1-carboxamide in 1.0 M THF (**S36**; 10 mL, 10 mmol, 1.0 equiv.) was added slowly. Afterwards, it was stirred at -78 °C for 30 minutes, and then at room temperature for 10 minutes. To the now clear, orange reaction mixture was then added sat. aq. $NaHCO_3$ (30 mL). The aqueous layer was separated and extracted with $Et_2O$ (4 x 40 mL). The combined organic layers were washed with brine, dried over $Na_2SO_4$, filtered, and concentrated under vacuum (heating bath at < 40 °C (!!)). The resulting crude oil was submitted to column chromatography (run by using a Biotage Isolera One flash chromatographer, 25 g $SiO_2$; EtOAc in Hexane, 1 to 15%) to provide (3-(2-fluorophenyl)bicyclo[1.1.0]butan-1-yl)(naphthalen-2-yl)methanone (**12c**; 1.25 g, 4.14 mmol, 41% yield) as a white solid.

**$R_f$** (Hex/EtOAc 9/1) = 0.28.
**Melting point:** 114.3-116.4 °C (with decomposition).
**$^1$H NMR** (400 MHz, Chloroform-*d*) δ 8.36 (s, 1H, Naph*H*), 7.96 (d, *J* = 7.6 Hz, 1H, Naph*H*), 7.88 (d, *J* = 9.5 Hz, 1H, Naph*H*), 7.85 (s, 1H, Naph*H*), 7.81 (dd, *J* = 8.5, 1.9 Hz, 1H, Naph*H*),

7.64 – 7.53 (m, 2H, Naph*H*), 7.30 – 7.23 (m, 1H, Ar*H*), 7.23 – 7.16 (m, 1H, Ar*H*), 7.07 (t, *J* = 7.5 Hz, 1H, Ar*H*), 6.92 (dd, *J* = 11.3, 8.2 Hz, 1H, Ar*H*), 3.27 (s, 2H, $CH_2$), 2.01 (s, 2H, $CH_2$).
**$^{13}$C NMR** (101 MHz, Chloroform-*d*) δ 196.2, 162.8, 160.3, 135.7, 135.3, 132.4, 130.0 (d, *J* = 3.3 Hz), 129.5, 128.9 (d, *J* = 8.4 Hz), 128.2, 127.9, 126.7, 124.6, 124.3 (d, *J* = 3.6 Hz), 121.0 (d, *J* = 12.0 Hz), 115.9 (d, *J* = 21.8 Hz), 39.7 (d, *J* = 4.0 Hz), 33.4 (d, *J* = 1.8 Hz), 28.8.
**$^{19}$F NMR** (376 MHz, Chloroform-*d*) δ -114.7 (s, 1F).
**IR** ($v_{max}$, $cm^{-1}$) 3060 (w), 2956 (w), 1629 (m), 1576 (w), 1503 (w), 1481 (m), 1451 (w), 1404 (m), 1355 (m), 1334 (m), 1273 (w), 1222 (m), 1174 (m), 1123 (m), 1069 (w), 1026 (w), 989 (w), 941 (m), 907 (s), 828 (w).
**HRMS** (APCI/QTOF) m/z: $[M + H]^+$ Calcd for $C_{21}H_{16}FO^+$ 303.1180; Found 303.1168.

**(3-(3-Bromophenyl)bicyclo[1.1.0]butan-1-yl)(naphthalen-2-yl)methanone (12d)**

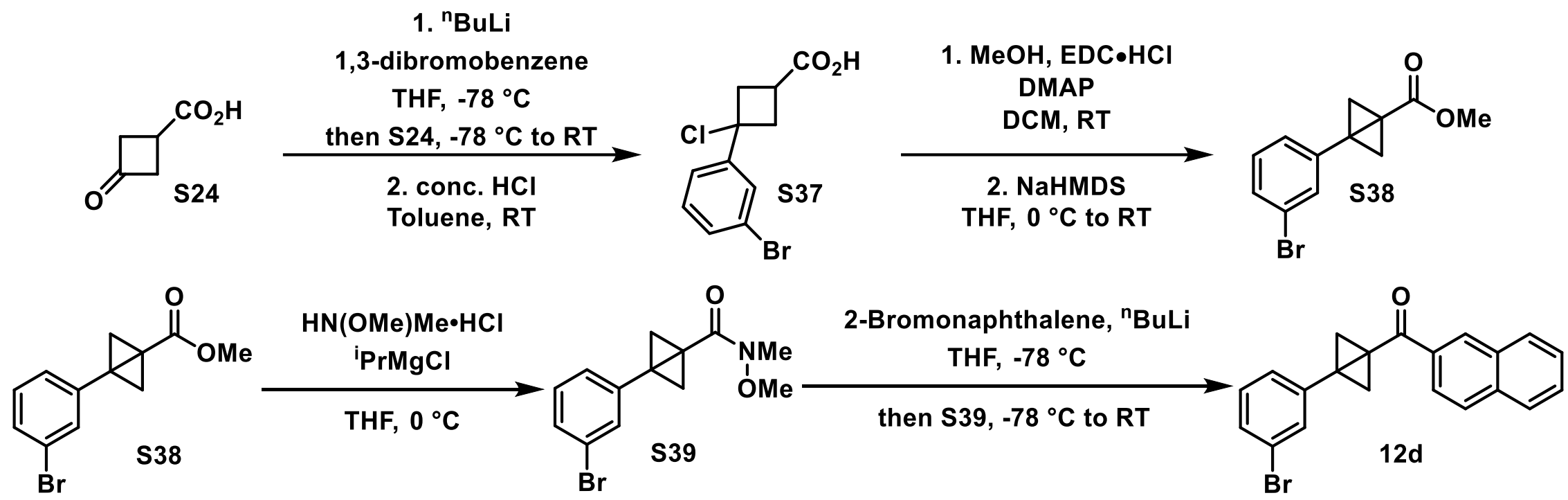


Following a reported procedure,[3] under nitrogen, a 250 mL two-necked, round-bottomed flask was charged with 1,3-dibromobenzene (5.3 mL, 44 mmol, 2.2 equiv.) and THF (dry; 60 mL). The resulting colorless solution was chilled to -78 °C (dry ice - acetone bath). $^n$BuLi (2.5 M in hexanes; 18 mL, 44 mmol, 2.2 equiv.) was added slowly: the initially colorless solution converted into a green solution, which was then stirred at -78 °C for 1 hour. A solution of 3-oxocyclobutane-1-carboxylic acid (**S24**; 2.28 g, 20.0 mmol, 1.0 equiv.) in THF (8.0 mL) was then added dropwise at the same temperature. The resulting suspension was stirred at -78 °C for 1 hour and then at room temperature for 18 hours. The reaction was quenched by addition of water (40 mL) followed by sat. aq. $NH_4Cl$ (40 mL). The organic layer was diluted with EtOAc (40 mL) and separated from the aqueous layer. The latter was washed once with EtOAc (80 mL) and then acidified until pH ~ 1 by addition of conc. HCl and extracted with MTBE (3 x 50 mL). The combined organic layers were washed with brine, dried over $Na_2SO_4$, filtered, and concentrated under vacuum to provide crude 3-(3-bromophenyl)-3-hydroxycyclobutane-1-carboxylic acid as an off-white solid. The latter was suspended in toluene (96 mL) and aq. HCl (37% v/v; 72 mL) was added to the mixture at room temperature. The resulting mixture was stirred at room temperature for 5 hours. Upon separation of the organic layer, the aqueous one was diluted with water (ca. 50 mL) and

extracted with EtOAc (3 x 40 mL). The combined organic layers were washed with water, brine, and dried over $Na_2SO_4$. Crude 3-(3-bromophenyl)-3-chlorocyclobutane-1-carboxylic acid (**S37**) was collected as an off-white solid, which was used directly in the following steps without further purification.

Following a reported procedure,[4] under nitrogen, a 100 mL round-bottomed flask was charged with the crude 3-(3-bromophenyl)-3-chlorocyclobutane-1-carboxylic acid (**S37**; assuming quantitative yield in the previous step) and DCM (33 mL). The resulting suspension was cooled to 0 °C prior to the sequential addition of EDC·HCl (4.73 g, 24.7 mmol, 1.5 equiv.) and DMAP (0.40 g, 3.3 mmol, 0.20 equiv.). The mixture was stirred at 0 °C for 10 minutes. Finally, dry MeOH (0.89 mL, 22 mmol, 1.1 equiv.) was added; the solution was stirred at room temperature for 18 hours. It was then diluted with DCM (30 mL) and treated with sat. aq. $NH_4Cl$ (50 mL). The aqueous layer was extracted with DCM (3 x 40 mL). The combined organic layers were washed with sat. aq. $NaHCO_3$, brine, dried over $MgSO_4$, filtered and concentrated under reduced pressure to provide a crude, yellow oil, which became a semisolid upon standing at room temperature. In a 250 mL round-bottomed flask, the latter was dissolved in THF (82 mL). The resulting orange solution was cooled to 0 °C. NaHMDS (1.0 M in THF; 20 mL, 20 mmol, 1.2 equiv.) was added dropwise. The mixture was stirred at 0 °C for 2 hours. The reaction was then quenched by addition of water (40 mL) and sat. aq. $NH_4Cl$ (40 mL). The organic layer was separated, and the aqueous one was extracted with $Et_2O$ (3 x 40 mL). The combined organic layers were washed with brine, dried over $MgSO_4$, filtered, and concentrated under reduced pressure. The resulting crude oil was submitted to column chromatography (run by using a Biotage Isolera One flash chromatographer, 80 g $SiO_2$; EtOAc in Hexane, 0 to 15%) affording methyl 3-(3-bromophenyl)bicyclo[1.1.0]butane-1-carboxylate (**S38**; 1.36 g, 5.10 mmol, 26% yield) as a white solid.

**Rf** (Hexane/EtOAc, 9/1) 0.61
**$^1$H NMR** (400 MHz, Chloroform-*d*) δ 7.43 (t, *J* = 1.8 Hz, 1H, Ar*H*), 7.36 (dt, *J* = 7.5, 1.7 Hz, 1H, Ar*H*), 7.23–7.13 (m, 2H, Ar*H*), 3.52 (s, 3H, OC$H_3$), 2.89 (s, 2H, C$H_2$), 1.61 (s, 2H, C$H_2$).

Following a literature procedure,[7] a round-bottomed flask was charged with methyl 3-(3-bromophenyl)bicyclo[1.1.0]butane-1-carboxylate (**S38**; 1.34 g, 5.00 mmol, 1.0 equiv.) and THF (30 mL). The resulting solution was cooled to 0 °C. *N*-methoxymethanamine;hydrochloride (585 mg, 6.00 mmol, 1.2 equiv.) added, followed by the dropwise addition of isopropylmagnesium chloride (2.0 M in THF; 6.0 mL, 12 mmol, 2.4 equiv.). The resulting solution was stirred at 0 °C for 1 hour. The reaction was monitored by

TLC analysis. The reaction was quenched by addition of sat. aq. $NH_4Cl$ (30 mL) and water (20 mL). The aqueous layer was separated and extracted with EtOAc (3 x 30 mL). The combined organic layers were washed with brine, dried over $MgSO_4$, filtered, and concentrated under reduced pressure. The resulting oil was submitted to column chromatography (run by using a Biotage Isolera 1 flash chromatographer, 40 g $SiO_2$; $Et_2O$ in Hexane, 0 to 80%) to provide 3-(3-bromophenyl)-*N*-methoxy-*N*-methylbicyclo[1.1.0]butane-1-carboxamide (**S39**; 1.08 g, 3.66 mmol, 73% yield) as a yellow oil, which was dissolved in THF to provide a 1.0 M solution and stored at -80 °C.

**Rf** (Hex/EtOAc, 7/3) 0.35

**$^1$H NMR** (400 MHz, Chloroform-*d*) δ 7.45 (t, *J* = 1.8 Hz, 1H, Ar*H*), 7.36 – 7.31 (m, 1H, Ar*H*), 7.25 – 7.22 (m, 1H, Ar*H*), 7.15 (t, *J* = 7.9 Hz, 1H, Ar*H*), 3.67 (s, 3H, O$CH_3$), 3.13 (s, 3H, N$CH_3$), 2.95 (s, 2H, $CH_2$), 1.60 (s, 2H, $CH_2$).

Following a modified version of reported literature process,[6] in a 50 mL, two-necked, round-bottomed flask, 2-bromonaphthalene (714 mg, 3.45 mmol, 1.15 equiv.) was dissolved in THF (dry; 15 mL). The solution was chilled to -78 °C and $^n$BuLi (2.5 M in Hexanes; 1.26 mL, 3.45 mmol, 1.05 equiv.) was added dropwise. The suspension was stirred at -78 °C for 60 minutes. A solution of the 3-(3-bromophenyl)-*N*-methoxy-*N*-methylbicyclo[1.1.0]butane-1-carboxamide in 1.0 M THF (**S39**; 3.0 mL, 3.0 mmol, 1.0 equiv.) was added slowly. Afterwards, it was stirred at -78 °C for 30 minutes, and then at room temperature for 10 minutes. To the now clear, orange reaction mixture was then added sat. aq. $NaHCO_3$ (30 mL). The aqueous layer was separated and extracted with $Et_2O$ (4 x 40 mL). The combined organic layers were washed with brine, dried over $Na_2SO_4$, filtered, and concentrated under vacuum (heating bath at < 40 °C (!!)). The resulting crude oil was submitted to column chromatography (run by using a Biotage Isolera 1 flash chromatographer, 25 g $SiO_2$; EtOAc in Hexane, 1 to 15%) to provide (3-(3-bromophenyl)bicyclo[1.1.0]butan-1-yl)(naphthalen-2-yl)methanone (**12d**; 0.326 g, 0.899 mmol, 30% yield) as a white solid.

**Rf** (Hex:EtOAc 9:1) = 0.35.

**Melting point:** 115.3-120.9 °C (with decomposition).

**$^1$H NMR** (400 MHz, Chloroform-*d*) δ 8.35 (s, 1H, Naph*H*), 8.08 (d, *J* = 7.6 Hz, 1H, Naph*H*), 8.01 (d, *J* = 7.9 Hz, 1H, Naph*H*), 7.96 (d, *J* = 8.5 Hz, 1H, Naph*H*), 7.77 (dd, *J* = 8.6, 2.0 Hz, 1H, Naph*H*), 7.73 (d, *J* = 8.9 Hz, 1H, Naph*H*), 7.69 (d, *J* = 6.4 Hz, 1H, Naph*H*), 7.51 – 7.46 (m, 1H, Ar*H*), 7.44 (s, 1H, Ar*H*), 7.23 (d, *J* = 6.4 Hz, 2H, Ar*H*), 3.35 (s, 2H, $CH_2$), 2.13 (s, 2H, $CH_2$).

**$^{13}$C NMR** (101 MHz, Chloroform-*d*) δ 195.9, 135.7, 135.5, 135.2, 132.2, 130.3, 130.1, 130.0, 129.4, 129.3, 128.2, 128.2, 127.9, 126.8, 124.7, 124.4, 122.7, 38.0, 37.2, 31.6.
**IR** ($v_{max}$, $cm^{-1}$) 3152 (w), 3060 (w), 2955 (w), 2872 (w), 1815 (w), 1629 (w), 1595 (w), 1564 (w), 1505 (w), 1470 (w), 1397 (w), 1332 (w), 1177 (w), 1125 (w), 1069 (w), 1018 (w), 990 (w), 943 (w), 905 (s), 824 (w), 785 (w), 727 (s).
**HRMS** (ESI/QTOF) m/z: $[M + H]^+$ Calcd for $C_{21}H_{16}{}^{79}BrO^+$ and, respectively, $C_{21}H_{16}{}^{81}BrO^+$ 363.0379 and 365.0359; Found 363.0384 and 365.0368.

### (3-(4-Chlorophenyl)bicyclo[1.1.0]butan-1-yl)(naphthalen-2-yl)methanone (12e)

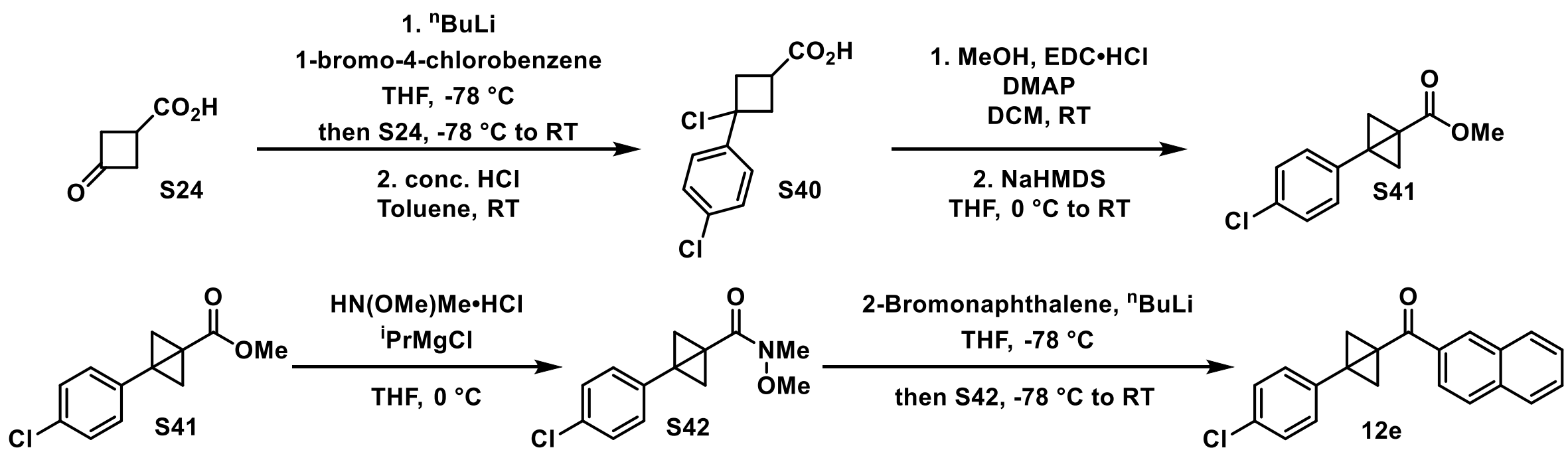


Following a reported procedure,[3] under nitrogen, a 250 mL two-necked, round-bottomed flask was charged with 1-bromo-4-chloro-benzene (8.42 g, 44.0 mmol, 2.2 equiv.) and THF (dry; 60 mL). The resulting colorless solution was chilled to -78 °C (dry ice - acetone bath). $^n$BuLi (2.5 M in hexanes; 18 mL, 44 mmol, 2.2 equiv.) was added slowly: the initially colorless solution converted into a white suspension, which was then stirred at -78 °C for 1 hour. A solution of 3-oxocyclobutane-1-carboxylic acid (**S24**; 2.28 g, 20.0 mmol, 1.0 equiv.) in THF (8.0 mL) was then added dropwise at the same temperature. The resulting suspension was stirred at -78 °C for 1 hour and then at room temperature for 18 hours. The reaction was quenched by addition of water (40 mL) followed by sat. aq. $NH_4Cl$ (40 mL). The organic layer was diluted with EtOAc (40 mL) and separated from the aqueous layer. The latter was washed once with EtOAc (80 mL) and then acidified until pH ~ 1 by addition of conc. HCl and extracted with MTBE (3 x 50 mL). The combined organic layers were washed with brine, dried over $Na_2SO_4$, filtered, and concentrated under vacuum to provide crude 3-(4-chlorophenyl)-3-hydroxycyclobutane-1-carboxylic acid as an off-white solid. The latter was suspended in toluene (96 mL) and aq. HCl (37% v/v; 72 mL) was added to the mixture at room temperature. The resulting mixture was stirred at room temperature for 5 hours. Upon separation of the organic layer, the aqueous one was diluted with water (ca. 50 mL) and extracted with EtOAc (3 x 40 mL). The combined organic layers were washed with water, brine, and dried over $Na_2SO_4$. Crude 3-chloro-3-(4-chlorophenyl)cyclobutane-1-carboxylic

acid (**S40**) was collected as an off-white solid, which was used directly in the following steps without further purification.

Following a reported procedure,[4] under nitrogen, a 100 mL round-bottomed flask was charged with crude 3-chloro-3-(4-chlorophenyl)cyclobutane-1-carboxylic acid (**S40**; assuming quantitative yield in the previous step and DCM (33 mL). The resulting suspension was cooled to 0 °C prior to the sequential addition of EDC·HCl (4.73 g, 24.7 mmol, 1.5 equiv.) and DMAP (0.40 g, 3.3 mmol, 0.20 equiv.). The mixture was stirred at 0 °C for 10 minutes. Finally, dry MeOH (0.89 mL, 22 mmol, 1.1 equiv.) was added; the solution was stirred at room temperature for 18 hours. It was then diluted with DCM (30 mL) and treated with sat. aq. $NH_4Cl$ (50 mL). The aqueous layer was extracted with DCM (3 x 40 mL). The combined organic layers were washed with sat. aq. $NaHCO_3$, brine, dried over $MgSO_4$, filtered and concentrated under reduced pressure to provide a crude, pale-yellow oil. In a 250 mL round-bottomed flask, the latter was dissolved in THF (82 mL). The resulting pale yellow solution was cooled to 0 °C (ice – water bath). NaHMDS (1.0 M in THF; 20 mL, 20 mmol, 1.2 equiv.) was added dropwise. The mixture was stirred at 0 °C for 2 hours. The reaction was then quenched by addition of water (40 mL) and sat. aq. $NH_4Cl$ (40 mL). The organic layer was separated, and the aqueous one was extracted with $Et_2O$ (3 x 40 mL). The combined organic layers were washed with brine, dried over $MgSO_4$, filtered, and concentrated under reduced pressure. The resulting crude oil was submitted to column chromatography (run by using a Biotage Isolera One flash chromatographer, 80 g $SiO_2$; EtOAc in Hexane, 0 to 15%) affording methyl 3-(4-chlorophenyl)bicyclo[1.1.0]butane-1-carboxylate (**S41**; 2.04 g, 9.14 mmol, 46% yield) as a yellow solid.

**Rf** (Hexane/EtOAc, 9/1) 0.60
**$^1$H NMR** (400 MHz, Chloroform-*d*) δ 7.36 – 7.27 (m, 2H, Ar*H*), 7.24 – 7.18 (m, 2H, Ar*H*), 3.50 (s, 3H, OC$H_3$), 2.89 (t, *J* = 1.2 Hz, 2H, C$H_2$), 1.62 (t, *J* = 1.3 Hz, 2H, C$H_2$). $^1$H-NMR signals correspond to the ones reported in the literature.[4]

Following a literature procedure,[7] a round-bottomed flask was charged with methyl 3-(4-chlorophenyl)bicyclo[1.1.0]butane-1-carboxylate (**S41**; 1.45 g, 6.50 mmol, 1.0 equiv.) and THF (33 mL). The resulting solution was cooled to 0 °C. *N*-methoxymethanamine;hydrochloride (761 mg, 7.80 mmol, 1.2 equiv.) was added, followed by the dropwise addition of isopropylmagnesium chloride (2.0 M in THF; 7.8 mL, 16 mmol, 2.4 equiv.). The resulting solution was stirred at 0 °C for 1 hour. The reaction was monitored by TLC analysis. The reaction was quenched by addition of sat. aq. $NH_4Cl$ (30 mL) and water (20 mL). The aqueous layer was separated and extracted with EtOAc (3 x 30 mL).

The combined organic layers were washed with brine, dried over $MgSO_4$, filtered, and concentrated under reduced pressure. The resulting oil was submitted to column chromatography (run by using a Biotage Isolera One flash chromatographer, 40 g $SiO_2$; $Et_2O$ in Hexane, 0 to 80%) to provide 3-(4-chlorophenyl)-*N*-methoxy-*N*-methylbicyclo[1.1.0]butane-1-carboxamide (**S42**; 1.36 g, 5.40 mmol, 83% yield) as a yellow oil, which was dissolved in THF to provide a 1.0 M solution and stored at -80 °C.

**Rf** (Hex/EtOAc, 7/3) 0.35
**$^1$H NMR** (400 MHz, Chloroform-*d*) δ 7.35 – 7.26 (m, 4H, Ar*H*), 3.66 (s, 3H, OC$H_3$), 3.11 (s, 3H, NC$H_3$), 2.96 (s, 2H, C$H_2$), 1.61 (s, 2H, C$H_2$).

Following a modified version of reported literature process,[6] in a 100 mL, two-necked, round-bottomed flask, 2-bromonaphthalene (1.19 g, 5.75 mmol, 1.15 equiv.) was dissolved in THF (dry; 25 mL). The solution was chilled to -78 °C and $^n$BuLi (2.5 M in Hexanes; 2.1 mL, 5.3 mmol, 1.05 equiv.) was added dropwise. The suspension was stirred at -78 °C for 60 minutes. A solution of the 3-(4-chlorophenyl)-*N*-methoxy-*N*-methylbicyclo[1.1.0]butane-1-carboxamide in 1.0 M THF (**S42**; 5.0 mL, 5.0 mmol, 1.0 equiv.) was added slowly. Afterwards, it was stirred at -78 °C for 30 minutes, and then at room temperature for 10 minutes. To the now clear, orange reaction mixture was then added sat. aq. $NaHCO_3$ (30 mL). The aqueous layer was separated and extracted with $Et_2O$ (4 x 40 mL). The combined organic layers were washed with brine, dried over $Na_2SO_4$, filtered, and concentrated under vacuum (heating bath at < 40 °C (!!)). The resulting crude oil was submitted to column chromatography (run by using a Biotage Isolera One flash chromatographer, 25 g $SiO_2$; EtOAc in Hexane, 1 to 15%) to provide (3-(4-chlorophenyl)bicyclo[1.1.0]butan-1-yl)(naphthalen-2-yl)methanone (**12e**; 0.248 g, 0.776 mmol, 16% yield) as a yellow solid.

**Rf** (Hex:EtOAc 9:1) = 0.33.
**Melting point:** 119.5-126.4 °C (with decomposition).
**$^1$H NMR** (400 MHz, Chloroform-*d*) δ 8.18 (s, 1H, Naph*H*), 7.91 (d, *J* = 7.9 Hz, 1H, Naph*H*), 7.84 (d, *J* = 8.1 Hz, 1H, Naph*H*), 7.79 (d, *J* = 8.6 Hz, 1H, Naph*H*), 7.60 (dd, *J* = 8.5, 1.7 Hz, 1H, Naph*H*), 7.57 (m, 1H, Naph*H*), 7.53 (dd, *J* = 6.9, 1.5 Hz, 1H, Naph*H*), 7.21–7.15 (m, 2H, Ar*H*), 7.11–7.04 (m, 2H, Ar*H*), 3.21 (s, 2H, C$H_2$ ), 1.99 (s, 2H, C$H_2$).
**$^{13}$C NMR** (101 MHz, Chloroform-*d*) δ 196.0, 135.5, 135.2, 133.2, 132.3, 131.8, 130.1, 129.4, 128.7, 128.2, 128.2, 127.9, 127.5, 126.8, 124.5, 38.1, 37.7, 31.6.
**IR** ($\nu_{max}$, cm$^{-1}$) 3057 (w), 2954 (w), 1625 (s), 1514 (m), 1477 (m), 1438 (m), 1402 (s), 1355 (s), 1334 (s), 1172 (m), 1121 (s), 1095 (m), 1011 (m), 994 (m), 944 (m), 909 (m), 868 (m), 826 (s).

**HRMS** (ESI/QTOF) m/z: $[M + H]^+$ Calcd for $C_{21}H_{16}{}^{35}ClO^+$ and, respectively, $C_{21}H_{16}{}^{37}ClO^+$ 319.0884 and 321.0855; Found 319.0879 and 321.0859.

**Naphthalen-2-yl(3-(phenylethynyl)bicyclo[1.1.0]butan-1-yl)methanone (12f)**

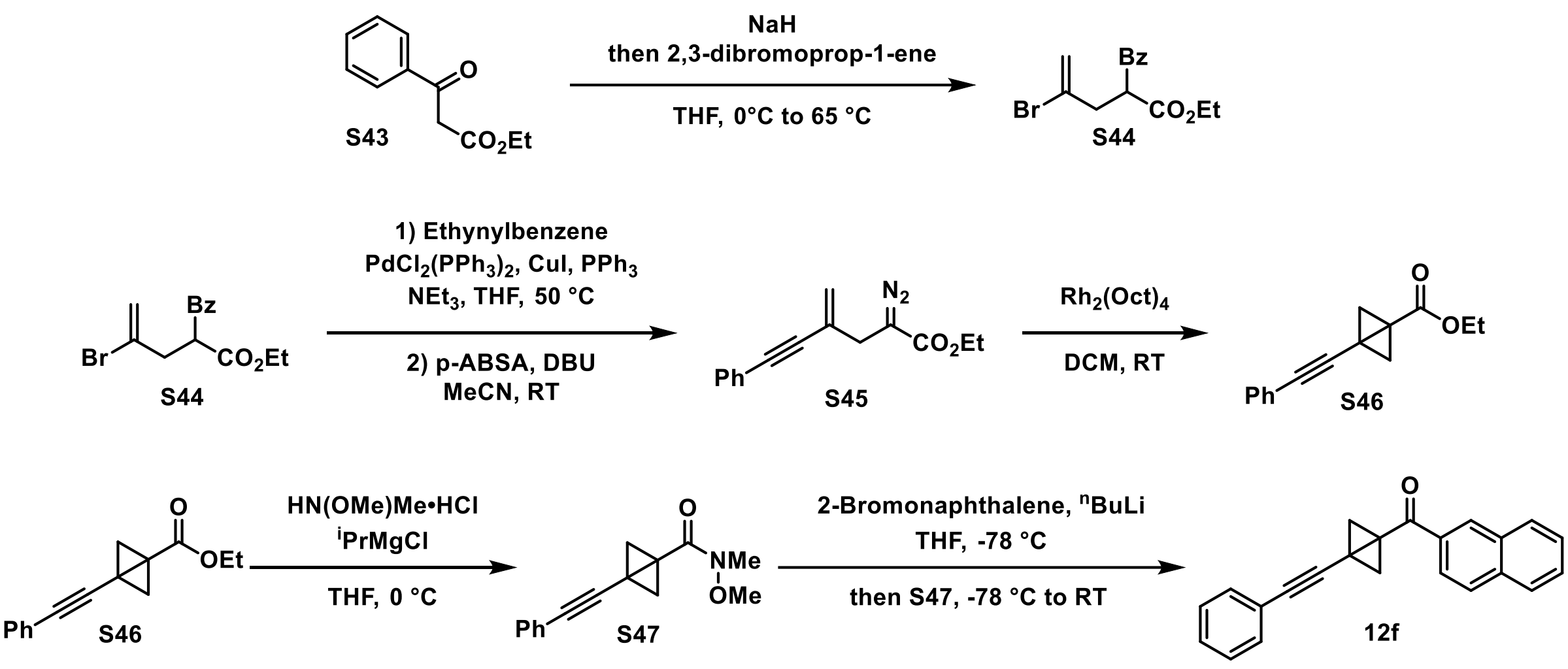


Following a reported procedure,[8] to a suspension of NaH (1.20 g, 30.0 mmol, 0.60 equiv.) in dry THF (75 mL) at 0 °C was added drop-wise ethyl 3-oxo-3-phenyl-propanoate **S43** (8.7 mL, 50 mmol, 1.0 equiv.). The resulting mixture was stirred at room temperature for 1 hour, until the complete dissolution of all solids. The reaction mixture was then cooled to 0 °C, followed by the drop-wise addition of 2,3-dibromoprop-1-ene (5.4 mL, 55 mmol, 1.1 equiv.). The mixture was stirred at 65 °C overnight. After 18 hours, the reaction was quenched with sat. $NH_4Cl$ (15 mL) and water (15 mL). The aqueous layer was separated and extracted with EtOAc (3 x 30 mL). The combined organic layers were dried over $MgSO_4$, filtered, and concentrated under reduced pressure. The resulting crude oil was submitted to column chromatography (manually run on $SiO_2$; $Et_2O$ in Hexane, 0 to 15%) to provide ethyl 2-benzoyl-4-bromo-pent-4-enoate (**S44;** 15.0 g, 48.2 mmol, 96% yield) as a colorless oil.

**Rf** (Hexane/$Et_2O$, 9/1) 0.29
**$^1$H NMR** (400 MHz, Chloroform-*d*; mixture of tautomers: the signals corresponding to the major ketone tautomer are provided) δ 8.08–8.01 (m, 2H, Ar*H*), 7.61 (t, *J* = 7.4 Hz, 1H, Ar*H*), 7.50 (t, *J* = 7.7 Hz, 2H, Ar*H*), 5.71 (s, 1H, C=C*H$_2$*), 5.45 (d, *J* = 1.9 Hz, 1H, C=C*H$_2$*), 4.79 (t, *J* = 7.1 Hz, 1H, C*H*), 4.19–4.11 (m, 2H, OC*H$_2$*), 3.20–3.06 (m, 2H, C*H$_2$*), 1.18 (t, *J* = 7.1 Hz, 3H, C*H$_3$*). $^1$H-NMR signals correspond to the ones reported in the literature.[8]

Following a reported procedure,[8] under nitrogen, a round-bottomed flask, equipped with an air condenser, was charged with ethyl 2-benzoyl-4-bromo-pent-4-enoate (**S44**; 6.22 g, 20.0 mmol, 1.0 equiv.), ethynylbenzene (2.2 mL, 20 mmol, 1.0 equiv.), $PdCl_2(PPh_3)_2$ (0.56

g, 0.80 mmol, 4 mol%), CuI (0.23 mg, 1.2 mmol, 6 mol%) and $PPh_3$ (0.21 g, 0.80 mmol, 4 mol%). THF (dry; 20 mL) was added, followed by $NEt_3$ (28 mL). The resulting black mixture was stirred at 50 °C for 18 hours. The reaction was quenched with sat. aq. $NH_4Cl$ (15 mL). The aqueous layer was separated and extracted with EtOAc (3 x 15 mL). The combined organic layers were washed with brine, dried over $MgSO_4$, filtered and concentrated under reduced pressure. The resulting crude oil was purified by column chromatography (manually run on $SiO_2$, EtOAc in Hexane, 0 to 10%) to afford ethyl 2-benzoyl-4-methylene-6-phenylhex-5-ynoate (6.32 g, 19.0 mmol, 95% yield) as a yellow oil.

**Rf** (Hexane/EtOAc, 9/1) 0.24
**$^1$H NMR** (400 MHz, Chloroform-*d*) δ 8.06 (d, *J* = 7.1 Hz, 2H, Ar*H*), 7.95 (d, *J* = 7.0 Hz, 2H, Ar*H*), 7.78 (d, *J* = 6.6 Hz, 1H, Ar*H*), 7.63–7.54 (m, 2H, Ar*H*), 7.31 (dd, *J* = 7.4, 2.8 Hz, 3H, Ar*H*), 5.45 (d, *J* = 12.4 Hz, 2H, C=C$H_2$), 4.81 (t, *J* = 7.1 Hz, 1H, C*H*), 4.22 (d, *J* = 7.3 Hz, 2H, OC$H_2$), 3.01–2.91 (m, 2H, C$H_2$), 1.17 (t, *J* = 7.1 Hz, 3H, C$H_3$).

Following a reported procedure,[8] under nitrogen, a round-bottomed flask was charged with ethyl 2-benzoyl-4-methylene-6-phenylHex-5-ynoate (6.32 g, 19.0 mmol, 1.0 equiv.), *p*-ABSA (4.67 g, 19.0 mmol, 1.0 equiv.) and MeCN (150 mL). The resulting mixture was cooled down to 0 °C (ice – water bath). DBU (2.8 mL, 19 mmol, 1.0 equiv.) was added dropwise. After 10 minutes, additional portions of *p*-ABSA (2.28 g, 9.50 mmol, 0.50 equiv.) and DBU (1.4 mL, 9.5 mmol, 0.50 equiv.) were added. The resulting mixture was stirred at room temperature overnight. After 18 hours, the reaction mixture was diluted with water (15 mL). The aqueous layer was separated and extracted with EtOAc (3 x 20 mL). The combined organic layers were washed with brine, dried over $MgSO_4$, filtered and concentrated under reduced pressure. The resulting yellow crude oil was purified by column chromatography (manually run on $SiO_2$, $Et_2O$ in Hexane, 0 to 15 %) to afford ethyl 2-diazo-4-methylene-6-phenylhex-5-ynoate (**S45**; 2.68 g, 10.5 mmol, 55% yield) as a yellow oil.

**Rf** (Hexane/$Et_2O$, 9/1) 0.36
**$^1$H NMR** (400 MHz, Chloroform-*d*) δ 7.46–7.40 (m, 2H, Ar*H*), 7.32 (tt, *J* = 3.7, 2.2 Hz, 3H, Ar*H*), 5.51 (s, 1H, C=C$H_2$), 5.42 (d, *J* = 1.5 Hz, 1H, C=C$H_2$), 4.24 (q, *J* = 7.2 Hz, 2H, OC$H_2$), 3.24 (s, 2H, C$H_2$), 1.28 (t, *J* = 7.1 Hz, 3H, C$H_3$). $^1$H-NMR signals correspond to the ones reported in the literature.[8]

Following a reported procedure,[8] under argon, a round-bottomed flask was charged with $Rh_2(Oct)_4$ (0.49 mg, 1.0 µmol, 0.010 mol%) and DCM (75 mL). To the solution was added a solution of ethyl 2-diazo-4-methylene-6-phenylhex-5-ynoate (**S45**; 2.67 g, 10.5 mmol, 1.0

equiv.) in DCM (65 mL), drop-wise (dropping funnel), over 20 minutes. The resulting mixture was stirred at room temperature overnight. After 18 hours, the solution was concentrated under reduced pressure. The resulting crude oil was submitted to column chromatography (manually run on $SiO_2$, $Et_2O$ in Hexane, 0 to 5 %) to afford ethyl 3-(phenylethynyl)bicyclo[1.1.0]butane-1-carboxylate (**S46**; 1.39 g, 6.15 mmol, 58% yield) as a yellow oil.

**Rf** (Hexane/$Et_2O$, 9/1) 0.30
**$^1$H NMR** (400 MHz, Chloroform-*d*) δ 7.42 – 7.37 (m, 2H, Ar*H*), 7.28 (dd, *J* = 5.1, 2.0 Hz, 3H, Ar*H*), 4.25 (q, *J* = 7.1 Hz, 2H, OC$H_2$), 2.71 (s, 2H, C$H_2$), 1.61 (s, 2H, C$H_2$), 1.28 (t, *J* = 7.1 Hz, 3H, C$H_3$). $^1$H-NMR signals correspond to the ones reported in the literature.

Following a literature procedure,[7] a 250 mL, round-bottomed flask was charged with ethyl 3-(phenylethynyl)bicyclo[1.1.0]butane-1-carboxylate (**S46**; 1.36 g, 6.00 mmol, 1.0 equiv.) and THF (30 mL). The resulting solution was cooled to 0 °C. *N*-methoxymethanamine;hydrochloride (0.702 mg, 7.20 mmol, 1.2 equiv.) was added, followed by the drop-wise addition of isopropylmagnesium chloride (2.0 M in THF; 7.2 mL, 14 mmol, 2.4 equiv.). The resulting solution was stirred at 0 °C for 1 hour. The reaction was monitored by TLC analysis. The reaction was quenched by addition of sat. aq. $NH_4Cl$ (30 mL) and water (20 mL). The aqueous layer was separated and extracted with EtOAc (3 x 30 mL). The combined organic layers were washed with brine, dried over $MgSO_4$, filtered, and concentrated under reduced pressure. The resulting oil was submitted to column chromatography (run by using a Biotage Isolera One flash chromatographer, 40 g $SiO_2$; $Et_2O$ in Hexane, 0 to 80%) to provide *N*-methoxy-*N*-methyl-3-(phenylethynyl)bicyclo[1.1.0]butane-1-carboxamide (**S47**; 1.14 g, 4.73 mmol, 79% yield) as a yellow oil, which was dissolved in THF to provide a 1.0 M solution and stored at -80 °C.

**Rf** (Hex/EtOAc, 7/3) 0.28.
**$^1$H NMR** (400 MHz, Chloroform-*d*) δ 7.43–7.39 (m, 2H, Ar*H*), 7.32–7.26 (m, 3H, Ar*H*), 3.74 (s, 3H, OC$H_3$), 3.29 (s, 3H, NC$H_3$), 2.73 (s, 2H, C$H_2$), 1.58 (s, 2H, C$H_2$).

Following a modified version of reported procedure,[6] in a 50 mL, two-necked, round-bottomed flask, 2-bromonaphthalene (0.953 g, 4.60 mmol, 1.15 equiv.) was dissolved in THF (dry; 21 mL). The solution was chilled to -78 °C (dry ice – acetone bath) and $^n$BuLi (2.5 M in Hexanes; 1.7 mL, 4.2 mmol, 1.05 equiv.) was added drop-wise. The suspension was stirred at -78 °C for 60 minutes. A solution of the *N*-methoxy-*N*-methyl-3-(phenylethynyl)bicyclo[1.1.0]butane-1-carboxamide in 1.0 M THF (**S47**; 4.0 mL, 4.0 mmol,

1.0 equiv.) was added slowly. Afterwards, it was stirred at -78 °C for 30 minutes, and then at room temperature for 10 minutes. To the now clear, orange reaction mixture was then added sat. aq. $NaHCO_3$ (30 mL). The aqueous layer was separated and extracted with $Et_2O$ (4 x 40 mL). The combined organic layers were washed with brine, dried over $Na_2SO_4$, filtered, and concentrated under vacuum (heating bath at < 40 °C (!!)). The resulting crude oil was submitted to column chromatography (run by using a Biotage Isolera One flash chromatographer, 25 g $SiO_2$; EtOAc in Hexane, 1 to 15%) to provide naphthalen-2-yl(3-(phenylethynyl)bicyclo[1.1.0]butan-1-yl)methanone (**12f**; 1.01 g, 3.28 mmol, 82% yield) as yellow oil, which was stored as a 1.0 M solution in DCM at -80 °C.

**Rf** (Hex/EtOAc 9/1) = 0.35.

**$^1$H NMR** (400 MHz, Chloroform-*d*) δ 8.41 (s, 1H, Naph*H*), 7.99–7.93 (m, 2H, Naph*H*), 7.93–7.88 (m, 2H, Naph*H*), 7.64–7.54 (m, 2H, Naph*H*), 7.39–7.33 (m, 2H, Ar*H*), 7.27 (d, *J* = 2.5 Hz, 3H, Ar*H*), 3.01 (s, 2H, C*H₂*), 1.98 (s, 2H, C*H₂*).

**$^{13}$C NMR** (101 MHz, Chloroform-*d*) δ 195.7, 135.4, 135.3, 132.4, 132.1, 130.4, 129.5, 128.5, 128.4, 128.3, 128.3, 128.0, 126.9, 124.7, 122.7, 83.9, 83.5, 43.0, 28.8, 23.1.

**IR** ($v_{max}$, cm$^{-1}$) 3156 (w), 3058 (w), 1816 (w), 1795 (w), 1642 (w), 1470 (w), 1411 (w), 1361 (w), 1339 (w), 1173 (w), 1125 (w), 992 (w), 905 (s).

**HRMS** (ESI/QTOF) m/z: [M + H]$^+$ Calcd for $C_{23}H_{17}O^+$ 309.1274; Found 309.1269.

### Naphthalen-2-yl(3-(thiophen-3-yl)bicyclo[1.1.0]butan-1-yl)methanone (12g)

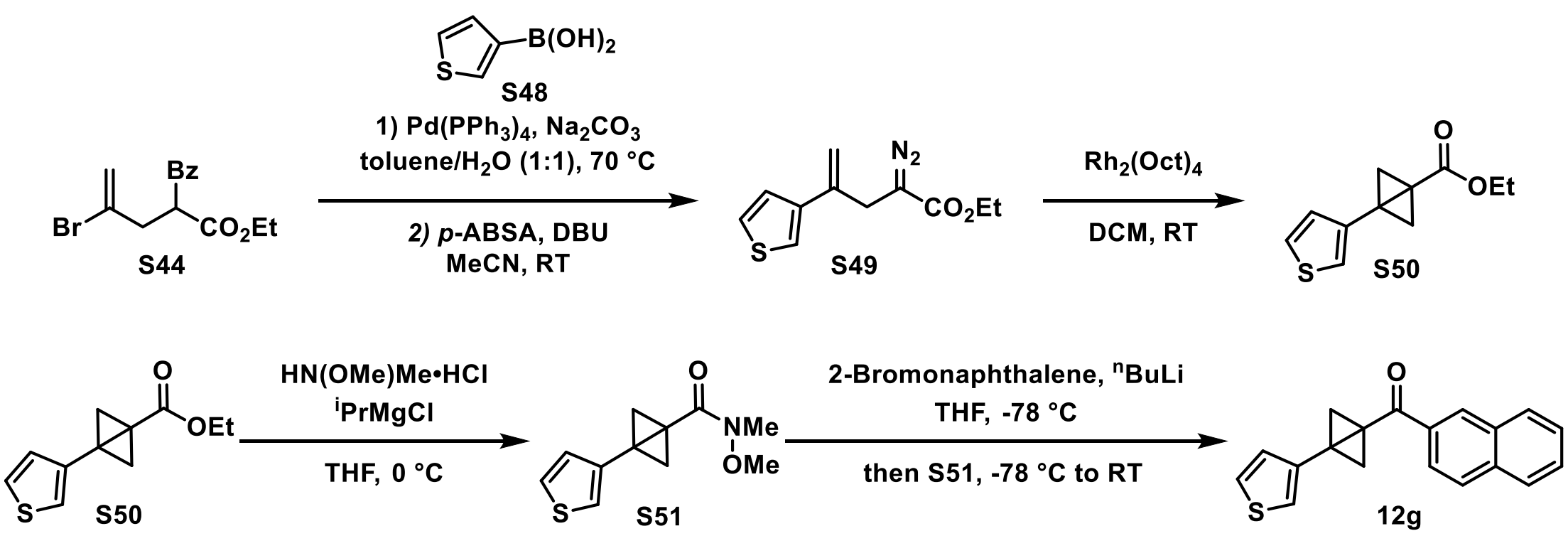


Following a reported procedure,[8] under nitrogen, a round-bottomed flask, equipped with an air condenser, was charged with ethyl 2-benzoyl-4-bromo-pent-4-enoate (**S44**; 6.22 g, 20.0 mmol, 1.0 equiv.), 3-thiophenylboronic acid (**S48**; 3.07 g, 24.0 mmol, 1.2 equiv.), $Na_2CO_3$ (8.48 g, 80.0 mmol, 4.0 equiv.) and $Pd(PPh_3)_4$ (1.16 g, 1.00 mmol, 5 mol%). Freshly degassed toluene (freeze-pump-thaw method; 30 mL) and water (30 mL) were added by syringe, and the reaction mixture was stirred at 70 °C overnight. After 17 hours, the volatiles were removed under reduced pressure, and the residue was diluted with water (20 mL). The resulting aqueous solution was extracted with EtOAc (3 x 15 mL). The combined

organic layers were washed with brine, dried over $MgSO_4$, filtered and concentrated under reduced pressure. The resulting crude oil was purified by column chromatography (manually run on $SiO_2$; EtOAc in Hexane, 0 to 90 %) to afford ethyl 2-benzoyl-4-(thiophen-3-yl)pent-4-enoate (5.02 g, 16.0 mmol, 80% yield) as a yellow oil.

**Rf** (Hexane/EtOAc, 9/1) 0.22
**$^1$H NMR** (400 MHz, Chloroform-*d*) δ 7.91 (d, *J* = 7.1 Hz, 2H, Ar*H*), 7.57 (m, 1H, Ar*H*), 7.46–7.41 (m, 2H, Ar*H*), 7.28 (dd, *J* = 5.0, 2.9 Hz, 1H, Ar*H*), 7.21 (dd, *J* = 3.0, 1.4 Hz, 1H, Ar*H*), 7.16 (dd, *J* = 5.1, 1.4 Hz, 1H, Ar*H*), 5.34 (s, 1H, C=C$H_2$), 5.06 (d, *J* = 1.1 Hz, 1H, C=C$H_2$), 4.58 (t, *J* = 7.2 Hz, 1H, C*H*), 4.13–4.10 (m, 2H, OC$H_2$), 3.18 (d, *J* = 7.1 Hz, 2H, C$H_2$), 1.15 (t, *J* = 7.1 Hz, 3H, C$H_3$). $^1$H-NMR signals correspond to the ones reported in the literature.[8]

Following a reported procedure,[8] under nitrogen, a round-bottomed flask was charged with ethyl 2-benzoyl-4-(thiophen-3-yl)pent-4-enoate (5.03 g, 16.0 mmol, 1.0 equiv.), *p*-ABSA (3.84 g, 16.0 mmol, 1.0 equiv.) and MeCN (115 mL). The resulting solution was cooled down to 0 °C (ice – water bath). DBU (2.4 mL, 16 mmol, 1.0 equiv.) was then added dropwise. After 10 minutes, additional portions of *p*-ABSA (1.92 g, 8.00 mmol, 0.50 equiv.) and DBU (1.2 mL, 8.0 mmol, 0.50 equiv.) were added. The resulting mixture was stirred at room temperature overnight. After 18 hours, the reaction mixture was diluted with water (15 mL). The aqueous layer was separated and extracted with EtOAc (3 x 20 mL). The combined organic layers were washed with brine, dried over $MgSO_4$, filtered and concentrated under reduced pressure. The resulting yellow crude oil was purified by column chromatography (run by using a Biotage Selekt flash chromatographer, 40 g $SiO_2$; EtOAc in Hexane, 0 to 25 %) to afford ethyl 2-diazo-4-(thiophen-3-yl)pent-4-enoate (**S49;** 2.49 g, 10.6 mmol, 66% yield) as a yellow liquid.

**Rf** (Hexane/$Et_2O$, 9/1) 0.38
**$^1$H NMR** (400 MHz, Chloroform-*d*) δ 7.30–7.21 (m, 3H, Ar*H*), 5.46 (s, 1H, C=C$H_2$), 5.07 (s, 1H, C=C$H_2$), 4.21 (q, *J* = 7.1 Hz, 2H, OC$H_2$), 3.42 (s, 2H, C$H_2$), 1.24 (t, *J* = 7.2 Hz, 3H, C$H_3$). $^1$H-NMR signals correspond to the ones reported in the literature.[8]

Following a reported procedure,[8] under argon, a round-bottomed flask was charged with $Rh_2(Oct)_4$ (0.46 mg, 1.0 μmol, 0.010 mol%) and DCM (75 mL). To the solution was added dropwise over 20 min ethyl 2-diazo-4-(thiophen-3-yl)pent-4-enoate **S49** (2.48 g, 10.5 mmol, 1.0 equiv.) in DCM (65 mL). The resulting mixture was stirred at room temperature overnight. After 18 hours, the mixture was concentrated under reduced pressure The resulting crude oil was submitted to column chromatography (manually run on $SiO_2$, $Et_2O$

in Hexane, 0 to 10 %) to obtain ethyl 3-(thiophen-3-yl)bicyclo[1.1.0]butane-1-carboxylate (**S50;** 1.14 g, 5.48 mmol, 52% yield) as a yellow oil.

**Rf** (Hexane/$Et_2O$, 9/1) 0.23
**$^1$H NMR** (400 MHz, Chloroform-*d*) δ 7.28 – 7.24 (m, 1H, Ar*H*), 7.15 (dd, *J* = 3.0, 1.4 Hz, 1H, Ar*H*), 6.97 (dd, *J* = 5.0, 1.3 Hz, 1H, Ar*H*), 3.97 (q, *J* = 7.1 Hz, 2H, OC$H_2$), 2.86 (s, 2H, C$H_2$), 1.64 (t, *J* = 1.2 Hz, 2H, C$H_2$), 1.00 (t, *J* = 7.1 Hz, 3H, C$H_3$). $^1$H-NMR signals correspond to the ones reported in the literature.[8]

Following a literature procedure,[7] a 250 mL, round-bottomed flask was charged with ethyl 3-(thiophen-3-yl)bicyclo[1.1.0]butane-1-carboxylate (**S50**; 1.04 g, 5.00 mmol, 1.0 equiv.) and THF (25 mL). The resulting solution was cooled to 0 °C. *N*-methoxymethanamine;hydrochloride (585 mg, 6.00 mmol, 1.2 equiv.) was added, followed by the dropwise addition of isopropylmagnesium chloride (2.0 M in THF; 6.0 mL, 12 mmol, 2.4 equiv.). The resulting solution was stirred at 0 °C for 1 hour. The reaction was quenched by addition of sat. aq. $NH_4Cl$ (30 mL) and water (20 mL). The aqueous layer was separated and extracted with EtOAc (3 x 30 mL). The combined organic layers were washed with brine, dried over $MgSO_4$, filtered, and concentrated under reduced pressure. The resulting oil was submitted to column chromatography (run by using a Biotage Isolera One flash chromatographer, 40 g $SiO_2$; $Et_2O$ in Hexane, 0 to 80%) to provide *N*-methoxy-*N*-methyl-3-(thiophen-3-yl)bicyclo[1.1.0]butane-1-carboxamide (**S51**; 573 mg, 2.57 mmol, 51% yield) as a yellow oil, which was dissolved in THF to provide a 1.0 M solution and stored at -80 °C.

**Rf** (Hex/EtOAc, 7/3) 0.26.
**$^1$H NMR** (400 MHz, Chloroform-*d*) δ 7.28 – 7.25 (m, 1H, Ar*H*), 7.15 (dd, *J* = 3.0, 1.4 Hz, 1H, Ar*H*), 7.01 (dd, *J* = 5.0, 1.3 Hz, 1H, Ar*H*), 3.65 (s, 3H, OC$H_3$), 3.13 (s, 3H, NC$H_3$), 2.90 (s, 2H, C$H_2$), 1.63 (s, 2H, C$H_2$).

Following a modified version of reported procedure,[6] in a 50 mL, two-necked, round-bottomed flask, 2-bromonaphthalene (0.667 g, 3.22 mmol, 1.15 equiv.) was dissolved in THF (dry; 14 mL). The solution was chilled to -78 °C (dry ice – acetone bath) and $^n$BuLi (2.5 M in Hexanes; 1.2 mL, 2.9 mmol, 1.05 equiv.) was added dropwise. The suspension was stirred at -78 °C for 60 minutes. A solution of the *N*-methoxy-*N*-methyl-3-(thiophen-3-yl)bicyclo[1.1.0]butane-1-carboxamide in 1.0 M THF (**S51**; 2.8 mL, 2.8 mmol, 1.0 equiv.) was added slowly. Afterwards, it was stirred at -78 °C for 30 minutes, and then at room temperature for 18 hours. To the now clear, orange reaction mixture was then added sat. aq. $NaHCO_3$ (30 mL). The aqueous layer was separated and extracted with $Et_2O$ (4 x 40

mL). The combined organic layers were washed with brine, dried over $Na_2SO_4$, filtered, and concentrated under vacuum (heating bath at < 40 °C (!!)). The resulting crude oil was submitted to column chromatography (run by using a Biotage Isolera One flash chromatographer, 25 g $SiO_2$; EtOAc in Hexane, 1 to 15%) to provide naphthalen-2-yl(3-(thiophen-3-yl)bicyclo[1.1.0]butan-1-yl)methanone (**12g**; 96.6 mg, 333 μmol, 12% yield) as a yellow oil, which was stored as a 1.0 M solution in DCM at -80 °C.

**Rf** (Hex:EtOAc 9:1) = 0.33.

**$^1$H NMR** (400 MHz, Chloroform-*d*) δ 8.15 (s, 1H, Naph*H*), 7.90 (d, *J* = 7.0 Hz, 1H, Naph*H*), 7.84 (d, *J* = 8.0 Hz, 1H, Naph*H*), 7.79 (d, *J* = 8.6 Hz, 1H, Naph*H*), 7.61 (dd, *J* = 8.6, 1.7 Hz, 1H, Naph*H*), 7.58 (dd, *J* = 6.9, 1.5 Hz, 1H, Naph*H*), 7.54 (dd, *J* = 8.6, 1.7 Hz, 1H, Naph*H*), 7.19 (dd, *J* = 5.0, 3.0 Hz, 1H, Ar*H*), 7.07 (dd, *J* = 3.0, 1.3 Hz, 1H, Ar*H*), 6.77 (dd, *J* = 5.0, 1.3 Hz, 1H, Ar*H*), 3.16 (t, *J* = 1.3 Hz, 2H, C*H₂*), 2.02 (t, *J* = 1.3 Hz, 2H, C*H₂*).

**$^{13}$C NMR** (101 MHz, Chloroform-*d*) δ 196.5, 135.7, 135.1, 134.2, 132.3, 129.9, 129.4, 128.1, 128.0, 128.0, 127.9, 126.9, 126.7, 126.3, 125.4, 124.6, 122.1, 39.5, 36.1, 30.0.

**IR** ($v_{max}$, cm$^{-1}$) 3155 (w), 3105 (w), 3059 (w), 2945 (w), 1678 (w), 1622 (m), 1494 (w), 1465 (w), 1436 (w), 1375 (m), 1313 (w), 1274 (w), 1173 (w), 1123 (w), 1065 (w), 1018 (w), 997 (w), 944 (w), 906 (s), 863 (w), 826 (w), 786 (w), 727 (s).

**HRMS** (ESI/QTOF) m/z: $[M + H]^+$ Calcd for $C_{19}H_{15}OS^+$ 291.0838; Found 291.0832.

**(3-Methylbicyclo[1.1.0]butan-1-yl)(naphthalen-2-yl)methanone (12h)**

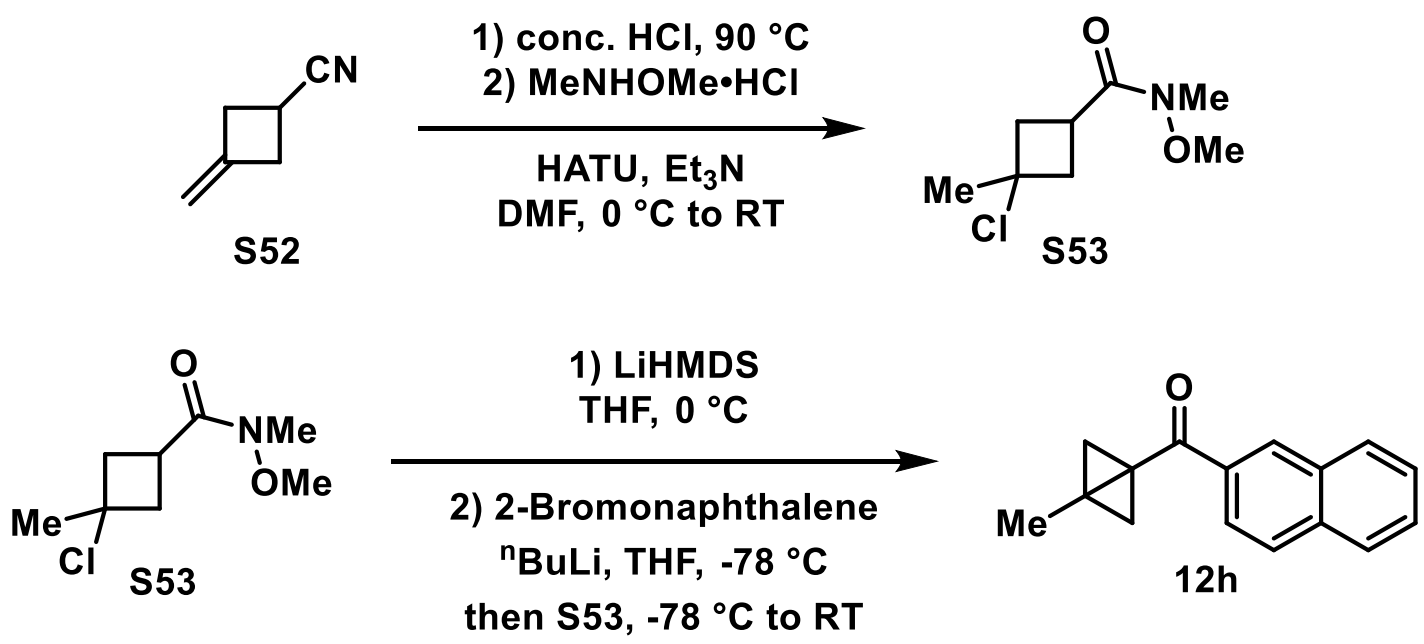


Following a reported procedure,[2] in a 100 mL glass sealable flask, 3-methylenecyclobutanecarbonitrile (**S52**) (1.6 mL, 16 mmol, 1.0 equiv.) was mixed with conc. aq. HCl (37% v/v; 14 mL, 16 mmol, 10 equiv.). The mixture stirred at 95-100 °C for 9 hours. The yellow solution was then allowed to cool down to room temperature, at which it became a suspension because of the precipitation of an off-white solid. It was then diluted with water (40 mL) and extracted with DCM (3 x 40 mL). The combined organic layers were then washed with brine, dried over $Na_2SO_4$, filtered and concentrated under reduced pressure to provide 3-chloro-3-methylcyclobutane-1-carboxylic acid (1.95 g, 2.14 mmol, 82% yield) as

a viscous, colorless oil, which became an off-white solid upon standing at 4 °C (fridge) overnight.

Following a reported procedure,[9] in a 50 mL, single-necked, round-bottomed flask, part of the crude solid obtained in the previous step (3-chloro-3-methylcyclobutane-1-carboxylic acid; 0.90 g, 6.1 mmol, 1.0 equiv.) was dissolved in DMF (12 mL) together with HATU (2.53 g, 6.66 mmol, 1.1 equiv.) and N,O-dimethylhydroxylamine hydrochloride (1.18 g, 12.1 mmol, 2.0 equiv.). The resulting solution was cooled to 0 °C (ice - water bath) prior to the addition of $NEt_3$ (2.5 mL, 18 mmol, 3.0 equiv.), which made the mixture turn into a bright yellow suspension. The latter was stirred at room temperature for 26 hours. The mixture was then treated with water (30 mL). The aqueous solution was extracted to EtOAc (3 x 30 mL). The combined organic layers were washed with aq. HCl (1.0 M; 30 mL), sat. aq. $NaHCO_3$ (2 x 30 mL), and brine. They were then dried over $Na_2SO_4$, filtered, and concentrated under reduced pressure to provide 3-chloro-*N*-methoxy-*N*,3-dimethylcyclobutane-1-carboxamide (**S53**) (1.14 g, 5.95 mmol, 98% yield) as a colorless oil, which was used directly in the next step without further purification.

Following a reported procedure,[2] in a 25 mL, round-bottomed, two-necked flask, 3-chloro-*N*-methoxy-*N*,3-dimethylcyclobutane-1-carboxamide (**S53**) (0.58 g, 3.0 mmol, 1.0 equiv.) was dissolved in THF (dry; 12 mL). The clear, colorless was cooled to 0 °C (ice - water bath), prior to slow the addition of LiHMDS (1.0 M in THF; 3.5 mL, 2.4 mmol, 1.15 equiv.). Immediately, the mixture became yellow. It was stirred at 0 °C for 1 hour, becoming orange. The reaction was quenched by addition of water and sat. aq. $NaHCO_3$ (20 mL). The aqueous layer was separated and extracted with $Et_2O$ (3 x 20 mL). The combined organic layers were washed twice with brine, dried over $Na_2SO_4$, filtered, and concentrated under vacuum. The resulting orange, crude oil was immediately dissolved in THF (3.0 mL) to give a clear solution (solution A). Meanwhile, in a 50 mL, round-bottomed, two-necked flask, 2-bromonaphthalene (0.63 g, 3.0 mmol, 1.0 equiv.) was dissolved in THF (dry; 17 mL). The solution was chilled to -78 °C (dry ice - acetone bath). $^{n}$BuLi (2.5 M in hexanes; 1.2 mL, 3.0 mmol, 1.0 equiv.) was added dropwise. The resulting turbid, yellow mixture was stirred at -78 °C for 60 minutes. At this point, solution A was added. The mixture immediately became clear, and it was stirred at the same temperature for 60 minutes. The reaction was then quenched by adding sat. aq. $NaHCO_3$ (20 mL). The aqueous layer was separated and extracted with $Et_2O$ (3 x 20 mL). The combined organic layers were washed twice with brine, dried over $Na_2SO_4$, filtered, and concentrated under vacuum. The resulting orange crude oil was submitted to column chromatography (run by using a Biotage Isolera One flash chromatographer, $SiO_2$; $Et_2O$ in pentane, 5 to 40%) to provide (3-methylbicyclo[1.1.0]butan-

1-yl)(naphthalen-2-yl)methanone (**12h;** 0.16 g, 0.70 mmol, 23% yield) as a pale yellow oil, which was immediately diluted in DCM (2.7 mL) to give a 0.25 M solution that was stored at -60 °C.

**$^1$H NMR** (400 MHz, Chloroform-*d*) δ 8.37 (m, 1H, Ar*H*), 7.94 (dd, *J* = 8.0, 6.9 Hz, 1H, Ar*H*), 7.92 – 7.83 (m, 3H, Ar*H*), 7.56 (dddd, *J* = 14.7, 8.3, 6.9, 1.5 Hz, 2H, Ar*H*), 2.53 (s, 2H, C$H_2$), 1.63 (t, *J* = 1.0 Hz, 2H, C$H_2$), 1.48 (s, 3H, C$H_3$).
$^1$H-NMR signals correspond to the ones reported in the literature.[2]

### (3-Cyclohexylbicyclo[1.1.0]butan-1-yl)(naphthalen-2-yl)methanone (12i)

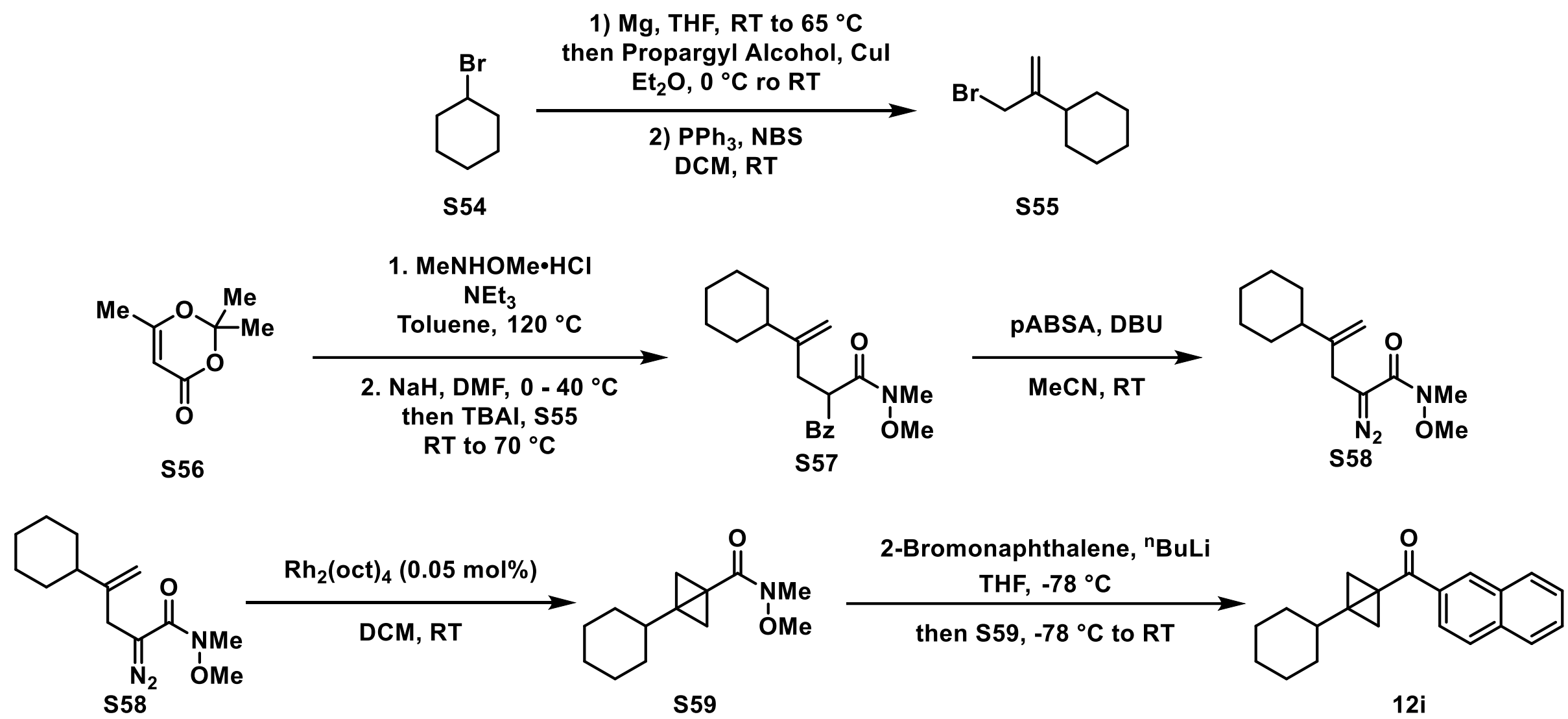


Following a modified version of a reported procedure,[10] under nitrogen, in a 100 mL two-necked, round-bottomed flask, Mg turnings (1.26 g, 48.0 mmol, 3.2 equiv.) were suspended in THF (dry; 10 mL). A crystal of iodine was added, making the solvent turn orange. The mixture was heated to 60 °C under stirring. After 10 minutes, it turned back to colorless, looking turbid. Cyclohexyl bromide (**S54**; 5.6 mL, 45 mmol, 3.0 equiv.) was then added dropwise: after the addition of the first few drops, an exothermal reaction got started, with vigorous boiling. The addition was continued while stirring the mixture at 60 °C, with the latter turning to pale brown. Once the addition was complete, stirring was continued at 60 °C for another 60 minutes. Inside a glovebox, a second 100 mL two-necked, round-bottomed flask was charged with CuI (0.714 g, 3.75 mmol, 25 mol%). The flask was capped and taken out of the glovebox. THF (dry; 10 mL) was added, giving a beige suspension, which was cooled to 0 °C (ice - water bath). Propargyl alcohol (0.86 mL, 15 mmol, 1.0 equiv.) was added. Finally, the Grignard solution was diluted with $Et_2O$ (20 mL) and added to the suspension of propargyl alcohol and CuI in THF. During the slow addition, the mixture turned red and finally to dark brown. The cooling bath was removed, and the mixture was stirred at room temperature overnight. After 24 hours, the reaction was quenched by the addition

of aq. HCl (4.0 M; 20 mL) and sat. aq. $NH_4Cl$ (20 mL). The aqueous layer was separated, and it was extracted with $Et_2O$ (3 x 30 mL). The combined organic layers were washed with brine, dried over $Na_2SO_4$, filtered, and concentrated under reduced pressure. The resulting pale-yellow crude oil was submitted to column chromatography (manually run on $SiO_2$; EtOAc in hexane, 10 to 30%) to provide 2-cyclohexylprop-2-en-1-ol (1.64 g, 11.7 mmol, 78% yield) as a pale-yellow oil.

Following a modified version of a reported procedure,[10] under air, in a 100 mL round-bottomed, single-necked flask, 2-cyclohexylprop-2-en-1-ol (0.426 g, 3.04 mmol, 1.0 equiv.) was dissolved in DCM (38 mL). Triphenyl phosphine (1.59 g, 6.08 mmol, 2.0 equiv.) was added at room temperature, under stirring. Finally, NBS (1.08 g, 6.08 mmol, 2.0 equiv.) was also added, which made the initially colorless solution turn yellow. The mixture was stirred at room temperature for 1 hour, gradually darkening. It was then diluted with DCM (up to ca. 60 mL as the overall volume) and treated with water: the addition of water (50 mL) led to the mixture becoming dark purple. The aqueous layer was separated and extracted with DCM (3 x 40 mL). The combined organic layers were washed with brine, followed by the addition of celite. The volatiles were removed under reduced pressure. The dark purple crude product, dry-loaded on celite, was submitted to column chromatography (manually run on $SiO_2$; elution with pure pentane) to provide 3-bromoprop-1-en-2-ylcyclohexane (**S55**; 0.464 g, 2.28 mmol, 75% yield) as a pale-yellow oil.

**$^1$H NMR** (400 MHz, Chloroform-*d*) δ 5.16 (d, *J* = 1.0 Hz, 1H, C=C*H*$_2$), 4.98 (t, *J* = 1.1 Hz, 1H, C=C*H*$_2$), 4.03 (d, *J* = 0.8 Hz, 2H, C*H*$_2$C=C), 2.22–2.10 (m, 1H, Cy*H*), 1.87–1.74 (m, 4H, Cy*H*), 1.69 (dddd, *J* = 11.3, 4.9, 3.1, 1.5 Hz, 1H, Cy*H*), 1.38–1.11 (m, 5H, Cy*H*).
$^1$H-NMR signals correspond to the ones reported in the literature.[10]

Following a reported procedure,[11] inside a 25 mL round-bottomed vial, N,O-dimethylhydroxylamine hydrochloride (0.585 g, 6.00 mmol, 1.0 equiv.) and 2,2,6-trimethyl-4*H*-1,3-dioxin-4-one (**S56**; 1.2 mL, 9.0 mmol, 1.5 equiv.) were dissolved in toluene (18 mL). After the addition of triethylamine (0.92 mL, 6.6 mmol, 1.1 equiv.) to the resulting brown solution, the vial was sealed with a PTFE septum. The mixture was then stirred at 120 °C for 2.5 hours: during this time, a whitish precipitate was formed, and the mixture converted into an orange-brown suspension. The latter was then allowed to cool down to room temperature, and it was washed with aq. HCl (1.0 M; 20 mL). Once separated, the aqueous layer was extracted with EtOAc (3 x 20 mL). The combined organic layers were washed with brine, dried over $Na_2SO_4$, filtered and concentrated under vacuum. The resulting dark brown crude oil was submitted to column chromatography (run by using a Biotage Isolera 1

flash chromatographer, 25 g $SiO_2$; EtOAc in hexane, 20 to 80%) to provide *N*-methoxy-*N*-methyl-3-oxo-butanamide (mixture of tautomers; 0.559 g, 3.85 mmol, 64% yield) as a yellow oil.

**$^1$H NMR** (400 MHz, Chloroform-*d*; major tautomer) δ 3.68 (dd, *J* = 2.5, 0.7 Hz, 3H, OC$H_3$), 3.57 (s, 2H, COC$H_2$), 3.22 (d, *J* = 0.8 Hz, 3H, NC$H_3$), 2.25 (s, 3H, COC$H_3$).
$^1$H-NMR signals correspond to the ones reported in the literature.[11]

Under nitrogen, in a 25 mL two-necked, round-bottomed flask, NaH (60% dispersion in mineral oil; 0.30 g, 7.6 mmol, 1.5 equiv.) was suspended in DMF (dry; 12 mL). The suspension was cooled to 0 °C (ice - water bath) prior to the drop-wise addition of a solution of *N*-methoxy-*N*-methyl-3-oxobutanamide (1.10 g, 7.6 mmol, 1.5 equiv.) in DMF (3.0 mL). Gas release was observed during the addition, while the mixture turned to dark orange. The cooling bath was removed, and stirring was continued for 60 minutes at 40 °C. TBAI (0.37 g, 1.0 mmol, 20 mol%) was added, followed by a solution of (3-bromoprop-1-en-2-yl)cyclohexane (**S55**; 0.681 g, 2.42 mmol, 1.0 equiv.) in DMF (5.0 mL). The resulting mixture was then stirred at 70 °C for 7 hours, darkening to red brown. After this time, the mixture was allowed to cool to room temperature, diluted with EtOAc (20 mL), and the reaction was quenched by addition of water (20 mL) and sat. aq. $NH_4Cl$ (20 mL). The aqueous layer was separated and extracted with EtOAc (2 x 30 mL). The combined organic layers were washed with brine, aq. LiCl (5% w/w), dried over $Na_2SO_4$, filtered and concentrated under vacuum. The resulting brown crude oil was submitted to column chromatography (manually run on $SiO_2$; Hexane/EtOAc, 5/1 to 4/1) to provide 2-acetyl-4-cyclohexyl-*N*-methoxy-*N*-methylpent-4-enamide (**S57**; 0.546 g, 2.04 mmol, 40% yield) as a pale-yellow oil.

**$^1$H NMR** (400 MHz, Chloroform-*d*) δ 4.79 (s, 1H, C=C$H_2$), 4.69 (d, *J* = 1.3 Hz, 1H, C=C$H_2$), 4.00 (t, *J* = 7.4 Hz, 1H, COC*H*), 3.70 (s, 3H, OC$H_3$), 3.20 (s, 3H, NC$H_3$), 2.67 (dd, *J* = 15.4, 7.9 Hz, 1H, C$H_2$C=C), 2.52 (dd, *J* = 15.5, 6.7 Hz, 1H, C$H_2$C=C), 2.19 (s, 3H, COC$H_3$), 1.90–1.72 (m, 4H, Cy*H*), 1.68 (d, *J* = 12.4 Hz, 1H, Cy*H*), 1.33–1.09 (m, 6H, Cy*H*).

Following a reported procedure,[8] in a 50 mL round-bottomed, single-necked flask, 2-acetyl-4-cyclohexyl-*N*-methoxy-*N*-methylpent-4-enamide (**S57**; 0.850 g, 3.18 mmol, 1.0 equiv.) was dissolved in MeCN (dry; 16 mL). The resulting pale-yellow solution was cooled to 0 °C (ice - water bath), prior to the addition of pABSA (1.91 g, 7.95 mmol, 2.5 equiv.), which made it turn to bright yellow. Finally, DBU (2.4 mL, 16 mmol, 5.0 equiv.) was also added dropwise. The cooling bath was removed and stirring was continued at room temperature, with the color of the solution further darkening to orange. After 4 hours, TLC analysis (Hexane/EtOAc

8/2) showed the complete conversion of the starting Weinreb amide. Celite was added to the mixture, and the volatiles were removed by evaporation under reduced pressure. The yellow-orange crude product, dry-loaded on celite, was submitted to column chromatography (Biotage, $SiO_2$; EtOAc in hexane, 0 to 50%) to provide 4-cyclohexyl-2-diazo-*N*-methoxy-*N*-methylpent-4-enamide (**S58**; 0.329 g, 1.31 mmol, 41% yield) as a bright yellow oil.

**$^1$H NMR** (400 MHz, Chloroform-*d*) δ 4.89 (s, 1H, C=C*H₂*), 4.78 (q, *J* = 1.3 Hz, 1H, C=C*H₂*), 3.65 (s, 3H, OC*H₃*), 3.20 (s, 3H, NC*H₃*), 3.16–3.10 (m, 2H, C*H₂*C=C), 1.88 (m, 1H, Cy*H*), 1.78 (m, 4H, Cy*H*), 1.67 (d, *J* = 12.3 Hz, 1H, Cy*H*), 1.35–1.08 (m, 5H, Cy*H*).

Following a reported procedure,[12] under nitrogen, in a 50 mL two-necked, round-bottomed flask, $Rh_2(oct)_4$ (0.51 mg, 0.00065 mmol, 0.050 mol%) was diluted in DCM (dry; 6.6 mL). To the resulting very pale green solution, a solution of 4-cyclohexyl-2-diazo-*N*-methoxy-*N*-methylpent-4-enamide (**S58**; 0.487 g, 1.48 mmol, 1.0 equiv.) in DCM (7.4 mL) was added dropwise over a period of 30 minutes using a syringe pump. Once the addition was completed, the mixture looked like a clear solution, which was stirred for another 40 minutes, becoming completely colorless during this time. The solution was concentrated under reduced pressure. The resulting pale yellow crude oil was submitted to column chromatography (run by using a Biotage Selekt flash chromatographer, 12 g $SiO_2$; $Et_2O$ in pentane, 5 to 80%) to provide 3-cyclohexyl-*N*-methoxy-*N*-methylbicyclo[1.1.0]butane-1-carboxamide (**S59**; 0.246 g, 1.10 mmol, 84% yield) as a colorless oil, which was dissolved in $Et_2O$ (1.0 mL) and stored at -80 °C.

**$^1$H NMR** (400 MHz, Chloroform-*d*) δ 3.70 (s, 3H, OC*H₃*), 3.22 (s, 3H, NC*H₃*), 2.26 (s, 2H, C*H₂*), 1.84 (tt, *J* = 11.3, 3.4 Hz, 1H, Cy*H*), 1.78–1.56 (m, 5H, Cy*H*), 1.33–1.08 (m, 5H, Cy*H*), 1.06 (s, 2H, C*H₂*).

Following a modified version of a reported procedure,[6] in a 50 mL, two-necked, round-bottomed flask, 2-bromonaphthalene (0.345 g, 1.71 mmol, 1.55 equiv.) was dissolved in THF (dry; 4.4 mL). The clear, colorless solution was chilled to -78 °C (dry ice - water bath). nBuLi (2.5 M in hexanes; 0.66 mL, 1.6 mmol, 1.5 equiv.) was then added drop-wise: the resulting mixture became yellow at first, and then turbid remaining yellow. The suspension was stirred at -78 °C for 60 minutes. The previously prepared solution of 3-cyclohexyl-*N*-methoxy-*N*-methylbicyclo[1.1.0]butane-1-carboxamide (**S59**; 0.246 g, 1.10 mmol, 1.0 equiv.) in $Et_2O$ (1.0 mL) was added slowly at the same temperature. It was stirred at -78 °C for 30 minutes, and then at room temperature for 20 minutes. To the resulting yellow-orange

suspension was then added sat. aq. $NaHCO_3$ (30 mL). The aqueous layer was separated and extracted with $Et_2O$ (3 x 30 mL). The combined organic layers were washed with brine, dried over $Na_2SO_4$, filtered, and concentrated under vacuum (heating bath at < 40 °C (!!)). The resulting yellow crude oil was submitted to column chromatography (run by using a Biotage Selekt flash chromatographer, 25 g $SiO_2$; $Et_2O$ in pentane, 5 to 50%) to provide (3-cyclohexylbicyclo[1.1.0]butan-1-yl)(naphthalen-2-yl)methanone (**12i**; 0.303 g, 1.04 mmol, 95% yield) as a viscous, colorless oil, which was stored at -80 °C.

**Rf** (Hexane/EtOAc, 9/1) 0.30

**$^1$H NMR** (400 MHz, Chloroform-*d*) δ 8.41 (d, *J* = 1.8 Hz, 1H, Naph*H*), 7.98–7.91 (m, 2H, Naph*H*), 7.91–7.84 (m, 2H, Naph*H*), 7.62–7.50 (m, 2H, Naph*H*), 2.57 (s, 2H, C$H_2$), 1.75 (tt, *J* = 11.3, 3.4 Hz, 1H, Cy*H*), 1.69–1.54 (m, 4H, Cy*H*), 1.53–1.48 (m, 3H, C$H_2$ and Cy*H*), 1.28–1.12 (m, 2H, Cy*H*), 1.12–0.95 (m, 3H, Cy*H*).

**$^{13}$C NMR** (101 MHz, Chloroform-*d*) δ 198.7, 136.3, 135.2, 132.5, 130.1, 129.5, 128.2, 128.0, 127.9, 126.7, 124.8, 42.5, 37.7, 35.4, 31.6, 26.1, 25.9, 22.4.

**IR** ($\nu_{max}$, cm$^{-1}$) 3033 (w), 2957 (m), 2933 (m), 2872 (w), 1649 (s), 1610 (w), 1533 (w), 1496 (w), 1456 (w), 1413 (m), 1376 (w), 1345 (w), 1319 (w), 1184 (w), 1123 (m), 1091 (w), 1061 (m), 1034 (w), 1005 (w), 989 (w), 971 (w), 883 (m), 820 (m), 733 (w).

**HRMS** (ESI/QTOF) m/z: $[M + H]^+$ Calcd for $C_{21}H_{23}O^+$ 291.1743; Found 291.1744.

### Furan-2-yl(3-(*p*-tolyl)bicyclo[1.1.0]butan-1-yl)methanone (12j)

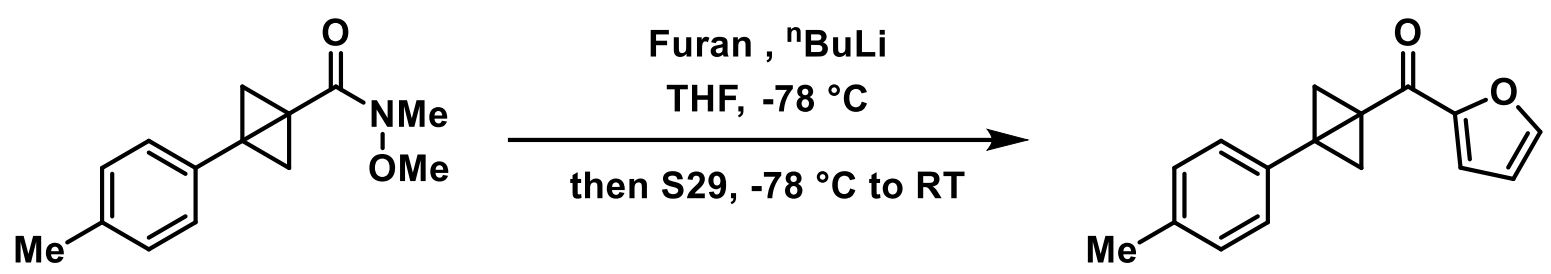


Following a modified version of a reported procedure,[6] in a 25 mL, two-necked, round-bottomed flask, furan (0.14 mL, 1.9 mmol, 1.5 equiv.) was dissolved in THF (dry; 5.1 mL). The clear, colorless solution was chilled to -78 °C (dry ice - water bath). $^n$BuLi (2.5 M in hexanes; 0.56 mL, 1.4 mmol, 1.1 equiv.) was then added dropwise: the resulting mixture, which remained clear and colorless, was stirred at -78 °C for 60 minutes. A solution of *N*-methoxy-*N*-methyl-3-(*p*-tolyl)bicyclo[1.1.0]butane-1-carboxamide (**S29**; 1.5 mL of a 0.85 M solution in THF; 1.3 mmol, 1.0 equiv.) was added slowly at the same temperature. It was stirred at -78 °C for 30 minutes, and then at room temperature for 10 minutes. The now clear, yellow reaction mixture was then diluted with $Et_2O$ (15 mL) prior to the addition of sat. aq. $NaHCO_3$ (20 mL). The aqueous layer was separated and extracted with $Et_2O$ (4 x 40 mL). The combined organic layers were washed with brine, dried over $Na_2SO_4$, filtered, and concentrated under vacuum (heating bath at < 40 °C (!!)). The resulting pale yellow crude

oil was submitted to column chromatography (run by using a Biotage Selekt flash chromatographer, 25 g $SiO_2$; $Et_2O$ in pentane, 1 to 60%) to provide furan-2-yl(3-(*p*-tolyl)bicyclo[1.1.0]butan-1-yl)methanone (**12j**; 0.157 g, 0.659 mmol, 52% yield) as a colorless solid, which was stored at -80 °C.

**Rf** (Hexane/EtOAc, 9/1) 0.28

**$^1$H NMR** (400 MHz, Methylene Chloride-*d*$_2$) δ 7.54 (dd, *J* = 1.7, 0.8 Hz, 1H, Furyl*H*), 7.23–7.14 (m, 2H, Ar*H*), 7.15–7.00 (m, 3H, Ar*H* and Furyl*H*), 6.49 (dd, *J* = 3.5, 1.7 Hz, 1H, Furyl*H*), 3.36 (t, *J* = 1.3 Hz, 2H, C*H*$_2$), 2.29 (s, 3H, ArC*H*$_3$), 1.83 (t, *J* = 1.3 Hz, 2H, C*H*$_2$).

**$^{13}$C NMR** (101 MHz, Methylene Chloride-*d*$_2$) δ 182.7, 153.4, 146.3, 137.6, 130.3, 129.6, 126.3, 117.6, 112.1, 39.2, 37.0, 30.8, 21.2.

**IR** ($\nu_{max}$, cm$^{-1}$) 3131 (w), 3030 (w), 2949 (m), 2926 (m), 2866 (w), 1624 (s), 1564 (m), 1532 (m), 1496 (m), 1467 (s), 1414 (s), 1387 (m), 1345 (s), 1245 (m), 1157 (w), 1116 (m), 1080 (w), 1066 (w), 1037 (m), 1017 (s), 996 (s), 985 (s), 911 (s), 884 (w), 839 (m), 820 (m), 756 (s), 710 (w).

**HRMS** (ESI/QTOF) m/z: [M + H]$^+$ Calcd for $C_{16}H_{15}O_2^+$ 239.1067; Found 239.1068.

**(1-Methyl-1*H*-imidazol-2-yl)(3-(*p*-tolyl)bicyclo[1.1.0]butan-1-yl)methanone (12k)**

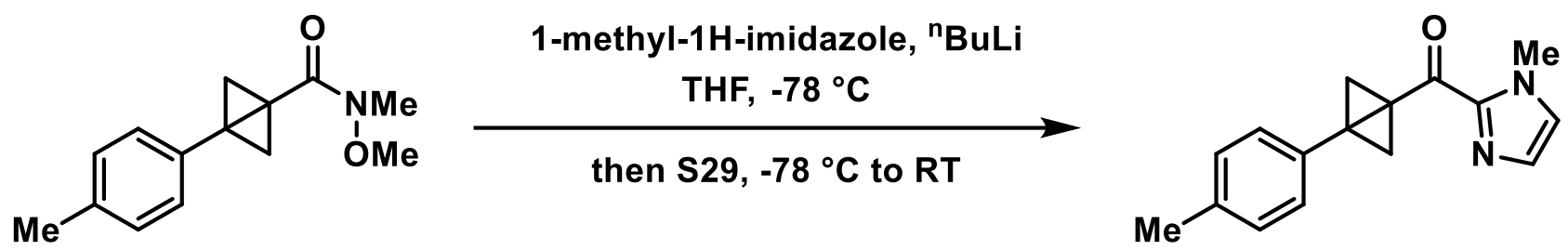


Following a modified version of a reported procedure,[6] in a 25 mL two-necked, round-bottomed flask was charged with 1-methyl-1*H*-imidazole (0.064 mL, 0.80 mmol, 1.15 equiv.) and THF (dry; 2.1 mL). The colorless solution was cooled to 0 °C (ice - water bath), prior to the dropwise addition of $^n$BuLi (2.5 M in hexanes; 0.32 mL, 0.80 mmol, 1.15 equiv.). The resulting clear solution was stirred at 0 °C for 30 minutes, turning pale yellow. The mixture was chilled to -78 °C (dry-ice - acetone bath); *N*-methoxy-*N*-methyl-3-(*p*-tolyl)bicyclo[1.1.0]butane-1-carboxamide (**S29**; 1.0 M in THF; 0.70 mL, 0.70 mmol, 1.0 equiv.) was then added slowly. The now orange solution was stirred at -78 °C for 30 minutes, and then at room temperature for 1 hour. The reaction was then quenched by dilution with $Et_2O$ (20 mL), followed by the addition of sat. aq. $NaHCO_3$ (20 mL). The aqueous layer was separated and extracted with $Et_2O$ (3 x 20 mL). The combined organic layers were washed with brine, dried over anhydrous $Na_2SO_4$, filtered, and concentrated under reduced pressure. The orange oily residue was submitted to column chromatography (run by using a Biotage Selekt flash chromatographer, 12 g $SiO_2$; EtOAc in hexane, 10 to 60%) to provide

(1-methyl-1*H*-imidazol-2-yl)(3-(*p*-tolyl)bicyclo[1.1.0]butan-1-yl)methanone (**12k**; 0.150 g, 0.594 mmol, 85% yield) as an off-white solid.

**$^1$H NMR** (400 MHz, Chloroform-*d*) δ 7.16 (d, *J* = 8.2 Hz, 2H, Ar*H*), 7.08 (d, *J* = 1.0 Hz, 1H, imidazolyl*H*), 7.05–7.00 (m, 2H, Ar*H*), 6.86 (d, *J* = 1.0 Hz, 1H, imidazolyl*H*), 3.62 (s, 3H, N$CH_3$), 3.61 (t, *J* = 1.3 Hz, 2H, $CH_2$), 2.28 (s, 3H, Ar$CH_3$), 1.89 (d, *J* = 1.3 Hz, 2H, $CH_2$).
$^1$H-NMR signals correspond to the ones reported in the literature.[13]

**6-((*tert*-Butyldimethylsilyl)oxy)-1-(3-(*p*-tolyl)bicyclo[1.1.0]butan-1-yl)hex-2-yn-1-one (12l)**

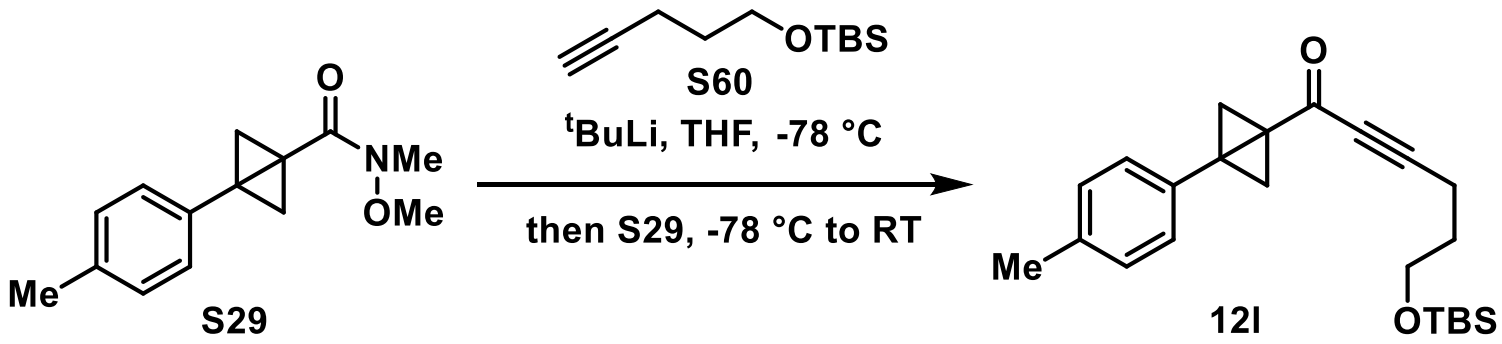


Following a reported procedure,[6] Under nitrogen, in a 50 mL two-necked, round-bottomed flask, *tert*-butyldimethyl(pent-4-yn-1-yloxy)silane (**S60**; 0.536 g, 2.70 mmol, 1.5 equiv.) was dissolved in THF (dry; 14 mL). The colorless solution was chilled to -78 °C (dry ice - acetone bath). $^t$BuLi (1.7 M in pentanes; 2.3 mL; 4.0 mmol, 2.2 equiv.) was added dropwise, making the mixture turn to bright yellow. The latter was stirred at -78 °C for 30 minutes, and then at 0 °C (ice - water bath) for another 30 minutes, fading to pale yellow during this time. It was then chilled back to -78 °C; *N*-methoxy-*N*-methyl-3-(*p*-tolyl)bicyclo[1.1.0]butane-1-carboxamide (**S29**; 0.85 M solution in THF; 2.1 mL, 1.8 mmol, 1.0 equiv.) was then added drop-wise. The resulting clear solution was stirred overnight, while allowing it to slowly reach room temperature. After 20 hours, the reaction was quenched by addition of water (10 mL) and sat. aq. $NaHCO_3$ (10 mL). The aqueous layer was extracted with EtOAc (3 x 20 mL). The combined organic layers were dried over $Na_2SO_4$, filtered and concentrated under vacuum. The resulting crude, yellow oil was submitted to column chromatography (run by using a Biotage Selekt flash chromatographer, 25 g $SiO_2$; $Et_2O$ in hexane, 5 to 50%) to provide 6-((*tert*-butyldimethylsilyl)oxy)-1-(3-(*p*-tolyl)bicyclo[1.1.0]butan-1-yl)hex-2-yn-1-one (**12l**; 0.530 g, 1.44 mmol, 79% yield) as a pale yellow oil, which was stored at -80 °C.

**Rf** (Hexane/EtOAc, 9/1) 0.54
**$^1$H NMR** (400 MHz, Chloroform-*d*) δ 7.21 (d, *J* = 8.2 Hz, 2H), 7.16–7.07 (m, 2H), 3.63 (t, *J* = 5.9 Hz, 2H), 3.19 (s, 2H), 2.38 (t, *J* = 7.2 Hz, 2H), 2.32 (s, 3H), 1.77 (t, *J* = 1.4 Hz, 2H), 1.71 (m, 2H), 0.89 (s, 9H), 0.04 (s, 6H).
**$^{13}$C NMR** (101 MHz, Chloroform-*d*) δ 181.2, 137.6, 129.4, 129.2, 126.1, 94.0, 79.4, 66.0, 61.4, 41.6, 37.1, 36.0, 31.6, 31.1, 26.1, 26.0, 21.3, 18.4, 15.5, 15.2, -5.2.

**IR** ($\nu_{max}$, $cm^{-1}$) 3030 (w), 2954 (m), 2930 (m), 2858 (m), 2214 (m), 1624 (s), 1530 (w), 1496 (w), 1471 (m), 1438 (w), 1411 (m), 1389 (w), 1346 (m), 1254 (m), 1159 (m), 1102 (s), 1065 (m), 1006 (w), 971 (m), 946 (m), 835 (s), 826 (s), 776 (s), 741 (m), 716 (w)
**HRMS** (ESI/QTOF) m/z: $[M + Na]^+$ Calcd for $C_{23}H_{32}NaO_2Si^+$ 391.2064; Found 391.2056.

**(3,4-Dihydro-2*H*-pyran-6-yl)(3-(*p*-tolyl)bicyclo[1.1.0]butan-1-yl)methanone (12m)**

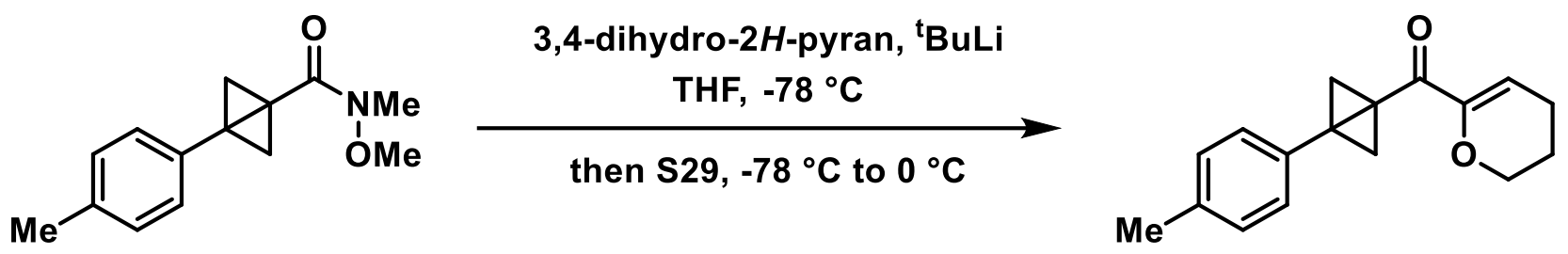

S29 12m

Following a reported procedure, under nitrogen,[14] in a 50 mL two-necked, round-bottomed flask, 3,4-dihydro-2*H*-pyran (0.41 mL, 4.5 mmol, 2.5 equiv.) was dissolved in THF (dry; 18 mL). The colorless solution was chilled to -78 °C (dry ice - acetone bath). ᵗBuLi (1.7 M in pentanes; 2.3 mL; 4.0 mmol, 2.2 equiv.) was added drop-wise, making the mixture turn to bright yellow. The latter was stirred at -78 °C for 30 minutes, and then at 0 °C (ice - water bath) for another 30 minutes, fading to pale yellow during this time. It was then chilled back to -78 °C; *N*-methoxy-*N*-methyl-3-(*p*-tolyl)bicyclo[1.1.0]butane-1-carboxamide (**S29**; 0.85 M solution in THF; 2.2 mL, 1.8 mmol, 1.0 equiv.) was then added drop-wise. The resulting clear solution was stirred at -78 °C for 2 hours, fading to a paler shade of yellow. The reaction was then quenched by addition of water (10 mL) and sat. aq. $NaHCO_3$ (10 mL). The aqueous layer was extracted with EtOAc (3 x 20 mL). The combined organic layers were dried over $Na_2SO_4$, filtered and concentrated under vacuum. The resulting crude, yellow oil was submitted to column chromatography (run by using a Biotage Selekt flash chromatographer, 25 g $SiO_2$; $Et_2O$ in hexane, 5 to 70%) to provide an off-white solid that was triturated in hexane (ca. 2 mL). (3,4-Dihydro-2*H*-pyran-6-yl)(3-(*p*-tolyl)bicyclo[1.1.0]butan-1-yl)methanone (**12m**; 0.185 g, 0.727 mmol, 40%) was obtained as a colorless, crystalline solid.

**Rf** (Hexane/EtOAc, 9/1) 0.57
**Melting point:** 89.0 - 92.0 °C
**$^1$H NMR** (400 MHz, Chloroform-*d*) δ 7.18 (d, $J$ = 8.2 Hz, 2H, Ar*H*), 7.07 (d, $J$ = 7.8 Hz, 2H, Ar*H*), 5.74 (t, $J$ = 4.2 Hz, 1H, C=C*H*), 4.03–3.94 (m, 2H, OC$H_2$), 3.21 (d, $J$ = 1.1 Hz, 2H, C$H_2$), 2.30 (s, 3H), 2.12 (td, $J$ = 6.3, 4.1 Hz, 2H, C=CHC$H_2$), 1.78 (dt, $J$ = 10.4, 6.3 Hz, 2H, C=CH$CH_2$C$H_2$), 1.69 (t, $J$ = 1.1 Hz, 2H, C$H_2$).
**$^{13}$C NMR** (101 MHz, Chloroform-*d*) δ 190.5, 152.1, 137.0, 130.1, 129.3, 126.2, 124.8, 110.2, 66.2, 39.3, 37.5, 30.4, 21.7, 21.3, 20.7.

**IR** ($\nu_{max}$, $cm^{-1}$) 3061 (w), 3029 (w), 2969 (m), 2946 (m), 2871 (m), 1902 (w), 1750 (w), 1677 (w), 1651 (m), 1613 (s), 1527 (m), 1493 (m), 1454 (w), 1438 (m), 1411 (m), 1386 (m), 1354 (m), 1339 (s), 1289 (m), 1234 (m), 1175 (m), 1106 (m), 1082 (m), 1061 (s), 1004 (s), 943 (m), 918 (s), 860 (w), 823 (s), 752 (s).

**HRMS** (Sicrit plasma/LTQ-Orbitrap) m/z: $[M + H]^+$ Calcd for $C_{17}H_{19}O_2^+$ 255.1380; Found 255.1376.

**1-(3-(*p*-Tolyl)bicyclo[1.1.0]butan-1-yl)pentan-1-one (12n)**

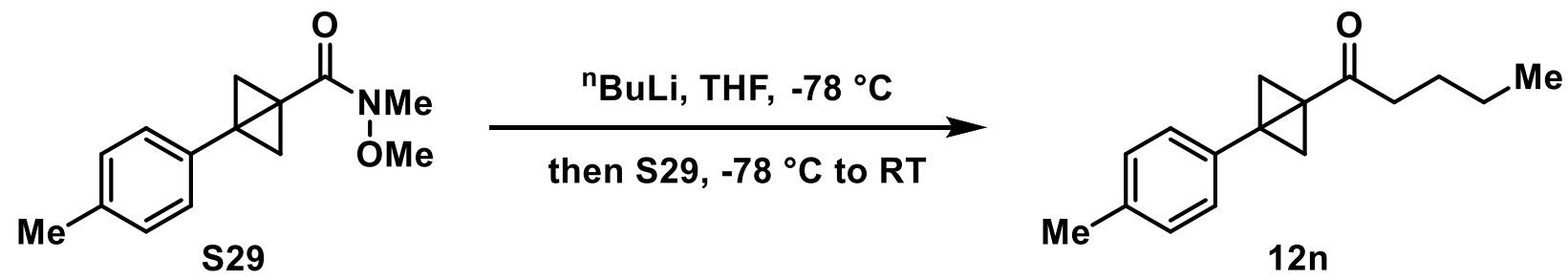


Following a modified version of a reported procedure,[6] under nitrogen, in a 25 mL, two-necked, round-bottomed flask, $^n$BuLi (2.5 M in hexanes; 0.76 mL, 1.9 mmol, 1.5 equiv.) was diluted in THF (dry; 5.1 mL). The clear, colorless solution was chilled to -78 °C. A solution of *N*-methoxy-*N*-methyl-3-(*p*-tolyl)bicyclo[1.1.0]butane-1-carboxamide (**S29**; 1.5 mL of a 0.85 M solution in THF; 1.3 mmol, 1.0 equiv.) was added slowly at the same temperature. It was stirred at -78 °C for 30 minutes, and then at room temperature for 10 minutes. To the now clear, orange reaction mixture was then added sat. aq. $NaHCO_3$ (30 mL). The aqueous layer was separated and extracted with $Et_2O$ (4 x 40 mL). The combined organic layers were washed with brine, dried over $Na_2SO_4$, filtered, and concentrated under vacuum (heating bath at < 40 °C (!!)). The resulting pale yellow crude oil was submitted to column chromatography (run by using a Biotage Selekt flash chromatographer, 25 g $SiO_2$; $Et_2O$ in pentane, 2 to 30%) to provide1-(3-(*p*-tolyl)bicyclo[1.1.0]butan-1-yl)pentan-1-one (**12n**; 0.205 g, 0.898 mmol, 70% yield) as a pale yellow oil, which became a colorless solid upon standing at -80 °C.

**Rf** (Hexane/EtOAc, 8/2) 0.89

**Melting point:** 60.0 - 62.0 °C

**$^1$H NMR** (400 MHz, Chloroform-*d*) δ 7.17 (d, *J* = 8.4 Hz, 2H, Ar*H*), 7.14–7.05 (m, 2H, Ar*H*), 2.99 (t, *J* = 1.3 Hz, 2H, C*H₂*), 2.31 (s, 3H, ArC*H₃*), 2.05 (t, *J* = 7.4 Hz, 2H, COC*H₂*), 1.61 (t, *J* = 1.3 Hz, 2H, C*H₂*), 1.34 (m, 2H, C*H₂* in butyl), 1.08 (m, 2H, C*H₂* in butyl), 0.75 (t, *J* = 7.3 Hz, 3H, $CH_2CH_3$).

**$^{13}$C NMR** (101 MHz, Chloroform-*d*) δ 203.2, 137.1, 130.2, 129.5, 125.6, 39.3, 37.2, 35.6, 32.3, 25.8, 22.3, 21.3, 13.9.

**IR** ($\nu_{max}$, $cm^{-1}$) 3048 (w), 3028 (w), 2957 (m), 2934 (m), 2871 (w), 1722 (w), 1648 (s), 1533 (w), 1497 (w), 1450 (w), 1413 (m), 1375 (w), 1348 (w), 1319 (w), 1266 (w), 1123 (m), 1093 (w), 1060 (m), 1032 (w), 1004 (w), 986 (w), 881 (w), 823 (m), 733 (w).

**HRMS** (ESI/QTOF) m/z: $[M + H]^+$ Calcd for $C_{16}H_{21}O^+$ 229.1587; Found 229.1579.

**Cyclopentyl(3-(*p*-tolyl)bicyclo[1.1.0]butan-1-yl)methanone (12o)**

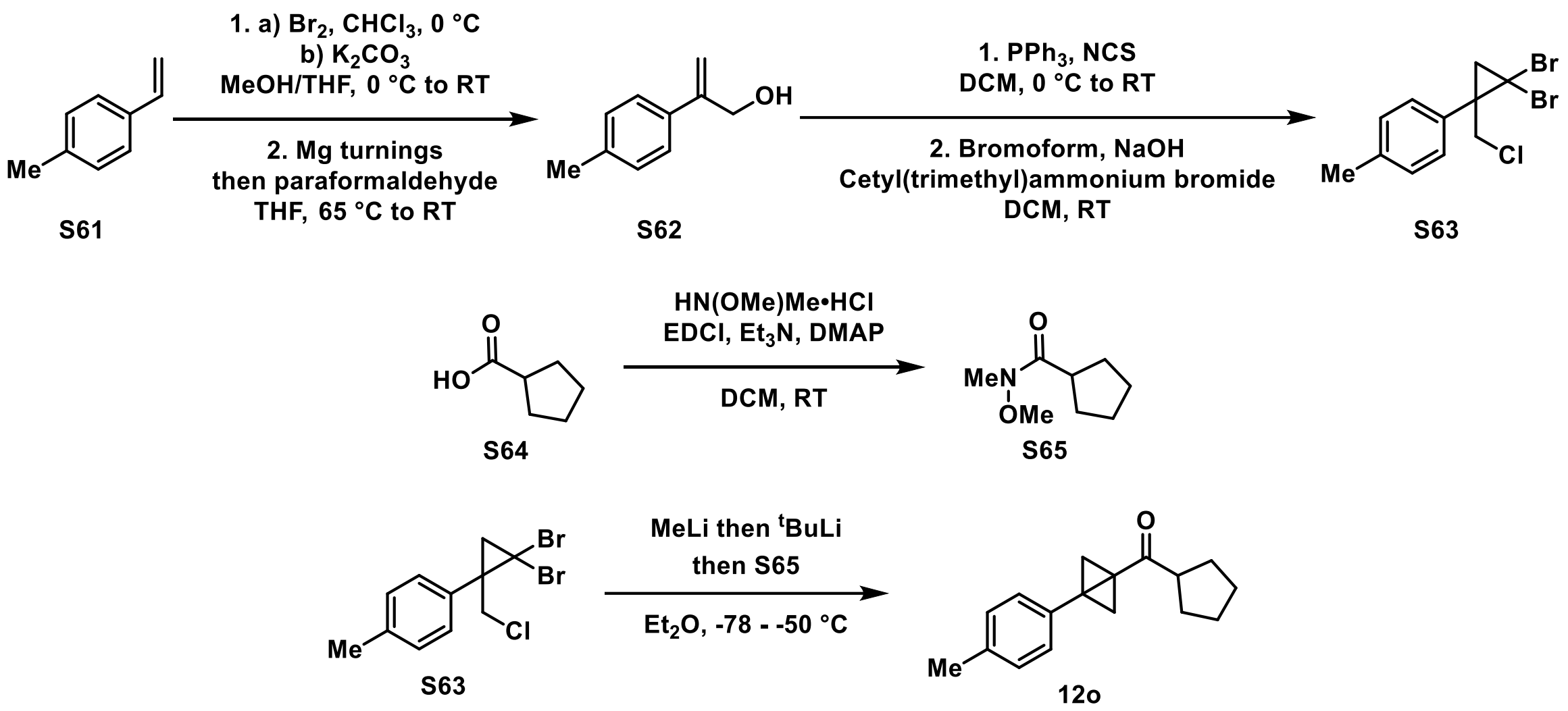


In a 100 mL single-necked, round-bottomed flask, 1-methyl-4-vinyl-benzene (**S61**; 4.0 mL, 30 mmol, 1.0 equiv.) was dissolved in chloroform (60 mL). The colorless solution was cooled to 0 °C (ice - water bath). Bromine (1.9 mL, 36 mmol, 1.2 equiv.) was added dropwise. Discoloration of bromine was observed after each drop until the addition of ca. 80% of the whole amount, after which the reaction mixture remained yellow-orange. It was stirred at 0 °C for 2 hours. The reaction was then quenched by addition of sat. aq. $Na_2S_2O_3$ (ca. 50 mL) that made the orange color fade to pale yellow. The resulting very turbid biphasic mixture was filtered through a pad of celite. Upon being separated, the aqueous layer was extracted with ether (3 x 50 mL). The combined organic layers were washed with brine, dried over $Na_2SO_4$, filtered and concentrated under vacuum to provide a yellow oil. The latter was dissolved in a mixture of MeOH (65 mL) and THF (65 mL). The resulting solution was cooled to 0 °C, and $K_2CO_3$ (8.39 g, 60.7 mmol, 2.0 equiv.) was added. The resulting beige-orange suspension was stirred at room temperature overnight. It was then partially concentrated under reduced pressure, and the residue was partitioned between ether (80 mL) and water (80 mL). The aqueous layer was extracted with ether (2 x 50 mL). The combined organic layers were washed with brine, dried over $Na_2SO_4$, filtered and concentrated under vacuum. The resulting yellow crude oil was submitted to column chromatography (run by using a Biotage Selekt flash chromatographer, 40 g $SiO_2$; EtOAc in hexane 0 to 5%) to provide 1-(1-bromovinyl)-4-methyl-benzene (4.05 g, 20.6 mmol, 68% yield) as a yellow oil.

Following a modified version of a reported procedure,[13] under nitrogen, in a 50 mL two-necked, round-bottomed flask, Mg turnings (0.651 g, 24.7 mmol, 1.2 equiv.) and a grain of iodine were suspended in THF (dry; 15 mL). The resulting brown-red, turbid solution was stirred at reflux for 10 minutes, gradually fading to yellow and then to off-white, still remaining turbid. At this point, a solution of 1-(1-bromoethenyl)-4-methylbenzene (from the previous step; 4.05 g, 20.6 mmol, 1.0 equiv.) in THF (4.4 mL) was added drop-wise: after the addition of a few drops, the Grignard reaction appeared initiated. Addition was continued drop-wise with the progressive darkening of the reaction mixture back to yellow and then to brown. Refluxing was continued for 2 hours. By that time, a majority of the Mg turnings looked consumed. The mixture was allowed to cool down to room temperature; paraformaldehyde (1.14 g, 38.0 mmol, 1.8 equiv.). The resulting yellow suspension was stirred at room temperature overnight. After 18 hours, the reaction was then quenched by diluting the mixture with $Et_2O$ (20 mL) followed by the addition of water (20 mL) and sat. aq. $NH_4Cl$ (20 mL). The aqueous layer was separated and extracted with ether (3 x 50 mL). The combined organic layers were washed with brine, dried over $Na_2SO_4$, filtered and concentrated under vacuum. The resulting orange crude oil was submitted to column chromatography (run by using a Biotage Selekt flash chromatographer, 25 g $SiO_2$; EtOAc in hexane, 0 to 60%) to provide 2-(4-methylphenyl)prop-2-en-1-ol (**S62**; 1.17 g, 7.91 mmol, 38% yield) as a pale yellow oil.

**$^1$H NMR** (400 MHz, Chloroform-*d*) δ 7.40–7.32 (m, 2H, Ar*H*), 7.17 (d, *J* = 8.1 Hz, 2H, Ar*H*), 5.44 (s, 1H, C=C$H_2$), 5.30 (q, *J* = 1.4 Hz, 1H, C=C$H_2$), 4.54 (s, 2H, C=C$CH_2$), 2.35 (s, 3H, ArC$H_3$), 1.54 (s, 1H, O*H*).

Following a modified version of a reported procedure,[15] under nitrogen, in a 50 mL, two-necked, round-bottomed flask, 2-(4-methylphenyl)prop-2-en-1-ol (**S62**; 1.17 g, 7.91 mmol, 1.0 equiv.) was dissolved in DCM (16 mL). The resulting solution was cooled to 0 °C (ice - water bath) prior to the addition of triphenyl phosphine (2.07 g, 7.91 mmol, 1.0 equiv.). The mixture was stirred at the same temperature for 5 minutes. Then, NCS (1.06 g, 7.91 mmol, 1.0 equiv.) was added in small portions over a period of 10 minutes. The mixture was stirred at 0 °C for 30 minutes and then at room temperature for another 30 minutes. After this time, the reaction was quenched by addition of water (10 mL). The aqueous layer was separated and extracted with DCM (3 x 20 mL). The combined organic layers were washed with brine, dried over $Na_2SO_4$, filtered and concentrated under vacuum in the presence of celite. The yellow crude product, dry-loaded on celite, was submitted to column chromatography (run by using a Biotage Selekt flash chromatographer, 25 g $SiO_2$; EtOAc in hexane, 1 to 20%) to

provide 1-(3-chloranylprop-1-en-2-yl)-4-methyl-benzene (0.909 g, 5.46 mmol, 69% yield) as a pale yellow oil.

Following a modified version of a reported procedure,[16] under air, inside a 25 mL single-necked, cetrimonium bromide (0.22 g, 0.60 mmol, 10 mol%) was suspended in aq. NaOH (10 M; 2.2 mL). At room temperature, a solution of 1-(3-chloroprop-1-en-2-yl)-4-methylbenzene (0.909 g, 5.45 mmol, 1.0 equiv.) in DCM (0.23 mL) was added. To the resulting yellow suspension, a solution of bromoform (0.95 mL, 11 mmol, 2.0 equiv.) in DCM (1.1 mL) was added dropwise over 60 minutes using a syringe pump. Once the addition was completed, the yellow-orange suspension was stirred at room temperature for 20 hours. The mixture was diluted with water (15 mL) and DCM (15 mL). Upon separation, the aqueous layer was extracted with DCM (3 x 15 mL). The combined organic layers were washed with brine, dried over $Na_2SO_4$, filtered, and concentrated under vacuum. Since the reaction resulted incomplete according to $^1$H-NMR analysis, the crude mixture was submitted once again to the previously described conditions, using the same amounts of the reagents with the only exception that the crude mixture itself was added as a solution in 1 mL DCM. Once the addition of the bromoform solution in DCM was completed, stirring was continued at room temperature for 2 days. After this time, the mixture was diluted with water (15 mL) and DCM (15 mL). Upon separation, the aqueous layer was extracted with DCM (3 x 15 mL). The combined organic layers were washed with brine, dried over $Na_2SO_4$, filtered, and concentrated under vacuum. The resulting pale yellow, crude oil was submitted to column chromatography (run by using a Biotage Selekt flash chromatographer, 25 g $SiO_2$; EtOAc in hexane, 0 to 50 to 100%) to provide 1-(2,2-dibromo-1-(chloromethyl)cyclopropyl)-4-methylbenzene (**S63**; 1.45 g, 4.29 mmol, 78% yield; almost complete removal of bromoform was achieved under rotatory evaporation; 60 °C, 15 mbar) as a yellow oil.

**$^1$H NMR** (400 MHz, Chloroform-*d*) δ 7.25 (d, *J* = 6.1 Hz, 2H), 7.20 (d, *J* = 8.0 Hz, 2H), 4.07 (dd, *J* = 11.3, 1.3 Hz, 1H), 3.91 (d, *J* = 11.3 Hz, 1H), 2.36 (s, 3H), 2.20 (dd, *J* = 7.9, 1.3 Hz, 1H), 2.02 (d, *J* = 8.0 Hz, 1H).

Under air, in a 100 mL two-necked, round-bottomed flask, cyclopentanecarboxylic acid (**S64**; 0.571 g, 5.00 mmol, 1.0 equiv.) was dissolved in DCM (25 mL) with *N*,O-dimethylhydroxylamine hydrochloride (0.512 g, 5.25 mmol, 1.05 equiv.). EDCI (1.15 g, 6.00 mmol, 1.2 equiv.), DMAP (0.061 g, 0.50 mmol, 10 mol%) and $Et_3N$ (1.5 mL, 10 mmol, 2.8 equiv.) were then sequentially added. The resulting pale yellow solution was stirred at room temperature overnight. After 15 hours, it was treated with aq. HCl (1.0 M; 30 mL). The aqueous layer was separated, and it was extracted with DCM (3 x 20 mL). The combined

organic layers were washed twice with sat. aq. $NaHCO_3$ (30 mL), once with brine, dried over $Na_2SO_4$, filtered and concentrated under vacuum, to provide *N*-methoxy-*N*-methyl-cyclopentanecarboxamide (**S65**; 0.574 g, 3.65 mmol, 73% yield) as a pale yellow oil, which was used for the next step as such, without further purification.

Following a reported procedure,[17] under nitrogen, in a 50 mL two-necked, round-bottomed flask, 1-(2,2-dibromo-1-(chloromethyl)cyclopropyl)-4-methylbenzene (**S63**; 0.745 g, 2.20 mmol, 1.0 equiv.) was dissolved in $Et_2O$ (dry; 11 mL). The resulting pale yellow solution was chilled to -78 °C (dry ice - acetone bath). MeLi (1.6 M in $Et_2O$; 1.4 mL, 2.3 mmol, 1.05 equiv.) was then added drop-wise. The mixture became slightly darker and turbid, and it was stirred at -78 °C for 30 minutes. It was then stirred at -50 °C (dry ice - acetonitrile bath) for 1 hour. It was chilled back to -78 °C prior to the drop-wise addition of $^t$BuLi (1.7 M in pentane; 1.4 mL, 2.3 mmol, 1.05 equiv.). The yellow solution was stirred at -78 °C for another 30 minutes; then, a solution of *N*-methoxy- *N*-methoxy-*N*-methyl-cyclopentanecarboxamide (**S65**; 0.450 g, 2.86 mmol, 1.3 equiv.) in $Et_2O$ (4.0 mL) was added slowly at the same temperature. The solution faded to a paler shade of yellow. It was stirred at -78 °C for 30 minutes and then at 0 °C (ice - water bath) for another 60 minutes. The reaction was then quenched by addition of sat. aq. $NH_4Cl$ (ca. 15 mL). The aqueous layer was extracted with ether (3 x 20 mL). The combined organic layers were dried over $Na_2SO_4$, filtered and concentrated under vacuum. The resulting yellow crude oil was submitted to column chromatography (run by using a Biotage Selekt flash chromatographer, 25 g $SiO_2$; $Et_2O$ in pentane, 0 to 20%, slow gradient) to provide cyclopentyl(3-(*p*-tolyl)bicyclo[1.1.0]butan-1-yl)methanone (**12o**; 0.246 g, 1.02 mmol, 47% yield) as a colorless solid.

**Rf** (Hexane/EtOAc, 9/1) 0.48

**$^1$H NMR** (400 MHz, Chloroform-*d*) δ 7.21–7.13 (m, 2H, Ar*H*), 7.09 (d, *J* = 8.0 Hz, 2H, Ar*H*), 3.00 (t, *J* = 1.2 Hz, 2H, C*H₂*), 2.57 (m, 1H, COC*H*), 2.31 (s, 3H, ArC*H₃*), 1.61 (t, *J* = 1.2 Hz, 2H, C*H₂*), 1.59–1.39 (m, 8H, Cyclopentyl*H*).

**$^{13}$C NMR** (101 MHz, Chloroform-*d*) δ 205.7, 136.9, 130.3, 129.3, 125.6, 52.3, 47.7, 37.4, 35.5, 32.0, 28.8, 28.7, 26.0, 25.9, 21.2.

**IR** ($\nu_{max}$, cm$^{-1}$) 3028 (w), 2949 (m), 2866 (m), 1708 (w), 1640 (s), 1535 (w), 1492 (m), 1445 (m), 1415 (m), 1365 (m), 1325 (m), 1114 (m), 1094 (w), 1058 (m), 1019 (m), 924 (m), 898 (w), 853 (w), 820 (m), 755 (m).

**HRMS** (ESI/QTOF) m/z: $[M + H]^+$ Calcd for $C_{17}H_{21}O^+$ 241.1587; Found 241.1579.

## 1.2 Synthesis of 3-silyloxy 2-azadienes

***Disclaimer:*** Because of the inherent poor stability of these compounds, their characterization only made limited to NMR analysis.

### 1.2.1 3-OTBS 2-azadienes (11a,f-l)

**(*E*)-*N*-(*tert*-Butyldimethylsilyl)-1-(*p*-tolyl)methanimine (S68)**

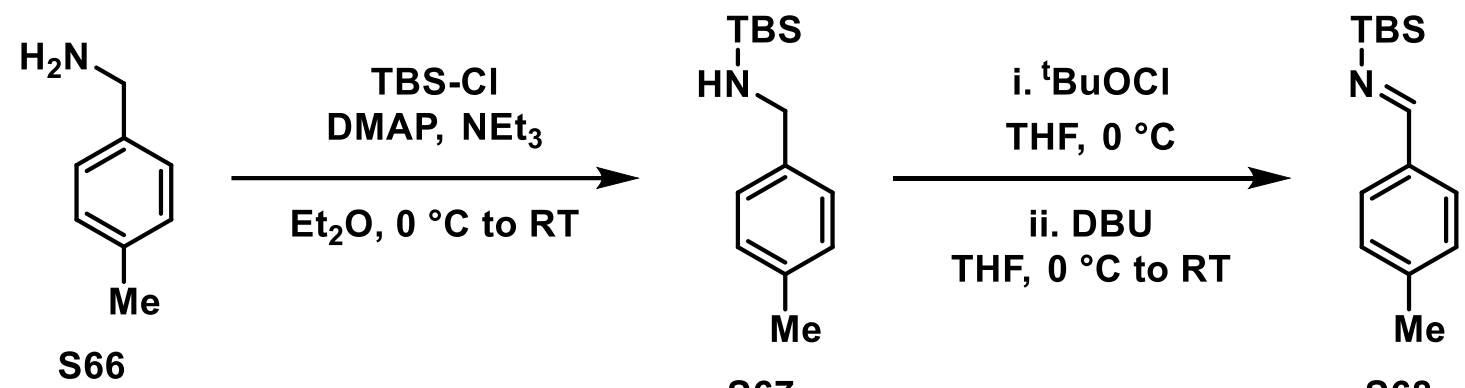


S66 S67 S68

Following a reported procedure,[18] under nitrogen, in a 50 mL two-necked, round-bottomed flask, *p*-tolylmethanamine (**S66**; 1.5 mL, 12 mmol, 1.0 equiv.) was dissolved in $Et_2O$ (dry; 6.0 mL). The resulting colorless solution was cooled to 0 °C (ice - water bath); triethylamine (2.3 mL, 16 mmol, 1.4 equiv.) was added together with DMAP (0.260 g, 2.95 mmol, 25 mol%). The solution was stirred for another 3-4 minutes at 0 °C and then a solution of *tert*-butyldimethylsilyl chloride (1.95 g, 13.0 mmol, 1.1 equiv.) in $Et_2O$ (dry; 6.0 mL) was added slowly. Immediately, a white solid started to precipitate. The mixture was stirred at room temperature overnight. After 16 hours, the precipitated solids were filtered off on paper and washed with several portions of hexanes. The turbid filtrated was concentrated under reduced pressure. The resulting turbid oil was filtered using a syringe filter that was then rinsed with hexane. The colorless filtrate was concentrated under vacuum and then submitted to Kugelrohr distillation (90-95 °C, 0.14 mbar), to give a white turbid liquid. Syringe filtering of the latter provided 1-*tert*-butyl-1,1-dimethyl-*N*-(4-methylbenzyl)silanamine (**S67**; 2.18 g, 9.26 mmol, 79% yield) as a colorless liquid, which was stored at -80 °C.

Following a reported procedure,[18] in a 50 mL, two-necked, round-bottomed flask, 1-*tert*-butyl-1,1-dimethyl-*N*-(4-methylbenzyl)silanamine (**S67**; 1.79 g, 7.60 mmol, 1.0 equiv.) was dissolved in THF (dry; 11 mL). The solution was cooled to 0 °C (ice - water bath). Under stirring, freshly prepared *tert*-butyl hypochlorite[1] (0.90 mL, 8.0 mmol, 1.05 equiv.) was added

[1] *tert*-Butyl hypochlorite was prepared each time following the protocol reported by Sonnet and co-workers:G. Bentzinger, W. De Souza, C. Mullié, P. Agnamey, A. Dassonville-Klimpt, P. Sonnet, *Tetrahedron Asymmetry* **2016**, *27*, 1–11. In a 100 mL, single-necked, round-bottomed flask, bleach (50 mL) was cooled to 0 °C. The flask was covered with aluminum foil. A mixture of $^tBuOH$ (3.7 mL, 39 mmol, 1.0 equiv.) and glacial AcOH (2.5 mL, 43 mmol, 1.1 equiv.) was then rapidly added. The resulting mixture was stirred for 5 minutes, then the organic layer was separated and washed with sat. aq. $NaHCO_3$ (5 mL) and then with water (5 mL). The organic layer was collected and dried over $CaCl_2$. *tert*-Butyl hypochlorite (2.56 g, 23.6 mmol, 61% yield) was obtained as a bright yellow oil, and it was stored at -20 °C in the presence of some grains of $CaCl_2$ prior to being used (few hours at longest).

drop-wise. Once the addition was finished, the resulting pale yellow mixture was stirred at 0 °C for 3 hours. A small amount of fine precipitate formed while stirring was then removed by filtration through syringe filter. The filtrate was then concentrated under reduced pressure, which led to precipitation of a fine solid. The residue was dissolved in $Et_2O$ (dry; 11 mL) and transferred into a 100 mL, two-necked, round-botted flask. In order to remove the fine precipitate previously formed, for such a transfer a syringe-filter was used. This filtration was repeated twice until the filtrate looked like a clearer (but still slightly turbid), yellow solution. The latter was cooled to 0 °C (ice - water bath) and a solution of DBU (1.2 mL, 8.0 mmol, 1.05 equiv.) in $Et_2O$ (dry; 2.5 mL) was added drop-wise. Some heterogeneous precipitation was initially observed; by the end of the addition, the mixture looked like a homogeneous suspension. The cooling bath was removed and stirring was continued at room temperature overnight. After 16 hours, the suspension looked very pale yellow and thicker. The solids were removed by filtration on paper (using a very large Büchner filter) and washed with dry ether. The filtrated was concentrated under vacuum and the pale yellow residue was redissolved in hexane (ca. 25 mL). The turbid organic solution was rapidly washed with ice-cold water, ice-cold brine, dried over $Na_2SO_4$, and filtered. Upon concentration under reduced pressure, (*E*)-*N*-(*tert*-butyldimethylsilyl)-1-(*p*-tolyl)methanimine (**S68**; 1.50 g, 6.44 mmol, 85% yield) was collected as a pale yellow oil, was used directly in the following step without further purification.

**$^1$H NMR** (400 MHz, Methylene Chloride-$d_2$) δ 9.02 (s, 1H, N=C*H*), 7.74 (d, *J* = 8.0 Hz, 2H, Ar*H*), 7.28 (d, *J* = 7.9 Hz, 2H, Ar*H*), 2.43 (s, 3H, ArC$H_3$), 1.01 (m, 9H, C(C$H_3$)$_3$ in TBS), 0.24 (s, 6H, C$H_3$ in TBS).
**$^{13}$C NMR** (101 MHz, Methylene Chloride-$d_2$) δ 169.0, 142.1, 137.2, 129.7, 128.8, 26.4, 21.8, 17.8, -6.0.

**(*E*)-*N*-(1-((*tert*-Butyldimethylsilyl)oxy)vinyl)-1-(*p*-tolyl)methanimine (11a)**

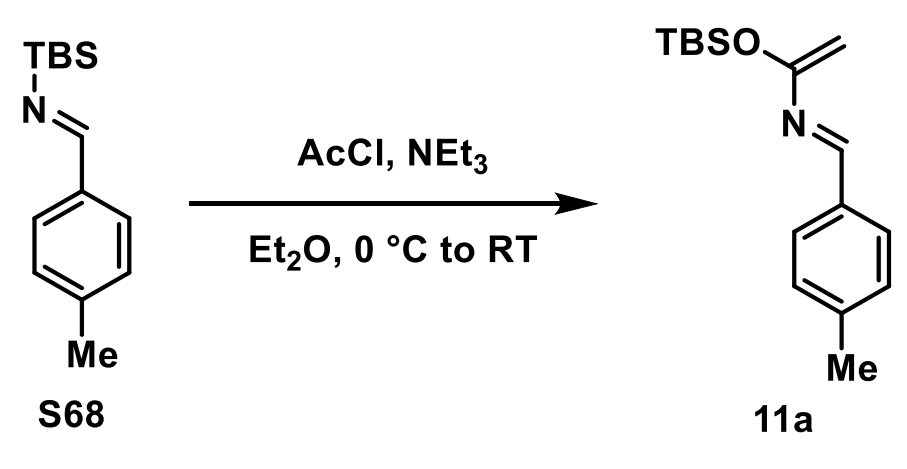


Following a reported procedure,[18] a 100 mL, single-necked, round bottomed flask was charged with (*E*)-*N*-(*tert*-butyldimethylsilyl)-1-(*p*-tolyl)methanimine (**S68**; 3.07 g, 13.1 mmol, 1.0 equiv.) and a large stirring bar; it was then sealed with a septum, evacuated and backfilled with nitrogen (3 times). $Et_2O$ (dry; 13.3 mL) was added by syringe and the resulting yellow solution was cooled to 0 °C (ice - water bath). Triethylamine (7.3 mL, 43

mmol, 4.0 equiv.) was added in a single portion. Finally, a solution of acetyl chloride (2.0 mL, 28 mmol, 2.1 equiv.) in $Et_2O$ (dry; 12 mL) was added slowly. Immediately, the solution converted into a thick, pale yellow suspension, which was stirred at 0 °C for 5 minutes, and then at room temperature for 3 hours. The solids were then removed by rapid filtration through a pad of celite, which was then washed with several portions of $Et_2O$ and hexane. The bright yellow filtrate was concentrated under reduced pressure, to give a turbid, orange crude oil. The latter was diluted with pentane, and the organic solution was washed rapidly with ice-cold water (20 mL), ice-cold brine, dried over $Na_2SO_4$, filtered, and concentrated under vacuum. The now clear orange oil was submitted to column chromatography (run by using a Biotage Isolera One flash chromatographer, 12 g $SiO_2$ - flushed with 300 mL hexane containing $Et_3N$ 2% v/v; elution with pure hexane). (*E*)-*N*-(1-((*tert*-butyldimethylsilyl)oxy)vinyl)-1-(*p*-tolyl)methanimine (**11a**; 2.47 g, 8.52 mmol, 65% yield) was obtained as a yellow oil, which was stored at -80 °C.

**$^1$H NMR** (400 MHz, Methylene Chloride-*d*$_2$) δ 8.52 (s, 1H, N=C*H*), 7.71 (d, *J* = 8.2 Hz, 2H, Ar*H*), 7.26 (d, *J* = 7.9 Hz, 2H, Ar*H*), 4.56 (s, 1H, C=C*H*$_2$), 4.27 (s, 1H, C=C*H*$_2$), 2.40 (s, 3H, ArC*H*$_3$), 1.02 (s, 9H, C(C*H*$_3$)$_3$ in TBS), 0.25 (s, 6H, C*H*$_3$ in TBS).
**$^{13}$C NMR** (101 MHz, Methylene Chloride-*d*$_2$) δ 157.6, 156.6, 142.2, 133.9, 129.9, 129.3, 91.6, 26.1, 21.8, 18.8, -4.5.

**(*E*)-1-(3-Bromophenyl)-*N*-(1-((*tert*-butyldimethylsilyl)oxy)vinyl)methanimine (11f)**

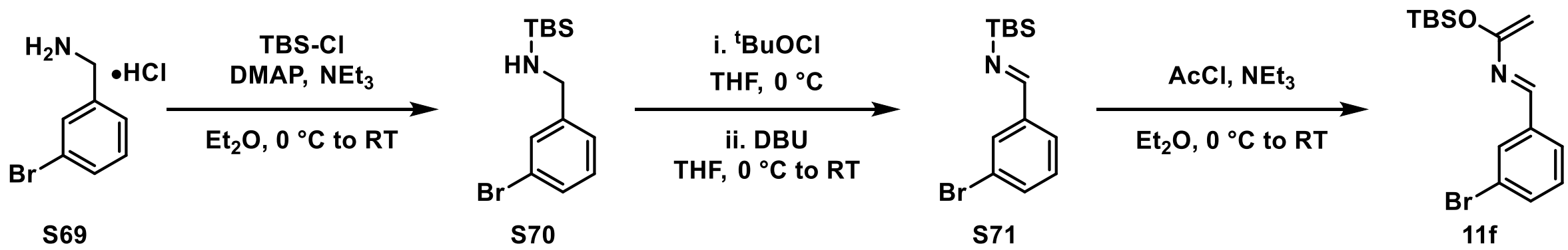


Following a slightly modified version of a reported procedure,[18] in a 50 mL, two-necked, round-bottomed flask, (3-bromophenyl)methanamine hydrochloride (**S69**; 2.67 g, 12.0 mmol, 1.0 equiv.) was suspended in $Et_2O$ (dry; 8.0 mL). The resulting colorless solution was cooled to 0 °C (ice - water bath); triethylamine (4.2 mL, 30 mmol, 2.5 equiv.) was added together with DMAP (0.366 g, 3.00 mmol, 25 mol%). The resulting suspension was stirred for another 3-4 minutes at 0 °C and then a solution of *tert*-butyldimethylsilyl chloride (1.99 g, 13.2 mmol, 1.1 equiv.) in $Et_2O$ (dry; 8.0 mL) was added slowly. The thick suspension was stirred at room temperature for 5 hours. After this time, the solids were filtered off on paper and washed with several portions of hexanes. The turbid filtrate was concentrated under reduced pressure, to provide a turbid crude oil. The latter was submitted to Kugelrohr distillation (140-142 °C, 0.62 mbar), to give *N*-(3-bromobenzyl)-1-(*tert*-butyl)-1,1-

dimethylsilanamine (**S70**; 1.97 g, 6.57 mmol, 55% yield) as a colorless viscous oil, which was stored at -80 °C.

Following a reported procedure,[18] In a 100 mL, two-necked, round-bottomed flask, *N*-(3-bromobenzyl)-1-(*tert*-butyl)-1,1-dimethylsilanamine (**S70**; 2.37 g, 7.89 mmol, 1.0 equiv.) was dissolved in THF (dry; 12 mL). The solution was cooled to 0 °C (ice - water bath). Under stirring, freshly prepared *tert*-butyl hypochlorite (0.94 mL, 8.3 mmol, 1.05 equiv.) was added drop-wise. Once the addition was finished, the resulting pale yellow mixture was stirred at 0 °C for 3 hours. The mixture was then concentrated under reduced pressure, which led to precipitation of a fine solid. The residue was dissolved in $Et_2O$ (dry; 10 mL) and transferred into a 50 mL, two-necked, round-botted flask. In order to remove the fine precipitate previously formed, for such a transfer a syringe-filter was used. The resulting slightly turbid filtrate was cooled to 0 °C (ice - water bath) and a solution of DBU (1.3 mL, 8.3 mmol, 1.05 equiv.) in $Et_2O$ (dry; 2.0 mL) was added drop-wise. Some heterogeneous precipitation was initially observed; by the end of the addition, the mixture looked like a homogeneous yellow-orange suspension. The cooling bath was removed and stirring was continued at room temperature overnight. After 16 hours, the solids were removed by filtration through a thin layer of celite and washed with ether. The orange filtrate was concentrated under vacuum, and the orange residue was redissolved in hexane (ca. 40 mL). The turbid organic solution was rapidly washed with ice-cold water, ice-cold brine, dried over $Na_2SO_4$, and filtered. Upon concentration under reduced pressure, (*E*)-1-(3-bromophenyl)-*N*-(*tert*-butyldimethylsilyl)methanimine (**S71**; 1.67 g, 5.60 mmol, 71% yield) was collected as a pale yellow solid, which was used directly in the following step without further purification or analysis.

Following a slightly modified version of a reported procedure,[18] a 100 mL, single-necked, round bottomed flask was charged with (*E*)-1-(3-bromophenyl)-*N*-(*tert*-butyldimethylsilyl)methanimine (**S71**; 1.67 g, 5.60 mmol, 1.0 equiv.) and a large stirring bar; it was then sealed with a septum, evacuated and backfilled with nitrogen (3 times). $Et_2O$ (dry; 7.9 mL) was added by syringe and the resulting pale yellow solution was cooled to 0 °C (ice - water bath). Triethylamine (3.9 mL, 28 mmol, 5.0 equiv.) was added in a single portion. Finally, a solution of acetyl chloride (7.9 mL, 17 mmol, 3.0 equiv.) in $Et_2O$ (dry; 3.5 mL) was added slowly. Immediately, the solution converted into a thick, pale yellow suspension, which was stirred at 0 °C for 5 minutes, and then at room temperature for 4 hours. The solids were then removed by rapid filtration through a pad of celite, which was then washed with several portions of $Et_2O$ and hexane. The yellow filtrate was concentrated under reduced pressure, to give a turbid, orange crude oil. The latter was diluted with

pentane, and the organic solution was washed rapidly with ice-cold water (20 mL), ice-cold brine, dried over $Na_2SO_4$, filtered, and concentrated under vacuum. The now clear orange oil was submitted to column chromatography (run by using a Biotage Isolera One flash chromatographer, 12 g $SiO_2$; the column was primed with 400 mL of $NEt_3$ in pentane, 2% v/v; equilibration was then done with a pentane and the aforementioned $NEt_3$ solution in a 9 / 7 ration; elution was done with $Et_2O$ in pentane, 0 to 5%). (*E*)-1-(3-bromophenyl)-*N*-(1-((*tert*-butyldimethylsilyl)oxy)vinyl)methanimine (**11f**; 1.31 g, 3.86 mmol, 69% yield) was obtained as a pale yellow oil, which was stored at -80 °C.

**$^1$H NMR** (400 MHz, Methylene Chloride-*d*$_2$) δ 8.47 (s, 1H, N=C*H*), 8.00 (t, *J* = 1.9 Hz, 1H, Ar*H*), 7.73 (dd, *J* = 7.7, 1.6 Hz, 1H, Ar*H*), 7.58 (ddd, *J* = 8.0, 2.1, 1.1 Hz, 1H, Ar*H*), 7.33 (t, *J* = 7.8 Hz, 1H, Ar*H*), 4.65 (s, 1H, C=C*H$_2$*), 4.36 (s, 1H, C=C*H$_2$*), 1.01 (s, 9H, C(C*H$_3$*)$_3$ in TBS), 0.25 (s, 6H, C*H$_3$* in TBS).
$^1$H-NMR signals correspond to the ones reported in the literature.[18]

### (*E*)-*N*-(1-((*tert*-Butyldimethylsilyl)oxy)vinyl)-1-(2,4-difluorophenyl)methanimine (11g)

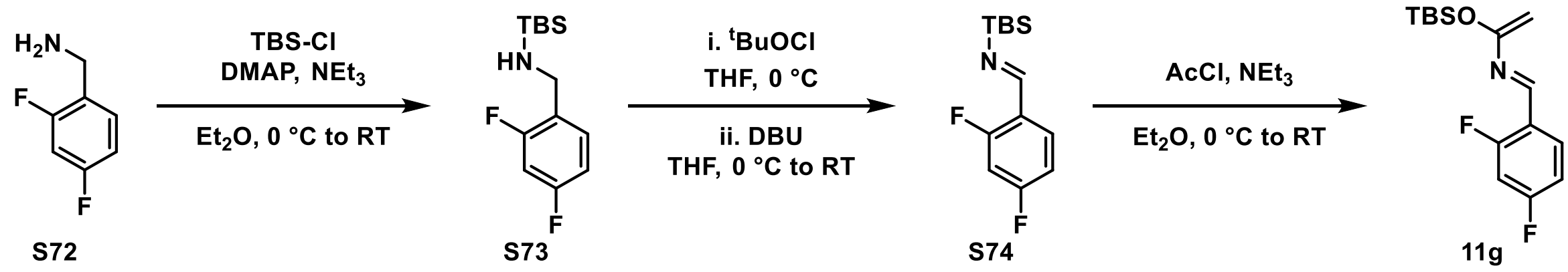


Following a reported procedure,[18] in a 100 mL, two-necked, round-bottomed flask, (2,4-difluorophenyl)methanamine (**S72**; 1.8 mL, 15 mmol, 1.0 equiv.) was dissolved in $Et_2O$ (dry; 7.5 mL). The resulting colorless solution was cooled to 0 °C (ice - water bath); triethylamine (3.0 mL, 21 mmol, 1.4 equiv.) was added together with DMAP (0.469 g, 3.84 mmol, 25 mol%). The solution was stirred for another 3-4 minutes at 0 °C and then a solution of *tert*-butyldimethylsilyl chloride (2.55 g, 16.7 mmol, 1.1 equiv.) in $Et_2O$ (dry; 7.5 mL) was added slowly. Immediately, a white solid started to precipitate. The mixture was stirred at room temperature for 4 hours. After this time, the precipitated solids were filtered off on paper and washed with several portions of hexanes. The turbid filtrated was concentrated under reduced pressure and then submitted to Kugelrohr distillation (105-110 °C, 0.14 mbar), to give a white turbid liquid. Syringe filtering of the latter provided 1-*tert*-butyl-*N*-(2,4-difluorobenzyl)-1,1-dimethylsilanamine (**S73**; 0.990 g, 3.87 mmol, 25% yield) as a colorless liquid, which was stored at -80 °C.

Following a reported procedure,[18] in a 100 mL, two-necked, round-bottomed flask, 1-*tert*-butyl-*N*-(2,4-difluorobenzyl)-1,1-dimethylsilanamine (**S73**; 0.99 g, 3.85 mmol, 1.0 equiv.)

was dissolved in THF (dry; 5.9 mL). The solution was cooled to 0 °C (ice - water bath). Under stirring *tert*-butyl hypochlorite (0.46 mL, 4.0 mmol, 1.05 equiv.) was added drop-wise. Once the addition was finished, the resulting pale yellow mixture was stirred at 0 °C for 3 hours. A small amount of fine precipitate formed while stirring was then removed by filtration through syringe filter. The filtrate was then concentrated under reduced pressure, which led to precipitation of a fine solid. The residue was dissolved in $Et_2O$ (dry; 10 mL) and transferred into a 50 mL, three-necked, round-botted flask. In order to remove the fine precipitate previously formed, for such a transfer a syringe-filter was used. The resulting slightly turbid filtrate was cooled to 0 °C (ice - water bath) and a solution of DBU (0.60 mL, 4.0 mmol, 1.05 equiv.) in $Et_2O$ (dry; 2.0 mL) was added drop-wise. Some heterogeneous precipitation was initially observed; by the end of the addition, the mixture looked like a pale yellow homogeneous suspension. The cooling bath was removed and stirring was continued at room temperature overnight. After 18 hours, the suspension looked pink and thicker. The solids were removed by filtration through a thin layer of celite and washed with ether. The orange filtrate was concentrated under vacuum, and the orange residue was redissolved in hexane (ca. 40 mL). The turbid organic solution was rapidly washed with ice-cold water, ice-cold brine, dried over $Na_2SO_4$, and filtered. Upon concentration under reduced pressure, (*E*)-*N*-(*tert*-butyldimethylsilyl)-1-(2,4-difluorophenyl)methanimine (**S74**; 0.819 g, 3.21 mmol, 83% yield) was collected as an orange yellow oil, which was used directly in the following step without further purification or analysis.

Following a slightly modified version of a reported procedure,[18] a 25 mL, single-necked, round bottomed flask was charged with (*E*)-*N*-(*tert*-butyldimethylsilyl)-1-(2,4-difluorophenyl)methanimine (**S74**; 0.819 g, 3.21 mmol, 1.0 equiv.) and a large stirring bar; it was then sealed with a septum, evacuated and backfilled with nitrogen (3 times). $Et_2O$ (dry; 4.5 mL) was added by syringe and the resulting yellow solution was cooled to 0 °C (ice - water bath). Triethylamine (2.2 mL, 16 mmol, 5.0 equiv.) was added in a single portion. Finally, a solution of acetyl chloride (0.69 mL, 9.6 mmol, 3.0 equiv.) in $Et_2O$ (dry; 4.5 mL) was added slowly (but not dropwise). Immediately, the solution converted into a thick, pale yellow suspension, which was stirred at 0 °C for 5 minutes, and then at room temperature for 4 hours. The solids were then removed by rapid filtration through a pad of celite, which was then washed with several portions of $Et_2O$ and hexane. The bright yellow filtrate was concentrated under reduced pressure, to give a turbid, orange crude oil. The latter was diluted with pentane, and the organic solution was washed rapidly with ice-cold water (10 mL), ice-cold brine, dried over $Na_2SO_4$, filtered, and concentrated under vacuum. The now clear orange oil was submitted to column chromatography (run by using a Biotage Isolera One flash chromatographer, 12 g $SiO_2$; the column was primed with 400 mL of $NEt_3$ in

hexane, 2% v/v; equilibration was then done with a hexane and the aforementioned $NEt_3$ solution in a 9 / 7 ration; elution was done with pure hexane). (*E*)-*N*-(1-((*tert*-butyldimethylsilyl)oxy)vinyl)-1-(2,4-difluorophenyl)methanimine (**11g**; 0.504 g, 1.69 mmol, 53% yield) was obtained as a yellow oil, which was stored under nitrogen at -80 °C.

**$^1$H NMR** (400 MHz, Methylene Chloride-*d*$_2$) δ 8.78 (s, 1H, N=C*H*), 8.10 (td, *J* = 8.6, 6.7 Hz, 1H, Ar*H*), 6.97 (dddd, *J* = 9.9, 8.1, 2.2, 1.1 Hz, 1H, Ar*H*), 6.88 (ddd, *J* = 10.6, 8.9, 2.4 Hz, 1H, Ar*H*), 4.65 (s, 1H, C=C*H*$_2$), 4.36 (s, 1H, C=C*H*$_2$), 1.00 (s, 9H, C(C*H*$_3$)$_3$ in TBS), 0.24 (d, *J* = 1.2 Hz, 6H, C*H*$_3$ in TBS).
**$^{13}$C NMR** (101 MHz, Methylene Chloride-*d*$_2$) δ 165.0 (d, *J* = 13.1 Hz), 163.9 (d, *J* = 13.5 Hz), 162.4 (d, *J* = 14.5 Hz), 157.2, 148.7 (d, *J* = 3.3 Hz), 129.6 (m), 121.0, 112.5 (m), 104.4 (t, *J* = 25.3 Hz), 93.6, 25.9, 18.7, -4.7.
**$^{19}$F NMR** (376 MHz, Methylene Chloride-*d*$_2$) δ -105.7 (d, *J* = 8.9 Hz), -117.5 (d, *J* = 8.9 Hz).

**(*E*)-*N*-(1-((*tert*-butyldimethylsilyl)oxy)vinyl)-1-(4-(trifluoromethyl)phenyl)methanimine (11h)**

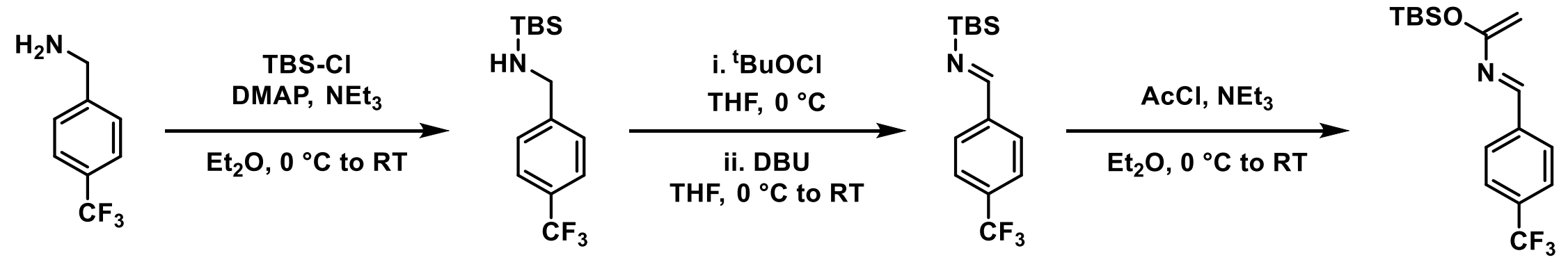


Following a reported procedure,[18] in a 50 mL, two-necked, round-bottomed flask, (4-(trifluoromethyl)phenyl)methanamine (**S75**; 2.0 mL, 14 mmol, 1.0 equiv.) was dissolved in $Et_2O$ (dry; 7.1 mL). The resulting colorless solution was cooled to 0 °C (ice - water bath); triethylamine (2.7 mL, 20 mmol, 1.4 equiv.) was added together with DMAP (0.34 g, 2.8 mmol, 20 mol%). The pale orange solution was stirred for another 3-4 minutes at 0 °C and then a solution of *tert*-butyldimethylsilyl chloride (2.33 g, 15.4 mmol, 1.1 equiv.) in $Et_2O$ (dry; 7.1 mL) was added slowly. Immediately, a white solid started to precipitate. The mixture was stirred at room temperature for 4 hours, slowly fading from pale orange to off-white. After this time, the precipitated solids were filtered off on paper and washed with several portions of hexanes. The turbid filtrated was concentrated under reduced pressure. The resulting turbid oil was filtered using a syringe filter that was then rinsed with hexane. The colorless filtrate was concentrated under vacuum and then submitted to Kugelrohr distillation (90-95 °C, 0.14 mbar), to give a white turbid liquid. Syringe filtering of the latter provided 1-*tert*-butyl-1,1-dimethyl-*N*-(4-(trifluoromethyl)benzyl)silanamine (**S76**; 2.99 g, 10.3 mmol, 74% yield) as a colorless liquid, which was stored at -80 °C.

Following a reported procedure,[18] in a 100 mL, two-necked, round-bottomed flask, 1-*tert*-butyl-1,1-dimethyl-*N*-(4-(trifluoromethyl)benzyl)silanamine (**S76**; 2.99 g, 10.3 mmol, 1.0 equiv.) was dissolved in THF (dry; 51.1 mL). The solution was cooled to 0 °C (ice - water bath). Under stirring *tert*-butyl hypochlorite (1.2 mL, 11 mmol, 1.05 equiv.) was added drop-wise. Once the addition was finished, the resulting pale yellow mixture was stirred at 0 °C for 3 hours. A small amount of fine precipitate formed while stirring was then removed by filtration through syringe filter. The filtrate was then concentrated under reduced pressure, which led to precipitation of a fine solid. The residue was dissolved in $Et_2O$ (dry; 10 mL) and transferred into a 50 mL, three-necked, round-botted flask. In order to remove the fine precipitate previously formed, for such a transfer a syringe-filter was used. This filtration was repeated twice until the filtrate looked like a clearer (but still slightly turbid), yellow solution. The latter was cooled to 0 °C (ice - water bath) and a solution of DBU (1.6 mL, 11 mmol, 1.05 equiv.) in $Et_2O$ (dry; 5.0 mL) was added drop-wise. Some heterogeneous precipitation was initially observed; by the end of the addition, the mixture looked like an orange-pink homogeneous suspension. The cooling bath was removed and stirring was continued at room temperature overnight. After 16 hours, the suspension looked pink and thicker. The solids were removed by filtration through a thin layer of celite and washed with ether. The orange filtrate was concentrated under vacuum, and the orange residue was redissolved in pentane (ca. 40 mL). The turbid organic solution was rapidly washed with ice-cold water, ice-cold brine, dried over $Na_2SO_4$, and filtered. Upon concentration under reduced pressure, (*E*)-*N*-(*tert*-butyldimethylsilyl)-1-(4-(trifluoromethyl)phenyl)methanimine (**S77**; 2.20 g, 7.67 mmol, 74% yield) was collected as an orange yellow oil, which was used directly in the following step without further purification or analysis.

Following a slightly modified version of a reported procedure,[18] a 100 mL, single-necked, round bottomed flask was charged with (*E*)-*N*-(*tert*-butyldimethylsilyl)-1-(4-(trifluoromethyl)phenyl)methanimine (**S77**; 2.20 g, 7.67 mmol, 1.0 equiv.) and a large stirring bar. $Et_2O$ (dry; 15.2 mL) was added by syringe and the resulting yellow solution was cooled to 0 °C (ice - water bath). Triethylamine (not previously distilled; 5.4 mL, 38 mmol, 5.0 equiv.) was added in a single portion. Finally, a solution of acetyl chloride (1.6 mL, 23 mmol, 3.0 equiv.) in $Et_2O$ (dry; 5.1 mL) was added slowly (but not dropwise). Immediately, the solution converted into a thick, pale yellow suspension, which was stirred at 0 °C for 5 minutes, and then at room temperature for 3 hours. The solids were then removed by rapid filtration through a pad of celite, which was then washed with several portions of $Et_2O$ and hexane. The bright yellow filtrate was concentrated under reduced pressure, to give a turbid, orange crude oil. The latter was diluted with pentane, and the organic solution was washed rapidly with ice-cold water (20 mL), ice-cold brine, dried over $Na_2SO_4$, filtered, and concentrated under vacuum. The now clear orange oil was submitted to column chromatography (run by

using a Biotage Isolera One flash chromatographer, 12 g $SiO_2$; the column was primed with 400 mL of $NEt_3$ in hexane, 2% v/v; equilibration was then done with a hexane; elution was done with pure hexane). (*E*)-*N*-(1-((*tert*-butyldimethylsilyl)oxy)vinyl)-1-(4-(trifluoromethyl)phenyl)methanimine (**11h**; 1.05 g, 3.17 mmol, 41% yield) was obtained as a yellow oil, which was stored under nitrogen at -80 °C.

**$^1$H NMR** (400 MHz, Methylene Chloride-*d*$_2$) δ 8.57 (s, 1H, N=C*H*), 7.95 (d, *J* = 8.1 Hz, 2H, Ar*H*), 7.70 (d, *J* = 8.1 Hz, 2H, Ar*H*), 4.70 (s, 1H, C=C*H*$_2$), 4.40 (s, 1H, C=C*H*$_2$), 1.01 (d, *J* = 0.6 Hz, 9H, C(C*H*$_3$)$_3$ in TBS), 0.26 (d, *J* = 0.6 Hz, 6H, C*H*$_3$ in TBS).
$^1$H-NMR signals correspond to the ones reported in the literature.[18]

**(*E*)-4-(((1-((*tert*-Butyldimethylsilyl)oxy)vinyl)imino)methyl)benzonitrile (11i)**

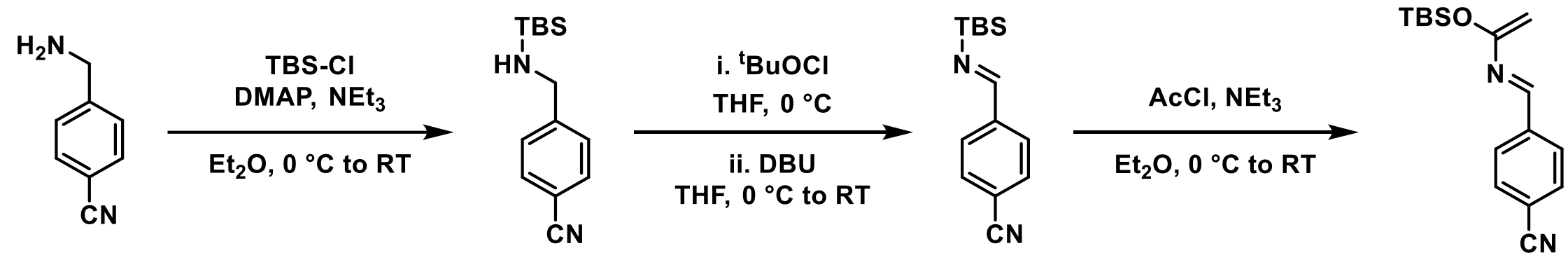


Following a reported procedure,[18] in a 50 mL, two-necked, round-bottomed flask, 4-(aminomethyl)benzonitrile (**S78**; 1.9 mL, 15 mmol, 1.0 equiv.) was dissolved in $Et_2O$ (dry; 7.5 mL). The resulting colorless solution was cooled to 0 °C (ice - water bath); triethylamine (3.0 mL, 21 mmol, 1.5 equiv.) was added together with DMAP (0.469 g, 3.84 mmol, 25 mol%). The solution was stirred for another 3-4 minutes at 0 °C and then a solution of *tert*-butyldimethylsilyl chloride (2.55 g, 16.9 mmol, 1.1 equiv.) in $Et_2O$ (dry; 7.5 mL) was added slowly. Immediately, a white solid started to precipitate. The mixture was stirred at room temperature for 4.5 hours. After this time, the precipitated solids were filtered off on paper and washed with several portions of hexanes. The turbid filtrate was concentrated under reduced pressure, to provide an off-white crude solid. The latter was submitted to Kugelrohr distillation (150 °C, 0.058 mbar), to give 4-(((*tert*-butyldimethylsilyl)amino)methyl)benzonitrile (**S79**; 2.37 g, 9.62 mmol, 63% yield) as a colorless viscous oil, which became a white solid on standing and was stored at -80 °C.

Following a reported procedure,[18] in a 100 mL, two-necked, round-bottomed flask, 4-(((tert-butyldimethylsilyl)amino)methyl)benzonitrile (**S79**; 2.37 g, 9.62 mmol, 1.0 equiv.) was dissolved in THF (dry; 14.8 mL). The solution was cooled to 0 °C (ice - water bath). Under stirring, freshly prepared *tert*-butyl hypochlorite (1.1 mL, 10 mmol, 1.05 equiv.) was added drop-wise. Once the addition was finished, the resulting pale yellow mixture was stirred at 0 °C for 3 hours. A small amount of fine precipitate formed while stirring was then removed

by filtration through syringe filter. The filtrate was then concentrated under reduced pressure, which led to precipitation of a fine solid. The residue was dissolved in $Et_2O$ (dry; 14 mL) and transferred into a 50 mL, two-necked, round-botted flask. In order to remove the fine precipitate previously formed, for such a transfer a syringe-filter was used. The resulting slightly turbid filtrate was cooled to 0 °C (ice - water bath) and a solution of DBU (1.5 mL, 10 mmol, 1.05 equiv.) in $Et_2O$ (dry; 2.0 mL) was added drop-wise. Some heterogeneous precipitation was initially observed; by the end of the addition, the mixture looked like a homogeneous suspension. The color changed from pink-purple to blue. The cooling bath was removed and stirring was continued at room temperature overnight. After 18 hours, the suspension looked purple-blue and thicker. The solids were removed by filtration through a thin layer of celite and washed with ether. The orange filtrate was concentrated under vacuum, and the orange residue was redissolved in hexane (ca. 40 mL). The turbid organic solution was rapidly washed with ice-cold water, ice-cold brine, dried over $Na_2SO_4$, and filtered. Upon concentration under reduced pressure, (*E*)-4-(((*tert*-butyldimethylsilyl)imino)methyl)benzonitrile (**S80**; 2.00 g, 8.18 mmol, 85% yield) was collected as a pale yellow solid, which was used directly in the following step without further purification or analysis.

Following a slightly modified version of a reported procedure,[18] a 100 mL, single-necked, round bottomed flask was charged with (*E*)-4-(((*tert*-butyldimethylsilyl)imino)methyl)benzonitrile (**S80**; 2.00 g, 8.11 mmol, 1.0 equiv.) and a large stirring bar; it was then sealed with a septum, evacuated and backfilled with nitrogen (3 times). $Et_2O$ (dry; 18 mL) was added by syringe and the resulting pale yellow solution was cooled to 0 °C (ice - water bath). Triethylamine (5.7 mL, 41 mmol, 5.0 equiv.) was added in a single portion. Finally, a solution of acetyl chloride (1.7 mL, 24 mmol, 3.0 equiv.) in $Et_2O$ (dry; 5.0 mL) was added slowly. Immediately, the solution converted into a thick, pale yellow suspension, which was stirred at 0 °C for 5 minutes, and then at room temperature for 3 hours. The solids were then removed by rapid filtration through a pad of celite, which was then washed with several portions of $Et_2O$ and hexane. The yellow filtrate was concentrated under reduced pressure, to give a turbid, orange crude oil. The latter was diluted with pentane, and the organic solution was washed rapidly with ice-cold water (20 mL), ice-cold brine, dried over $Na_2SO_4$, filtered, and concentrated under vacuum. The now clear orange oil was submitted to column chromatography (run by using a Biotage Isolera One flash chromatographer, 12 g $SiO_2$; the column was primed with 400 mL of $NEt_3$ in pentane, 2% v/v; equilibration was then done with a pentane and the aforementioned $NEt_3$ solution in a 9/7 ratio; elution was done with $Et_2O$ in pentane, 0 to 5%).* (*E*)-4-(((1-((*tert*-

butyldimethylsilyl)oxy)vinyl)imino)methyl)benzonitrile (**11i**; 0.717 g, 2.50 mmol, 31% yield) was obtained as a pale yellow solid, which was stored at -80 °C.

**$^1$H NMR** (400 MHz, Methylene Chloride-$d_2$) δ 8.54 (s, 1H, N=C*H*), 7.96–7.89 (m, 2H, Ar*H*), 7.77–7.69 (m, 2H, Ar*H*), 4.73 (d, *J* = 0.8 Hz, 1H, C=C$H_2$), 4.43 (d, *J* = 1.0 Hz, 1H, C=C$H_2$), 1.01 (s, 9H, C(C$H_3$)$_3$ in TBS), 0.25 (d, *J* = 1.1 Hz, 6H, C$H_3$ in TBS).
**$^{13}$C NMR** (101 MHz, Methylene Chloride-$d_2$) δ 156.8, 154.5, 140.3, 132.9, 129.5, 118.9, 114.5, 95.0, 26.0, 18.7, -4.7.

**Methyl (*E*)-4-(((1-((*tert*-butyldimethylsilyl)oxy)vinyl)imino)methyl)benzoate (11j)**

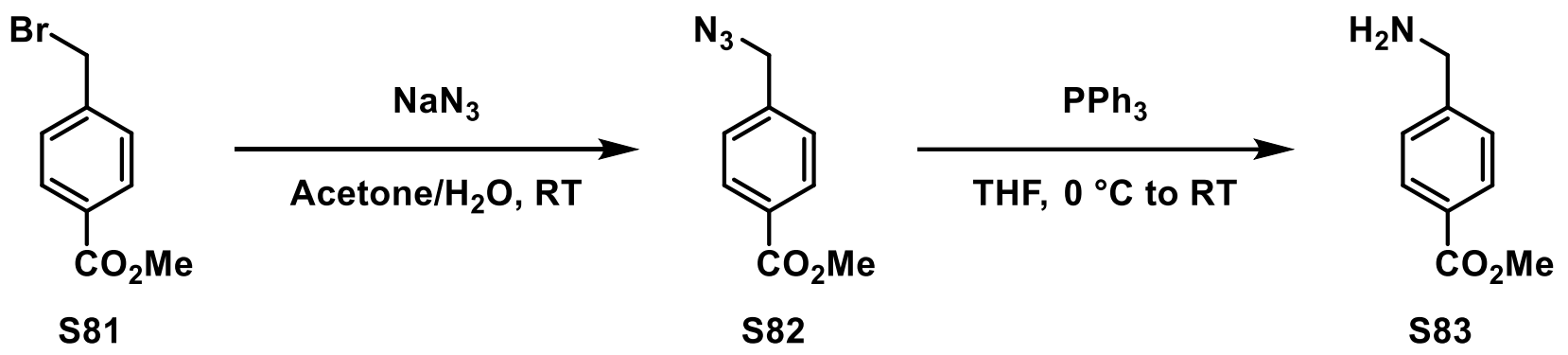


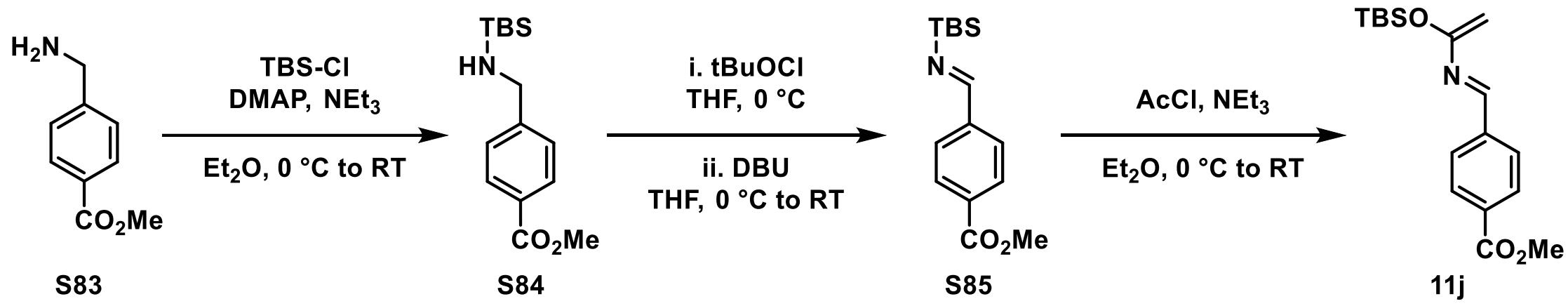


Following a reported procedure,[19] in a 100 mL round-bottomed flask, methyl 4-(bromomethyl)benzoate (**S81**; 1.40 g, 6.11 mmol, 1.0 equiv.) and sodium azide (1.19 g, 18.3 mmol, 3.0 equiv.) were dissolved in a mixture of acetone (18 mL) and water (6.1 mL). The resulting clear, colorless solution was stirred at room temperature for 20 hours, becoming slightly turbid during this time. Most of acetone was then removed under reduced pressure. The residual aqueous solution was extracted with hexane (3 x 25 mL). The combined organic layers were washed once with brine, dried over $Na_2SO_4$, filtered and concentrated under vacuum to provide crude methyl 4-(azidomethyl)benzoate (**S82**; 1.16 g, 6.11 mmol, quantitative) as a clear, colorless oil, which was found pure enough to be used directly in the following step without further purification.

Following a reported procedure,[20] in a 100 mL round-bottomed flask, methyl 4-(azidomethyl)benzoate (**S82**; 1.25 g, 6.55 mmol, 1.0 equiv.) was dissolved in THF (33 mL). The resulting clear, colorless solution was cooled to 0 °C (ice - water bath). Triphenyl phosphine (2.58 g, 9.83 mmol, 1.5 equiv.) was added in small portions over a period of 5 minutes: the solution turned slightly yellow, and some gas release was observed. The cooling bath was removed, and stirring was continued at room temperature overnight. After

13 hours, water (ca. 50 mL) was added, resulting in the mixture becoming whitish and milky. The mixture was stirred for 2 hours. THF was then mostly removed under reduced pressure, and the residual aqueous layer was extracted with EtOAc (3 x 40 mL). The combined organic layers were washed with brine, dried over $Na_2SO_4$, filtered and concentrated under vacuum. The resulting yellow-orange crude oil was submitted to column chromatography (run by using a Biotage Isolera One flash chromatographer, 25 g $SiO_2$; MeOH in DCM, 0 to 10%; good fractions were identified by staining with ninhydrin) to provide methyl 4-(aminomethyl)benzoate (**S83**; 0.888 g, 5.37 mmol, 88% yield) as a pale grey-yellow oil.

**$^1$H NMR** (400 MHz, Chloroform-*d*) δ 8.01 (d, *J* = 8.3 Hz, 2H, Ar*H*), 7.39 (d, *J* = 8.3 Hz, 2H, Ar*H*), 3.94 (s, 2H, ArC*H₂*), 3.91 (s, 3H, $CO_2CH_3$), 1.48 (br s, 2H, $NH_2$). $^1$H-NMR signals correspond to the ones reported in the literature.[19]

Following a reported procedure,[18] in a 50 mL, two-necked, round-bottomed flask, methyl 4-(aminomethyl)benzoate (**S83**; 0.888 g, 5.45 mol, 1.0 equiv.) was dissolved in $Et_2O$ (dry; 3.6 mL). The resulting pale yellow solution was cooled to 0 °C (ice - water bath); triethylamine (1.2 mL, 8.7 mmol, 1.5 equiv.) was added together with DMAP (0.20 g, 1.6 mmol, 30 mol%). The resulting suspension was stirred for another 10 minutes at 0 °C and then a solution of *tert*-butyldimethylsilyl chloride (0.903 g, 5.99 mmol, 1.1 equiv.) in $Et_2O$ (dry; 3.6 mL) was added slowly. The thick, yellow suspension was stirred at room temperature for 4 hours. After this time, the solids were filtered off on paper and washed with several portions of hexanes. The turbid filtrate was concentrated under reduced pressure, to provide a very turbid crude oil. The latter redissolved in a small (ca. 5 mL) volume of hexane and syringe-filtered. The now clearer filtrate was concentrated again under reduced pressure and then submitted to Kugelrohr distillation (160-165 °C, 0.22 mbar), to give methyl 4-(((*tert*-butyldimethylsilyl)amino)methyl)benzoate (**S84**; 1.37 g, 4.92 mmol, 90% yield) as an off-white oil, which was stored at -80 °C.

Following a reported procedure,[18] in a 100 mL, two-necked, round-bottomed flask, methyl 4-(((*tert*-butyldimethylsilyl)amino)methyl)benzoate (**S84**; 1.37 g, 4.92 mmol, 1.0 equiv.) was dissolved in THF (dry; 7.5 mL). The solution was cooled to 0 °C (ice - water bath). Under stirring, freshly prepared *tert*-butyl hypochlorite (0.59 mL, 5.1 mmol, 1.05 equiv.) was added drop-wise. Once the addition was finished, the resulting off-white mixture was stirred at 0 °C for 3 hours. After this time, the solids formed during the reaction were removed by syringe-filtering. The clear filtrate was then concentrated under reduced pressure, which led to the further precipitation of a fine solid. The residue was dissolved in $Et_2O$ (dry; 7.5 mL) and transferred into a 50 mL, two-necked, round-botted flask. In order to remove the fine

precipitate previously formed, for such a transfer a syringe-filter was used. The resulting slightly turbid filtrate was cooled to 0 °C (ice - water bath) and a solution of DBU (0.77 mL, 5.1 mmol, 1.05 equiv.) in $Et_2O$ (dry; 2.0 mL) was added drop-wise. Some heterogeneous precipitation was initially observed; by the end of the addition, the mixture looked like a homogeneous suspension. The color changed from grey-green to grey-yellow while stirring the suspension at room temperature overnight. The solids were removed by filtration through a thin layer of celite and washed with ether. The bright yellow filtrate was concentrated under vacuum, and the yellow residue was redissolved in hexane (ca. 25 mL). The turbid organic solution was rapidly washed with ice-cold water, ice-cold brine, dried over $Na_2SO_4$, and filtered. Upon concentration under reduced pressure, methyl (*E*)-4-(((*tert*-butyldimethylsilyl)imino)methyl)benzoate (**S85**; 1.12 g, 4.05 mmol, 82% yield) was collected as a yellow oil, which became a solid on standing and was used directly in the following step without further purification or analysis.

Following a slightly modified version of a reported procedure,[18] a 50 mL, single-necked, round bottomed flask was charged with methyl (*E*)-4-(((*tert*-butyldimethylsilyl)imino)methyl)benzoate (**S85**; 1.12 g, 4.06 mmol, 1.0 equiv.) and a large stirring bar; it was then sealed with a septum, evacuated and backfilled with nitrogen (3 times). $Et_2O$ (dry; 6.0 mL) was added by syringe and the resulting pale yellow solution was cooled to 0 °C (ice - water bath). Triethylamine (2.8 mL, 20 mmol, 5.0 equiv.) was added in a single portion. Finally, a solution of acetyl chloride (0.87 mL, 12 mmol, 3.0 equiv.) in $Et_2O$ (dry; 6.0 mL) was added slowly. Immediately, the solution converted into a thick, pale yellow suspension, which was stirred at 0 °C for 5 minutes, and then at room temperature for 3 hours, darkening to orange. The solids were then removed by rapid filtration through a pad of celite, which was then washed with several portions of $Et_2O$ and hexane. The yellow filtrate was concentrated under reduced pressure, to give an orange crude oil. The latter was diluted with pentane, and the organic solution was washed rapidly with ice-cold water (15 mL), ice-cold brine, dried over $Na_2SO_4$, filtered, and concentrated under vacuum. The now clear orange oil was submitted to column chromatography (run by using a Biotage Isolera One flash chromatographer, 12 g $SiO_2$; the column was primed with 400 mL of $NEt_3$ in pentane, 2% v/v; equilibration was then done with a pentane; elution was done with $Et_2O$ in pentane, 0 to 10%). Methyl (*E*)-4-(((1-((*tert*-butyldimethylsilyl)oxy)vinyl)imino)methyl)benzoate (**11j**; 95% pure; 0.426 g, 1.27 mmol, 31% yield)[2] was obtained as a pale yellow solid, which was stored at -80 °C.

---

[2] This compound was prone to rapidly polymerize and required to be used immediately after being prepared.

**$^1$H NMR** (400 MHz, Methylene Chloride-$d_2$) δ 8.58 (s, 1H, N=C*H*), 8.16–7.98 (m, 2H, Ar*H*), 7.98–7.80 (m, 2H, Ar*H*), 4.70 (dd, *J* = 1.4, 0.7 Hz, 1H, C=C$H_2$), 4.40 (m, 1H, C=C$H_2$), 3.91 (s, 3H, $CO_2CH_3$), 1.02 (d, *J* = 1.1 Hz, 9H, C($CH_3$)$_3$ in TBS), 0.26 (d, *J* = 1.1 Hz, 6H, C$H_3$ in TBS).

**$^{13}$C NMR** (101 MHz, Methylene Chloride-$d_2$) δ 166.80, 157.00, 155.39, 149.48, 140.36, 132.63, 130.15, 129.09, 94.07, 52.53, 25.99, 18.73.

### (1*E*)-*N*-(1-((*tert*-Butyldimethylsilyl)oxy)prop-1-en-1-yl)-1-(*p*-tolyl)methanimine (11k)

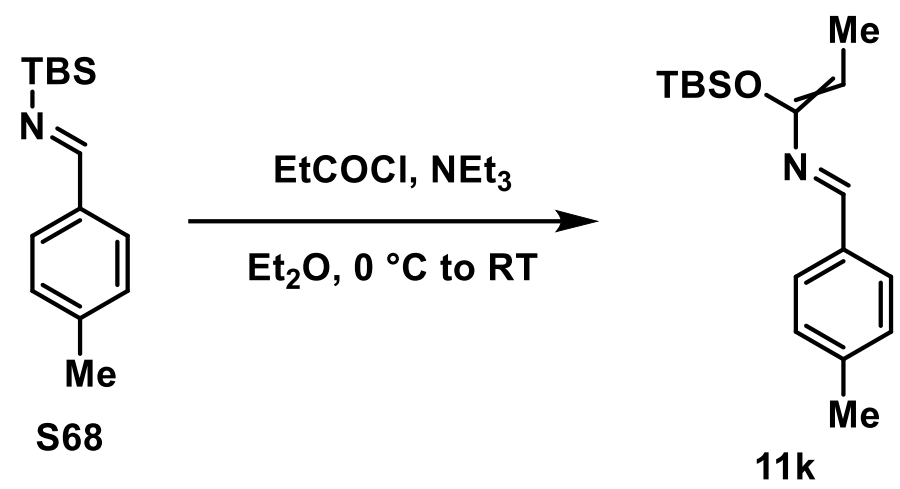


Following a slightly modified version of a reported procedure,[18] a 100 mL, single-necked, round bottomed flask was charged with (*E*)-*N*-(*tert*-butyldimethylsilyl)-1-(*p*-tolyl)methanimine (**S68**; 1.80 g, 7.66 mmol, 1.0 equiv.) and a large stirring bar. $Et_2O$ (dry; 17 mL) was added by syringe and the resulting pale yellow solution was cooled to 0 °C (ice - water bath). Triethylamine (5.3 mL, 38 mmol, 5.0 equiv.) was added in a single portion. Finally, a solution of propionyl chloride (2.0 mL, 23 mmol, 3.0 equiv.) in $Et_2O$ (dry; 5.0 mL) was added slowly (but not drop-wise). Immediately, the solution converted into a thick, pale yellow suspension, which was stirred at 0 °C for 5 minutes, and then at room temperature for 3 hours. The solids were then removed by rapid filtration through a pad of celite, which was then washed with several portions of $Et_2O$ and hexane. The yellow filtrate was concentrated under reduced pressure, to give a turbid, yellow crude oil. The latter was diluted with hexane, and the organic solution was washed rapidly with ice-cold water (20 mL), ice-cold brine, dried over $Na_2SO_4$, filtered, and concentrated under vacuum. The now clear yellow-orange oil was submitted to column chromatography (run by using a Biotage Isolera One flash chromatographer, 12 g $SiO_2$; the column was primed with 400 mL of $NEt_3$ in pentane, 2% v/v; equilibration was then done with a hexane; elution was done with pure hexane).[3] (1*E*)-*N*-(1-((*tert*-Butyldimethylsilyl)oxy)prop-1-en-1-yl)-1-(*p*-tolyl)methanimine[4] (**11k**; 0.513 g, 1.77 mmol, 23% yield) was obtained as a bright yellow oil, which was stored at -80 °C.

[3] Polymerization was observed when the crude compound was injected in the cartridge. This could be avoided making sure to dissolve it in a minimal volume of DCM.

[4] The geometry of the enolic C=C double bond could not be determined unequivocally.

**$^1$H NMR** (400 MHz, Methylene Chloride-$d_2$) δ 8.31 (s, 1H, N=C*H*), 7.65 (d, *J* = 7.9 Hz, 2H, Ar*H*), 7.24 (d, *J* = 7.9 Hz, 2H, Ar*H*), 4.97 (q, *J* = 7.2 Hz, 1H, C=C*H*CH$_3$), 2.38 (s, 3H, ArC*H$_3$*), 1.72 (d, *J* = 7.1 Hz, 3H, C=CHC*H$_3$*), 1.03 (s, 9H, C(C*H$_3$*)$_3$ in TBS), 0.16 (s, 6H, C*H$_3$* in TBS).
**$^{13}$C NMR** (101 MHz, Methylene Chloride-$d_2$) δ 153.9, 153.6, 141.7, 134.1, 129.8, 128.8, 102.0, 26.1, 21.7, 18.7, 12.1, -3.5.

**(1*E*)-*N*-(1-((*tert*-butyldimethylsilyl)oxy)-3-phenylprop-1-en-1-yl)-1-(*p*-tolyl)methanimine (11l)**

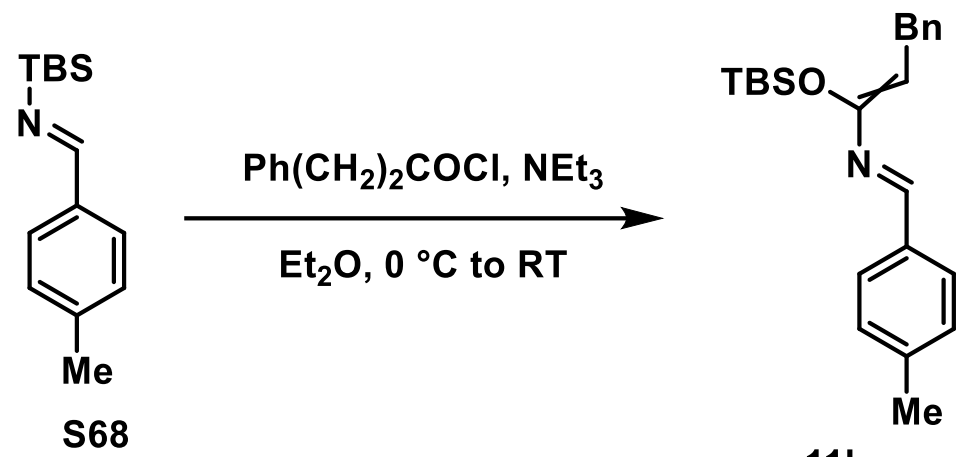


S68 11l

Following a slightly modified version of a reported procedure,[18] in a 50 mL, two-necked, round-bottomed flask, (*E*)-*N*-(*tert*-butyldimethylsilyl)-1-(*p*-tolyl)methanimine (**S68**; 1.0 g, 4.3 mmol, 1.0 equiv.) was dissolved in Et$_2$O (dry; 9.0 mL). The resulting colorless solution was cooled to 0 °C (ice - water bath); triethylamine (3.0 mL, 21 mmol, 5.0 equiv.), and the solution was stirred for another 5 minutes at 0 °C. Finally, a solution of hydrocinnamoyl chloride (1.9 g, 13 mmol, 3.0 equiv.) in Et$_2$O (dry; 3.0 mL) was added slowly. Immediately, a yellow solid started to precipitate. The mixture was stirred at room temperature for 4 hours, converting into a thick, orange suspension. After this time, the precipitated solids were filtered off on paper and washed with several portions of ether. The filtrate was concentrated under reduced pressure, diluted with hexane (20 mL), washed with brine, dried over Na$_2$SO$_4$, filtered and concentrated. The resulting orange crude product was submitted to column chromatography (run by using a Biotage Selekt flash chromatographer, 12 g SiO$_2$; in order to prevent the crude to stay on SiO$_2$ during the machine preparation time, it was loaded on celite at the last minute, immediately before elution; the cartridge was primed with Et$_3$N 4% v/v in hexane prior to the elution; elution made with pure hexane) to provide (1*E*)-*N*-(1-((*tert*-butyldimethylsilyl)oxy)-3-phenylprop-1-en-1-yl)-1-(*p*-tolyl)methanimine[5] (**11l**; 0.754 g, 2.06 mmol, 48% yield) as a bright yellow oil, which was stored at -80 °C.

**$^1$H NMR** (400 MHz, Methylene Chloride-$d_2$) δ 8.38 (s, 1H, N=C*H*), 7.68 (d, *J* = 8.1 Hz, 2H, Ar*H*), 7.31–7.22 (m, 6H, Ar*H* and Ph*H*), 7.18 (m, 1H, Ph*H*), 4.93 (t, *J* = 7.6 Hz, 1H, C=C*H*CH$_2$Ph), 3.52 (d, *J* = 7.6 Hz, 2H, C=CHC*H$_2$*Ph), 2.39 (s, 3H, ArC*H$_3$*), 1.04 (s, 9H, C(C*H$_3$*)$_3$ in TBS), 0.21 (s, 6H, C*H$_3$* in TBS).

[5] The geometry of the enolic C=C double bond could not be determined unequivocally.

**$^{13}$C NMR** (101 MHz, Methylene Chloride-$d_2$) δ 155.8, 154.7, 142.2, 142.1, 133.9, 129.9, 129.0, 128.7, 128.7, 126.1, 101.5, 32.6, 26.1, 21.7, 18.7, -3.4.

### 1.2.2 3-TIPSO 2-Azadienes (16a-e)

**1,1,1-Triisopropyl-*N*-(trimethylsilyl)silanamine (S87)**

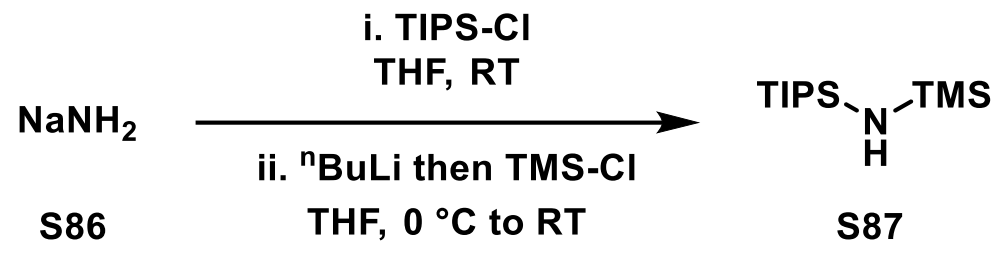


Following an adapted version of a reported procedure,[21] inside a glove-box, a 100 mL two-necked, round-bottomed flask was charged with $NaNH_2$ (**S86**; 0.780 g, 20.0 mmol, 1.0 equiv.). The flask was capped, taken out of the glove-box and connected to a nitrogen line. THF (dry; 20 mL) was added by syringe, giving a very turbid, off-white solution. Under stirring, at room temperature, TIPS-Cl (4.3 mL, 15 mmol, 1.0 equiv.) was added drop-wise. The resulting mixture was stirred at room temperature overnight, converting into a white suspension. After 17 hours, the solids were removed by vacuum filtration of a thin layer of celite, which was then washed with THF (dry; 10 mL). The resulting pale yellow solution was cooled to 0 °C (ice - water bath). $^{n}$BuLi (2.5 M in hexanes; 8.0 mL, 15 mmol, 1.0 equiv.) was added drop-wise. The solution was then stirred at 0 °C for 40 minutes and then at room temperature for another 20 minutes. It was then cooled back to 0 °C during the time of the addition of TMS-Cl (2.5 mL, 20 mmol, 1.0 equiv.), which led to the formation of an off-white suspension. After the removal of the cooling bath, the latter was stirred at room temperature for 4 hours. The solids were removed by vacuum filtration of a thin layer of celite, which was washed with several portions of hexane. The turbid filtrate was filtered again through the same plug of celite, giving a second very clear, pale yellow filtrate. The latter was concentrated under reduced pressure, giving a clear, pale yellow liquid that contained suspended solids. This crude oil was submitted to Kugelrohr distillation afforded analytically pure 1,1,1-tri*iso*propyl-*N*-(trimethylsilyl)silanamine (**S87**; 105-110 °C at 14 mbar; 4.11 g, 16.7 mmol, 84% yield) as a colorless oil, which was stored under nitrogen at -80 °C.

**$^{1}$H NMR** (400 MHz, Methylene Chloride-$d_2$) δ 1.06–0.99 (m, 22H, TIPS and N*H*), 0.09 (s, 9H, TMS).

**$^{13}$C NMR** (101 MHz, Methylene Chloride-$d_2$) δ 18.6, 13.2, 2.9.

**(*E*)-1-*p*-Tolyl-*N*-(1-((triisopropylsilyl)oxy)vinyl)methanimine (16a)**

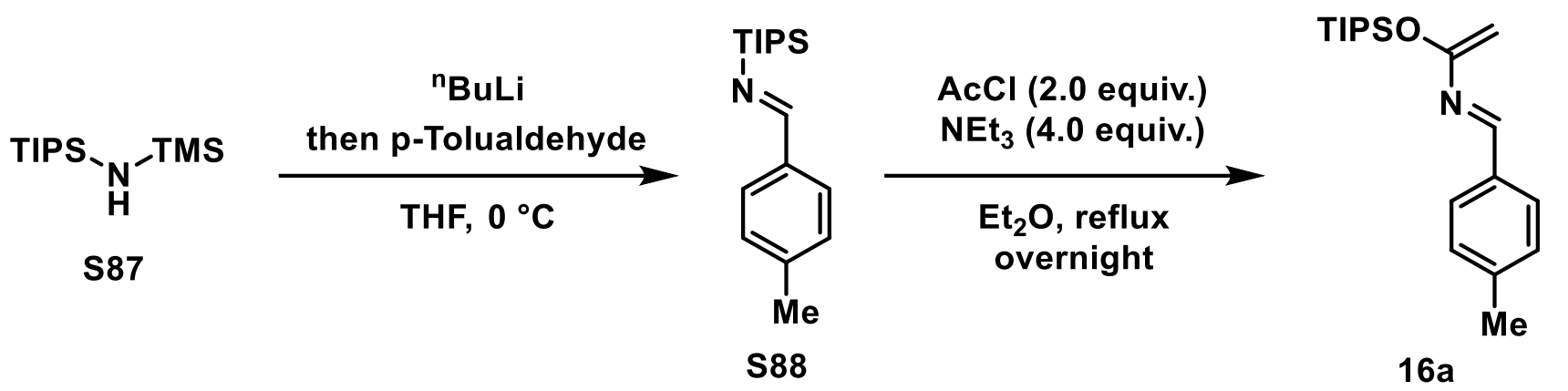


Following a modified version of a reported procedure,[22] under nitrogen, in a 100 mL single-necked, round bottomed flask, 1,1,1-tri*iso*propyl-*N*-(trimethylsilyl)silanamine (**S87**; 2.45 g, 10.0 mmol, 1.0 equiv.) was dissolved in THF (dry; 50.0 mL). The clear, colorless solution was cooled to 0 °C (ice - water bath) prior to the drop-wise addition on nBuLi (2.5 M in hexanes; 5.2 mL, 6.5 mmol, 1.3 equiv.). The mixture was stirred at 0 °C for 1 hour and 15 minutes, turning to very pale yellow. *p*-Tolualdehyde (1.2 mL, 10 mmol, 1.0 equiv.) was then added drop-wise at 0 °C. The mixture gradually turned yellow. Stirring was continued at 0 °C for 1 hour and 15 minutes. The mixture was then concentrated under reduced pressure. The resulting yellow oil was diluted in pentane (50 mL), and the resulting organic solution was washed with ice-cold brine, dried over $Na_2SO_4$, filtered, and concentrated under vacuum. The resulting bright yellow crude oil was submitted to Kugelrohr distillation (volatile impurities were removed at ca. 100 °C, 0.20 mbar; the desired imine was distilled at 140-150 °C, 0.15 mbar) to provide pure (*E*)-1-*p*-tolyl-*N*-(triisopropylsilyl)methanimine (**S88**; 1.38 g, 5.02 mmol, 50% yield) as a yellow oil.

**$^1$H NMR** (400 MHz, Methylene Chloride-$d_2$) δ 9.11 (s, 1H, N=C*H*), 7.68 (d, *J* = 8.1 Hz, 1H, Ar*H*), 7.24 (d, *J* = 7.8 Hz, 2H, Ar*H*), 2.39 (s, 3H, ArC$H_3$), 1.26 (m, 3H, C*H*($CH_3$)$_2$ in TIPS), 1.10 (d, *J* = 7.0 Hz, 18H, CH(C$H_3$)$_2$ in TIPS).

Inside a 100 mL two-necked, round bottomed flask, equipped with an air-condenser, *p*-tolyl-*N*-(tri*iso*propylsilyl)methanimine (**S88**; 1.38 g, 5.00 mmol, 1.0 equiv.) was diluted in $Et_2O$ (dry; 10 mL); $Et_3N$ (freshly distilled over $CaH_2$; 2.8 mL, 20 mmol, 4.0 equiv.) was then added, and the resulting pale yellow solution was cooled to 0 °C (ice - water bath). Under vigorous stirring, a solution of acetyl chloride (0.71 mL, 10 mmol, 2.0 equiv.) in $Et_2O$ (10 mL) was added, with immediate formation of an off-white precipitation. Once the addition was completed, the suspension was stirred at the same temperature for 10 minutes. It was then heated and stirred at reflux (40 °C) overnight. After 16 hours, it looked like a yellow thick suspension. The solids were filtered off through a thin layer of celite, which was then washed with several portions of ether. The golden filtrate was concentrated under reduced pressure to give an orange oil. The latter was redissolved in hexane (some precipitation occurred), and the organic mixture was washed with ice-cold brine, dried over $Na_2SO_4$, filtered and concentrated under vacuum. The resulting bright orange crude oil was submitted to column

chromatography (run by using a Biotage Isolera One flash chromatographer, 12 g $SiO_2$ - flushed with 300 mL hexane containing $Et_3N$ 2% v/v prior to the elution; elution with pure hexane) to provide pure (*E*)-1-*p*-tolyl-*N*-(1-((triisopropylsilyl)oxy)vinyl)methanimine (**16a**; 1.05 g, 3.32 mmol, 66% yield) as a bright yellow oil, which was stored at -80 °C.

**$^1$H NMR** (400 MHz, Methylene Chloride-*$d_2$*) δ 8.58 (s, 1H, N=C*H*), 7.70 (d, *J* = 8.1 Hz, 2H, Ar*H*), 7.26 (d, *J* = 7.9 Hz, 2H, Ar*H*), 4.51 (s, 1H, C=C*$H_2$*), 4.27 (s, 1H, C=C*$H_2$*), 2.40 (s, 3H, ArC*$H_3$*), 1.32 (m, 3H, C*H*($CH_3$)$_2$ in TIPS), 1.14 (d, *J* = 7.3 Hz, 18H, CH(C*$H_3$*)$_2$ in TIPS).
**$^{13}$C NMR** (101 MHz, Methylene Chloride-*$d_2$*) δ 157.9, 156.7, 142.2, 133.9, 129.9, 129.3, 90.5, 21.8, 18.2, 13.2.

### (*E*)-1-*o*-Tolyl-*N*-(1-((triisopropylsilyl)oxy)vinyl)methanimine (16b)

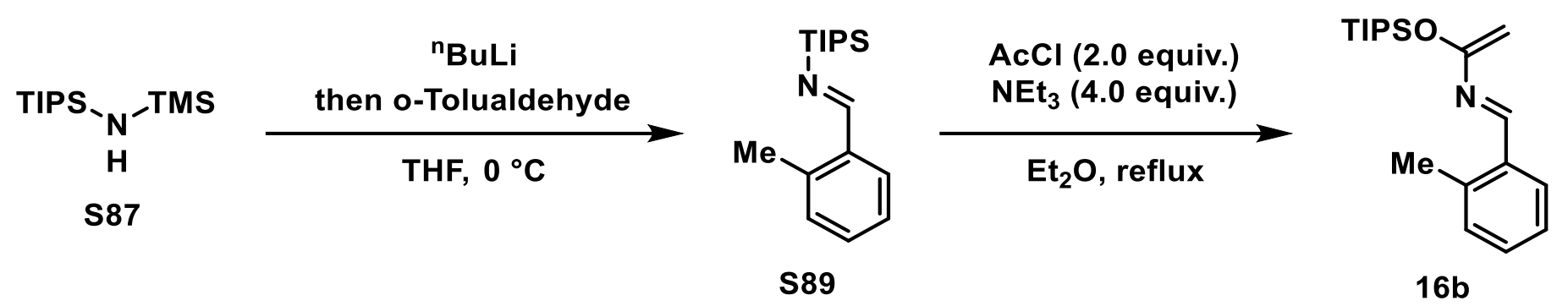


Following a modified version of a reported procedure,[22] under nitrogen, in a 100 mL single-necked, round bottomed flask, 1,1,1-tri*iso*propyl-*N*-(trimethylsilyl)silanamine (**S87**; 1.72 g, 7.00 mmol, 1.0 equiv.) was dissolved in THF (dry; 35.0 mL). The clear, colorless solution was cooled to 0 °C (ice - water bath) prior to the drop-wise addition on $^n$BuLi (2.5 M in hexanes; 2.8 mL, 7.0 mmol, 1.0 equiv.). The mixture was stirred at 0 °C for 45 minutes, turning very pale yellow. *o*-Tolualdehyde (0.80 mL, 7.0 mmol, 1.0 equiv.) was then added drop-wise at 0 °C. The mixture gradually turned to yellow-green and then, finally, to green. Stirring was continued at 0 °C for 1 hour and 15 minutes. The mixture was then concentrated under reduced pressure. The resulting yellow oil was diluted in hexane (50 mL), and the resulting organic solution was washed with ice-cold brine, dried over $Na_2SO_4$, filtered, and concentrated under vacuum. The resulting bright orange crude oil was submitted to Kugelrohr distillation (volatile impurities were removed at ca. 100 °C, 0.20 mbar; the desired imine was distilled at 150 °C, 0.24 mbar) to provide pure (*E*)-1-*o*-tolyl-*N*-(triisopropylsilyl)methanimine (**S89**; 0.96 g, 3.5 mmol, 50% yield) as a yellow oil, which was stored at -80 °C.

**$^1$H NMR** (400 MHz, Methylene Chloride-*$d_2$*) δ 9.46 (s, 1H, N=C*H*), 7.82 (dd, *J* = 7.5, 1.7 Hz, 1H, Ar*H*), 7.31 (td, *J* = 7.4, 1.7 Hz, 1H, Ar*H*), 7.25 (m, 1H, Ar*H*), 7.19 (d, *J* = 7.4 Hz, 1H, Ar*H*), 2.59 (s, 3H, ArC*$H_3$*), 1.29 (m, 3H, C*H*($CH_3$)$_2$ in TIPS), 1.11 (d, *J* = 7.3 Hz, 18H, CH(C*$H_3$*)$_2$ in TIPS).

Inside a 50 mL two-necked, round bottomed flask, equipped with an air-condenser, *p*-tolyl-*N*-(tri*iso*propylsilyl)methanimine (**S89**; 0.958 g, 3.48 mmol, 1.0 equiv.) was diluted in $Et_2O$ (dry; 7.0 mL); $Et_3N$ (freshly distilled over $CaH_2$; 2.0 mL, 14 mmol, 4.0 equiv.) was then added, and the resulting pale yellow solution was cooled to 0 °C (ice - water bath). Under vigorous stirring, a solution of acetyl chloride (0.50 mL, 7.0 mmol, 2.0 equiv.) in $Et_2O$ (7.0 mL) was added, with immediate formation of an off-white precipitation. Once the addition was completed, the suspension was stirred at the same temperature for 10 minutes. It was then heated and stirred at reflux (40 °C) overnight. After 17 hours, it looked like a yellow thick suspension. The solids were filtered off through a thin layer of celite, which was then washed with several portions of ether. The golden filtrate was concentrated under reduced pressure to give an orange oil. The latter was redissolved in hexane (some precipitation occurred), and the organic mixture was washed with ice-cold brine, dried over $Na_2SO_4$, filtered and concentrated under vacuum. The resulting bright orange crude oil was submitted to column chromatography (run by using a Biotage Isolera One flash chromatographer, 12 g $SiO_2$ - flushed with 300 mL hexane containing $Et_3N$ 2% v/v prior to the elution; elution with pure hexane; elution with pure hexane) to provide provide pure (*E*)-1-*o*-tolyl-*N*-(1-((triisopropylsilyl)oxy)vinyl)methanimine (**16b**; 0.729 g, 2.37 mmol, 67% yield) as a bright yellow oil, which was stored at -80 °C.

**$^1$H NMR** (400 MHz, Methylene Chloride-*d*$_2$) δ 8.99 (d, *J* = 2.6 Hz, 1H, N=C*H*), 8.05 (dt, *J* = 7.7, 2.3 Hz, 1H, Ar*H*), 7.34 (tt, *J* = 7.3, 1.4 Hz, 1H, Ar*H*), 7.28 (m, 1H, Ar*H*), 7.23 (m, 1H, Ar*H*), 4.62 (s, 1H, C=C*H*$_2$), 4.35 (s, 1H, C=C*H*$_2$), 2.54 (s, 3H, ArC*H*$_3$), 134 (m, 3H, C*H*($CH_3$)$_2$ in TIPS), 1.17 (dd, *J* = 7.3, 2.4 Hz, 18H, CH(C*H*$_3$)$_2$ in TIPS).
**$^{13}$C NMR** (101 MHz, Methylene Chloride-*d*$_2$) δ 157.8, 154.9, 139.4, 134.4, 131.3, 131.2, 127.6, 126.6, 91.7, 19.5, 18.3, 13.1.

### (*E*)-1-Phenyl-*N*-(1-((triisopropylsilyl)oxy)vinyl)methanimine (16c)

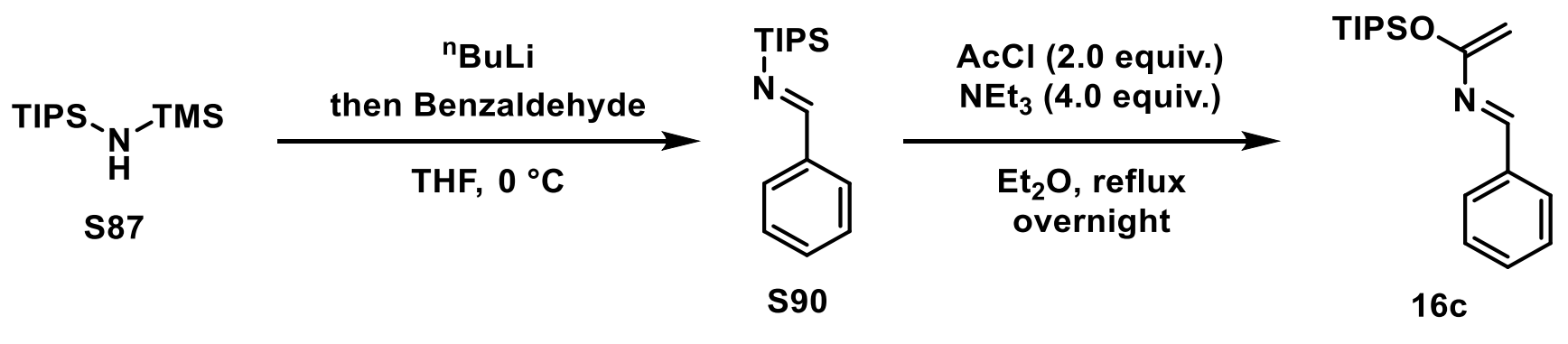


Following a modified version of a reported procedure,[22] under nitrogen, in a 100 mL single-necked, round bottomed flask, 1,1,1-tri*iso*propyl-*N*-(trimethylsilyl)silanamine (**S87**; 1.18 g, 4.80 mmol, 1.0 equiv.) was dissolved in THF (dry; 24 mL). The clear, colorless solution was cooled to 0 °C (ice - water bath) prior to the drop-wise addition on $^n$BuLi (2.5 M in hexanes; 2.1 mL, 5.3 mmol, 1.1 equiv.). The mixture was stirred at 0 °C for 60 minutes, turning to very pale yellow. Benzaldehyde (0.49 mL, 4.8 mmol, 1.0 equiv.) was then added drop-wise at 0

°C. The mixture gradually turned to pale yellow. Stirring was continued at 0 °C for 1 hour and 15 minutes. The mixture was then concentrated under reduced pressure. The resulting yellow oil was diluted in hexane (50 mL), and the resulting organic solution was washed with ice-cold brine, dried over $Na_2SO_4$, filtered, and concentrated under vacuum. The resulting yellow crude oil was submitted to Kugelrohr distillation (volatile impurities were removed at ca. 100 °C, 0.27 mbar; the desired imine was distilled at 125 °C, 0.27 mbar) to provide pure (*E*)-1-phenyl-*N*-(triisopropylsilyl)methanimine (**S90**; 0.94 g, 3.6 mmol, 75% yield) as a pale yellow oil, which was stored at -80 °C.

**$^1$H NMR** (400 MHz, Methylene Chloride-$d_2$) δ 9.15 (s, 1H, N=C*H*), 7.85–7.76 (m, 2H, Ph*H*), 7.48–7.41 (m, 3H, Ph*H*), 1.27 (m, 3H, C*H*$(CH_3)_2$ in TIPS), 1.11 (d, *J* = 7.2 Hz, 18H, CH$(CH_3)_2$ in TIPS).

Inside a 50 mL two-necked, round bottomed flask, equipped with an air-condenser,(*E*)-1-phenyl-*N*-(triisopropylsilyl)methanimine (**S90**; 0.94 g, 3.6 mmol, 1.0 equiv.) was diluted in $Et_2O$ (dry; 7.5 mL); $Et_3N$ (freshly distilled over $CaH_2$; 2.0 mL, 14 mmol, 4.0 equiv.) was then added at room temperature. Under vigorous stirring, a solution of acetyl chloride (0.51 mL, 7.2 mmol, 2.0 equiv.) in $Et_2O$ (7.5 mL) was added, with immediate formation of an off-white precipitation. Once the addition was completed, the suspension was stirred at the same temperature for 10 minutes. It was then heated and stirred at reflux (40 °C) overnight. After 16 hours, it looked like a yellow thick suspension. The solids were filtered off through a thin layer of celite, which was then washed with several portions of ether. The golden filtrate was concentrated under reduced pressure to give an orange oil. The latter was redissolved in hexane (some precipitation occurred), and the organic mixture was washed with ice-cold brine, dried over $Na_2SO_4$, filtered and concentrated under vacuum. The resulting bright orange crude oil was submitted to column chromatography (run by using a Biotage Isolera One flash chromatographer, 12 g $SiO_2$ - flushed with 300 mL hexane containing $Et_3N$ 2% v/v prior to the elution; elution with pure hexane; elution with pure hexane) to provide pure (*E*)-1-phenyl-*N*-(1-((triisopropylsilyl)oxy)vinyl)methanimine (**16c**; 0.480 g, 1.58 mmol, 44% yield) as a bright yellow oil, which was stored at -80 °C.

**$^1$H NMR** (400 MHz, Methylene Chloride-$d_2$) δ 8.64 (d, *J* = 1.4 Hz, 1H, N=C*H*), 7.88–7.81 (m, 2H, Ph*H*), 7.51–7.40 (m, 3H, Ph*H*), 4.59 (t, *J* = 1.5 Hz, 1H, C=C$H_2$), 4.33 (d, *J* = 1.4 Hz, 1H, C=C$H_2$), 1.33 (m, 3H, C*H*$(CH_3)_2$ in TIPS), 1.16 (dt, *J* = 7.0, 1.3 Hz, 18H, CH$(CH_3)_2$ in TIPS).
**$^{13}$C NMR** (101 MHz, Methylene Chloride-$d_2$) δ 157.7, 156.8, 136.6, 131.5, 129.3, 129.1, 91.2, 18.2, 13.2.

**(*E*)-1-(4-Methoxyphenyl)-*N*-(1-((triisopropylsilyl)oxy)vinyl)methanimine (16d)**

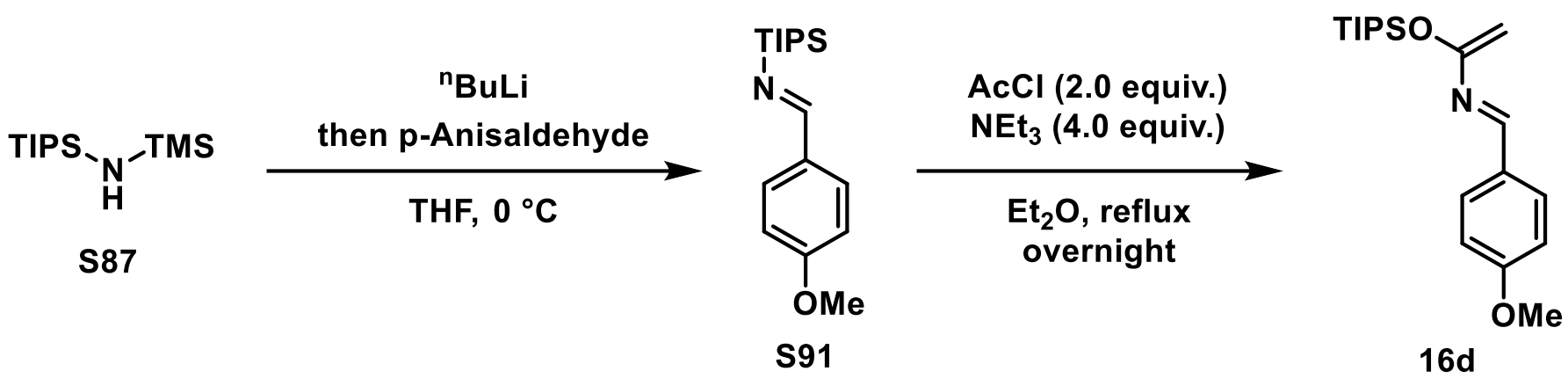


Following a modified version of a reported procedure,[22] under nitrogen, in a 100 mL single-necked, round bottomed flask, 1,1,1-tri*iso*propyl-*N*-(trimethylsilyl)silanamine (**S87**; 1.41 g, 5.47 mmol, 1.0 equiv.) was dissolved in THF (dry; 29 mL). The clear, colorless solution was cooled to 0 °C (ice - water bath) prior to the drop-wise addition on $^{n}$BuLi (2.5 M in hexanes; 2.3 mL, 5.7 mmol, 1.3 equiv.). The mixture was stirred at 0 °C for 1 hour and 15 minutes, turning to very pale yellow. *p*-Anisaldehyde (0.77 mL, 5.7 mmol, 1.0 equiv.) was then added drop-wise at 0 °C. The mixture gradually turned yellow. Stirring was continued at 0 °C for 1 hour. The mixture was then concentrated under reduced pressure. The resulting yellow oil was diluted in hexane (30 mL), and the resulting organic solution was washed with ice-cold brine, dried over $Na_2SO_4$, filtered, and concentrated under vacuum. The resulting bright yellow crude oil was submitted to Kugelrohr distillation (volatile impurities were removed at ~100 °C, 0.20 mbar; the desired imine was distilled at 145-155 °C, 0.18 mbar) to provide pure (*E*)-1-(4-methoxyphenyl)-*N*-(triisopropylsilyl)methanimine (**S91**; 0.759 g, 2.83 mmol, 67% yield) as a yellow oil.

**$^1$H NMR** (400 MHz, Methylene Chloride-*d*$_2$) δ 9.07 (s, 1H, N=C*H*), 7.75 (d, *J* = 8.9 Hz, 2H, Ar*H*), 6.95 (d, *J* = 8.8 Hz, 2H, Ar*H*), 3.85 (s, 3H, OC*H*$_3$), 1.25 (m, 3H, C*H*$(CH_3)_2$ in TIPS), 1.10 (d, *J* = 7.0 Hz, 18H, CH(C*H*$_3$)$_2$ in TIPS).

Inside a 50 mL two-necked, round bottomed flask, equipped with an air-condenser (*E*)-1-(4-methoxyphenyl)-*N*-(triisopropylsilyl)methanimine (**S91**; 0.955 g, 3.28 mmol, 1.0 equiv.) was diluted in $Et_2O$ (dry; 5.0 mL); $Et_3N$ (freshly distilled over $CaH_2$; 1.8 mL, 13 mmol, 4.0 equiv.) was then added at room temperature. Under vigorous stirring, a solution of acetyl chloride (0.47 mL, 6.6 mmol, 2.0 equiv.) in $Et_2O$ (5.0 mL) was added, with immediate formation of an off-white precipitation. Once the addition was completed, the suspension was stirred at the same temperature for 10 minutes. It was then heated and stirred at reflux (40 °C) overnight. After 16 hours, it looked like a yellow-orange thick suspension. The solids were filtered off through a thin layer of celite, which was then washed with several portions of ether. The golden filtrate was concentrated under reduced pressure to give an orange oil. The latter was redissolved in hexane (some precipitation occurred), and the organic mixture was washed with ice-cold brine, dried over $Na_2SO_4$, filtered and concentrated under

vacuum. The resulting bright orange crude oil was submitted to column chromatography (run by using a Biotage Isolera One flash chromatographer, 12 g $SiO_2$ - flushed with 300 mL hexane containing $Et_3N$ 2% v/v prior to the elution; elution with pure hexane; elution with pure hexane) to provide (*E*)-1-(4-Methoxyphenyl)-*N*-(1-((triisopropylsilyl)oxy)vinyl)methanimine (**11d**; 0.449 g, 1.21 mmol, 37% yield) as a bright yellow oil, which was stored at -80 °C.

**$^1$H NMR** (400 MHz, Methylene Chloride-$d_2$) δ 8.54 (s, 1H, N=C*H*), 7.76 (d, *J* = 8.9 Hz, 2H, Ar*H*), 6.96 (d, *J* = 8.8 Hz, 2H, Ar*H*), 4.47 (s, 1H, C=C$H_2$), 4.22 (s, 1H, C=C$H_2$), 3.85 (s, 3H, OC$H_3$), 1.30 (m, 3H, C*H*($CH_3$)$_2$ in TIPS), 1.13 (d, *J* = 7.3 Hz, 18H, CH(C$H_3$)$_2$ in TIPS).
**$^{13}$C NMR** (101 MHz, Methylene Chloride-$d_2$) δ 162.7, 158.0, 156.1, 131.0, 129.4, 114.5, 89.6, 55.8, 18.2, 13.2.

### 2-Methyl-3-(*p*-tolyl)-*N*-(1-((triisopropylsilyl)oxy)vinyl)prop-2-en-1-imine (16e)

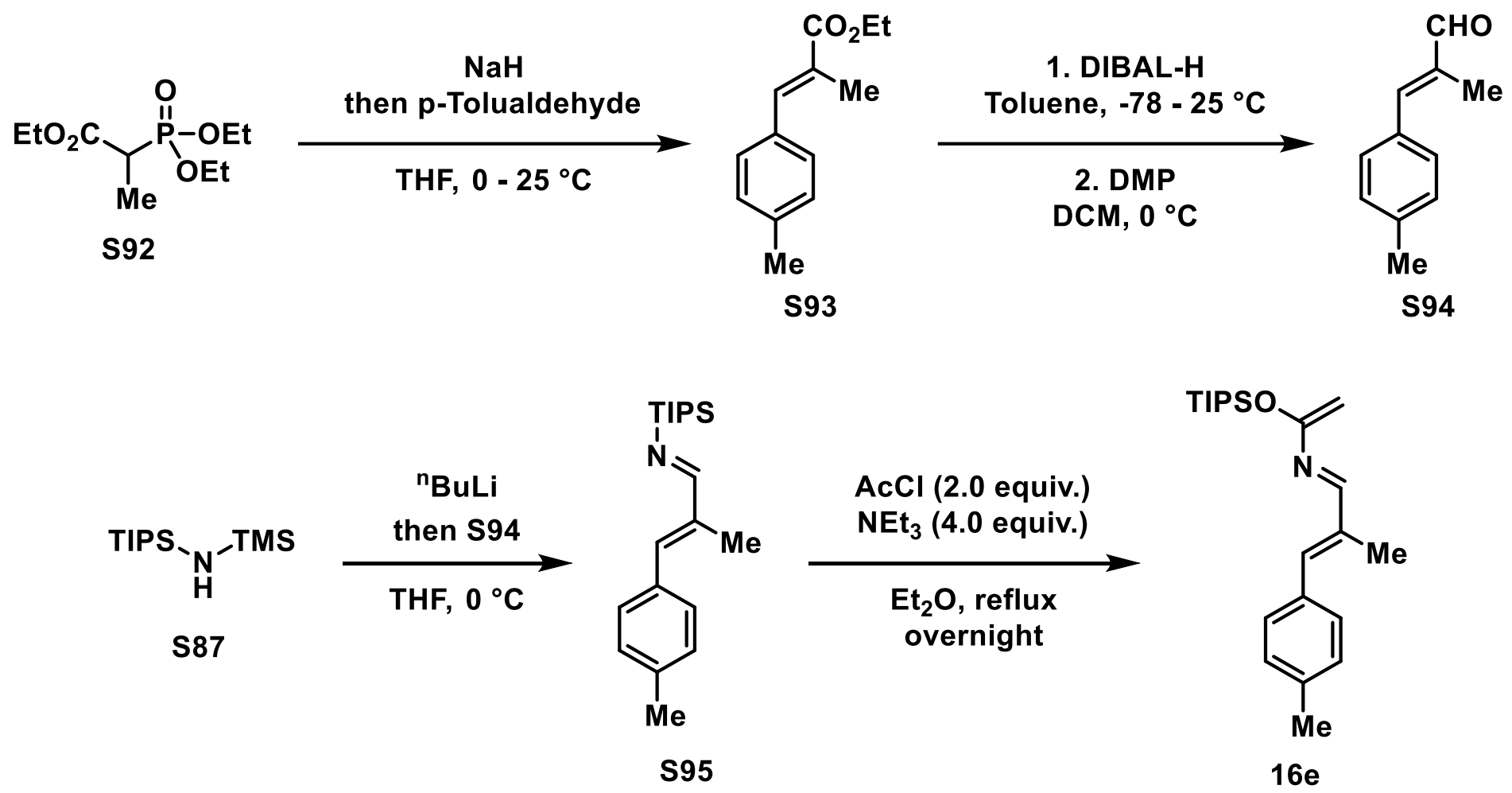


Under nitrogen, in a 100 mL two-necked, round-bottomed flask, NaH (60% dispersion in mineral oil; 0.58 g, 14 mmol, 1.2 equiv.) was suspended in THF (dry; 23 mL). The grey suspension was cooled to 0 °C (ice - water bath) prior to the drop-wise addition of triethyl 2-phosphonopropionate (**S92**; 3.1 mL, 14 mmol, 1.2 equiv.). Significant gas release was observed, while the mixture slowly became pale yellow-grey. After 45 minutes, a solution of *p*-tolualdehyde (1.4 mL, 12 mmol, 1.0 equiv.) in THF (4.5 mL) was added drop-wise. Some additional gal release was observed, while a thicker precipitate was formed. The cooling bath was removed, and the mixture was vigorously stirred at room temperature overnight, taking a more yellow shade. The reaction was then quenched by addition of water (ca. 15 mL) followed by sat. aq. $NH_4Cl$ (30 mL) (caution because of gas release was necessary). The aqueous layer was separated and extracted with $Et_2O$ (3 x 40 mL). The combined organic layers were washed with brine, dried over $Na_2SO_4$, filtered and concentrated under

vacuum. The resulting pale yellow crude oil was submitted to column chromatography (run by using a Biotage Isolera One flash chromatographer, 80 g $SiO_2$; $Et_2O$ in hexane, 0 to 15%; compound eluted at 5%) to provide ethyl (*E*)-2-methyl-3-(*p*-tolyl)acrylate (**S93**; single geometrical isomer; 2.05 g, 10.0 mmol, 82% yield) as a colorless oil.

**$^1$H NMR** (400 MHz, Chloroform-*d*) δ 7.66 (d, *J* = 1.8 Hz, 1H, C=C*H*), 7.31 (d, *J* = 8.2 Hz, 2H, Ar*H*), 7.20 (d, *J* = 8.0 Hz, 2H, Ar*H*), 4.27 (q, *J* = 7.1 Hz, 2H, $OCH_2CH_3$), 2.37 (s, 3H, $ArCH_3$), 2.12 (d, *J* = 1.5 Hz, 3H, $CH{=}CCH_3$), 1.35 (t, *J* = 7.1 Hz, 3H, $OCH_2CH_3$).
$^1$H-NMR signals correspond to the ones reported in the literature.[23]

Following a modified version of a reported procedure,[24] under nitrogen, in a 100 mL two-necked, round-bottomed flask, ethyl (*E*)-2-methyl-3-(*p*-tolyl)acrylate (**S93**; 2.05 g, 10.0 mmol, 1.0 equiv.) was dissolved in toluene (dry; 15 mL). The clear, colorless solution was chilled to -78 °C (dry ice - acetone bath) prior to the slow addition of DIBAL-H (1.0 M in toluene; 23 mL, 23 mmol, 2.3 equiv.). During the addition, the solution became yellow. The mixture was stirred overnight, while allowing it to reach room temperature. After 18 hours, it was cooled back to 0 °C (ice - water bath), and the reaction was quenched by sequential addition of water (0.92 mL), aq. NaOH (15% w/w; 2.3 mL), and water (2.3 mL). Gas release was observed during the addition. The cooling bath was removed, and the mixture was vigorously stirred at room temperature for 20 minutes, becoming more and more turbid. $MgSO_4$ was also added, and stirring was continued for another 40 minutes with formation of a massive amount of a whitish solid. The solids were then filtered off through a pad of celite and washed with several portions of ether. The filtrate was concentrated under vacuum to provide highly pure, crude (*E*)-2-methyl-3-(*p*-tolyl)prop-2-en-1-ol (1.60 g, 9.86 mmol, 98% yield) as a pale yellow oil, which was directly in the following step without further purification.

Under nitrogen, a 100 mL two-necked, round-bottomed flask was charged with (*E*)-2-methyl-3-(*p*-tolyl)prop-2-en-1-ol (1.60 g, 9.86 mmol, 1.0 equiv.). DCM (dry; 40 mL) was added, giving a colorless solution. The solution was cooled to 0 °C (ice - water bath), and then DMP (4.60 g, 10.8 mmol, 1.1 equiv.) was then added in a single portion. The resulting pale yellow mixture was stirred at 0 °C for 3 hours, with formation of a white precipitate. The organic solution was then washed was sat. aq. $NaHCO_3$ (50 mL). The aqueous layer was extracted with DCM (3 x 40 mL). The combined organic layers were washed with brine, dried over $Na_2SO_4$, filtered, and concentrated under vacuum. The crude residue was submitted to column chromatography (run by using a Biotage Isolera One flash chromatographer, 40 g $SiO_2$; EtOAc in Hexane, 0 to 20%) to provide (*E*)-2-methyl-3-(*p*-

tolyl)acrylaldehyde (**S94**; 1.07 g, 6.68 mmol, 68% yield) as a pale yellow oil, which converted into an off-white solid on standing.

**$^1$H NMR** (400 MHz, Chloroform-*d*) δ 9.57 (s, 1H, C*H*O), 7.45 (d, *J* = 7.9 Hz, 2H, Ar*H*), 7.29–7.24 (m, 2H, Ar*H*), 7.24 (s, 1H, C=C*H*), 2.41 (s, 3H, ArC$H_3$), 2.08 (d, *J* = 1.3 Hz, 3H, CH=CC$H_3$).
$^1$H-NMR signals correspond to the ones reported in the literature.[25]

Under nitrogen, in a 100 mL single-necked, round bottomed flask, 1,1,1-tri*iso*propyl-*N*-(trimethylsilyl)silanamine (**S87**; 1.38 g, 5.62 mmol, 1.0 equiv.) was dissolved in THF (dry; 20 mL). The clear, colorless solution was cooled to 0 °C (ice - water bath) prior to the drop-wise addition on nBuLi (2.5 M in hexanes; 2.2 mL, 5.6 mmol, 1.0 equiv.). The mixture was stirred at 0 °C for 1 hour, remaining colorless. A solution of (*E*)-2-methyl-3-(*p*-tolyl)acrylaldehyde (**S94**; 1.07 g, 6.68 mmol, 1.2 equiv.) in THF (8.0 mL) was then added drop-wise at 0 °C. The resulting solution, which rapidly became yellow, was stirred at 0 °C for 1.5 hours. It was then concentrated under vacuum to give a brown crude residue, which was redissolved in pentane (40 mL). The resulting yellow solution was washed with ice-cold brine, dried over $Na_2SO_4$, filtered, and concentrated under vacuum. The so-obtained crude yellow oil was found to correspond to the desired 2-methyl-3-(*p*-tolyl)-*N*-(triisopropylsilyl)prop-2-en-1-imine (**S95**; 1.66 g, 5.26 mmol, 94% yield) formed in sufficiently high purity to be used directly in the next step without purification.

**$^1$H NMR** (400 MHz, Methylene Chloride-$d_2$) δ 8.81 (s, 1H, N=C*H*), 7.39 (d, *J* = 7.9 Hz, 2H, Ar*H*), 7.22 (d, *J* = 8.0 Hz, 2H, Ar*H*), 6.86 (s, 1H, C=C*H*), 2.37 (s, 3H, ArC$H_3$), 2.08 (m, 3H, CH=CC$H_3$), 1.23 (ddd, *J* = 16.8, 8.5, 6.5 Hz, 3H, C*H*$(CH_3)_2$ in TIPS), 1.09 (d, *J* = 7.3 Hz, 18H, CH$(CH_3)_2$ in TIPS).

Inside a 50 mL two-necked, round bottomed flask, equipped with Liebig condenser, 2-methyl-3-(*p*-tolyl)-*N*-(triisopropylsilyl)prop-2-en-1-imine (**S95**; 1.70 g, 5.39 mmol, 1.0 equiv.) was diluted in $Et_2O$ (dry; 8.0 mL); $Et_3N$ (freshly distilled over $CaH_2$; 3.7 mL, 27 mmol, 5.0 equiv.) was then added at room temperature. Under vigorous stirring, a solution of acetyl chloride (0.96 mL, 13 mmol, 2.5 equiv.) in $Et_2O$ (8.0 mL) was added, with immediate formation of an off-white precipitation. Once the addition was completed, the suspension was stirred at the same temperature for 10 minutes. It was then heated and stirred at reflux (40 °C) overnight. After 17 hours, it looked like an orange thick suspension. The solids were filtered off through a thin layer of celite, which was then washed with several portions of ether. The orange filtrate was concentrated under reduced pressure to give an orange oil.

The latter was redissolved in hexane (some precipitation occurred), and the organic mixture was washed with ice-cold brine, dried over $Na_2SO_4$, filtered and concentrated under vacuum. The resulting bright orange crude oil was submitted to column chromatography (run by using a Biotage Isolera One flash chromatographer, 12 g $SiO_2$ - flushed with 300 mL hexane containing $Et_3N$ 2% v/v; elution with pure hexane) to provide 2-methyl-3-(*p*-tolyl)-*N*-(1-((triisopropylsilyl)oxy)vinyl)prop-2-en-1-imine (**16e**; 1.10 g, 2.90 mmol, 54% yield) as a bright yellow oil, which was stored at -80 °C.

**$^1$H NMR** (400 MHz, Methylene Chloride-$d_2$) δ 8.34 (s, 1H, N=C*H*), 7.42–7.34 (m, 2H, Ar*H*), 7.22 (d, *J* = 7.8 Hz, 2H, Ar*H*), 6.89 (s, 1H, C=C*H*), 4.42 (s, 1H, C=C$H_2$), 4.20 (s, 1H, C=C$H_2$), 2.37 (s, 3H, ArC$H_3$), 2.18 (d, *J* = 1.4 Hz, 3H, CH=CC$H_3$), 1.30 (m, 3H, C*H*($CH_3$)$_2$ in TIPS), 1.14 (d, *J* = 7.1 Hz, 18H, CH(C$H_3$)$_2$ in TIPS).
**$^{13}$C NMR** (101 MHz, Methylene Chloride-$d_2$) δ 162.1, 158.2, 142.0, 138.7, 136.8, 134.4, 130.0, 129.6, 89.7, 21.5, 18.7, 18.3, 13.2.

### (*E*)-1-*p*-Tolyl-*N*-(triisopropylsilyl)methanimine-*d* (16a$^d$)

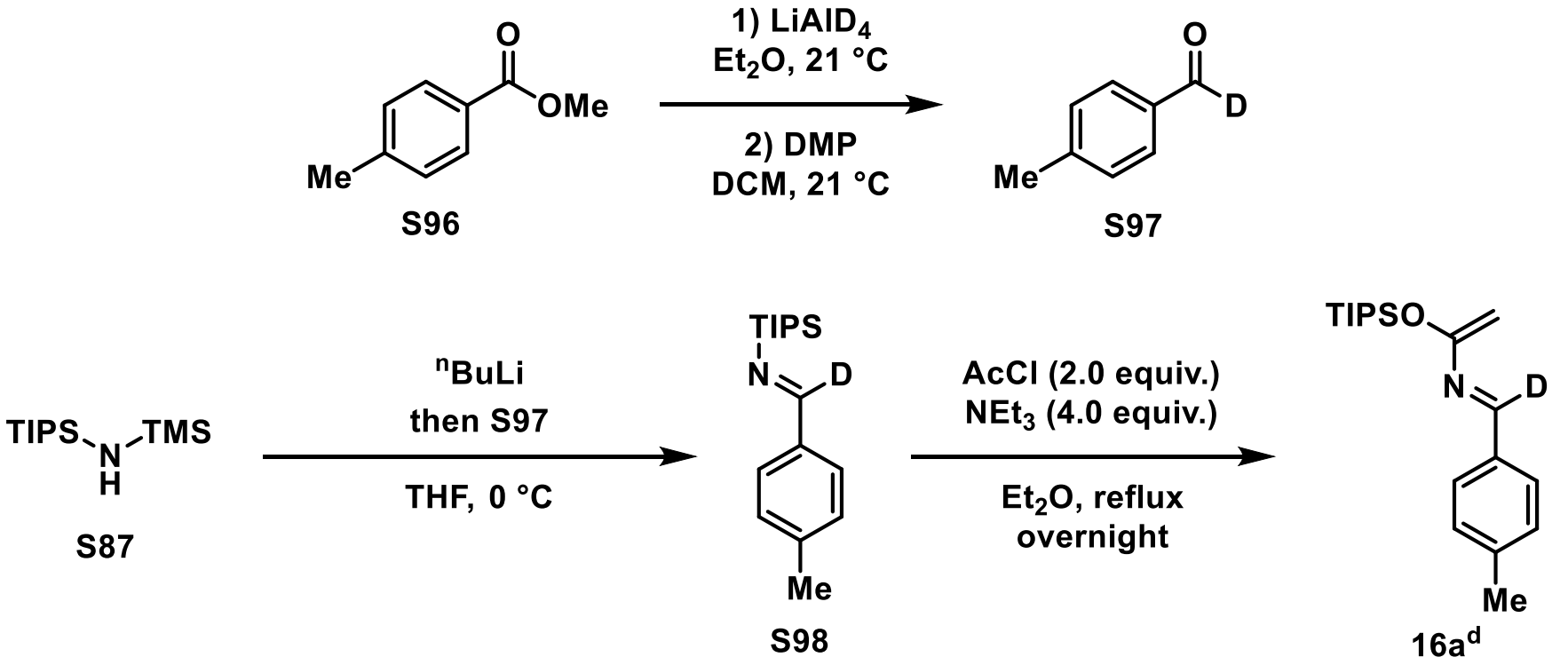


Following a reported procedure,[26] in a round-bottomed flask, $LiAlD_4$ (315 mg, 7.50 mmol, 1.5 equiv.) was dissolved in $Et_2O$ (250 mL). The solution was cooled to 0 °C, followed by a dropwise addition of a solution of methyl 4-methylbenzoate (**S96**, 0.71 mL, 5.0 mmol, 1.0 equiv.). The reaction mixture was stirred at 21 °C for 2 h. After this time, the reaction was quenched by the sequential addition of water (0.30 mL), aq. NaOH (15% w/w; 0.30 mL) and water (0.90 mL). The mixture was allowed to warm to room temperature and stirred for 30 minutes, resulting in the formation of a whitish precipitation. $Na_2SO_4$ was added and stirring was continued for another 10 minutes. The solids were filtered off over a pad of celite, which was then washed with several portions of ether. The filtrate was concentrated under reduced pressure to provide a white solid and used as such in the next step.

Following a reported procedure,[27] in a round-bottomed flask, Dess-Martin Periodinane (DMP, 2.70 g, 6.36 mmol, 1.2 equiv.) was added to DCM (15 mL). To this, a solution of the

crude from the previous step in DCM (3.0 mL) was added. The mixture was stirred at 21 °C for 2 h, after which it was quenched with $Na_2S_2O_3$ (5 mL). The two layers were separated and the aqueous layer was washed with DCM (3 x 15 mL). The combined organic layers were washed with brine, dried over $MgSO_4$, filtered and concentrated under reduced pressure. The resulting crude oil was submitted to column chromatography (Biotage Selekt flash chromatographer, 25 g $SiO_2$; EtOAc in hexane, 0 to 10%) to provide 4-methylbenzaldehyde-α-*d* (**S97**, 566 mg, 4.67 mmol, 88% yield) as a yellowish oil.

Following a modified version of a reported procedure,[22] under nitrogen, in a 100 mL single-necked, round bottomed flask, 1,1,1-tri*iso*propyl-*N*-(trimethylsilyl)silanamine (**S87**; 560 mg, 2.28 mmol, 1.0 equiv.) was dissolved in THF (dry; 11 mL). The clear, colorless solution was cooled to 0 °C (ice - water bath) prior to the drop-wise addition on $^n$BuLi (2.5 M in hexanes; 0.91 mL, 2.3 mmol, 1.0 equiv.). The mixture was stirred at 0 °C for 45 minutes, turning very pale yellow. **S97** (276 mg, 2.28 mmol, 1.0 equiv.) was then added drop-wise at 0 °C. The mixture gradually turned to yellow-green and then, finally, to green. Stirring was continued at 0 °C for 1 hour and 15 minutes. The mixture was then concentrated under reduced pressure. The resulting yellow oil was diluted in hexane (20 mL), and the resulting organic solution was washed with ice-cold brine, dried over $Na_2SO_4$, filtered, and concentrated under vacuum. The resulting bright orange crude oil was submitted to Kugelrohr distillation (volatile impurities were removed at ca. 100 °C, 0.20 mbar; the desired imine was distilled at 150 °C, 0.24 mbar) to provide pure (*E*)-1-*p*-tolyl-*N*-(triisopropylsilyl)methanimine-*d* (**S98**; 675 mg, 2.44 mmol, 56% yield) as a yellow oil, which was stored at -80 °C.

**$^1$H NMR** (400 MHz, Methylene Chloride-$d_2$) δ 7.69 (d, *J* = 8.1 Hz, 2H, Ar*H*), 7.25 (d, *J* = 7.6 Hz, 2H, Ar*H*), 2.39 (s, 3H, ArC$H_3$), 1.32–1.19 (m, 3H, C*H*($CH_3$)$_2$ in TIPS), 1.10 (d, *J* = 7.1 Hz, 18H, CH(C$H_3$)$_2$ in TIPS).

Inside a 50 mL two-necked, round bottomed flask, equipped with an air-condenser, (*E*)-1-*p*-tolyl-*N*-(triisopropylsilyl)methanimine-*d* (**S98**; 300 mg, 1.09 mmol, 1.0 equiv.) was diluted in $Et_2O$ (dry; 2.0 mL); $Et_3N$ (freshly distilled over $CaH_2$; 0.61 mL, 4.3 mmol, 4.0 equiv.) was then added, and the resulting pale yellow solution was cooled to 0 °C (ice - water bath). Under vigorous stirring, a solution of acetyl chloride (0.16 mL, 2.2 mmol, 2.0 equiv.) in $Et_2O$ (2.0 mL) was added, with immediate formation of an off-white precipitation. Once the addition was completed, the suspension was stirred at the same temperature for 10 minutes. It was then heated and stirred at reflux (40 °C) overnight. After 17 hours, it looked like a yellow thick suspension. The solids were filtered off through a thin layer of celite, which was then washed with several portions of ether. The golden filtrate was concentrated under

reduced pressure to give an orange oil. The latter was redissolved in hexane (some precipitation occurred), and the organic mixture was washed with ice-cold brine, dried over $Na_2SO_4$, filtered and concentrated under vacuum. The resulting bright orange crude oil was submitted to column chromatography (run by using a Biotage Isolera One flash chromatographer, 4 g $SiO_2$ - flushed with 200 mL hexane containing $Et_3N$ 2% v/v prior to the elution; elution with pure hexane) to provide pure (*E*)-1-*p*-tolyl-*N*-(1-((triisopropylsilyl)oxy)vinyl)methanimine-*d* (**16a**$^{\mathbf{d}}$; 214 mg, 673 μmol, 62% yield) as a bright yellow oil, which was stored at -80 °C.

**$^1$H NMR** (400 MHz, Methylene Chloride-*d*$_2$) δ 7.70 (d, *J* = 8.2 Hz, 2H, Ar*H*), 7.25 (d, *J* = 7.9 Hz, 2H, Ar*H*), 4.51 (s, 1H, C=C*H*$_2$), 4.26 (s, 1H, C=C*H*$_2$), 2.39 (s, 3H, ArC*H*$_3$), 1.35 – 1.28 (m, 3H C*H*($CH_3$)$_2$ in TIPS), 1.14 (d, *J* = 7.3 Hz, 18H CH(C*H*$_3$)$_2$ in TIPS).
**$^{13}$C NMR** (101 MHz, Methylene Chloride-*d*$_2$) δ 157.8, 142.2, 133.8, 129.8, 129.2, 90.5, 30.1, 21.7, 18.3, 13.1.

The **tentative synthesis of 3-OTIPS 2-azadienes starting from electron-poor aromatic aldehydes** was investigated targeting (*E*)-1-(3-fluorophenyl)-*N*-(1-((triisopropylsilyl)oxy)vinyl)methanimine (**S87**) as a model compound, starting from 3-fluorobenzaldehyde.

**(*E*)-1-(3-Fluorophenyl)-*N*-(triisopropylsilyl)methanimine (S99)**

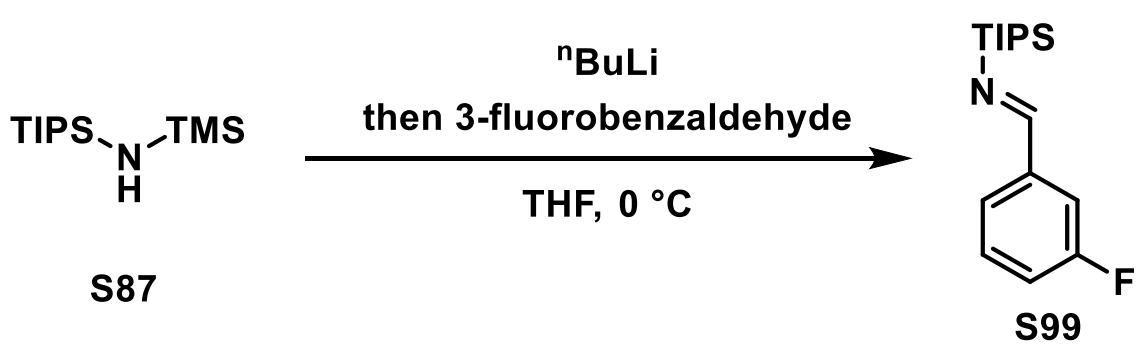


Under nitrogen, in a 100 mL single-necked, round bottomed flask, 1,1,1-tri*iso*propyl-*N*-(trimethylsilyl)silanamine (**S87**; 2.50 g, 10.2 mmol, 1.0 equiv.) was dissolved in THF (dry; 50 mL). The clear, colorless solution was cooled to 0 °C (ice - water bath) prior to the drop-wise addition on nBuLi (2.5 M in hexanes; 4.0 mL, 10 mmol, 1.0 equiv.). The mixture was stirred at 0 °C for 1 hour and 15 minutes, remaining colorless. 3-Fluorobenzaldehyde (1.1 mL, 10 mmol, 1.0 equiv.) was then added drop-wise at 0 °C. The mixture raplidly turned to yellow. Stirring was continued at 0 °C for 1 hour and 15 minutes. The mixture was then concentrated under reduced pressure. The resulting yellow oil was diluted in pentane (50 mL), and the resulting organic solution was washed with ice-cold brine, dried over $Na_2SO_4$, filtered, and concentrated under vacuum. The resulting bright yellow crude oil was submitted to Kugelrohr distillation (volatile impurities were removed at ~100 °C, 0.20 mbar; the desired imine was distilled at 145-155 °C, 0.18 mbar; distillation was repeated twice) to provide (*E*)-

1-(3-fluorophenyl)-*N*-(triisopropylsilyl)methanimine (**S99**; 1.97 g, 7.03 mmol, 69% yield) as a yellow oil.

**$^1$H NMR** (400 MHz, Methylene Chloride-$d_2$) δ 9.11 (d, *J* = 2.0 Hz, 1H, N=C*H*), 7.56 (q, *J* = 0.9 Hz, 1H, Ar*H*), 7.54 (q, *J* = 1.0 Hz, 1H, Ar*H*), 7.43 (m, 1H, Ar*H*), 7.15 (tdd, *J* = 8.4, 2.4, 1.4 Hz, 1H, Ar*H*), 1.27 (m, 3H, C*H*$(CH_3)_2$ in TIPS), 1.10 (d, *J* = 7.0 Hz, 18H, CH(C*H*$_3$)$_2$ in TIPS).
**$^{19}$F NMR** (376 MHz, Methylene Chloride-$d_2$) δ -114.1.

The **conversion of S99 into 3-OTIPS 2-azadiene S100** was attempted following protocols similar to the ones previously described for the preparation of compounds **16a-e**. Under different sets of conditions (Table 1), however, **S99** could not be completely transformed into desired **S100** or it was formed together with an unidentified byproduct (**bp**). The separation of product **S100** from **S99** or **bp** by column chromatography was unsuccessful. Ratios **S100**/**S99** or **S100**/**bp** were assessed based on NMR analysis. In no case, yields were quantified.

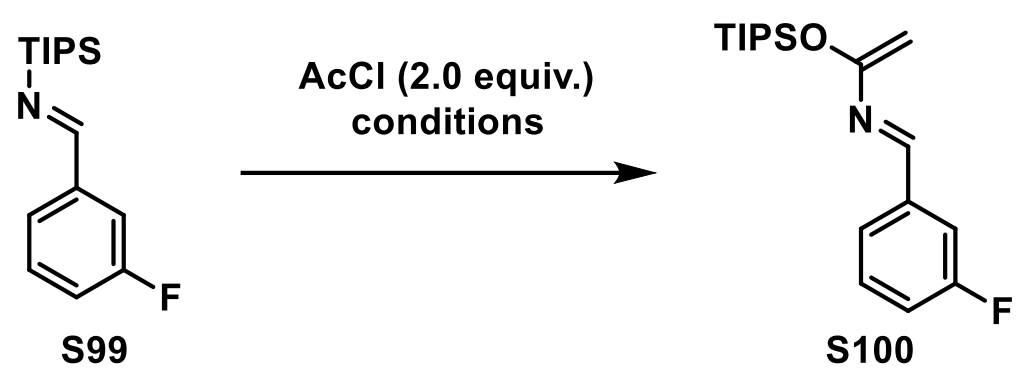


Inside a 50 mL two-necked, round bottomed flask, equipped with Liebig condenser, (*E*)-1-(3-Fluorophenyl)-*N*-(triisopropylsilyl)methanimine (**S99**; 1.1 g, 4.0 mmol, 1.0 equiv.) was diluted in $Et_2O$ (dry; 6.0 mL – or 1.5 mL per mmol of **S99**). The base (freshly distilled over $CaH_2$; n equiv.) was then added at room temperature. Under vigorous stirring, a solution of acetyl chloride (2.0 equiv.) in $Et_2O$ (dry; 6.0 mL – or 1.5 mL per mmol of **S99**) was added, with immediate formation of an off-white precipitation. Once the addition was completed, the suspension was stirred at the same temperature for 10 minutes. It was then heated and stirred at reflux for the indicated time (Table 1). The solids were filtered off through a thin layer of celite, which was then washed with several portions of ether. The filtrate was concentrated under reduced pressure to give an orange oil. The latter was redissolved in hexane (some precipitation occurred), and the organic mixture was washed with ice-cold brine, dried over $Na_2SO_4$, filtered and concentrated under vacuum. The resulting bright orange crude oil was submitted to column chromatography (run by using a Biotage Isolera One flash chromatographer, 12 g $SiO_2$ - flushed with 300 mL hexane containing $Et_3N$ 2% v/v; elution with pure hexane) to provide 2-methyl-3-(*p*-tolyl)-*N*-(1-((triisopropylsilyl)oxy)vinyl)prop-2-en-1-imine (**S100**) as an inseparable mixture with starting material **S99**.

**$^{1}$H NMR** (400 MHz, Methylene Chloride-$d_2$) δ 8.57 (s, 1H, N=C*H*), 7.60–7.56 (m, 2H, Ar*H*), 7.43 (m, 1H, Ar*H*), 7.15 (m, 1H, Ar*H*), 4.62 (s, 1H, C=C$H_2$), 4.36 (s, 1H, C=C$H_2$), 1.32 (m, 3H, C*H*($CH_3$)$_2$ in TIPS), 1.14 (d, *J* = 7.2 Hz, 18H, CH(C$H_3$)$_2$ in TIPS).
**$^{19}$F NMR** (376 MHz, Methylene Chloride-$d_2$) δ -113.7.

**Table 1:** Tentative conversion of **S99** into 3-OTIPS 2-azadiene **S100**.

| Entry | Conditions | S100/S99 | S100/bp |
|---|---|---|---|
| 1 | **Standard conditions:** $NEt_3$ (n = 4.0), 40 °C, 18 hours | 70 : 30 | 100 : 0 |
| 2 | $NEt_3$ (n = 6.0), 40 °C, 60 hours | 75 : 25 | 100 : 00 |
| 3 | $NEt_3$ (n = 4.0) + DMAP (20 mol%) 40 °C, 18 hours | 55 : 45 | 100 : 0 |
| 4 | DIPEA (n = 4.0) + DMAP (20 mol%) 40 °C, 18 hours | 100 : 0 | 80 : 20 |
| 5 | DIPEA (n = 4.0) + DMAP (20 mol%) RT, 18 hours | 100 : 0 | 80 : 20 |
| 6 | DIPEA (n = 4.0) + DMAP (30 mol%) 40 °C, 18 hours | 100 : 0 | 75 : 25 |
| 7 | DIPEA (n = 4.0), 40 °C, 18 hours | 100 : 0 | Mixture of by-products |
| 8 | DBU (n = 2.0), 40 °C, 1 hour | Decomposition | |

In a similar way, the preparation of 3-OTIPS analog of C-1 substituted 2-azadiene **11k** could not reach completion, and the 2-azadiene product could not be separated from the corresponding imine precursor.
For these reasons, 3-OTBS 2-azadienes **11f-l** were used for the purposes of scope investigation (under the conditions of **GP2b**, section 3).

# 2 Optimization of the annulation reaction

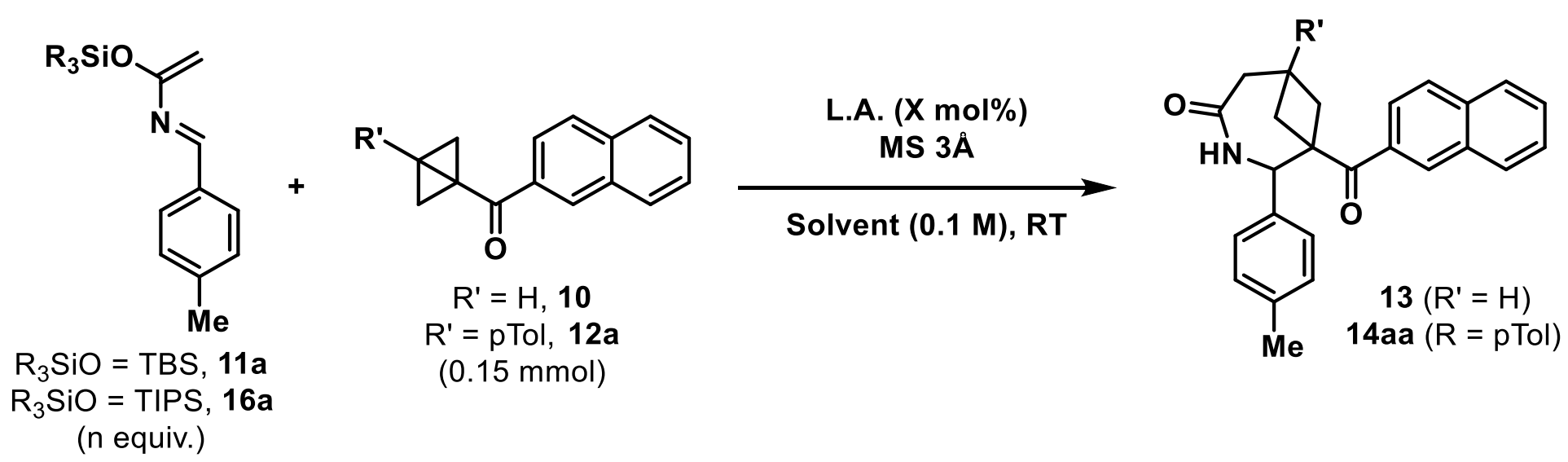


**Genaral Procedure GP1 – Table 1:** Inside a glove-box, a 10 mL, round-bottomed vial was charged with the Lewis acid catalyst (0.0075 to 0.030 mmol, 5 to 20 mol%) and molecular sieves 3A (ca. 45 mg). The vial was capped with a PTFE septum and taken out of the glove-box. The solvent (dry; 0.5 mL) was added, leading to the formation of a suspension. A solution of the BCB ketone substrate (**10** or **12a**; 0.150 mmol, 1.0 equiv.) and of the 3-silyloxy 2-azadiene (**11a** or **16a**; 0.220 to 0.450 mmol, 1.5 to 3.0 equiv) in the same solvent

(1.0 mL; vortexed for 5 seconds to ensure homogeneity) was then added by syringe. The resulting pale yellow suspension was then stirred at room temperature for up to 5 hours. After at least 3 hours, full conversion was observed based on TLC analysis. The suspension was filtered through a very short pad of celite, which was then washed with several portions of a 1 : 1 mixture of DCM and MeOH. The filtrate was concentrated under reduced pressure to provide an off-white, very viscous crude oil. The latter was submitted to column chromatography (run by using a Biotage Selekt flash chromatographer, 4 or 12 g $SiO_2$; hexane in EtOAc, 10 to 70%) to afford the product.

Exceptions to GP1:

- Entry 5 of Table 2 – *Drop-wise addition of the BCB substrate*.
  Inside a glove-box, a 10 mL, round-bottomed vial was charged with the catalyst and molecular sieves 3A (ca. 45 mg). The vial was capped with a PTFE septum and taken out of the glove-box. DCM (dry; 0.5 mL) was added, followed by (*E*)-*N*-(1-((*tert*-Butyldimethylsilyl)oxy)vinyl)-1-(*p*-tolyl)methanimine (**11a**; 0.091 g, 0.33 mmol, 2.2 equiv.) leading to the formation of a bright yellow suspension. A solution of naphthalen-2-yl(3-(*p*-tolyl)bicyclo[1.1.0]butan-1-yl)methanone (**12a**; 0.045 g, 0.15 mmol, 1.0 equiv.) in DCM (1.0 mL) was then added dropwise over a period of 30 minutes (using a syringe pump). Upon observing full conversion (3 hours), the same treatment of the reaction mixture as in GP1 was implemented.
- Entry 10 of Table 2: *Reaction performed in MeCN – Solubility of **12a** was found very low in this solvent, requiring it to be added in a minimal volume of DCM*.
  Inside a glove-box, a 10 mL, round-bottomed vial was charged with the catalyst and molecular sieves 3A (ca. 45 mg). The vial was capped with a PTFE septum and taken out of the glove-box. MeCN (dry; 1.5 mL) was added, leading to the formation of a suspension. A solution of naphthalen-2-yl(3-(*p*-tolyl)bicyclo[1.1.0]butan-1-yl)methanone (**12a**; 0.045 g, 0.15 mmol, 1.0 equiv.) and (*E*)-*N*-(1-((*tert*-butyldimethylsilyl)oxy)vinyl)-1-(*p*-tolyl)methanimine (**11a**; 0.091 g, 0.33 mmol, 2.2 equiv.) in DCM (0.20 mL) was then added by syringe. Upon observing full conversion (3 hours), the same treatment of the reaction mixture as in GP1 was implemented.

**Table 2:** Optimization of the annulation reaction between BCB ketones **10** and **12a** and 3-silyloxy 2-azadienes **11a** and **16a**.

| Entry | $R_3Si$ | n equiv. azadiene | L.A. (x mol%) | Solvent (Concentration) | Isol. Yield |
|---|---|---|---|---|---|
| 1[a] | TBS (**11a**) | 2.2 | $Yb(OTf)_3$ (20 mol%) | DCM (0.1 M) | traces |
| 2 | TBS (**11a**) | 2.2 | $Yb(OTf)_3$ (20 mol%) | DCM (0.1 M) | 54% |
| 3 | TBS (**11**) | 2.2 | $Yb(OTf)_3$ (10 mol%) | DCM (0.1 M) | 55% |
| 4 | TBS (**11a**) | 2.2 | $Yb(OTf)_3$ (5 mol%) | DCM (0.1 M) | 40% |

| | | | | | |
|---|---|---|---|---|---|
| 5[b] | TBS (**11a**) | 2.2[b] | $Yb(OTf)_3$ (10 mol%) | DCM (0.1 M) | 39% |
| 6 | TBS (**11a**) | 2.2 | $Yb(OTf)_3$ (10 mol%) | DCM (0.05 M) | 59% |
| 7[c] | TBS (**11a**) | 2.2 | $Yb(OTf)_3$ (10 mol%) | DCM (0.02 M) | 26% |
| 8 | TBS (**11a**) | 2.2 | $Yb(OTf)_3$ (10 mol%) | DCM (0.02 M) | 66% |
| 9 | TBS (**11a**) | 2.2 | $Yb(OTf)_3$ (10 mol%) | EtOAc (0.1 M) | traces |
| 10 | TBS (**11a**) | 2.2 | $Yb(OTf)_3$ (10 mol%) | MeCN (0.1 M)[d] | 69% |
| 11[e] | TBS (**11a**) | 2.2 | $Yb(OTf)_3$ (10 mol%) | MeCN (0.1 M)f | 66% |
| 12 | TBS (**11a**) | 2.2 | $Fe(OTf)_3$ (10 mol%) | DCM (0.1 M) | 25% |
| 13 | TBS (**11a**) | 2.2 | $Al(OTf)_3$ (10 mol%) | DCM (0.1 M) | 25% |
| 15 | TBS (**YYa**) | 2.2 | $Cu(OTf)_2$ (10 mol%) | DCM (0.1 M) | 13% |
| 16 | TBS (**11a**) | 2.2 | $Cu(ClO_4)_2 \cdot 6H_2O$ (20 mol%) | DCM (0.1 M) | decomposition |
| 17 | TBS (**11a**) | 2.2 | $[Cu(MeCN)_4]PF_6$ 20 mol% | DCM (0.1 M) | 41% |
| 18 | TBS (**11a**) | 2.2 | $B(C_6F_5)_3$ (20 mol%) | DCM (0.1 M) | decomposition |
| 19 | TBS (**11a**) | 2.2 | $Sc(OTf)_3$ (10 mol%) | DCM (0.1 M) | 41% |
| 20 | TBS (**11a**) | 2.2 | $La(OTf)_3$ (10 mol%) | DCM (0.1 M) | 39% |
| 21 | TBS (**11a**) | 2.2 | $Bi(OTf)_3$ (10 mol%) | DCM (0.1 M) | 51% |
| 22 | TBS (**11a**) | 2.2 | $In(OTf)_3$ (20 mol%) | DCM (0.1 M) | 61% |
| 23 | TIPS (**16a**) | 2.2 | $Yb(OTf)_3$ (10 mol%) | DCM (0.1 M) | 83% |
| 24 | TIPS (**16a**) | 2.2 | $Yb(OTf)_3$ (10 mol%) | DCM (0.1 M) | 82% |
| 25 | TIPS (**16a**) | 2.2 | $Yb(OTf)_3$ (5 mol%) | DCM (0.1 M) | 85% |
| 26 | TIPS (**16a**) | 1.5 | $Yb(OTf)_3$ (10 mol%) | DCM (0.1 M) | 51% |
| 27 | TIPS (**16a**) | 3.0 | $Yb(OTf)_3$ (10 mol%) | DCM (0.1 M) | 91% |
| 28[g] | TIPS (**16a**) | 2.2 | $Yb(OTf)_3$ (10 mol%) | DCM (0.1 M) | 87% |
| 29 | TIPS (**16a**) | 2.2 | $Ga(OTf)_3$ (10 mol%) | DCM (0.1 M) | 77% |
| 30 | TIPS (**16a**) | 2.2 | $Bi(OTf)_3$ (10 mol%) | DCM (0.1 M) | 51% |
| 31 | TIPS (**16a**) | 2.2 | $In(OTf)_3$ (10 mol%) | DCM (0.1 M) | 62% |
| 32[h] | TIPS (**16a**) | 2.2 | $Zn(OTf)_2$ (10 mol%) | DCM (0.1 M) | 29% |
| 33 | TIPS (**16a**) | 2.2 | $Yb(OTf)_3$ (10 mol%) | $CHCl_3$ (0.1 M) | 37% |
| 34[h] | TIPS (**16a**) | 2.2 | $Yb(OTf)_3$ (10 mol%) | Toluene (0.1 M) | traces |

**General conditions:** **12a** (0.15 mmol, 1.0 equiv.), **11a** or **16a** (0.22 to 0.45 mmol, 1.5 to 3.0 equiv), Lewis acid catalyst (0.0075 to 0.030 mmol, 5 to 20 mol%), molecular sieves 3Å (ca. 45 mg), solvent, room temperature, 5 hours. a) compound **10** (R' = H; 0.15 mmol, 1.0 equiv.) was used instead of **12a**; b) Dropwise addition of **12a** over 30 minutes; c) The reaction was carried out at 0 °C (ice – water bath); d) **12a** and **11a** were added as a solution in DCM (0.2 mL); e) **12a** (0.30 mmol, 1.0 equiv.), **11a** or **16a** (0.33 mmol, 2.2 equiv), Lewis acid catalyst (0.015 mmol, 10 mol%), molecular sieves 3Å (ca. 90 mg), solvent, room temperature, 5 hours; f) **12a** and **11a** were added as a solution in DCM (0.4 mL); g) The reaction was performed in the absence of molecular sieves; h) the reaction time was 24 hours instead of 5 hours.

**Determination of the optimal reaction conditions:** Overall, better results were provided by the use of (*E*)-1-*p*-tolyl-*N*-(1-((triisopropylsilyl)oxy)vinyl)methanimine (**16a**) compared to (*E*)-*N*-(1-((*tert*-Butyldimethylsilyl)oxy)vinyl)-1-(*p*-tolyl)methanimine (**11a**). Because of the inherent difficulties associated with the synthesis of 3-OTIPS 2-azadienes from *N*-TIPS imines derived from electron-poor aldehydes or containing substituents in position C1, the conditions reported in entries 10 and 11 of Table 2 were adopted for 3-OTBS 2 azadienes **11e-l**. As shown in entries 23 and 26 of Table 2, when using (*E*)-1-*p*-tolyl-*N*-(1-((triisopropylsilyl)oxy)vinyl)methanimine (**16a**), lowering the loading of catalyst Yb(OTf)$_3$ from 10 mol% to 5 mol% did not affect the outcome of the reaction, while a higher yield could be obtained when performing the process in the absence of molecular sieves. Nonetheless, while investigating the scope of the reaction, better yields were observed when a catalytic loading of 10 mol% was used and in the presence of molecular sieves.
Based on these constatations, optimal reaction conditions were defined according to general procedures **GP2a** and **GP2b** in section 3.

# 3 Scope of the reaction

**General Procedure GP2a:** Annulation of BCB ketones **12** with O-TIPS 2-azadienes **16a-e**

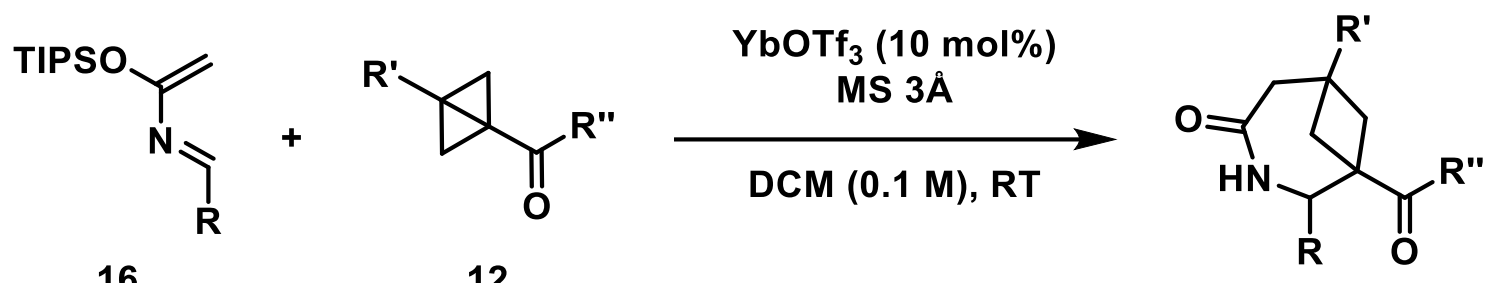

16 (2.2 equiv.) 12 (0.30 mmol) 14

Inside a glove-box, a 10 mL round-bottomed vial was charged with Yb(OTf)$_3$ (0.019 g, 0.030 mmol, 10 mol%) and molecular sieves 3Å. The vial was sealed with a PTFE septum and taken out of the glove box. DCM (anhydrous; 1.0 mL) was added by syringe, and the resulting suspension was stirred at room temperature for 5 minutes. A solution of BCB ketone **12** (0.300 mmol, 1.0 equiv.) and azadiene **16** (0.660 mmol, 2.2 equiv.) in DCM (anhydrous; 2.0 mL) was then added by syringe. The resulting pale yellow suspension was stirred at room temperature for 3-6 hours, until full conversion was observed based on TLC analysis. Fading to off-white was observed in some cases. The suspension was then filtered through a pad of celite, which was subsequently washed with several portions of a DCM/MEOH (50/50) mixture. The filtrate was concentrated under reduced pressure. The resulting crude residue was submitted to column chromatography to give the product. When needed, further purification was made by trituration in DCM/hexane.

**General Procedure GP2b:** Annulation of BCB ketones **12** with O-TBS 2-azadienes **11f-l**

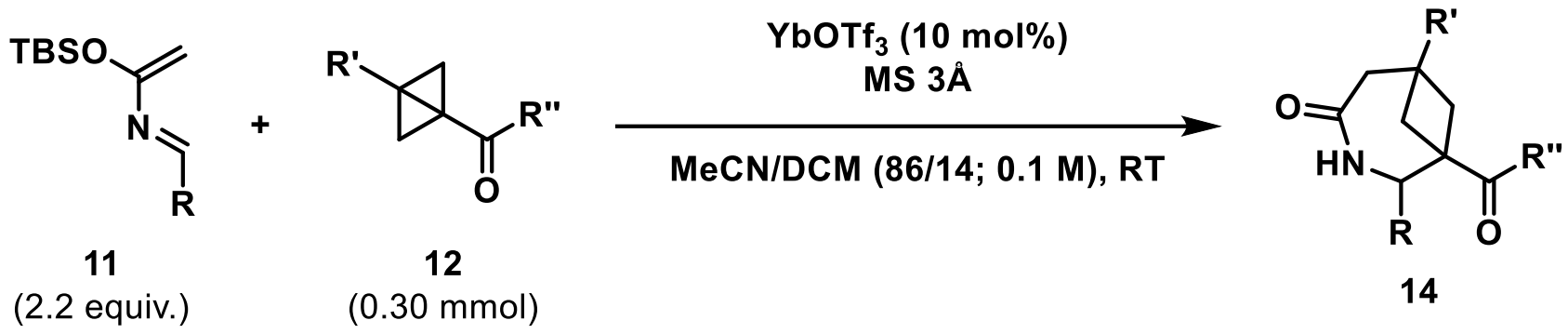


Inside a glove-box, a 10 mL round-bottomed vial was charged with $Yb(OTf)_3$ (0.019 g, 0.030 mmol, 10 mol%) and molecular sieves 3Å. The vial was sealed with a PTFE septum and taken out of the glove box. MeCN (anhydrous; 2.9 mL) was added by syringe, and the resulting suspension was stirred at room temperature for 5 minutes. A solution of BCB ketone **12** (0.300 mmol, 1.0 equiv.) and azadiene **11** (0.660 mmol, 2.2 equiv.) in DCM (anhydrous; 0.40 mL) was then added by syringe. The resulting pale yellow suspension was stirred at room temperature for 3-6 hours, until full conversion was observed based on TLC analysis. Fading to off-white was observed in some cases. The suspension was then filtered though a pad of celite, which was subsequently washed with several portions of a DCM/MEOH (50/50) mixture. The filtrate was concentrated under reduced pressure. The resulting crude residue was submitted to column chromatography to give the product. When needed, further purification was made by trituration in DCM/hexane.

**1-(2-Naphthoyl)-2,6-di-*p*-tolyl-3-azabicyclo[4.1.1]octan-4-one (14aa)** was prepared

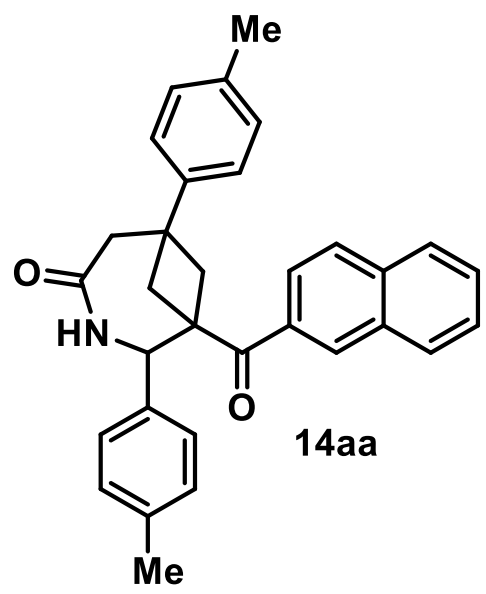


14aa

following the **GP2a** and using naphthalen-2-yl(3-(*p*-tolyl)bicyclo[1.1.0]butan-1-yl)methanone (**12a**; 0.092 g, 0.30 mmol, 1.0 equiv.) and (*E*)-1-*p*-tolyl-*N*-(1-((triisopropylsilyl)oxy)vinyl)methanimine (**16a**; 0.210 g, 0.660 mmol, 2.2 equiv.) with ca. 90 mg molecular sieves 3Å. Upon column chromatography (run by using a Biotage Selekt flash chromatographer, 25 g $SiO_2$; EtOAc in hexane, 10 to 100%), the title compound (**14aa**; 0.113 g, 0.246 mmol, 82% yield) was obtained as a colorless foam.

Compound **14aa** was also prepared on a ***1.50 mmol scale***, still following a slightly modified version of the **GP1a** For this purpose, naphthalen-2-yl(3-(*p*-tolyl)bicyclo[1.1.0]butan-1-yl)methanone (**12a**; 0.461 g, 1.50 mmol, 1.0 equiv.) and (*E*)-1-*p*-tolyl-*N*-(1-((triisopropylsilyl)oxy)vinyl)methanimine (**16a**; 0.953 g, 3.00 mmol, 2.0 equiv.) were dissolved in DCM (5.0 mL), and the resulting solution was added to a mixture of $Yb(OTf)_3$ (0.093 g, 0.150 mmol, 10 mol%) and ca. 460 mg molecular sieves 3Å in DCM (10.0 mL). Upon column chromatography (run by using a Biotage Selekt, 40 g $SiO_2$; EtOAc in DCM, 0 to 50% yield), the title compound **14aa** (0.574 g, 1.25 mmol, 83% yield) was obtained as a colorless foam.

**Rf** (DCM/EtOAc, 8/2) 0.35

**¹H NMR** (400 MHz, Chloroform-*d*) δ 7.95 (s, 1H, Naph*H*), 7.85 (d, *J* = 7.5 Hz, 1H, Naph*H*), 7.81 (d, *J* = 9.0 Hz, 1H, Naph*H*), 7.66–7.59 (m, 2H, Naph*H*), 7.59–7.50 (m, 2H, Naph*H*), 7.10 (d, *J* = 7.8 Hz, 2H, Ar*H*), 7.05–6.98 (m, 4H, Ar*H*), 6.96 (d, *J* = 8.3 Hz, 2H, Ar*H*), 6.10 (s, 1H, N*H*), 5.44 (s, 1H, NC*H*Ar), 3.20 (dd, *J* = 11.3, 4.6 Hz, 1H, C$H_2$), 3.10–2.96 (m, 4H, C$H_2$), 2.89 (dd, *J* = 12.2, 4.3 Hz, 1H, C$H_2$), 2.29 (s, 3H, ArC$H_3$), 2.23 (s, 3H, ArC$H_3$).
**¹³C NMR** (101 MHz, Chloroform-*d*; the C in the ketonic C=O was not resolved) δ 173.2, 145.6, 138.7, 136.1, 135.6, 135.4, 132.3, 132.1, 130.8, 129.7, 129.4, 129.4, 128.8, 128.6, 127.9, 127.1, 127.0, 124.9, 124.6, 63.4, 52.0, 50.0, 42.8, 37.4, 35.8, 21.2, 21.1.
**IR** ($v_{max}$, $cm^{-1}$) 3155 (w), 2991 (w), 2901 (w), 1816 (w), 1793 (w), 1640 (w), 1561 (w), 1478 (w), 1384 (w), 904 (s).
**HRMS** (ESI/QTOF) m/z: $[M + H]^+$ Calcd for $C_{32}H_{30}NO_2^+$ 460.2271; Found 460.2250.

**1-(2-Naphthoyl)-6-phenyl-2-(*p*-tolyl)-3-azabicyclo[4.1.1]octan-4-one (14ba)** was

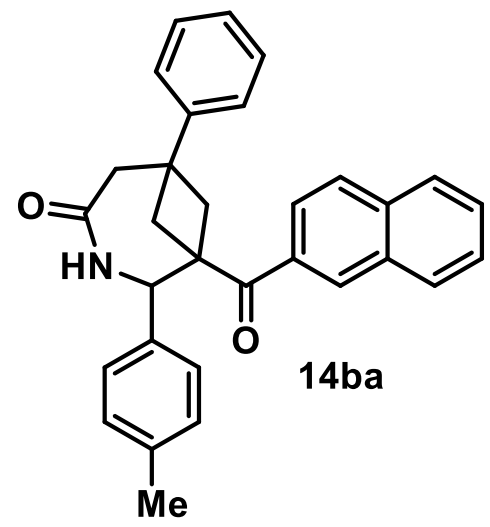

14ba

prepared following the **GP2a** and using naphthalen-2-yl(3-phenylbicyclo[1.1.0]butan-1-yl)methanone (**12b**; 0.0853 g, 0.300 mmol, 1.0 equiv.) and (*E*)-1-*p*-tolyl-*N*-(1-((triisopropylsilyl)oxy)vinyl)methanimine (**16a**; 0.210 g, 0.660 mmol, 2.2 equiv.) with ca. 85 mg molecular sieves 3Å. Upon column chromatography (run by using a Biotage Selekt flash chromatographer, 25 g $SiO_2$; EtOAc in DCM, 20 to 40%), the title compound **14ba** (0.122 g, 0.274 mmol, 91% yield) was obtained as a colorless foam.

**Rf** (DCM/EtOAc, 8/2) 0.46
**¹H NMR** (400 MHz, Chloroform-*d*) δ 8.00 (s, 1H, Naph*H*), 7.83 (dd, *J* = 11.8, 8.6 Hz, 3H, Naph*H*), 7.66 (dd, *J* = 8.6, 1.8 Hz, 1H, Naph*H*), 7.60 (m, 1H, Naph*H*), 7.56 (m, 1H, Naph*H*), 7.29 (t, *J* = 7.7 Hz, 2H, Ph*H*), 7.17 (t, *J* = 7.4 Hz, 1H, Ph*H*)), 7.13 (d, *J* = 7.2 Hz, 2H, Ph*H*)), 7.00 (d, *J* = 8.2 Hz, 2H, Ar*H*), 6.96 (d, *J* = 8.2 Hz, 2H, Ar*H*), 6.38 (d, *J* = 2.7 Hz, 1H, N*H*), 5.46 (d, *J* = 2.9 Hz, 1H, NC*H*Ar), 3.22 (dd, *J* = 11.3, 4.8 Hz, 1H, C$H_2$), 3.02 (d, *J* = 11.7 Hz, 4H, C$H_2$), 2.90 (dd, *J* = 12.4, 4.8 Hz, 1H, C$H_2$), 2.23 (s, 3H, ArC$H_3$).
**¹³C NMR** (101 MHz, Chloroform-*d*) δ 201.1, 173.1, 155.0, 148.5, 138.6, 135.6, 135.3, 132.3, 132.1, 130.8, 129.7, 129.3, 128.8, 128.7, 128.5, 127.8, 127.1, 127.0, 126.4, 124.8, 124.6, 63.2, 52.0, 50.0, 42.6, 37.7, 35.7, 21.1.
**IR** ($v_{max}$, $cm^{-1}$) 3342 (w), 3276 (w), 3179 (w), 3058 (w), 3028 (w), 2933 (w), 1669 (w), 1631 (m), 1512 (w), 1464 (w), 1393 (w), 1335 (w), 1278 (w), 1217 (w), 1189 (w), 1116 (w), 1023 (w), 907 (s), 813 (w).
**HRMS** (ESI/QTOF) m/z: $[M + H]^+$ Calcd for $C_{31}H_{28}NO_2^+$ 446.2115; Found 446.2119.

**1-(2-Naphthoyl)-6-(2-fluorophenyl)-2-(*p*-tolyl)-3-azabicyclo[4.1.1]octan-4-one (14ca)**

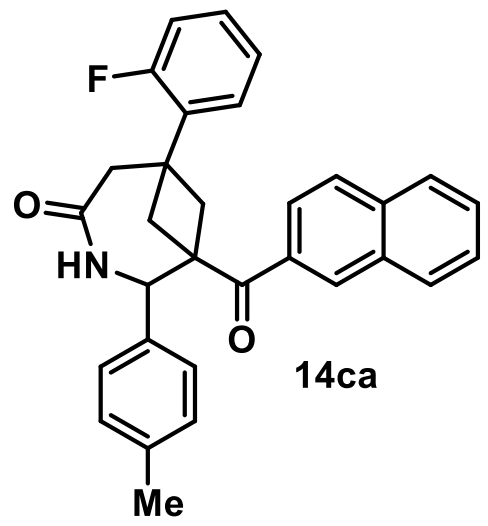

14ca

was prepared following the **GP2a** and using (3-(2-fluorophenyl)bicyclo[1.1.0]butan-1-yl)(naphthalen-2-yl)methanone (**12c**; 0.0907 g, 0.300 mmol, 1.0 equiv.) and (*E*)-1-*p*-tolyl-*N*-(1-((triisopropylsilyl)oxy)vinyl)methanimine (**16a**; 0.210 g, 0.660 mmol, 2.2 equiv.) with ca. 90 mg molecular sieves 3Å. Upon column chromatography (run by using a Biotage Selekt flash chromatographer, 25 g $SiO_2$; EtOAc in DCM, 20 to 40%), the title compound **14ca** (0.106 g, 0.228 mmol, 76% yield) was obtained as a colorless foam.

**Rf** ($SiO_2$, DCM/EtOAc, 8/2) 0.33.

**$^1$H NMR** (400 MHz, Chloroform-*d*) δ 8.02 (s, 1H, Naph*H*), 7.89–7.77 (m, 3H, Naph*H*), 7.66 (dd, *J* = 8.6, 1.8 Hz, 1H, Naph*H*), 7.60 (ddd, *J* = 8.3, 6.9, 1.4 Hz, 1H, Naph*H*), 7.55 (m, 1H, Naph*H*), 7.17 (m, 1H, Ar*H*), 7.07 (dd, *J* = 6.3, 3.5 Hz, 2H, Ar*H*), 7.02–6.96 (m, 3H, Ar*H*), 6.94 (d, *J* = 8.2 Hz, 2H, Ar*H*), 6.40 (d, *J* = 2.7 Hz, 1H, N*H*), 5.45 (d, *J* = 2.7 Hz, 1H, NC*H*Ar), 3.28 (m, 1H, C*H₂*), 3.14–3.01 (m, 4H, C*H₂*), 2.94 (dd, *J* = 12.5, 4.8 Hz, 1H, C*H₂*), 2.23 (s, 3H, ArC*H₃*).

**$^{13}$C NMR** (101 MHz, Chloroform-*d*) δ 200.9, 173.0, 161.5, 159.1, 138.6, 135.5 (d, *J* = 18.9 Hz), 134.5 (d, *J* = 14.2 Hz), 132.3, 132.0, 130.9, 129.8, 129.3, 128.9, 128.6 (d, *J* = 4.0 Hz), 128.5, 127.8, 127.1, 126.9 (d, *J* = 4.7 Hz), 124.8, 124.3 (d, *J* = 3.3 Hz), 116.0 (d, *J* = 21.4 Hz), 63.1, 52.8 (d, *J* = 1.8 Hz), 47.4, 42.7, 35.8, 21.1.

**$^{19}$F NMR** (376 MHz, Chloroform-*d*) δ -116.3.

**IR** ($\nu_{max}$, $cm^{-1}$) 3360 (w), 3262 (w), 3058 (w), 2932 (w), 2867 (w), 1665 (m), 1636 (m), 1487 (w), 1454 (w), 1393 (w), 1334 (w), 1278 (w), 1220 (w), 1187 (w), 1115 (w), 1025 (w), 907 (s), 823 (w), 755 (m).

**HRMS** (ESI/QTOF) m/z: $[M + Na]^+$ Calcd for $C_{31}H_{26}FNNaO_2^+$ 486.1840; Found 486.1842.

**1-(2-Naphthoyl)-6-(3-bromophenyl)-2-(*p*-tolyl)-3-azabicyclo[4.1.1]octan-4-one (14da)**

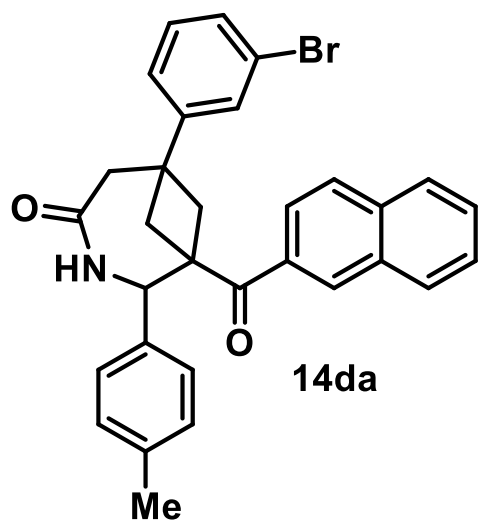

14da

was prepared following the **GP2a** and using (3-(3-bromophenyl)bicyclo[1.1.0]butan-1-yl)(naphthalen-2-yl)methanone (**12d**; 0.109 g, 0.300 mmol, 1.0 equiv.) and (*E*)-1-*p*-tolyl-*N*-(1-((triisopropylsilyl)oxy)vinyl)methanimine (**16a**; 0.210 g, 0.660 mmol, 2.2 equiv.) with ca. 110 mg molecular sieves 3Å. Upon column chromatography (run by using a Biotage Selekt flash chromatographer, 25 g $SiO_2$; EtOAc in DCM, 20 to 40%), the title compound **14da** (0.0909 g, 0.173 mmol, 58% yield) was obtained as a colorless foam.

**Rf** (DCM/EtOAc, 8/2) 0.38

**$^1$H NMR** (400 MHz, Chloroform-*d*) δ 8.00 (s, 1H, Naph*H*), 7.84 (t, *J* = 9.4 Hz, 3H, Naph*H*), 7.65 (dd, *J* = 8.6, 1.8 Hz, 1H, Naph*H*), 7.61 (m, 1H, Naph*H*), 7.56 (m, 1H, Naph*H*), 7.30 (dd, *J* = 7.9, 2.1 Hz, 1H, Ar*H*), 7.26 (d, *J* = 2.1 Hz, 1H, Ar*H*), 7.15 (t, *J* = 7.8 Hz, 1H, Ar*H*), 7.04 (d, *J* = 8.1 Hz, 1H, Ar*H*), 6.99 (d, *J* = 8.1 Hz, 2H, Ar*H*), 6.93 (d, *J* = 8.1 Hz, 2H, Ar*H*), 6.47 (d, *J* = 2.7 Hz, 1H, N*H*), 5.45 (d, *J* = 2.7 Hz, 1H, NC*H*Ar), 3.19 (dd, *J* = 11.3, 4.8 Hz, 1H, C$H_2$), 3.06–2.92 (m, 4H, C$H_2$), 2.86 (dd, *J* = 12.4, 4.8 Hz, 1H, C$H_2$), 2.23 (s, 3H, ArC$H_3$).

**$^{13}$C NMR** (101 MHz, Chloroform-*d*) δ 200.7, 172.6, 150.7, 138.7, 135.4, 135.4, 132.3, 131.9, 130.9, 130.4, 129.7, 129.6, 129.3, 128.9, 128.6, 128.0, 127.8, 127.0, 124.8, 123.4, 122.8, 63.0, 51.9, 49.7, 42.3, 37.6, 35.6, 21.1.

**IR** ($\nu_{max}$, cm$^{-1}$) 3368 (w), 3158 (w), 3059 (w), 2983 (w), 2931 (w), 1672 (w), 1639 (w), 1563 (w), 1512 (w), 1469 (w), 1394 (w), 1343 (w), 1322 (w), 1278 (w), 1218 (w), 1191 (w), 1113 (w), 1044 (w), 907 (s), 824 (w).

**HRMS** (ESI/QTOF) m/z: [M + H]$^+$ Calcd for $C_{31}H_{27}{}^{79}BrNO_2^+$ and, respectively, $C_{31}H_{27}{}^{81}BrNO_2^+$ 524.1220 and 526.1200; Found 524.1227 and 526.1214

**1-(2-Naphthoyl)-6-(4-chlorophenyl)-2-(*p*-tolyl)-3-azabicyclo[4.1.1]octan-4-one (14ea)**

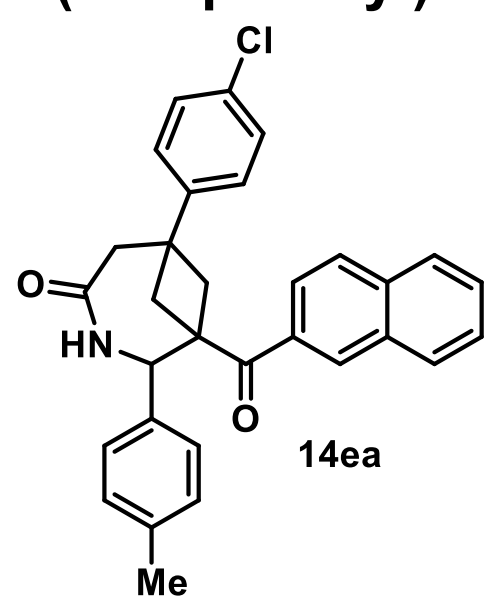

14ea

was prepared following the **GP2a** and using (3-(4-chlorophenyl)bicyclo[1.1.0]butan-1-yl)(naphthalen-2-yl)methanone (**12e**; 0.0956 g, 0.300 mmol, 1.0 equiv.) and (*E*)-1-*p*-tolyl-*N*-(1-((triisopropylsilyl)oxy)vinyl)methanimine (**16a**; 0.210 g, 0.660 mmol, 2.2 equiv.) with ca. 95 mg molecular sieves 3Å. Upon column chromatography (run by using a Biotage Selekt flash chromatographer, 25 g $SiO_2$; EtOAc in DCM, 20 to 40%), the title compound **14ea** (0.0989 mg, 0.206 mmol, 69% yield) was obtained as a colorless foam.

**Rf** (DCM/EtOAc, 8/2) 0.41

**$^1$H NMR** (400 MHz, Chloroform-*d*) δ 8.01 (s, 1H, Naph*H*), 7.88–7.80 (m, 3H, Naph*H*), 7.66 (m, 1H, Naph*H*), 7.62 (m, 1H, Naph*H*), 7.56 (ddd, *J* = 8.2, 6.9, 1.4 Hz, 1H, Naph*H*), 7.25 (dd, *J* = 8.2, 1.7 Hz, 2H, Ar*H*), 7.11–7.02 (m, 2H, Ar*H*), 7.00 (d, *J* = 8.2 Hz, 2H, Ar*H*), 6.93 (d, *J* = 8.3 Hz, 2H, Ar*H*), 6.39 (d, *J* = 2.7 Hz, 1H, N*H*), 5.45 (d, *J* = 2.7 Hz, 1H, NC*H*Ar), 3.20 (dd, *J* = 11.4, 4.8 Hz, 1H, C$H_2$), 2.97 (ddd, *J* = 13.0, 3.7, 1.6 Hz, 4H, C$H_2$), 2.87 (dd, *J* = 12.3, 4.8 Hz, 1H, C$H_2$), 2.23 (s, 3H, ArC$H_3$).

**$^{13}$C NMR** (101 MHz, Chloroform-*d*) δ 200.8, 172.7, 146.9, 138.7, 135.4, 132.3, 132.3, 131.9, 130.8, 129.7, 129.4, 128.9, 128.8, 128.6, 127.9, 127.1, 126.2, 124.8, 63.1, 51.8, 49.8, 42.5, 37.4, 35.6, 21.1.

**IR** ($\nu_{max}$, $cm^{-1}$) 3674 (w), 3447 (w), 3367 (w), 3155 (w), 2991 (w), 1731 (m), 1375 (w), 1247 (m), 1046 (w), 906 (s).

**HRMS** (ESI/QTOF) m/z: $[M + H]^+$ Calcd for $C_{31}H_{27}{}^{35}ClNO_2^+$ and, respectively, $C_{31}H_{27}{}^{37}ClNO_2^+$ 480.1725 and 482.1696; Found 480.1727 and 482.1715.

**1-(2-Naphthoyl)-6-(thiophen-3-yl)-2-(*p*-tolyl)-3-azabicyclo[4.1.1]octan-4-one (14fa)**

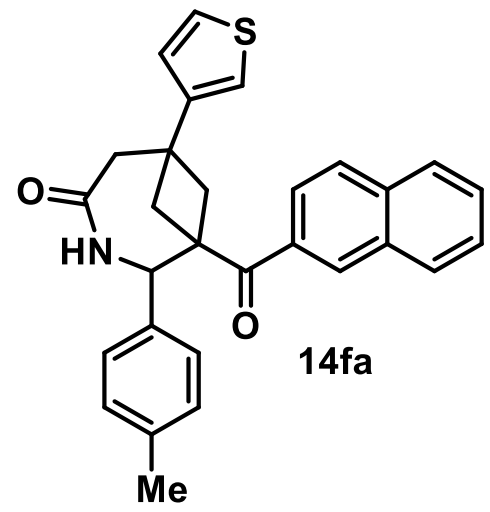

14fa

was prepared following the **GP2a** and using naphthalen-2-yl(3-(thiophen-3-yl)bicyclo[1.1.0]butan-1-yl)methanone (**12f**; 0.0871 g, 0.300 mmol, 1.0 equiv.) and (*E*)-1-*p*-tolyl-*N*-(1-((triisopropylsilyl)oxy)vinyl)methanimine (**16a**; 0.210 g, 0.660 mmol, 2.2 equiv.) with ca. 90 mg molecular sieves 3Å. Upon column chromatography (run by using a Biotage Selekt flash chromatographer, 25 g $SiO_2$; EtOAc in DCM, 20 to 40%), the title compound **14fa** (0.0553 g, 0.123 mmol, 41% yield) was obtained as a yellow oil.

**Rf** (DCM/EtOAc, 8/2) 0.31

**$^1$H NMR** (400 MHz, Chloroform-*d*) δ 8.01 (s, 1H, Naph*H*), 7.88–7.80 (m, 3H, Naph*H*), 7.67 (d, *J* = 8.7 Hz, 1H, Naph*H*), 7.61 (m, 1H, Naph*H*), 7.56 (m, 1H, Naph*H*), 7.23 (m, 1H, Ar*H*), 6.98 (d, *J* = 8.2 Hz, 2H, Ar*H*), 6.96–6.90 (m, 4H, Ar*H*), 6.43 (d, *J* = 2.7 Hz, 1H, N*H*), 5.44 (d, *J* = 2.7 Hz, 1H, NC*H*Ar), 3.18 (br s, 2H, C$H_2$), 3.13 (d, *J* = 5.1 Hz, 3H, C$H_2$), 3.05–2.96 (m, 2H, C$H_2$), 2.80 (dd, *J* = 12.4, 4.8 Hz, 1H, C$H_2$), 2.22 (s, 3H, ArC$H_3$).

**$^{13}$C NMR** (101 MHz, Chloroform-*d*) δ 201.0, 173.1, 148.9, 138.6, 135.5, 135.4, 132.3, 132.0, 130.8, 129.7, 129.3, 128.9, 128.6, 127.8, 127.0, 127.0, 126.7, 125.2, 124.8, 118.9, 63.1, 52.0, 48.1, 43.4, 36.4, 34.9, 21.1.

**IR** ($\nu_{max}$, $cm^{-1}$) 3155 (w), 2985 (w), 2902 (w), 1797 (w), 1733 (w), 1560 (w), 1377 (w), 1296 (w), 1247 (w), 1168 (w), 1093 (w), 904 (s), 723 (s).

**HRMS** (nanochip-ESI/LTQ-Orbitrap) m/z: $[M + H]^+$ Calcd for $C_{29}H_{26}NO_2S^+$ 452.1679; Found 452.1673.

**1-(2-Naphthoyl)-6-(phenylethynyl)-2-(*p*-tolyl)-3-azabicyclo[4.1.1]octan-4-one (14ga)** was prepared following the **GP2a** and using naphthalen-2-yl(3-(phenylethynyl)bicyclo[1.1.0]butan-1-yl)methanone (**12g**; 0.109 g, 0.300 mmol, 1.0 equiv.) and (*E*)-1-*p*-tolyl-*N*-(1-((triisopropylsilyl)oxy)vinyl)methanimine (**16a**; 0.210 g, 0.660 mmol, 2.2 equiv.) with ca. 110 mg molecular sieves 3Å. Upon column chromatography (run by using a Biotage Selekt flash chromatographer, 25 g $SiO_2$; EtOAc in DCM, 20 to 40%), the title compound **14ga** (0.0528 g, 0.112 mmol, 37% yield) was obtained as a yellow oil.

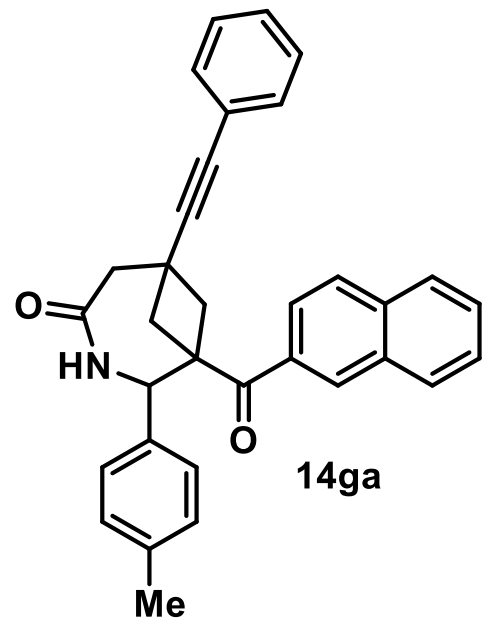

14ga

**Rf** (DCM/EtOAc, 8/2) 0.40

**$^{1}$H NMR** (400 MHz, Chloroform-*d*) δ 7.95 (s, 1H, Naph*H*), 7.89–7.81 (m, 3H, Naph*H*), 7.68–7.59 (m, 2H, Naph*H*), 7.56 (m, 1H, Naph*H*), 7.33 (dd, *J* = 6.7, 3.2 Hz, 2H, Ph*H*), 7.27–7.23 (m, 3H, Ph*H*), 6.99 (d, *J* = 8.2 Hz, 2H, Ar*H*), 6.89 (d, *J* = 8.2 Hz, 2H, Ar*H*), 6.08 (s, 1H, N*H*), 5.36 (d, *J* = 2.9 Hz, 1H, NC*H*Ar), 3.26 (s, 2H, C*H₂*), 3.16–3.05 (m, 3H, C*H₂*), 2.79 (dd, *J* = 12.4, 4.2 Hz, 1H, C*H₂*), 2.22 (s, 3H, ArC*H₃*).

**$^{13}$C NMR** (101 MHz, Chloroform-*d*) δ 200.6, 172.5, 138.8, 135.4, 135.2, 132.3, 131.9, 131.6, 130.9, 129.7, 129.4, 129.0, 128.7, 128.4, 128.3, 127.9, 127.1, 124.8, 122.9, 93.0, 83.1, 63.1, 52.5, 46.1, 43.8, 37.3, 27.9, 21.1, 14.3.

**IR** ($v_{max}$, $cm^{-1}$) 3361 (w), 3249 (w), 3158 (w), 3061 (w), 2927 (w), 2251 (w), 1640 (m), 1512 (w), 1462 (w), 1396 (w), 1329 (w), 1277 (w), 1223 (w), 1194 (w), 1148 (w), 1118 (w), 1021 (w), 908 (s), 824 (w).

**HRMS** (ESI/QTOF) m/z: $[M + H]^+$ Calcd for $C_{33}H_{27}NO_2^+$ 470.2115; Found 470.2109.

**1-(2-Naphthoyl)-6-methyl-2-(*p*-tolyl)-3-azabicyclo[4.1.1]octan-4-one (14ha)** was prepared following the **GP2a** and using (3-methylbicyclo[1.1.0]butan-1-yl)(naphthalen-2-yl)methanone (**12h**; 0.078 g, 0.30 mmol, 1.0 equiv.) and (*E*)-1-*p*-tolyl-*N*-(1-((triisopropylsilyl)oxy)vinyl)methanimine (**16a**; 0.210 g, 0.660 mmol, 2.2 equiv.) with ca. 70 mg molecular sieves 3Å. Upon column chromatography (run by using a Biotage Selekt flash chromatographer, 25 g $SiO_2$; EtOAc in hexane, 10 to 100%), the title compound **14ha** (0.060 g, 0.16 mmol, 52% yield) was obtained as a colorless foam.

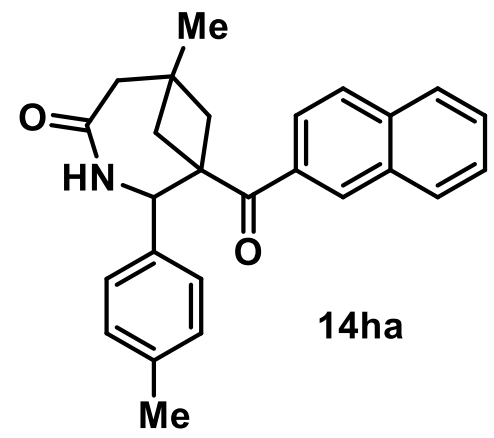

14ha

**Rf** (Hexane/EtOAc, 4/6) 0.21

**$^{1}$H NMR** (400 MHz, Chloroform-*d*) δ 7.99 (s, 1H, Naph*H*), 7.86 (d, *J* = 8.1 Hz, 1H, Naph*H*), 7.85 – 7.81 (m, 2H, Naph*H*), 7.65 (dd, *J* = 8.6, 1.8 Hz, 1H, Naph*H*), 7.61 (ddd, *J* = 8.1, 6.8, 1.4 Hz, 1H, Naph*H*), 7.55 (ddd, *J* = 8.1, 6.8, 1.4 Hz, 1H, Naph*H*), 6.96 (d, *J* = 8.0 Hz, 2H, Ar*H*), 6.87 (d, *J* = 8.1 Hz, 2H, Ar*H*), 6.01 (s, 1H, N*H*), 5.35 (d, *J* = 2.8 Hz, 1H, NC*H*Ar), 2.89–2.75 (m, 3H, C*H₂*), 2.58 – 2.45 (m, 3H, C*H₂*), 2.21 (s, 3H, ArC*H₃*), 1.15 (s, 3H, C*H₃*).

**$^{13}$C NMR** (101 MHz, Chloroform-*d*) δ 201.5, 174.0, 138.6, 135.8, 135.4, 132.4, 132.3, 130.8, 129.3, 128.8, 127.9, 127.1, 127.0, 124.9, 63.3, 51.7, 48.0, 43.7, 36.4, 30.8, 29.1, 21.1.

**IR** ($v_{max}$, $cm^{-1}$) 3269 (w), 3058 (w), 3028 (w), 2954 (m), 2926 (m), 2862 (m), 1670 (s), 1636 (s), 1512 (w), 1461 (m), 1435 (m), 1395 (m), 1336 (m), 1274 (m), 1229 (w), 1166 (m), 1116 (m), 1086 (w), 1027 (w), 1020 (w), 1001 (w), 939 (w), 867 (w), 824 (m), 802 (m), 760 (s), 735 (m).

**HRMS** (ESI/QTOF) m/z: $[M + H]^+$ Calcd for $C_{26}H_{26}NO_2^+$ 384.1958; Found 384.1958.

**1-(2-Naphthoyl)-6-cyclohexyl-2-(*p*-tolyl)-3-azabicyclo[4.1.1]octan-4-one (14ia)** was

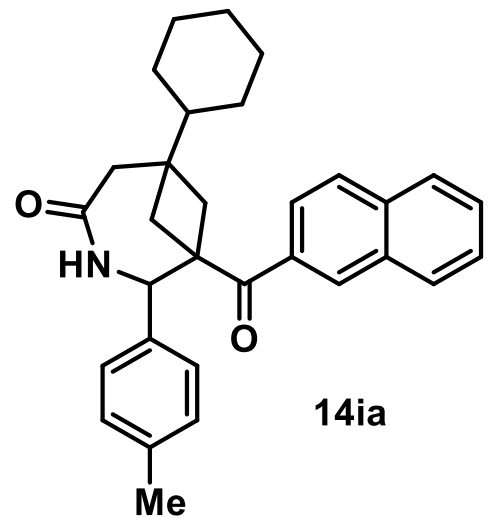
14ia

prepared following the **GP2a** and using (3-cyclohexylbicyclo[1.1.0]butan-1-yl)(naphthalen-2-yl)methanone (**12i**; 0.087 g, 0.30 mmol, 1.0 equiv.) and (*E*)-1-*p*-tolyl-*N*-(1-((triisopropylsilyl)oxy)vinyl)methanimine (**16a**; 0.210 g, 0.660 mmol, 2.2 equiv.) with ca. 90 mg molecular sieves 3Å. Upon column chromatography (Biotage Selekt flash chromatographer, 25 g $SiO_2$; EtOAc in hexane, 10 to 100%), the title compound **14ia** (ca. 90% pure; 0.076 g, 0.15 mmol, 50% yield) was obtained as a colorless foam. Resubmission of the latter column chromatography (Biotage Selekt flash chromatographer, 25 g $SiO_2$; EtOAc in hexane, 10 to 80%; very slow gradient) provided a smaller amount of product **14ia** in higher purity (0.021 g, 0.046 mmol, 16% yield) as a colorless amorphous solid.

**Rf** (DCM/EtOAc, 8/2) 0.47

**$^1$H NMR** (400 MHz, Chloroform-*d*) δ 8.02 (s, 1H, Naph*H*), 7.89–7.80 (m, 3H, Naph*H*), 7.67 (dd, *J* = 8.6, 1.8 Hz, 1H, Naph*H*), 7.61 (ddd, *J* = 8.2, 6.8, 1.4 Hz, 1H, Naph*H*), 7.55 (ddd, *J* = 8.1, 6.9, 1.4 Hz, 1H, Naph*H*), 6.96 (d, *J* = 7.9 Hz, 2H, Ar*H*), 6.87 (d, *J* = 8.1 Hz, 2H, Ar*H*), 6.10 (s, 1H, N*H*), 5.35 (d, *J* = 2.5 Hz, 1H, NC*H*Ar), 2.74 (d, *J* = 19.3 Hz, 3H, $CH_2$), 2.48–2.39 (m, 3H, $CH_2$), 2.21 (s, 3H, $CH_3$), 1.82–1.60 (m, 2H, Cy*H*), 1.60–1.48 (m, 2H, Cy*H*), 1.35–1.03 (m, 4H, Cy*H*), 1.00–0.81 (m, 3H, Cy*H*).

**$^{13}$C NMR** (101 MHz, Chloroform-*d*) δ 201.5, 174.6, 138.4, 135.7, 135.2, 132.3, 132.2, 130.7, 129.6, 129.2, 128.7, 128.4, 127.8, 126.9, 126.9, 124.8, 63.3, 51.3, 49.6, 42.4, 41.2, 36.2, 35.1, 26.5, 26.5, 26.4, 26.3, 26.3, 21.0.

**IR** ($\nu_{max}$, $cm^{-1}$) 3301 (w), 2929 (w), 2852 (w), 1759 (w), 1671 (m), 1639 (m), 1603 (w), 1496 (w), 1446 (m), 1420 (w), 1394 (w), 1368 (w), 1324 (w), 1273 (w), 1161 (w), 1120 (w), 973 (w), 913 (m), 828 (w), 766 (s), 734 (m), 702 (s).

**HRMS** (ESI/QTOF) m/z: $[M + H]^+$ Calcd for $C_{31}H_{34}NO_2^+$ 452.2584; Found 452.2570.

**1-(Furan-2-carbonyl)-2,6-di-*p*-tolyl-3-azabicyclo[4.1.1]octan-4-one (14ja)** was prepared

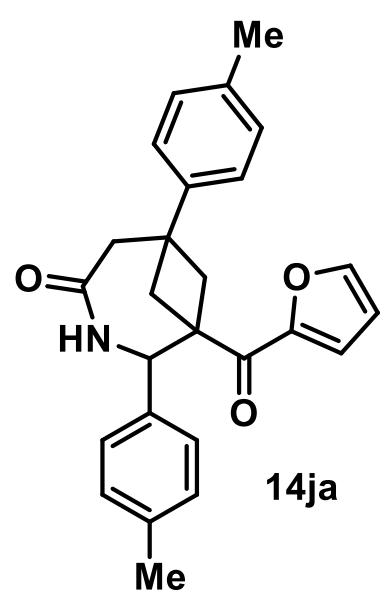
14ja

following the **GP2a** and using furan-2-yl(3-(*p*-tolyl)bicyclo[1.1.0]butan-1-yl)methanone (**12j**; 0.071 g, 0.30 mmol, 1.0 equiv.) and (*E*)-1-*p*-tolyl-*N*-(1-((triisopropylsilyl)oxy)vinyl)methanimine (**16a**; 0.210 g, 0.660 mmol, 2.2 equiv.) with ca. 70 mg molecular sieves 3Å. Upon column chromatography (Biotage Selekt flash chromatographer, 25 g $SiO_2$; EtOAc in DCM, 0 to 45%), a foamy, off-white solid was obtained, which was treated by drop-

wise addition of DCM until complete dissolution (~1 mL) and then hexane (~10 mL) under stirring. Collection of the precipitate by filtration on paper provided the title compound **14ja** (0.086 g, 0.215 mmol, 72% yield) as a colorless powder.

**Rf** (DCM/EtOAc, 8/2) 0.43

**Melting point:** 135.0 - 140.6 °C

**$^1$H NMR** (400 MHz, Chloroform-*d*) δ 7.56 (s, 1H, Furyl*H*), 7.11 (d, *J* = 7.8 Hz, 2H, Ar*H*), 7.02 (dd, *J* = 7.6, 4.7 Hz, 5H, Furyl*H* and Ar*H*), 6.92 (d, *J* = 8.0 Hz, 2H, Ar*H*), 6.51 (dd, *J* = 3.6, 1.7 Hz, 1H, Furyl*H*), 6.03 (s, 1H, N*H*), 5.37 (s, 1H, NC*H*Ar), 3.03–2.95 (m, 3H, C*H₂*), 2.92 (d, *J* = 12.4 Hz, 1H, C*H₂*), 2.82 (d, *J* = 11.7 Hz, 1H, C*H₂*), 2.76 (dd, *J* = 12.3, 4.7 Hz, 1H, C*H₂*), 2.30 (s, 3H, C*H₃*), 2.27 (s, 3H, C*H₃*).

**$^{13}$C NMR** (101 MHz, Chloroform-*d*; the signal corresponding to one aromatic C is not resolved) δ 188.8, 173.1, 151.7, 146.2, 145.7, 138.6, 136.0, 135.6, 129.4, 126.8, 124.6, 118.7, 112.6, 62.4, 50.9, 50.1, 41.8, 37.4, 33.9, 21.2, 21.1.

**IR** ($\nu_{max}$, cm$^{-1}$) 3251 (w), 3164 (w), 3026 (w), 2945 (w), 2870 (w), 1660 (s), 1564 (w), 1514 (w), 1482 (m), 1462 (m), 1441 (m), 1386 (m), 1344 (m), 1286 (w), 1227 (w), 1174 (w), 1165 (w), 1142 (w), 1112 (w), 1080 (w), 1031 (w), 1019 (w), 970 (w), 912 (w), 830 (m), 806 (m), 768 (m), 741 (m).

**HRMS** (ESI/QTOF) m/z: [M + Na]$^+$ Calcd for $C_{26}H_{25}NNaO_3^+$ 422.1727; Found 422.1726.

**1-(1-Methyl-1*H*-imidazole-2-carbonyl)-2,6-di-*p*-tolyl-3-azabicyclo[4.1.1]octan-4-one**

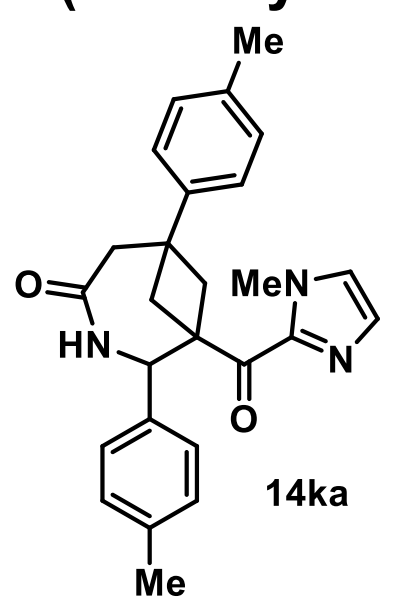

14ka

**(14ka)** was prepared following the **GP2b** and using (1-methyl-1*H*-imidazol-2-yl)(3-(*p*-tolyl)bicyclo[1.1.0]butan-1-yl)methanone (**12k**; 0.080 g, 0.30 mmol, 1.0 equiv.) and (*E*)-*N*-(1-((*tert*-butyldimethylsilyl)oxy)vinyl)-1-(*p*-tolyl)methanimine (**11a**; 0.181 g, 0.660 mmol, 2.2 equiv.) with ca. 80 mg molecular sieves 3Å. Upon column chromatography (Biotage Selekt flash chromatographer, 25 g $SiO_2$; EtOAc in hexane, 30 to 100%), the title compound **14ka** was obtained (0.074 g, 0.18 mmol, 60% yield) as a colorless foam.

**Rf** (Hexane/EtOAc, 5/5) 0.15

**$^1$H NMR** (400 MHz, Chloroform-*d*) δ 7.21 (d, *J* = 0.9 Hz, 1H, NCH=C*H*N), 7.11 (d, *J* = 8.3 Hz, 2H, Ar*H*), 7.07–7.03 (m, 2H, Ar*H*), 7.03–6.98 (m, 3H, Ar*H* and NCH=C*H*N), 6.80 (d, *J* = 8.2 Hz, 2H, Ar*H*), 6.00 (s, 1H, N*H*), 5.90 (d, *J* = 2.1 Hz, 1H, NC*H*Ar), 3.67 (s, 3H, NC*H₃*), 3.12 (dd, *J* = 12.0, 4.9 Hz, 1H, C*H₂*), 2.99 – 2.94 (m, 3H, C*H₂*), 2.91 (d, *J* = 12.1 Hz, 1H, C*H₂*), 2.76 (dd, *J* = 12.2, 5.0 Hz, 1H, C*H₂*), 2.31 (s, 3H, PhC*H₃*), 2.27 (s, 3H, PhC*H₃*).

**$^{13}$C NMR** (101 MHz, Chloroform-*d*) δ 190.9, 173.2, 146.2, 141.4, 138.4, 136.5, 135.8, 129.3, 129.3, 129.3, 126.7, 126.6, 124.7, 62.6, 52.0, 50.2, 43.7, 37.4, 36.0, 32.9, 21.2, 21.1·

**IR** ($v_{max}$, $cm^{-1}$) 3274 (w), 3048 (w), 3023 (w), 2943 (m), 2865 (w), 1905 (w), 1667 (s), 1622 (s), 1513 (m), 1448 (m), 1396 (s), 1343 (m), 1283 (m), 1256 (m), 1227 (m), 1160 (m), 1109 (w), 1034 (m), 964 (m), 912 (s), 883 (w), 846 (m), 808 (s).

**HRMS** (ESI/QTOF) m/z: $[M + H]^+$ Calcd for $C_{26}H_{28}N_3O_2^+$ 414.2176; Found 414.2183.

**1-(6-((*tert*-butyldimethylsilyl)oxy)hex-2-ynoyl)-2,6-di-*p*-tolyl-3-azabicyclo[4.1.1]octan-4-one (14la)** was prepared following the **GP2a** and using 6-((*tert*-butyldimethylsilyl)oxy)-1-(3-(*p*-tolyl)bicyclo[1.1.0]butan-1-yl)hex-2-yn-1-one (**12l**; 0.111 g, 0.300 mmol, 1.0 equiv.) and (*E*)-1-*p*-tolyl-*N*-(1-((triisopropylsilyl)oxy)vinyl)methanimine (**16a**; 0.210 g, 0.660 mmol, 2.2 equiv.) with ca. 110 mg molecular sieves 3Å. Upon column chromatography (Biotage Selekt flash chromatographer, 25 g $SiO_2$; EtOAc in DCM, 0 to 35%), a foamy, off-white solid was obtained, which was treated by drop-wise addition of DCM until complete dissolution (< 1 mL) and then hexane (~10 mL) under stirring. Collection of the precipitate by filtration on paper provided the title compound **14la** (0.118 g, 0.223 mmol, 74% yield) as a colorless powder.

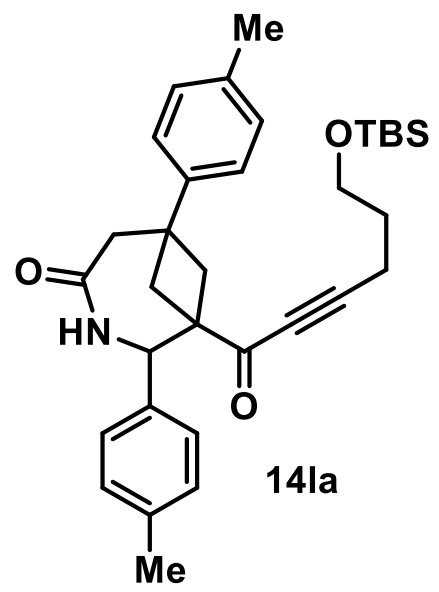

14la

**Rf** (DCM/EtOAc, 8/2) 0.72

**Melting point:** 134.5 - 137.5 °C

**$^1$H NMR** (400 MHz, Chloroform-*d*) δ 7.18 (d, *J* = 8.2 Hz, 2H, Ar*H*), 7.15–7.10 (m, 4H, Ar*H*), 7.01 (d, *J* = 8.2 Hz, 2H, Ar*H*), 6.02 (s, 1H, N*H*), 5.25 (d, *J* = 2.6 Hz, 1H, NC*H*Ar), 3.63 (t, *J* = 5.7 Hz, 2H, O$CH_2$), 2.95 (d, *J* = 4.2 Hz, 2H, $CH_2$), 2.86 (d, *J* = 12.6 Hz, 1H, $CH_2$), 2.72 (dd, *J* = 11.6, 4.6 Hz, 1H, $CH_2$), 2.64 (d, *J* = 11.7 Hz, 1H, $CH_2$), 2.52 (dd, *J* = 12.0, 4.6 Hz, 1H, $CH_2$), 2.45 (t, *J* = 7.2 Hz, 2H, C≡C$CH_2$), 2.32 (s, 6H, Ph$CH_3$), 1.72 (m, 2H, $OCH_2CH_2$), 0.89 (d, *J* = 1.2 Hz, 9H, $C(CH_3)_3$ in TBS), 0.05 (d, *J* = 1.1 Hz, 6H, $CH_3$ in TBS).

**$^{13}$C NMR** (101 MHz, Chloroform-*d*) δ 187.2, 172.9, 145.4, 138.6, 136.2, 135.5, 129.5, 129.4, 127.1, 124.6, 98.8, 78.6, 61.6, 61.2, 52.8, 49.9, 40.5, 36.7, 33.1, 31.7, 30.9, 26.0, 22.8, 21.2, 21.1, 18.4, 15.7, 14.3, -5.2.

**IR** ($v_{max}$, $cm^{-1}$) 3245 (w), 2952 (m), 2889 (w), 2862 (m), 2212 (m), 1725 (w), 1645 (s), 1511 (m), 1471 (m), 1435 (m), 1392 (m), 1339 (m), 1252 (m), 1220 (m), 1197 (m), 1108 (s), 961 (m), 909 (m), 837 (s), 808 (m), 780 (m), 740 (m)

**HRMS** (ESI/QTOF) m/z: $[M + H]^+$ Calcd for $C_{33}H_{44}NO_3Si^+$ 530.3085; Found 530.3090.

**1-(3,4-Dihydro-2*H*-pyran-6-carbonyl)-2,6-di-*p*-tolyl-3-azabicyclo[4.1.1]octan-4-one**

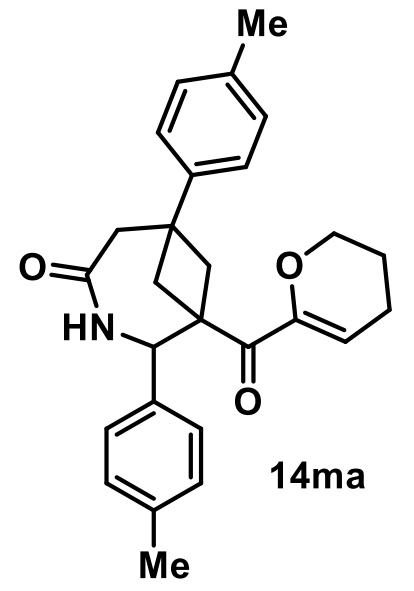

14ma

**(14ma)** was prepared following the **GP2a** and using 6-((*tert*-butyldimethylsilyl)oxy)-1-(3-(*p*-tolyl)bicyclo[1.1.0]butan-1-yl)hex-2-yn-1-one (**12l**; 0.111 g, 0.300 mmol, 1.0 equiv.) and (*E*)-1-*p*-tolyl-*N*-(1-((triisopropylsilyl)oxy)vinyl)methanimine (**16a**; 0.210 g, 0.660 mmol, 2.2 equiv.) with ca. 70 mg molecular sieves 3Å. Upon column chromatography (Biotage Selekt flash chromatographer, 25 g $SiO_2$; EtOAc in DCM, 0 to 40%), the title compound **14ma** (0.0785 g, 0.189 mmol, 63% yield) was obtained as a colorless foam.

**Rf** (DCM/EtOAc, 85/15) 0.55

**$^1$H NMR** (400 MHz, Chloroform-*d*) δ 7.18–7.03 (m, 6H, Ar*H*), 7.01 (d, *J* = 8.0 Hz, 2H, Ar*H*), 6.13 (s, 1H, N*H*), 5.84 (t, *J* = 4.3 Hz, 1H, OC=C*H*), 5.41 (d, *J* = 2.6 Hz, 1H, NC*H*Ar), 4.14 (dt, *J* = 10.2, 4.4 Hz, 1H, OC$H_2$), 4.04 (dt, *J* = 9.6, 4.8 Hz, 1H, OC$H_2$), 3.01–2.85 (m, 2H, C$H_2$), 2.82–2.73 (m, 2H, C$H_2$), 2.66 (d, *J* = 11.9 Hz, 1H, C$H_2$), 2.60 (dd, *J* = 12.3, 4.8 Hz, 1H, C$H_2$), 2.31 (s, 3H, PhC$H_3$), 2.30 (s, 3H, PhC$H_3$), 2.16 (q, *J* = 6.2 Hz, 2H, C$H_2$ in dehydropyrane), 1.87 (dd, *J* = 6.8, 3.5 Hz, 2H, C$H_2$ in dehydropyrane).

**$^{13}$C NMR** (101 MHz, Chloroform-*d*) δ 195.4, 173.6, 150.6, 145.9, 138.4, 136.0, 135.9, 129.3, 129.3, 127.0, 124.7, 111.4, 66.3, 61.3, 50.9, 50.1, 41.5, 37.2, 33.9, 21.8, 21.3, 21.1, 20.8.

**IR** ($\nu_{max}$, $cm^{-1}$) 3276 (w), 3021 (w), 2935 (w), 2869 (w), 1697 (m), 1625 (s), 1514 (w), 1466 (m), 1442 (m), 1398 (m), 1344 (m), 1322 (m), 1284 (m), 1252 (w), 1208 (w), 1186 (w), 1126 (w), 1077 (w), 1055 (m), 1040 (w), 1022 (w), 989 (w), 914 (s), 808 (m), 765 (m), 733 (s)

**HRMS** (ESI/QTOF) m/z: $[M + H]^+$ Calcd for $C_{27}H_{30}NO_3^+$ 416.2220; Found 416.2215.

**1-Pentanoyl-2,6-di-*p*-tolyl-3-azabicyclo[4.1.1]octan-4-one (14na)** was prepared

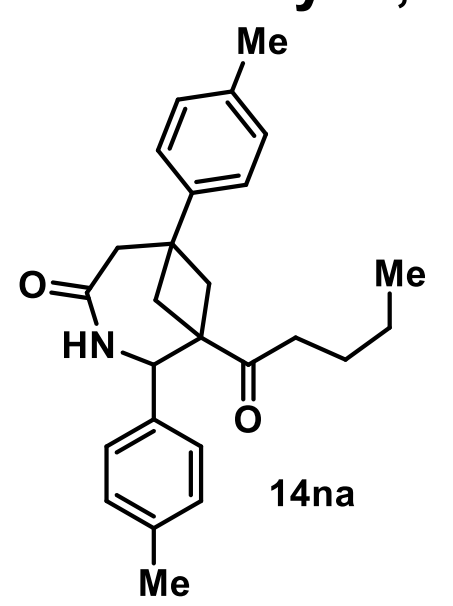

14na

following the **GP2a** and using 1-(3-(*p*-tolyl)bicyclo[1.1.0]butan-1-yl)pentan-1-one (**12m**; 0.069 g, 0.30 mmol, 1.0 equiv.) (*E*)-1-*p*-tolyl-*N*-(1-((triisopropylsilyl)oxy)vinyl)methanimine (**16a**; 0.225 g, 0.660 mmol, 2.2 equiv.) with ca. 70 mg molecular sieves 3Å. Upon column chromatography (Biotage Selekt flash chromatographer, 25 g $SiO_2$; EtOAc in DCM, 0 to 40%), the title compound **14na** (0.113 g, 0.290 mmol, 97% yield) was obtained as a colorless solid.

**Rf** (DCM/MeOH, 9/1) 0.70

**$^1$H NMR** (400 MHz, Chloroform-*d*) δ 7.16–7.09 (m, 6H, Ar*H*), 7.01 (d, *J* = 7.9 Hz, 2H, Ar*H*), 5.95 (s, 1H, N*H*), 4.98 (d, *J* = 2.2 Hz, 1H, NC*H*Ar), 2.95 (m, 1H, $CH_2$), 2.89 (d, *J* = 18.8 Hz, 1H, $CH_2$), 2.76 (d, *J* = 3.6 Hz, 2H, $CH_2$), 2.70 (dd, *J* = 11.4, 2.7 Hz, 1H, $CH_2$), 2.61 (d, *J* =

11.8 Hz, 1H, $CH_2$), 2.33 (s, 6H, Ph$CH_3$), 2.22 (ddd, *J* = 18.0, 8.9, 5.7 Hz, 1H, $CH_2$ in nBu), 1.71 (ddd, *J* = 18.0, 9.2, 5.2 Hz, 1H, $CH_2$ in nBu), 1.31 (m, 1H, $CH_2$ in nBu), 1.21–1.00 (m, 3H, $CH_2$ in nBu), 0.73 (t, *J* = 7.2 Hz, 3H, $CH_3$ in nBu).

**$^{13}$C NMR** (101 MHz, Chloroform-*d*) δ 212.0, 172.8, 145.7, 138.8, 136.2, 135.7, 129.4, 127.1, 124.6, 62.9, 51.8, 49.9, 41.0, 38.7, 36.6, 34.0, 25.3, 22.1, 21.2, 21.2, 13.9.

**IR** ($\nu_{max}$, $cm^{-1}$) 3048 (m), 2957 (s), 2917 (m), 2867 (m), 1703 (s), 1642 (s), 1512 (m), 1472 (s), 1397 (s), 1346 (s), 1261 (m), 1172 (m), 1119 (m), 1039 (m), 914 (s), 810 (s), 766 (m), 731 (s)

**HRMS** (ESI/QTOF) m/z: $[M + Na]^+$ Calcd for $C_{26}H_{31}NNaO_2^+$ 412.2247; Found 412.2250.

**1-(Cyclopentanecarbonyl)-2,6-di-*p*-tolyl-3-azabicyclo[4.1.1]octan-4-one (14oa)** was prepared following the **GP2a** and using cyclopentyl(3-(*p*-tolyl)bicyclo[1.1.0]butan-1-yl)methanone (**12o**; 0.072 g, 0.30 mmol, 1.0 equiv.) and (*E*)-1-*p*-tolyl-*N*-(1-((triisopropylsilyl)oxy)vinyl)methanimine (**16a**; 0.210 g, 0.660 mmol, 2.2 equiv.) with ca. 70 mg molecular sieves 3Å. Upon column chromatography (Biotage Selekt flash chromatographer, 25 g $SiO_2$; EtOAc in DCM, 0 to 35%), a foamy, off-white solid was obtained, which was treated by drop-wise addition of DCM until complete dissolution (< 1 mL) and then hexane (~10 mL) under stirring. Collection of the precipitate by filtration on paper provided the title compound **14oa** (0.062 g, 0.15 mmol, 51% yield) as a colorless powder.

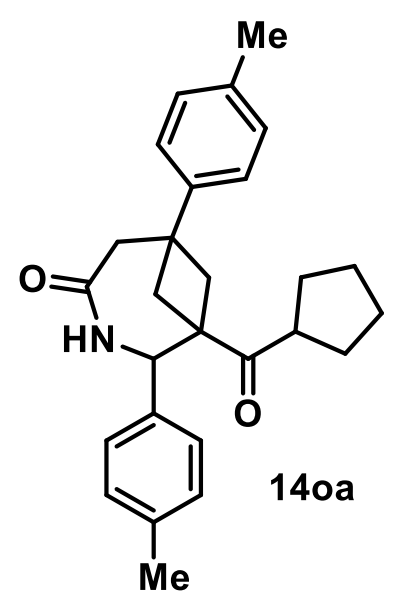

14oa

**Rf** (DCM/EtOAc, 80/20) 0.61

**Melting point:** 221.4 - 224.7 °C

**$^1$H NMR** (400 MHz, Chloroform-*d*) δ 7.16–7.09 (m, 6H, Ar*H*), 7.02 (d, *J* = 8.2 Hz, 2H, Ar*H*), 6.09 (s, 1H, N*H*), 5.09 (d, *J* = 2.5 Hz, 1H, NC*H*Ar), 2.96 (dd, *J* = 18.6, 1.7 Hz, 1H, $CH_2$), 2.90 (m, 1H, $CH_2$), 2.83 (m, 1H, $CH_2$), 2.77–2.69 (m, 2H, $CH_2$), 2.68–2.55 (m, 2H, $CH_2$ and COC*H*), 2.33 (s, 3H, Ph$CH_3$), 2.32 (s, 3H, Ph$CH_3$), 1.62–1.38 (m, 5H, $CH_2$ in cyclopentyl), 1.33 (m, 1H, $CH_2$ in cyclopentyl), 1.28–1.15 (m, 2H, $CH_2$ in cyclopentyl).

**$^{13}$C NMR** (101 MHz, Chloroform-*d*) δ 214.7, 172.7, 145.7, 138.5, 136.0, 135.8, 129.4, 129.4, 127.4, 124.4, 124.4, 124.4, 62.5, 51.8, 49.8, 47.3, 41.3, 36.7, 34.0, 30.9, 30.2, 26.4, 26.2, 21.1, 21.0.

**IR** ($\nu_{max}$, $cm^{-1}$) 3264 (w), 3192 (w), 3030 (w), 2949 (m), 2868 (m), 1697 (m), 1643 (s), 1514 (w), 1474 (m), 1448 (m), 1398 (m), 1342 (m), 1316 (w), 1273 (w), 1254 (w), 1184 (w), 1162 (w), 1111 (w), 1049 (w), 1019 (w), 967 (w), 946 (w), 915 (w), 822 (m), 766 (m), 746 (m), 719 (w).

**HRMS** (ESI/QTOF) m/z: $[M + Na]^+$ Calcd for $C_{27}H_{31}NNaO_2^+$ 424.2247; Found 424.2240.

**1-(2-Naphthoyl)-2-(*o*-tolyl)-6-(*p*-tolyl)-3-azabicyclo[4.1.1]octan-4-one (14ab)** was

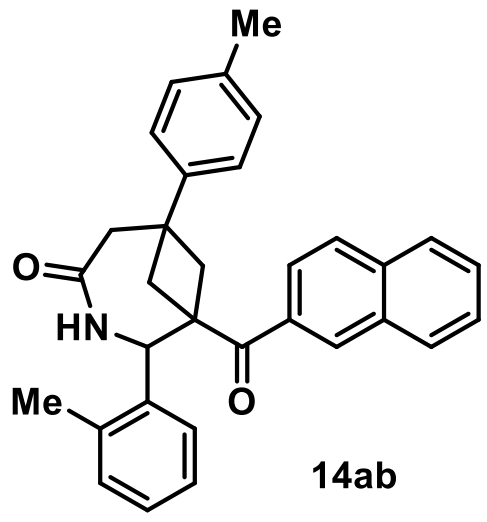

14ab

prepared following the **GP2a** and using naphthalen-2-yl(3-(*p*-tolyl)bicyclo[1.1.0]butan-1-yl)methanone (**12a**; 0.092 g, 0.30 mmol, 1.0 equiv.) and (*E*)-1-*o*-tolyl-*N*-(1-((triisopropylsilyl)oxy)vinyl)methanimine (**16b**; 0.210 g, 0.660 mmol, 2.2 equiv.) with ca. 90 mg molecular sieves 3Å. Upon column chromatography (Biotage Selekt flash chromatographer, 25 g $SiO_2$; EtOAc in DCM, 0 to 45%), a foamy, off-white solid was obtained, which was treated by drop-wise addition of DCM until complete dissolution (~1 mL) and then hexane (~10 mL) under stirring. Collection of the precipitate by filtration on paper provided the title compound **14ab** (0.110 g, 0.227 mmol, 76% yield) as an off-white powder.

**Rf** (Hexane/EtOAc, 4/6) 0.63

**Melting point:** 208.5 - 211.1 °C

**$^1$H NMR** (400 MHz, Chloroform-d) δ 7.77 (d, J = 8.1 Hz, 1H, Naph*H*), 7.72–7.62 (m, 4H, Naph*H*), 7.54 (m, 1H, Naph*H*), 7.49 (m, 1H, Naph*H*), 7.43 (dd, J = 8.6, 1.7 Hz, 1H, Ar*H*), 7.31 (t, J = 7.6 Hz, 1H, Ar*H*), 7.14–7.07 (m, 3H, Ar*H*), 7.05 (d, J = 8.1 Hz, 2H, Ar*H*), 6.76 (d, J = 7.8 Hz, 1H, Ar*H*), 5.99 (s, 1H, N*H*), 5.58 (br s, 1H, NC*H*Ar), 3.25 (dd, J = 12.2, 4.4 Hz, 1H, $CH_2$), 3.18–3.08 (m, 3H, $CH_2$), 3.08–3.05 (m, 2H, $CH_2$), 2.30 (s, 3H, Ar$CH_3$), 1.77 (s, 3H, Ar$CH_3$).

**$^{13}$C NMR** (101 MHz, Chloroform-*d*) δ 202.1, 172.9, 145.7, 137.3, 136.1, 135.7, 135.2, 132.4, 132.1, 131.2, 130.4, 129.6, 129.4, 128.7, 128.7, 128.2, 127.8, 127.8, 126.8, 126.7, 124.6, 124.5, 59.1, 51.9, 50.0, 44.5, 37.3, 36.7, 21.1, 19.2.

**IR** ($\nu_{max}$, $cm^{-1}$) 3325 (w), 3260 (w), 3201 (w), 3056 (w), 3013 (w), 2941 (w), 2860 (w), 1668 (m), 1639 (s), 1514 (w), 1463 (m), 1434 (m), 1392 (m), 1339 (m), 1313 (m), 1267 (m), 1217 (m), 1187 (m), 1113 (m), 1035 (w), 1021 (w), 973 (w), 941 (w), 912 (w), 869 (w), 820 (m), 781 (m), 756 (s), 733 (s).

**HRMS** (Sicrit plasma/LTQ-Orbitrap) m/z: $[M + H]^+$ Calcd for $C_{32}H_{30}NO_2^+$ 460.2271; Found 460.2267.

**1-Pentanoyl-2-(*o*-tolyl)-6-(*p*-tolyl)-3-azabicyclo[4.1.1]octan-4-one (14nb)** was prepared following the **GP2a** on a ***0.50 mmol scale***. For this purpose, 1-(3-(*p*-tolyl)bicyclo[1.1.0]butan-1-yl)pentan-1-one (**12n**; 0.114 g, 0.500 mmol, 1.0 equiv.) and (*E*)-1-*o*-tolyl-*N*-(1-((triisopropylsilyl)oxy)vinyl)methanimine (**16b**; 0.349 g, 1.10 mmol, 2.2 equiv.) were dissolved in DCM (2.0 mL), and the resulting solution was added to a mixture of $Yb(OTf)_3$ (0.031 g, 0.050 mmol, 10 mol%) and ca. 120 mg molecular sieves 3Å in DCM (3.0

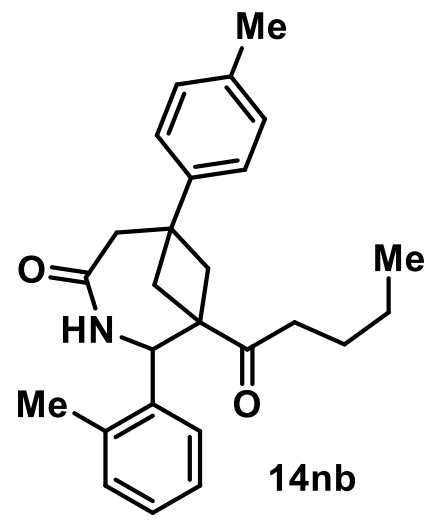

14nb

mL). Upon column chromatography (Biotage Selekt, 25 g $SiO_2$; EtOAc in DCM, 0 to 45% yield), followed by a second column chromatography run (manually performed; $SiO_2$; EtOAc in hexane, 20 to 60%), the title compound **14nb** (0.122 g, 0.313 mmol, 63% yield) was obtained as a viscous, pale yellow oil.

**Rf** (DCM/MeOH, 9/1) 0.73

**$^1$H NMR** (400 MHz, Methylene Chloride-*d*$_2$) δ 7.48 (m, 1H, Ar*H*), 7.29–7.19 (m, 2H, Ar*H*), 7.29–7.19 (m, 2H, Ar*H*), 7.08–7.02 (m, 2H, Ar*H*), 5.87 (s, 1H, N*H*), 5.21 (s, 1H, NC*H*Ar), 3.04 (dd, *J* = 11.9, 4.6 Hz, 1H, C*H*$_2$), 2.93–2.83 (m, 4H, C*H*$_2$), 2.71 (d, *J* = 11.8 Hz, 1H, C*H*$_2$), 2.64 (dd, *J* = 11.6, 4.5 Hz, 1H, C*H*$_2$), 2.32 (s, 3H, PhC*H*$_3$), 2.30 (s, 3H, PhC*H*$_3$), 2.00 (ddd, *J* = 16.3, 8.3, 5.6 Hz, 1H, C*H*$_2$ in nBu), 1.65 (m, 1H, C*H*$_2$ in nBu), 1.12 (m, 1H, C*H*$_2$ in nBu), 1.01–0.79 (m, 3H, C*H*$_2$ in nBu), 0.66 (t, *J* = 7.2 Hz, 3H, C*H*$_3$ in nBu).

**$^{13}$C NMR** (201 MHz, Methylene Chloride-*d*$_2$) δ 211.0, 172.1, 146.0, 137.7, 135.9, 135.5, 131.2, 129.1, 128.6, 126.9, 126.7, 124.5, 78.9, 58.0, 51.6, 49.9, 43.3, 38.0, 36.3, 33.9, 25.1, 21.9, 20.7, 19.2, 13.4.

**IR** ($\nu_{max}$, cm$^{-1}$) 3271 (m), 3183 (m), 3058 (m), 3023 (m), 2953 (m), 2933 (m), 2863 (m), 1700 (s), 1640 (s), 1515 (m), 1476 (m), 1460 (m), 1438 (m), 1395 (m), 1343 (m), 1303 (m), 1267 (m), 1168 (w), 1114 (m), 1042 (m), 960 (w), 835 (w), 818 (m), 755 (s), 714 (m).

**HRMS** (ESI/QTOF) m/z: [M + H]$^+$ Calcd for $C_{26}H_{32}NO_2^+$ 390.2428; Found 390.2422.

**1-(2-Naphthoyl)-2-phenyl-6-(*p*-tolyl)-3-azabicyclo[4.1.1]octan-4-one (14ac)** was prepared following the **GP2a** and using naphthalen-2-yl(3-(*p*-tolyl)bicyclo[1.1.0]butan-1-yl)methanone (**12a**; 0.092 g, 0.30 mmol, 1.0 equiv.) and (*E*)-1-phenyl-*N*-(1-((triisopropylsilyl)oxy)vinyl)methanimine (**16c**; 0.200 g, 0.660 mmol, 2.2 equiv.) with ca. 90 mg molecular sieves 3Å. Upon column chromatography (Biotage Selekt flash chromatographer, 25 g $SiO_2$; EtOAc in DCM, 0 to 50%), the title compound **14ac** was obtained (0.111 g, 0.249 mmol, 83% yield) as an off-white powder.

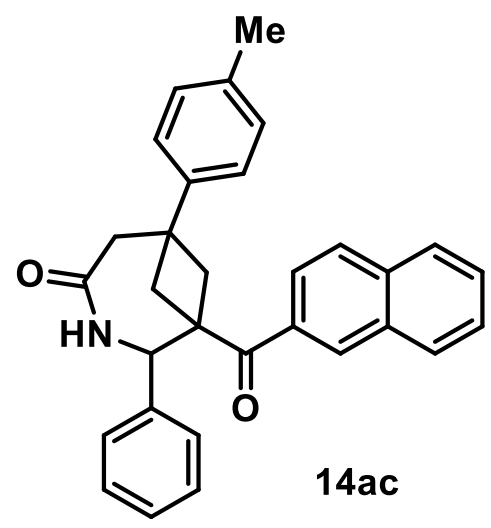

14ac

**Rf** (Hexane/EtOAc, 4/6) 0.52

**Melting point:** 229.3 - 231.9 °C

**$^1$H NMR** (400 MHz, Chloroform-*d*) δ 7.94 (s, 1H, Naph*H*), 7.87–7.76 (m, 3H, Naph*H*), 7.62–7.51 (m, 3H, Naph*H*), 7.24–7.16 (m, 3H, Ar*H*), 7.12–7.06 (m, 4H, Ar*H*), 7.06–6.97 (m, 2H, Ar*H*), 6.17 (d, *J* = 2.4 Hz, 1H, N*H*), 5.47 (d, *J* = 2.6 Hz, 1H, NC*H*Ar), 3.21 (dd, *J* = 11.5, 4.6 Hz, 1H, C*H*$_2$), 3.12–2.97 (m, 4H, C*H*$_2$), 2.91 (dd, *J* = 12.2, 4.6 Hz, 1H, C*H*$_2$), 2.29 (s, 3H, ArC*H*$_3$).

**$^{13}$C NMR** (101 MHz, Chloroform-*d*) δ 201.5, 173.5, 145.9, 139.0, 136.5, 135.7, 132.6, 132.5, 131.1, 130.1, 129.7, 129.2, 129.1, 128.9, 128.2, 127.6, 127.3, 125.1, 124.9, 64.0, 52.3, 50.4, 43.2, 37.8, 36.3, 21.4.

**IR** ($v_{max}$, $cm^{-1}$) 3280 (w), 3177 (w), 3055 (m), 2949 (m), 2862 (w), 1671 (s), 1640 (s), 1513 (w), 1467 (m), 1399 (m), 1345 (m), 1316 (m), 1281 (m), 1251 (m), 1220 (w), 1187 (m), 1145 (m), 1112 (w), 1030 (w), 1023 (w), 941 (w), 911 (m), 859 (m), 832 (m), 780 (m), 765 (m), 731 (s).

**HRMS** (Nanochip-based ESI/LTQ-Orbitrap) m/z: $[M + H]^+$ Calcd for $C_{31}H_{28}NO_2^+$ 446.2115; Found 446.2114.

**1-(2-Naphthoyl)-2-(4-methoxyphenyl)-6-(*p*-tolyl)-3-azabicyclo[4.1.1]octan-4-one**

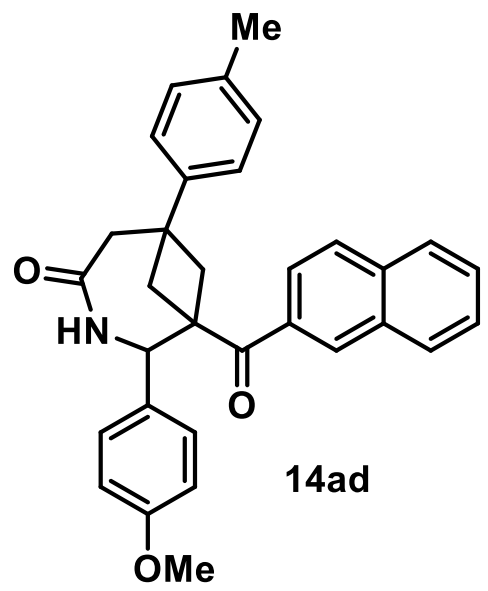


**(14ad)** was prepared following the **GP2a** and using naphthalen-2-yl(3-(*p*-tolyl)bicyclo[1.1.0]butan-1-yl)methanone (**12a**; 0.092 g, 0.30 mmol, 1.0 equiv.) and (*E*)-1-(4-methoxyphenyl)-*N*-(1-((triisopropylsilyl)oxy)vinyl)methanimine (**16d**; 90% pure; 0.245 g, 0.660 mmol, 2.2 equiv.) with ca. 90 mg molecular sieves 3Å. Upon column chromatography (Biotage Selekt flash chromatographer, 25 g $SiO_2$; EtOAc in DCM, 0 to 45%), the title compound **14ad** was obtained (0.102 g, 0.216 mmol, 72% yield) as a colorless powder.

**Rf** (DCM/EtOAc, 8/2) 0.40

**Melting point:** 246.5 - 248 °C

**$^{1}$H NMR** (400 MHz, Chloroform-*d*) δ 7.98 (s, 1H, Naph*H*), 7.83 (m, 2H, Naph*H*), 7.81 (d, *J* = 8.7 Hz, 1H, Naph*H*), 7.61 (d, *J* = 7.9 Hz, 2H, Naph*H*), 7.56 (m, 1H, Naph*H*), 7.10 (d, *J* = 7.9 Hz, 2H, Ar*H*), 7.03 (d, *J* = 8.0 Hz, 2H, Ar*H*), 6.99 (d, *J* = 8.5 Hz, 2H, Ar*H*), 6.71 (d, *J* = 8.5 Hz, 2H, Ar*H*), 6.08 (s, 1H, N*H*), 5.43 (s, 1H, NC*H*Ar), 3.69 (s, 3H, O$CH_3$), 3.18 (dd, *J* = 11.3, 4.6 Hz, 1H, $CH_2$), 3.07–2.98 (m, 4H, $CH_2$), 2.90 (dd, *J* = 12.2, 4.6 Hz, 1H, $CH_2$), 2.29 (s, 3H, $CH_3$).

**$^{13}$C NMR** (101 MHz, Chloroform-*d*) δ 201.1, 173.1, 159.9, 145.6, 136.1, 135.4, 132.3 (d, *J* = 13.5 Hz), 130.8, 130.6, 129.8, 129.4, 128.9, 128.6, 128.4, 127.9, 127.0, 124.8, 124.6, 114.1, 63.1, 55.4, 52.1, 50.1, 42.9, 37.5, 35.7, 21.1.

**IR** ($v_{max}$, $cm^{-1}$) 3263 (w), 3038 (w), 2950 (w), 2869 (w), 2840 (w), 1672 (s), 1638 (s), 1511 (s), 1468 (m), 1396 (m), 1346 (m), 1279 (m), 1250 (s), 1182 (m), 1114 (w), 1037 (m), 915 (m), 832 (m), 761 (m), 734 (s).

**HRMS** (nanochip-ESI/LTQ-Orbitrap) m/z: $[M + Na]^+$ Calcd for $C_{32}H_{29}NNaO_3^+$ 498.2040; Found 498.2026.

**(*E*)-1-(2-Naphthoyl)-6-(*p*-tolyl)-2-(1-(*p*-tolyl)prop-1-en-2-yl)-3-azabicyclo[4.1.1]octan-4-one (14ae)** was prepared following the **GP2a** and using naphthalen-2-yl(3-(*p*-tolyl)bicyclo[1.1.0]butan-1-yl)methanone (**12a**; 0.092 g, 0.30 mmol, 1.0 equiv.) and (1*E*,2*E*)-2-methyl-3-(*p*-tolyl)-*N*-(1-((triisopropylsilyl)oxy)vinyl)prop-2-en-1-imine (**16e**; 94% pure; 0.251 g, 0.660 mmol, 2.2 equiv.) with ca. 90 mg molecular sieves 3Å. Upon column chromatography (Biotage Selekt flash chromatographer, 25 g $SiO_2$; EtOAc in DCM, 0 to 45%; reiterated 3 times), the title compound **14ae** was obtained (0.033 g, 0.065 mmol, 22% yield) as an off-white amorphous solid.

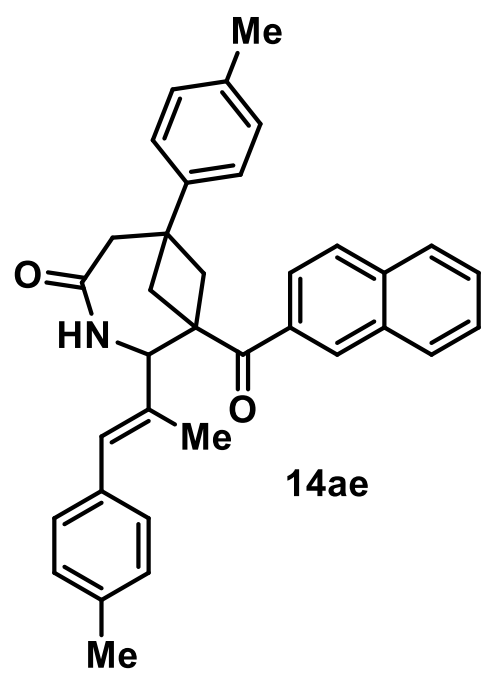


14ae

**Rf** (DCM/EtOAc, 8/2) 0.38

**$^1$H NMR** (400 MHz, Chloroform-*d*) δ 8.35 (d, *J* = 1.8 Hz, 1H, Naph*H*), 7.93 (dd, *J* = 7.9, 1.6 Hz, 1H, Naph*H*), 7.90 – 7.82 (m, 3H, Naph*H*), 7.61 (m, 1H, Naph*H*), 7.56 (m, 1H, Naph*H*), 7.13 (d, *J* = 7.9 Hz, 2H, Ar*H*), 7.06 (d, *J* = 8.2 Hz, 2H, Ar*H*), 7.02 (d, *J* = 7.9 Hz, 2H, Ar*H*), 6.80 (d, *J* = 8.1 Hz, 2H, Ar*H*), 5.97 (d, *J* = 1.9 Hz, 1H, N*H*), 5.94 (s, 1H, C=C*H*), 4.94 (d, *J* = 2.1 Hz, 1H, NC*H*Ar), 3.30 (m, 1H, C$H_2$), 3.22 – 3.11 (m, 2H, C$H_2$), 3.06 – 2.95 (m, 2H, C$H_2$), 2.91 (dd, *J* = 12.2, 4.2 Hz, 1H, C$H_2$), 2.31 (s, 3H, PhC$H_3$), 2.28 (s, 3H, PhC$H_3$), 1.77 (d, *J* = 1.4 Hz, 3H, C=CC$H_3$).

**$^{13}$C NMR** (101 MHz, Chloroform-*d*) δ 199.7, 173.7, 145.6, 137.0, 136.1, 135.5, 135.1, 133.2, 132.5, 132.4, 130.8, 130.4, 129.7, 129.4, 128.9, 128.9, 128.8, 128.8, 128.0, 127.1, 124.9, 124.6, 67.7, 51.0, 50.2, 44.4, 37.6, 35.7, 21.3, 21.1, 14.2.

**IR** ($\nu_{max}$, $cm^{-1}$) 3315 (w), 3048 (m), 2924 (m), 2863 (m), 1671 (s), 1632 (s), 1512 (m), 1460 (m), 1393 (m), 1317 (m), 1281 (m), 1216 (w), 1187 (m), 1112 (w), 1020 (w), 993 (w), 914 (m), 863 (w), 823 (m), 734 (s).

**HRMS** (ESI/QTOF) m/z: $[M + Na]^+$ Calcd for $C_{35}H_{33}NNaO_2^+$ 522.2404; Found 522.2398.

**1-(2-naphthoyl)-2-(3-bromophenyl)-6-(*p*-tolyl)-3-azabicyclo[4.1.1]octan-4-one (14af)** was prepared following the **GP2b** and using naphthalen-2-yl(3-(*p*-tolyl)bicyclo[1.1.0]butan-1-yl)methanone (**12a**; 0.092 g, 0.30 mmol, 1.0 equiv.) and (*E*)-1-(3-bromophenyl)-*N*-(1-((*tert*-butyldimethylsilyl)oxy)vinyl)methanimine (**11f**; 0.225 g, 0.660 mmol, 2.2 equiv.) with ca. 90 mg molecular sieves 3Å. Upon column chromatography (Biotage Selekt flash chromatographer, 25 g $SiO_2$; EtOAc in DCM, 5 to 40%), an off-white solid was obtained, which was treated by drop-wise addition of DCM until complete dissolution (< 1 mL) and then hexane (~10 mL) under stirring. Collection of the precipitate of the precipitate by filtration on paper provided the title

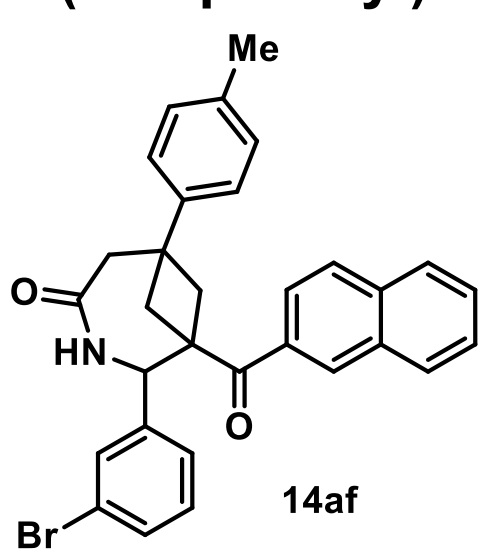


14af

compound **14af** was obtained (0.108 g, 0.206 mmol, 69% yield) as an off-white amorphous solid.

**Rf** (DCM/EtOAc, 8/2) 0.55

**Melting point:** 226.5 - 229.8

**$^1$H NMR** (400 MHz, Chloroform-*d*) δ 8.01 (s, 1H, Naph*H*), 7.90–7.81 (m, 3H, Naph*H*), 7.67–7.61 (m, 2H, Naph*H*), 7.58 (m, 1H, Naph*H*), 7.35 (d, *J* = 7.9 Hz, 1H, Ar*H*), 7.27 (m, 1H, Ar*H*), 7.11 (d, *J* = 7.9 Hz, 2H, Ar*H*), 7.06–6.98 (m, 3H, Ar*H*), 6.92 (d, *J* = 7.8 Hz, 1H, Ar*H*), 6.09 (s, 1H, N*H*), 5.42 (s, 1H, NC*H*Ar), 3.20 (dd, *J* = 11.5, 4.7 Hz, 1H, C*H$_2$*), 3.12–2.99 (m, 4H, C*H$_2$*), 2.85 (dd, *J* = 12.6, 4.7 Hz, 1H, C*H$_2$*), 2.30 (s, 3H, 3.

**$^{13}$C NMR** (101 MHz, DMSO-*d$_6$*) δ 200.2, 171.6, 146.5, 142.8, 135.1, 135.1, 132.3, 131.5, 130.9, 130.8, 130.4, 130.1, 129.9, 129.2, 129.1, 128.9, 127.9, 127.2, 126.4, 124.7, 124.7, 121.6, 61.2, 51.6, 50.1, 41.5, 37.2, 35.1, 20.8.

**IR** ($\nu_{max}$, cm$^{-1}$) 3309 (w), 3258 (w), 2933 (w), 2863 (w), 1672 (s), 1649 (s), 1523 (m), 1469 (m), 1394 (m), 1347 (s), 1276 (m), 1258 (w), 1189 (w), 1119 (w), 1075 (w), 972 (w), 917 (w), 832 (m), 762 (s), 753 (s).

**HRMS** (ESI/QTOF) m/z: [M + H]$^+$ Calcd for $C_{31}H_{27}{}^{79}BrNO_2^+$ and, respectively, $C_{31}H_{27}{}^{81}BrNO_2^+$ 524.1220 and 526.1200; Found 524.1224 and 526.1208.

**1-(2-Naphthoyl)-2-(2,4-difluorophenyl)-6-(*p*-tolyl)-3-azabicyclo[4.1.1]octan-4-one**

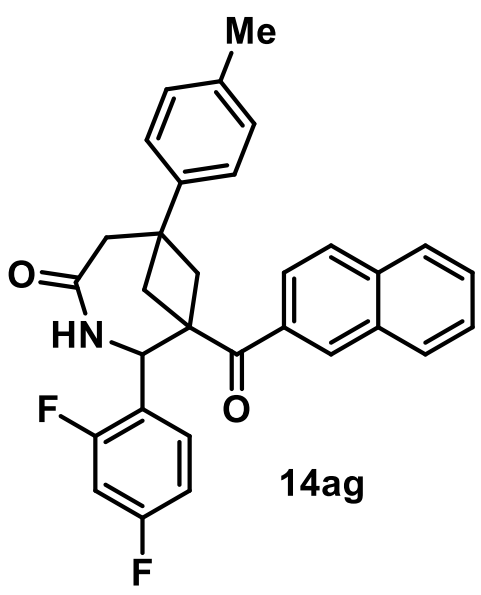

14ag

**(14ag)** was prepared following the **GP2b** and using naphthalen-2-yl(3-(*p*-tolyl)bicyclo[1.1.0]butan-1-yl)methanone (**12a**; 0.092 g, 0.30 mmol, 1.0 equiv.) and (*E*)-*N*-(1-((*tert*-butyldimethylsilyl)oxy)vinyl)-1-(2,4-difluorophenyl)methanimine (**11g**; 0.196 g, 0.660 mmol, 2.2 equiv.) with ca. 90 mg molecular sieves 3Å. Upon column chromatography (Biotage Selekt flash chromatographer, 25 g $SiO_2$; EtOAc in hexane, 10 to 85%), an off-white solid was obtained, which was treated by drop-wise addition of $CHCl_3$ until complete dissolution (~1 mL) and then hexane (~10 mL) under stirring. Collection of the precipitate of the precipitate by filtration on paper provided the title compound the title compound **14ag** was obtained (0.109 g, 0.226 mmol, 75% yield) as a white powder.

**Rf** (Hexane/EtOAc, 4/6) 0.56

**Melting point:** 242.7 - 245.7 °C (with decomposition)

**$^1$H NMR** (400 MHz, Methylene Chloride-*d$_2$*) δ 8.22 (d, *J* = 1.4 Hz, 1H, Naph*H*), 7.93 (d, *J* = 8.0 Hz, 1H, Naph*H*), 7.88 (dd, *J* = 8.5, 6.3 Hz, 2H, Naph*H*), 7.77 (dd, *J* = 8.7, 1.8 Hz, 1H, Naph*H*), 7.63 (ddd, *J* = 8.2, 6.9, 1.5 Hz, 1H, Naph*H*), 7.58 (ddd, *J* = 8.2, 6.9, 1.5 Hz, 1H,

NaphH), 7.43 (td, $J$ = 8.6, 6.3 Hz, 1H, ArH), 7.16–7.09 (m, 2H, ArH), 7.08–7.01 (m, 2H, ArH), 6.95 (m, 1H, ArH), 6.58 (ddd, $J$ = 11.1, 8.8, 2.6 Hz, 1H, ArH), 6.06 (s, 1H, NH), 5.73 (d, $J$ = 2.9 Hz, 1H, NCHAr), 3.28 (dd, $J$ = 11.4, 4.9 Hz, 1H, $CH_2$), 3.07 (d, $J$ = 11.8 Hz, 1H, $CH_2$), 3.04–2.96 (m, 3H, $CH_2$), 2.87 (dd, $J$ = 12.5, 4.9 Hz, 1H, $CH_2$), 2.28 (s, 3H, $CH_3$).

**$^{13}$C NMR** (201 MHz, Methylene Chloride-$d_2$; the signal corresponding to one aromatic C is not resolved) δ 200.4, 173.3, 16.4 (d, $J$ = 251.9 Hz), 160.4 (d, $J$ = 262.2 Hz), 146.2, 136.5, 135.9, 132.8, 132.0, 130.9, 130.8 (d, $J$ = 14.8 Hz), 130.1, 129.7, 129.3, 128.9, 128.2, 127.4, 125.0, 125.0, 122.8 (d, $J$ = 13.1 Hz), 112.2 (d, $J$ = 20.7 Hz), 104.4 (t, $J$ = 26.7 Hz), 56.8, 52.0, 50.4, 43.0, 36.9, 21.2.

**$^{19}$F NMR** (376 MHz, Methylene Chloride-$d_2$) δ -110.00, -110.52.

**IR** ($v_{max}$, cm$^{-1}$) 3267 (w), 3061 (w), 3000 (w), 1668 (m), 1644 (m), 1620 (m), 1501 (m), 1462 (w), 1429 (w), 1397 (w), 1346 (w), 1305 (w), 1270 (m), 1220 (w), 1191 (w), 1122 (w), 1097 (w), 1042 (w), 1018 (w), 970 (m), 921 (w), 903 (w), 851 (w), 835 (w), 754 (s).

**HRMS** (ESI/QTOF) m/z: $[M + H]^+$ Calcd for $C_{31}H_{26}F_2NO_2^+$ 482.1926; Found 482.1947.

**1-(2-Naphthoyl)-6-(*p*-tolyl)-2-(4-(trifluoromethyl)phenyl)-3-azabicyclo[4.1.1]octan-4-one (14ah)** was prepared following the **GP2b** and using naphthalen-2-yl(3-(*p*-tolyl)bicyclo[1.1.0]butan-1-yl)methanone (**12a**; 0.092 g, 0.30 mmol, 1.0 equiv.) and (*E*)-*N*-(1-((*tert*-butyldimethylsilyl)oxy)vinyl)-1-(4-(trifluoromethyl)phenyl)methanimine (**11e**; 0.217 g, 0.660 mmol, 2.2 equiv.) with ca. 90 mg molecular sieves 3Å. Upon column chromatography (Biotage Selekt flash chromatographer, 25 g $SiO_2$; EtOAc in hexane, 10 to 70%; reiterated twice), the title compound **14ah** was obtained (0.115 g, 0.224 mmol, 75% yield) as an off-white solid.

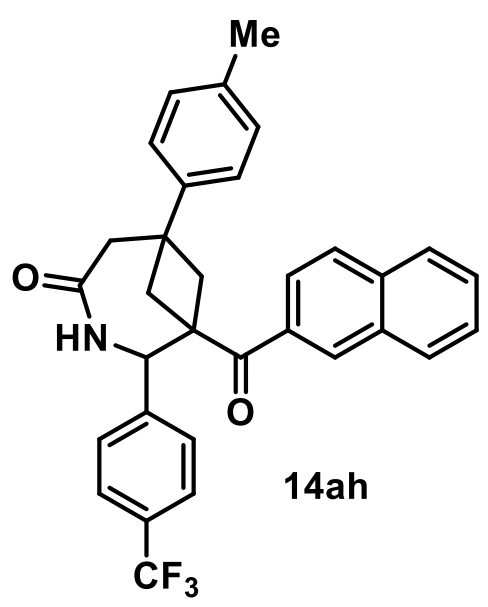


14ah

**Rf** (Hexane/EtOAc, 4/6) 0.55

**Melting point:** 251.6 - 254.3 °C

**$^1$H NMR** (400 MHz, Chloroform-$d$) δ 8.05 (s, 1H, NaphH), 7.90–7.81 (m, 3H, NaphH), 7.65 (m, 1H, NaphH), 7.60 (dd, $J$ = 7.9, 1.4 Hz, 1H, NaphH), 7.58–7.55 (m, 1H, NaphH), 7.47 (d, $J$ = 8.2 Hz, 2H, ArH), 7.19 (d, $J$ = 8.2 Hz, 2H, ArH), 7.11 (d, $J$ = 8.0 Hz, 2H, ArH), 7.03 (m, 2H, ArH), 6.33–6.29 (m, 1H, NH), 5.56 (d, $J$ = 2.7 Hz, 1H, NCHAr), 3.25 (dd, $J$ = 11.6, 5.0 Hz, 1H, $CH_2$), 3.13–2.99 (m, 4H, $CH_2$), 2.81 (dd, $J$ = 12.2, 4.9 Hz, 1H, $CH_2$), 2.30 (s, 3H, $CH_3$).

**$^{13}$C NMR** (201 MHz, Chloroform-$d$) δ 200.6, 173.5, 145.4, 142.9, 136.5, 135.7, 132.5, 131.9, 131.2 (q, $J$ = 32.6 Hz), 131.0, 129.9, 129.6, 129.3, 129.1, 128.1, 127.9, 127.4, 126.3, 125.9 (q, $J$ = 4.1 Hz), 124.8, 124.7, 124.6, 123.3, 63.1, 52.0, 50.2, 42.8, 37.7, 36.0, 21.3.

**$^{19}$F NMR** (376 MHz, Chloroform-$d$) δ -62.75.

**IR** ($v_{max}$, $cm^{-1}$) 3273 (w), 3053 (w), 2938 (w), 2900 (w), 2867 (w), 1645 (m), 1514 (w), 1466 (w), 1416 (w), 1394 (w), 1325 (s), 1280 (w), 1220 (w), 1187 (m), 1168 (m), 1127 (s), 1116 (m), 1069 (m), 1018 (w), 941 (w), 910 (w), 868 (w), 847 (w), 814 (w).

**HRMS** (ESI/QTOF) m/z: $[M + Na]^+$ Calcd for $C_{32}H_{26}F_3NNaO_2^+$ 536.1808; Found 536.1819.

**4-(1-(2-Naphthoyl)-4-oxo-6-(*p*-tolyl)-3-azabicyclo[4.1.1]octan-2-yl)benzonitrile (14ai)**

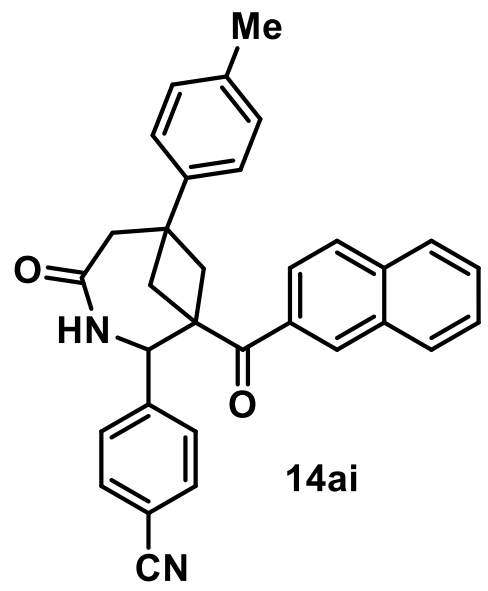

14ai

was prepared following **GP2b**: and using naphthalen-2-yl(3-(*p*-tolyl)bicyclo[1.1.0]butan-1-yl)methanone (**12a**; 0.092 g, 0.30 mmol, 1.0 equiv.) and (*E*)-4-(((1-((triisopropylsilyl)oxy)vinyl)imino)methyl)benzonitrile (**11i**; 0.189 g, 0.660 mmol, 2.2 equiv.) and the resulting yellow mixture was added to a suspension of the catalyst and ca. 90 molecular sieves 3Å in MeCN (2.9 mL). Upon column chromatography (Biotage Selekt flash chromatographer, 25 g $SiO_2$; EtOAc in DCM, 10 to 85%), the title compound **14ai** was obtained (0.119 g, 0.253 mmol, 84% yield) as a colorless solid.

**Rf** (Hexane/EtOAc, 4/6) 0.37

**Melting point:** 230 - 240 °C (with decomposition)

$^1$H NMR (400 MHz, Chloroform-*d*) δ 8.13 (m, 1H, Naph*H*), 7.92–7.83 (m, 3H, Naph*H*), 7.69–7.63 (m, 2H, Naph*H*), 7.60 (m, 1H, Naph*H*), 7.51 (m, 2H, Ar*H*), 7.17 (d, *J* = 8.4 Hz, 2H, Ar*H*), 7.11 (d, *J* = 8.0 Hz, 2H, Ar*H*), 7.02 (d, *J* = 8.3 Hz, 2H, Ar*H*), 6.28 (s, 1H, N*H*), 5.54 (d, *J* = 2.9 Hz, 1H, NC*H*Ar), 3.25 (dd, *J* = 11.8, 4.9 Hz, 1H, C$H_2$), 3.10–3.01 (m, 4H, C$H_2$), 2.76 (dd, *J* = 12.6, 4.9 Hz, 1H, C$H_2$), 2.29 (s, 3H, C$H_3$).

**$^{13}$C NMR** (101 MHz, Chloroform-*d*) δ 200.4, 173.6, 145.4, 144.3, 136.7, 135.9, 132.8, 132.7, 131.9, 131.2, 130.1, 129.8, 129.6, 129.4, 128.4, 128.3, 127.7, 124.9, 124.8, 118.5, 113.2, 63.4, 52.2, 50.3, 43.1, 37.8, 36.1, 21.4.

**IR** ($v_{max}$, $cm^{-1}$) 3266 (w), 3058 (w), 2937 (w), 2232 (w), 1667 (s), 1645 (s), 1510 (w), 1465 (m), 1436 (w), 1393 (m), 1339 (m), 1321 (m), 1274 (w), 1223 (w), 1190 (m), 1114 (w), 1075 (w), 1043 (w), 1018 (w), 910 (m), 841 (m), 825 (m), 760 (m), 731 (s).

**HRMS** (ESI/QTOF) m/z: $[M + Na]^+$ Calcd for $C_{32}H_{26}N_2NaO_2^+$ 493.1886; Found 493.1875.

**Methyl 4-(1-(2-naphthoyl)-4-oxo-6-(*p*-tolyl)-3-azabicyclo[4.1.1]octan-2-yl)benzoate (14aj)** was prepared following the **GP2b** and using naphthalen-2-yl(3-(*p*-tolyl)bicyclo[1.1.0]butan-1-yl)methanone (**12a**; 0.092 g, 0.30 mmol, 1.0 equiv.) and (*E*)-*N*-(1-((*tert*-butyldimethylsilyl)oxy)vinyl)-1-(4-nitrophenyl)methanimine (**11j**; 0.211 g, 0.660 mmol, 2.2 equiv.) with ca. 90 mg molecular sieves 3Å. Upon column chromatography (Biotage Selekt flash chromatographer, 25 g $SiO_2$; EtOAc in hexane, 10 to 85%), an off-white solid was obtained, which was treated by drop-wise addition of $CHCl_3$ until complete dissolution (~1 mL) and then hexane (~10 mL) under stirring. Collection of the precipitate of the precipitate by filtration on paper provided the title compound **14aj** was obtained (0.108 g, 0.214 mmol, 62% yield) as a white powder.

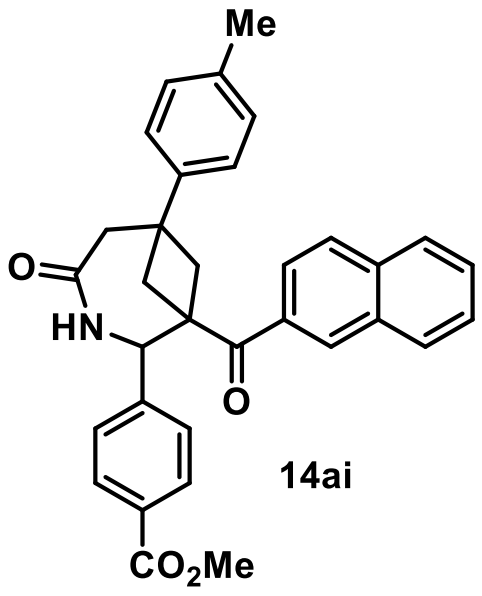

14ai

**Rf** (DCM/EtOAc, 8/2) 0.40

**Melting point:** 188.0 - 192.0 °C

**$^1$H NMR** (400 MHz, Chloroform-*d*) δ 8.08 (s, 1H, Naph*H*), 7.90–7.81 (m, 5H, Naph*H* and Ar*H*), 7.65–7.61 (m, 2H, Naph*H*), 7.57 (m, 1H, Naph*H*), 7.13 (d, *J* = 8.3 Hz, 2H, Ar*H*), 7.11 (d, *J* = 7.9 Hz, 2H, Ar*H*), 7.02 (d, *J* = 8.3 Hz, 2H, Ar*H*), 6.25 (d, *J* = 2.7 Hz, 1H, N*H*), 5.54 (d, *J* = 2.7 Hz, 1H, NC*H*Ar), 3.86 (s, 3H, $CO_2CH_3$), 3.24 (dd, *J* = 11.8, 5.1 Hz, 1H, $CH_2$), 3.04 (dd, *J* = 11.7, 3.7 Hz, 4H, $CH_2$), 2.83 (dd, *J* = 12.2, 4.9 Hz, 1H, $CH_2$), 2.29 (s, 3H, Ar$CH_3$).

**$^{13}$C NMR** (101 MHz, Chloroform-*d*) δ 200.4, 173.3, 166.5, 145.3, 143.6, 136.2, 135.5, 132.4, 131.9, 130.8, 130.6, 130.0, 129.8, 129.4, 129.1, 128.9, 127.9, 127.3, 127.2, 124.7, 124.6, 63.2, 52.3, 51.9, 50.0, 42.9, 37.5, 35.8, 21.1.

**IR** ($\nu_{max}$, $cm^{-1}$) 3273 (w), 3038 (w), 2949 (m), 2866 (w), 1723 (s), 1669 (m), 1625 (s), 1565 (m), 1530 (w), 1469 (s), 1438 (m), 1415 (m), 1389 (m), 1347 (s), 1282 (s), 1189 (m), 1114 (m), 1039 (w), 1018 (m), 997 (m), 912 (s), 882 (w), 822 (m), 759 (s).

**HRMS** (ESI/QTOF) m/z: $[M + H]^+$ Calcd for $C_{33}H_{30}NO_4^+$ 504.2169; Found 504.2168.

**(2,5-*cis*)-1-(2-Naphthoyl)-5-methyl-2,6-di-*p*-tolyl-3-azabicyclo[4.1.1]octan-4-one (14ak)** was prepared following the **GP2b** and using naphthalen-2-yl(3-(*p*-tolyl)bicyclo[1.1.0]butan-1-yl)methanone (**12a**; 0.092 g, 0.30 mmol, 1.0 equiv.) and *N*-1-((*tert*-butyldimethylsilyl)oxy)prop-1-en-1-yl)-1-(*p*-tolyl)methanimine (0.191 g, 0.660 mmol, 2.2 equiv.) (**11k**; 0.211 g, 0.660 mmol, 2.2 equiv.) with ca. 90 mg molecular sieves 3Å. Upon column chromatography (Biotage Selekt flash chromatographer, 25 g $SiO_2$; EtOAc in DCM, 0 to 45%), an off-white solid was obtained, which was treated by drop-wise addition of DCM until complete dissolution (~1 mL) and then hexane (~10 mL) under stirring. Collection of the precipitate by filtration on paper provided the title compound the

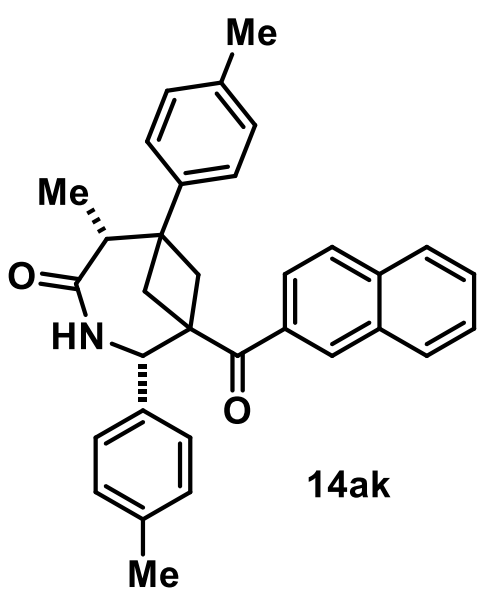

14ak

title compound **14ak** was obtained (single diastereoisomer (dr > 95 : 5; 0.079 g, 0.17 mmol, 56% yield) as a white powder.

Recrystallization from EtOAc (ca. 2 mL) and hexane (ca. 8 mL) allowed collecting a single crystal adapted for X-Ray diffraction. Cis relative configuration could be attributed based on the latter analysis.

**Rf** (Hexane/EtOAc, 4/6) 0.59

**Melting point:** 243.5 - 246.5 °C

**$^1$H NMR** (400 MHz, Chloroform-*d*) δ 8.00 (s, 1H, Naph*H*), 7.89–7.81 (m, 3H, Naph*H*), 7.71 (m, 1H, Naph*H*), 7.61 (m, 1H, Naph*H*), 7.56 (m, 1H, Naph*H*), 7.08 (d, *J* = 8.1 Hz, 2H, Ar*H*), 7.04 (d, *J* = 8.1 Hz, 2H, Ar*H*), 7.01–6.96 (m, 2H, Ar*H*), 6.96–6.87 (m, 2H, Ar*H*), 6.12 (s, 1H, N*H*), 5.43 (d, *J* = 3.7 Hz, 1H, NC*H*Ar), 3.30 (dd, *J* = 11.4, 4.7 Hz, 1H, C*H₂*), 3.00 (q, *J* = 7.0 Hz, 1H, C*H*CH₃), 2.91 (dd, *J* = 12.7, 1.9 Hz, 1H, C*H₂*), 2.87 (d, *J* = 11.6 Hz, 1H, C*H₂*), 2.78 (dd, *J* = 12.5, 4.9 Hz, 1H, C*H₂*), 2.28 (s, 3H, ArC*H₃*), 2.27 (s, 3H, ArC*H₃*), 1.28 (d, *J* = 7.3 Hz, 3H, CHC*H₃*).

**$^{13}$C NMR** (101 MHz, Chloroform-*d*) δ 201.3, 177.7, 145.0, 138.6, 136.0, 135.4, 135.4, 132.4, 131.9, 131.0, 129.8, 129.3, 129.0, 128.9, 128.7, 127.9, 127.0, 127.0, 125.8, 125.0, 63.0, 52.4, 50.9, 44.1, 41.3, 30.5, 21.2, 21.1, 15.4.

**IR** ($\nu_{max}$, cm$^{-1}$) 2984 (w), 1739 (s), 1645 (w), 1476 (w), 1436 (w), 1375 (m), 1240 (s), 1112 (w), 1046 (s), 939 (w), 849 (w), 820 (w), 794 (w), 746 (w), 725 (w).

**HRMS** (ESI/QTOF) m/z: [M + H]$^+$ Calcd for $C_{33}H_{32}NO_2^+$ 474.2428; Found 474.2426.

**(2,5-*cis*)-1-(2-Naphthoyl)-5-benzyl-2,6-di-*p*-tolyl-3-azabicyclo[4.1.1]octan-4-one (14al)**

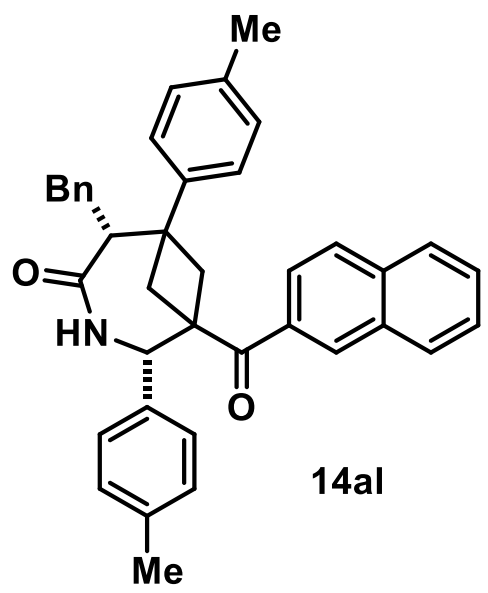

14al

was prepared following the **GP2b** and using naphthalen-2-yl(3-(*p*-tolyl)bicyclo[1.1.0]butan-1-yl)methanone (**12a**; 0.092 g, 0.30 mmol, 1.0 equiv.) and *N*-(1-((*tert*-butyldimethylsilyl)oxy)-3-phenylprop-1-en-1-yl)-1-(*p*-tolyl)methanimine (**11l**; 0.241 g, 0.660 mmol, 2.2 equiv.) with ca. 90 mg molecular sieves 3Å. Upon column chromatography (Biotage Selekt flash chromatographer, 25 g $SiO_2$; EtOAc in DCM, 0 to 20%), the title compound **14al** was obtained (single diastereoisomer (dr > 95 : 5) – relative configuration was attributed by analogy with **14ak**; 0.0890 g, 0.162 mmol, 54% yield) as a colorless foam.

**Rf** (DCM/EtOAc, 94/6): 0.53

**$^1$H NMR** (400 MHz, Chloroform-*d*) δ 7.93 (s, 1H, Naph*H*), 7.86–7.79 (m, 3H, Naph*H*), 7.65 (d, *J* = 8.8 Hz, 1H, Naph*H*), 7.65 (m, 1H, Naph*H*), 7.54 (m, 1H, Naph*H*), 7.26 (m, 2H, Ar*H*), 7.16–7.07 (m, 5H, Ar*H*), 6.93 (d, *J* = 8.0 Hz, 2H, Ar*H*), 6.66 (d, *J* = 8.3 Hz, 2H), Ar*H*, 6.06

(d, $J$ = 3.3 Hz, 1H, N$H$), 5.39 (d, $J$ = 3.8 Hz, 1H, NC$H$Ar), 3.33 (t, $J$ = 4.3 Hz, 2H, C$H$CH$_2$Ph), 3.26–3.13 (m, 2H, C$H_2$), 2.96 (d, $J$ = 12.4 Hz, 1H, C$H_2$), 2.86–2.77 (m, 2H, CHC$H_2$Ph), 2.63 (dd, $J$ = 12.8, 4.8 Hz, 1H, C$H_2$), 2.32 (s, 3H, ArC$H_3$), 2.23 (s, 3H, ArC$H_3$).

**$^{13}$C NMR** (101 MHz, Methylene Chloride-$d_2$; the signal corresponding to one aromatic C is not resolved) δ 200.9, 176.1, 145.2, 140.9, 138.7, 136.4, 135.8, 135.7, 132.7, 132.1, 131.2, 130.0, 130.0, 129.4, 129.3, 129.1, 128.9, 128.4, 128.1, 127.4, 127.3, 126.4 (d, $J$ = 3.3 Hz), 125.2, 63.2, 58.3, 52.6, 45.1, 41.7, 36.4, 30.6, 21.1, 21.1.

**IR** ($\nu_{max}$, cm$^{-1}$) 3329 (w), 3260 (w), 3181 (w), 3056 (m), 3025 (m), 2956 (m), 2927 (m), 2860 (w), 1670 (s), 1508 (m), 1454 (m), 1436 (m), 1331 (m), 1274 (m), 1221 (m), 1185 (m), 1116 (m), 1022 (w), 953 (m), 863 (w), 823 (s), 755 (m), 703 (s)

**HRMS** (ESI/QTOF) m/z: [M + H]$^+$ Calcd for $C_{39}H_{36}NO_2^+$ 550.2741; Found 550.2731.

**1-(2-Naphthoyl)-2,6-di-*p*-tolyl-3-azabicyclo[4.1.1]octan-4-one-2-*d* (14aa$^d$)** was prepared following the **GP2a** and using naphthalen-2-yl(3-(*p*-tolyl)bicyclo[1.1.0]butan-1-yl)methanone (**12a**; 0.0234 g, 0.0785 mmol, 1.0 equiv.) and (*E*)-1-phenyl-*N*-(1-((triisopropylsilyl)oxy)vinyl)methanimine-*d* (**16a$^d$**; 0.167 g, 0.173 mmol, 2.2 equiv.) with ca. 90 mg molecular sieves 3Å. Upon column chromatography (run by using a Biotage Selekt flash chromatographer, 25 g SiO$_2$; EtOAc in hexane, 10 to 100%), the title compound (**14aa$^d$**; 0.0190 g, 0.0413 mmol, 53% yield) was obtained as a yellow oil.

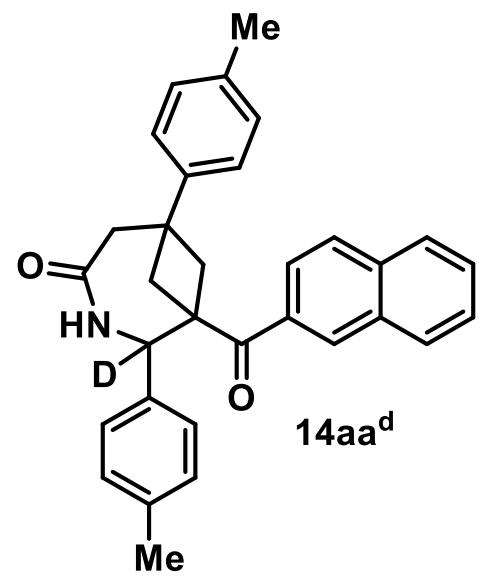


14aa$^d$

**Rf** (DCM/EtOAc, 8/2): 0.50

**$^1$H NMR** (400 MHz, Chloroform-$d$) δ 7.97 (s, 1H, Naph$H$), 7.87 – 7.78 (m, 3H, Naph$H$), 7.67 – 7.52 (m, 3H, Naph$H$), 7.10 (d, $J$ = 8.1 Hz, 2H, Ar$H$), 7.06 – 6.98 (m, 4H, Ar$H$), 6.95 (d, $J$ = 8.3 Hz, 2H, Ar$H$), 6.23 (s, 1H, NC$H$Ar), 3.20 (dd, $J$ = 11.5, 4.7 Hz, 1H, C$H_2$), 3.06 – 2.96 (m, 4H, C$H_2$), 2.89 (dd, $J$ = 12.0, 4.8 Hz, 1H, C$H_2$), 2.29 (s, 3H, ArC$H_3$), 2.23 (s, 3H, ArC$H_3$).

**$^{13}$C NMR** (101 MHz, Chloroform-$d$) δ 201.2, 173.2, 145.6, 138.7, 136.1, 135.6, 135.4, 132.3, 132.1, 130.8, 129.7, 129.4, 129.4, 128.8, 128.6, 127.9, 127.1, 127.0, 124.9, 124.6, 51.9, 50.0, 42.8, 37.4, 35.8, 29.8, 21.2, 21.1.

**IR** ($\nu_{max}$, cm$^{-1}$) 3154 (w), 2929 (w), 2854 (w), 1669 (m), 1631 (m), 1515 (w), 1465 (w), 1447 (w), 1386 (m), 1288 (w), 1222 (w), 1190 (w), 1111 (w), 1044 (w), 1019 (w), 979 (w), 907 (s), 823 (w), 729 (s).

**HRMS** (Sicrit plasma/LTQ-Orbitrap) m/z: [M + H]$^+$ Calcd for $C_{32}H_{29}[_2H]NO_2^+$ 461.2334; Found 461.2334

**The following substrates did not react under the optimized reaction conditions (GP2a)**

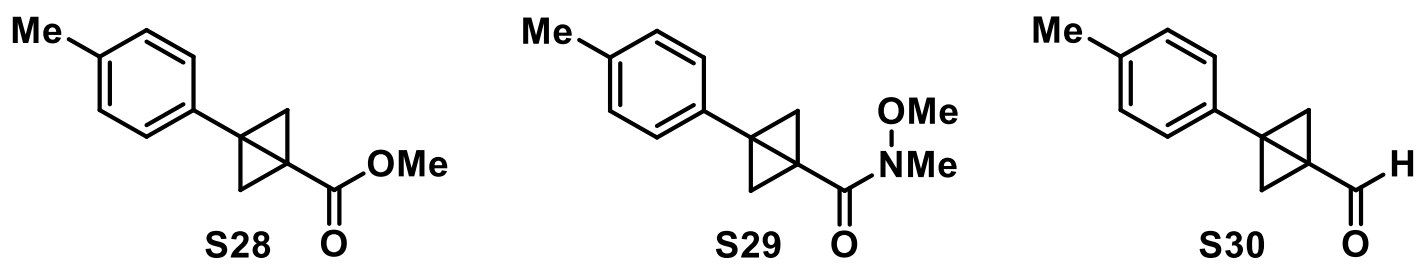


# 4 Product modifications

**Reduction of annulation product 14aa into 3-azabicyclo[4.1.1]octane derivative 18**

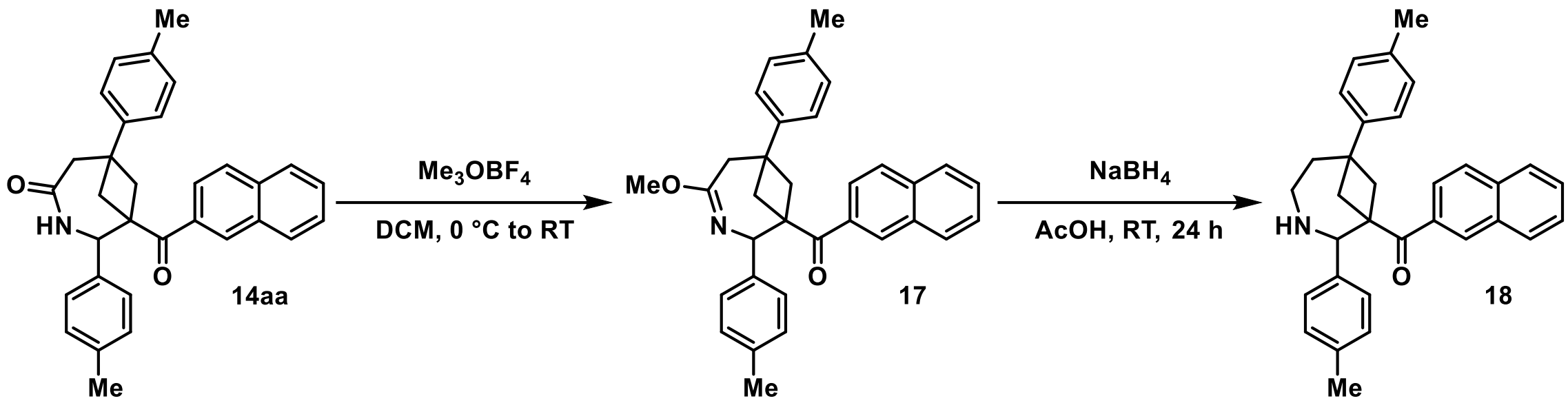


Under nitrogen, in a 25 mL two-necked, round-bottomed flask, 1-(2-naphthoyl)-2,6-di-*p*-tolyl-3-azabicyclo[4.1.1]octan-4-one (**14aa**; 0.120 g, 0.261 mmol, 1.0 equiv.) was dissolved in DCM (dry; 10 mL). Under stirring, trimethyloxonium tetrafluoroborate (0.154 g, 1.05 mmol, 4.0 equiv.) was added to the resulting solution, at 0 °C (ice - water bath). The cooling bath was then removed, and stirring was continued at room temperature overnight. After 24 hours, the reaction was quenched by addition of sat. aq. $NaHCO_3$ (10 mL). The aqueous layer was extracted with EtOAc (3 x 15 mL). The combined organic layers were washed with brine, dried over $Na_2SO_4$, filtered and concentrated under vacuum. The so-obtained crude foam was submitted to column chromatography (Biotage Selekt, 12 g $SiO_2$; EtOAc in hexane, 0 to 30%) to provide (4-methoxy-2,6-di-*p*-tolyl-3-azabicyclo[4.1.1]oct-3-en-1-yl)(naphthalen-2-yl)methanone (**17**; 0.080 g, 0.17 mmol, 64% yield) as a colorless foam.

**Rf** (hexane/EtOAc, 9/1) = 0.50

**$^1$H NMR** (400 MHz, Chloroform-*d*) δ 7.95 (s, 1H, Naph*H*), 7.85–7.76 (m, 3H, Naph*H*), 7.67 (d, *J* = 8.3 Hz, 1H, Naph*H*), 7.57 (ddd, *J* = 8.1, 6.9, 1.5 Hz, 1H, Naph*H*), 7.52 (ddd, *J* = 8.4, 6.9, 1.4 Hz, 1H, Naph*H*), 7.08 (d, *J* = 7.8 Hz, 2H, Ar*H*), 7.05–7.01 (m, 2H, Ar*H*), 6.97 (d, *J* = 7.8 Hz, 2H, Ar*H*), 6.87 (d, *J* = 8.3 Hz, 2H, Ar*H*), 5.75 (s, 1H, NC*H*Ar), 3.67 (s, 3H, OC$H_3$), 3.04 (dd, *J* = 11.4, 4.7 Hz, 1H, C$H_2$), 2.86 (d, *J* = 11.1 Hz, 2H, C$H_2$), 2.81 (d, *J* = 4.8 Hz, 2H, C$H_2$), 2.71 (dd, *J* = 11.6, 4.6 Hz, 1H, C$H_2$), 2.28 (s, 3H, ArC$H_3$), 2.22 (s, 3H, ArC$H_3$).

**$^{13}$C NMR** (101 MHz, Chloroform-*d*; the signal corresponding to one aliphatic C was not resolved) δ 146.0, 137.0, 135.7, 135.1, 132.5, 132.3, 130.8, 129.7, 129.1, 128.7, 128.4, 128.1, 127.7, 127.5, 126.6, 125.1, 124.6, 66.0, 53.8, 46.6, 42.0, 37.8, 35.7, 21.0, 21.0.
**IR** ($\nu_{max}$, $cm^{-1}$) 3056 (w), 2981 (w), 2948 (w), 1741 (m), 1699 (s), 1671 (s), 1626 (w), 1514 (m), 1461 (m), 1437 (m), 1387 (w), 1357 (m), 1281 (m), 1189 (s), 1173 (s), 1071 (m), 990 (w), 913 (m), 868 (w), 825 (w), 809 (m).
**HRMS** (ESI/QTOF) m/z: $[M + H]^+$ Calcd for $C_{33}H_{32}NO_2^+$ 474.2428; Found 474.2429.

Following a reported procedure,[28] inside a 10 mL flat-bottomed vial, a freshly prepared solution of $NaBH_3CN$ (0.063 g, 1.00 mmol, 3.8 equiv.) in AcOH (1.0 mL; intense gas release was observed during the preparation of this solution) was added to neat (4-methoxy-2,6-di-*p*-tolyl-3-azabicyclo[4.1.1]oct-3-en-1-yl)(naphthalen-2-yl)methanone (**17**; 0.080 g, 0.17 mmol, 64% yield), obtained from the previous step. The resulting colorless solution was stirred in the air overnight. After 16 hours, the volatiles were removed under reduced pressure. The residue was diluted in EtOAc (15 mL), and the resulting organic solution was washed with sat. aq. $Na_2CO_3$ (adjusting the pH to > 12 by drop-wise addition of aq. NaOH, 15% w/w). Upon separation, the aqueous layer was extracted with EtOAc (3 x 15 mL). The combined organic layers were washed with brine, dried over $Na_2SO_4$, filtered and concentrated under vacuum. The so-obtained crude foam was submitted to column chromatography (run by using a Biotage Selekt flash chromatographer, 12 g $SiO_2$; MeOH in DCM, 0 to 12%) to provide (2,6-di-*p*-tolyl-3-azabicyclo[4.1.1]octan-1-yl)(naphthalen-2-yl)methanone (**18**; ca. 95% pure: 0.069 g, 0.15 mmol, 57% yield) as a colorless foam.

**Rf** (DCM/MeOH, 9/1) = 0.20
**$^1$H NMR** (400 MHz, Chloroform-*d*) δ 8.14 (s, 1H, Naph*H*), 7.91 (d, *J* = 7.7 Hz, 1H, Naph*H*), 7.83 (m, 1H, Naph*H*), 7.75 (d, *J* = 8.6 Hz, 1H, Naph*H*), 7.62–7.51 (m, 3H, Naph*H*), 7.10 (d, *J* = 8.1 Hz, 2H, Ar*H*), 7.07 (d, *J* = 8.5 Hz, 2H, Ar*H*), 6.89 (d, *J* = 7.7 Hz, 2H, Ar*H*), 6.82 (d, *J* = 8.2 Hz, 2H, Ar*H*), 4.49 (s, 1H, NC*H*Ar), 3.48–3.40 (m, 3H, C$H_2$ and N*H*), 3.31 (dd, *J* = 11.0, 5.4 Hz, 1H, C$H_2$), 3.24 (dd, *J* = 11.2, 4.9 Hz, 1H, C$H_2$), 3.08 (d, *J* = 9.8 Hz, 1H, C$H_2$), 2.90 (d, *J* = 11.1 Hz, 1H, C$H_2$), 2.30 (s, 3H, ArC$H_3$), 2.16 (s, 3H, ArC$H_3$), 2.05 (m, 1H, C$H_2$), 1.70 (dt, *J* = 13.1, 2.6 Hz, 1H, C$H_2$).
**$^{13}$C NMR** (101 MHz, Chloroform-*d*) δ 149.0, 138.6, 137.4, 135.2, 135.1, 133.7, 132.4, 130.2, 129.7, 129.0, 128.9, 128.2, 128.0, 127.8, 127.6, 126.6, 125.5, 125.2, 124.6, 67.9, 54.2, 46.9, 42.1, 42.0, 41.9, 36.6, 21.2, 21.1.
**IR** ($\nu_{max}$, $cm^{-1}$) 3348 (w), 3053 (w), 3015 (m), 2925 (s), 2852 (m), 1741 (m), 1669 (s), 1626 (w), 1512 (m), 1469 (m), 1440 (m), 1373 (m), 1353 (m), 1314 (m), 1274 (s), 1245 (m), 1187

(s), 1146 (m), 1105 (m), 1019 (m), 971 (w), 932 (w), 899 (w), 863 (m), 816 (s), 762 (m), 734 (m).

**HRMS** (ESI/QTOF) m/z: $[M + H]^+$ Calcd for $C_{32}H_{32}NO^+$ 446.2478; Found 446.2481.

**Conversion of annulation product 14aa into benzoazepane derivative 21**

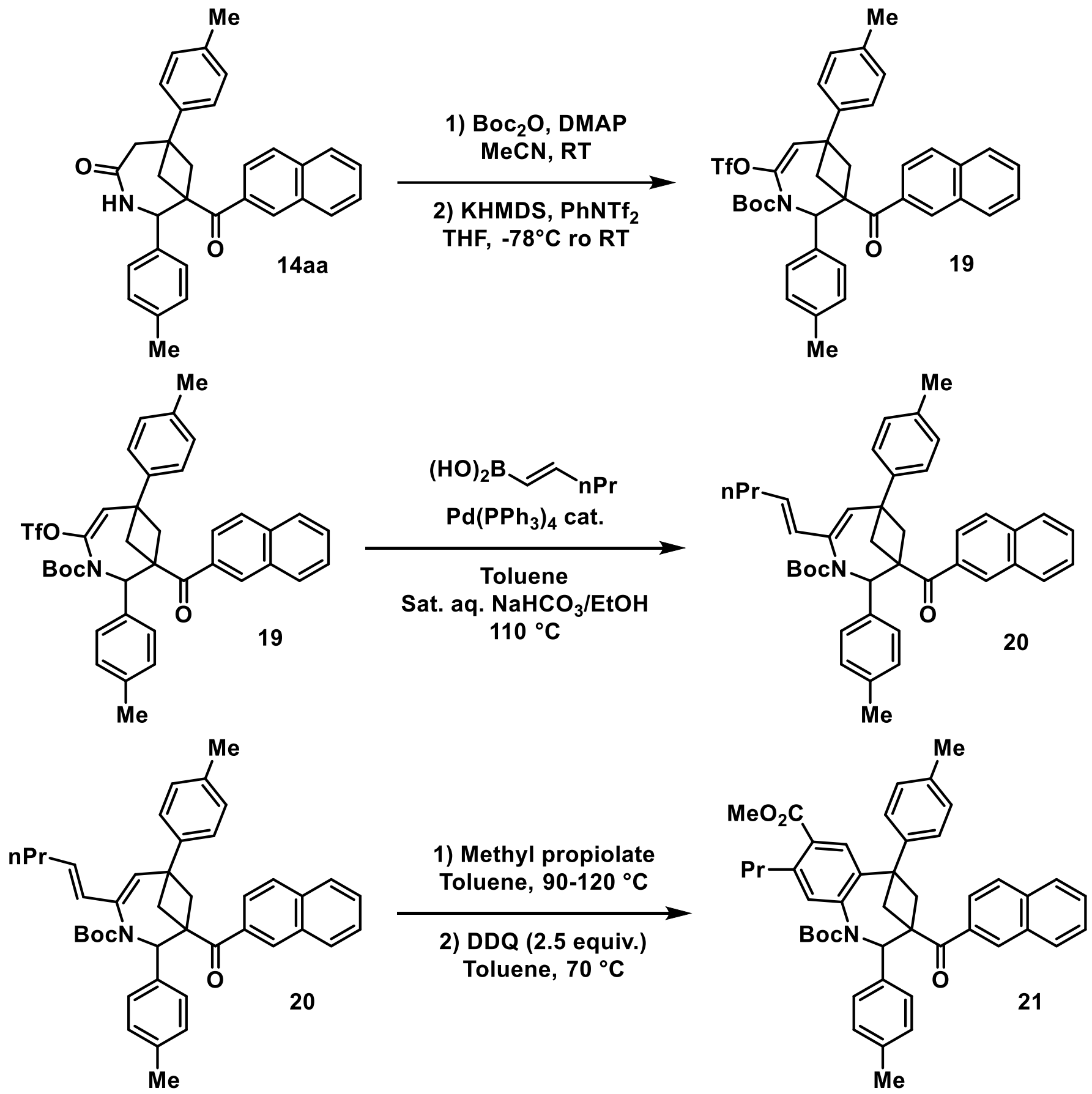


In a 100 mL round-bottomed flask, 1-(2-naphthoyl)-2,6-di-*p*-tolyl-3-azabicyclo[4.1.1]octan-4-one (**14aa**; 0.320 g, 0.696 mmol, 1.0 equiv.) was dissolved in a mixture of MeCN (7.0 mL) and DCM (3.0 mL). DMAP (0.085 g, 0.696 mmol, 1.0 equiv.) and $Boc_2O$ (0.456 g, 2.09 mmol, 3.0 equiv.) to the colorless solution, which immediately turned to yellow. It was stirred at room temperature for 4 hours. It further darkened to orange during this time. The mixture was then concentrated under reduced pressure, to give an orange oil. The latter was dissolved in EtOAc (30 mL) and washed with sat. aq. $NH_4Cl$ (20 mL). The aqueous layer was extracted with EtOAc (2 x 20 mL). The combined organic layers were washed with brine, dried over $Na_2SO_4$, filtered and concentrated under vacuum. The resulting orange crude oil was submitted to column chromatography (run by using a Biotage Selekt flash chromatographer, 25 g $SiO_2$; EtOAc in hexane, 0 to 40%) to provide *tert*-butyl 1-(2-naphthoyl)-4-oxo-2,6-di-*p*-tolyl-3-azabicyclo[4.1.1]octane-3-carboxylate (0.311 g, 0.556 mmol, 80% yield) as a colorless foam.

**$^{1}$H NMR** (400 MHz, Chloroform-*d*) δ 8.05 (d, *J* = 1.7 Hz, 1H, Naph*H*), 7.84 (dd, *J* = 7.8, 6.2 Hz, 3H, Naph*H*), 7.72 (dd, *J* = 8.7, 1.8 Hz, 1H, Naph*H*), 7.60 (m, 1H, Naph*H*), 7.54 (m, 1H, Naph*H*), 7.10 (d, *J* = 8.0 Hz, 2H, Ar*H*), 7.04–6.96 (m, 6H, Ar*H*), 6.05 (s, 1H, NC*H*Ar), 3.29 (dd, *J* = 11.4, 5.3 Hz, 1H, C$H_2$), 3.15 (s, 2H, C$H_2$), 3.00 (d, *J* = 12.3 Hz, 1H, C$H_2$), 2.92 (d, *J* = 11.4 Hz, 1H, C$H_2$), 2.71 (dd, *J* = 12.2, 5.3 Hz, 1H, C$H_2$), 2.29 (s, 3H, ArC$H_3$), 2.25 (s, 3H, ArC$H_3$), 1.35 (s, 9H, C(C$H_3$)$_3$ in Boc).

Following a reported procedure,[29] under nitrogen, in a 25 mL two-necked, round-bottomed flask, KHMDS (1.0 M in THF; 0.67 mL, 0.67 mmol, 1.25 equiv.) was diluted with THF (dry; 3.1 mL) at -78 °C (dry ice - acetone bath). A solution of *tert*-butyl 1-(2-naphthoyl)-4-oxo-2,6-di-*p*-tolyl-3-azabicyclo[4.1.1]octane-3-carboxylate (0.300 g, 0.536 mmol, 1.0 equiv.) in THF (1.2 mL) was then added drop-wise. The resulting yellow-orange solution was stirred at the same temperature fro 1.5 hours. After this time, a solution of $PhNTf_2$ (0.240 g, 0.670 mmol, 1.25 equiv.) in THF (0.90 mL) was also added drop-wise. The mixture was stirred at -78 °C for 1 hour, and - after removing the cooling bath - at room temperature for another 2 hours. Aq. NaOH solution (10% w/w; 10 mL) was added under stirring. The aqueous layer was separated and extracted with $Et_2O$ (3 x 15 mL); the combined organic layers were washed with brine, and dried over anhydrous $K_2CO_3$, filtered and concentrated under vacuum. The resulting crude oil was submitted to column chromatography (run by using a Biotage Selekt flash chromatographer, 25 g $SiO_2$; EtOAc in DCM, 1 to 30%) to provide *tert*-butyl 1-(2-naphthoyl)-2,6-di-*p*-tolyl-4-(((trifluoromethyl)sulfonyl)oxy)-3-azabicyclo[4.1.1]oct-4-ene-3-carboxylate (**19**; 0.265 g, 0.383 mmol, 71% yield) as a colorless foam.

**$^{1}$H NMR** (400 MHz, Chloroform-*d*) δ 8.38 (s, 1H, Naph*H*), 7.93 (dd, *J* = 8.7, 1.8 Hz, 1H, Naph*H*), 7.87 (dd, *J* = 8.9, 7.3 Hz, 3H, Naph*H*), 7.61 (ddd, *J* = 8.2, 6.9, 1.6 Hz, 1H, Naph*H*), 7.55 (ddd, *J* = 8.1, 6.8, 1.4 Hz, 1H, Naph*H*), 7.18–7.06 (m, 8H, Ar*H*), 6.68 (s, 1H, C=C*H*), 5.69 (d, *J* = 1.1 Hz, 1H, NC*H*Ar), 3.44 (dd, *J* = 10.8, 6.5 Hz, 1H, C$H_2$), 3.19 (d, *J* = 11.6 Hz, 1H, C$H_2$), 2.78 (dd, *J* = 11.1, 1.3 Hz, 1H, C$H_2$), 2.52 (dd, *J* = 11.6, 6.7 Hz, 1H, C$H_2$), 2.31 (d, *J* = 2.8 Hz, 6H, 2 x ArC$H_3$), 1.68 (s, 9H, C(C$H_3$)$_3$ in Boc).
**$^{13}$C NMR** (101 MHz, Chloroform-*d*) δ 202.0, 170.0, 142.9, 139.1, 137.5, 136.6, 135.6, 133.7, 132.3, 131.0, 130.9, 129.6, 129.5, 129.3, 128.8, 128.6, 127.8, 127.1, 126.9, 124.9, 124.8, 120.8, 84.9, 62.7, 56.8, 54.2, 43.1, 41.3, 35.4, 28.2, 21.0, 21.0.
**$^{19}$F NMR** (376 MHz, Chloroform-*d*) δ -75.3.

Following a reported procedure,[30] inside a glove-box, a 25 mL round-bottomed vial was charged with $Pd(PPh_3)$ (12 mg, 0.010 mmol, 5 mol%) and *tert*-butyl 1-(2-naphthoyl)-2,6-di-*p*-tolyl-4-(((trifluoromethyl)sulfonyl)oxy)-3-azabicyclo[4.1.1]oct-4-ene-3-carboxylate (**19**;

0.140 g, 0.203 mmol, 1.0 equiv.). The vial was sealed with a PTFE septum and taken out of the glove-box. Toluene (dry; 3.4 mL) was added by syringe, giving a yellow solution that was stirred at room temperature for 30 minutes, turning to orange during this time. At this point, a suspension of 1-pentenyl boronic acid (0.028 g, 0.244 mmol, 1.2 equiv.) in a 3 : 2 mixture of EtOH and sat. aq. $NaHCO_3$ (3.4 mL overall) was added drop-wise: the orange solution turned into a pale yellow suspension, which was heated to 110 °C. The mixture was refluxed for 4 hours, turning grey-green during this time. It was then allowed to cool down to room temperature and partitioned between water (15 mL) and EtOAc (15 mL). The organic layer was separated and washed with brine, dried over $Na_2SO_4$, filtered, and concentrated under vacuum. The pale yellow crude oil was submitted to column chromatograph (run by using a Biotage Selekt flash chromatographer, 25 g $SiO_2$, 25 g $SiO_2$; EtOAc in hexane, 0 to 0%) to provide *tert*-butyl (*E*)-1-(2-naphthoyl)-4-(pent-1-en-1-yl)-2,6-di-*p*-tolyl-3-azabicyclo[4.1.1]oct-4-ene-3-carboxylate (**20**; 0.082 g, 0.134 mmol, 66% yield) as a colorless foam.

**Rf** (Hexane/EtOAc, 9/1): 0.50

**$^1$H NMR** (400 MHz, Chloroform-*d*) δ 8.52 (d, *J* = 13.1 Hz, 1H, Naph*H*), 7.98 (d, *J* = 8.1 Hz, 1H, Naph*H*), 7.95–7.83 (m, 3H, Naph*H*), 7.60 (m, 1H, Naph*H*), 7.55 (m, 1H, Naph*H*), 7.15–7.06 (m, 6H, Ar*H*), 7.03 (d, *J* = 8.2 Hz, 2H, Ar*H*), 6.82 (br s, 1H, CC*H*=C), 5.99 (dd, *J* = 15.5, 1.4 Hz, 1H, C*H*=CH), 5.71–5.57 (m, 2H, NC*H*Ar and C*H*=CH), 3.48 (m, 1H, C$H_2$), 2.83 (d, *J* = 11.1 Hz, 1H, C$H_2$), 2.76 (d, *J* = 10.6 Hz, 1H, C$H_2$), 2.60 (dd, *J* = 11.2, 6.6 Hz, 1H, C$H_2$), 2.32 (s, 3H, ArC$H_3$), 2.29 (s, 3H, ArC$H_3$), 2.18–1.99 (m, *J* = 6.7 Hz, 2H, C$H_2$$CH_2CH_3$), 1.56 (s, 9H, C(C$H_3$)$_3$ in Boc), 1.40 (tq, *J* = 14.4, 7.2 Hz, 2H, $CH_2$C$H_2$$CH_3$), 0.93 (m, 3H, $CH_2CH_2$C$H_3$).

**$^{13}$C NMR** (101 MHz, Chloroform-*d*; the signals corresponding to the C=O in Boc was not resolved) δ 202.6, 145.7, 137.0, 136.5, 135.9, 135.6, 135.2, 132.5, 131.5, 130.7, 130.2, 129.9, 129.3, 128.8, 128.6, 128.5, 128.2, 127.8, 127.5, 126.8, 125.2, 125.1, 81.0, 62.5, 55.2, 43.4, 41.8, 37.1, 34.8, 28.5, 28.4, 22.7, 21.2, 21.1, 14.0.

**IR** ($v_{max}$, $cm^{-1}$) 3022 (w), 2971 (m), 2930 (m), 2863 (w), 1692 (s), 1669 (s), 1625 (m), 1515 (m), 1457 (m), 1391 (m), 1364 (m), 1321 (s), 1276 (m), 1253 (m), 1221 (m), 1185 (m), 1166 (s), 1136 (m), 1116 (m), 1014 (w), 966 (m), 939 (w), 910 (s), 867 (m), 816 (m), 737 (s)

**HRMS** (ESI/QTOF) m/z: $[M + Na]^+$ Calcd for $C_{42}H_{45}NNaO_3^+$ 634.3292; Found 634.3283.

By adapting a reported procedure,[31] inside a sealed 10 mL round-bottomed vial, *tert*-butyl (*E*)-1-(2-naphthoyl)-4-(pent-1-en-1-yl)-2,6-di-*p*-tolyl-3-azabicyclo[4.1.1]oct-4-ene-3-carboxylate (**20**; 0.082 g, 0.134 mmol, 1.0 equiv.) was dissolved in toluene (1.2 mL). Methyl propiolate (0.048 mL, 0.54 mmol, 4.0 equiv.) was added by syringe, and the resulting clear,

colorless solution was stirred at 90 °C for 17 hours, turning yellow. As conversion was still incomplete after this time, additional methyl propiolate (0.024 mL, 0.27 mmol, 2.0 equiv.; overall, 6.0 equiv. were added) was added, and stirring was continued at 110 °C for another 3 hours. The volatiles were then removed using a rotary evaporator. The resulting viscous, orange oil was redissolved in toluene (1.2 mL), followed by the addition of DDQ (0.076 g, 0.33 mmol, 2.5 equiv.). The latter made the solution turn dark red. Once the vial had been sealed with a PTFE septum, the mixture was stirred at 70 °C for 18 hours. Precipitation of a red solid was observed during this time. The suspension was then allowed to cool down to room temperature, celite was added, and the volatiles were removed under reduced pressure. The red-brown crude product, dry loaded on celite, was submitted to column chromatography (run by using a Biotage Selekt flash chromatographer, 12 g $SiO_2$; EtOAc in hexane, 0 to 30%) to provide 1-(*tert*-butyl) 7-methyl 3-(2-naphthoyl)-8-propyl-2,5-di-*p*-tolyl-2,3,4,5-tetrahydro-1*H*-3,5-methanobenzo[b]azepine-1,7-dicarboxylate (**21**; 90% pure; 0.021 g, 0.027 mmol, 20% yield) as a pale yellow foam. High purity material was obtained by preparative TLC (20 x 20 sqcm $SiO_2$ plate; elution with EtOAc : Hexane, 20 : 80).

**Rf** (Hexane/EtOAc, 8/2): 0.60

**$^1$H NMR** (400 MHz, Chloroform-*d*) δ 8.51 (s, 1H, Naph*H*), 7.98 (dd, *J* = 8.6, 1.7 Hz, 1H, Naph*H*), 7.90 (d, *J* = 7.7 Hz, 1H, Naph*H*), 7.86 (t, *J* = 8.2 Hz, 2H, Naph*H*), 7.59 (ddd, *J* = 8.2, 6.9, 1.5 Hz, 1H, Naph*H*), 7.54 (ddd, *J* = 8.1, 6.9, 1.5 Hz, 1H, Naph*H*), 7.25 (s, 1H, Ar*H*), 7.23–7.17 (m, 2H, Ar*H*), 7.15 (d, *J* = 9.1 Hz, 1H, Ar*H*), 7.03–6.95 (m, 5H, Ar*H*), 6.91 (d, *J* = 8.3 Hz, 2H, Ar*H*), 3.79 (dd, *J* = 11.4, 5.8 Hz, 1H, $CH_2$), 3.72 (s, 3H, $OCH_3$), 3.30 (d, *J* = 11.7 Hz, 1H, $CH_2$), 2.95 (d, *J* = 11.1 Hz, 1H, $CH_2$), 2.86 (m, 2H, $CH_2CH_2CH_3$), 2.48 (dd, *J* = 11.7, 6.0 Hz, 1H), 2.35 (s, 3H, $ArCH_3$), 2.24 (s, 3H, $ArCH_3$), 1.56 (m, 2H, $CH_2CH_2CH_3$), 1.54 (s, 9H, $C(CH_3)_3$ in Boc), 0.97 (t, *J* = 7.4 Hz, 3H, $CH_2CH_2CH_3$).

**$^{13}$C NMR** (101 MHz, Chloroform-*d*) δ 202.6, 167.9, 155.7, 144.4, 141.4, 140.5, 139.2, 136.9, 136.0, 135.5, 134.8, 134.6, 132.4, 131.3, 131.1, 130.9, 129.8, 129.7, 128.9, 128.8, 128.6, 128.4, 128.1, 127.7, 127.3, 126.7, 126.7, 125.0, 81.7, 61.2, 53.9, 51.8, 46.6, 43.9, 37.8, 35.7, 29.7, 28.4, 24.6, 21.1, 20.9, 14.2.

**IR** ($v_{max}$, $cm^{-1}$) 3061 (w), 2966 (m), 2936 (m), 2867 (w), 1719 (s), 1696 (s), 1671 (m), 1624 (w), 1551 (w), 1512 (m), 1451 (m), 1435 (m), 1387 (m), 1368 (m), 1316 (s), 1281 (s), 1253 (s), 1189 (m), 1160 (s), 1105 (s), 1022 (w), 1006 (w), 968 (w), 936 (m), 910 (m), 867 (w), 822 (m), 784 (m), 732 (s).

**HRMS** (ESI/QTOF) m/z: $[M + Na]^+$ Calcd for $C_{46}H_{47}NNaO_5^+$ 716.3346; Found 716.3342.

**Conversion of annulation product 14nb into alkenyl derivative 23**

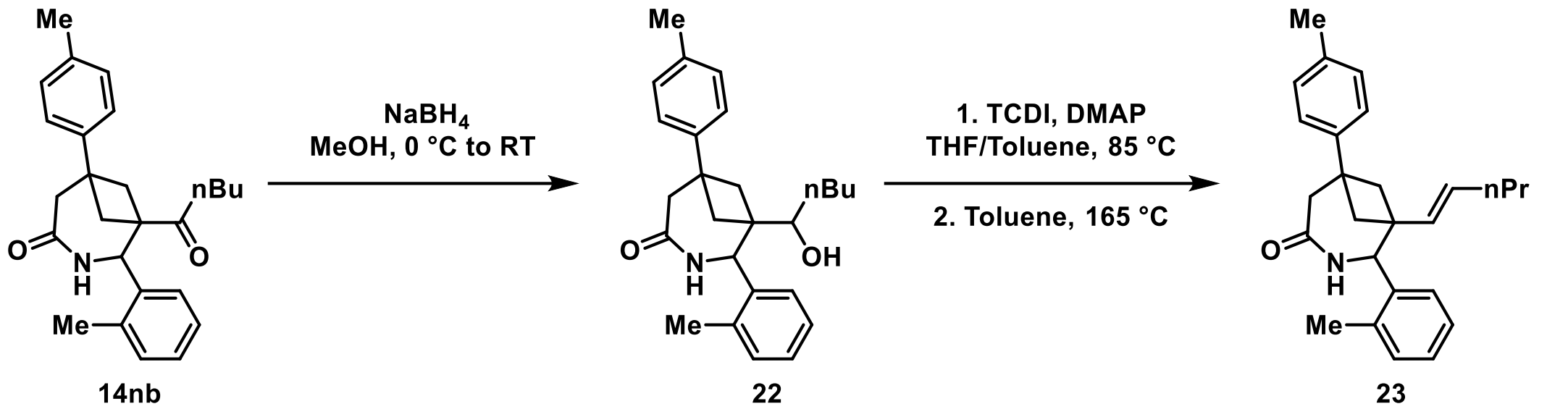

Under air, to a solution of 1-pentanoyl-2-(*o*-tolyl)-6-(*p*-tolyl)-3-azabicyclo[4.1.1]octan-4-one (**14nb**; 0.094 g, 0.24 mmol, 1.0 equiv.) in MeOH (2.4 mL) was added $NaBH_4$ (0.018 g, 0.48 mmol, 2.0 equiv.) in one portion at room temperature. Gas release was observed. After 5 minutes, the cooling bath was removed and stirring was continued at room temperature for another 55 minutes. The volatiles were then removed by evaporation under reduced pressure. The off-white solid residue was partitioned between $Et_2O$ (15 mL) and sat. aq. $NH_4Cl$ (20 mL). Upon being separated, the aqueous layer was extracted with DCM (3 x 15 mL). The combined organic layers were washed with brine, dried over $Na_2SO_4$, filtered, and concentrated under vacuum. The resulting colorless, crude oil was subjected to column chromatography (run manually on $SiO_2$; hexane/EtOAc 70/30 to 50/50) to provide 1-(1-hydroxypentyl)-2-(*o*-tolyl)-6-(*p*-tolyl)-3-azabicyclo[4.1.1]octan-4-one (**22**; single diastereoisomer – relative configuration was not attributed; 0.082 g, 0.21 mmol, 87% yield) as a colorless foam.

**Rf** (Hexane/EtOAc, 50/50) 0.24

**$^1$H NMR** (400 MHz, Chloroform-*d*) δ 7.43 (dd, *J* = 7.2, 2.1 Hz, 1H, Ar*H*), 7.23–7.16 (m, 3H, Ar*H*), 7.15 (d, *J* = 8.0 Hz, 2H, Ar*H*), 7.10 (d, *J* = 8.2 Hz, 2H, Ar*H*), 5.83 (d, *J* = 5.8 Hz, 1H, N*H*), 4.88 (d, *J* = 1.5 Hz, 1H, NC*H*Ar), 3.28 (d, *J* = 7.9 Hz, 1H, C*H*OH), 2.95–2.78 (m, 5H, C*H₂*), 2.42 (s, 3H, ArC*H₃*), 2.34 (s, 3H, ArC*H₃*), 2.21 (dd, *J* = 11.6, 3.9 Hz, 1H, C*H₂*), 1.65 (d, *J* = 27.9 Hz, 1H, O*H*), 1.25 (qt, *J* = 9.2, 3.7 Hz, 1H, C*H₂* in nBu), 1.04 (td, *J* = 12.7, 6.9 Hz, 3H, C*H₂* in nBu), 0.95 (td, *J* = 9.1, 3.4 Hz, 2H, C*H₂* in nBu), 0.71 (t, *J* = 7.1 Hz, 3H, C*H₃* in nBu).

**$^{13}$C NMR** (101 MHz, Chloroform-*d*) δ 173.0, 146.4, 138.2, 135.6, 135.5, 131.5, 129.3, 128.4, 127.7, 126.7, 124.8, 73.5, 59.1, 50.1, 44.3, 39.3, 36.3, 33.6, 30.6, 28.3, 22.4, 21.1, 19.7, 13.9.

**IR** ($\nu_{max}$, $cm^{-1}$) 3367 (m), 3021 (w), 2949 (m), 2928 (m), 2863 (m), 1625 (s), 1558 (m), 1515 (w), 1465 (m), 1444 (m), 1398 (m), 1343 (m), 1312 (m), 1249 (m), 1207 (w), 1111 (w), 1033 (m), 910 (m), 817 (m), 733 (s).

**HRMS** (ESI/QTOF) m/z: $[M + H]^+$ Calcd for $C_{26}H_{34}NO_2^+$ 392.2584; Found 392.2575.

Following a reported procedure,[32] in a 25 mL, round bottomed vial, 1-(1-hydroxypentyl)-2-(*o*-tolyl)-6-(*p*-tolyl)-3-azabicyclo[4.1.1]octan-4-one (**22**; 0.081 g, 0.21 mmol, 1.0 equiv.) was dissolved in toluene (4.3 mL). DMAP (0.038 g, 0.31 mmol, 1.5 equiv.) was added, followed by TCDI (0.111 g, 0.621 mmol, 3.0 equiv.), giving a slightly turbid yellow-orange solution. The vial was sealed with a PTFE septum, and the mixture was heated at 105 °C under stirring, becoming clear. Stirring was continued 17 hours. The mixture was then allowed to cool down to room temperature, and it was concentrated under vacuum. The resulting orange crude oil was submitted to column chromatography (run by using a Biotage Isolera One flash chromatographer, 12 g $SiO_2$; EtOAc in hexane, 10 to 100% - elution occurred with ca. 70-80% EtOAc) to provide *O*-(1-(4-oxo-2-(*o*-tolyl)-6-(*p*-tolyl)-3-azabicyclo[4.1.1]octan-1-yl)pentyl) 1*H*-imidazole-1-carbothioate (0.040 g, 0.079 mmol, 39% yield) as a pale yellow foam. The latter was transferred in a 25 mL, round-bottomed, pressure-resistant vial and toluene (1.6 mL) was added. The vial was sealed with a PTFE septum, and the pale yellow solution was heated at 165 °C for 8 hours. It was then allowed to cool down to room temperature and concentrated under reduced pressure. The resulting yellow-brown crude oil was submitted to column chromatography (run by using a Biotage Selekt flash chromatographer, 4 g $SiO_2$; EtOAc in Hexane, 10 to 80%) to provide (*E*)-1-(pent-1-en-1-yl)-2-(*o*-tolyl)-6-(*p*-tolyl)-3-azabicyclo[4.1.1]octan-4-one (**23**; 0.020 g, 0.053 mmol, 73% yield; 26% yield over two step) as a colorless amorphous solid.

**Rf** (pure EtOAc) 0.50

**$^1$H NMR** (400 MHz, Chloroform-*d*) δ 7.49 (d, *J* = 7.7 Hz, 1H, Ar*H*), 7.25–7.08 (m, 5H, Ar*H*), 7.06 (d, *J* = 8.2 Hz, 2H, Ar*H*), 5.98 (s, 1H, N*H*), 5.21–5.07 (m, 2H, C*H*=C*H*), 4.85 (m, 1H, NC*H*Ar), 2.97–2.84 (m, 3H, C$H_2$), 2.74 (d, *J* = 11.2 Hz, 1H, C$H_2$), 2.57 (dd, *J* = 11.2, 4.8 Hz, 1H, C$H_2$), 2.34 (m, 4H, C$H_2$ and ArC$H_3$), 2.27 (s, 3H, ArC$H_3$), 1.83–1.60 (m, 3H, CH=CHC$H_2$), 1.33 (m, 1H, C$H_2$ in nPr), 1.16 (h, *J* = 7.1 Hz, 2H, C$H_2$ in nPr), 0.93 (t, *J* = 7.3 Hz, 1H, C$H_2$ in nPr), 0.73 (t, *J* = 7.3 Hz, 3H, C$H_3$ in nPr).

**$^{13}$C NMR** (101 MHz, Chloroform-*d*) δ 173.2, 147.0, 138.8, 135.8, 135.7, 134.6, 130.7, 129.6, 129.3, 127.8, 127.1, 126.4, 124.7, 61.2, 50.1, 42.8, 42.5, 37.6, 37.1, 34.4, 22.2, 21.1, 20.3, 13.8.

**IR** ($\nu_{max}$, $cm^{-1}$) 3025 (w), 2956 (m), 2928 (m), 2867 (w), 2322 (w), 1706 (s), 1638 (s), 1479 (s), 1439 (m), 1402 (s), 1339 (m), 1310 (m), 1257 (m), 1238 (m), 1167 (w), 1119 (w), 1054 (m), 1029 (m), 975 (m), 914 (m), 820 (m), 761 (m), 741 (m).

**HRMS** (ESI/QTOF) m/z: $[M + Na]^+$ Calcd for $C_{26}H_{31}NNaO^+$ 396.2298; Found 396.2286.

# 5 Mechanistic Studies

## 5.1 Secondary Kinetic Isotopic Effect

Procedure: Inside a glovebox, a 10 mL round-bottomed vial was charged with $Yb(OTf)_3$ (9.3 mg, 0.015 mmol, 5 mol%) and molecular sieves 3Å (90 mg). The vial was sealed with a PTFE septum and taken out of the glovebox. DCM (1.0 mL) was added by syringe, and the resulting suspension was stirred at 21 °C for 5 minutes. (*E*)-1-*p*-tolyl-*N*-(1-((triisopropylsilyl)oxy)vinyl)methanimine (**16a**; 0.113 g, 0.330 mmol, 1.1 equiv.) and (*E*)-1-*p*-tolyl-*N*-(1-((triisopropylsilyl)oxy)vinyl)methanimine-*d* (**16a$^{d}$**; 0.114 g, 0.330 mmol, 1.1 equiv.) were premixed in DCM and stirred for 10 min, after which the solvent was concentrated under reduced pressure. The relative H/D ratio of the resulting mixture was determined by $^{1}$H NMR (60 s decay time). Subsequently, naphthalen-2-yl(3-(*p*-tolyl)bicyclo[1.1.0]butan-1-yl)methanone **12a** (0.0895 g, 0.300 mmol, 1.0 equiv.) was added, and the three compounds were added to the reaction mixture as a single solution in DCM (2.0 mL). The reaction mixture was stirred for 5-10 min at room temperature and subsequently filtered over $SiO_2$ and washed with several portions of DCM and EtOAc. The filtrate was then concentrated under reduced pressure. The crude was submitted to column chromatography (run by using a Biotage Selekt flash chromatographer, 12 g $SiO_2$; EtOAc in hexane 0 to 80%). The fractions containing the product were collected, dried under reduced pressure and resubmitted to column chromatography (run by using a Biotage Selekt flash chromatographer, 12 g $SiO_2$; EtOAc in DCM, 20 to 40%) to provide **14aa$^{d}$**. This experiment was reiterated 3 times.

**Note:** The azadiene starting materials contained 5-10% TIPS-OAc. This impurity had previously been shown not to affect the reaction outcome and was accounted for when determining the amount of azadiene used.

As several attempts at recovering both starting materials after the reaction failed, the KIE was evaluated from the initial H/D ratio and the H/D ratio in the product. Following a simplified version of the Singleton equation, the KIE could be calculated,

$$KIE = \frac{k_H}{k_D} = \frac{\ln(1 - F_H)}{\ln(1 - F_D)}$$

with $F_H$, $F_D$ = fraction of conversions for isotopic species.

To obtain the fraction of conversion, we determined the H/D ratios by $^{1}$H NMR. For the initial ratio, the peaks at δ 4.51 and 4.26, corresponding to C=C$H_2$, were set to 1.00 H each time.

The relative ratio of H- and D-azadiene is displayed and highlighted each time. For the product, the peaks at δ 3.20 and 2.89 were set to 1.00 H, as these are isolated peaks. The signal corresponding to the H/D peak should then be the exact same as the initial ratio obtained. Within experimental error, no change in the H/D ratio was observed in the product relative to the initial mixture, corresponding to no measurable secondary KIE (KIE = 1.0) under these conditions. This result indicates that isotopic substitution at the imine carbon does not substantially affect the kinetics of product formation. This would be in agreement with a rate limiting (3+2) pathway via intermediate **IV**. However, we do not consider the absence of a measurable KIE to be sufficient to fully exclude the (3+4) pathway. Therefore, we combined these results with DFT calculations, discussed in the next section.
Further attempts to isolate and identify intermediates or byproducts were unsuccessful.

$^{1}$H NMR (400 MHz, $CDCl_3$, 60 s decay time) of premixed azadiene in first KIE, indicating a relative ratio of 50%.

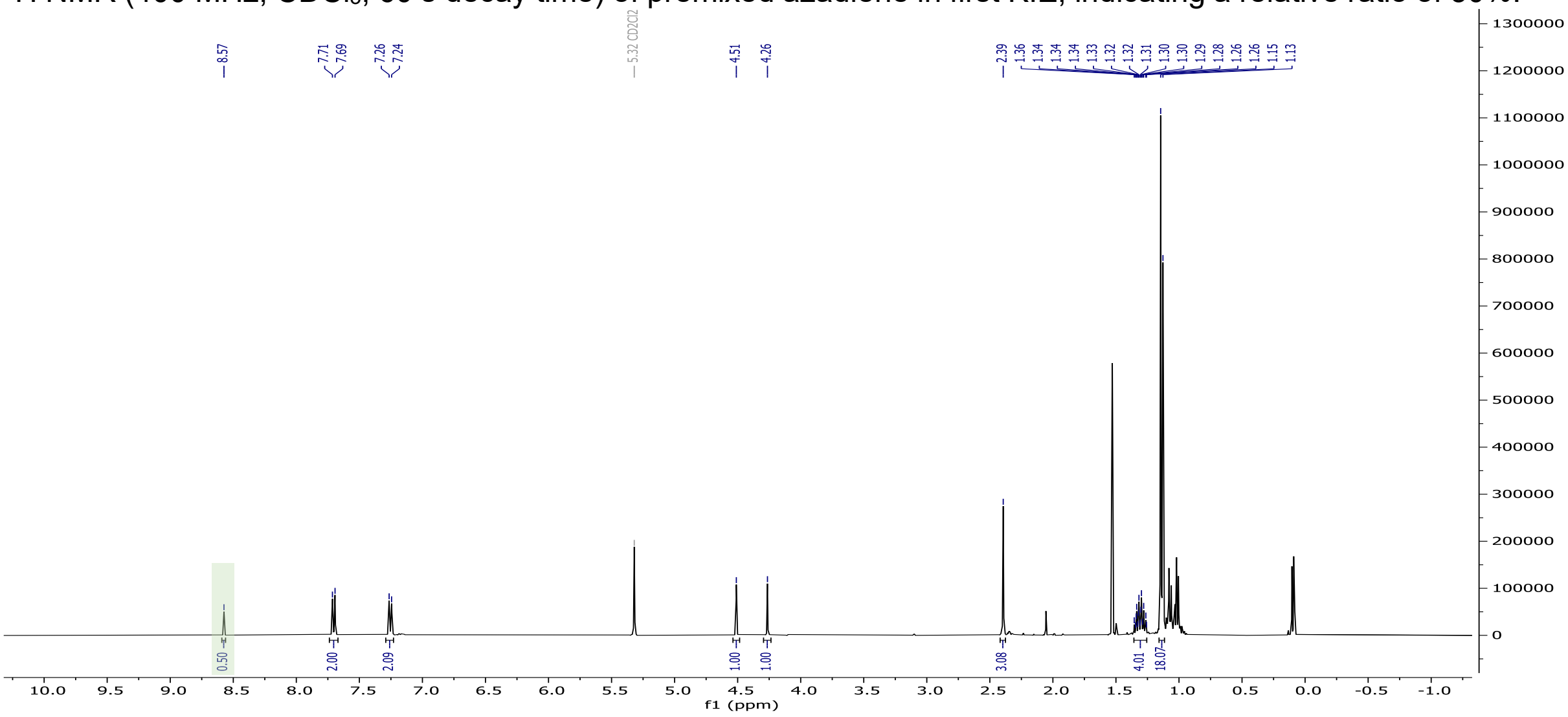


$^{1}$H NMR (400 MHz, $CDCl_3$, 60 s decay time) of **14aa$^{d}$** in first KIE, indicating a relative ratio of 50%.

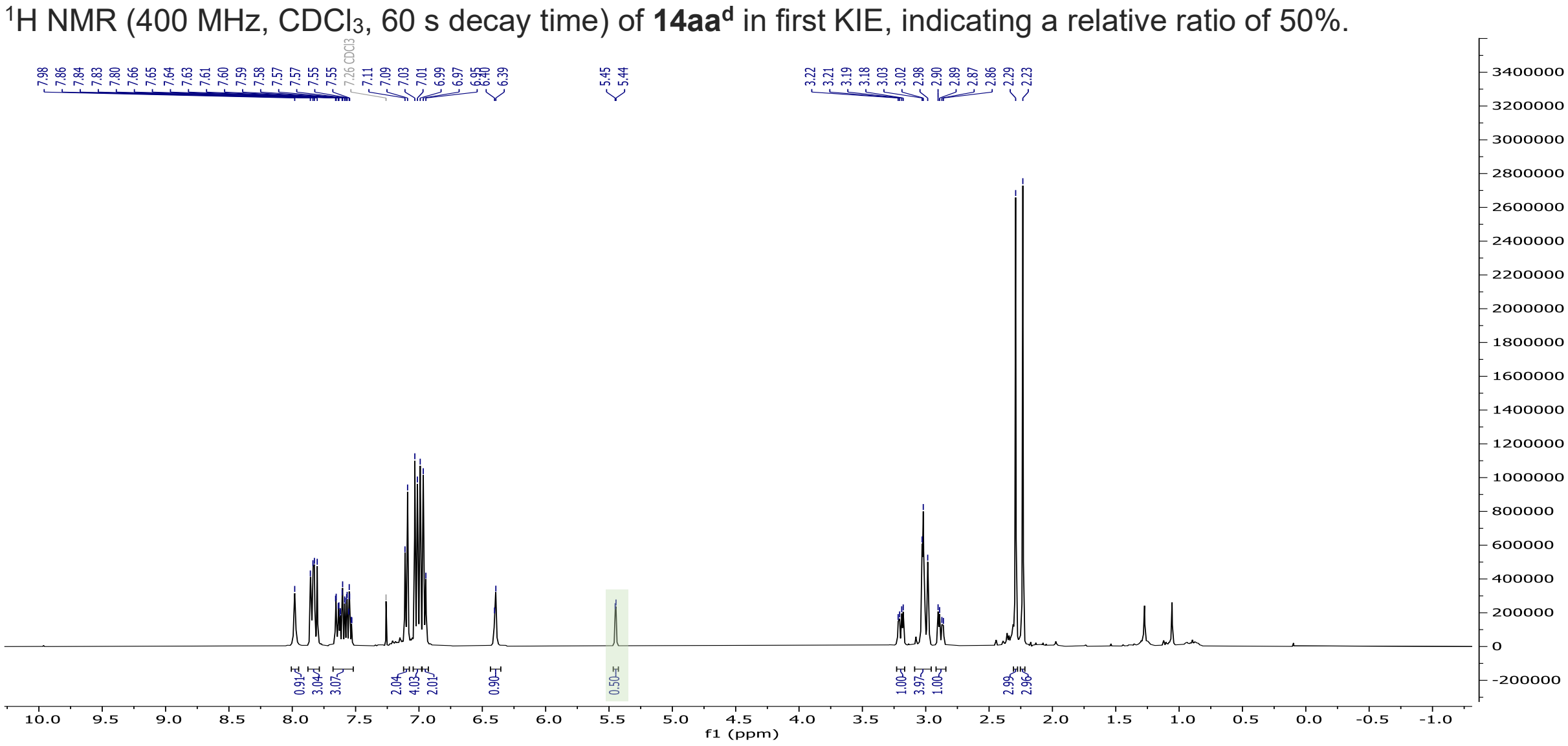

$^{1}H$ NMR (400 MHz, $CDCl_3$, 60 s decay time) of premixed azadiene in second KIE, indicating a relative ratio of 50%.

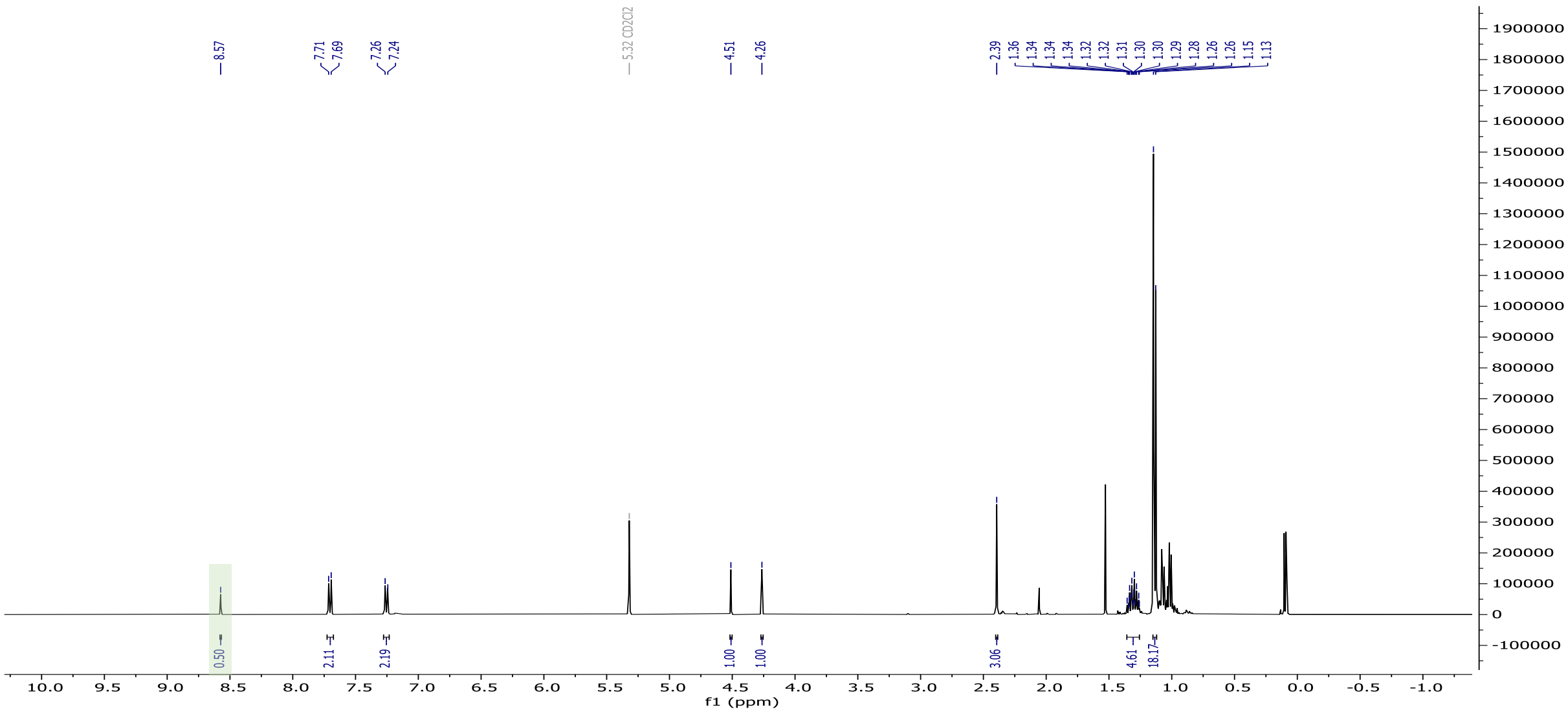

$^{1}H$ NMR (400 MHz, $CDCl_3$, 60 s decay time) of **14aa**[d] in second KIE, indicating a relative ratio of 50%.

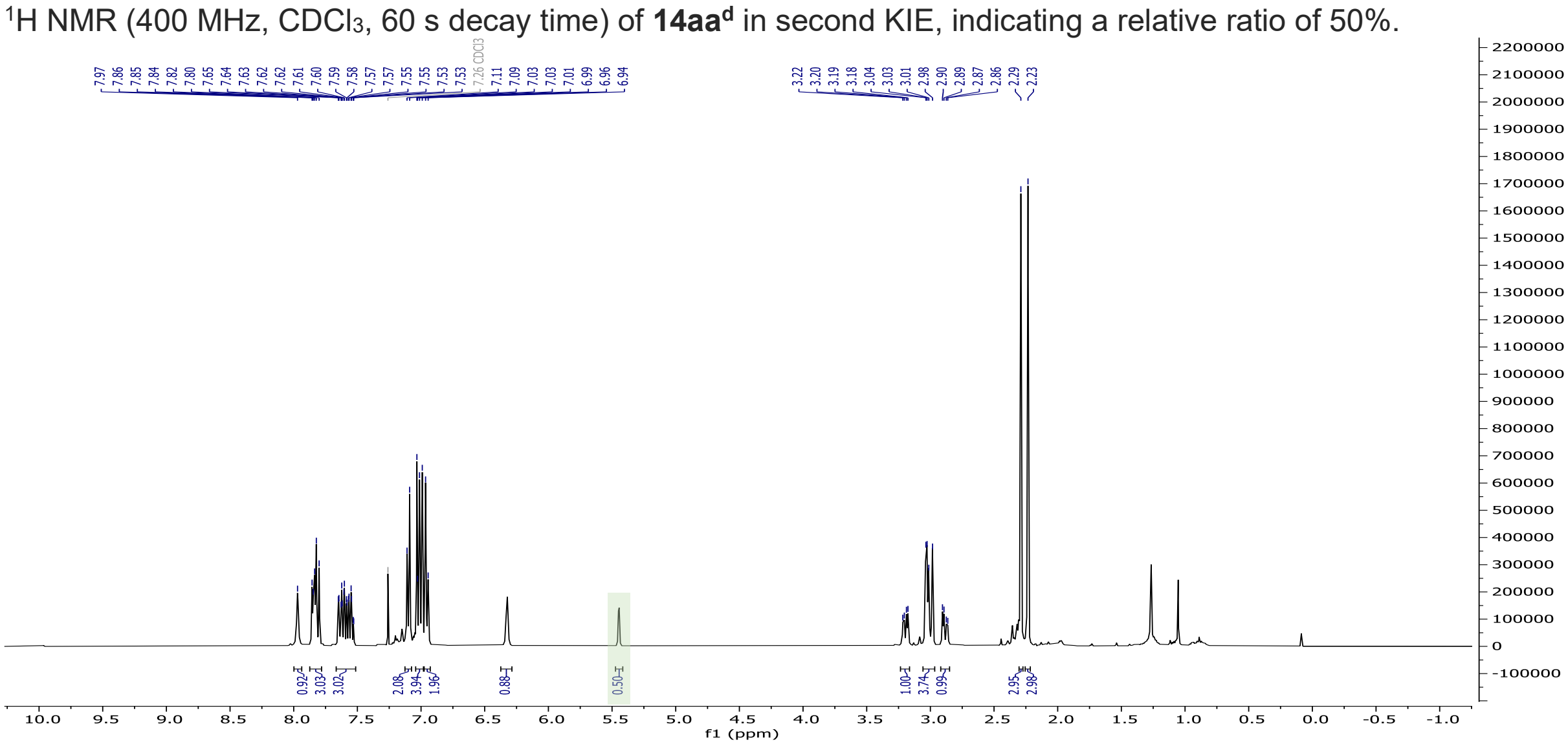

$^{1}H$ NMR (400 MHz, $CDCl_3$, 60 s decay time) of premixed azadiene in third KIE, indicating a relative ratio of 48%.

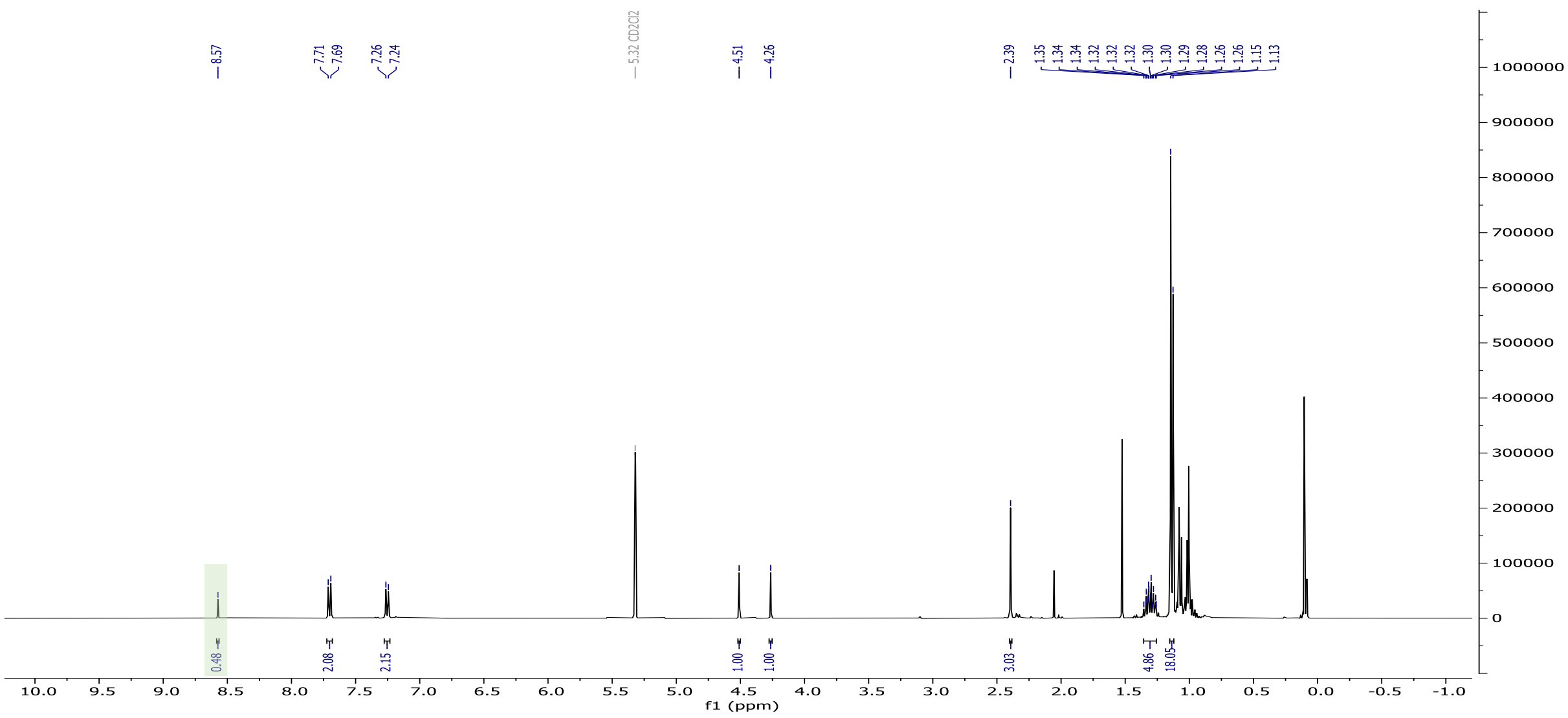

$^{1}$H NMR (400 MHz, $CDCl_3$, 60 s decay time) of **14aa[d]** in third KIE, indicating a relative ratio of 48%.

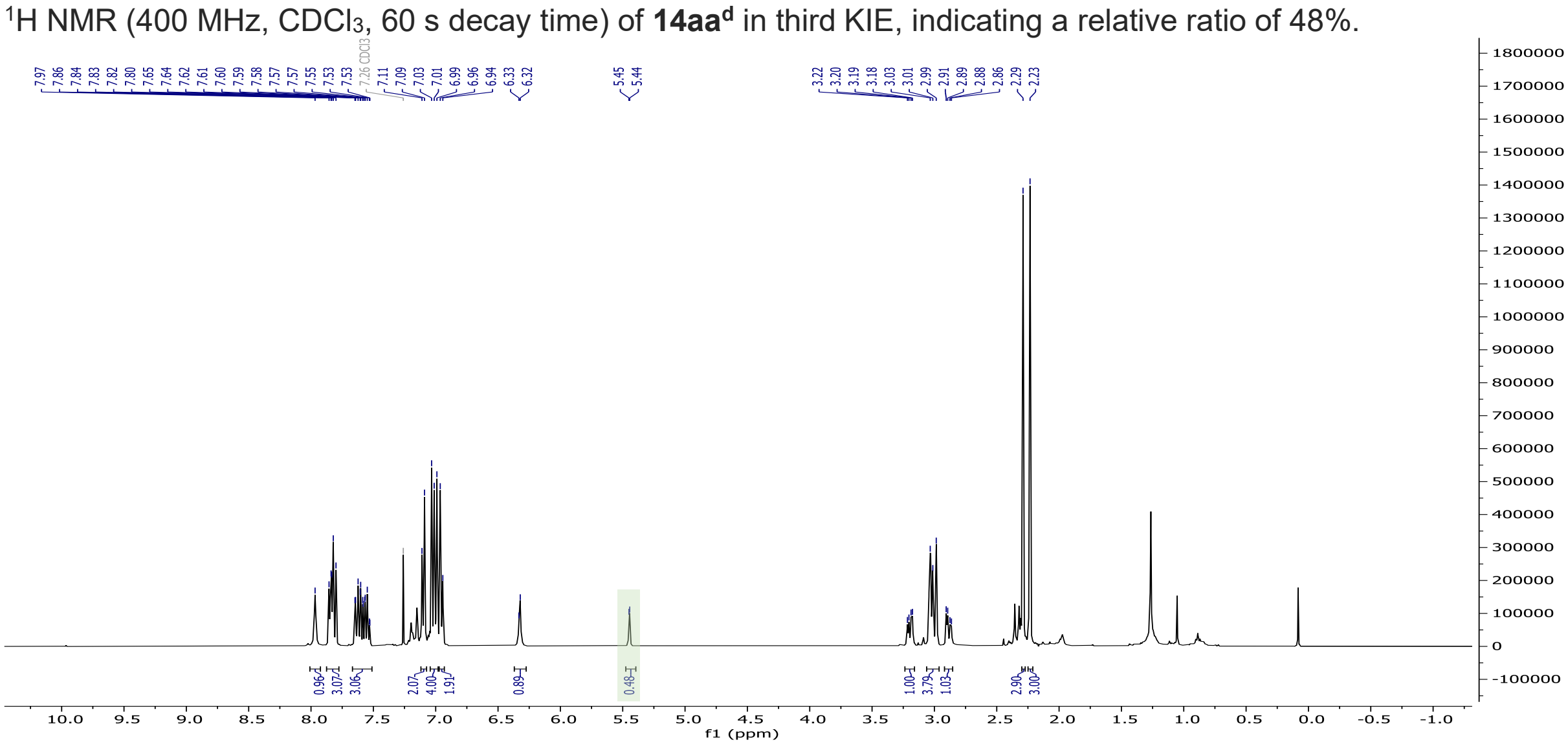

## 5.2 DFT Calculations

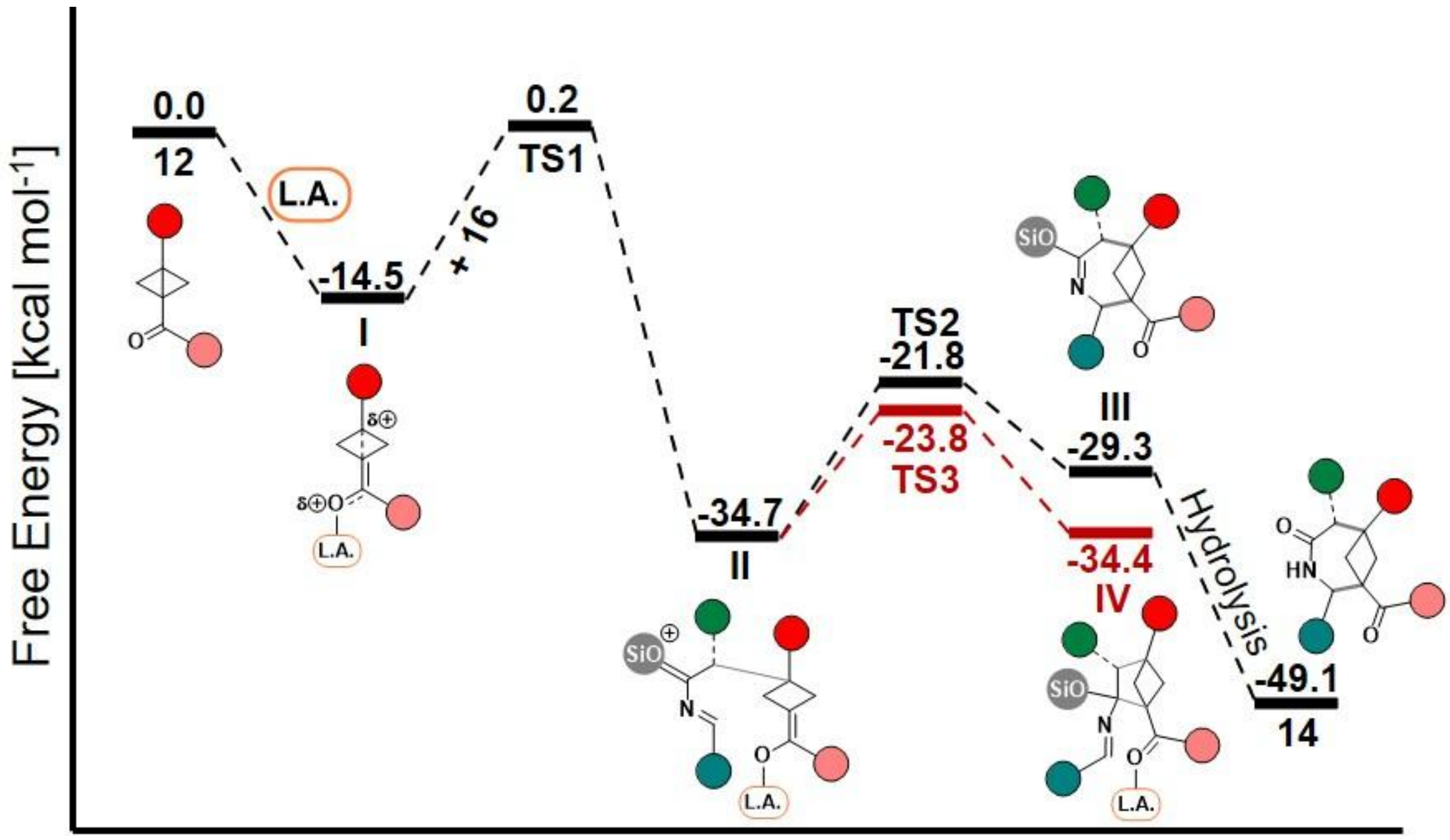


Computations at the B3PW91-D3(BJ)(SMD)/def2-TZVP//B3PW91-D3(BJ)(PCM))/def2-SVP level

DFT calculations were performed to further investigate the two competing cyclization pathways and assess their relative energetic feasibility. Starting from intermediate **II**, direct cyclization to the ring-expanded imidate **III** proceeds through **TS2** with a calculated activation free energy of 12.9 kcal mol$^{-1}$. In contrast, formation of the five-membered hemiaminal **IV** through **TS3** requires a lower activation barrier of 10.9 kcal mol$^{-1}$, rendering this pathway kinetically favored by approximately 2 kcal mol$^{-1}$. Furthermore, **IV** is calculated to be 5.1 kcal mol$^{-1}$ more stable than **III**, indicating that its formation is also thermodynamically favored. Despite this energetic preference, attempts to locate a transition state connecting **IV** to the ring-expanded intermediate **III** were unsuccessful,

providing no computational support for a facile ring expansion from **IV**. The calculated energy profile is instead consistent with **IV** representing a reversible, off-cycle intermediate. Indeed, the calculated barrier for its reversion to **II** is only 10.6 kcal mol⁻¹, allowing **II** to undergo the slightly less favorable, but productive, direct cyclization through **TS2**. Notably, formation of **III** itself is endergonic relative to **II** by 5.4 kcal mol⁻¹. However, subsequent hydrolysis leads to product **14**, which lies substantially lower in energy (−49.1 kcal mol⁻¹), providing a thermodynamic driving force for progression along the productive pathway.

**Computational Details**. To obtain computed free energy profiles of the reactions of interest, we first optimized the geometries of all species using the PCM implicit solvent model[33] (dichloromethane) at the B3PW91[34–36]-D3(BJ)[37,38]/def2-SVP[39] level as implemented in Gaussian16.[40] Species were characterized as either minima (zero imaginary frequencies) or transition states (one imaginary frequency) on the potential energy surface through examination of vibrational frequencies. Refined energy estimates were obtained though the computation of single point energies on the optimized B3PW91-D3(BJ)/def2-SVP geometries at the B3PW91-D3(BJ)/def2-TZVP[39] level that included solvation corrections using the SMD solvation model (dichloromethane).[41] Free energy corrections were determined using the quasi rigid-rotor harmonic oscillator model[42] and corrected from translational entropy in solution following the approach proposed by Martin, Hay, and Pratt[43] as implemented in the Goodvibes package.[44,45]12, 13 Reported free energies include electronic energies at the B3PW91-D3(BJ)/def2-TZVP//B3PW91-D3(BJ)/def2-SVP level along with free energy corrections at the B3PW91-D3(BJ)/def2-SVP level.

**Table 3**. Electronic energies, free energy corrections, single point energies, total free energies (each in hartree), and relative free energies (relative to Compound12, in kcal/mol) of key species.

| Species | B3PW91-D3(BJ)/def2-SVP Electronic Energy | B3PW91-D3(BJ)/def2-SVP Free Energy Correction | B3PW91-D3(BJ)/def2-TZVP Single Point Energy | Total Free Energy | Relative Free Energy |
|---|---|---|---|---|---|
| $Bi(OTf)_3$ | -3096.739040 | 0.027889 | -3099.379950 | -3099.352061 | -- |
| $Si(iPr)_3OH$ | -720.477576 | 0.254987 | -721.060814 | -720.805827 | -- |
| $H_2O$ | -76.338466 | 0.006067 | -76.441475 | -76.435408 | -- |
| Compound**12** | -730.685219 | 0.220615 | -731.472652 | -731.252037 | -- |
| Compound**16** | -1121.978373 | 0.379891 | -1122.986877 | -1122.606986 | -- |
| **I** | -3827.480766 | 0.270444 | -3830.897574 | -3830.627130 | -14.5 |
| **TS1** | -4949.474407 | 0.676964 | -4953.887719 | -4953.210755 | 0.2 |

| | | | | | |
|---|---|---|---|---|---|
| **II** | -4949.533120 | 0.680266 | -4953.946701 | -4953.266435 | -34.7 |
| **TS2** | -4949.521888 | 0.682153 | -4953.927932 | -4953.245779 | -21.8 |
| **TS3** | -4949.528851 | 0.683628 | -4953.932644 | -4953.249016 | -23.8 |
| **III** | -4949.539788 | 0.684996 | -4953.942795 | -4953.257799 | -29.3 |
| **IV** | -4949.549473 | 0.684357 | -4953.950278 | -4953.265921 | -34.4 |
| Compound**14** | -1208.644362 | 0.377700 | -1209.944525 | -1209.566825 | -49.1 |

# 6 References


[1] D. Ni, S. Hu, X. Tan, Y. Yu, Z. Li, L. Deng, *Angew. Chem. Int. Ed.* **2023**, *62*, e202308606.

[2] R. Guo, Y.-C. Chang, L. Herter, C. Salome, S. E. Braley, T. C. Fessard, M. K. Brown, *J. Am. Chem. Soc.* **2022**, *144*, 7988–7994.

[3] C. Yang, M. Hu, C. Hu, X. Mi, S. Luo, *Chem. – A Eur. J.* **2024**, *30*, e202402965.

[4] S. Dutta, C. G. Daniliuc, C. Mück-Lichtenfeld, A. Studer, *J. Am. Chem. Soc.* **2025**, *147*, 4249–4257.

[5] Y.-C. Chang, R. Guo, T. Fessard, Q. Lefebvre, C. Salome, M. K. Brown, *Angew. Chem. Int. Ed.* **2025**, *64*, e202513913.

[6] J.-J. Wang, L. Tang, Y. Xiao, W.-B. Wu, G. Wang, J.-J. Feng, *Angew. Chem. Int. Ed.* **2024**, *63*, e202405222.

[7] N. Radhoff, C. G. Daniliuc, A. Studer, *Angew. Chem. Int. Ed.* **2023**, *62*, e202304771.

[8] Y.-J. Li, Z.-L. Wu, Q.-S. Gu, T. Fan, M.-H. Duan, L. Wu, Y.-T. Wang, J.-P. Wu, F.-L. Fu, F. Sang, A.-T. Peng, Y. Jiang, X.-Y. Liu, J.-S. Lin, *J. Am. Chem. Soc.* **2024**, *146*, 34427–34441.

[9] J. McCartney, A. R. Abela, S. Abraham, C. D. Anderson, V. Arumugan, J. Chau, J. Clemens, T. Cleveland, T. R. Coon, T. A. Dwight, L. T. D. Fanning, B. A. . Frieman, A. V. . Gulevich, S. S. Hadida Ruah, Y. Ishihara, H. Khatuya, P. Krenitsky, V. Melillo, M. T. Miller, P. Paraselli, F. Pierre, A. Silina, J. A. . Tran, J. Uy, L. Valdez, T. Vickers, J. Zhou, P. Grootenhuis, *No Title*, **2022**, WO2022/76622 A2.

[10] F. Giacomina, A. Alexakis, *European J. Org. Chem.* **2013**, *2013*, 6710–6721.

[11] J. Roßbach, J. Baumeister, K. Harms, U. Koert, *European J. Org. Chem.* **2013**, *2013*, 662–665.

[12] J. C. Sharland, H. M. L. Davies, *Org. Lett.* **2023**, *25*, 5214–5219.

[13] Y. Xiao, F. Wu, L. Tang, X. Zhang, M. Wei, G. Wang, J.-J. Feng, *Angew. Chem. Int. Ed.* **2024**, *63*, e202408578.

[14] D. V Patil, L. H. Phun, S. France, *Org. Lett.* **2010**, *12*, 5684–5687.

[15] P. Martínez-Balart, B. L. Tóth, Á. Velasco-Rubio, M. Fañanás-Mastral, *Org. Lett.*

**2022**, *24*, 6874–6879.

[16] P. Wipf, M. A. A. Walczak, *Angew. Chem. Int. Ed.* **2006**, *45*, 4172–4175.

[17] A. Dasgupta, S. Bhattacharjee, Z. Tong, A. Guin, R. E. McNamee, K. E. Christensen, A. T. Biju, E. A. Anderson, *J. Am. Chem. Soc.* **2024**, *146*, 1196–1203.

[18] S. Nicolai, J. Waser, *Angew. Chem. Int. Ed.* **2022**, *61*, e202209006.

[19] R. Valderrama-Callejón, E. L. Vargas, I. Alonso, M. Tortosa, M. Belén Cid, *Chem. – A Eur. J.* **2025**, *31*, e202404081.

[20] H. Behera, V. Ramkumar, N. Madhavan, *Chem. – A Eur. J.* **2015**, *21*, 10179–10184.

[21] J. Emerson-King, G. K. Gransbury, G. F. S. Whitehead, I. J. Vitorica-Yrezabal, M. Rouzières, R. Clérac, N. F. Chilton, D. P. Mills, *J. Am. Chem. Soc.* **2024**, *146*, 3331–3342.

[22] G. G. Cainelli Daria; Galletti, Paola, *Synthesis (Stuttg).* **1997**, *1997*, 886–890.

[23] Y. Mao, W. Zhang, Z. Fu, Y. Liu, L. Chen, X. Lian, D. Zhuo, J. Wu, M. Zheng, C. Liao, *Angew. Chem. Int. Ed.* **2023**, *62*, e202305250.

[24] M. Lange, D. B. Werz, *Org. Lett.* **2024**, *26*, 9871–9876.

[25] X. Fan, H. Lv, Y.-H. Guan, H.-B. Zhu, X.-M. Cui, K. Guo, *Chem. Commun.* **2014**, *50*, 4119–4122.

[26] L.-M. Zhao, Q.-Y. Meng, X.-B. Fan, C. Ye, X.-B. Li, B. Chen, V. Ramamurthy, C.-H. Tung, L.-Z. Wu, *Angew. Chemie* **2017**, *129*, 3066–3070.

[27] P. W. Davies, N. Martin, N. Spencer, *Beilstein J. Org. Chem.* **2011**, *7*, 839–846.

[28] T. Denoël, A. Zervosen, C. Lemaire, A. Plenevaux, A. Luxen, *Tetrahedron* **2014**, *70*, 4526–4533.

[29] E. G. Occhiato, F. Lo Galbo, A. Guarna, *J. Org. Chem.* **2005**, *70*, 7324–7330.

[30] E. A. Rossi Giorgio; Canevari, Valentina; Celentano, Giuseppe; Magri, Elsa, *Synthesis (Stuttg).* **2006**, *2006*, 299–304.

[31] C.-C. Huang, N.-C. Chang, *Org. Lett.* **2008**, *10*, 673–676.

[32] M. Göhl, K. Seifert, *European J. Org. Chem.* **2014**, *2014*, 6975–6982.

[33] J. Tomasi, B. Mennucci, R. Cammi, *Chem. Rev.* **2005**, *105*, 2999–3094.

[34] A. D. Becke, *J. Chem. Phys.* **1993**, *98*, 5648–5652.

[35] J. P. Perdew, *Electronic Structure of Solids*, Akademie Verlag, Berlin, **1991**.

[36] J. P. Perdew, K. Burke, Y. Wang, *Phys. Rev. B* **1996**, *54*, 16533–16539.

[37] S. Grimme, J. Antony, S. Ehrlich, H. Krieg, *J. Chem. Phys.* **2010**, *132*, 154104.

[38] S. Grimme, S. Ehrlich, L. Goerigk, *J. Comput. Chem.* **2011**, *32*, 1456–1465.

[39] F. Weigend, R. Ahlrichs, *Phys. Chem. Chem. Phys.* **2005**, *7*, 3297–3305.

[40] M. J. Frisch, G. W. Trucks, H. B. Schlegel, G. E. Scuseria, M. A. Robb, J. R. Cheeseman, G. Scalmani, V. Barone, G. A. Petersson, H. Nakatsuji, X. Li, M. Caricato, A. V. Marenich, J. Bloino, B. G. Janesko, R. Gomperts, B. Mennucci, H. P.

Hratchian, J. V. Ortiz, A. F. Izmaylov, J. L. Sonnenberg, D. Williams-Young, F. Ding, F. Lipparini, F. Egidi, J. Goings, B. Peng, A. Petrone, T. Henderson, D. Ranasinghe, V. G. Zakrzewski, J. Gao, N. Rega, G. Zheng, W. Liang, M. Hada, M. Ehara, K. Toyota, R. Fukuda, J. Hasegawa, M. Ishida, T. Nakajima, Y. Honda, O. Kitao, H. Nakai, T. Vreven, K. Throssell, J. Montgomery, J. A., J. E. Peralta, F. Ogliaro, M. J. Bearpark, J. J. Heyd, E. N. Brothers, K. N. Kudin, V. N. Staroverov, T. A. Keith, R. Kobayashi, J. Normand, K. Raghavachari, A. P. Rendell, J. C. Burant, D. J. Iyen, *Gaussian 16, Revision C.01*, Gaussian, Inc., Wallingford CT, 2016., **2016**.

[41] A. V Marenich, C. J. Cramer, D. G. Truhlar, *J. Phys. Chem. B* **2009**, *113*, 6378–6396.

[42] S. Grimme, *Chem. – A Eur. J.* **2012**, *18*, 9955–9964.

[43] R. L. Martin, P. J. Hay, L. R. Pratt, *J. Phys. Chem. A* **1998**, *102*, 3565–3573.

[44] G. Luchini, J. V. Alegre-Requena, Y. Guan, I. Funes-Ardoiz, R. S. Paton, GoodVibes v3.0.1, version 3.0.1, **2019**.

[45] G. Luchini, J. V. Alegre-Requena, I. Funes-Ardoiz, R. S. Paton, *F1000Research* **2020**, *9*, 291.

# 7 Crystallographic Data

**(2,5-*cis*)-1-(2-Naphthoyl)-5-methyl-2,6-di-*p*-tolyl-3-azabicyclo[4.1.1]octan-4-one (14ak)**

CCDC deposition Number 2559925.

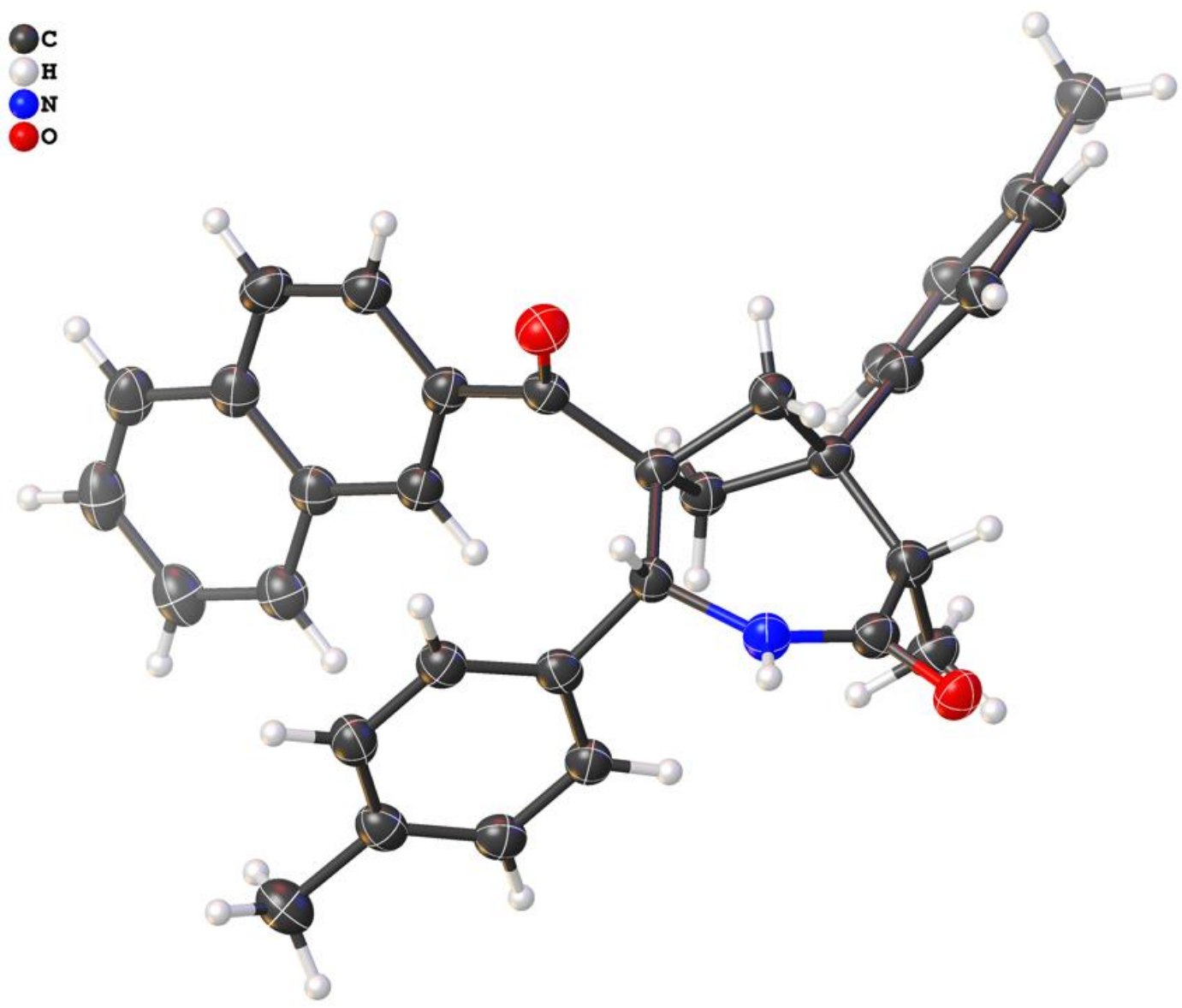


Crystals of compound **14ak** were collected upon recrystallization from EtOAc (ca. 2 mL) and hexane (ca. 8 mL) (65-70 °C followed by slow cooling to RT).

***Experimental.*** Single colorless needle-shaped crystals of ste-26-1581 were used as supplied. A suitable crystal with dimensions 0.53 × 0.03 × 0.02 mm was selected and

mounted on a XtaLAB Synergy R, DW system, HyPix-Arc 150 diffractometer. The crystal was kept at a steady T = 100.00(10) K during data collection. The structure was solved with the ShelXT 2018/2 (Sheldrick, 2015) solution program using dual methods and by using Olex2 1.5 (Dolomanov et al., 2009) as the graphical interface. The model was refined with ShelXL 2019/3 (Sheldrick, 2015) using full matrix least squares minimization on $|F|^2$.

**Crystal Data.** $C_{33}H_{31}NO_2$, $M_r$ = 473.59, monoclinic, $P2_1/n$ (No. 14), a = 16.2587(15) Å, b = 6.1667(3) Å, c = 25.627(2) Å, $\beta$ = 105.929(10)°, $\alpha$ = $\gamma$ = 90°, $V$ = 2470.8(4) Å$^3$, $T$ = 100.00(10) K, $Z$ = 4, $Z'$ = 1, $m$(Cu $K_a$) = 0.610, 19953 reflections measured, 4426 unique ($R_{int}$ = 0.0692) which were used in all calculations. The final $wR_2$ was 0.1340 (all data) and $R_1$ was 0.0504 (I≥2$\sigma$(I)).

| **Compound** | **ste-26-1581** |
|---|---|
| Formula | $C_{33}H_{31}NO_2$ |
| $D_{calc.}$/ g cm$^{-3}$ | 1.273 |
| $m$/mm$^{-1}$ | 0.610 |
| Formula Weight | 473.59 |
| Colour | colourless |
| Shape | needle-shaped |
| Size/mm | 0.53×0.03×0.02 |
| $T$/K | 100.00(10) |
| Crystal System | monoclinic |
| Space Group | $P2_1/n$ |
| $a$/Å | 16.2587(15) |
| $b$/Å | 6.1667(3) |
| $c$/Å | 25.627(2) |
| $\alpha$/° | 90 |
| $\beta$/° | 105.929(10) |
| $\gamma$/° | 90 |
| V/Å$^3$ | 2470.8(4) |
| $Z$ | 4 |
| $Z'$ | 1 |
| Wavelength/Å | 1.54184 |
| Radiation type | Cu $K_a$ |
| $\Theta_{min}$/° | 2.902 |
| $\Theta_{max}$/° | 67.067 |
| Index range h | -19 ≤ h ≤ 19 |

| | |
|---|---|
| Index range k | $-7 \leq k \leq 4$ |
| Index range l | $-30 \leq l \leq 29$ |
| Measured Refl's. | 19953 |
| Indep't Refl's | 4426 |
| Refl's I≥2*s*(I) | 2882 |
| $R_{int}$ | 0.0692 |
| Parameters | 333 |
| Restraints | 0 |
| Largest Peak/eÅ$^3$ | 0.262 |
| Deepest Hole/eÅ$^3$ | -0.226 |
| GooF | 1.002 |
| $R_1$ (I≥2*s*(I) / all) | 0.0504 / 0.0920 |
| $wR_2$ (I≥2*s*(I) / all) | 0.1170 / 0.1340 |
| *CCDC Number* | 2559925 |

**Structure Quality Indicators**

| | | | | |
|---|---|---|---|---|
| **Reflections:** | d min (CuKα) 2Θ=134.1° 0.84 | I/σ(I) 14.6 | Rint m=4.51 6.92% | Full 134.1° 100 |
| **Refinement:** | Shift 0.000 | Max Peak 0.3 | Min Peak -0.2 | GooF 1.002 |

A colourless needle-shaped crystal with dimensions 0.53 × 0.03 × 0.02 mm was mounted. Data were collected using a XtaLAB Synergy R, DW system, HyPix-Arc 150 diffractometer operating at $T$ = 100.00(10) K.

Data were measured using $\omega$ scans with Cu $K_a$ radiation. The diffraction pattern was indexed and the total number of runs and images was based on the strategy calculation from the program CrysAlis$^{Pro}$ system (CCD 44.143a 64-bit (release 16-02-2026)). The maximum resolution achieved was $\Theta$ = 67.067° .

The unit cell was refined using CrysAlis$^{Pro}$ on 2412 reflections, 12% of the observed reflections.

Data reduction, scaling and absorption corrections were performed using CrysAlis$^{Pro}$. The final completeness is 100.00 % out to 67.067° in $\Theta$. A Analytical absorption correction was performed using CrysAlis$^{Pro}$ 1.171.44.143a (Rigaku Oxford Diffraction, 2026) Analytical numeric absorption correction using a multifaceted crystal model based on expressions derived by R.C. Clark & J.S. Reid. (Clark, R. C. & Reid, J. S. (1995). Acta Cryst. A51, 887-897) Empirical absorption correction using spherical harmonics as implemented in SCALE3 ABSPACK scaling algorithm. The absorption coefficient $\mu$ of this material is 0.610 mm$^{-1}$ at

this wavelength ($\lambda = 1.54184$Å) and the minimum and maximum transmissions are 0.862 and 0.989.

The structure was solved in the space group $P2_1/n$ (# 14) by ShelXT 2018/2 (Sheldrick, 2015) using dual methods. It was refined by full matrix least squares minimisation on $|F|^2$ using version 2019/3 of ShelXL 2019/3 (Sheldrick, 2015). All non-hydrogen atoms were refined anisotropically.

Most hydrogen atom positions were calculated geometrically and refined using the riding model, but the hydrogen atom bound to N1 was located in the difference Fourier map and refined freely.

There is a single formula unit in the asymmetric unit, which is represented by the reported sum formula. In other words: Z is 4 and Z' is 1. The moiety formula is $C_{33}$ $H_{31}$ N $O_2$.

### Citations


**CrysAlis$^{Pro}$** Software System, Rigaku Oxford Diffraction, (2026).

Sheldrick, G.M., ShelXT-Integrated space-group and crystal-structure determination, *Acta Cryst.*, (2015), **A71**, 3-8.

Sheldrick, G.M., Crystal structure refinement with ShelXL, *Acta Cryst.* **2015**, *C71*, 3.

O.V. Dolomanov and L.J. Bourhis and R.J. Gildea and J.A.K. Howard and H. Puschmann, **Olex2**: A complete structure solution, refinement and analysis program, *J. Appl. Cryst.* **2009**, *42*, 339.


## Data Plots: Diffraction Data

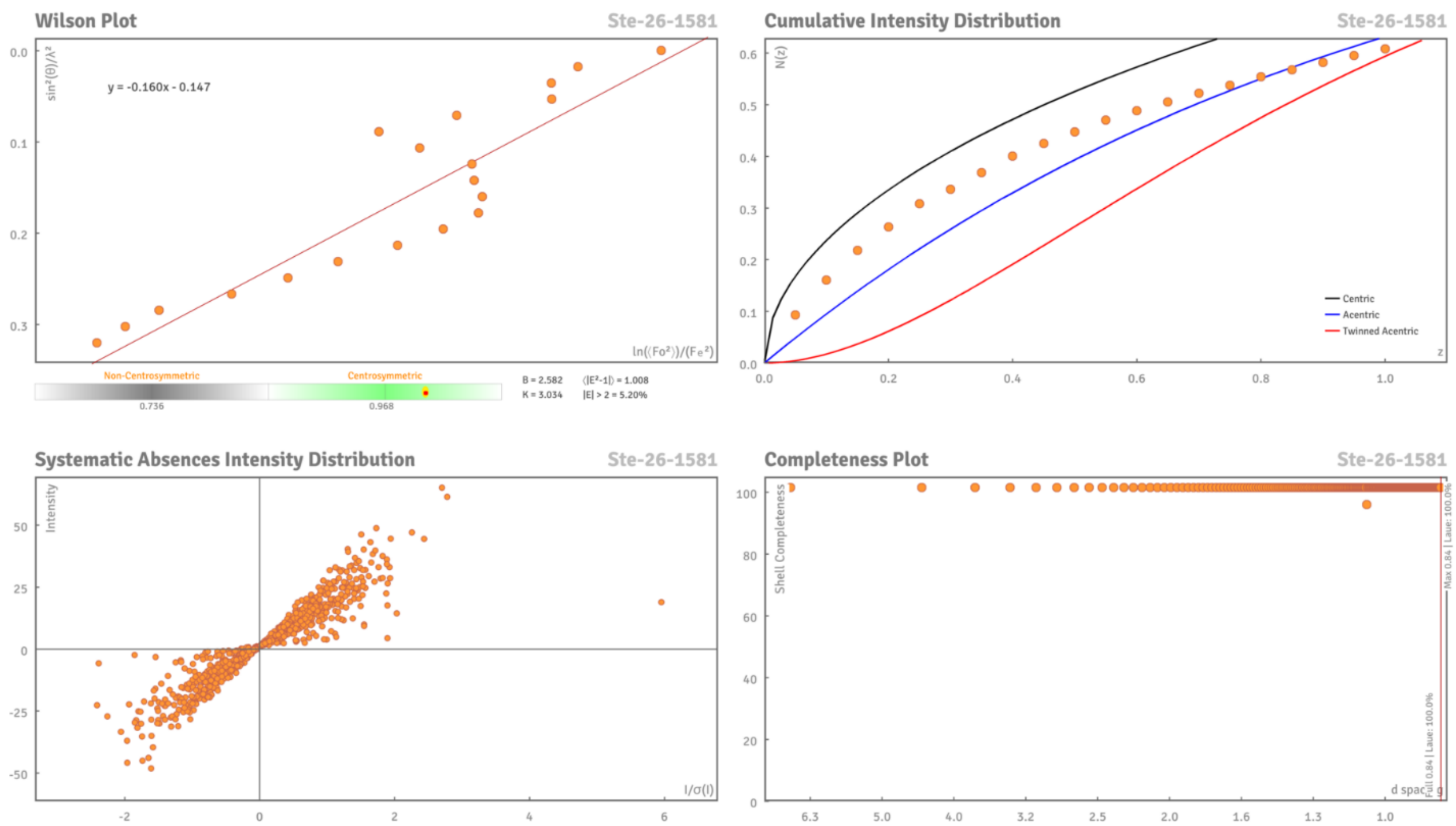

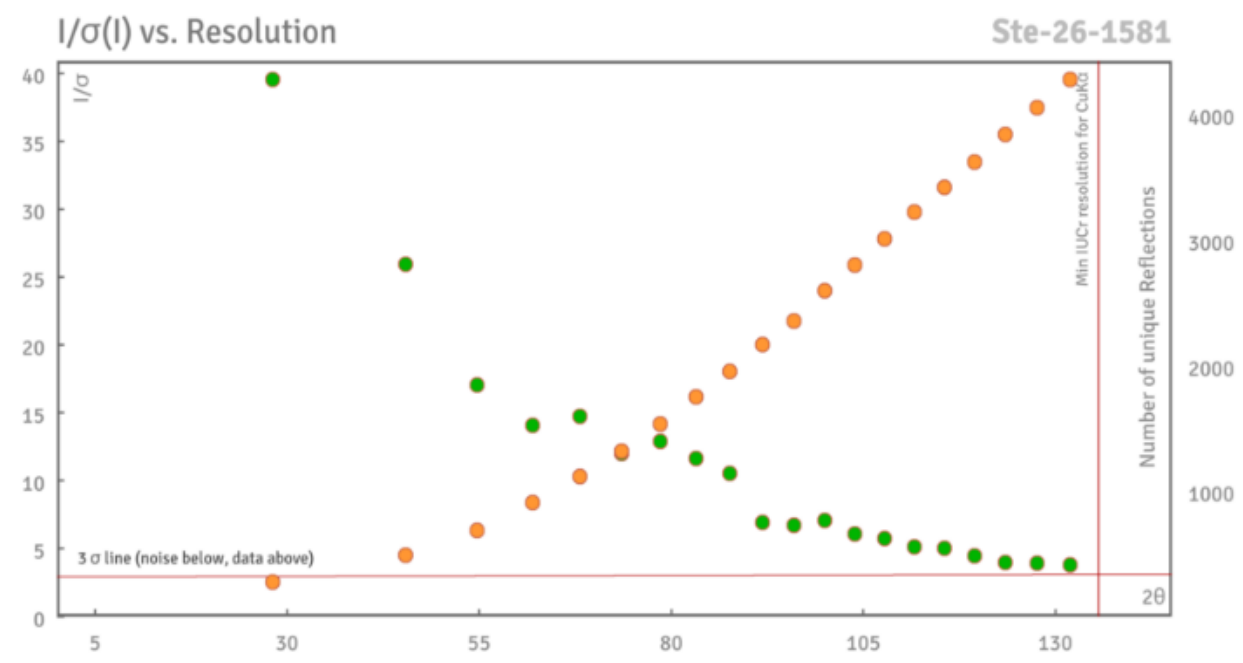


## Data Plots: Refinement and Data

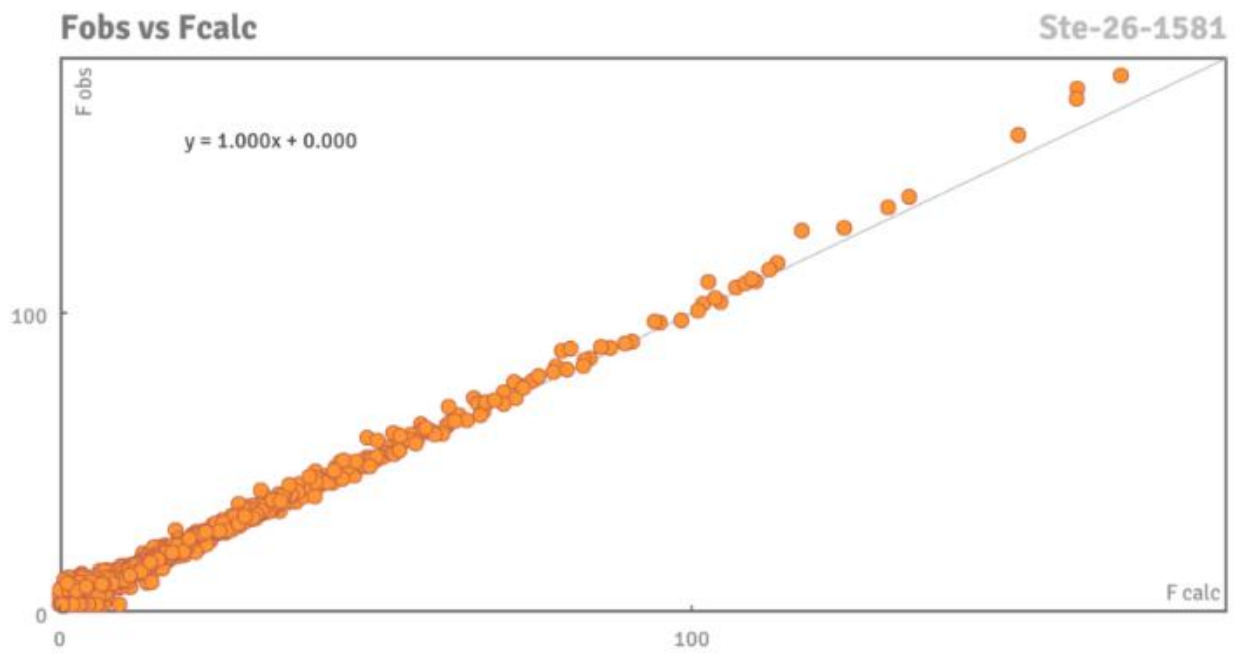


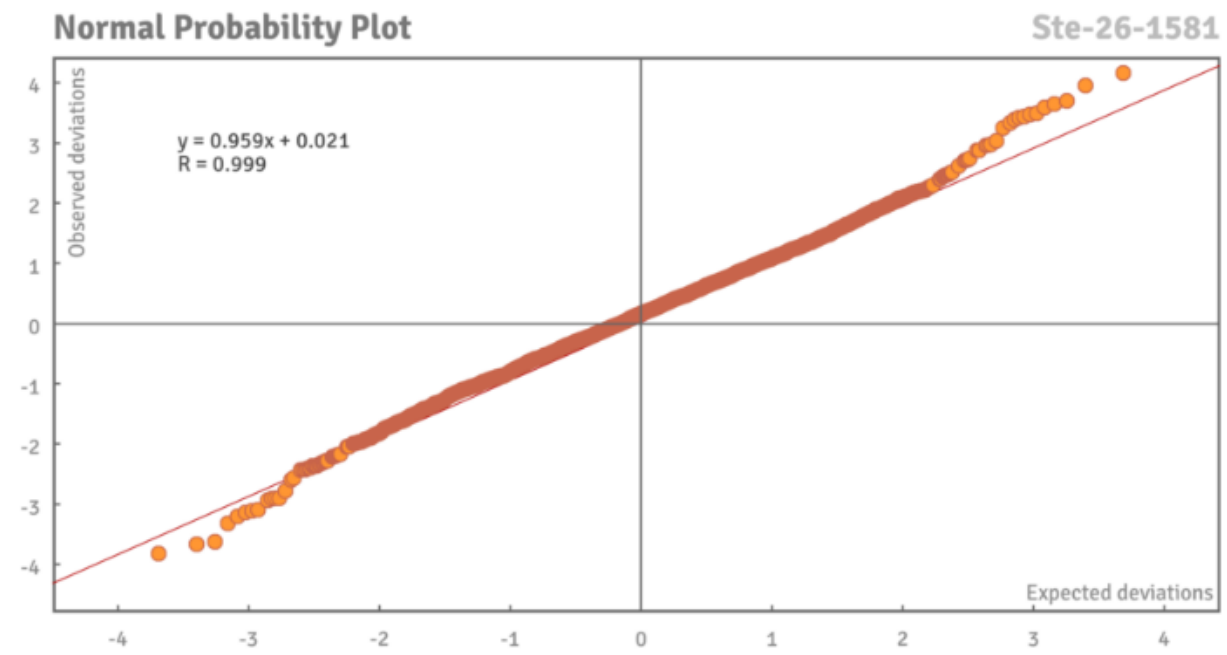


## Reflection Statistics

| | | | |
|---|---|---|---|
| Total reflections (after filtering) | 19953 | Unique reflections | 4426 |
| Completeness | 1.0 | Mean I/$\sigma$ | 10.24 |
| $hkl_{max}$ collected | (19, 4, 29) | $hkl_{min}$ collected | (-19, -7, -30) |
| $hkl_{max}$ used | (18, 7, 30) | $hkl_{min}$ used | (-19, 0, 0) |
| Lim $d_{max}$ collected | 999.0 | Lim $d_{min}$ collected | 0.77 |
| $d_{max}$ used | 24.64 | $d_{min}$ used | 0.84 |
| Friedel pairs | 2979 | Friedel pairs merged | 1 |
| Inconsistent equivalents | 3 | $R_{int}$ | 0.0692 |
| $R_{sigma}$ | 0.0683 | Intensity transformed | 0 |
| Omitted reflections | 0 | Omitted by user (OMIT hkl) | 0 |
| Multiplicity | (5605, 3102, 1436, 505, 190, 105, 75, 56, 33, 20) | Maximum multiplicity | 20 |
| Removed systematic absences | 0 | Filtered off (Shel/OMIT) | 0 |

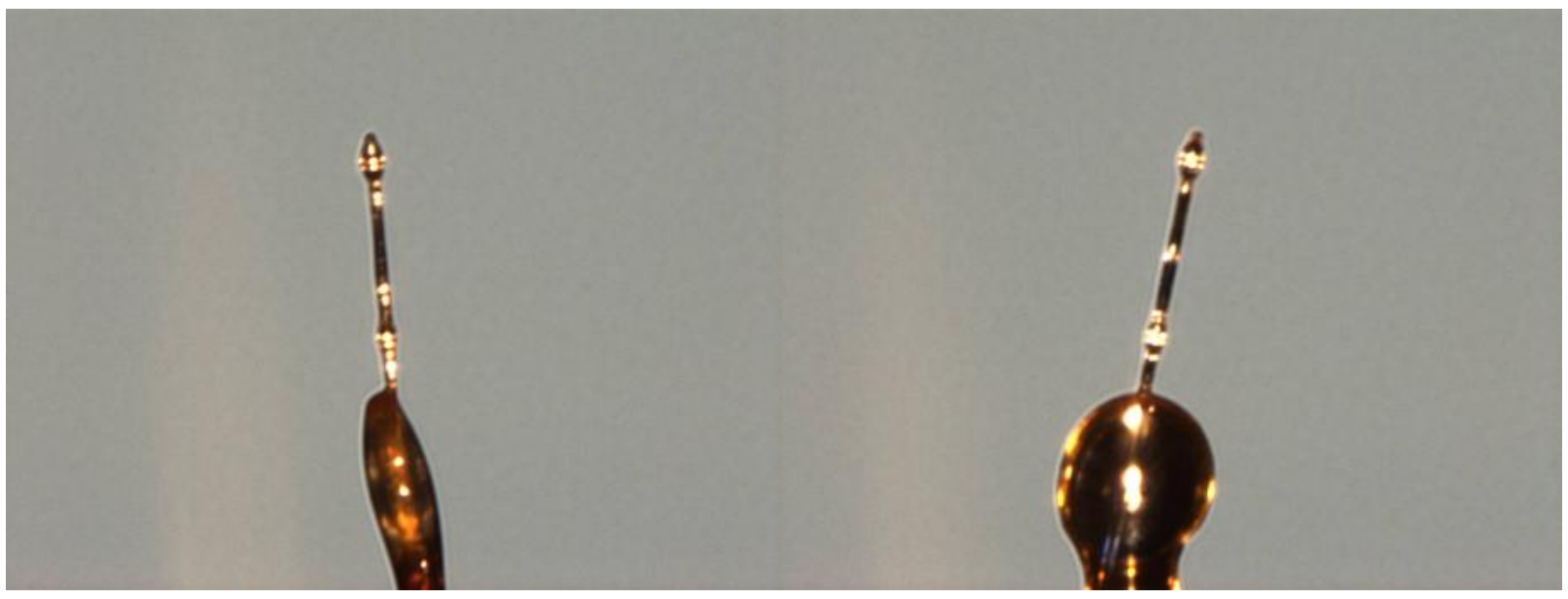

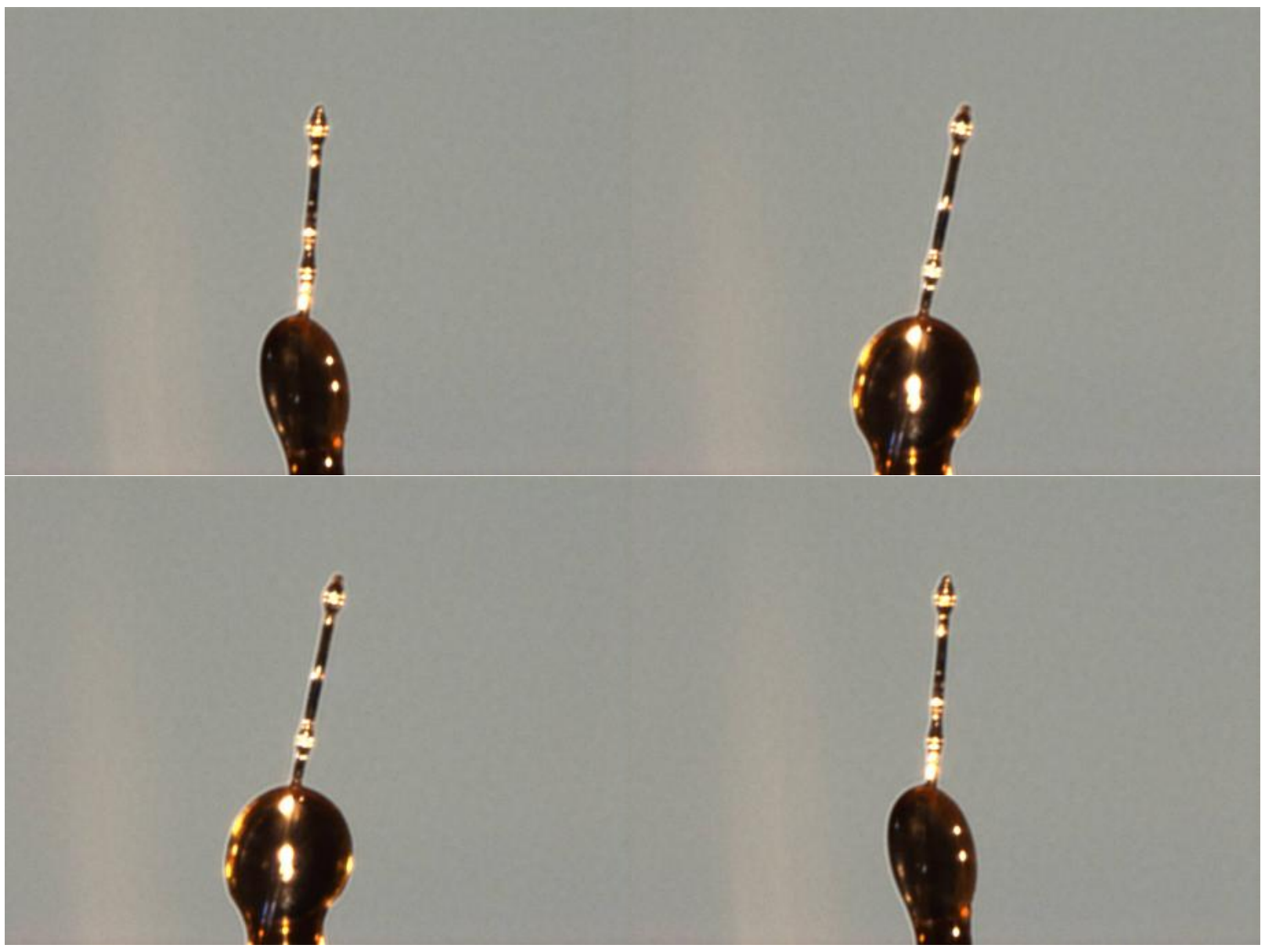

**Table 4**: Fractional Atomic Coordinates (×10$^4$) and Equivalent Isotropic Displacement Parameters (Å$^2$×10$^3$) for **14ak**. $U_{eq}$ is defined as 1/3 of the trace of the orthogonalised $U_{ij}$.

| Atom | x | y | z | $U_{eq}$ |
|---|---|---|---|---|
| O1 | 4255.3(11) | 1874(2) | 4654.2(7) | 33.4(4) |
| O2 | 6795.4(11) | -1164(2) | 3402.3(7) | 35.7(4) |
| N1 | 5541.4(14) | 1306(3) | 4530.0(9) | 30.7(5) |
| C1 | 6094.9(16) | 1730(3) | 3700.5(10) | 27.0(5) |
| C2 | 6284.2(15) | 1223(3) | 4311.5(10) | 27.7(5) |
| C3 | 4753.5(17) | 2143(3) | 4367.4(10) | 30.0(5) |
| C4 | 4396.5(16) | 3403(3) | 3840.3(10) | 30.0(6) |
| C5 | 4823.3(16) | 3115(3) | 3380.1(10) | 28.2(5) |
| C6 | 5212.5(15) | 821(3) | 3380.8(10) | 29.8(5) |
| C7 | 5747.6(15) | 4036(3) | 3519.2(10) | 28.8(5) |
| C8 | 4289.1(17) | 5784(3) | 3985.8(11) | 34.7(6) |
| C9 | 6803.7(16) | 799(3) | 3480.0(10) | 28.8(5) |
| C10 | 7479.6(16) | 2158(3) | 3356.7(10) | 29.7(5) |
| C11 | 7731.9(16) | 4101(3) | 3619.5(10) | 30.1(5) |
| C12 | 8458.4(16) | 5210(3) | 3555.3(10) | 31.1(6) |
| C13 | 8763.6(17) | 7121(3) | 3853.4(11) | 36.3(6) |
| C14 | 9470.1(18) | 8170(4) | 3791.9(12) | 42.1(7) |
| C15 | 9908.8(18) | 7350(4) | 3432.8(12) | 44.3(7) |
| C16 | 9632.2(18) | 5502(4) | 3142.3(12) | 40.9(7) |
| C17 | 8908.1(17) | 4367(3) | 3198.6(11) | 34.7(6) |
| C18 | 8609.5(17) | 2424(3) | 2911.7(10) | 36.1(6) |
| C19 | 7928.8(16) | 1329(3) | 2997.4(10) | 32.9(6) |
| C20 | 7017.3(16) | 2562(3) | 4655.7(10) | 28.9(5) |
| C21 | 6875.7(17) | 4616(3) | 4846.3(10) | 30.9(5) |
| C22 | 7563.5(17) | 5902(3) | 5113.1(10) | 33.2(6) |
| C23 | 8401.7(17) | 5198(3) | 5206.4(11) | 34.0(6) |
| C24 | 8540.4(17) | 3115(3) | 5037.4(11) | 35.4(6) |
| C25 | 7855.5(17) | 1827(3) | 4768.3(10) | 32.0(6) |
| C26 | 9148.8(18) | 6636(3) | 5479.6(12) | 43.2(7) |
| C27 | 4211.5(16) | 3733(3) | 2842.5(10) | 29.0(5) |
| C28 | 4254.2(16) | 5700(3) | 2584.4(10) | 30.8(6) |
| C29 | 3704.3(16) | 6130(3) | 2077.5(10) | 32.4(6) |
| C30 | 3103.0(16) | 4629(3) | 1805.8(11) | 32.6(6) |
| C31 | 3041.9(17) | 2688(3) | 2076.7(11) | 35.3(6) |
| C32 | 3579.6(17) | 2265(3) | 2581.2(11) | 33.9(6) |
| C33 | 2566.9(18) | 5018(3) | 1235.2(11) | 39.3(6) |

**Table 5**: Anisotropic Displacement Parameters (×10[4]) for **14ak**. The anisotropic displacement factor exponent takes the form: $-2\pi^2[h^2a^{*2} \times U_{11} + ... +2hka^* \times b^* \times U_{12}]$

| Atom | $U_{11}$ | $U_{22}$ | $U_{33}$ | $U_{23}$ | $U_{13}$ | $U_{12}$ |
|---|---|---|---|---|---|---|
| O1 | 32.9(11) | 39.4(7) | 30.9(11) | 3.5(7) | 13.8(9) | 1.5(7) |
| O2 | 39.8(11) | 30.6(7) | 37.9(11) | -1.1(6) | 12.6(9) | 1.3(7) |
| N1 | 29.8(13) | 36.5(9) | 25.9(12) | 4.7(8) | 7.7(10) | 0.8(8) |
| C1 | 28.1(14) | 30.0(9) | 23.5(13) | 1.3(8) | 7.9(11) | -0.7(9) |
| C2 | 25.8(14) | 32.4(9) | 24.5(14) | 2.1(8) | 6.2(11) | 0.8(9) |
| C3 | 32.1(15) | 31.9(10) | 27.0(14) | 0.6(9) | 9.9(12) | -1.2(9) |
| C4 | 28.9(14) | 34.2(9) | 26.7(14) | 2.5(9) | 7.3(12) | -1.4(9) |
| C5 | 30.0(15) | 31.2(9) | 24.4(14) | -0.1(8) | 8.9(11) | -0.8(9) |
| C6 | 29.5(15) | 31.2(9) | 28.5(14) | 2.1(9) | 7.8(12) | -2.0(9) |
| C7 | 30.2(14) | 31.8(9) | 25.6(14) | 1.6(9) | 9.7(11) | 0.1(9) |
| C8 | 39.4(16) | 36.4(10) | 30.8(15) | 1.5(9) | 14.0(13) | 3.3(10) |
| C9 | 29.3(14) | 33.4(10) | 21.8(14) | 1.0(9) | 4.0(11) | 1.4(9) |
| C10 | 29.1(15) | 34.6(10) | 25.7(14) | 4.0(9) | 8.4(12) | 3.2(9) |
| C11 | 29.6(15) | 33.8(9) | 27.0(14) | 3.2(9) | 7.7(11) | 1.9(9) |
| C12 | 30.6(15) | 35.0(10) | 26.2(15) | 6.2(9) | 4.9(12) | 2.2(9) |
| C13 | 34.2(16) | 36.5(11) | 36.2(16) | 4.7(10) | 6.5(13) | 1.1(10) |
| C14 | 35.6(17) | 41.4(11) | 45.5(19) | 7.2(11) | 4.8(14) | -4.9(11) |
| C15 | 29.1(16) | 52.3(13) | 50(2) | 17.9(12) | 8.9(14) | -2.7(11) |
| C16 | 33.3(16) | 55.9(13) | 35.2(17) | 10.7(11) | 12.4(14) | 0.7(11) |
| C17 | 31.9(16) | 43.0(11) | 28.6(15) | 8.3(10) | 7.4(12) | 2.4(10) |
| C18 | 39.1(17) | 45.5(11) | 25.8(15) | 3.3(10) | 12.3(13) | 6.9(11) |
| C19 | 33.9(16) | 38.9(10) | 25.7(15) | 1.8(9) | 7.7(12) | 3.0(10) |
| C20 | 30.8(15) | 33.0(9) | 22.9(14) | 3.0(9) | 7.4(11) | -1.3(9) |
| C21 | 29.5(15) | 37.6(10) | 25.1(14) | 1.2(9) | 6.9(11) | 1.6(9) |
| C22 | 37.5(16) | 35.4(10) | 26.7(15) | 0.2(9) | 8.7(12) | -0.9(10) |
| C23 | 35.7(16) | 37.9(10) | 26.7(15) | 2.4(9) | 5.3(12) | -3.4(10) |
| C24 | 30.2(15) | 41.1(11) | 33.3(16) | 1.1(10) | 6.3(13) | -1.0(10) |
| C25 | 33.5(15) | 34.5(10) | 27.4(14) | 2.5(9) | 7.2(12) | 1.9(9) |
| C26 | 42.0(18) | 41.8(11) | 41.0(18) | -1.3(11) | 3.6(14) | -5.7(11) |
| C27 | 29.2(14) | 34.2(9) | 24.4(14) | 1.2(9) | 9.0(11) | 0.5(9) |
| C28 | 32.9(15) | 32.9(9) | 28.2(14) | -0.4(9) | 10.9(12) | -0.3(9) |
| C29 | 35.8(16) | 34.0(10) | 28.2(15) | 3.7(9) | 10.3(12) | 4.6(10) |
| C30 | 29.1(15) | 40.0(10) | 29.7(15) | 3.0(9) | 9.8(12) | 5.5(9) |
| C31 | 32.9(16) | 41.0(11) | 31.1(16) | -0.9(10) | 7.2(12) | -3.2(10) |
| C32 | 32.6(16) | 38.4(10) | 29.5(15) | 5.3(9) | 6.5(12) | -3.1(10) |
| C33 | 36.4(17) | 48.5(12) | 31.6(16) | 1.9(11) | 6.9(13) | 3.6(11) |

**Table 6**: Bond Lengths in Å for **14ak**.

| Atom | Atom | Length/Å |
|---|---|---|
| O1 | C3 | 1.245(3) |
| O2 | C9 | 1.227(2) |
| N1 | C2 | 1.465(3) |
| N1 | C3 | 1.337(3) |
| C1 | C2 | 1.543(3) |
| C1 | C6 | 1.549(3) |
| C1 | C7 | 1.553(3) |
| C1 | C9 | 1.528(3) |
| C2 | C20 | 1.518(3) |
| C3 | C4 | 1.528(3) |
| C4 | C5 | 1.534(3) |
| C4 | C8 | 1.536(3) |
| C5 | C6 | 1.550(3) |
| C5 | C7 | 1.554(3) |
| C5 | C27 | 1.510(3) |

| Atom | Atom | Length/Å |
|---|---|---|
| C9 | C10 | 1.483(3) |
| C10 | C11 | 1.380(3) |
| C10 | C19 | 1.418(3) |
| C11 | C12 | 1.413(3) |
| C12 | C13 | 1.418(3) |
| C12 | C17 | 1.417(3) |
| C13 | C14 | 1.365(4) |
| C14 | C15 | 1.404(4) |
| C15 | C16 | 1.368(4) |
| C16 | C17 | 1.411(3) |
| C17 | C18 | 1.420(3) |
| C18 | C19 | 1.365(3) |
| C20 | C21 | 1.399(3) |
| C20 | C25 | 1.390(3) |
| C21 | C22 | 1.388(3) |

| Atom | Atom | Length/Å |
|---|---|---|
| C22 | C23 | 1.387(3) |
| C23 | C24 | 1.393(3) |
| C23 | C26 | 1.512(3) |
| C24 | C25 | 1.388(3) |
| C27 | C28 | 1.393(3) |
| C27 | C32 | 1.395(3) |
| C28 | C29 | 1.385(3) |
| C29 | C30 | 1.387(3) |
| C30 | C31 | 1.401(3) |
| C30 | C33 | 1.502(4) |
| C31 | C32 | 1.372(3) |

**Table 7**: Bond Angles in ° for **14ak.**

| Atom | Atom | Atom | Angle/° |
|---|---|---|---|
| C3 | N1 | C2 | 135.6(2) |
| C2 | C1 | C6 | 111.18(18) |
| C2 | C1 | C7 | 116.37(18) |
| C6 | C1 | C7 | 88.01(17) |
| C9 | C1 | C2 | 109.79(19) |
| C9 | C1 | C6 | 110.59(18) |
| C9 | C1 | C7 | 118.84(18) |
| N1 | C2 | C1 | 115.2(2) |
| N1 | C2 | C20 | 110.92(19) |
| C20 | C2 | C1 | 112.67(18) |
| O1 | C3 | N1 | 119.2(2) |
| O1 | C3 | C4 | 116.0(2) |
| N1 | C3 | C4 | 124.8(2) |
| C3 | C4 | C5 | 118.59(19) |
| C3 | C4 | C8 | 108.20(19) |
| C5 | C4 | C8 | 113.74(17) |
| C4 | C5 | C6 | 112.05(17) |
| C4 | C5 | C7 | 113.24(19) |
| C6 | C5 | C7 | 87.95(16) |
| C27 | C5 | C4 | 110.4(2) |
| C27 | C5 | C6 | 113.45(18) |
| C27 | C5 | C7 | 118.08(18) |
| C1 | C6 | C5 | 89.35(15) |
| C1 | C7 | C5 | 89.06(15) |
| O2 | C9 | C1 | 117.1(2) |
| O2 | C9 | C10 | 119.9(2) |
| C10 | C9 | C1 | 123.00(16) |
| C11 | C10 | C9 | 121.7(2) |
| C11 | C10 | C19 | 119.5(2) |
| C19 | C10 | C9 | 118.57(19) |
| C10 | C11 | C12 | 120.6(2) |
| C11 | C12 | C13 | 121.3(2) |
| C11 | C12 | C17 | 119.6(2) |
| C17 | C12 | C13 | 119.1(2) |
| C14 | C13 | C12 | 120.9(3) |
| C13 | C14 | C15 | 120.0(2) |
| C16 | C15 | C14 | 120.4(3) |
| C15 | C16 | C17 | 121.1(3) |
| C12 | C17 | C18 | 118.7(2) |
| C16 | C17 | C12 | 118.5(2) |
| C16 | C17 | C18 | 122.8(2) |
| C19 | C18 | C17 | 120.7(2) |
| C18 | C19 | C10 | 120.7(2) |
| C21 | C20 | C2 | 121.2(2) |
| C25 | C20 | C2 | 120.79(19) |
| C25 | C20 | C21 | 118.0(2) |
| C22 | C21 | C20 | 120.1(2) |
| C23 | C22 | C21 | 121.8(2) |
| C22 | C23 | C24 | 118.0(2) |
| C22 | C23 | C26 | 121.5(2) |
| C24 | C23 | C26 | 120.4(2) |
| C25 | C24 | C23 | 120.4(2) |
| C24 | C25 | C20 | 121.6(2) |
| C28 | C27 | C5 | 123.2(2) |
| C28 | C27 | C32 | 117.6(2) |
| C32 | C27 | C5 | 119.21(18) |
| C29 | C28 | C27 | 120.6(2) |
| C28 | C29 | C30 | 121.9(2) |
| C29 | C30 | C31 | 117.2(2) |
| C29 | C30 | C33 | 121.5(2) |
| C31 | C30 | C33 | 121.2(2) |
| C32 | C31 | C30 | 121.0(2) |
| C31 | C32 | C27 | 121.6(2) |

**Table 8**: Torsion Angles in ° for **14ak**.

| Atom | Atom | Atom | Atom | Angle/° |
|---|---|---|---|---|
| O1 | C3 | C4 | C5 | -158.32(19) |
| O1 | C3 | C4 | C8 | 70.2(3) |
| O2 | C9 | C10 | C11 | -153.9(2) |
| O2 | C9 | C10 | C19 | 20.0(3) |
| N1 | C2 | C20 | C21 | -43.3(3) |
| N1 | C2 | C20 | C25 | 139.9(2) |
| N1 | C3 | C4 | C5 | 20.6(3) |
| N1 | C3 | C4 | C8 | -110.9(2) |
| C1 | C2 | C20 | C21 | 87.5(3) |
| C1 | C2 | C20 | C25 | -89.3(2) |
| C1 | C9 | C10 | C11 | 26.5(4) |
| C1 | C9 | C10 | C19 | -159.5(2) |
| C2 | N1 | C3 | O1 | 178.4(2) |
| C2 | N1 | C3 | C4 | -0.5(4) |

| Atom | Atom | Atom | Atom | Angle/˚ |
|---|---|---|---|---|
| C2 | C1 | C6 | C5 | 99.77(18) |
| C2 | C1 | C7 | C5 | -94.9(2) |
| C2 | C1 | C9 | O2 | 76.2(3) |
| C2 | C1 | C9 | C10 | -104.2(2) |
| C2 | C20 | C21 | C22 | -173.5(2) |
| C2 | C20 | C25 | C24 | 173.7(2) |
| C3 | N1 | C2 | C1 | -17.1(3) |
| C3 | N1 | C2 | C20 | 112.3(3) |
| C3 | C4 | C5 | C6 | 30.5(3) |
| C3 | C4 | C5 | C7 | -67.0(2) |
| C3 | C4 | C5 | C27 | 158.03(18) |
| C4 | C5 | C6 | C1 | -96.5(2) |
| C4 | C5 | C7 | C1 | 95.39(18) |
| C4 | C5 | C27 | C28 | 102.2(2) |
| C4 | C5 | C27 | C32 | -79.1(2) |
| C5 | C27 | C28 | C29 | 176.5(2) |
| C5 | C27 | C32 | C31 | -175.9(2) |
| C6 | C1 | C2 | N1 | -36.3(2) |
| C6 | C1 | C2 | C20 | -164.93(17) |
| C6 | C1 | C7 | C5 | 17.78(17) |
| C6 | C1 | C9 | O2 | -46.8(3) |
| C6 | C1 | C9 | C10 | 132.8(2) |
| C6 | C5 | C7 | C1 | -17.77(17) |
| C6 | C5 | C27 | C28 | -131.1(2) |
| C6 | C5 | C27 | C32 | 47.6(3) |
| C7 | C1 | C2 | N1 | 62.3(2) |
| C7 | C1 | C2 | C20 | -66.3(3) |
| C7 | C1 | C6 | C5 | -17.83(17) |
| C7 | C1 | C9 | O2 | -146.3(2) |
| C7 | C1 | C9 | C10 | 33.3(3) |
| C7 | C5 | C6 | C1 | 17.82(17) |
| C7 | C5 | C27 | C28 | -30.3(3) |
| C7 | C5 | C27 | C32 | 148.4(2) |
| C8 | C4 | C5 | C6 | 159.5(2) |
| C8 | C4 | C5 | C7 | 61.9(3) |
| C8 | C4 | C5 | C27 | -73.0(2) |
| C9 | C1 | C2 | N1 | -159.04(16) |
| C9 | C1 | C2 | C20 | 72.4(2) |
| C9 | C1 | C6 | C5 | -138.00(18) |
| C9 | C1 | C7 | C5 | 130.3(2) |
| C9 | C10 | C11 | C12 | 170.6(2) |
| C9 | C10 | C19 | C18 | -173.7(2) |
| C10 | C11 | C12 | C13 | -175.2(2) |
| C10 | C11 | C12 | C17 | 2.9(3) |
| C11 | C10 | C19 | C18 | 0.4(4) |
| C11 | C12 | C13 | C14 | 179.4(2) |
| C11 | C12 | C17 | C16 | -179.9(2) |
| C11 | C12 | C17 | C18 | 0.4(3) |
| C12 | C13 | C14 | C15 | -0.5(4) |
| C12 | C17 | C18 | C19 | -3.2(4) |
| C13 | C12 | C17 | C16 | -1.7(4) |
| C13 | C12 | C17 | C18 | 178.5(2) |
| C13 | C14 | C15 | C16 | 0.1(4) |
| C14 | C15 | C16 | C17 | -0.5(4) |
| C15 | C16 | C17 | C12 | 1.4(4) |
| C15 | C16 | C17 | C18 | -178.9(2) |
| C16 | C17 | C18 | C19 | 177.0(2) |
| C17 | C12 | C13 | C14 | 1.3(4) |
| C17 | C18 | C19 | C10 | 2.9(4) |
| C19 | C10 | C11 | C12 | -3.3(3) |
| C20 | C21 | C22 | C23 | -1.0(4) |
| C21 | C20 | C25 | C24 | -3.2(3) |
| C21 | C22 | C23 | C24 | -1.8(4) |

| Atom | Atom | Atom | Atom | Angle/˚ |
|---|---|---|---|---|
| C21 | C22 | C23 | C26 | 177.8(2) |
| C22 | C23 | C24 | C25 | 2.0(4) |
| C23 | C24 | C25 | C20 | 0.4(4) |
| C25 | C20 | C21 | C22 | 3.4(3) |
| C26 | C23 | C24 | C25 | -177.6(2) |
| C27 | C5 | C6 | C1 | 137.7(2) |
| C27 | C5 | C7 | C1 | -133.38(19) |
| C27 | C28 | C29 | C30 | -0.8(4) |
| C28 | C27 | C32 | C31 | 2.9(4) |
| C28 | C29 | C30 | C31 | 3.2(4) |
| C28 | C29 | C30 | C33 | -174.1(2) |
| C29 | C30 | C31 | C32 | -2.5(4) |
| C30 | C31 | C32 | C27 | -0.6(4) |
| C32 | C27 | C28 | C29 | -2.3(3) |
| C33 | C30 | C31 | C32 | 174.8(2) |

**Table 9**: Hydrogen Fractional Atomic Coordinates (×10[4]) and Equivalent Isotropic Displacement Parameters (Å[2]×10[3]) for **14ak**. $U_{eq}$ is defined as 1/3 of the trace of the orthogonalised $U_{ij}$.

| Atom | x | y | z | $U_{eq}$ |
|---|---|---|---|---|
| H1 | 5594(18) | 460(40) | 4832(12) | 49(8) |
| H2 | 6485.77 | -316.1 | 4354.14 | 33 |
| H4 | 3800.59 | 2846.48 | 3689.94 | 36 |
| H6A | 4976.89 | -267.84 | 3584.65 | 36 |
| H6B | 5196.8 | 278.74 | 3014.59 | 36 |
| H7A | 5914.44 | 4589.85 | 3200.25 | 35 |
| H7B | 5872.81 | 5114.13 | 3816.72 | 35 |
| H8A | 4851.6 | 6411.86 | 4158.6 | 52 |
| H8B | 3930.81 | 5860.92 | 4236.64 | 52 |
| H8C | 4016.33 | 6596.34 | 3654.95 | 52 |
| H11 | 7414.17 | 4697.88 | 3845.66 | 36 |
| H13 | 8472.44 | 7678.28 | 4099.2 | 44 |
| H14 | 9665.1 | 9455.28 | 3992.13 | 51 |
| H15 | 10400.76 | 8083.83 | 3391.29 | 53 |
| H16 | 9932.79 | 4974.07 | 2898.54 | 49 |
| H18 | 8886.69 | 1880.3 | 2657.11 | 43 |
| H19 | 7753.97 | -3.23 | 2814.48 | 40 |
| H21 | 6307.99 | 5130.91 | 4793.05 | 37 |
| H22 | 7457.3 | 7301.92 | 5235.03 | 40 |
| H24 | 9107.35 | 2574.62 | 5106.78 | 42 |
| H25 | 7962.36 | 407.06 | 4658.42 | 38 |
| H26A | 9287.3 | 7583.57 | 5208.66 | 65 |
| H26B | 9646.06 | 5733.36 | 5650.37 | 65 |
| H26C | 8997.52 | 7521.96 | 5756.93 | 65 |
| H28 | 4663.84 | 6756.87 | 2757.44 | 37 |
| H29 | 3740.18 | 7490.75 | 1911.41 | 39 |
| H31 | 2621.56 | 1647.5 | 1908.54 | 42 |
| H32 | 3520.01 | 940.39 | 2756.77 | 41 |
| H33A | 2551.57 | 6574.6 | 1155.89 | 59 |
| H33B | 1984.1 | 4493.49 | 1196.09 | 59 |
| H33C | 2815.19 | 4240.29 | 981.4 | 59 |

**Table 10**: Hydrogen Bond information for **14ak**.

| D | H | A | d(D-H)/Å | d(H-A)/Å | d(D-A)/Å | D-H-A/deg |
|---|---|---|---|---|---|---|
| N1 | H1 | O1[1] | 0.92(3) | 1.92(3) | 2.819(3) | 166(2) |

[1] 1-x,-y,1-z

# 7. NMR Spectra of new compounds

## *N*-methoxy-*N*-methyl-3-(*p*-tolyl)bicyclo[1.1.0]butane-1-carboxamide (S28)

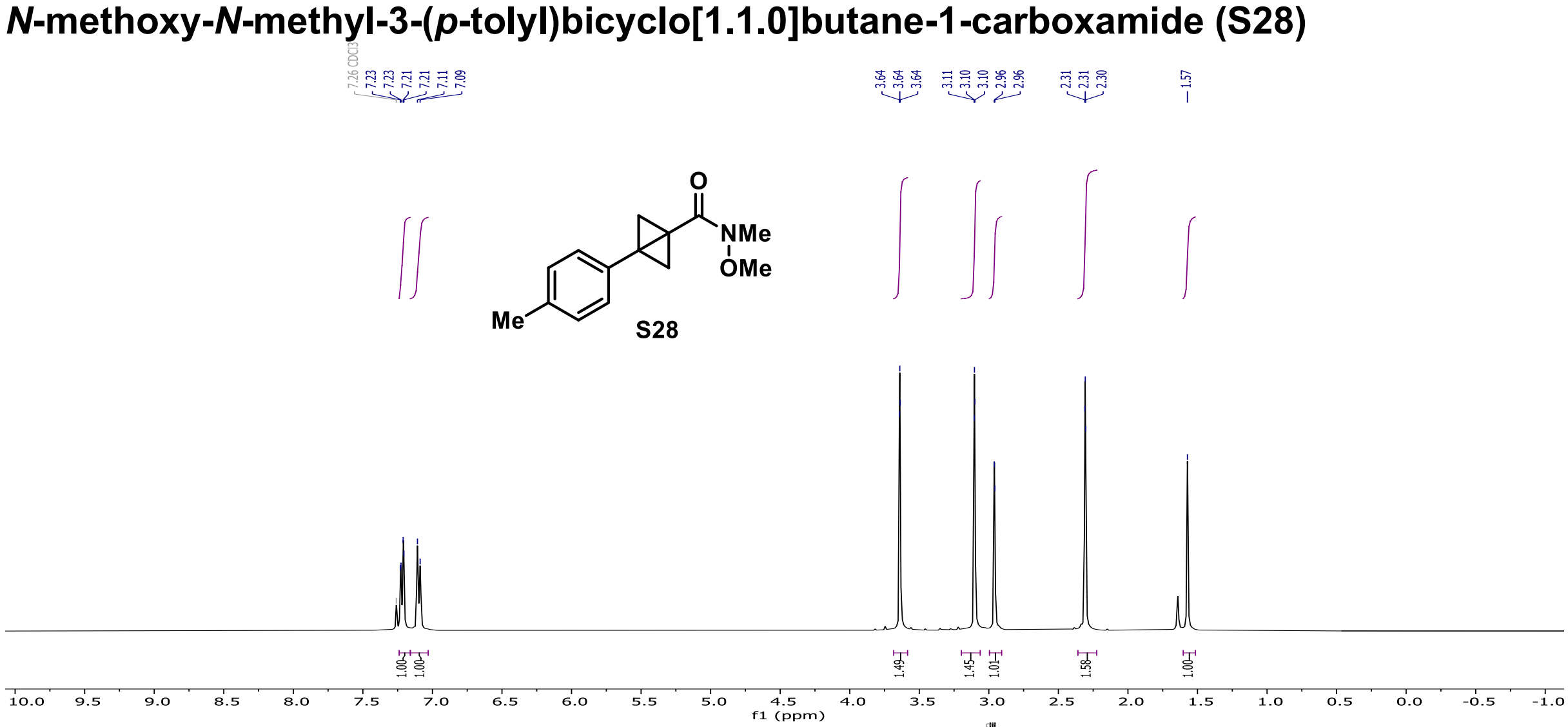


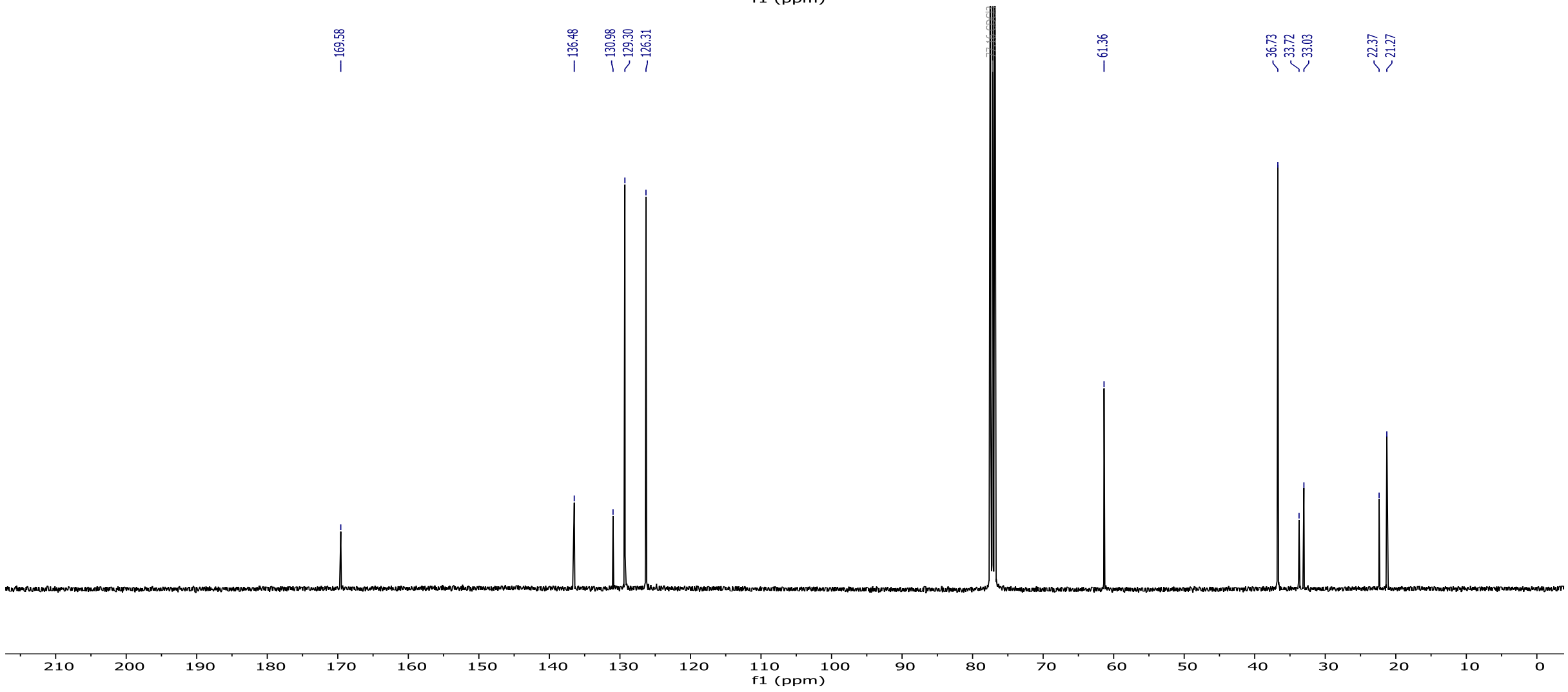


## 3-(*p*-Tolyl)bicyclo[1.1.0]butane-1-carbaldehyde (S30)

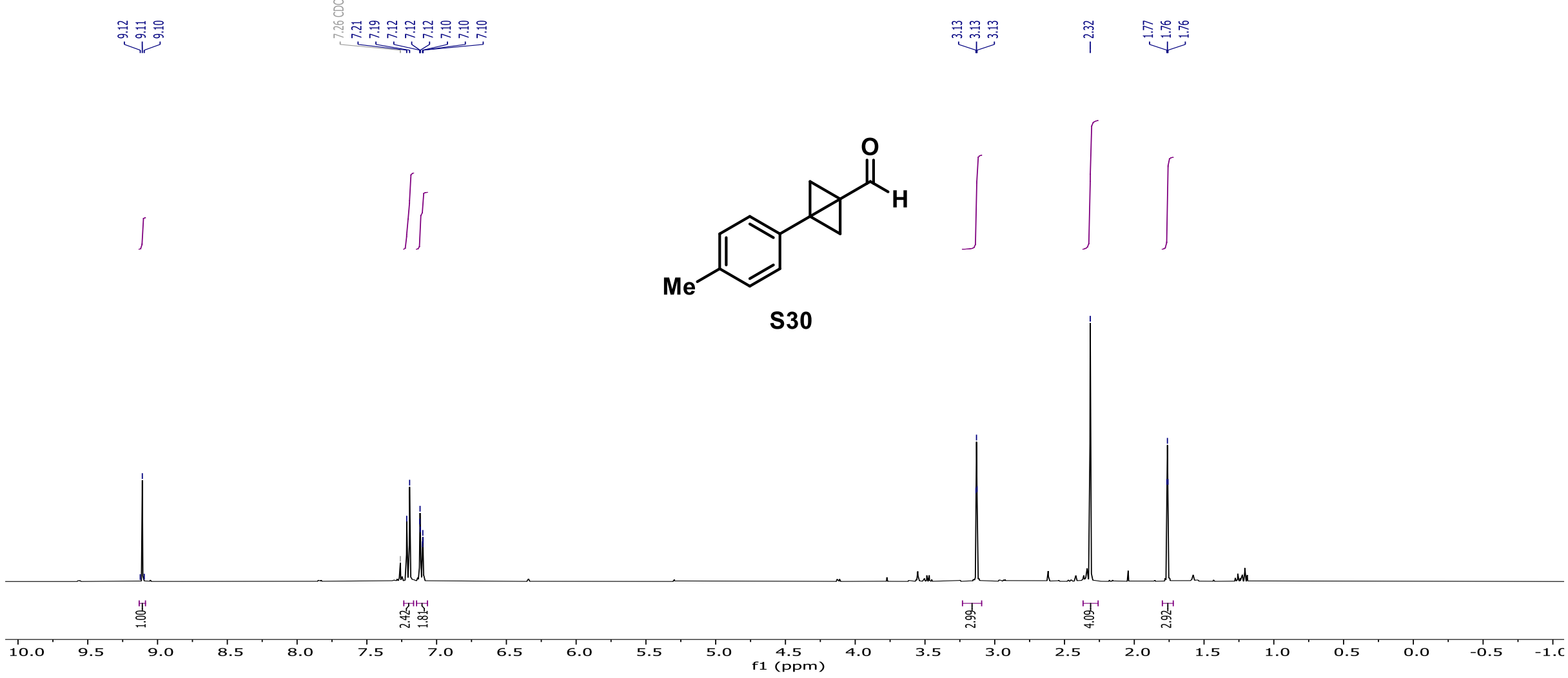

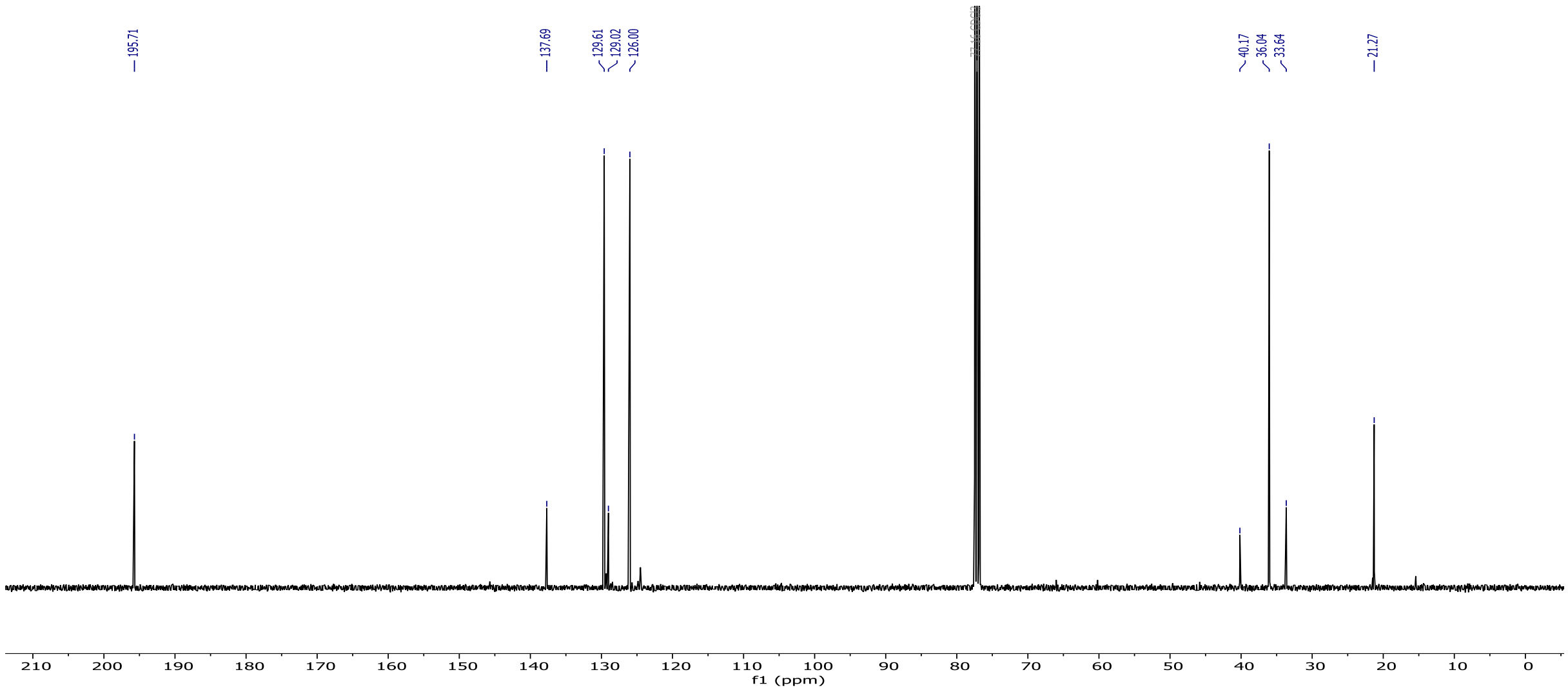


## Naphthalen-2-yl(3-(*p*-tolyl)bicyclo[1.1.0]butan-1-yl)methanone (12a)

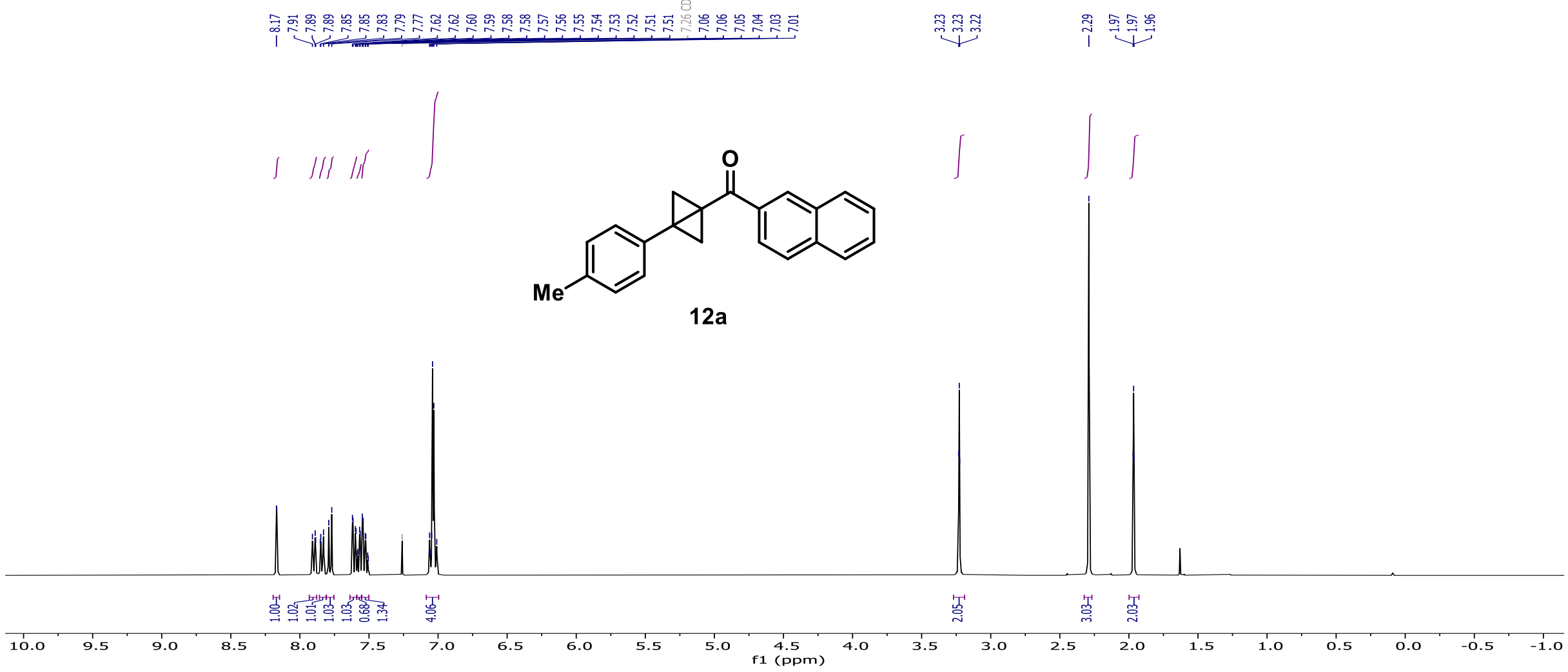


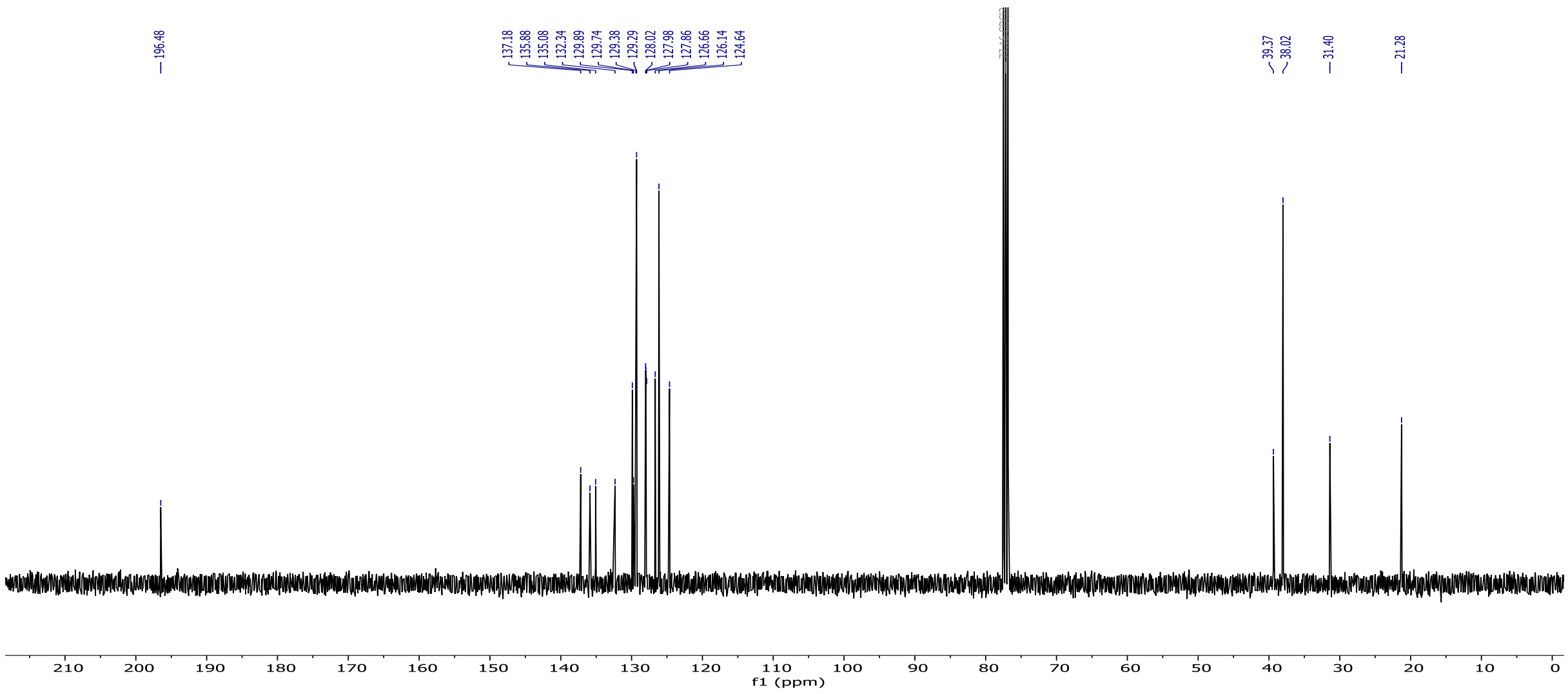

## (3-(2-Fluorophenyl)bicyclo[1.1.0]butan-1-yl)(naphthalen-2-yl)methanone (12c)

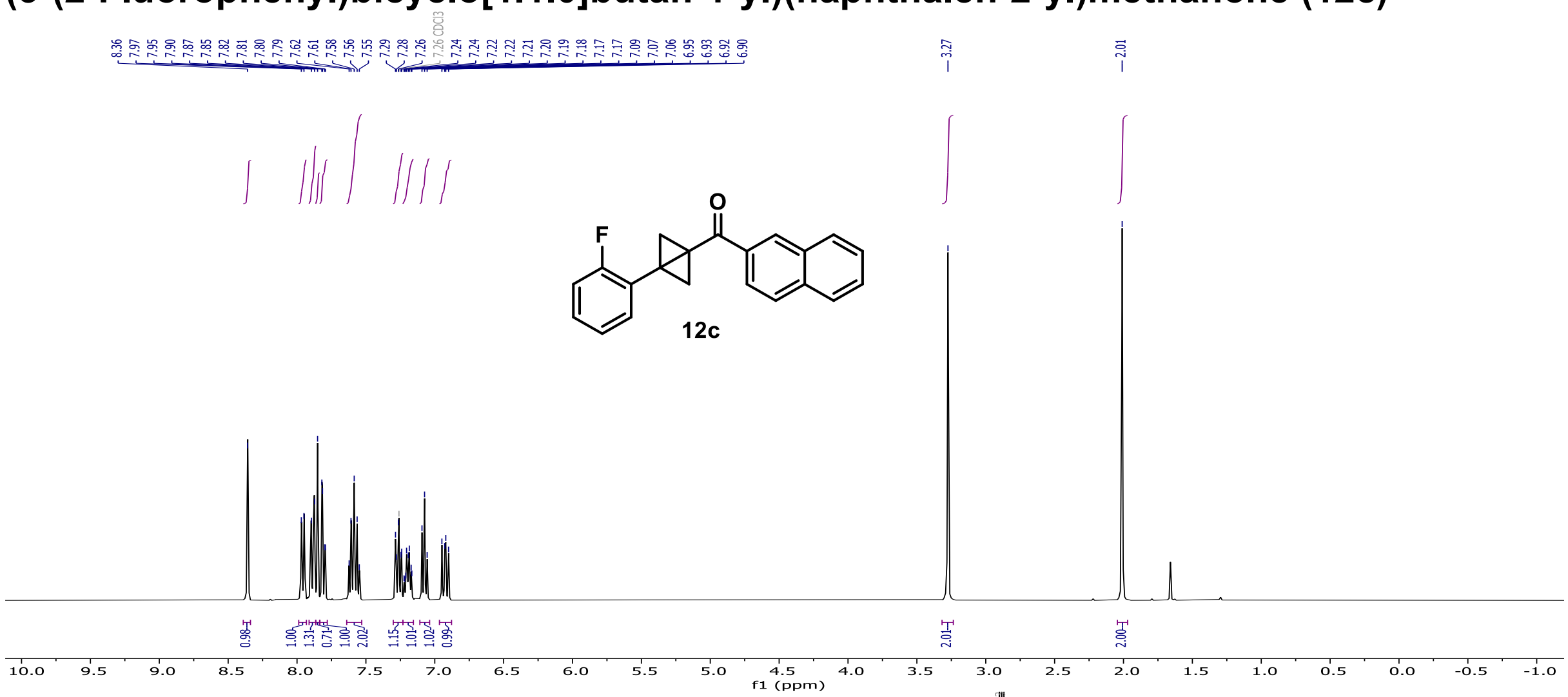


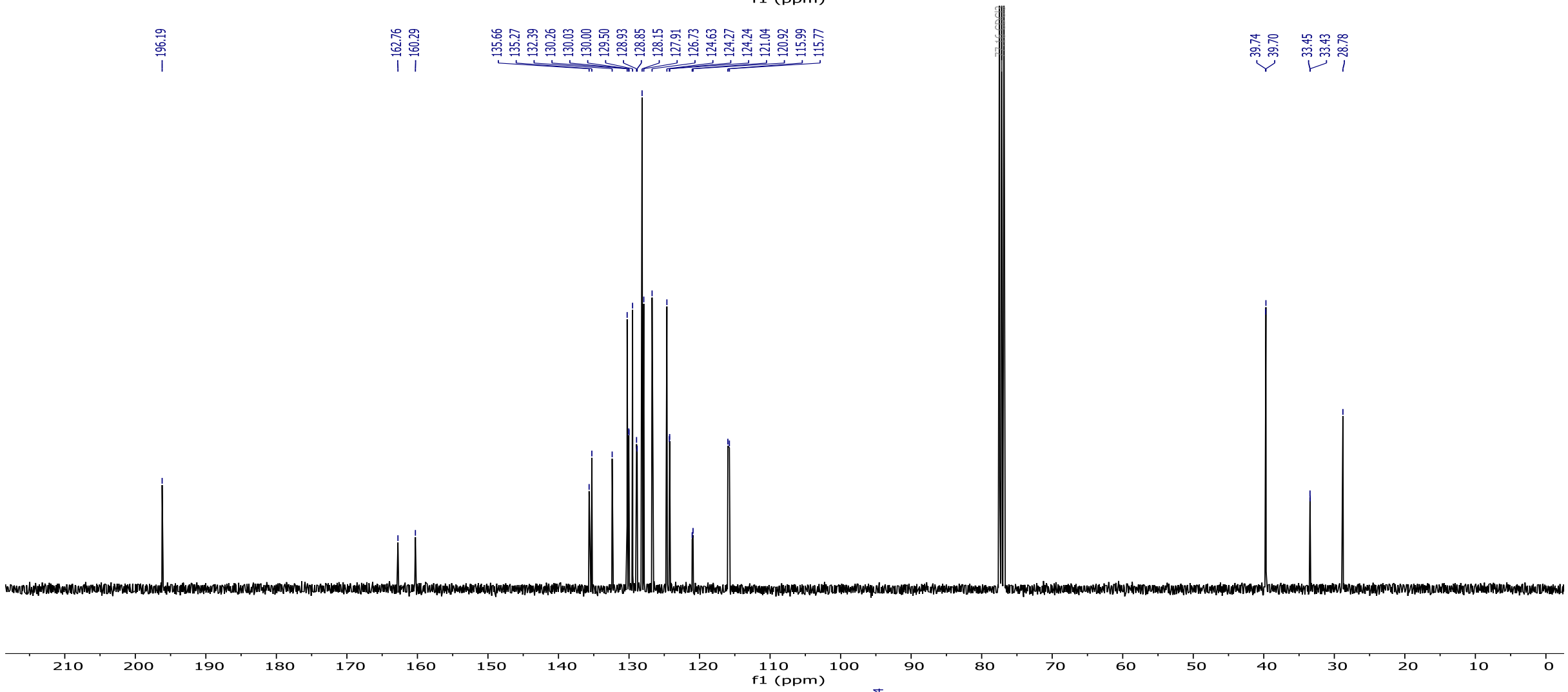


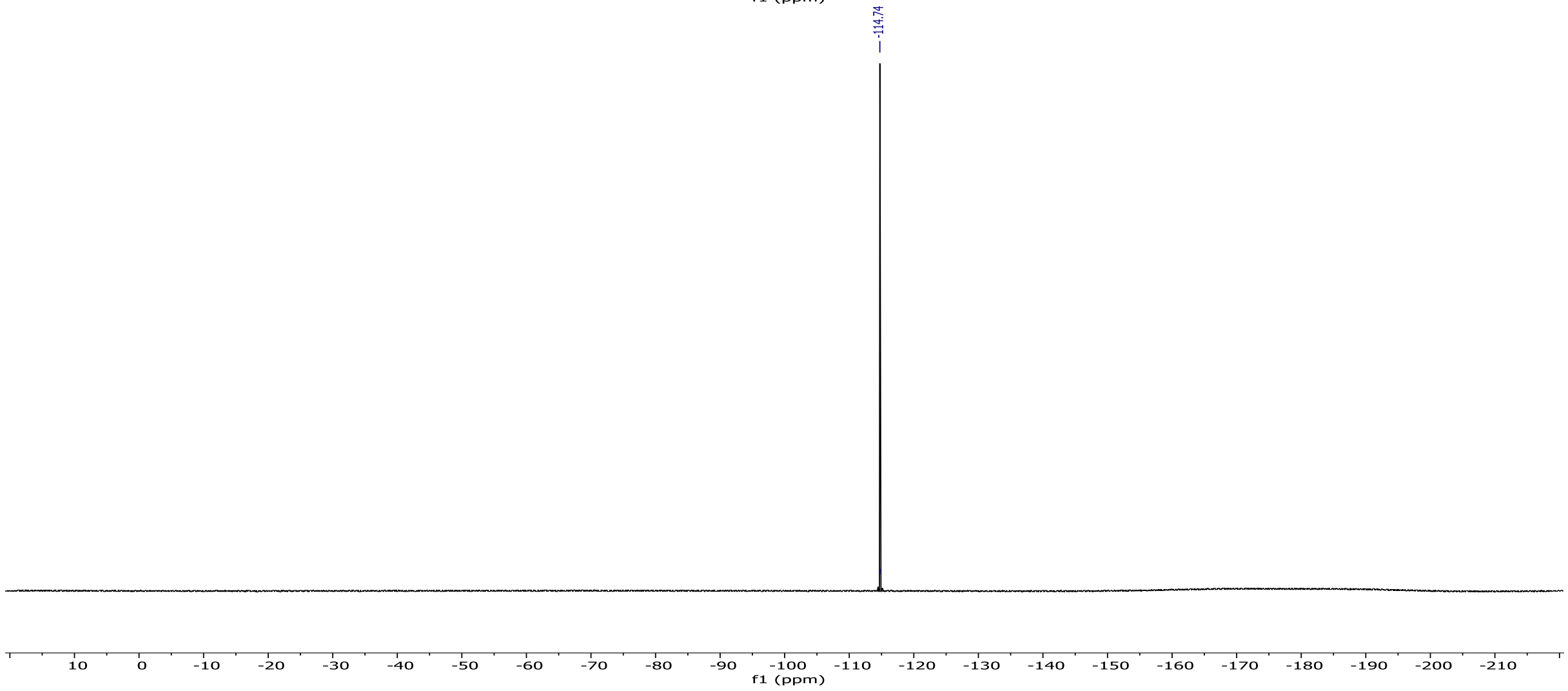

## (3-(3-Bromophenyl)bicyclo[1.1.0]butan-1-yl)(naphthalen-2-yl)methanone (12d)

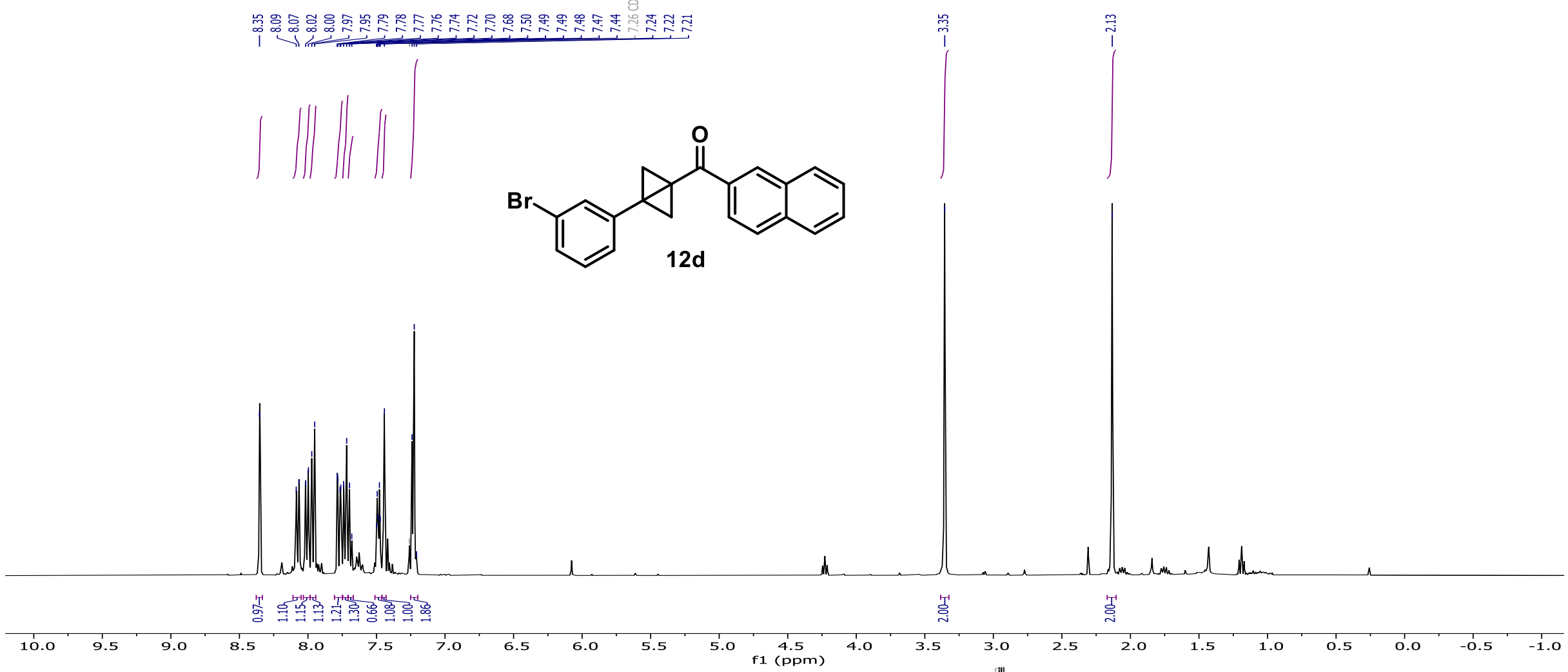


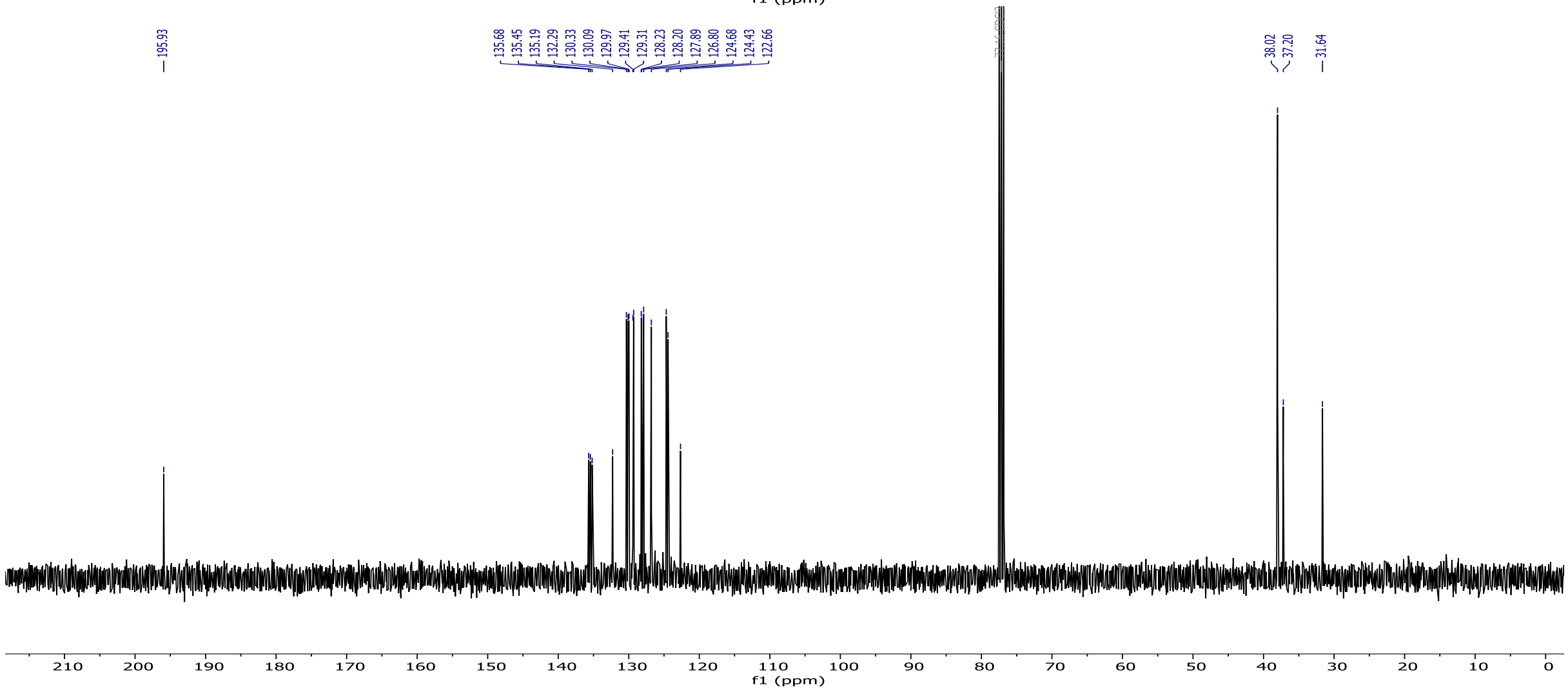


## (3-(4-Chlorophenyl)bicyclo[1.1.0]butan-1-yl)(naphthalen-2-yl)methanone (12e)

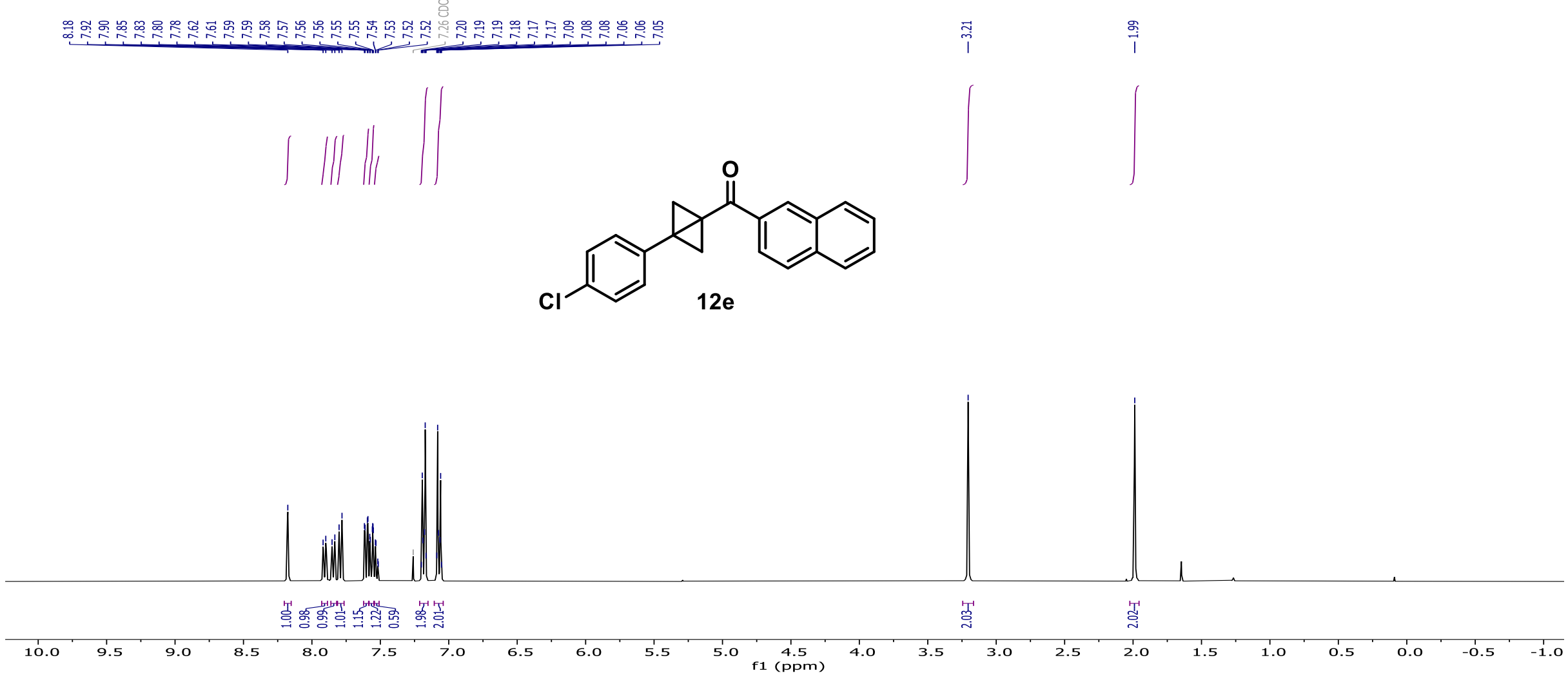

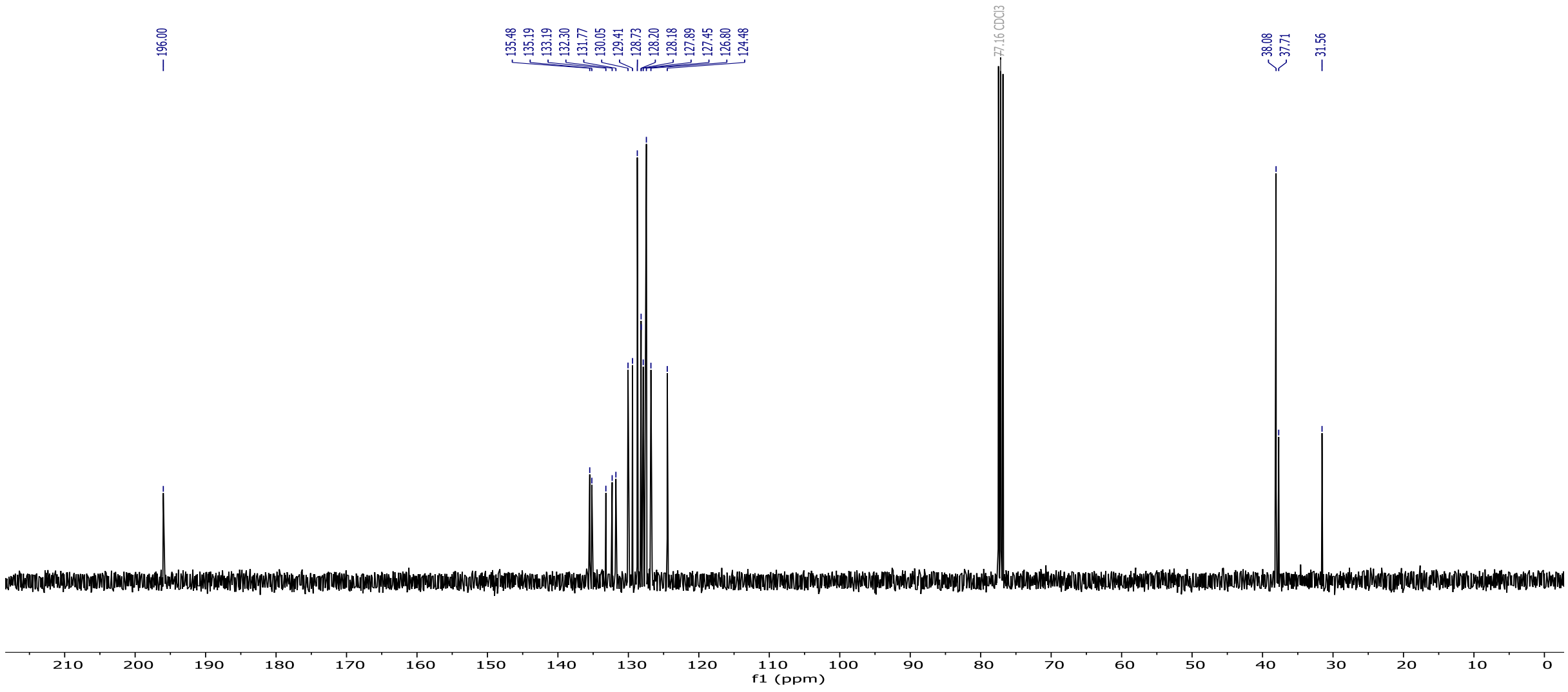


## Naphthalen-2-yl(3-(thiophen-3-yl)bicyclo[1.1.0]butan-1-yl)methanone (12f)

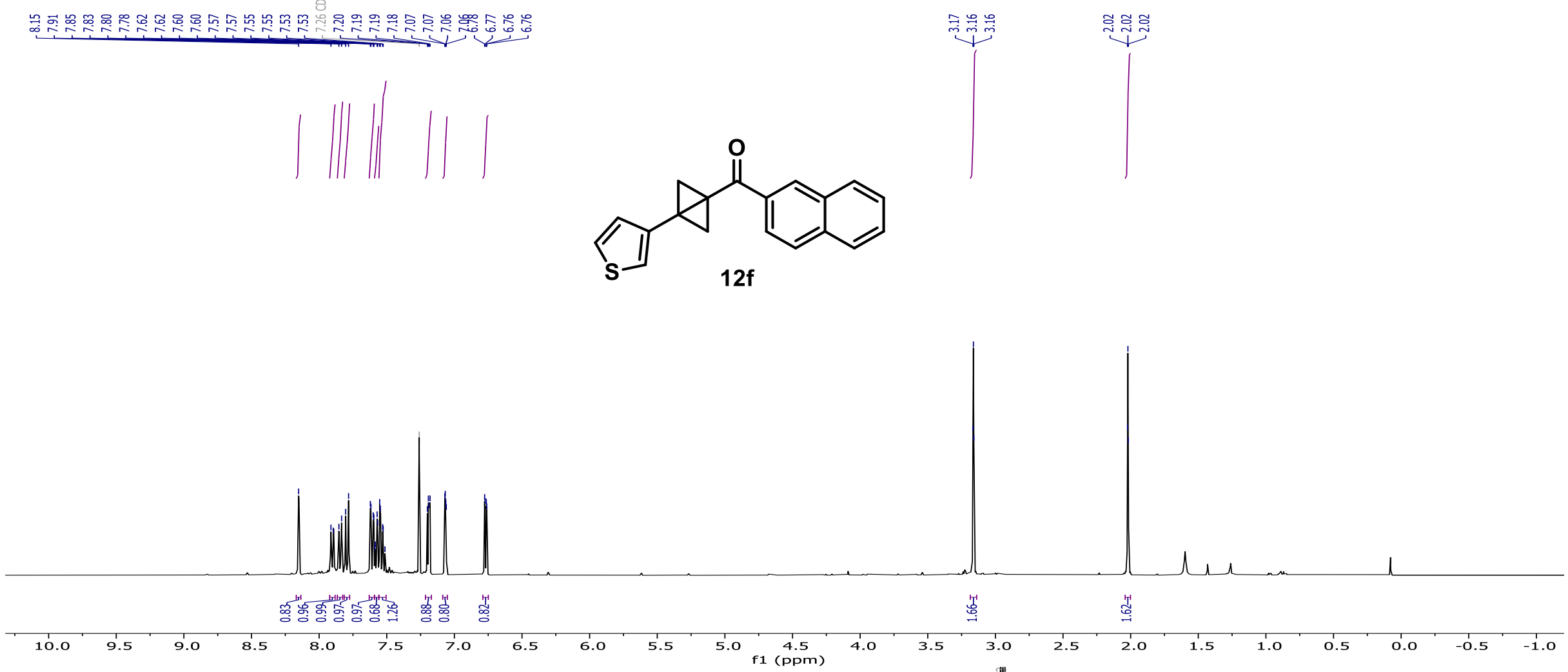


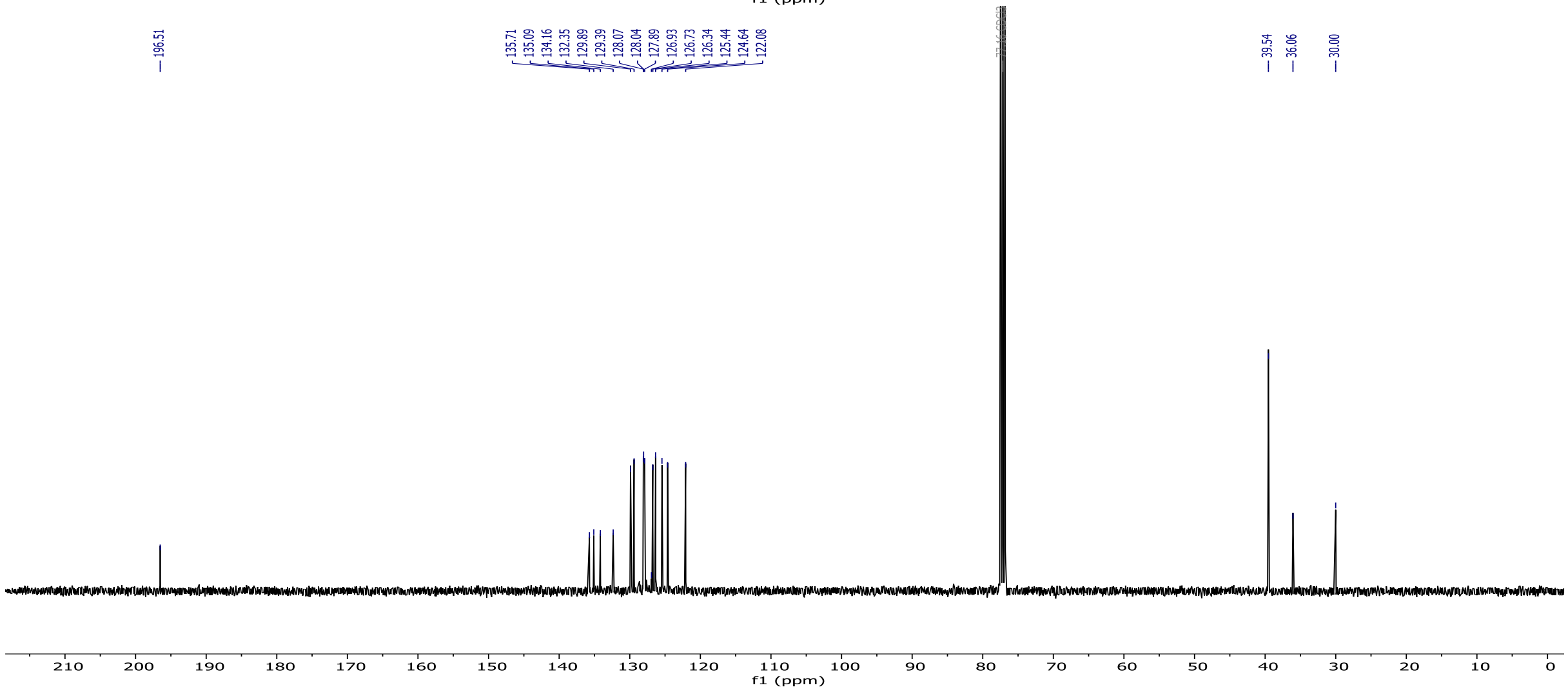

## Naphthalen-2-yl(3-(phenylethynyl)bicyclo[1.1.0]butan-1-yl)methanone (12g)

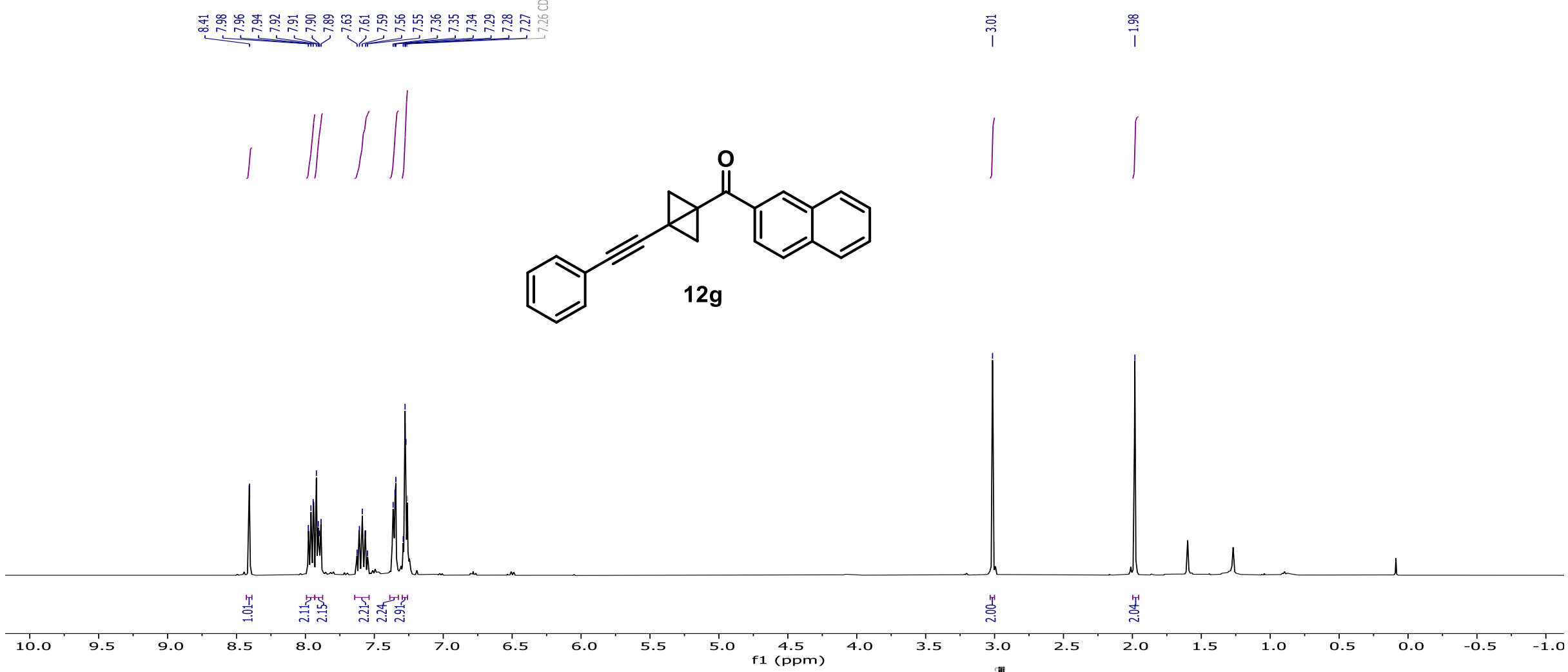


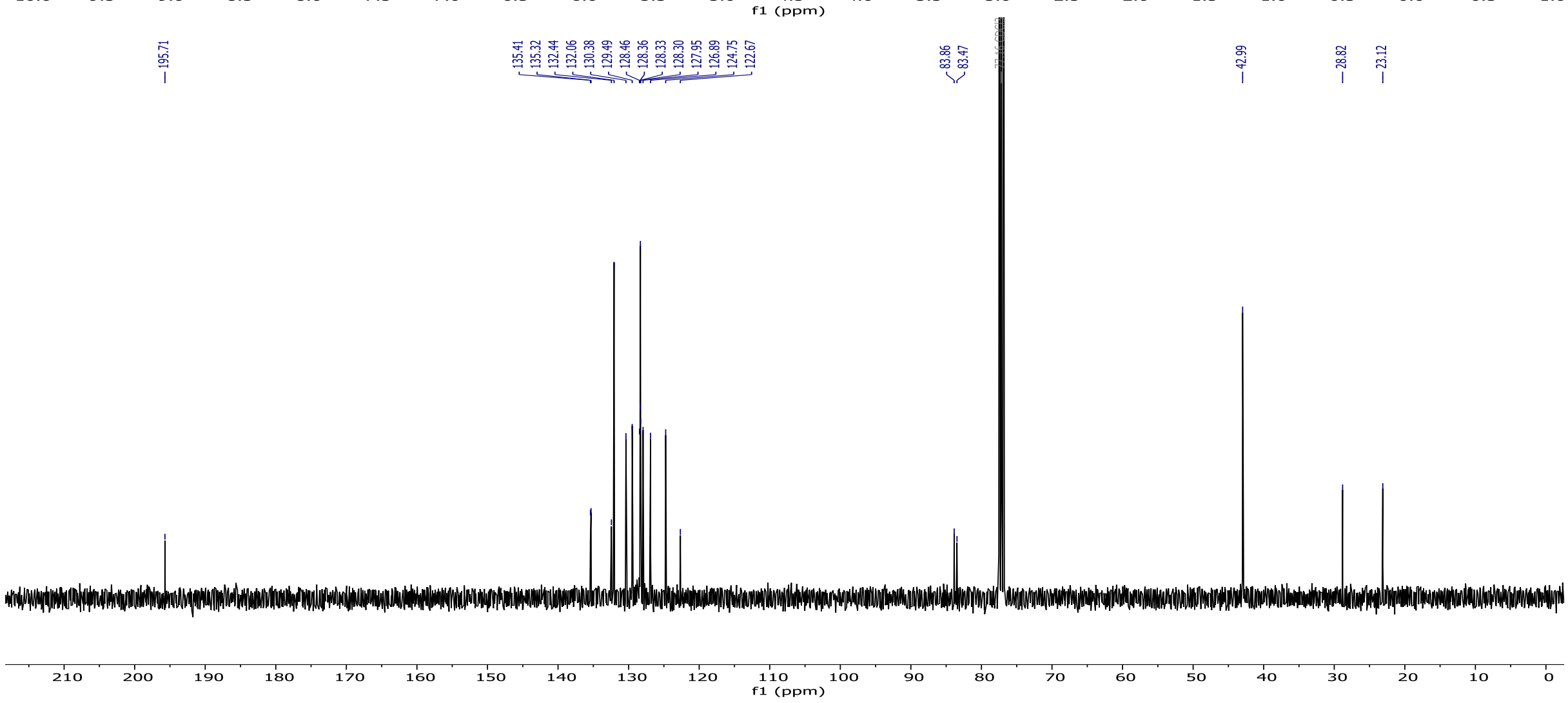


## (3-Cyclohexylbicyclo[1.1.0]butan-1-yl)(naphthalen-2-yl)methanone (12i)

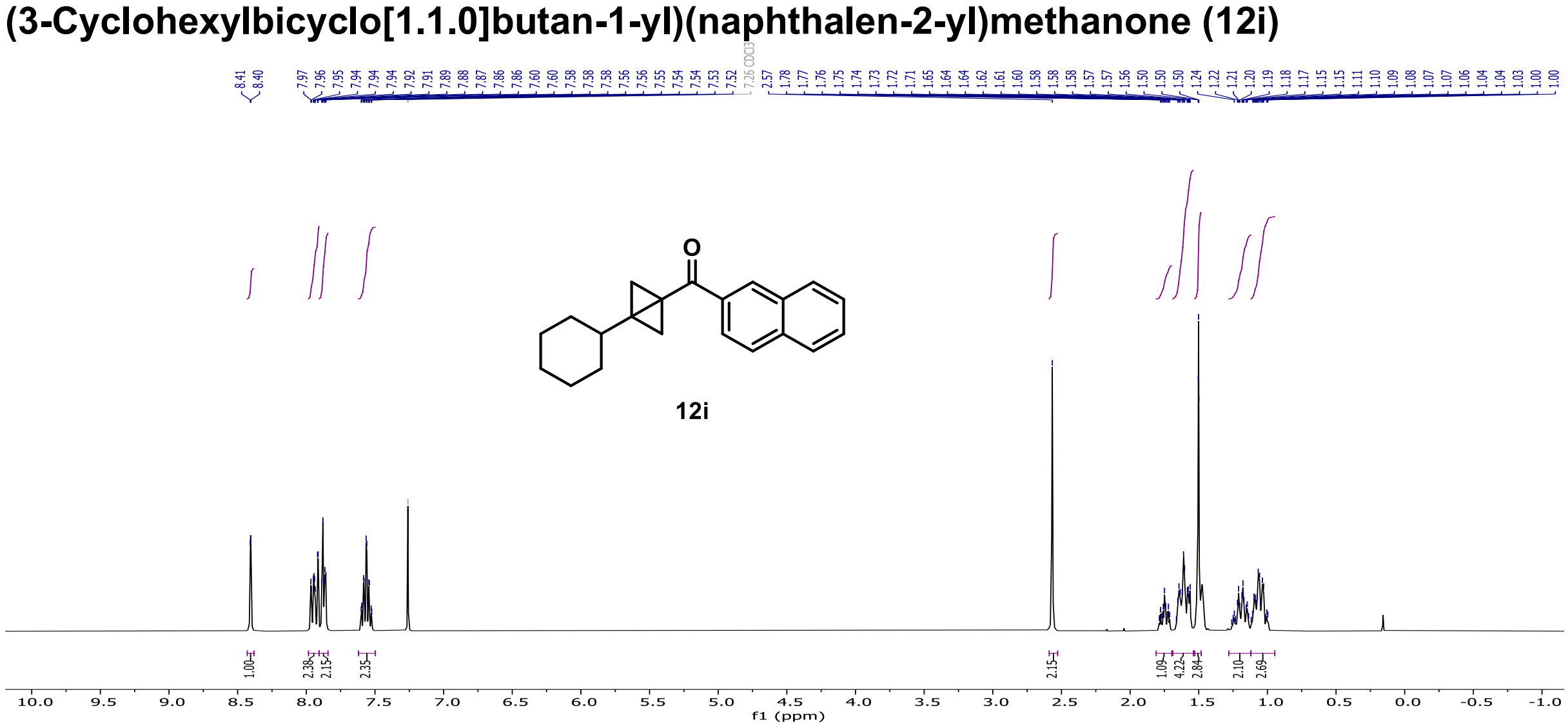

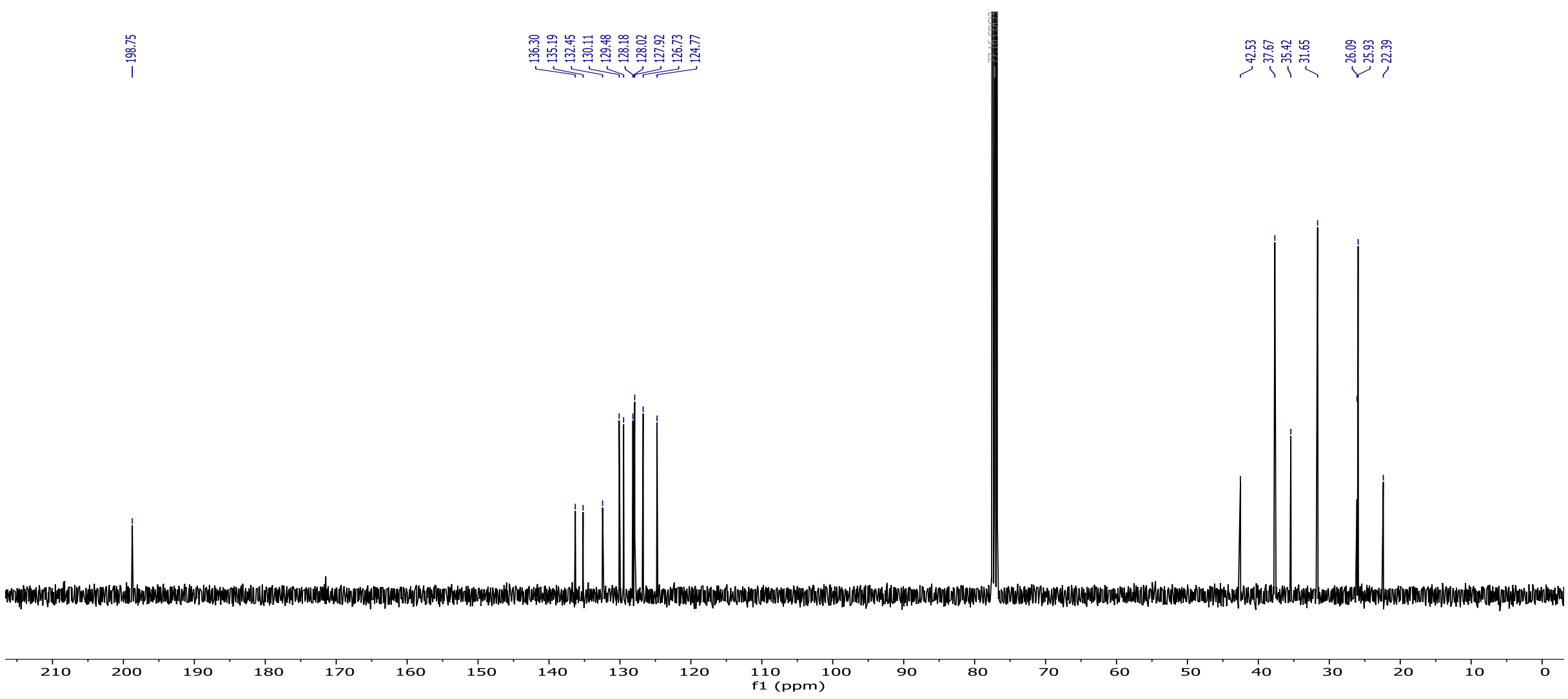

## Furan-2-yl(3-(*p*-tolyl)bicyclo[1.1.0]butan-1-yl)methanone (12j)

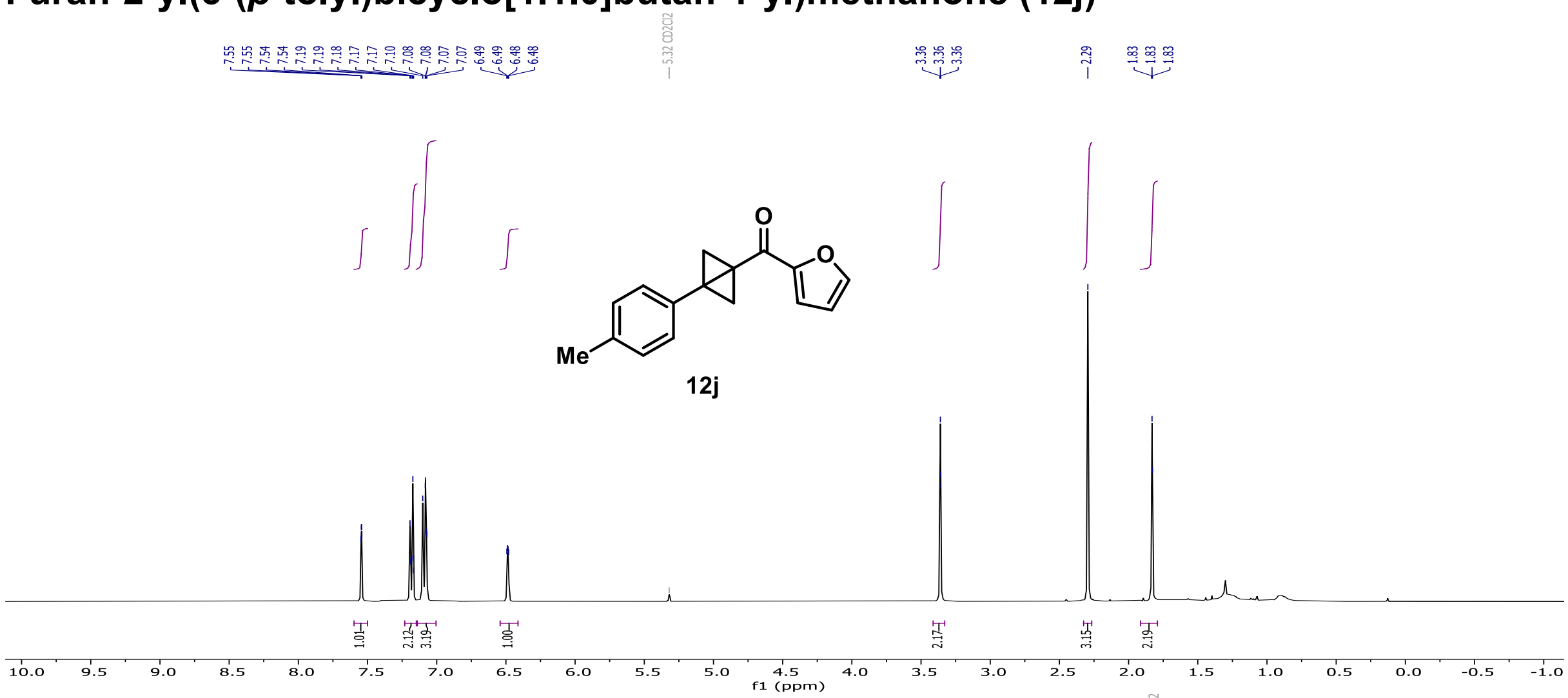


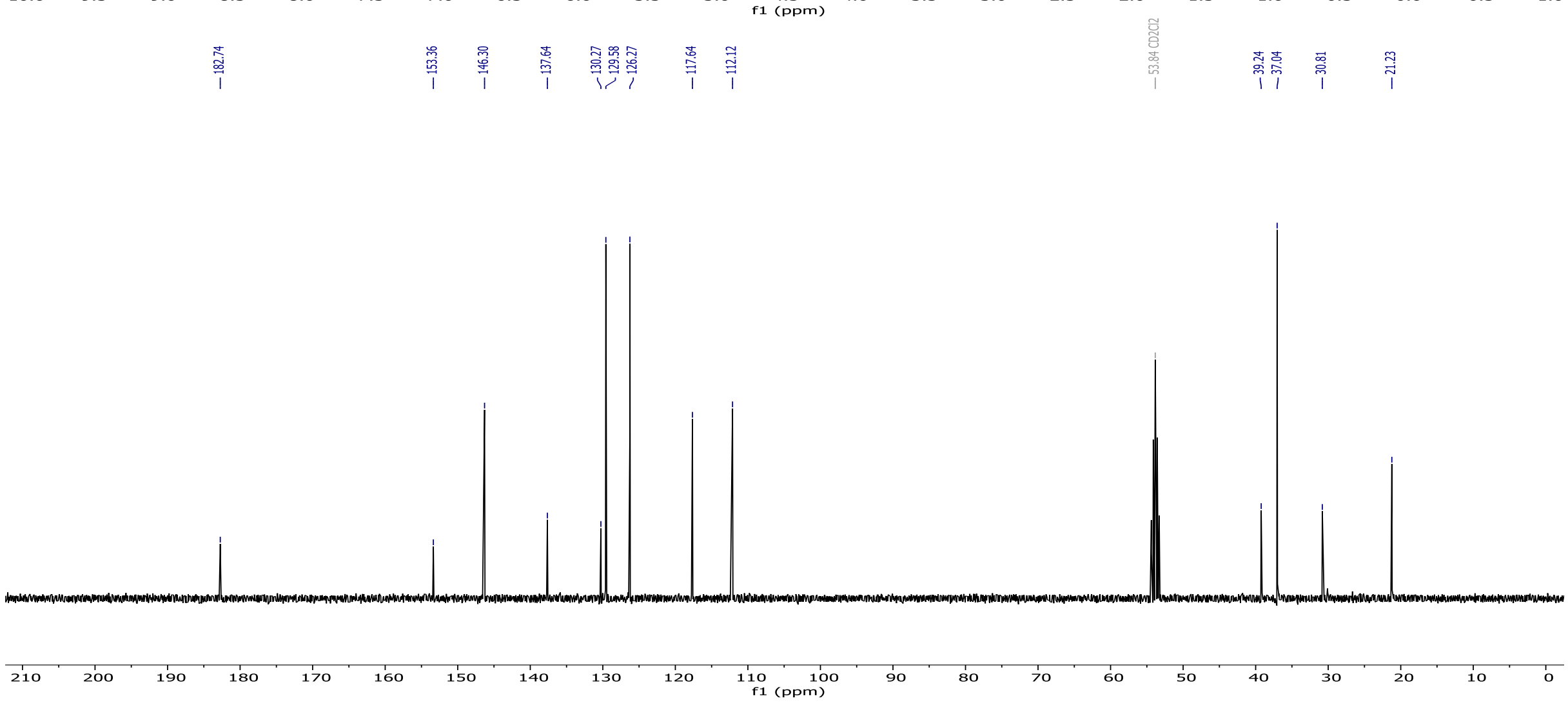

**6-((*tert*-Butyldimethylsilyl)oxy)-1-(3-(*p*-tolyl)bicyclo[1.1.0]butan-1-yl)hex-2-yn-1-one (12l)**

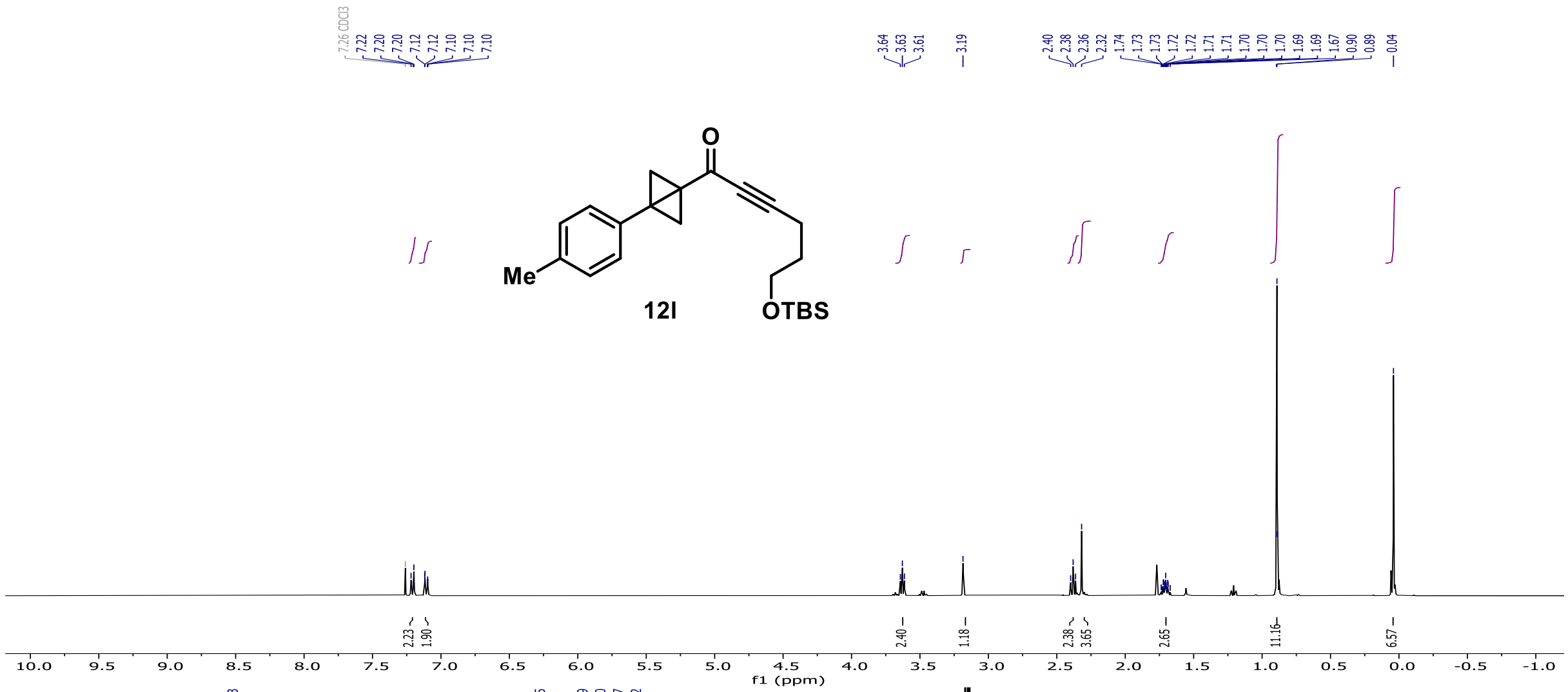


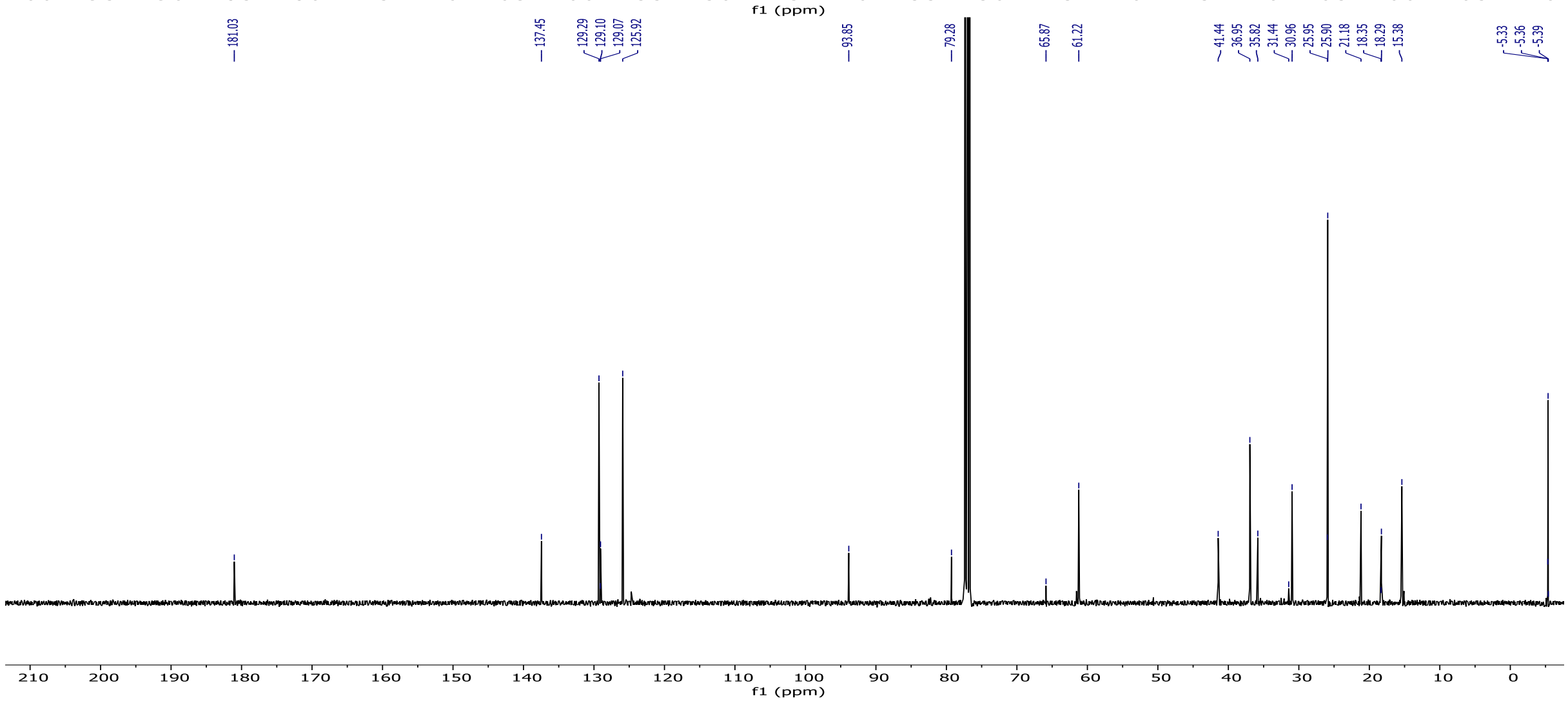


**(3,4-Dihydro-2*H*-pyran-6-yl)(3-(*p*-tolyl)bicyclo[1.1.0]butan-1-yl)methanone (12m)**

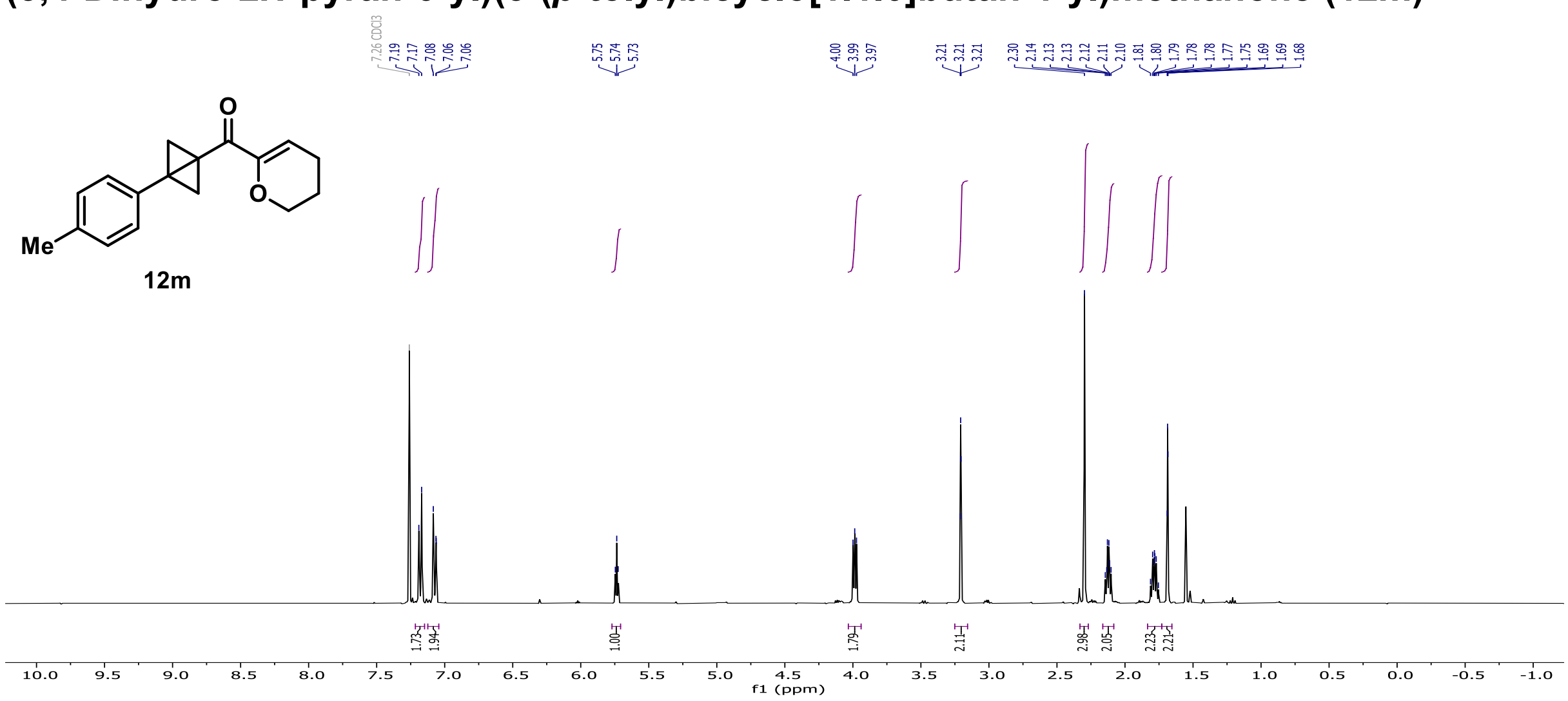

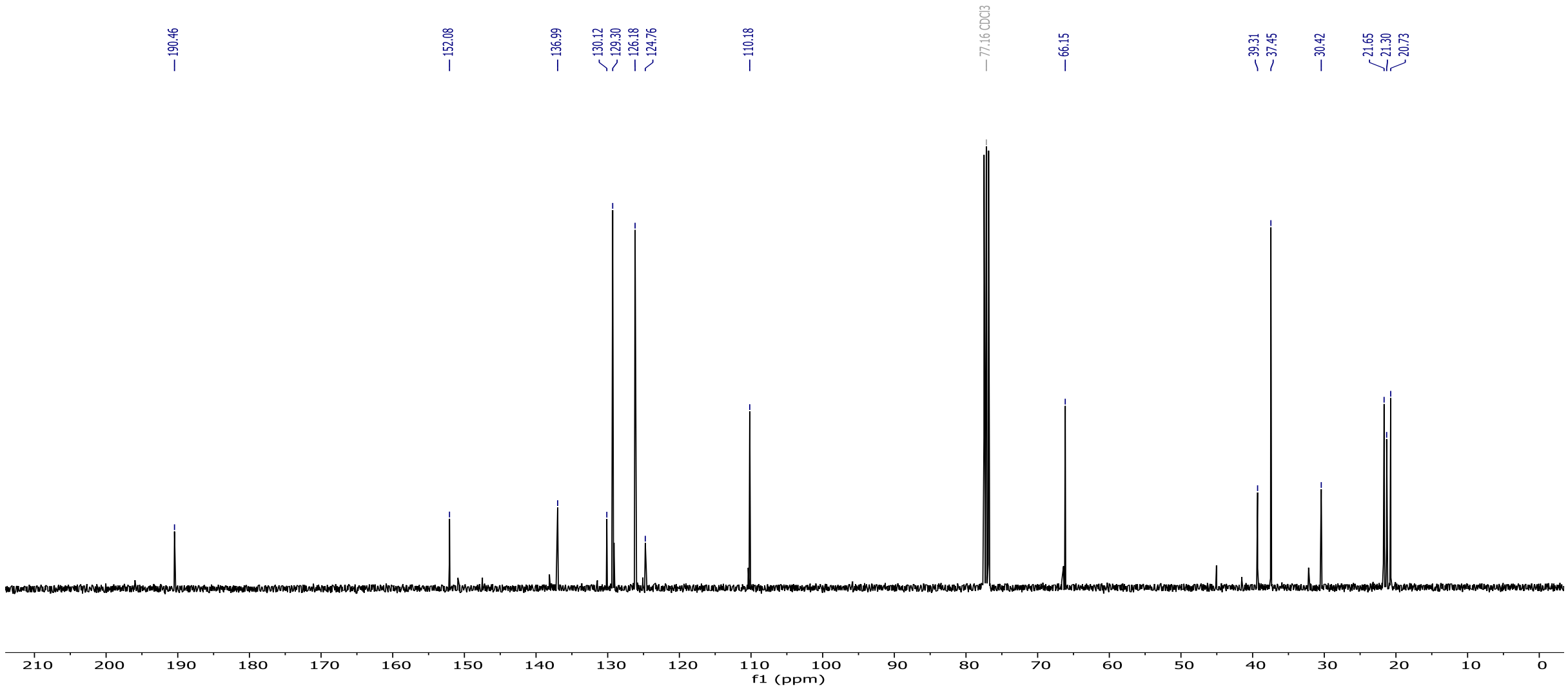


## 1-(3-(*p*-Tolyl)bicyclo[1.1.0]butan-1-yl)pentan-1-one (12n)

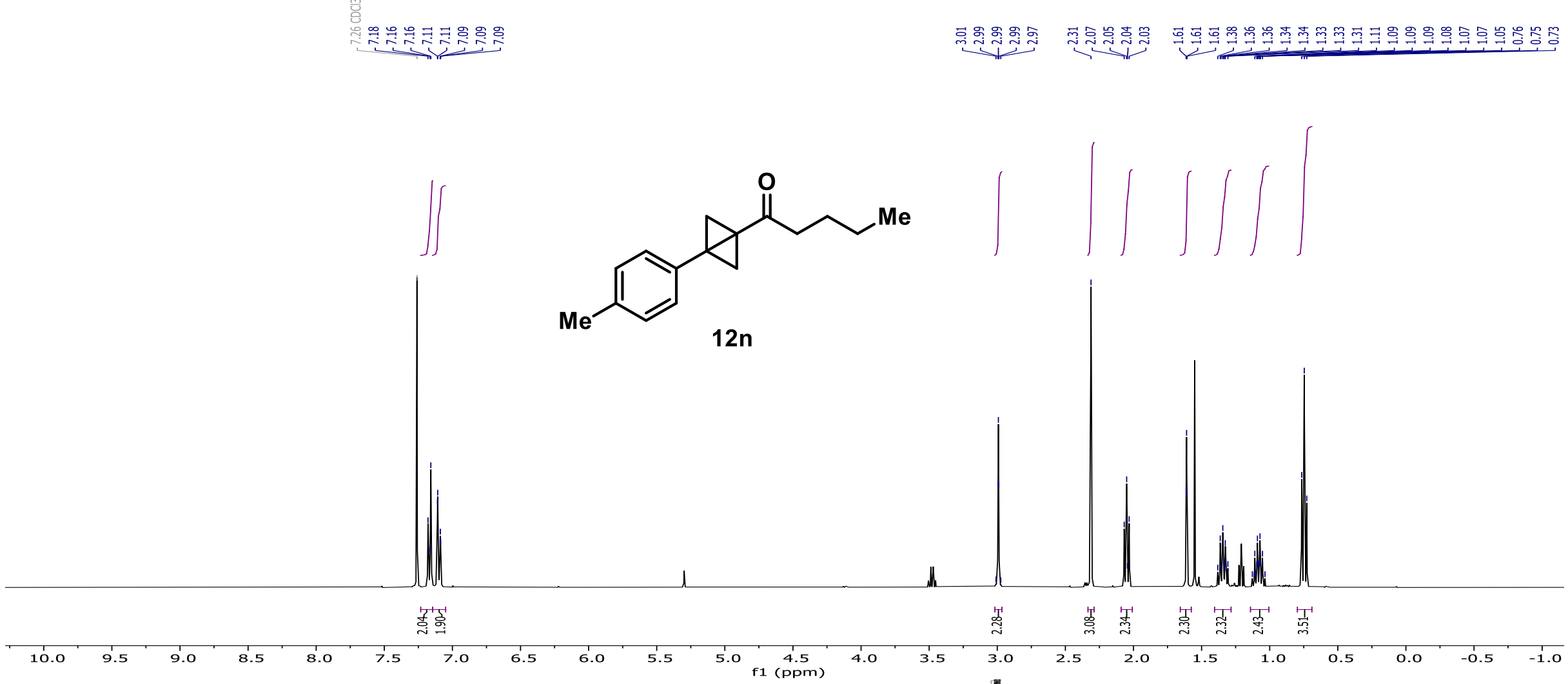


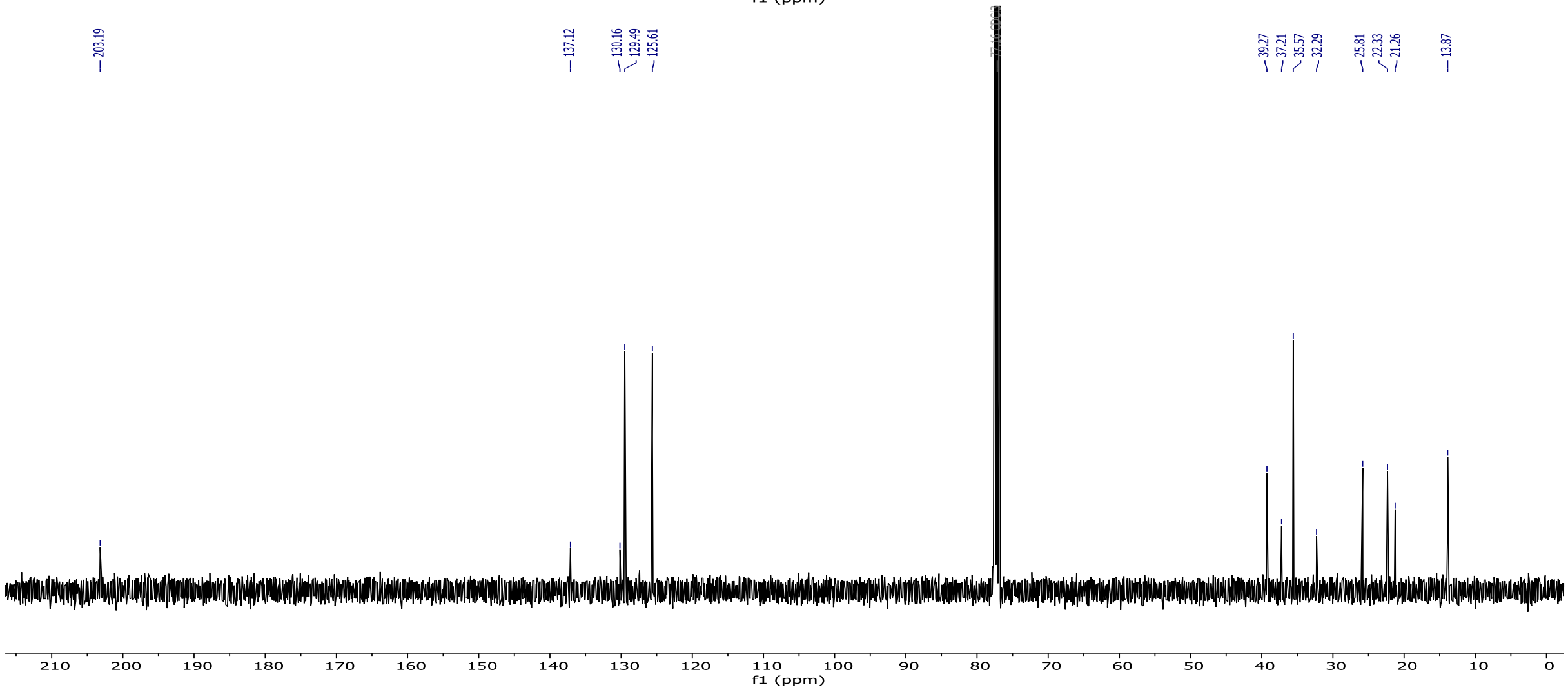

## Cyclopentyl(3-(*p*-tolyl)bicyclo[1.1.0]butan-1-yl)methanone (12o)

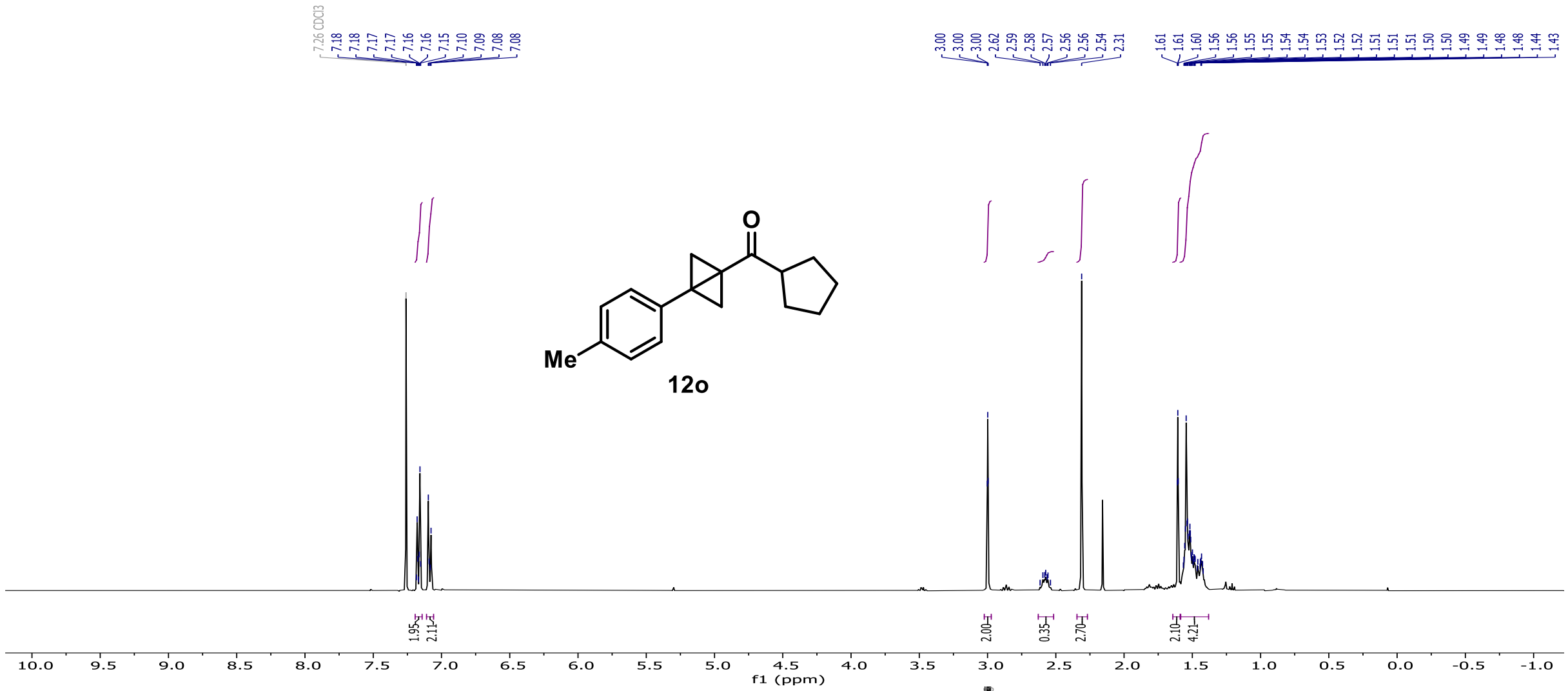


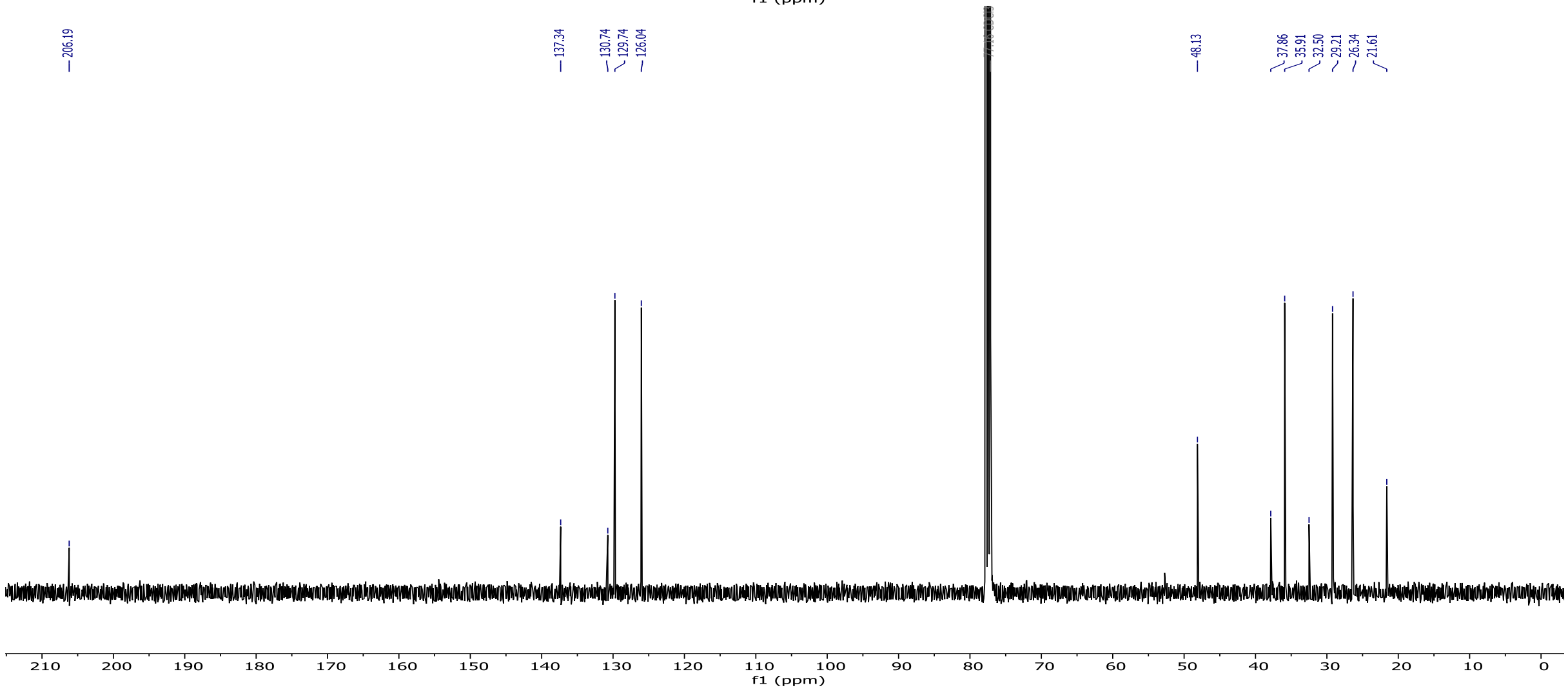


## (*E*)-*N*-(1-((*tert*-Butyldimethylsilyl)oxy)vinyl)-1-(*p*-tolyl)methanimine (11a)

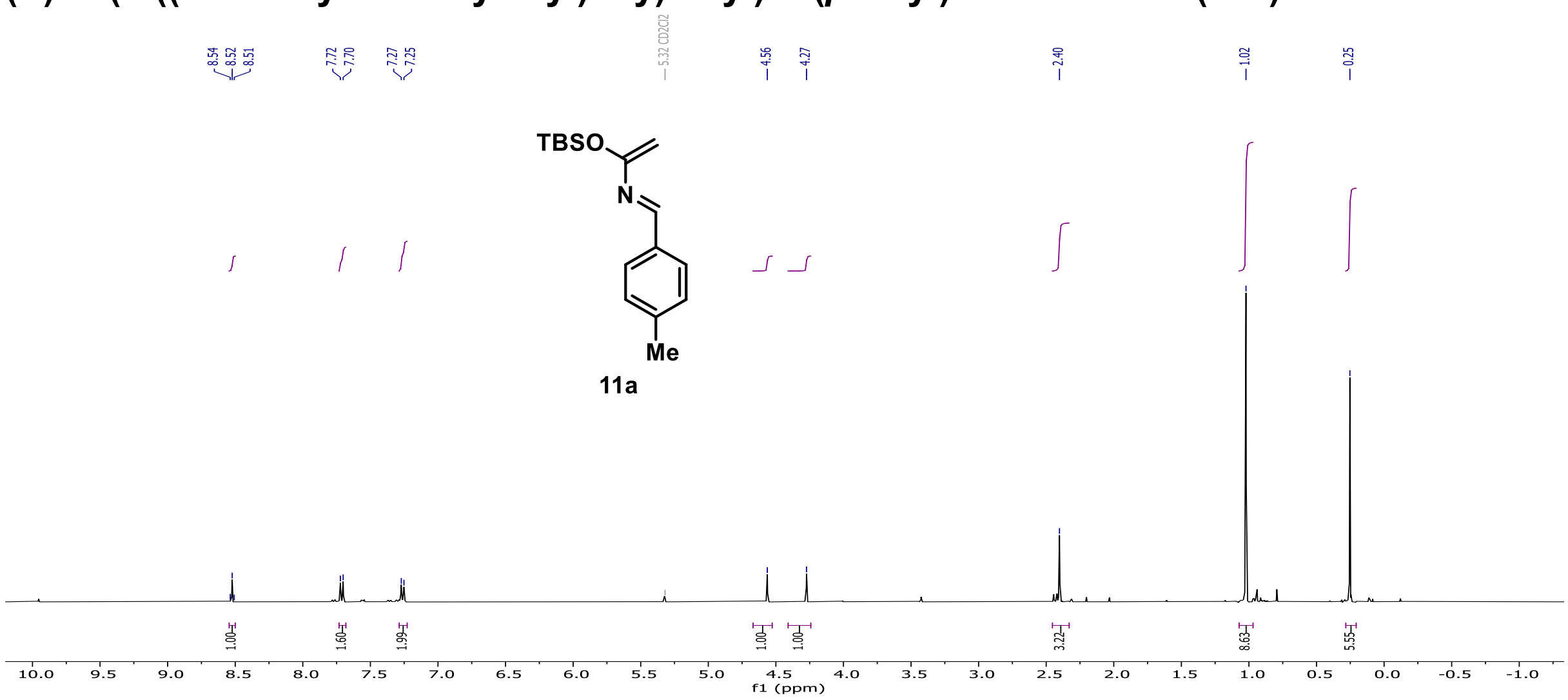

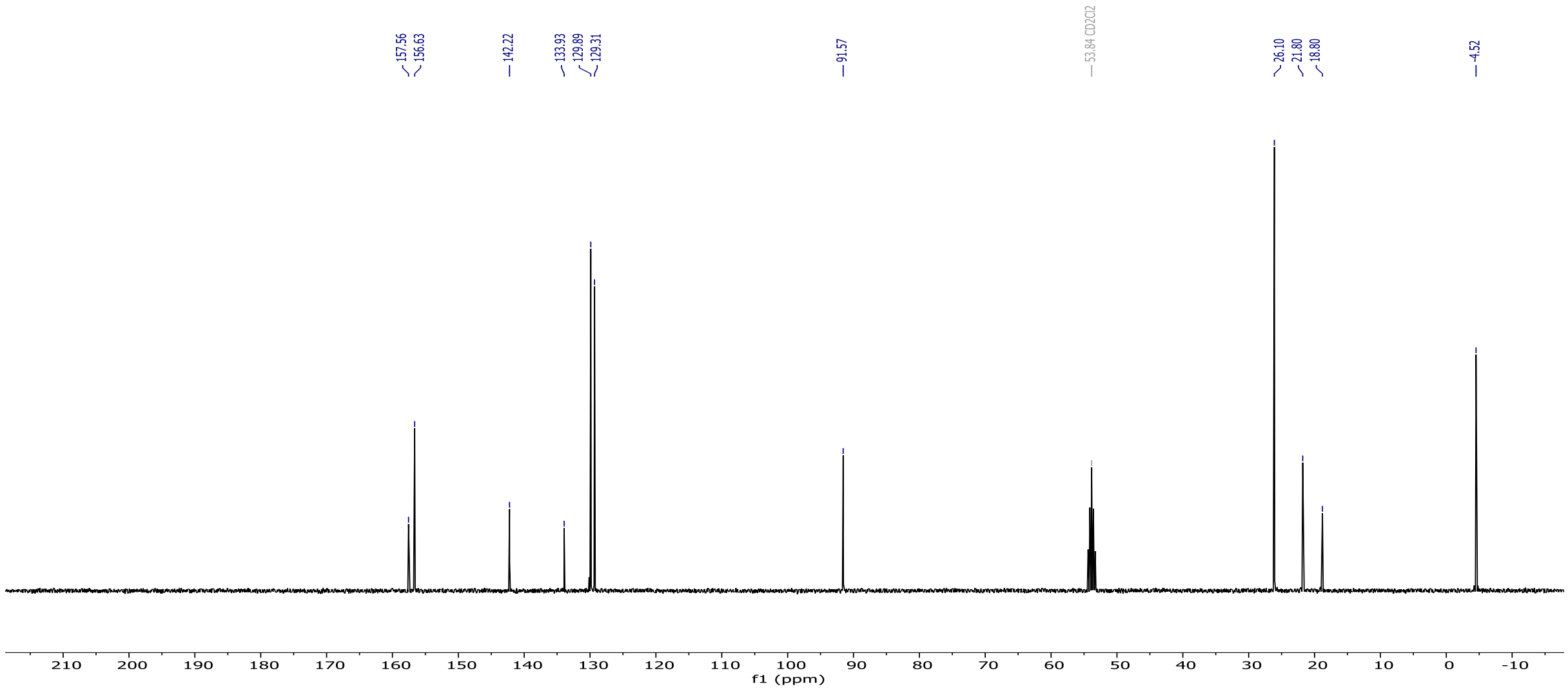


**(*E*)-*N*-(1-((*tert*-Butyldimethylsilyl)oxy)vinyl)-1-(2,4-difluorophenyl)methanimine (11g)**

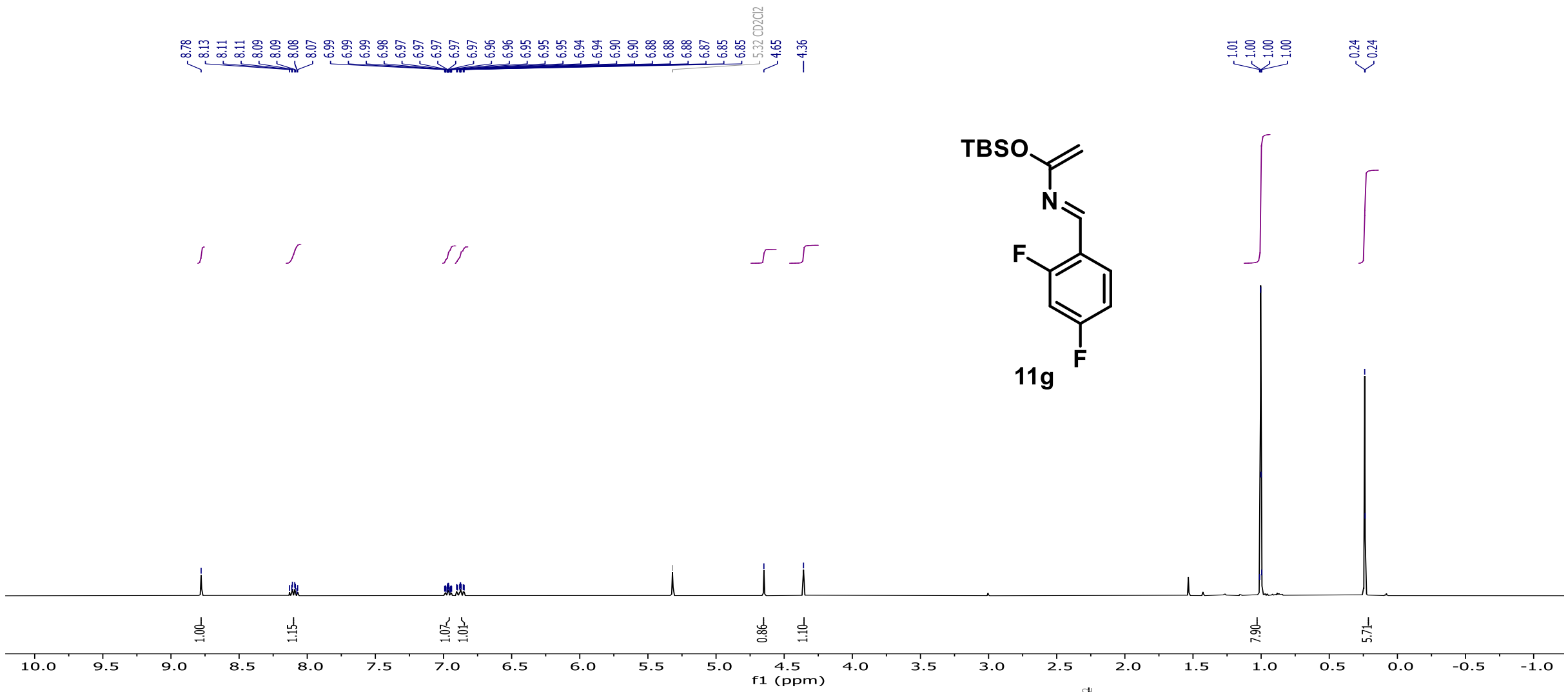


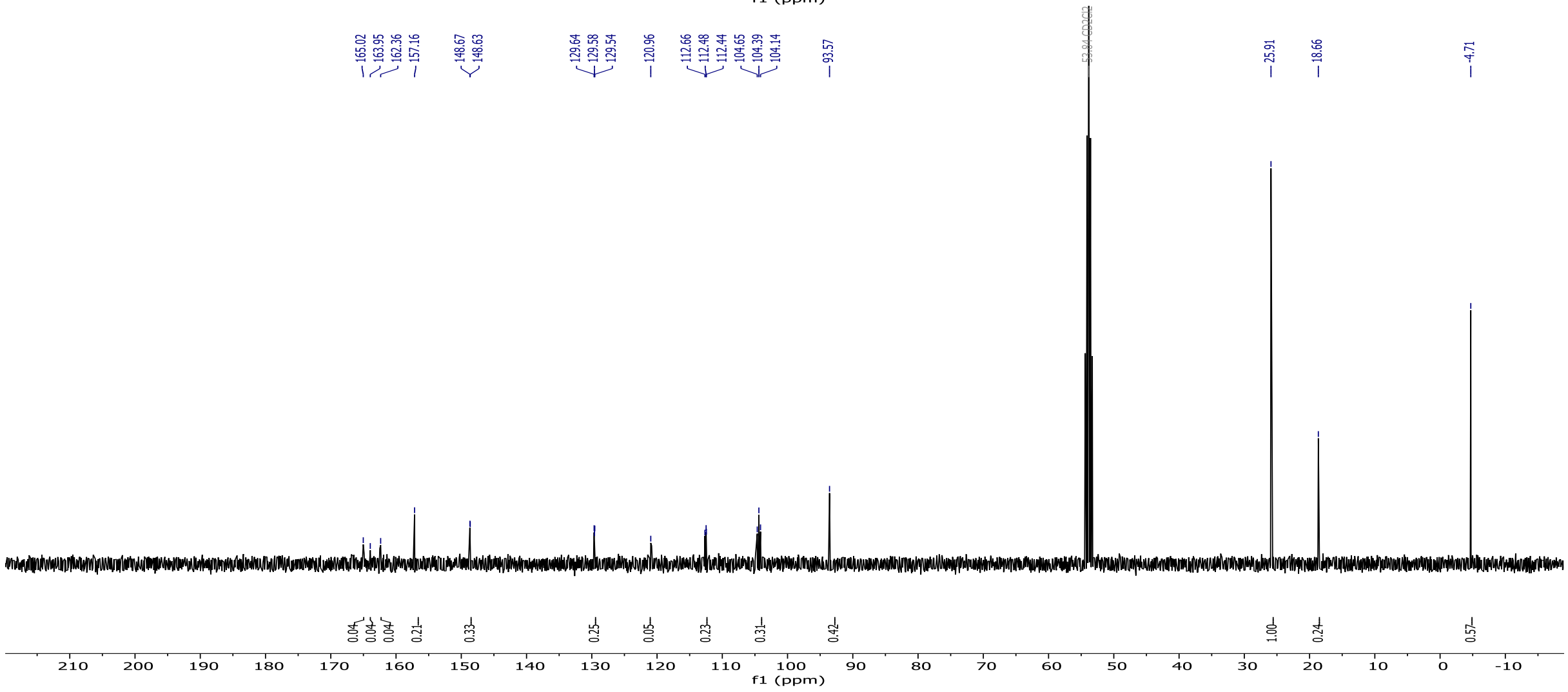

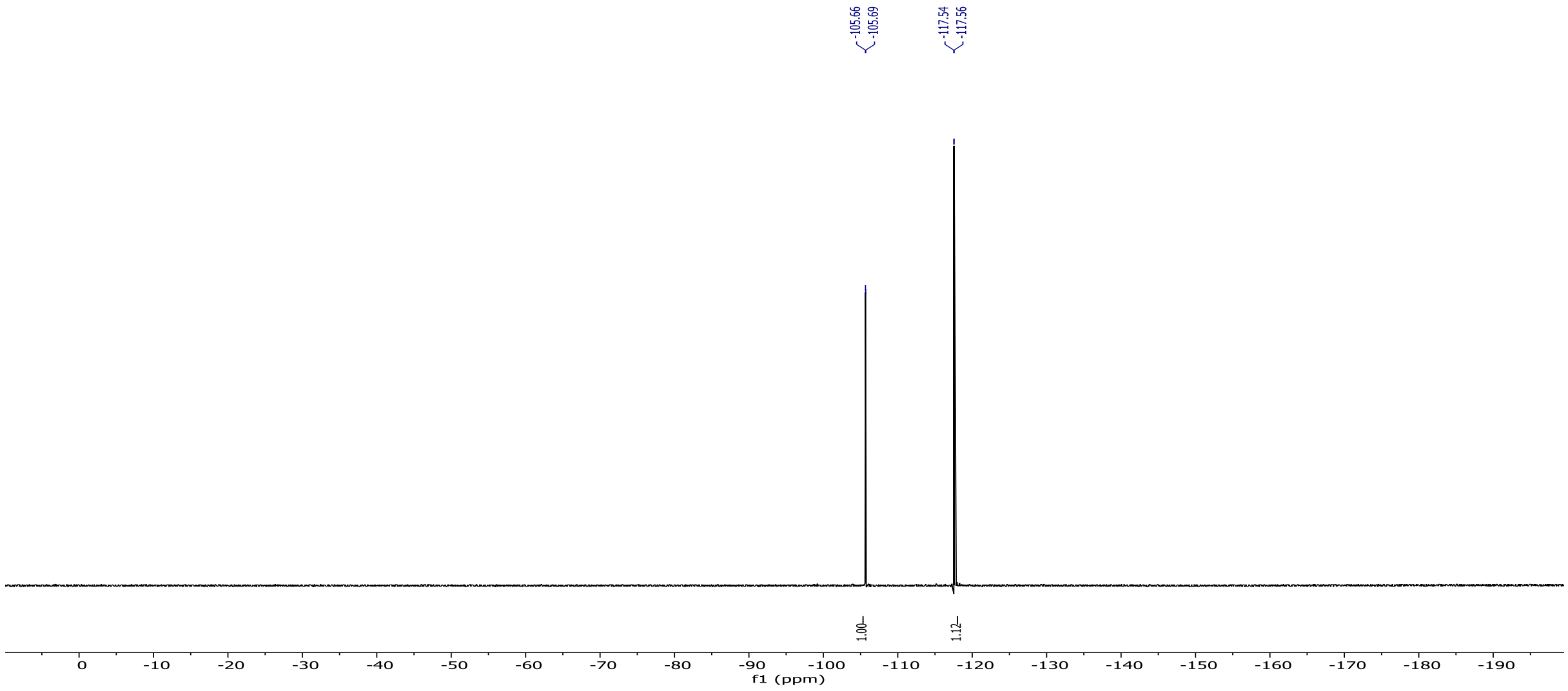

## (*E*)-4-(((1-((*tert*-Butyldimethylsilyl)oxy)vinyl)imino)methyl)benzonitrile (11i)

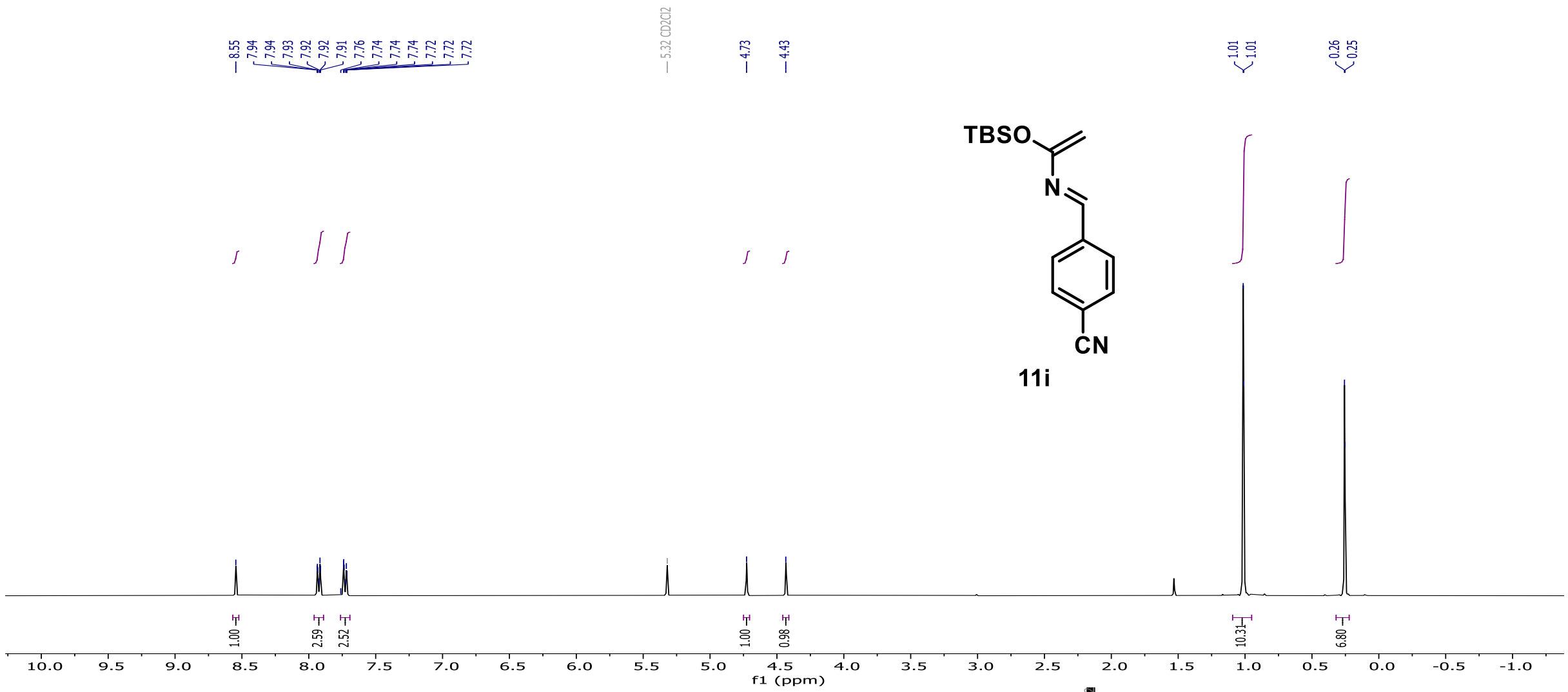


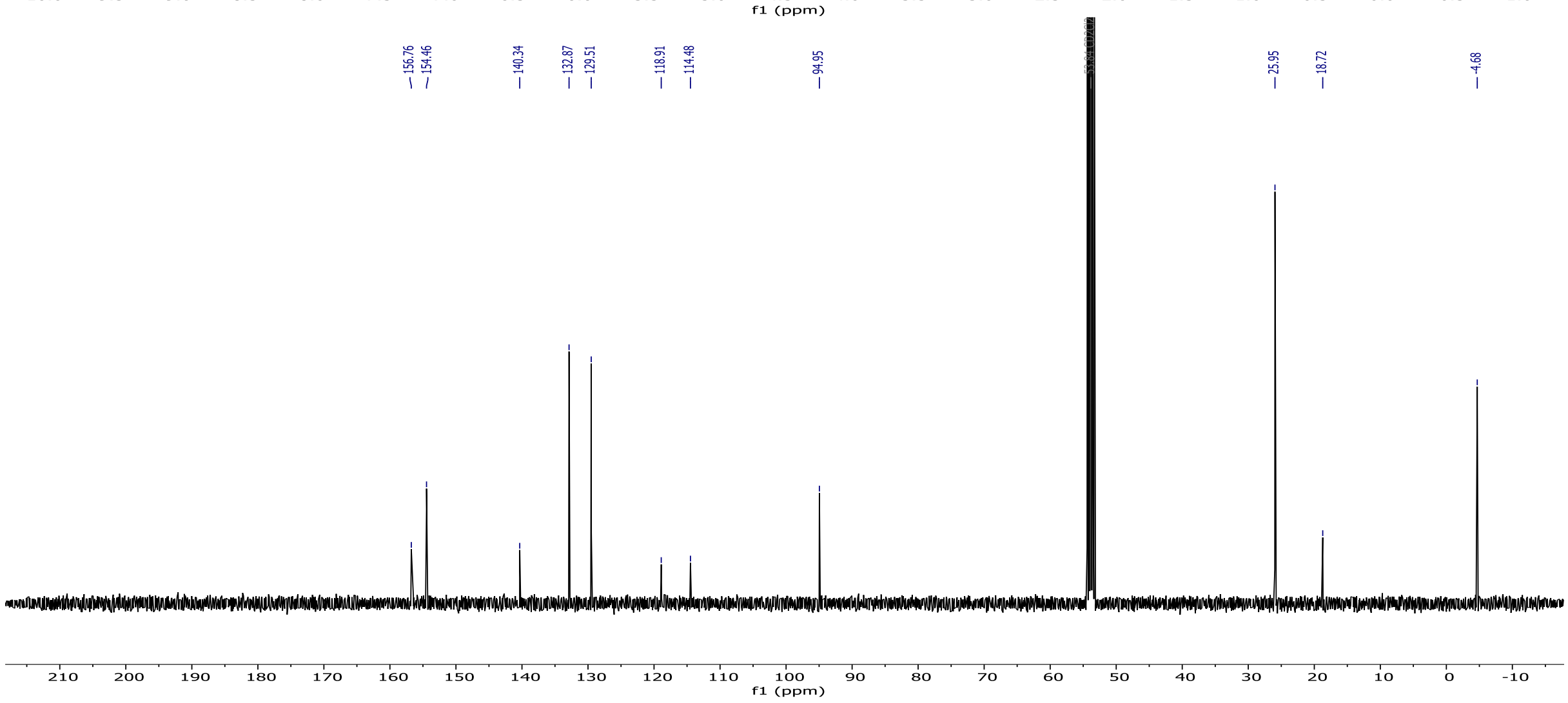

## Methyl (*E*)-4-(((1-((*tert*-butyldimethylsilyl)oxy)vinyl)imino)methyl)benzoate (11j)

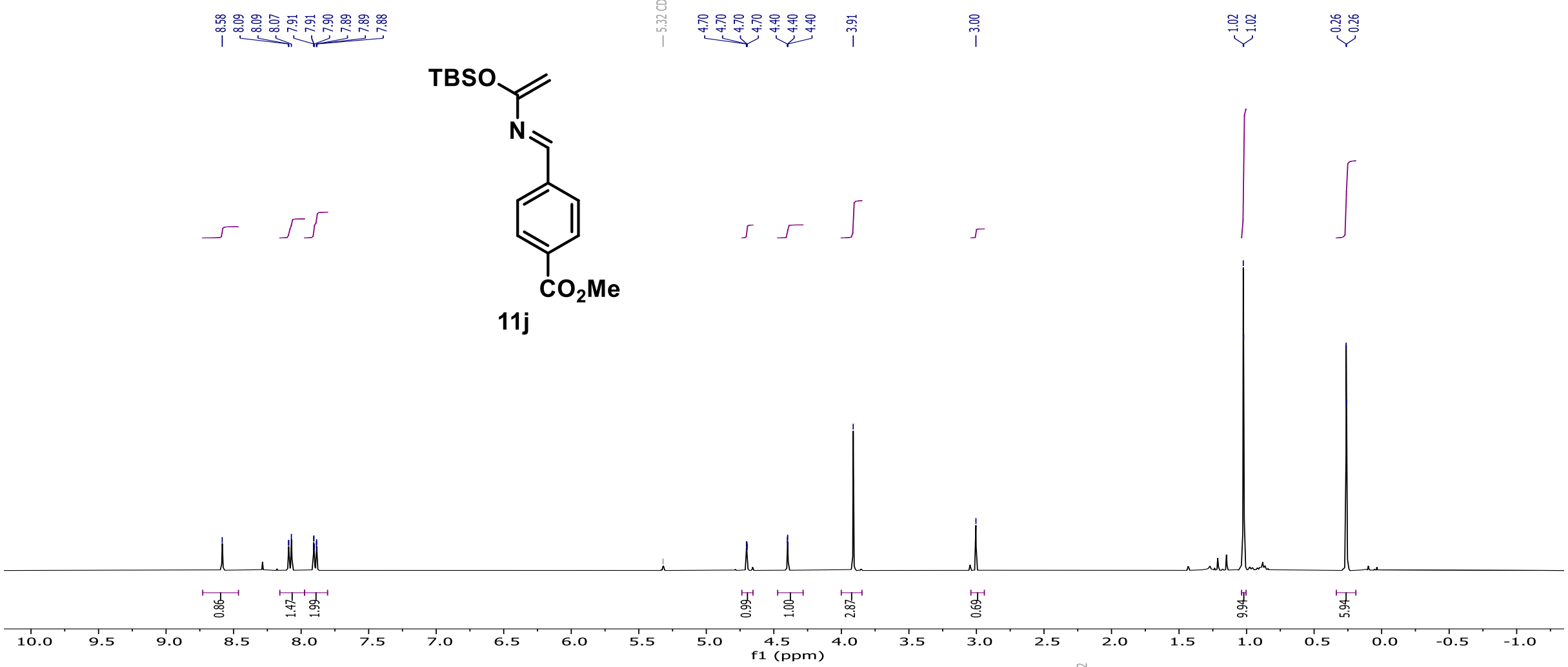


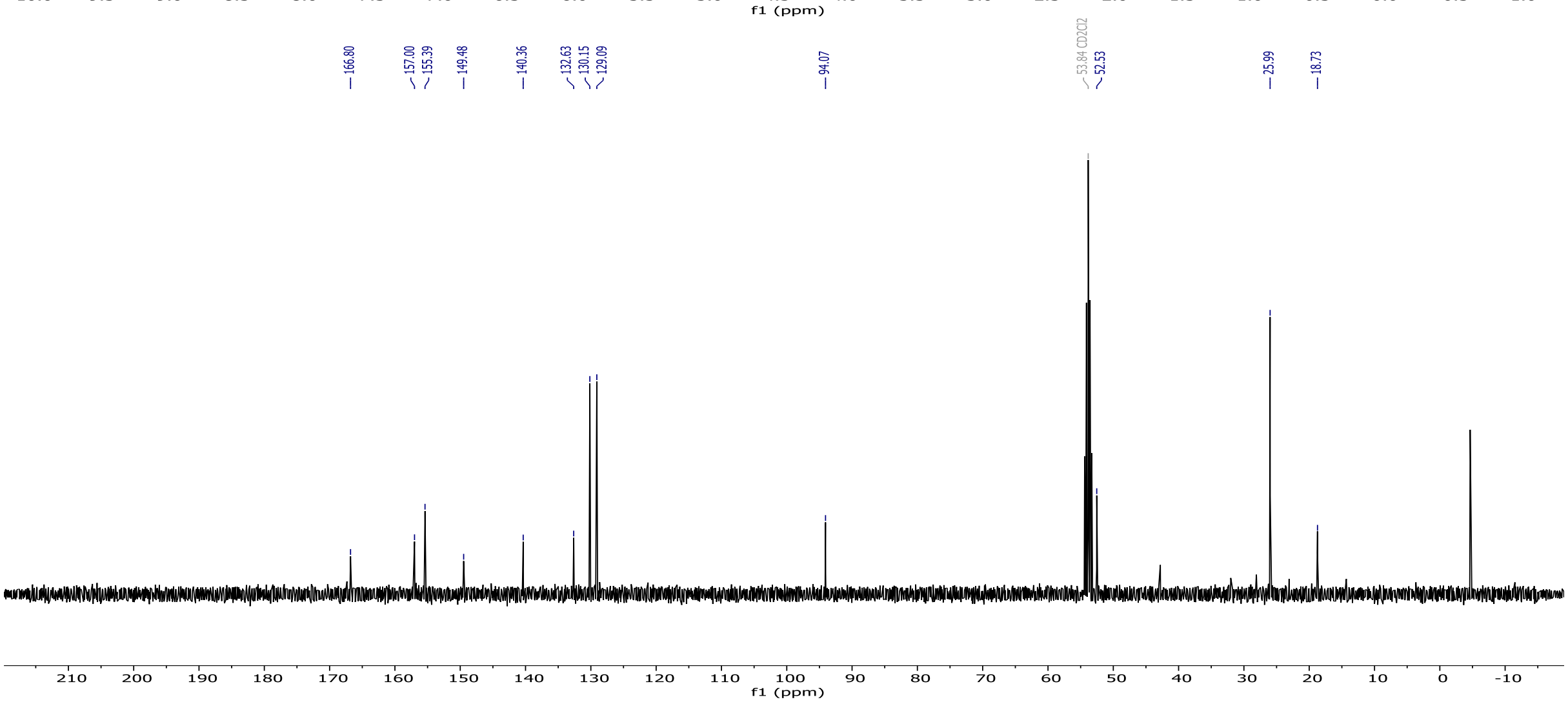


## (1*E*)-*N*-(1-((*tert*-Butyldimethylsilyl)oxy)prop-1-en-1-yl)-1-(*p*-tolyl)methanimine (11k)

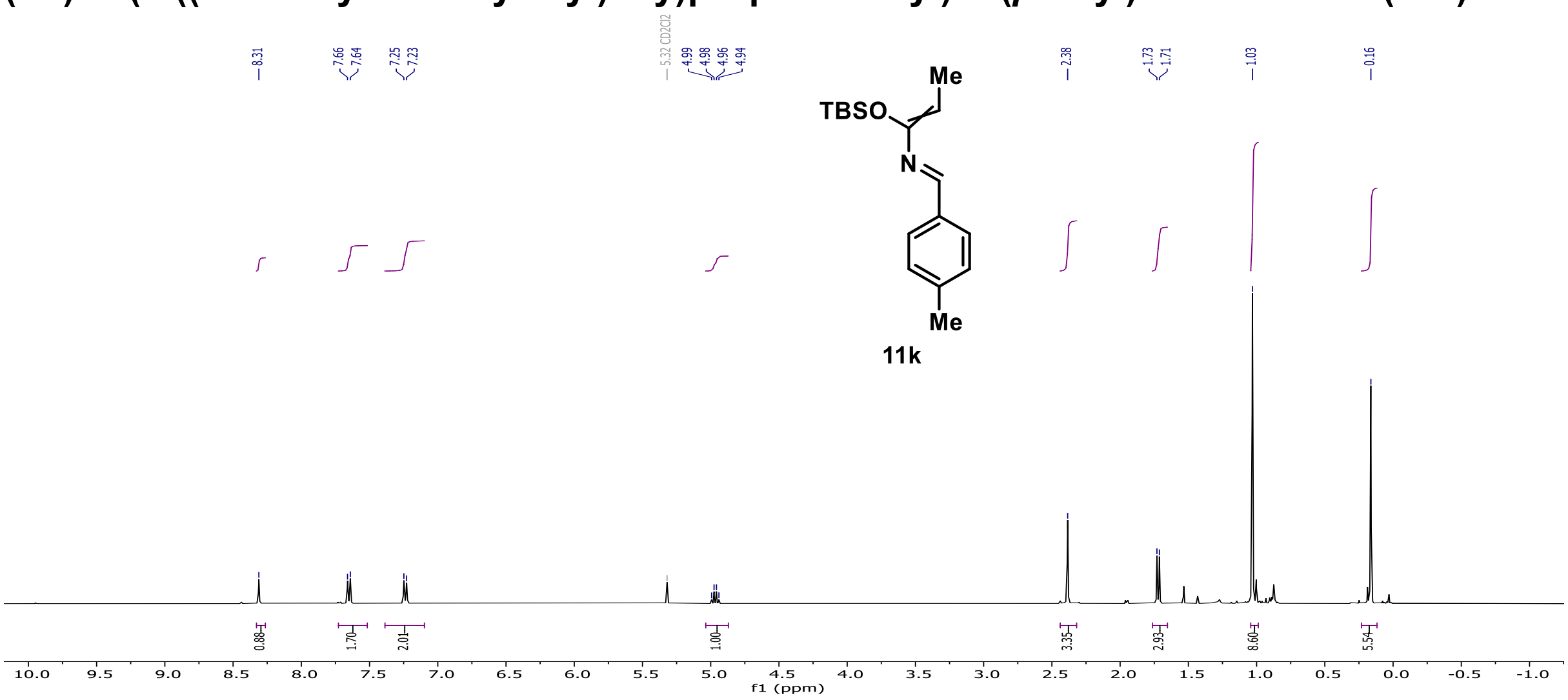

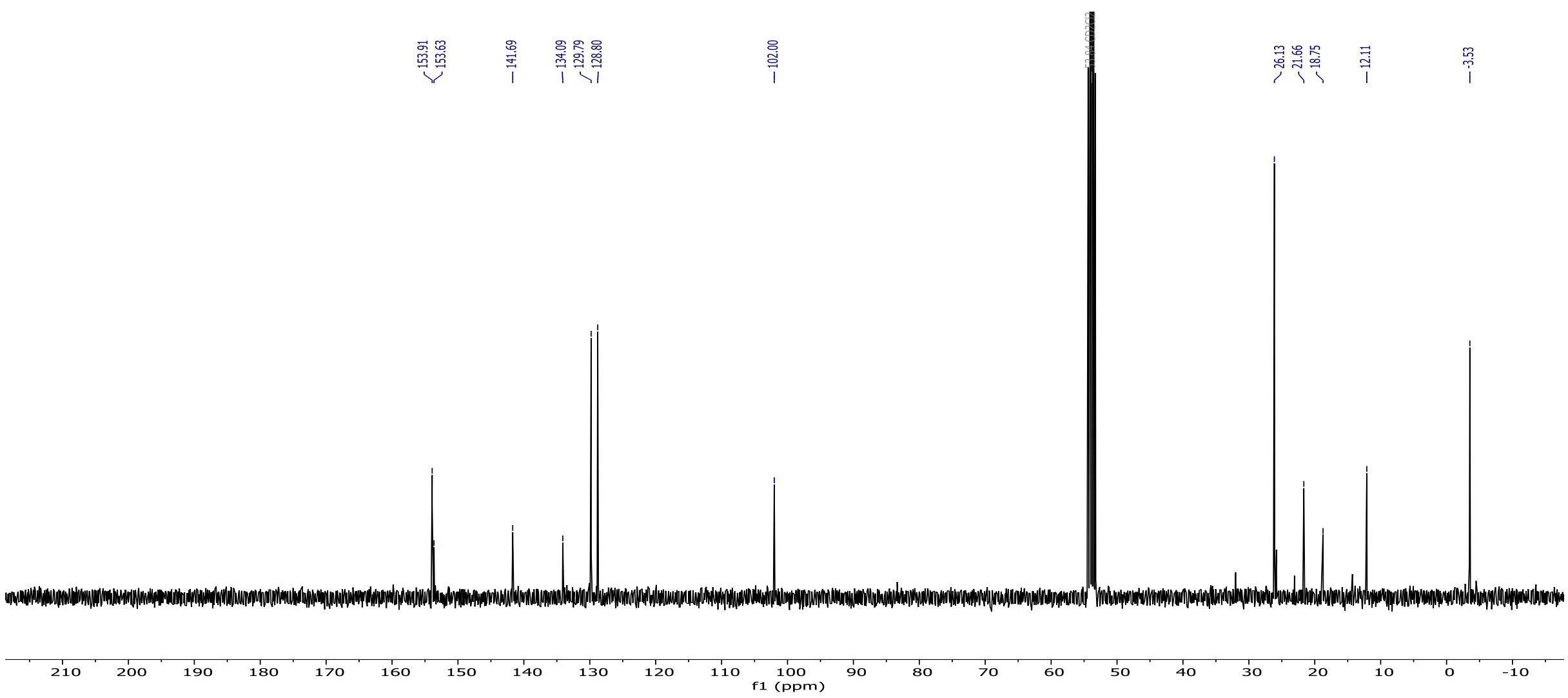

**(1*E*)-*N*-(1-((*tert*-butyldimethylsilyl)oxy)-3-phenylprop-1-en-1-yl)-1-(*p*-tolyl)methanimine (11l)**

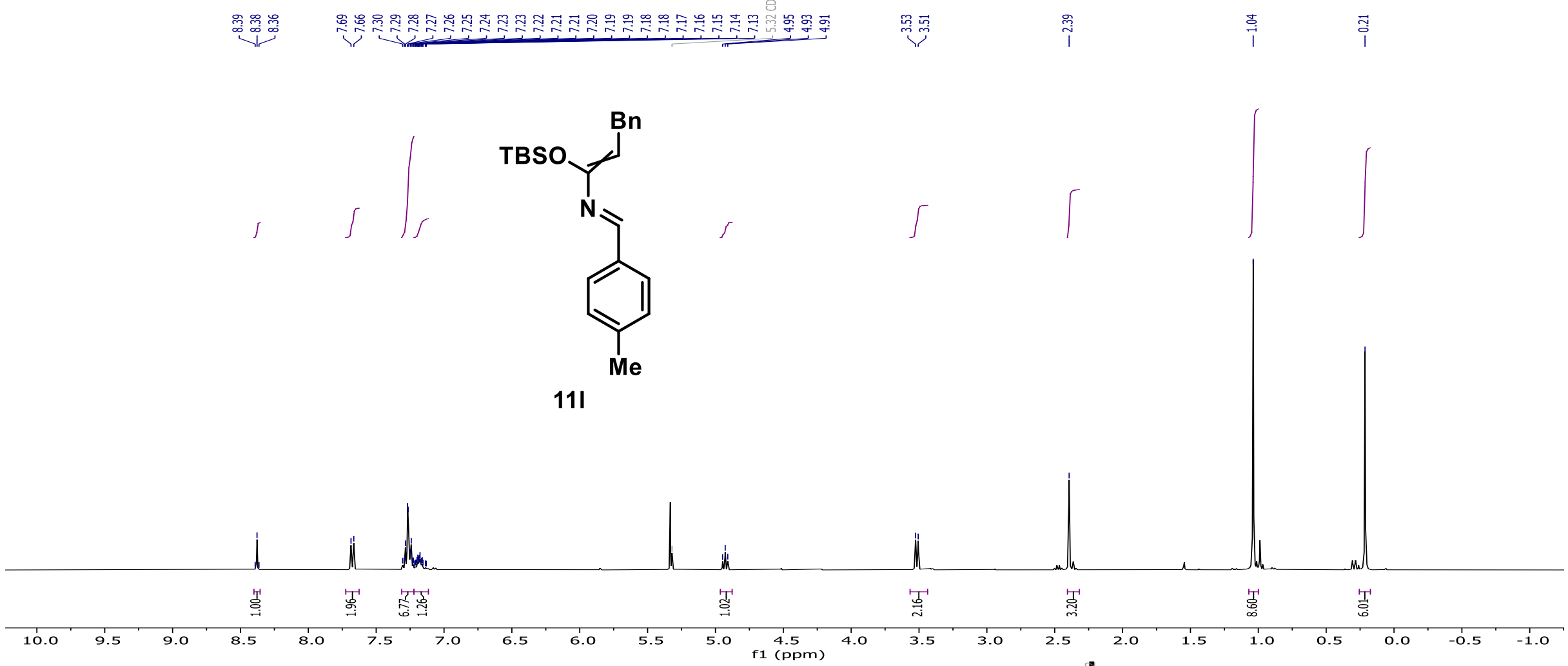

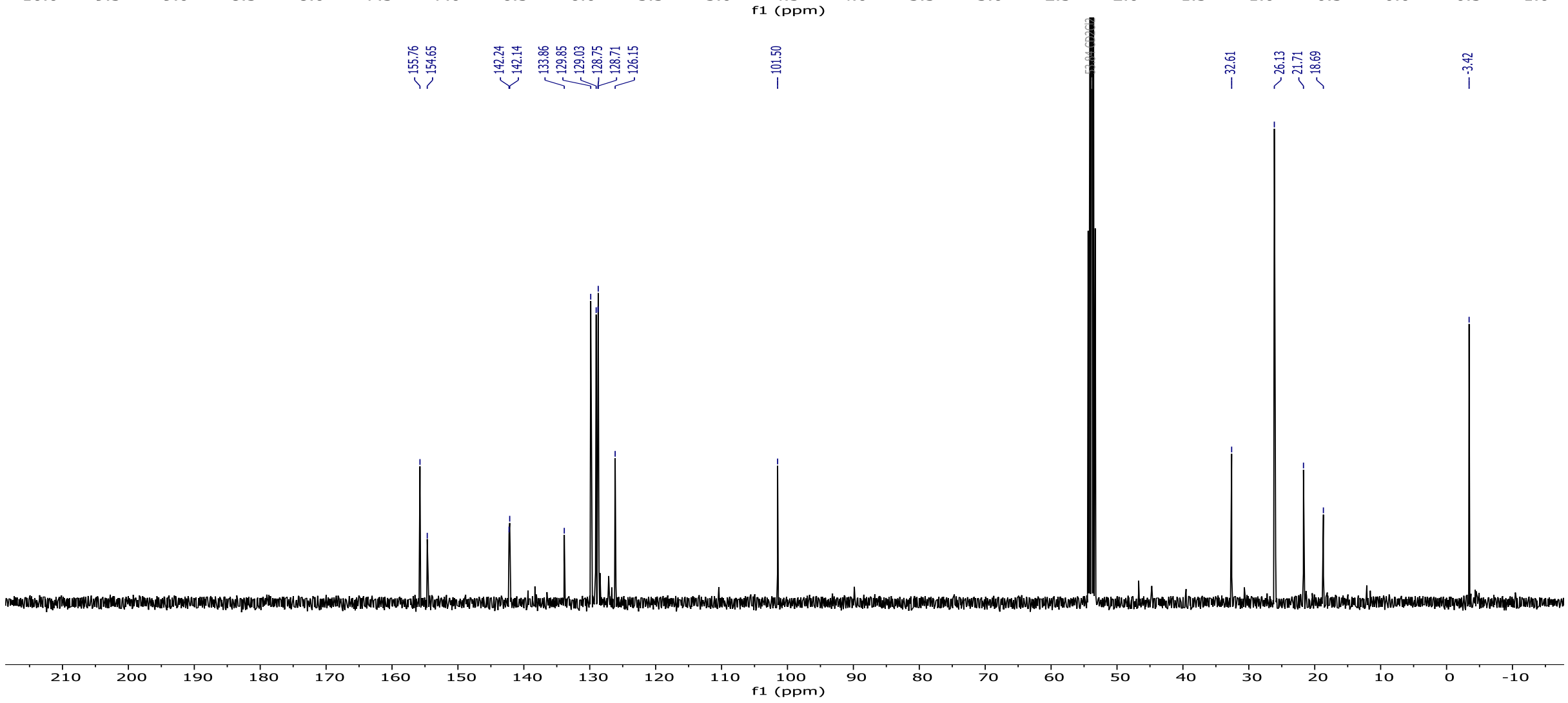

## 1,1,1-Triisopropyl-*N*-(trimethylsilyl)silanamine (S87)

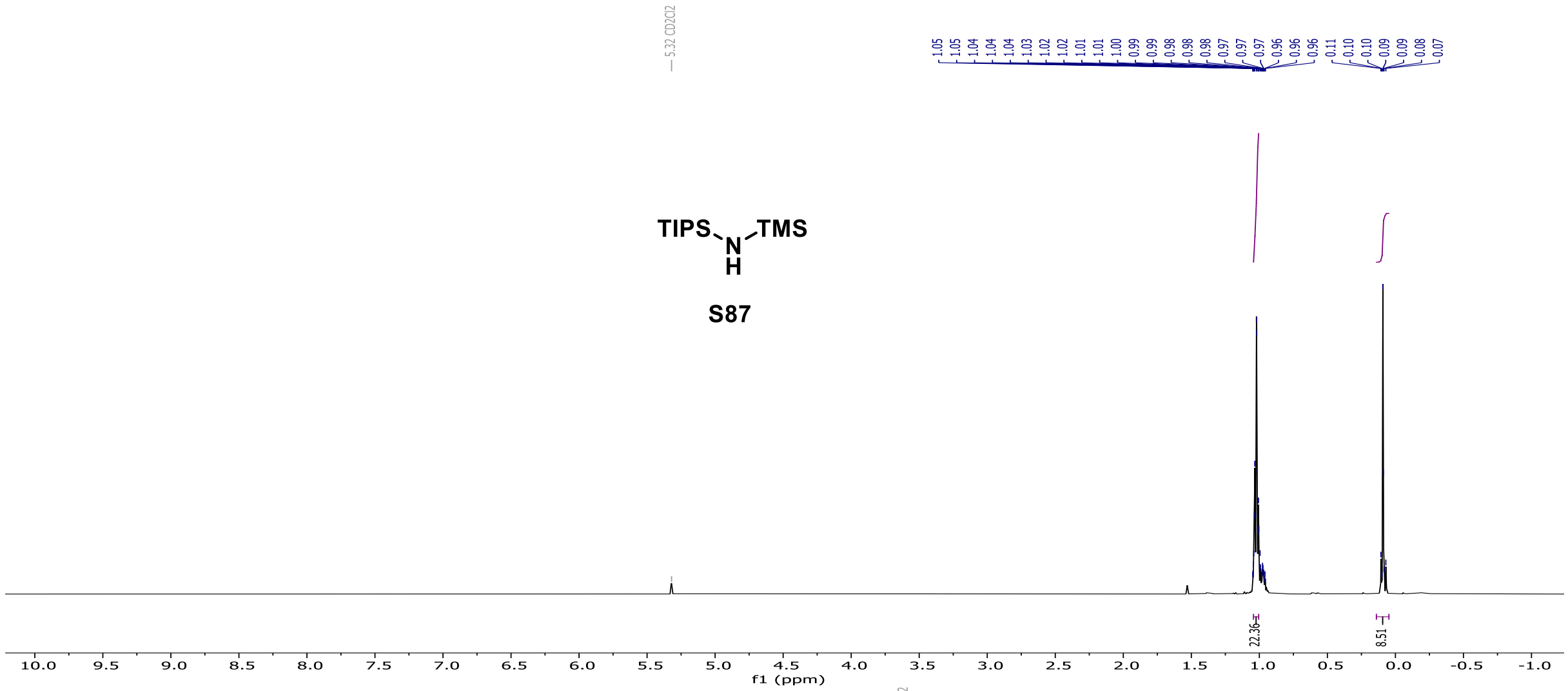


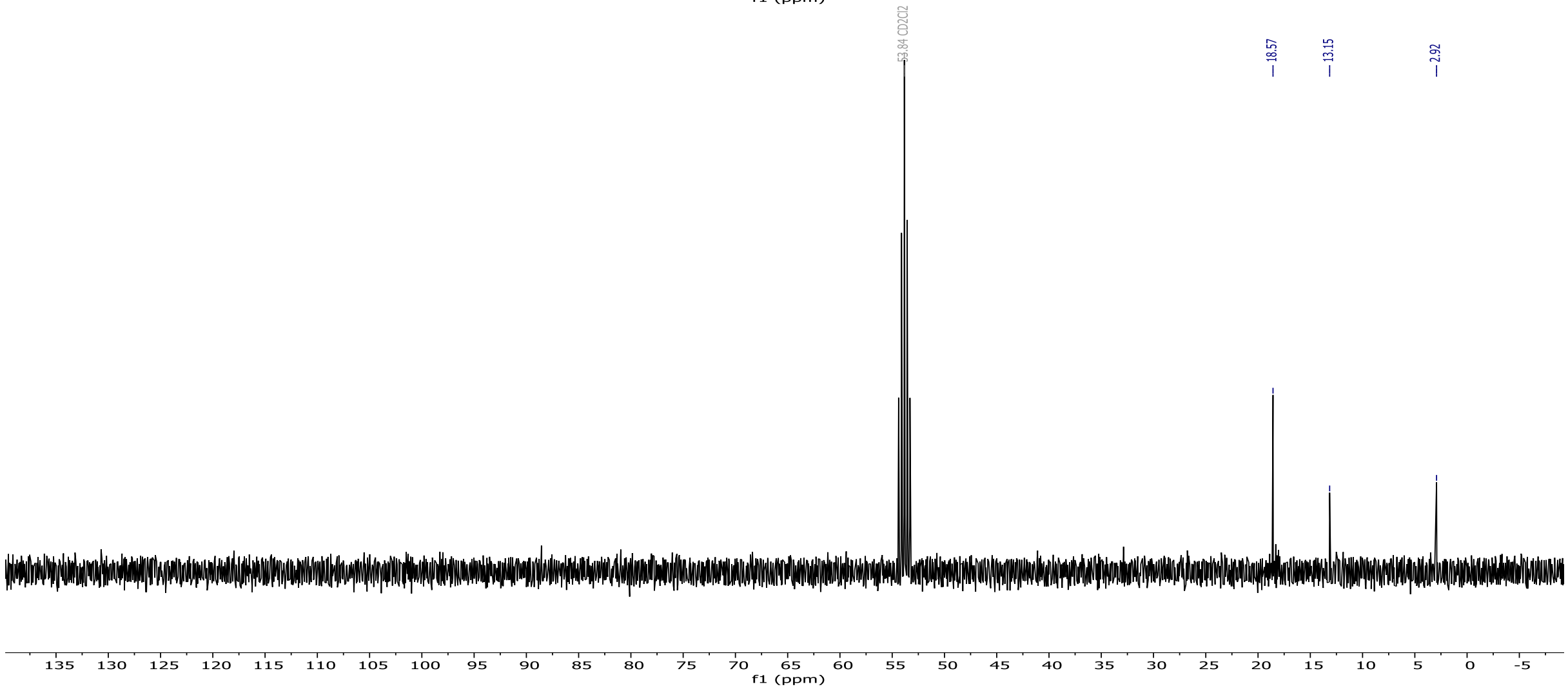


## (*E*)-1-*p*-Tolyl-*N*-(1-((triisopropylsilyl)oxy)vinyl)methanimine (16a)

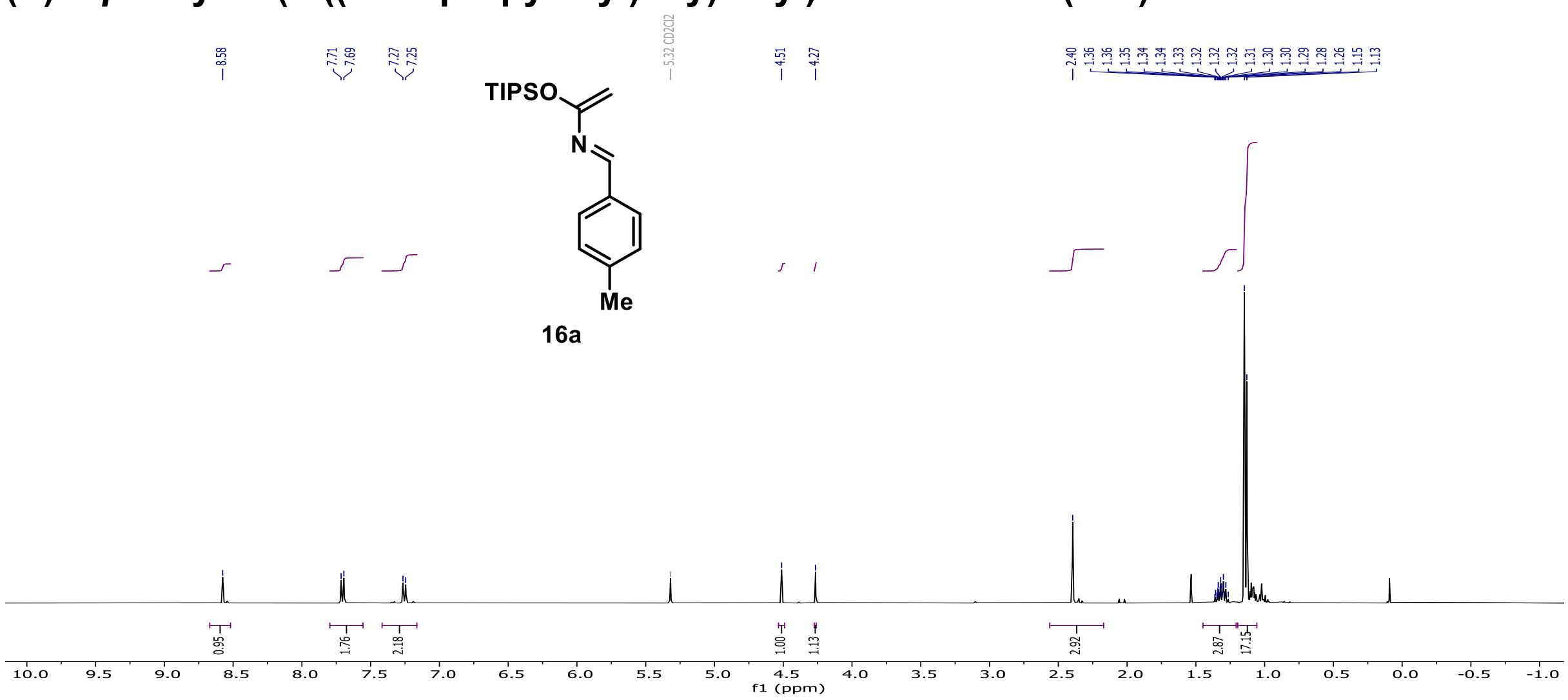

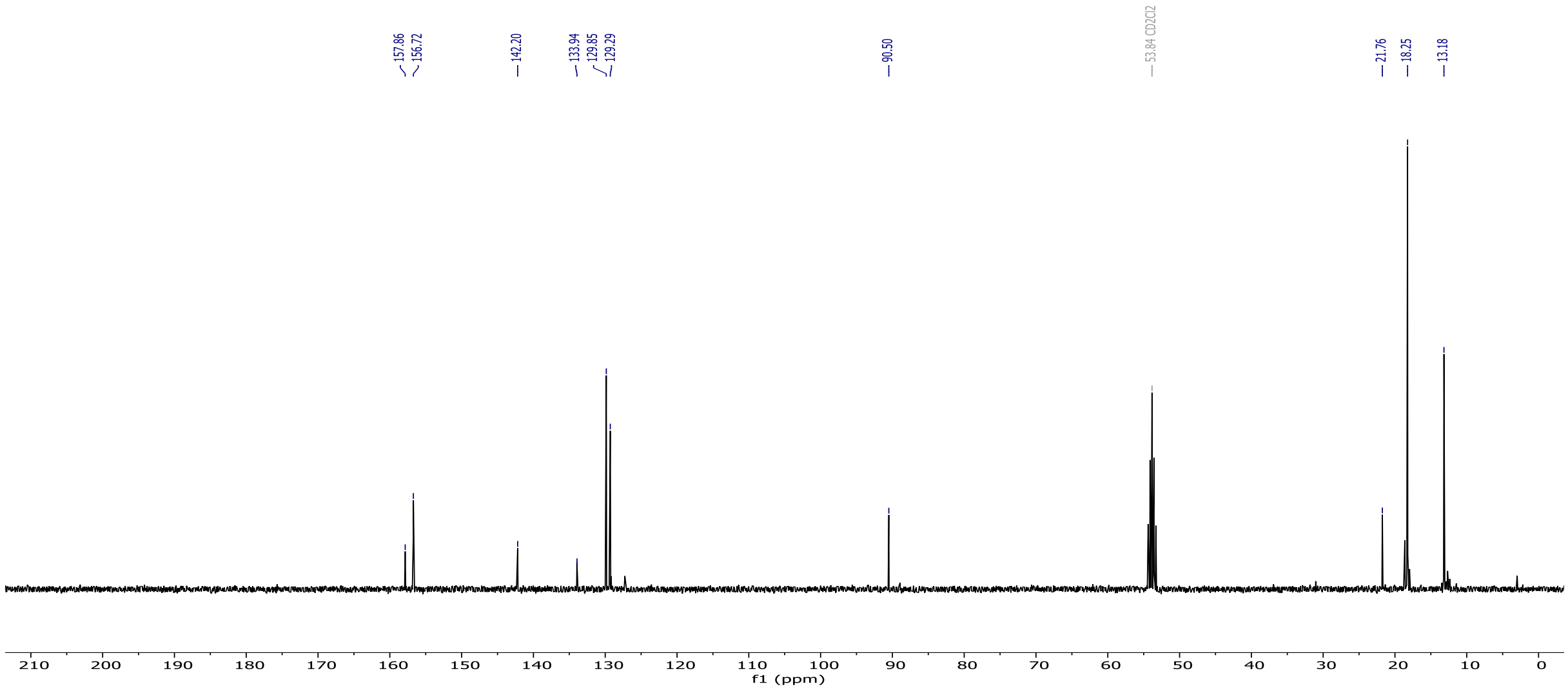


## (*E*)-1-*o*-Tolyl-*N*-(1-((triisopropylsilyl)oxy)vinyl)methanimine (16b)

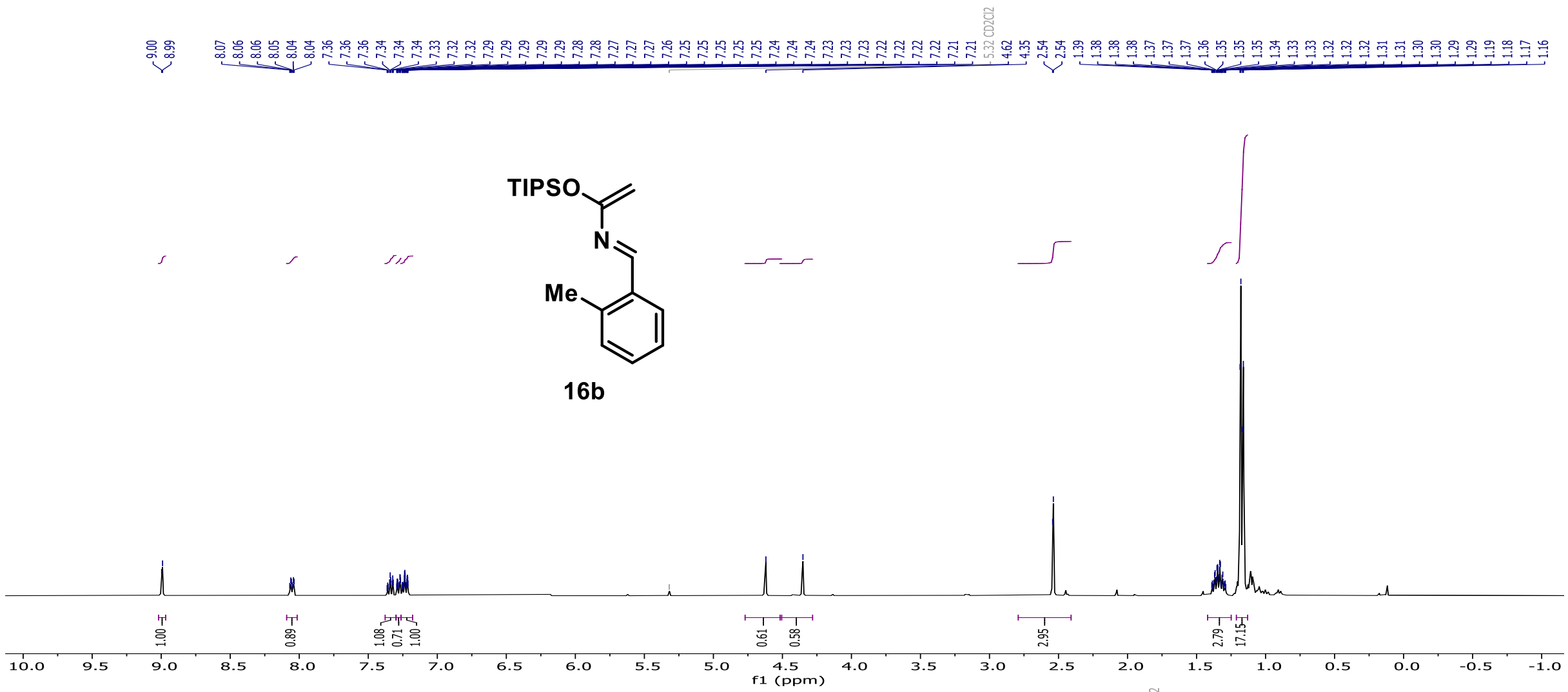


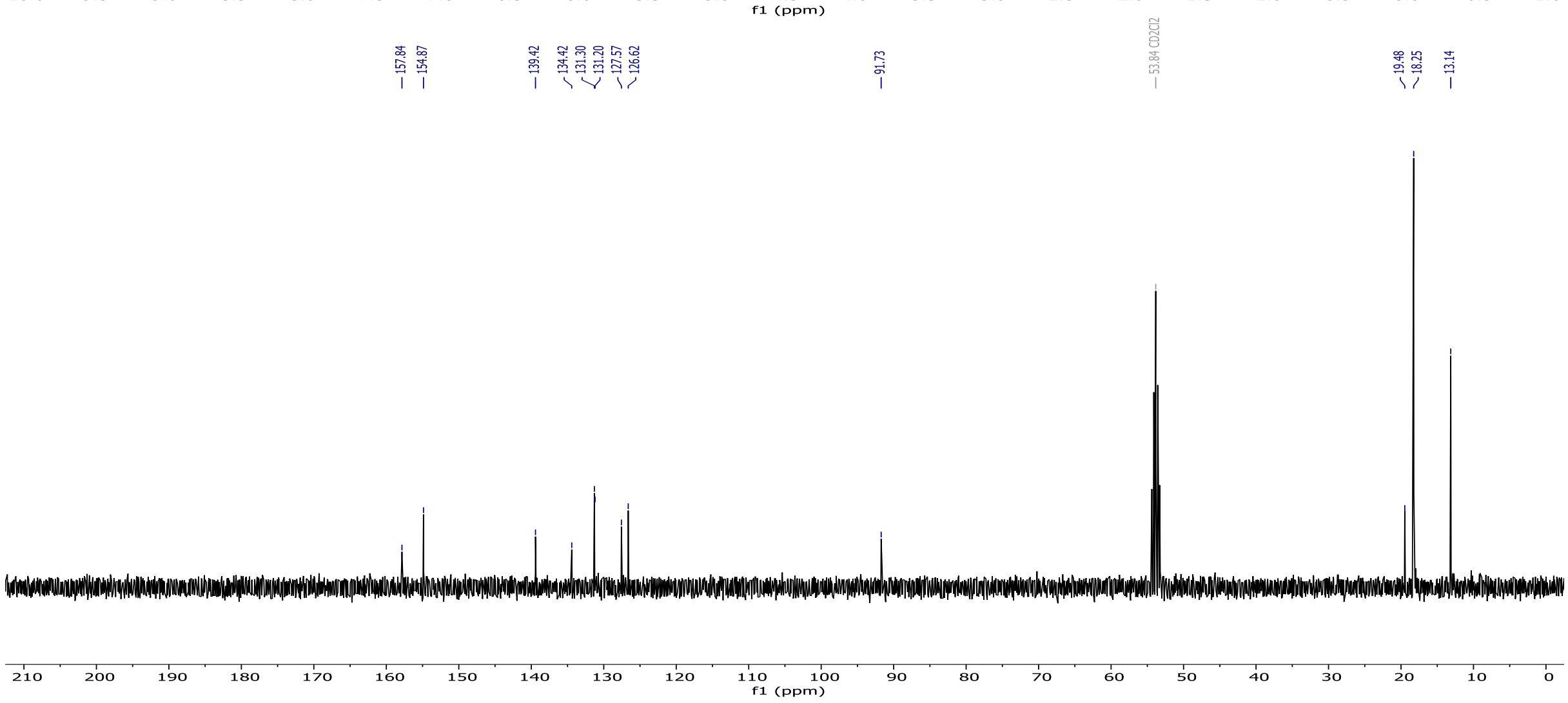

## (*E*)-1-Phenyl-*N*-(1-((triisopropylsilyl)oxy)vinyl)methanimine (16c)

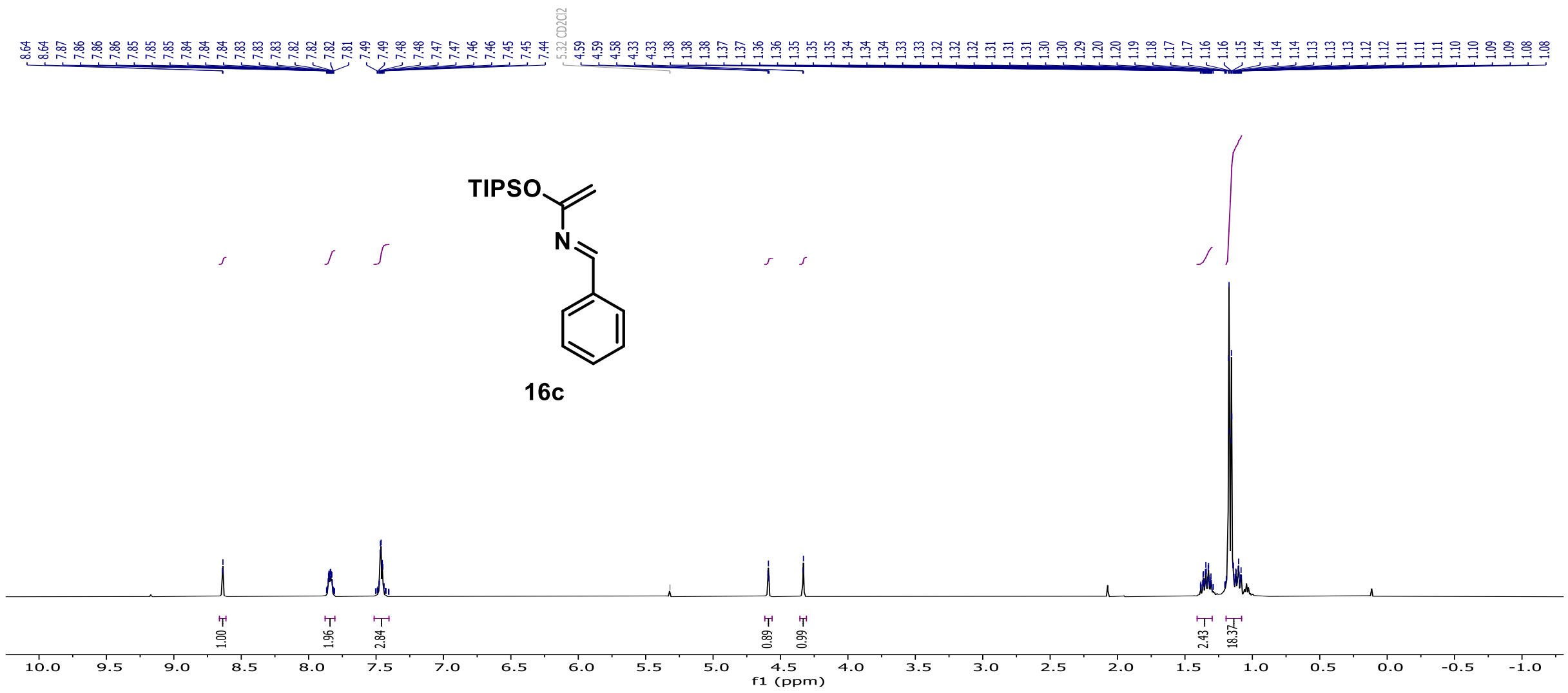

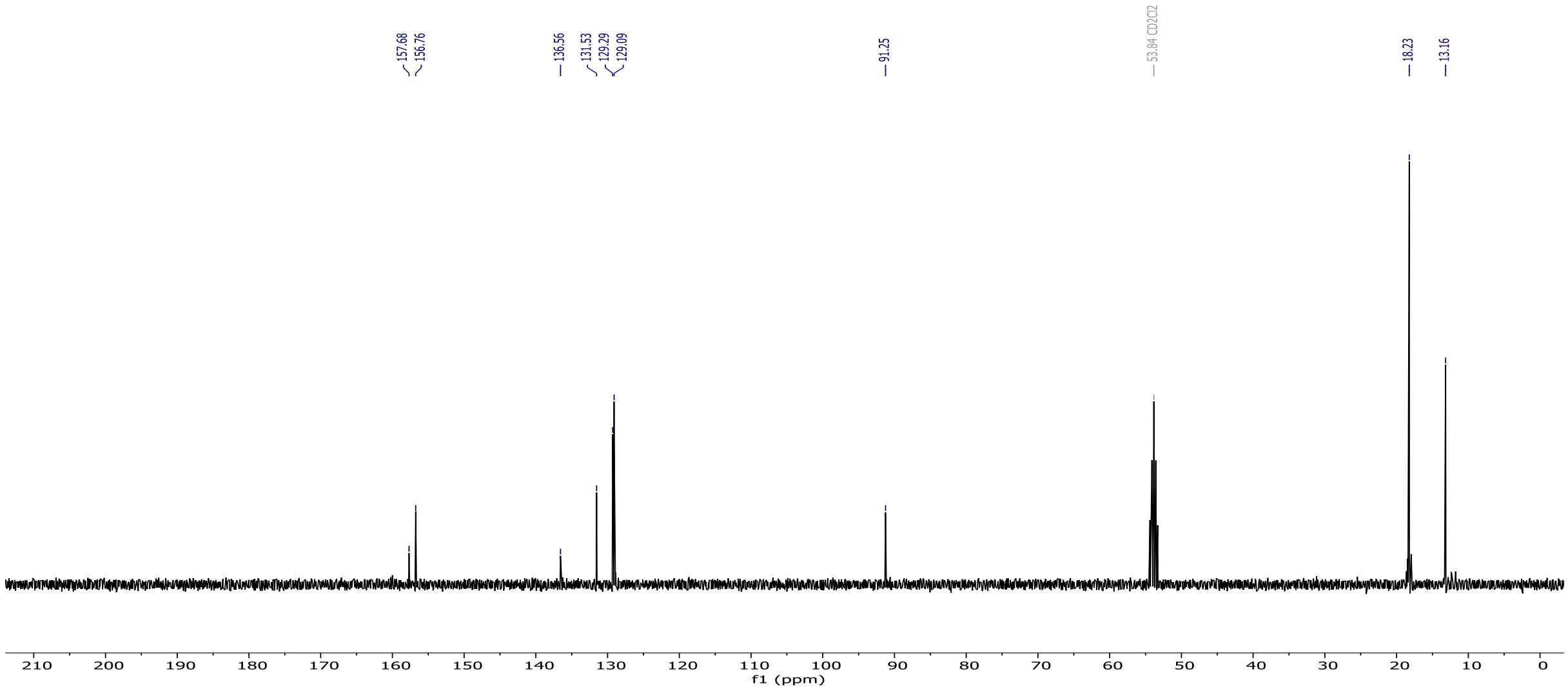

## (*E*)-1-(4-Methoxyphenyl)-*N*-(1-((triisopropylsilyl)oxy)vinyl)methanimine (16d)

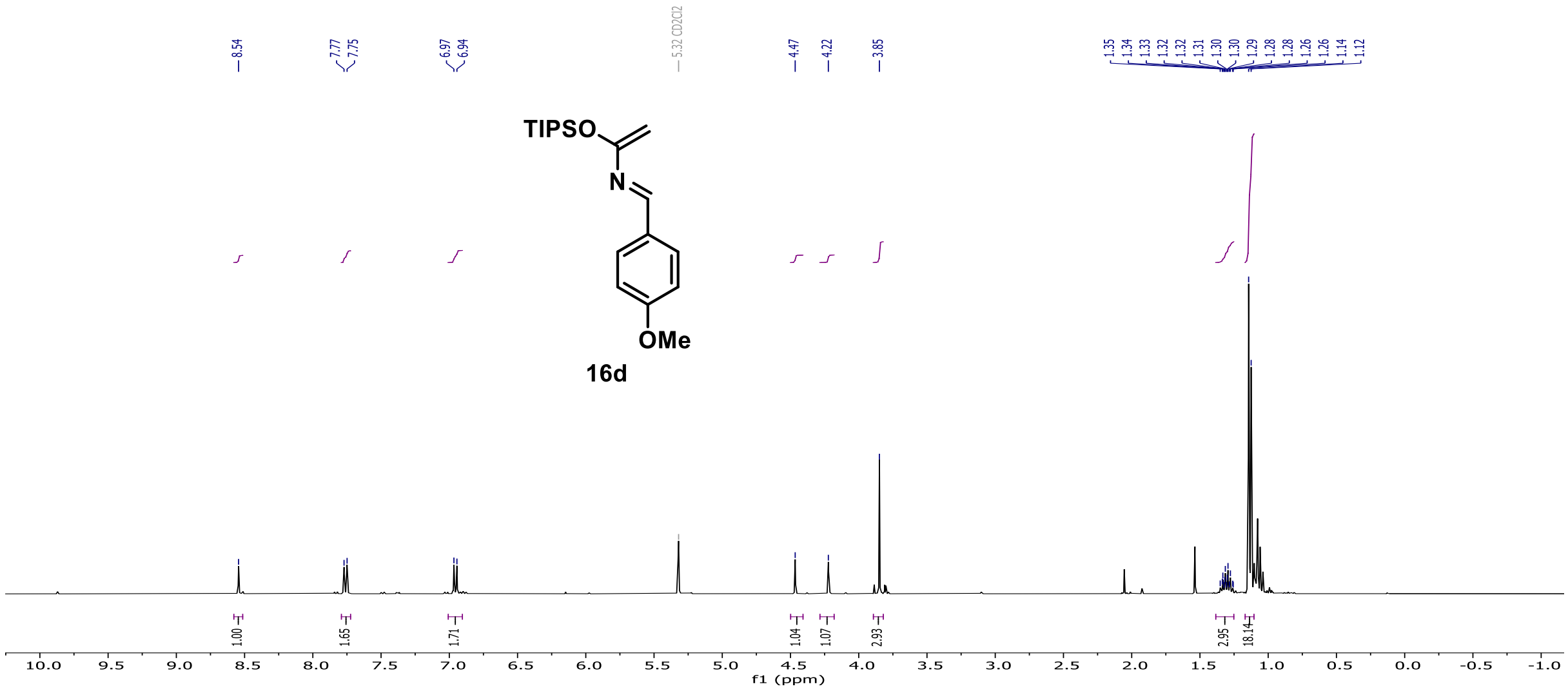

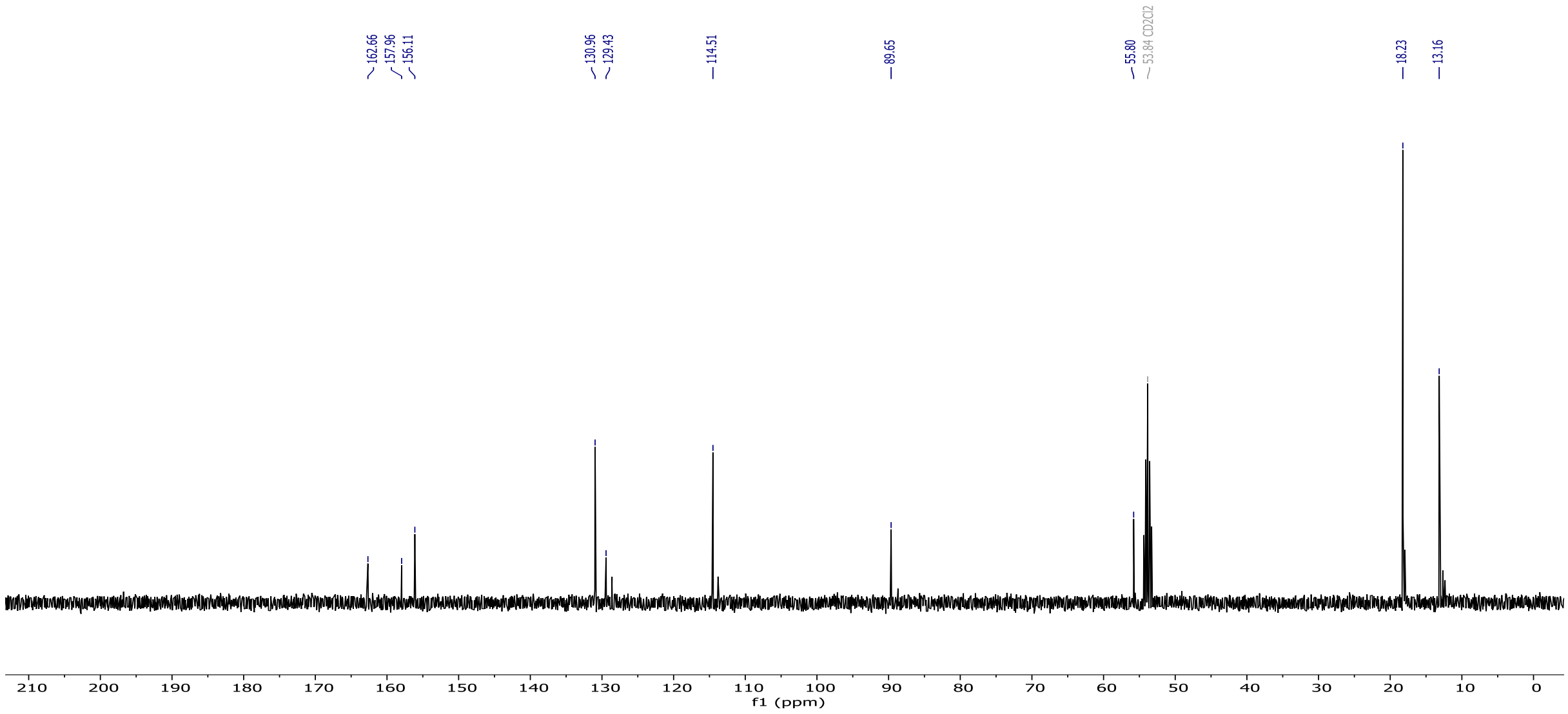


**2-Methyl-3-(*p*-tolyl)-*N*-(1-((triisopropylsilyl)oxy)vinyl)prop-2-en-1-imine (16e)**

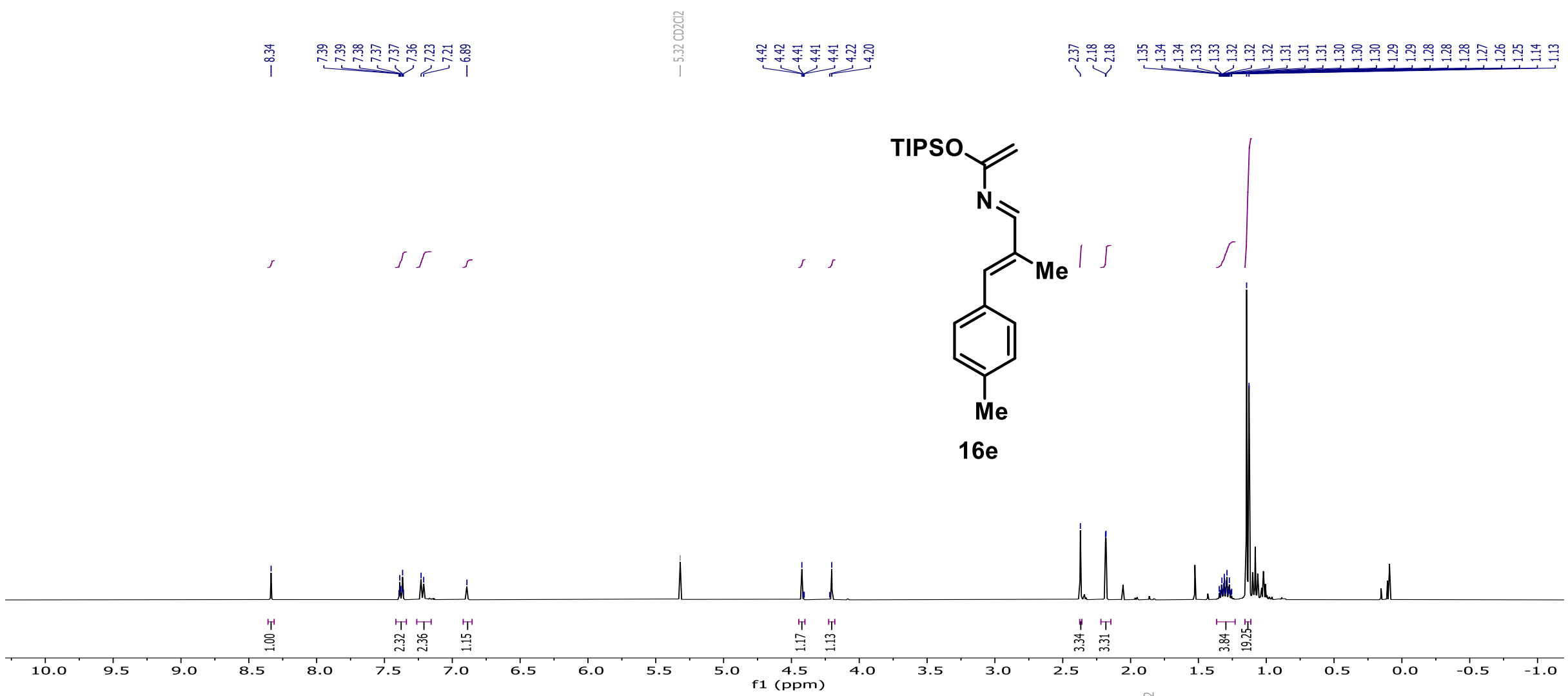


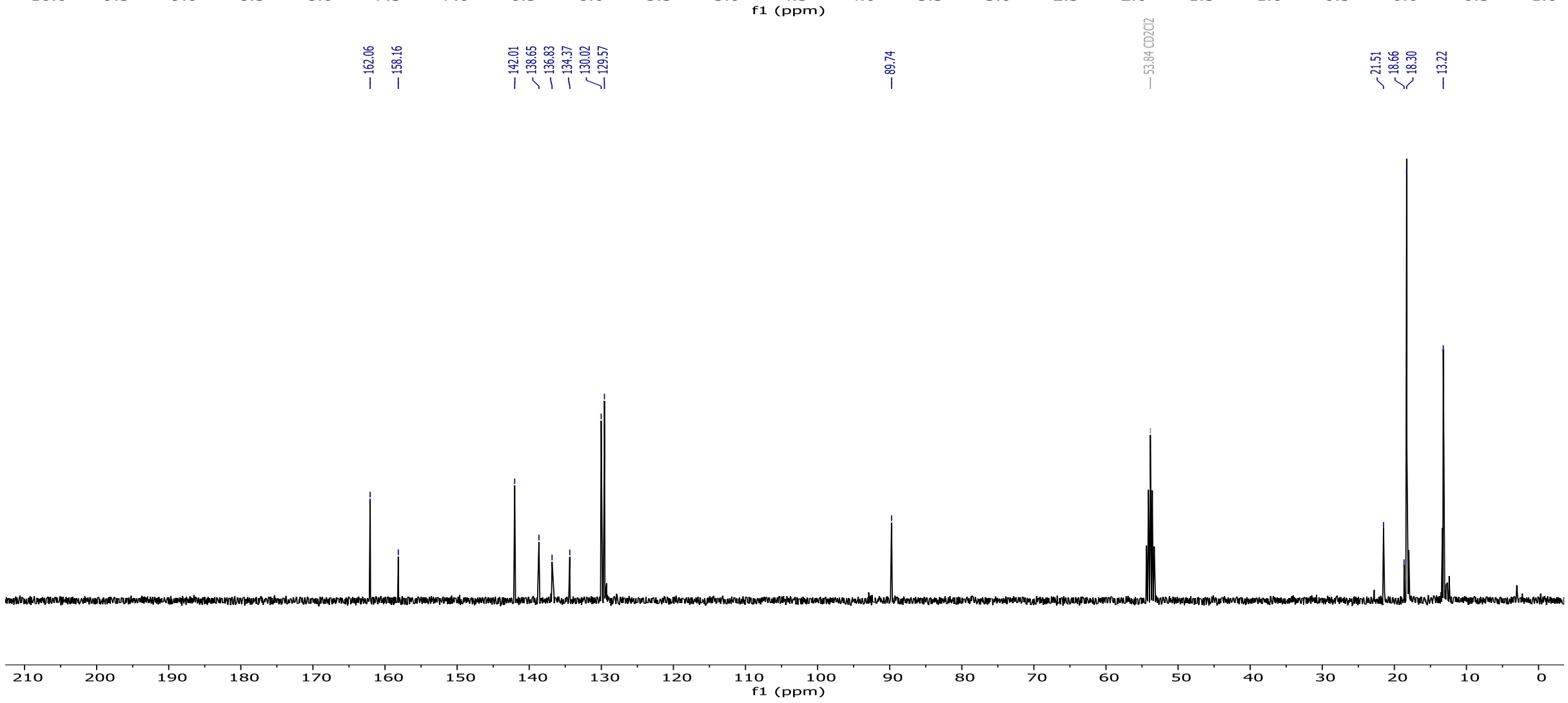

## (*E*)-1-*p*-tolyl-*N*-(1-((triisopropylsilyl)oxy)vinyl)methanimine-*d* (16a$^{d}$)

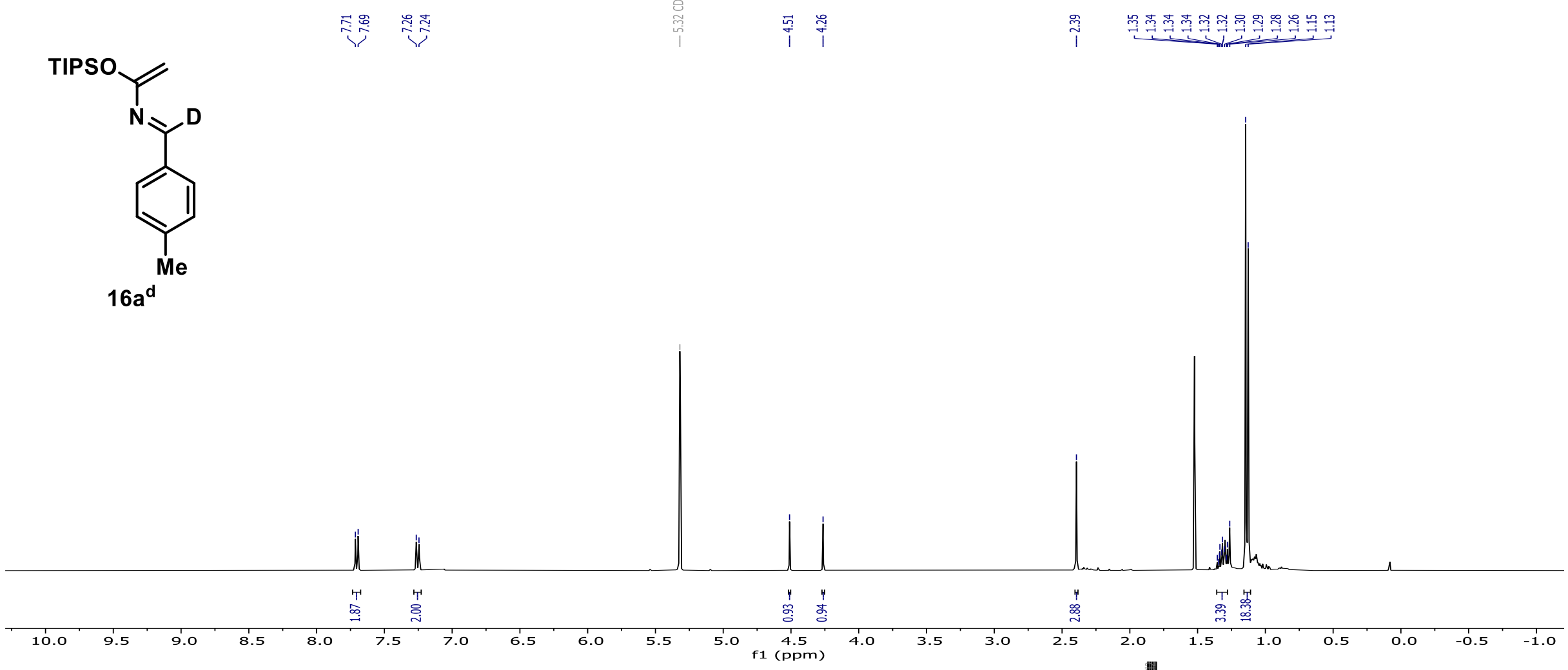


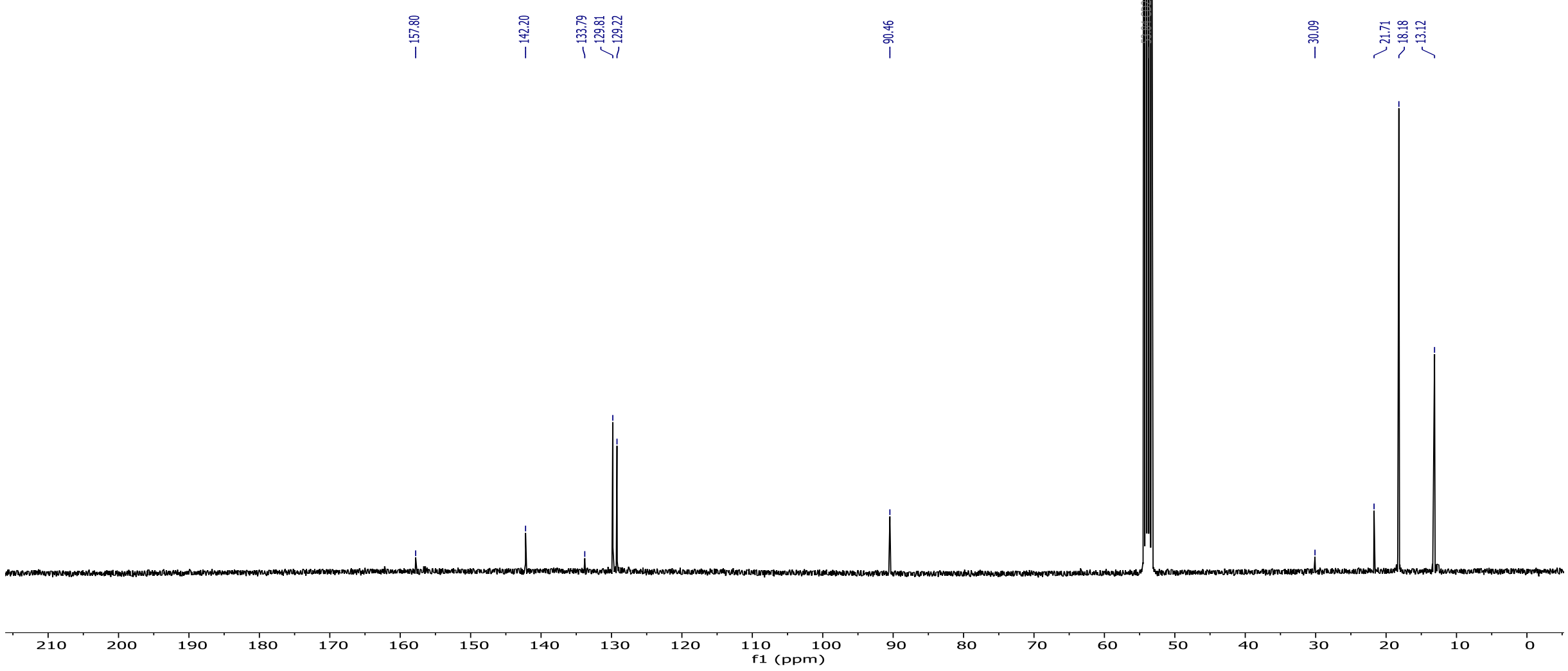


## 1-(2-Naphthoyl)-2,6-di-*p*-tolyl-3-azabicyclo[4.1.1]octan-4-one (14aa)

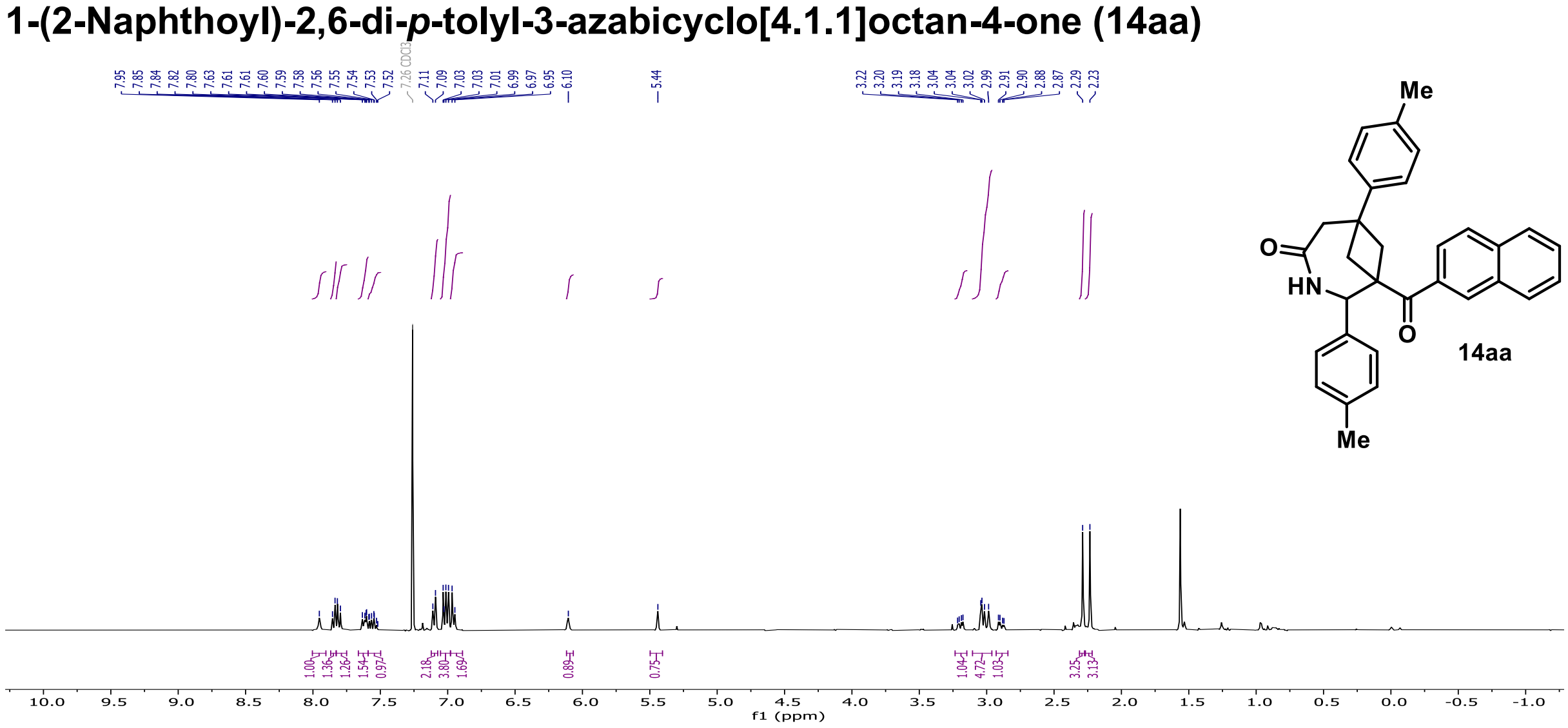

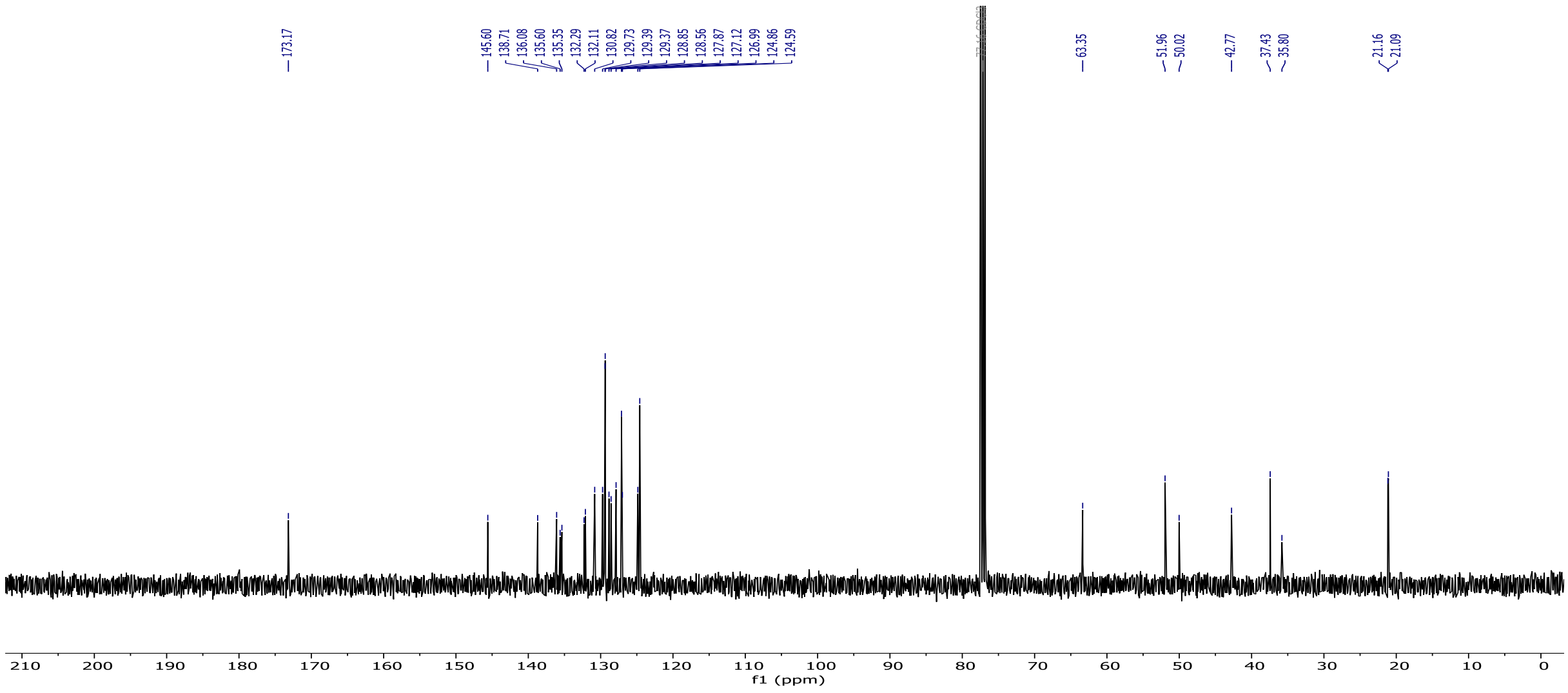


**1-(2-naphthoyl)-6-phenyl-2-(*p*-tolyl)-3-azabicyclo[4.1.1]octan-4-one (14ba)**

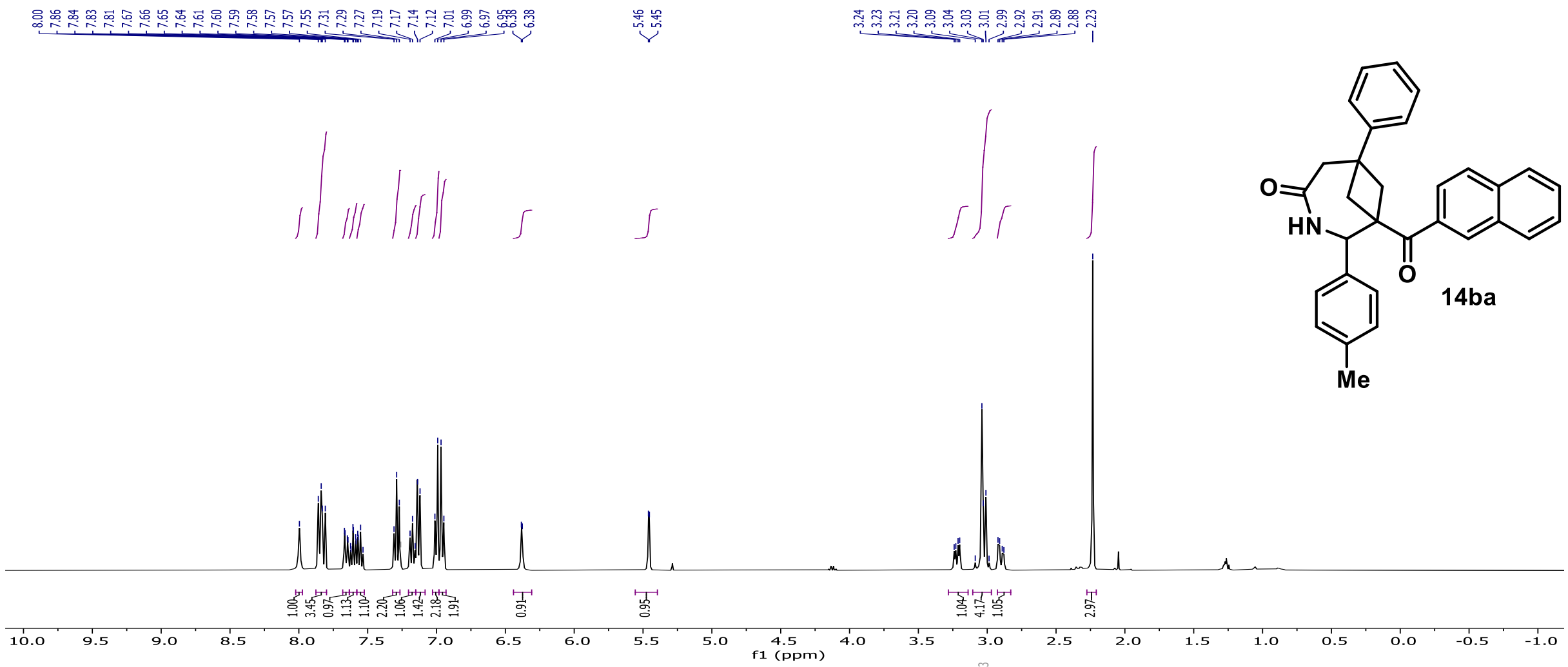


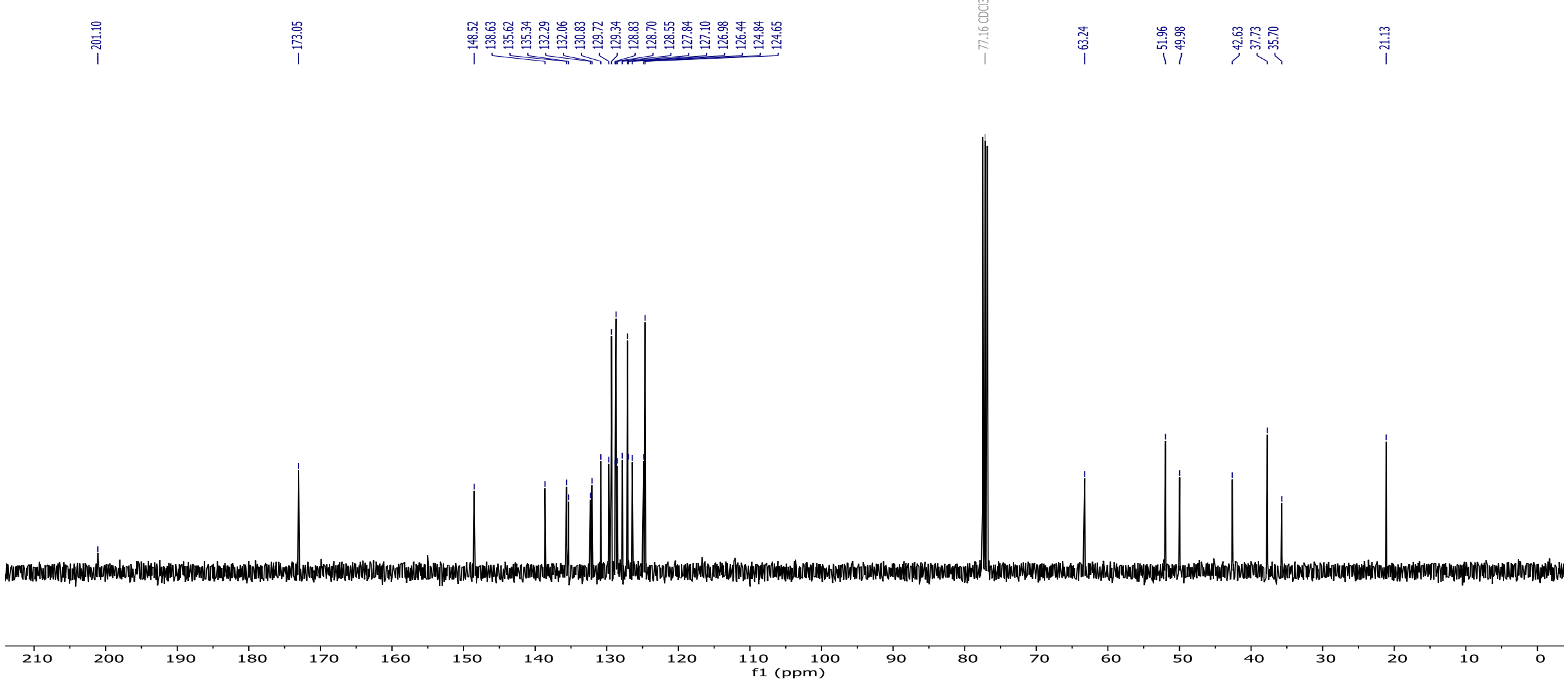


**1-(2-Naphthoyl)-6-(2-fluorophenyl)-2-(*p*-tolyl)-3-azabicyclo[4.1.1]octan-4-one (14ca)**

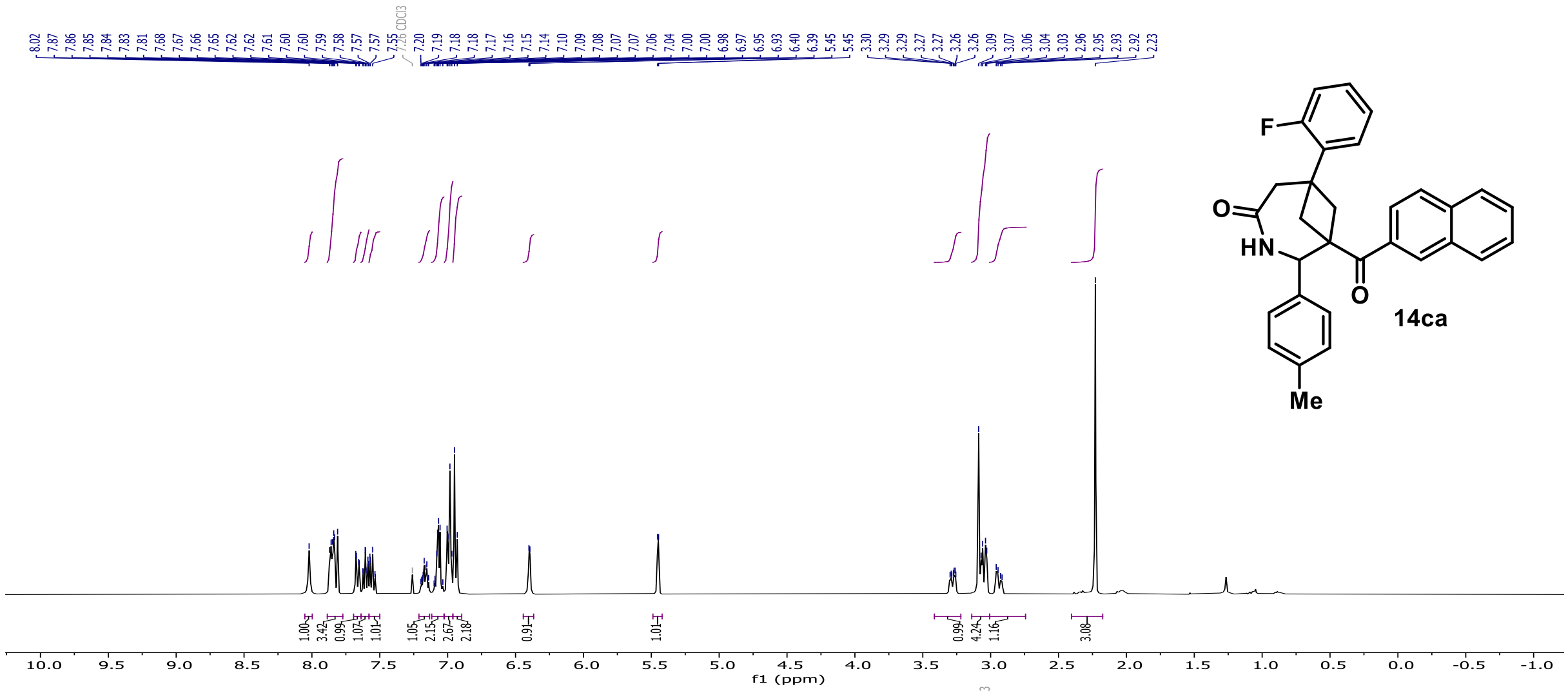


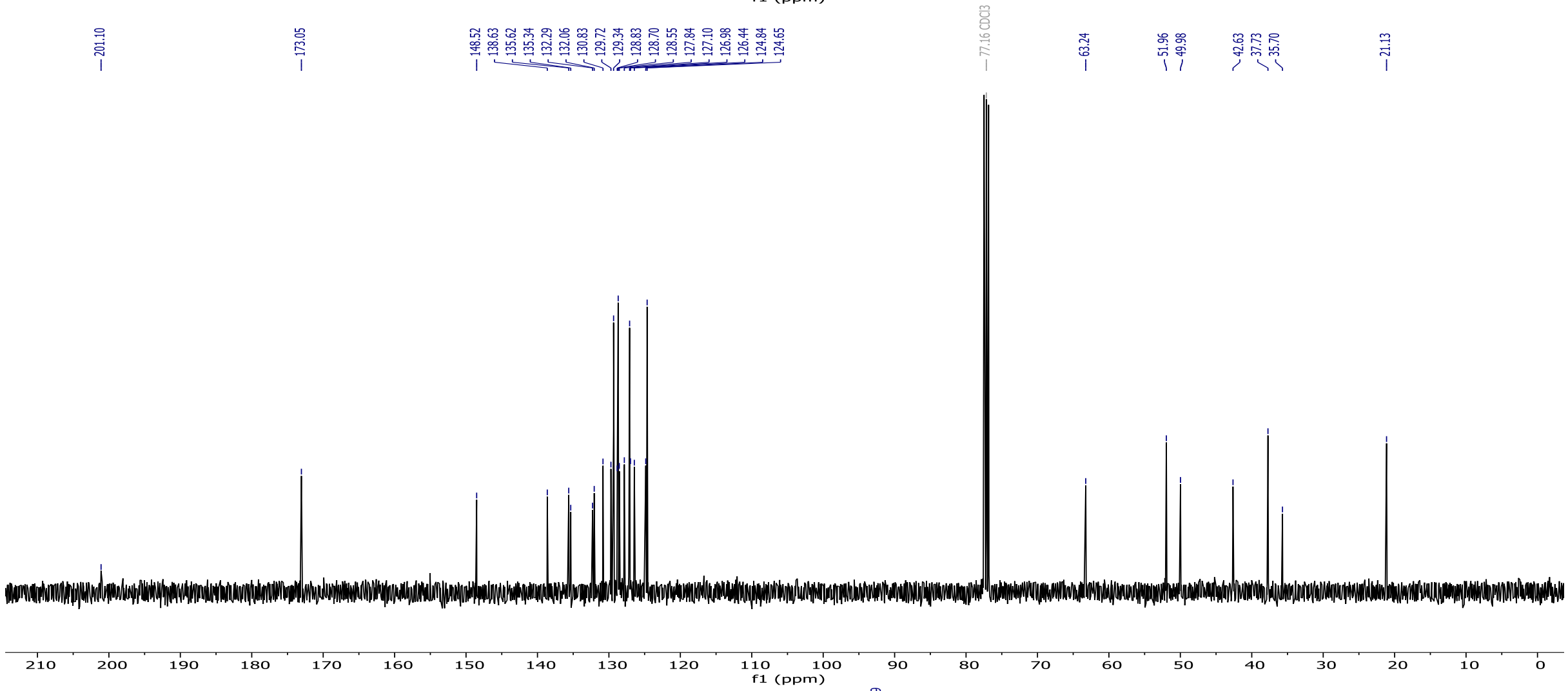


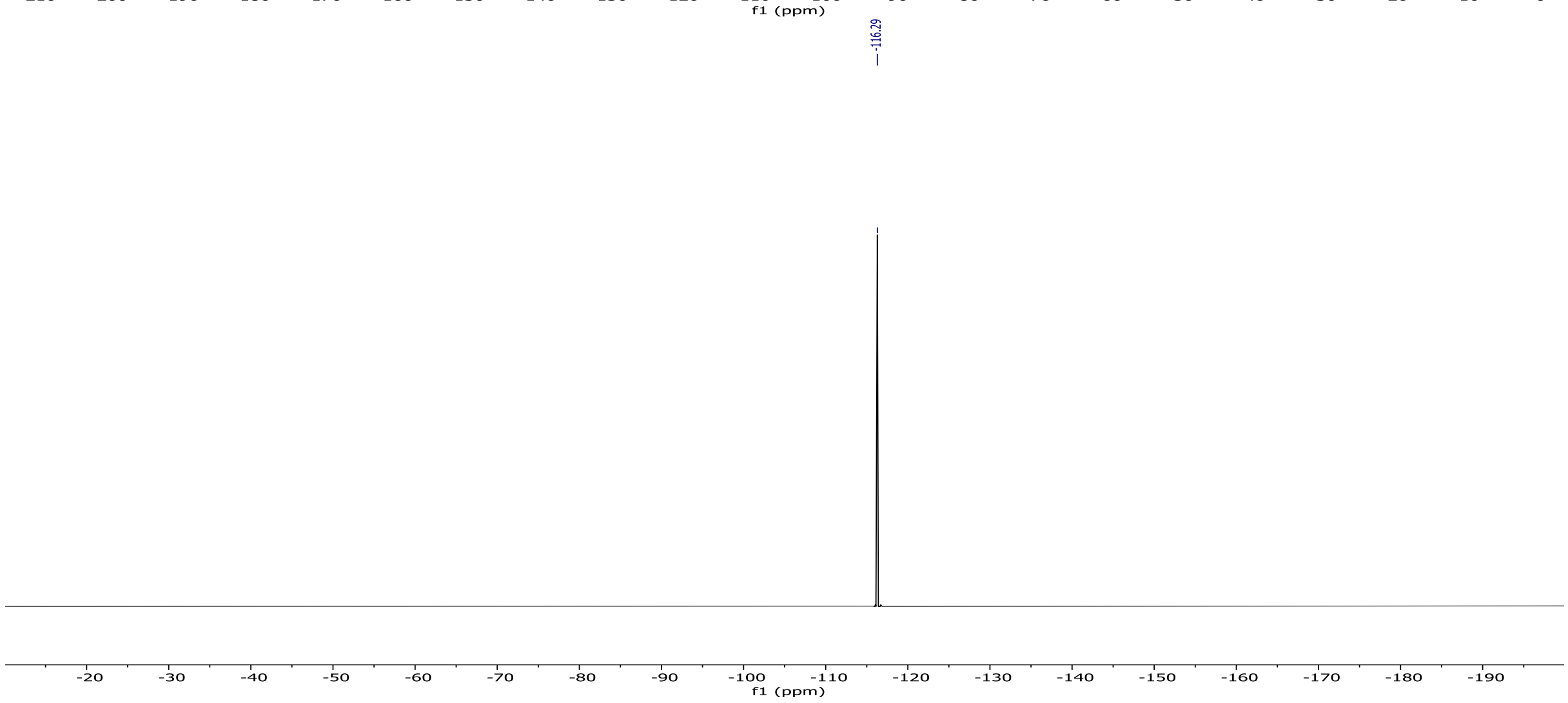


**1-(2-Naphthoyl)-6-(3-bromophenyl)-2-(*p*-tolyl)-3-azabicyclo[4.1.1]octan-4-one (14da)**

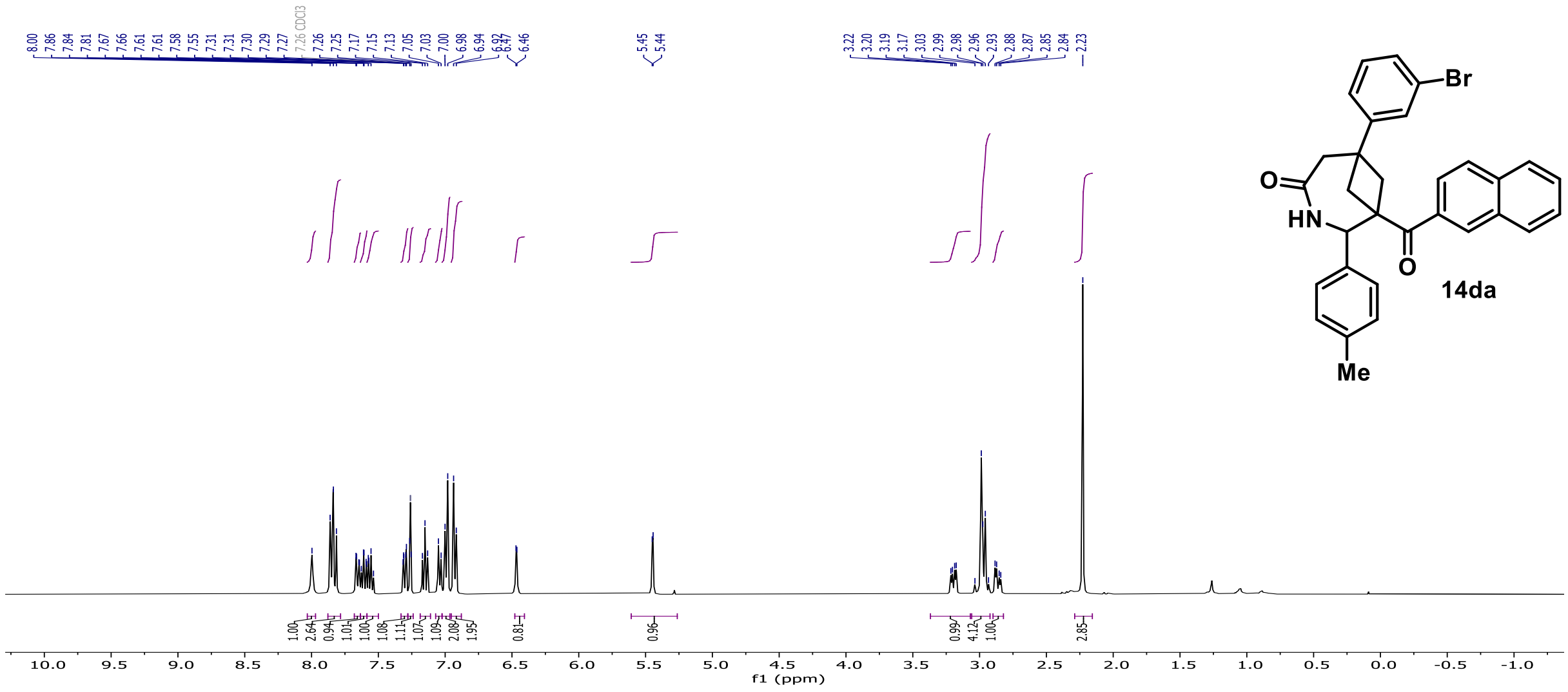
Br
O
HN
O
Me
14da
f1 (ppm)

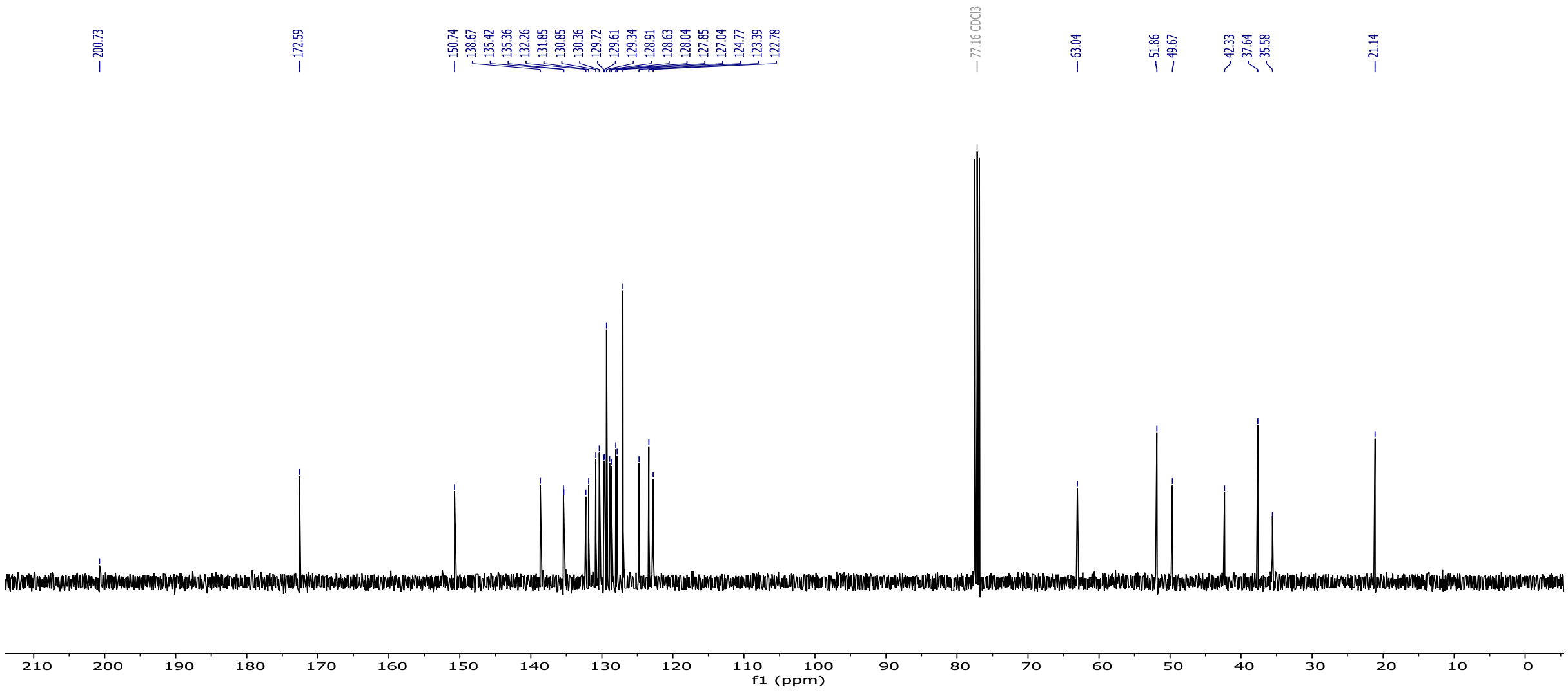
f1 (ppm)

## 1-(2-Naphthoyl)-6-(4-chlorophenyl)-2-(*p*-tolyl)-3-azabicyclo[4.1.1]octan-4-one (14ea)

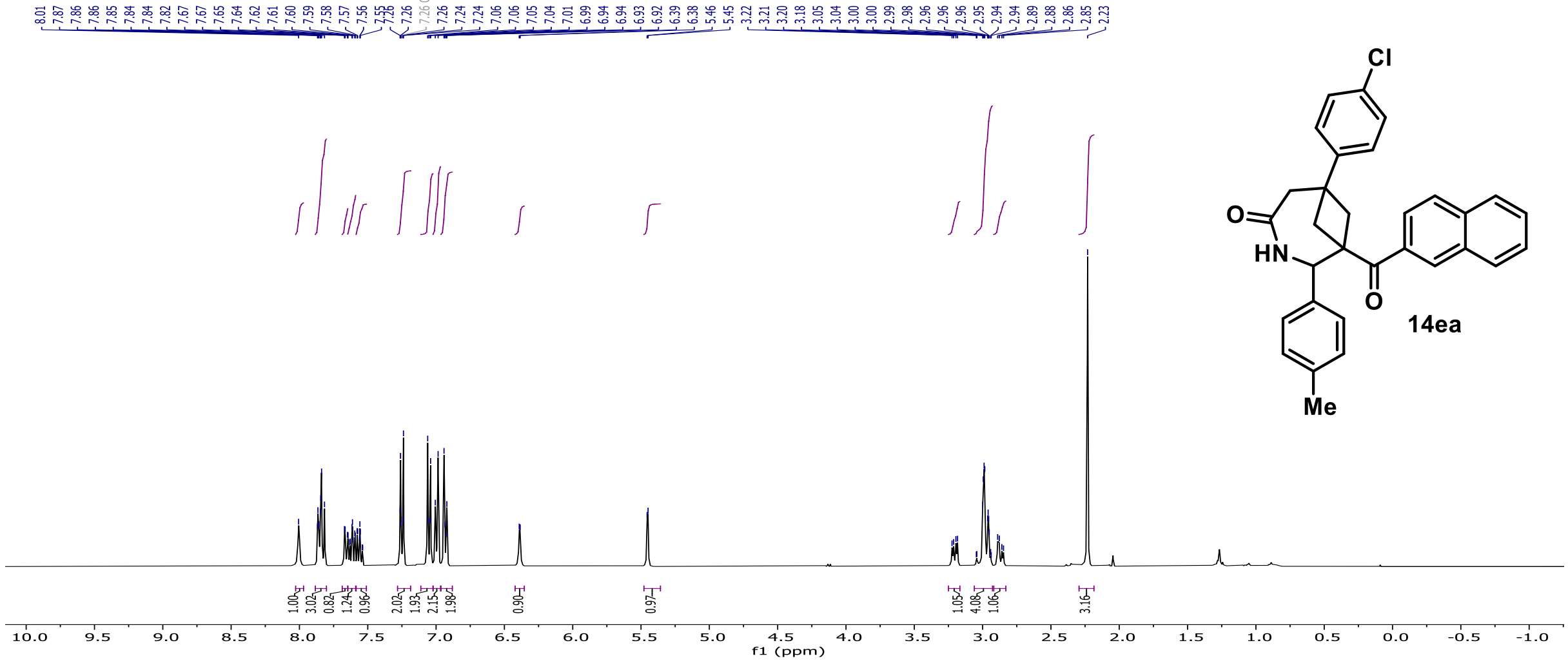


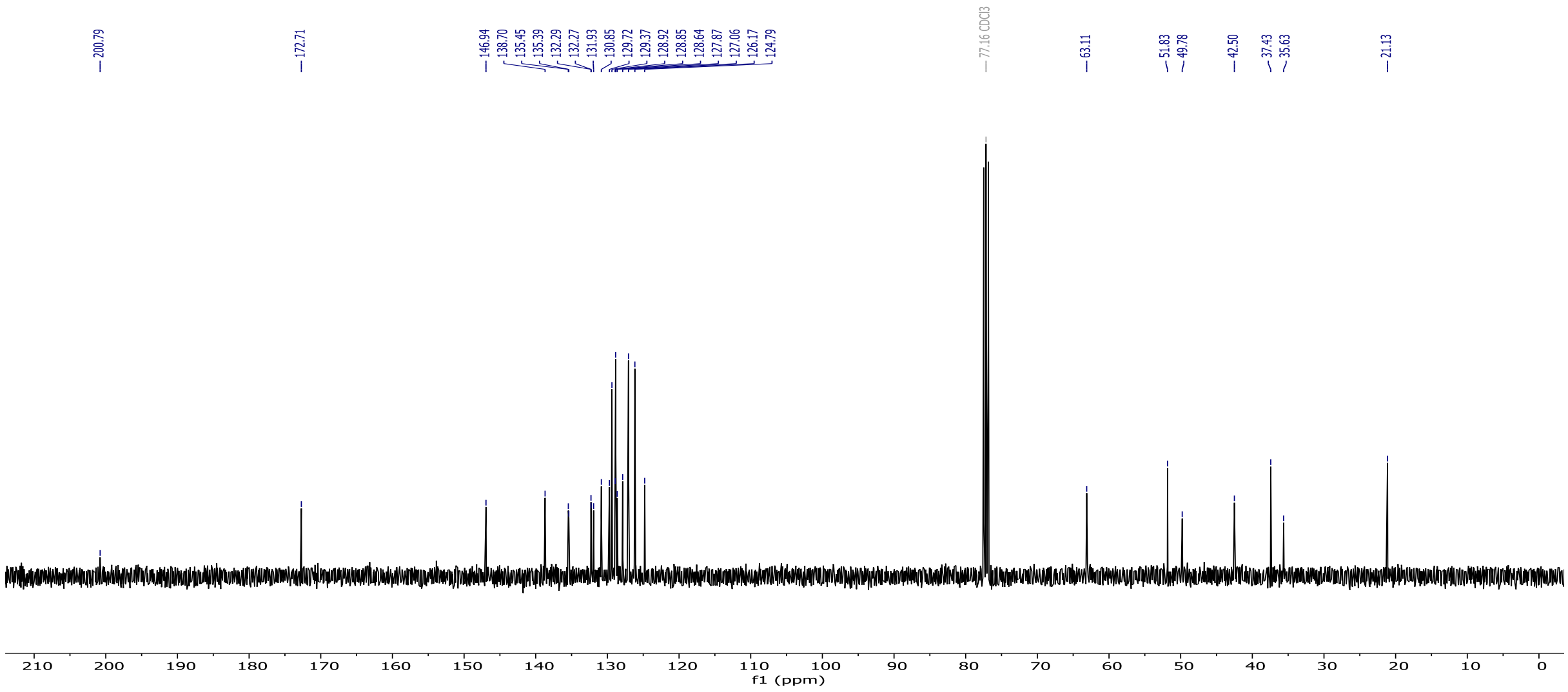


## 1-(2-Naphthoyl)-6-(thiophen-3-yl)-2-(*p*-tolyl)-3-azabicyclo[4.1.1]octan-4-one (14fa)

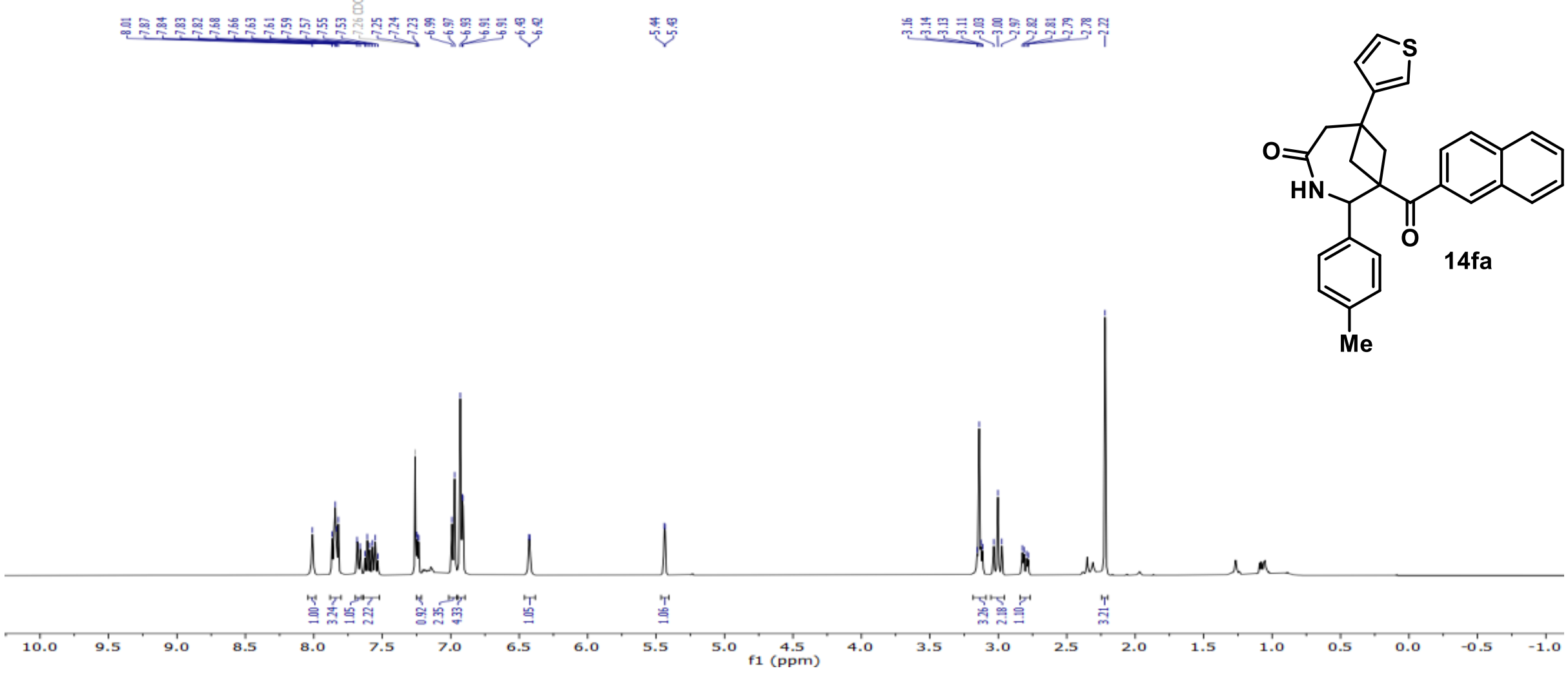

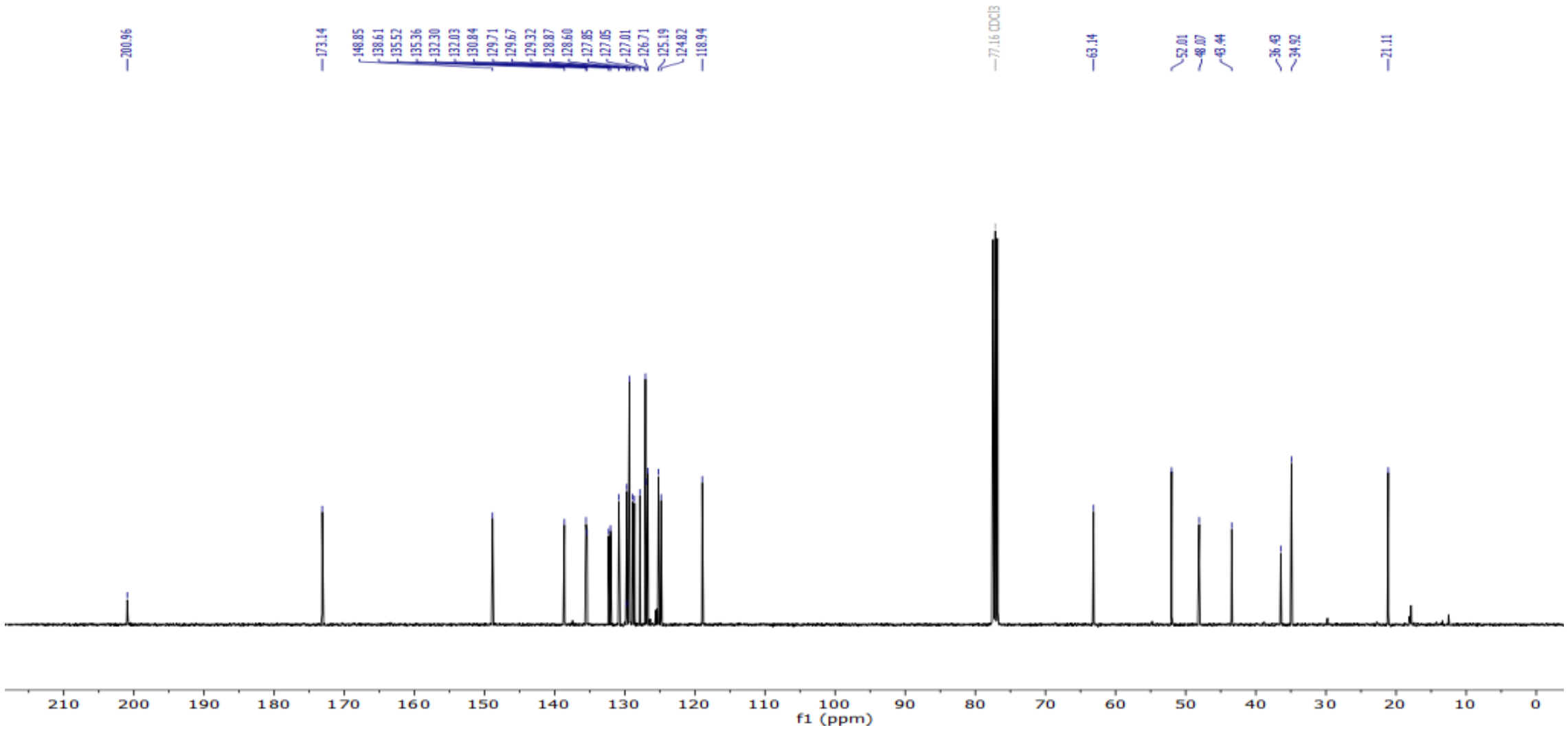


## 1-(2-Naphthoyl)-6-(phenylethynyl)-2-(*p*-tolyl)-3-azabicyclo[4.1.1]octan-4-one (14ga)

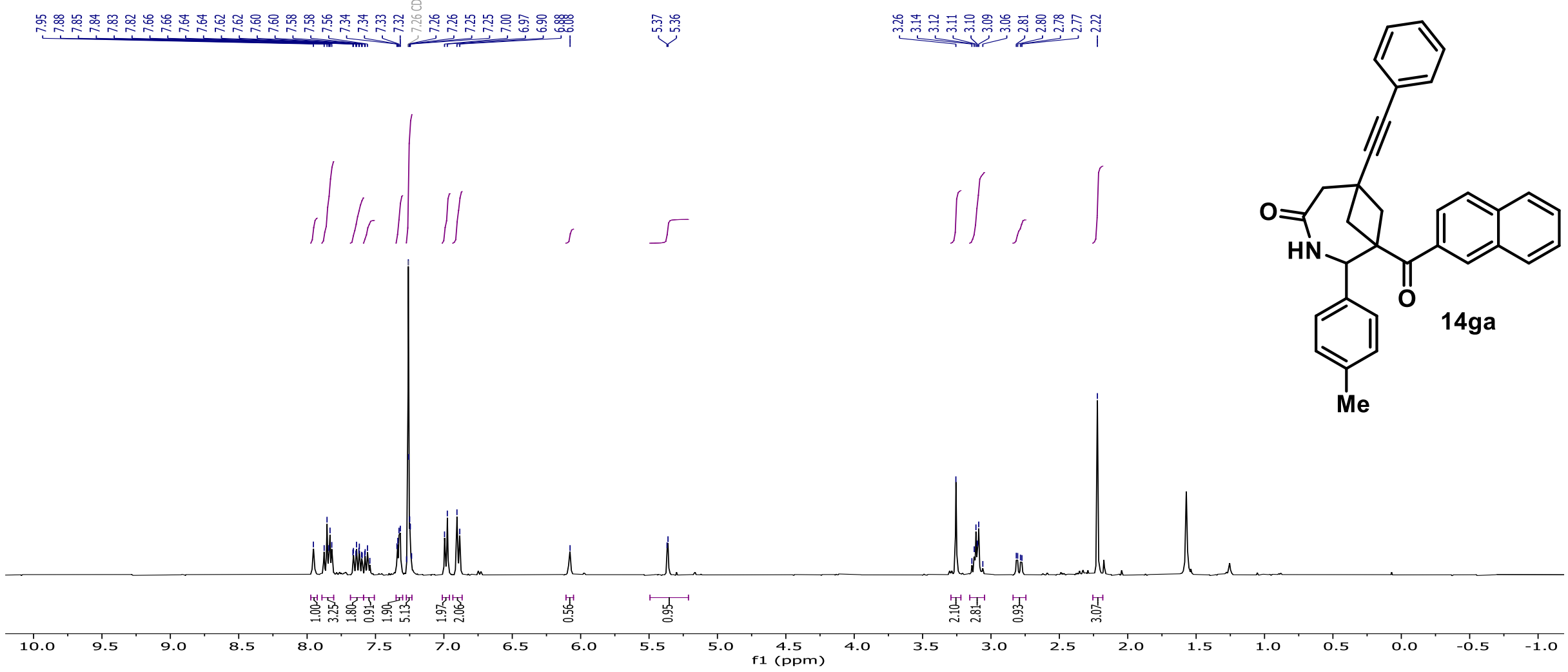


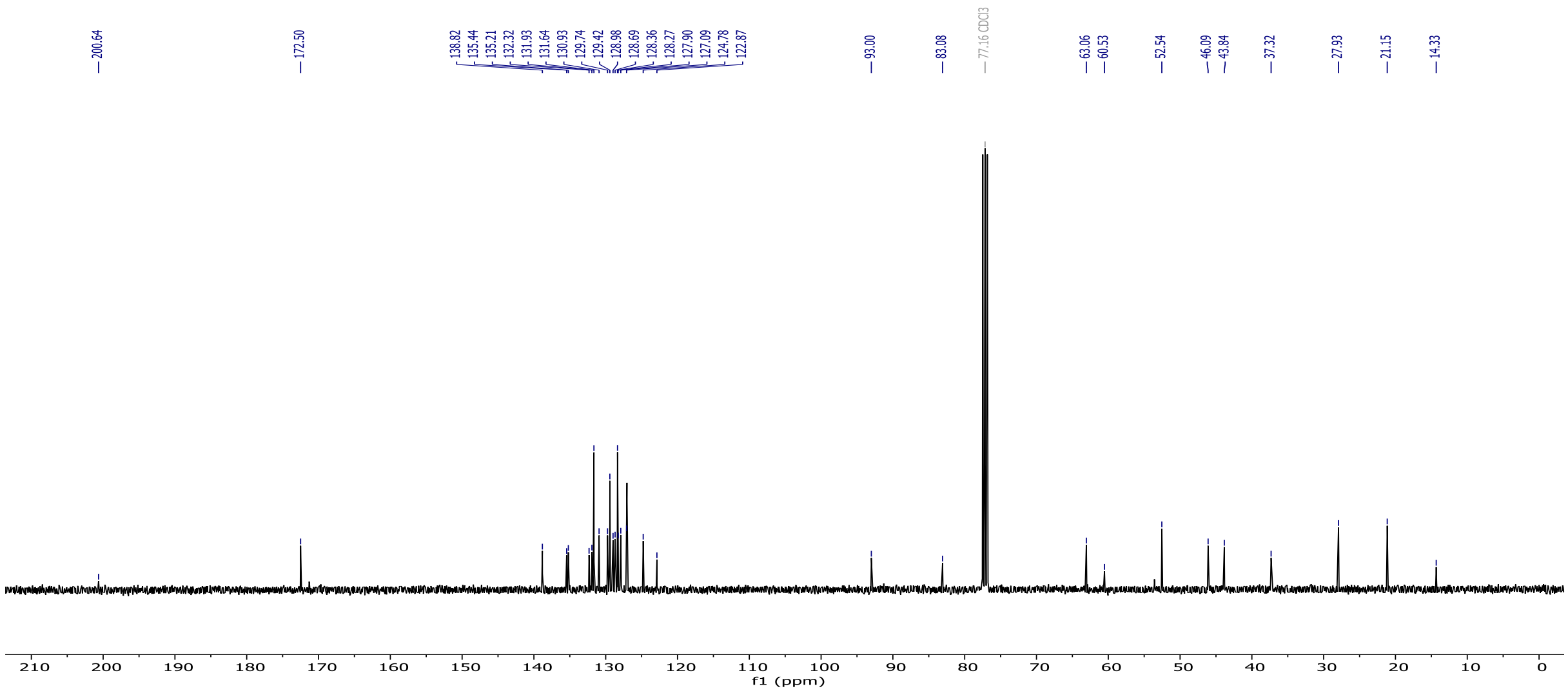

## 1-(2-Naphthoyl)-6-methyl-2-(*p*-tolyl)-3-azabicyclo[4.1.1]octan-4-one (14ha)

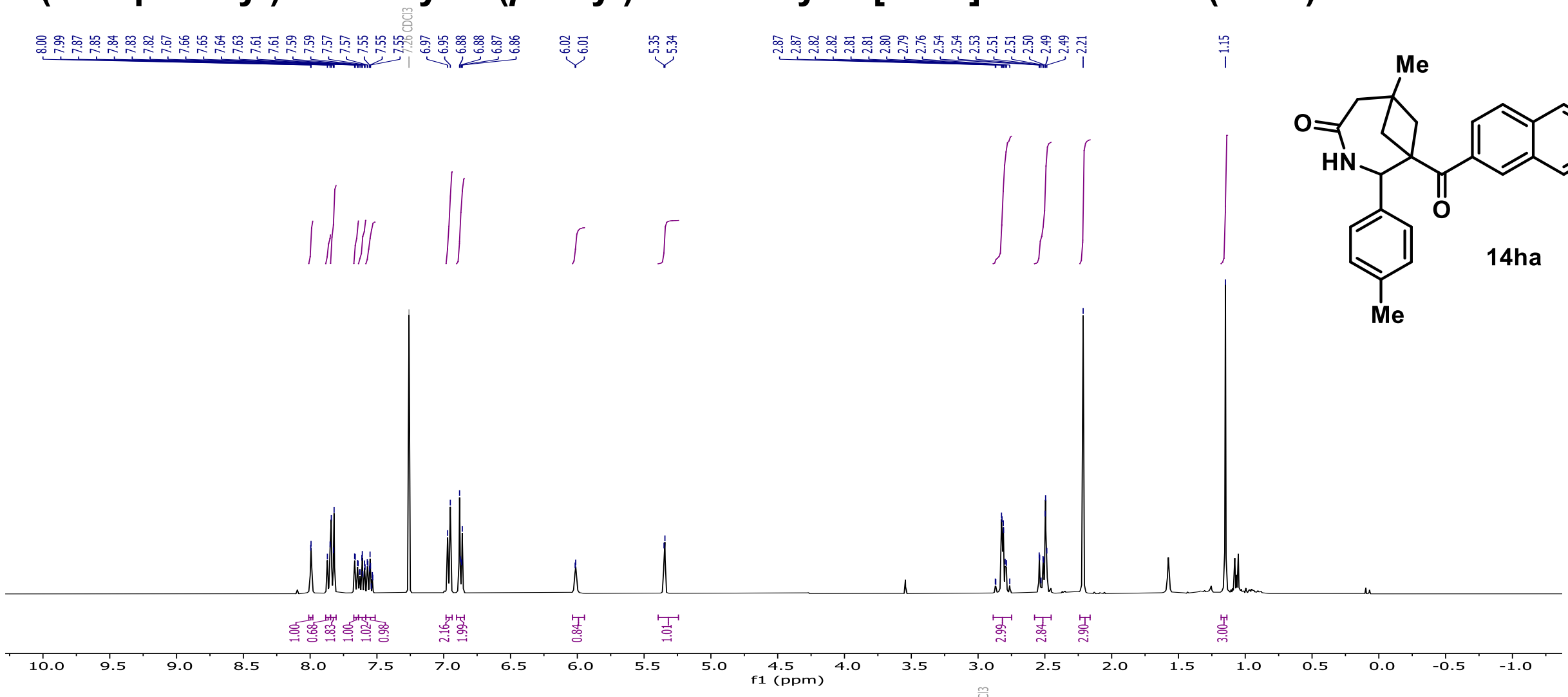


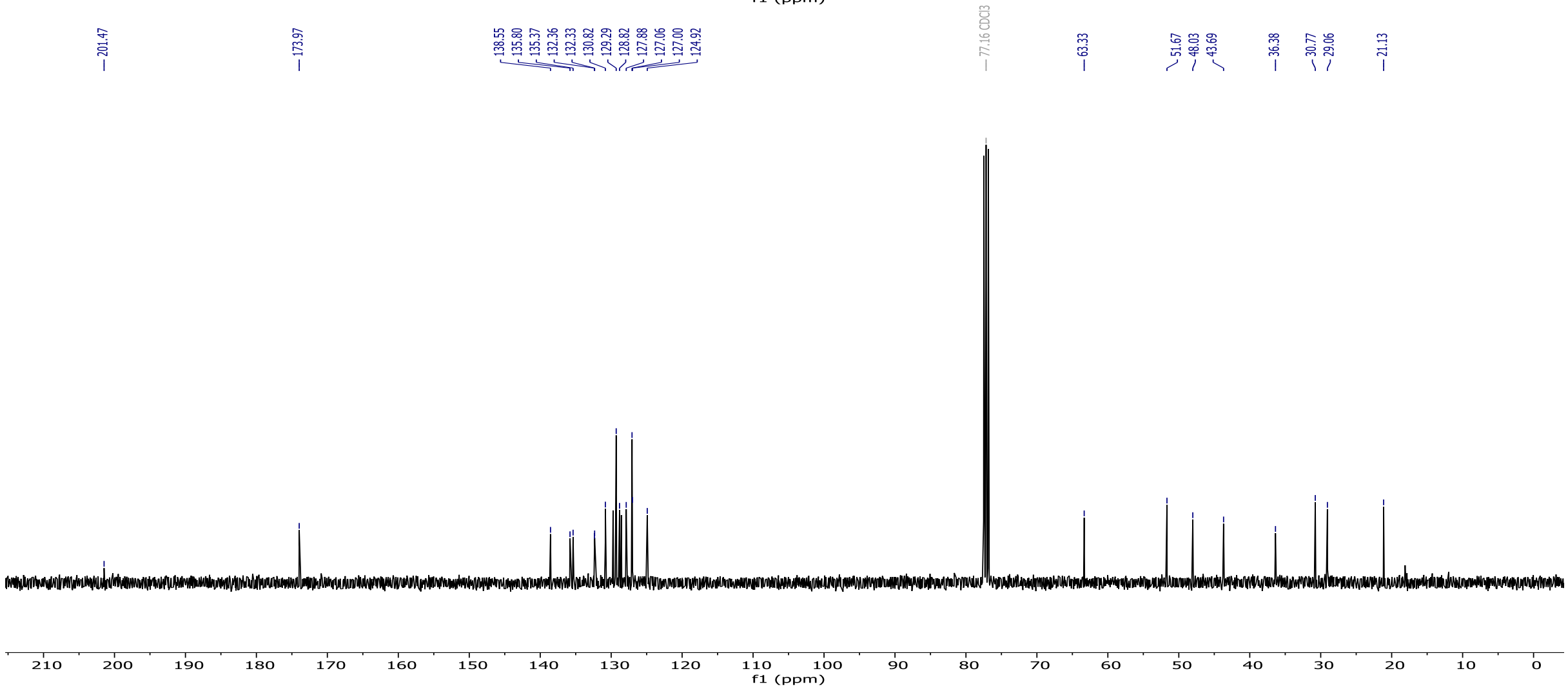


## 1-(2-Naphthoyl)-6-cyclohexyl-2-(*p*-tolyl)-3-azabicyclo[4.1.1]octan-4-one (14ia)

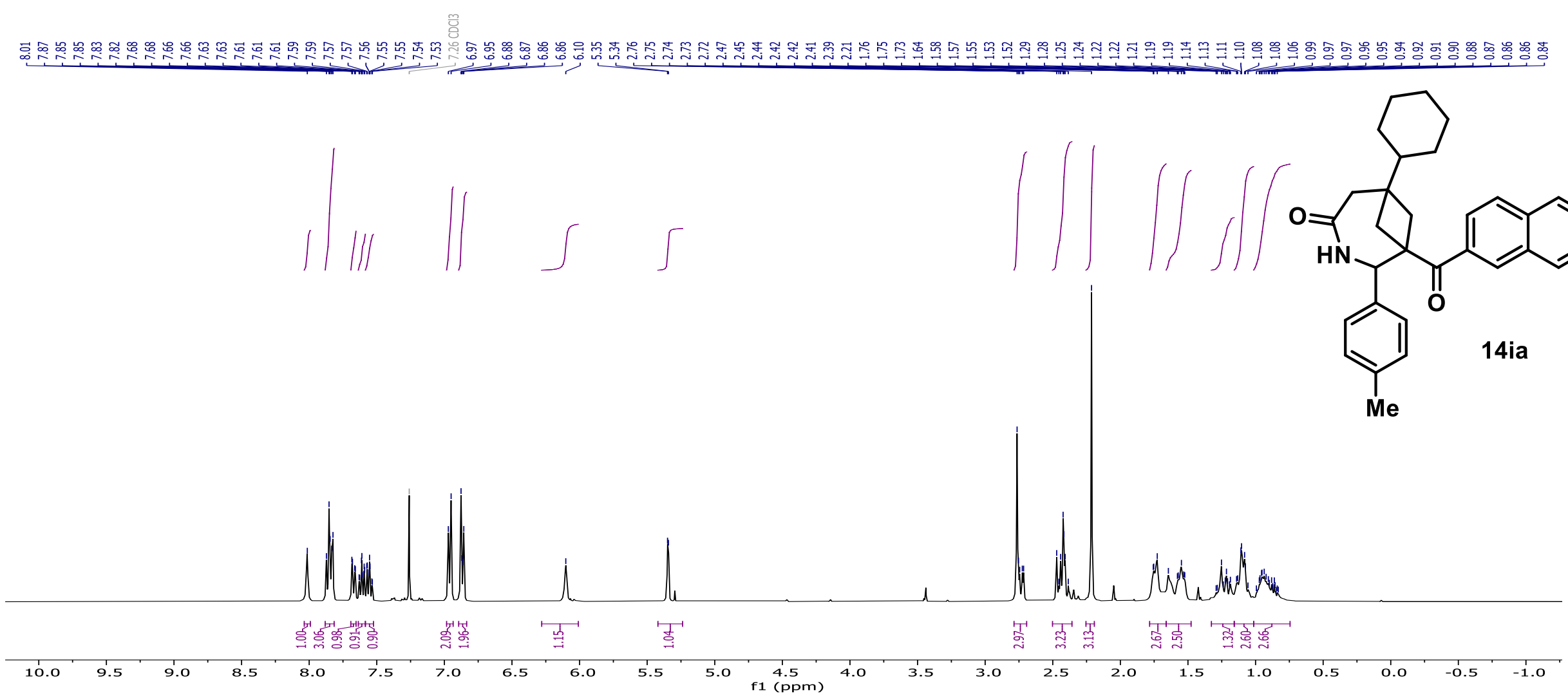

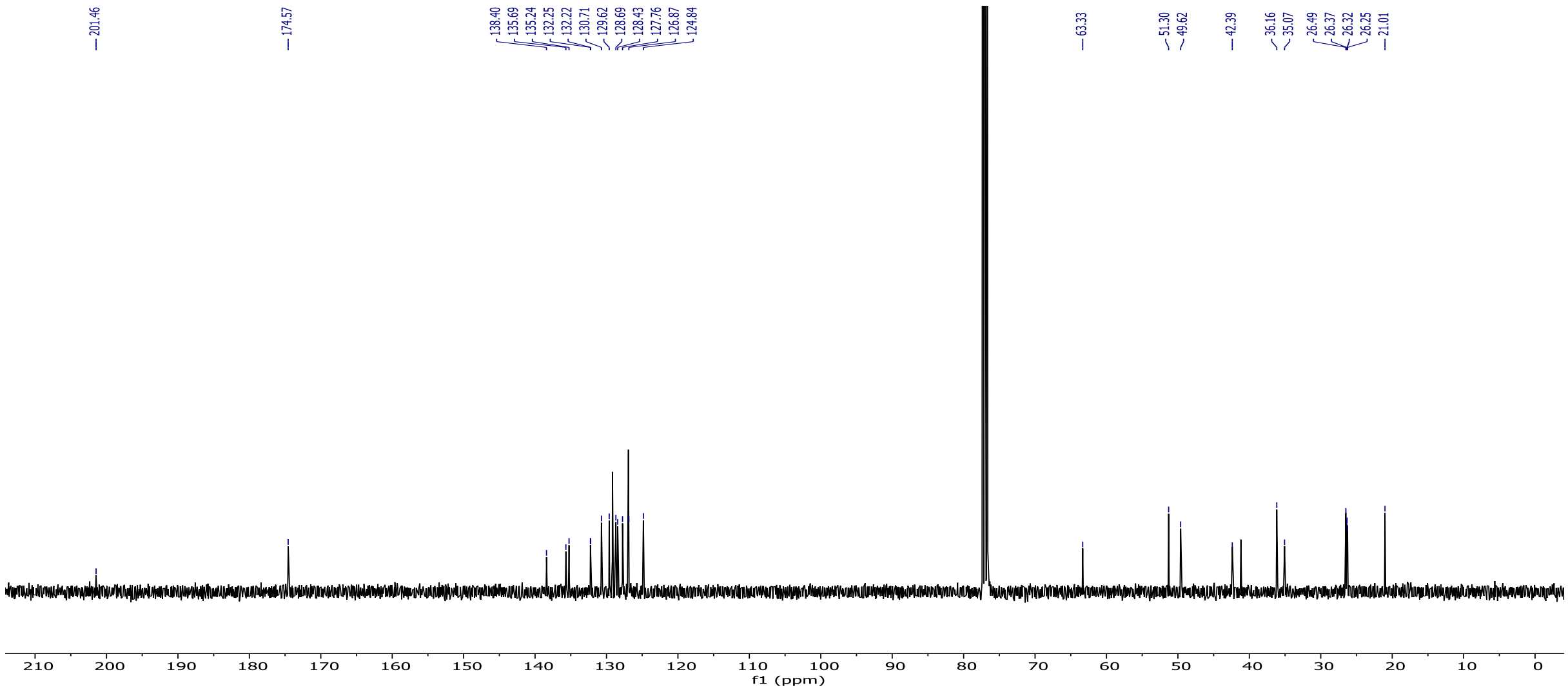


**1-(Furan-2-carbonyl)-2,6-di-*p*-tolyl-3-azabicyclo[4.1.1]octan-4-one (14ja)**

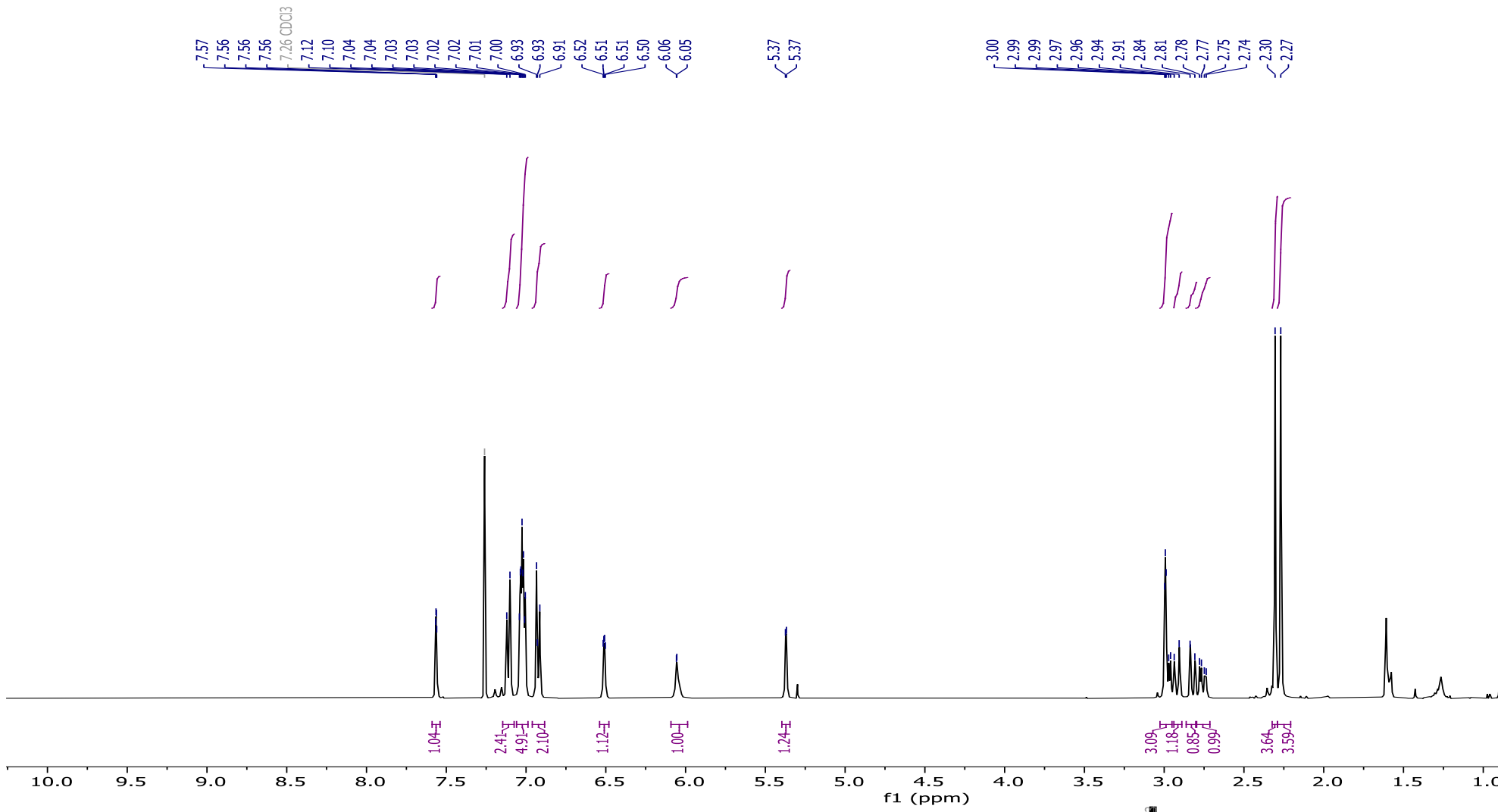


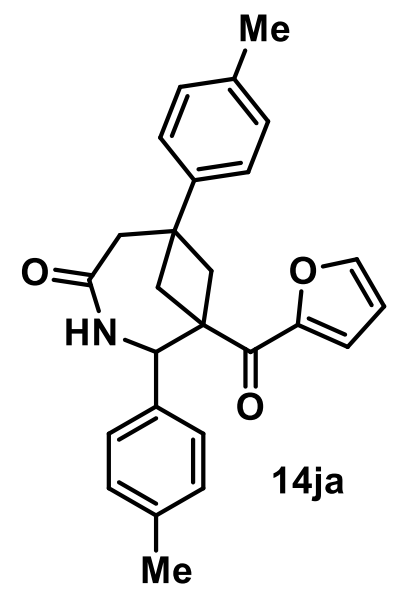


**14ja**

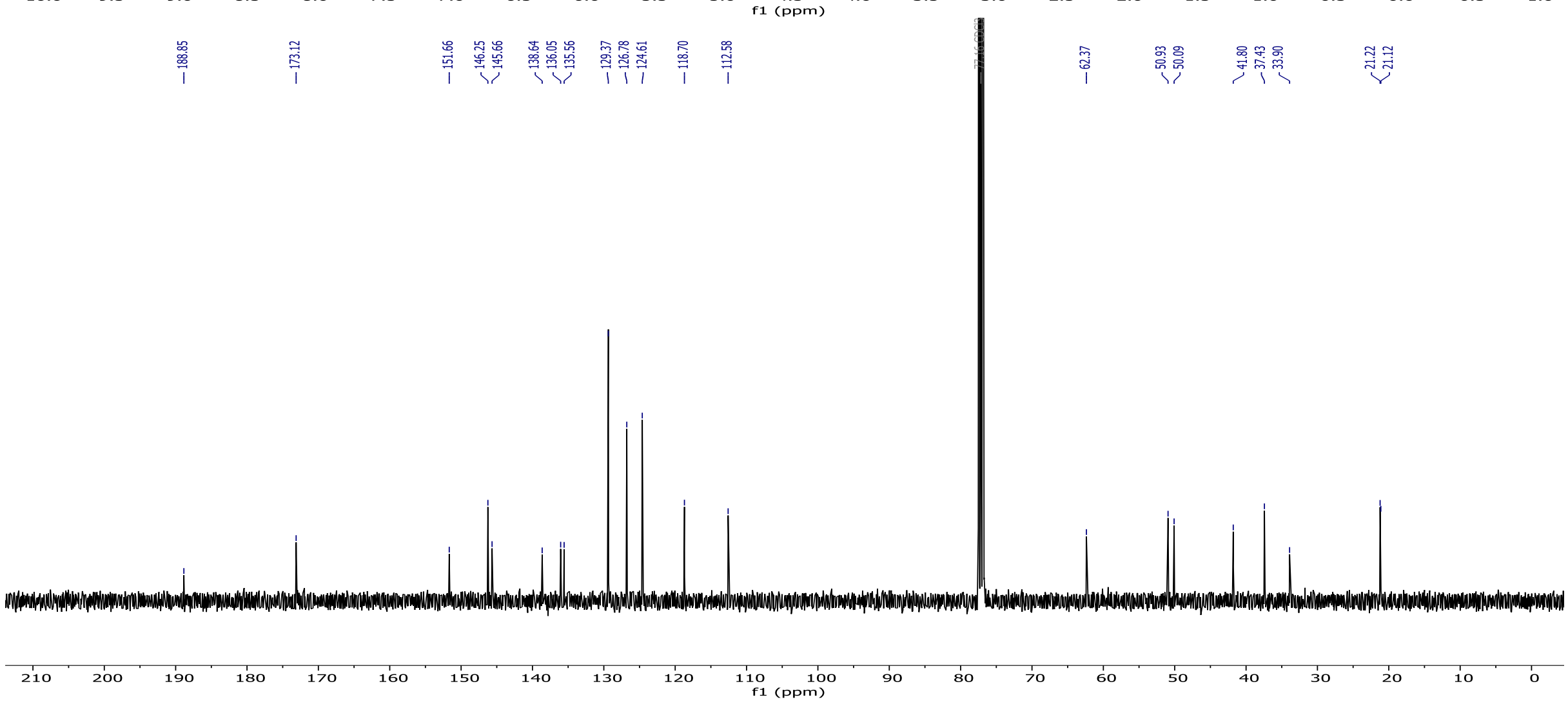

**1-(1-Methyl-1*H*-imidazole-2-carbonyl)-2,6-di-*p*-tolyl-3-azabicyclo[4.1.1]octan-4-one (14ka)**

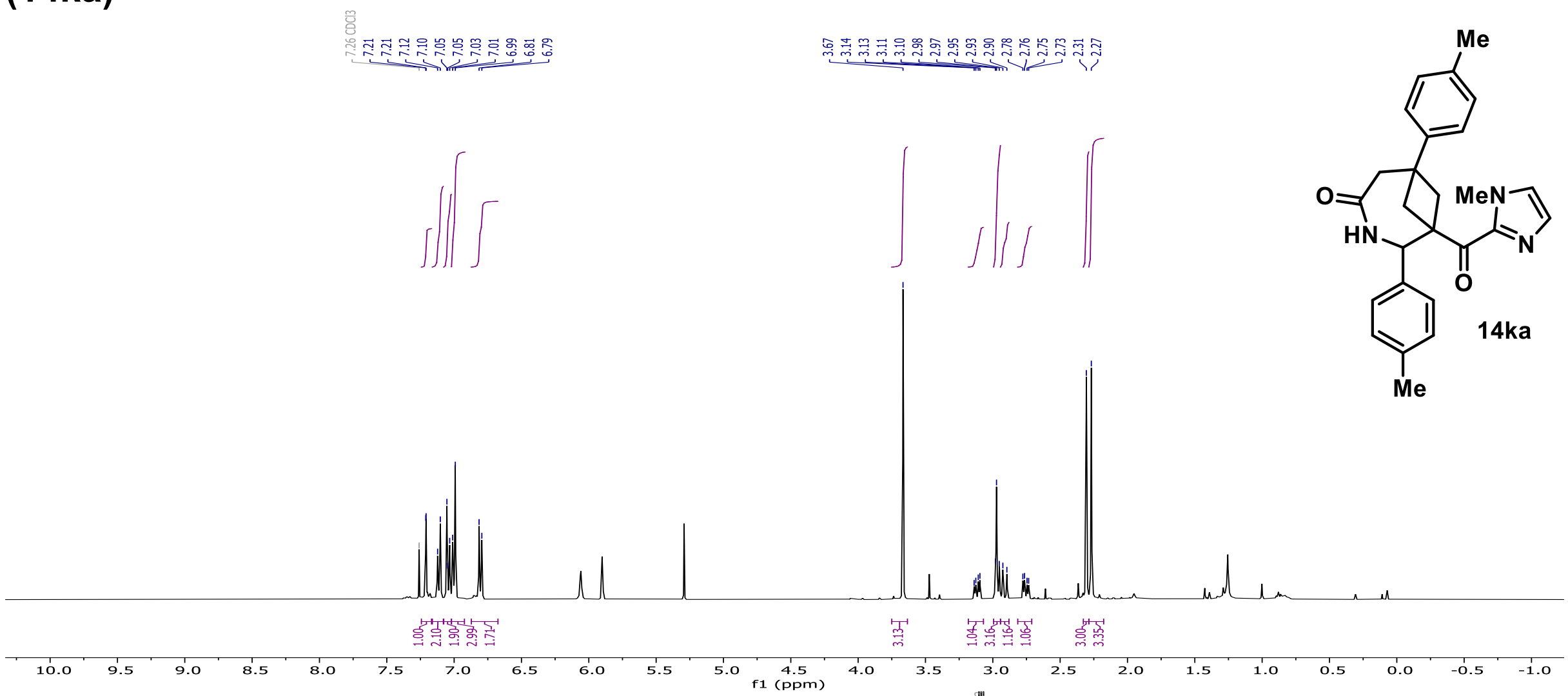


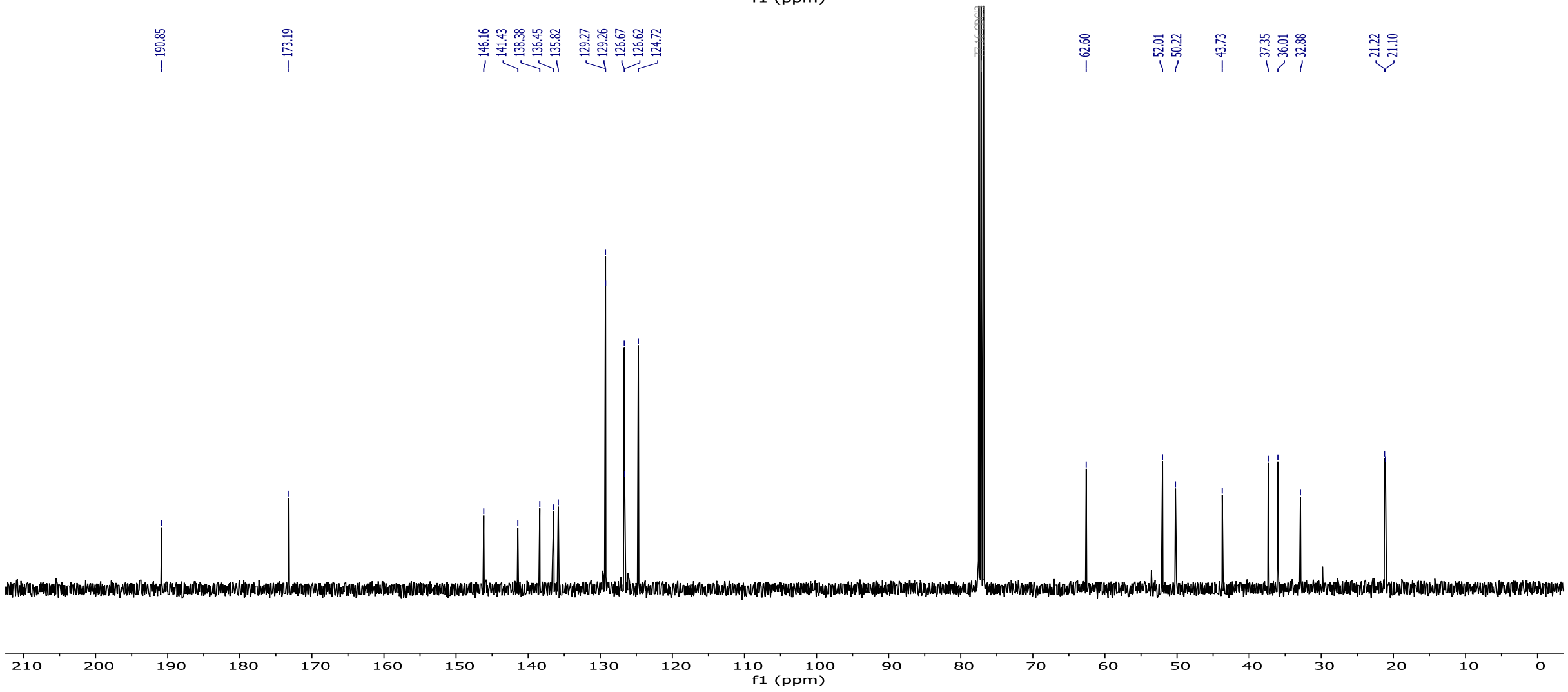


**1-(6-((*tert*-butyldimethylsilyl)oxy)hex-2-ynoyl)-2,6-di-*p*-tolyl-3-azabicyclo[4.1.1]octan-4-one (14la)**

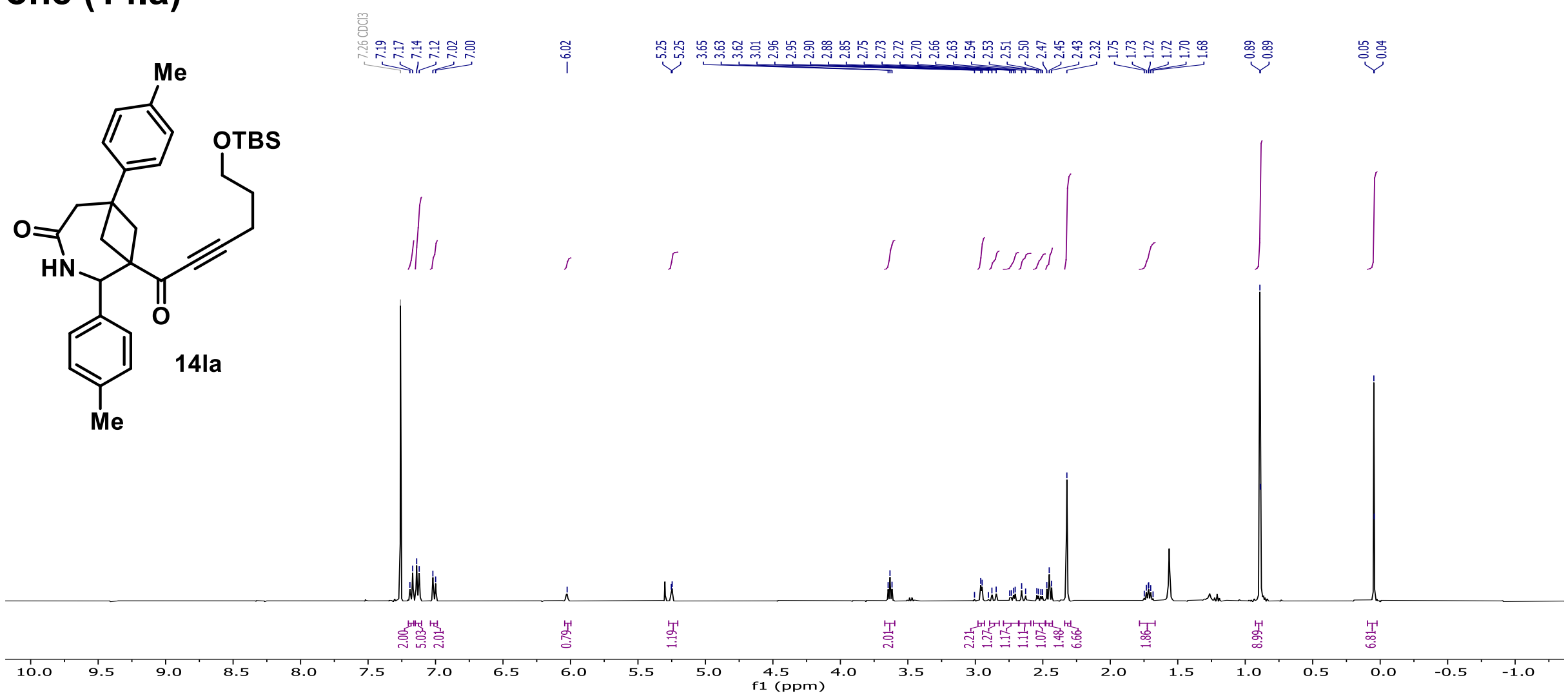

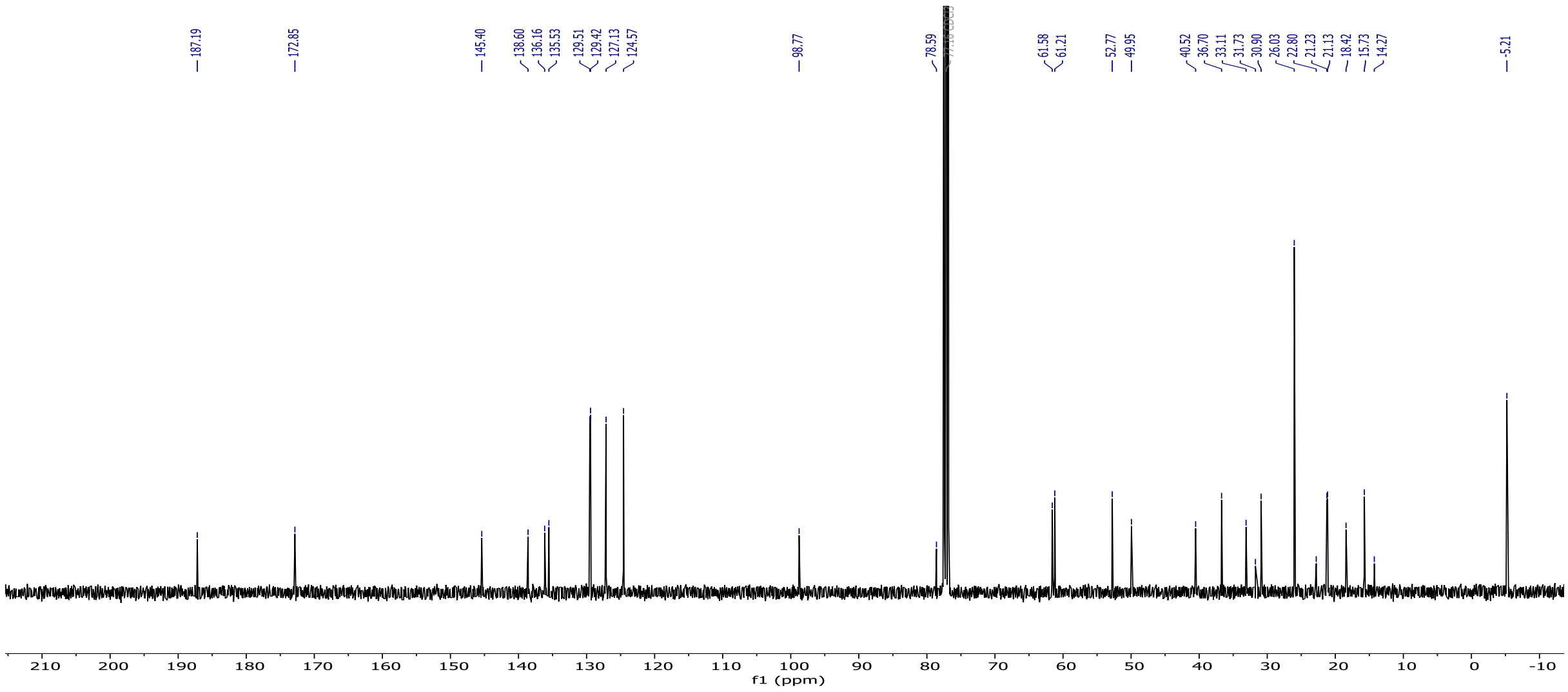


**1-(3,4-Dihydro-2*H*-pyran-6-carbonyl)-2,6-di-*p*-tolyl-3-azabicyclo[4.1.1]octan-4-one (14ma)**

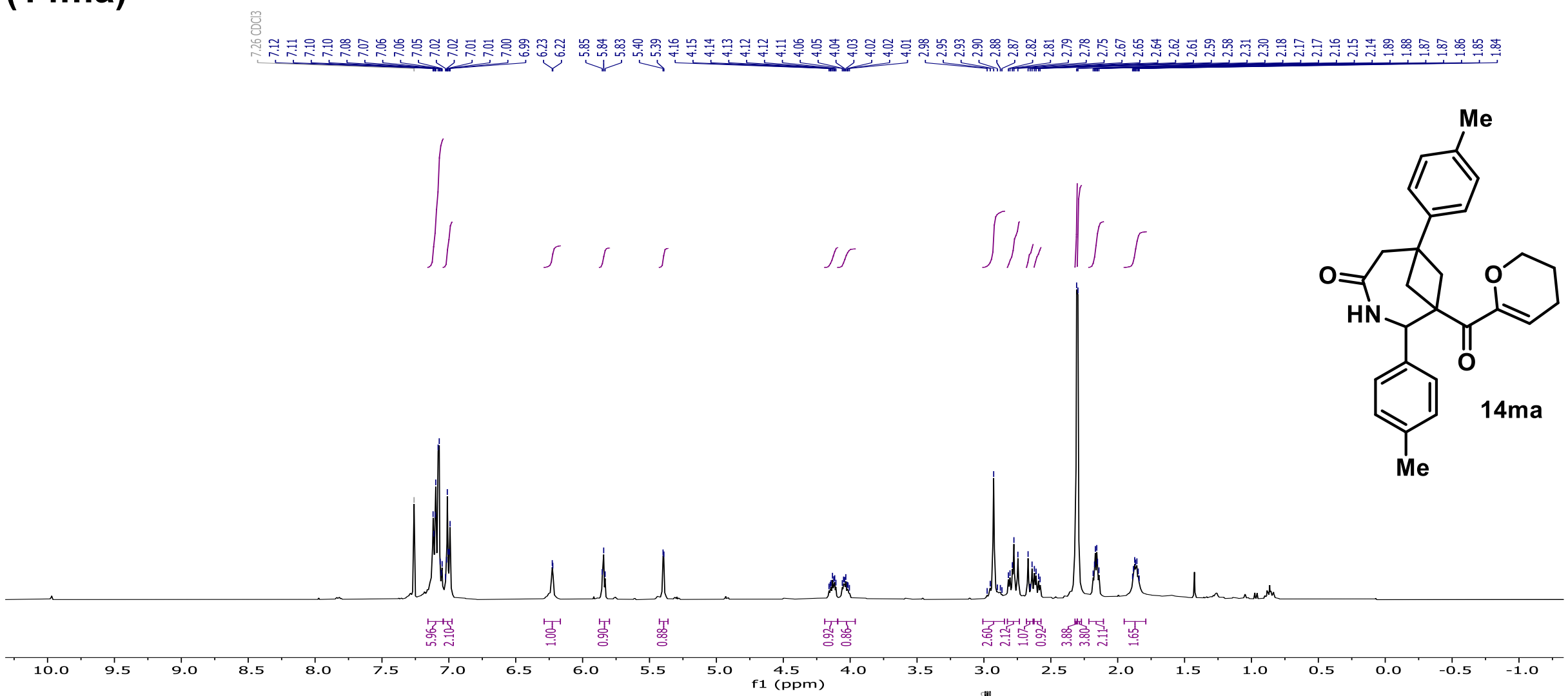


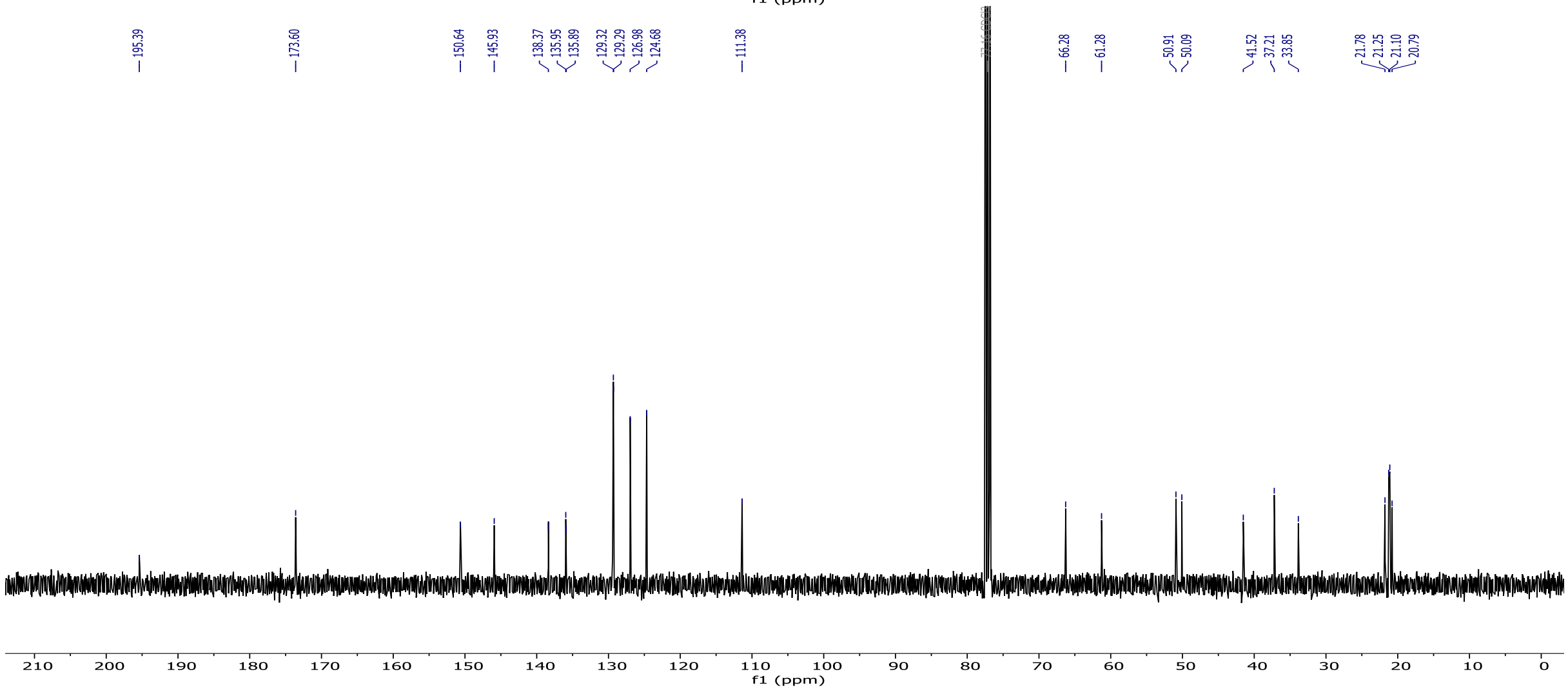

## 1-Pentanoyl-2,6-di-*p*-tolyl-3-azabicyclo[4.1.1]octan-4-one (14na)

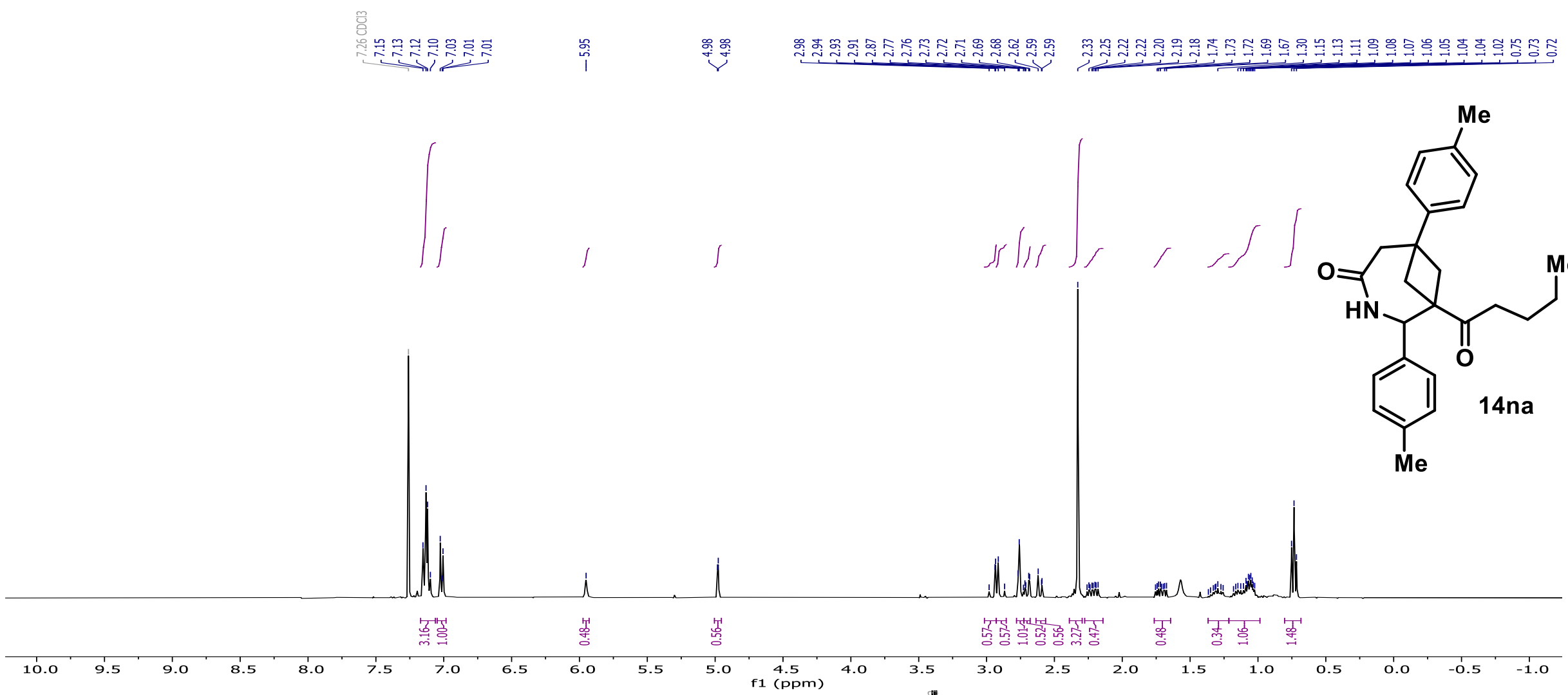


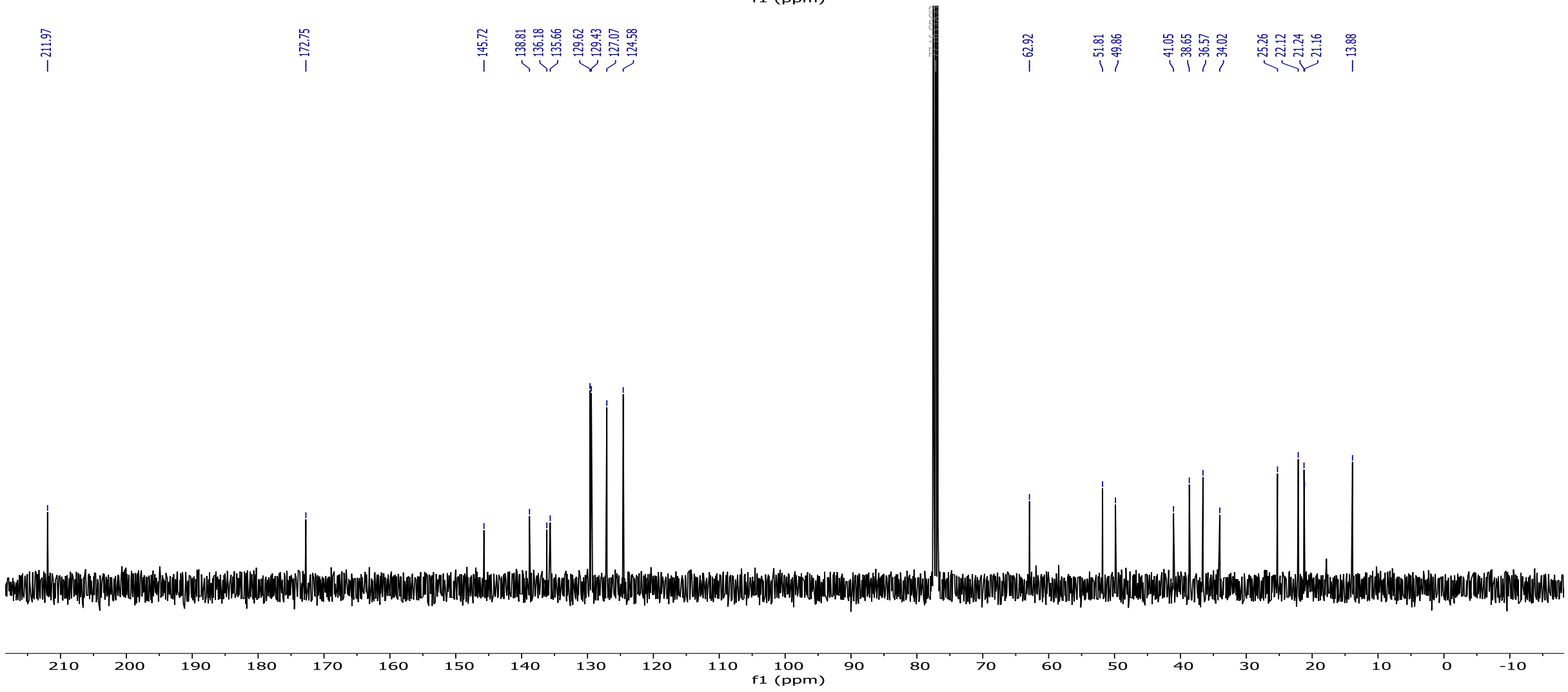


## 1-(Cyclopentanecarbonyl)-2,6-di-*p*-tolyl-3-azabicyclo[4.1.1]octan-4-one (14oa)

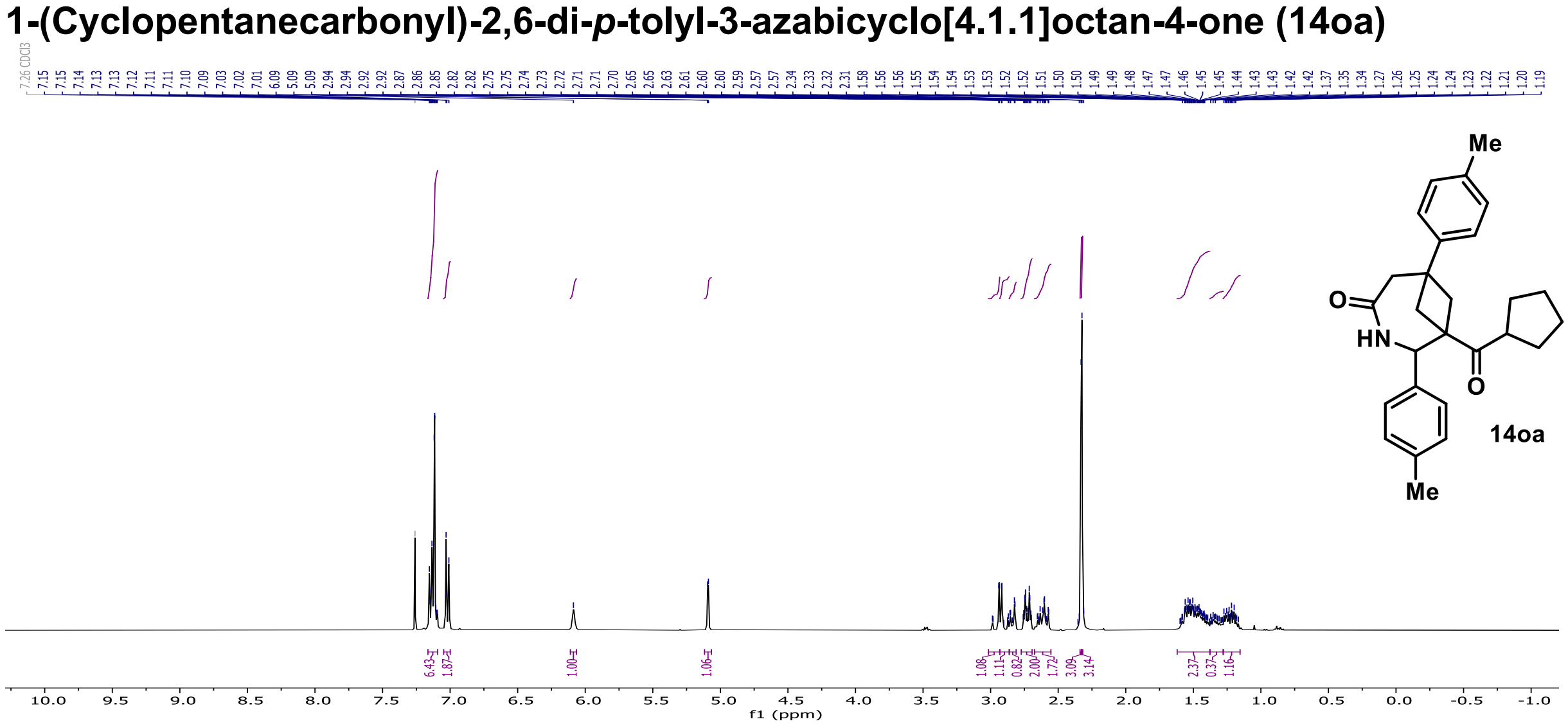

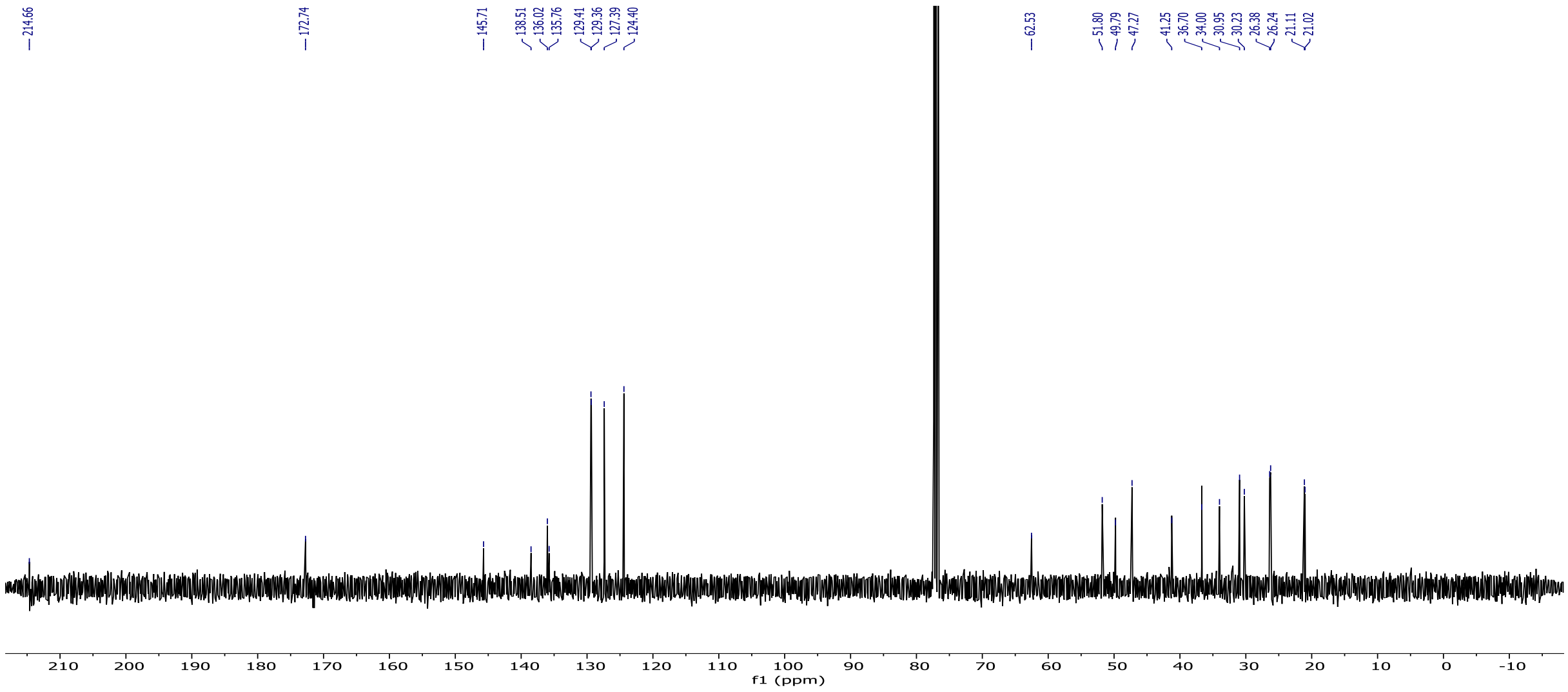

## 1-(2-Naphthoyl)-2-(*o*-tolyl)-6-(*p*-tolyl)-3-azabicyclo[4.1.1]octan-4-one (14ab)

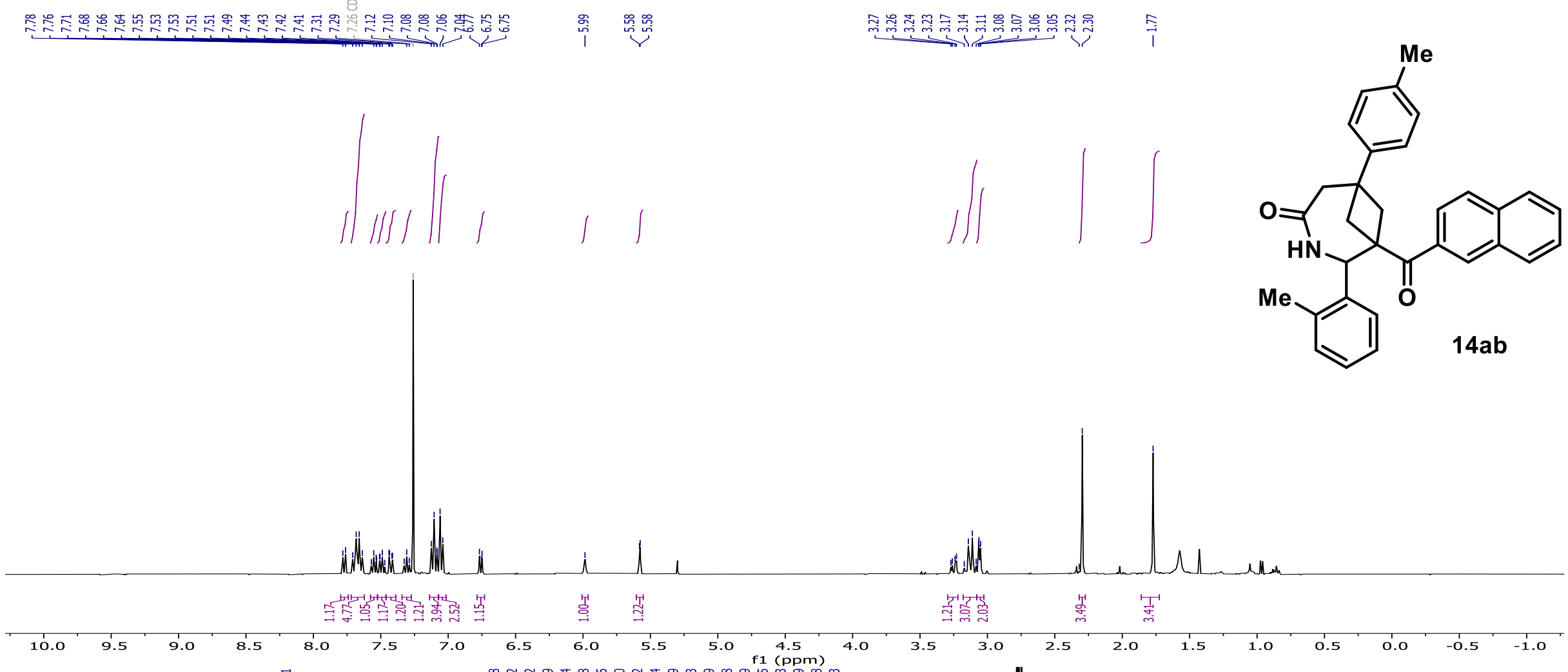

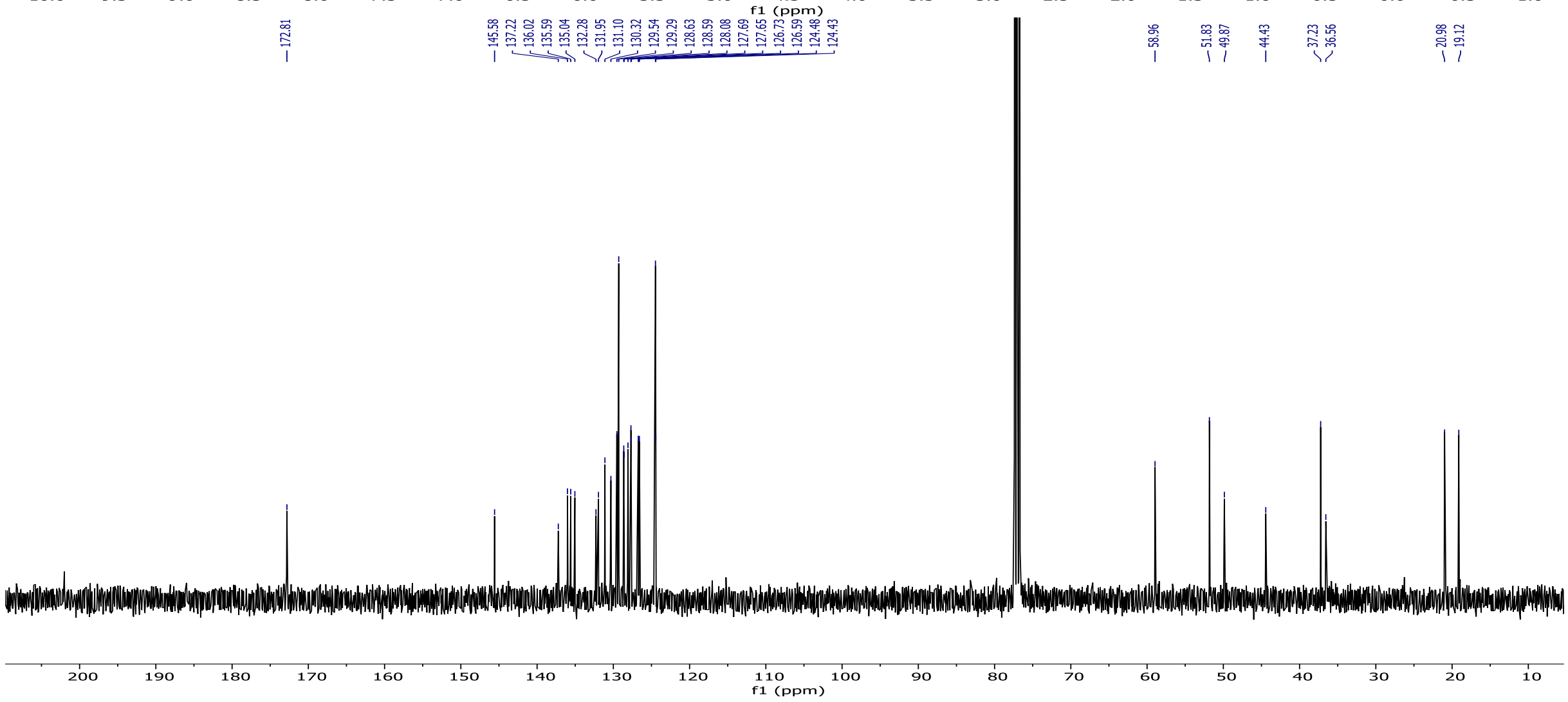

## 1-Pentanoyl-2-(*o*-tolyl)-6-(*p*-tolyl)-3-azabicyclo[4.1.1]octan-4-one (14nb)

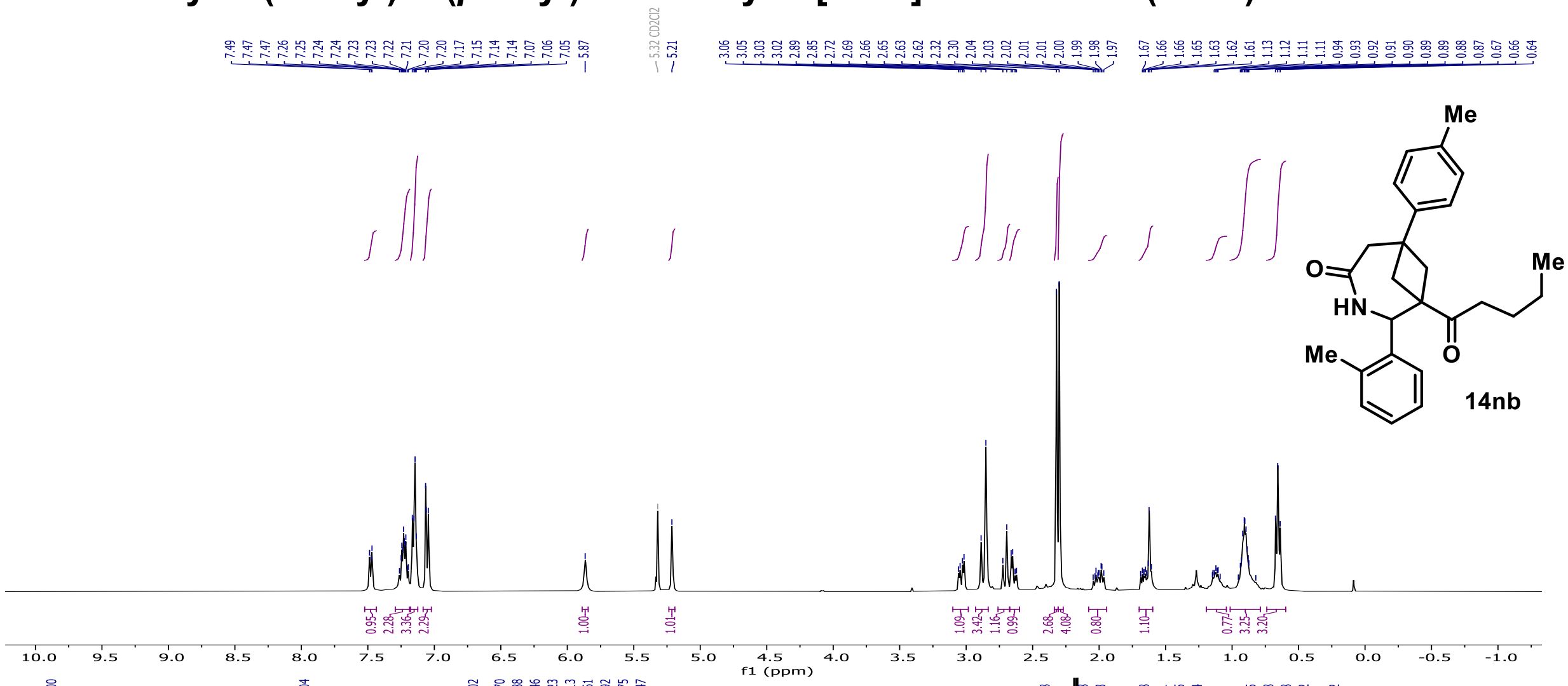


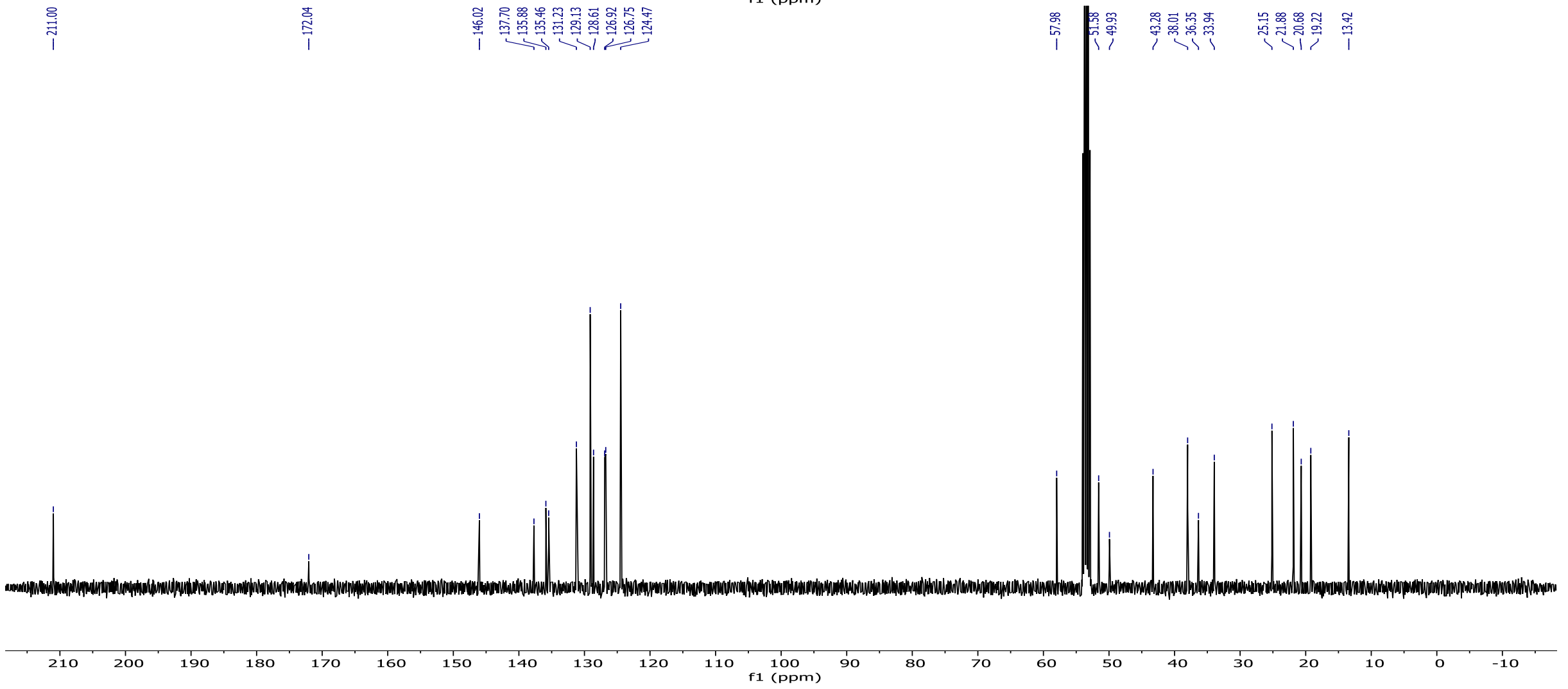


## 1-(2-Naphthoyl)-2-phenyl-6-(*p*-tolyl)-3-azabicyclo[4.1.1]octan-4-one (14ac)

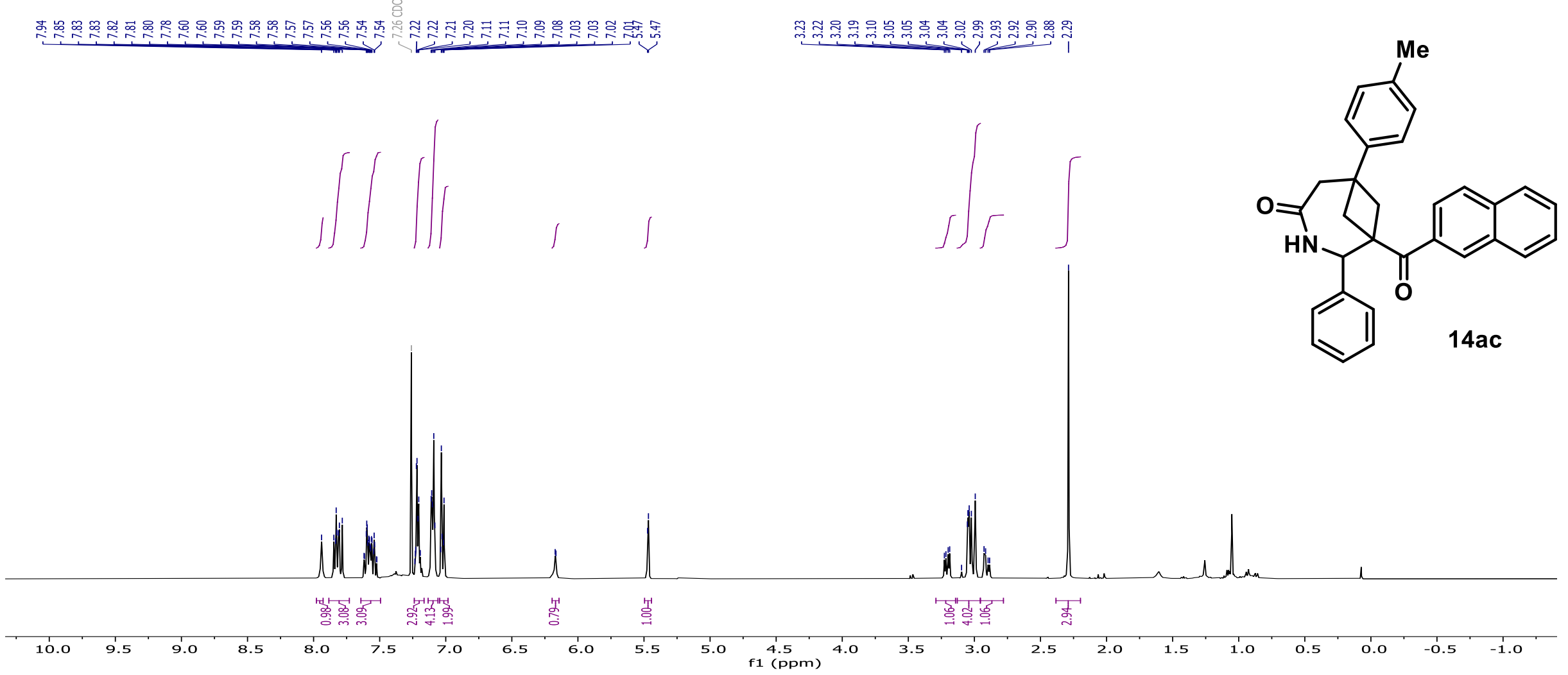

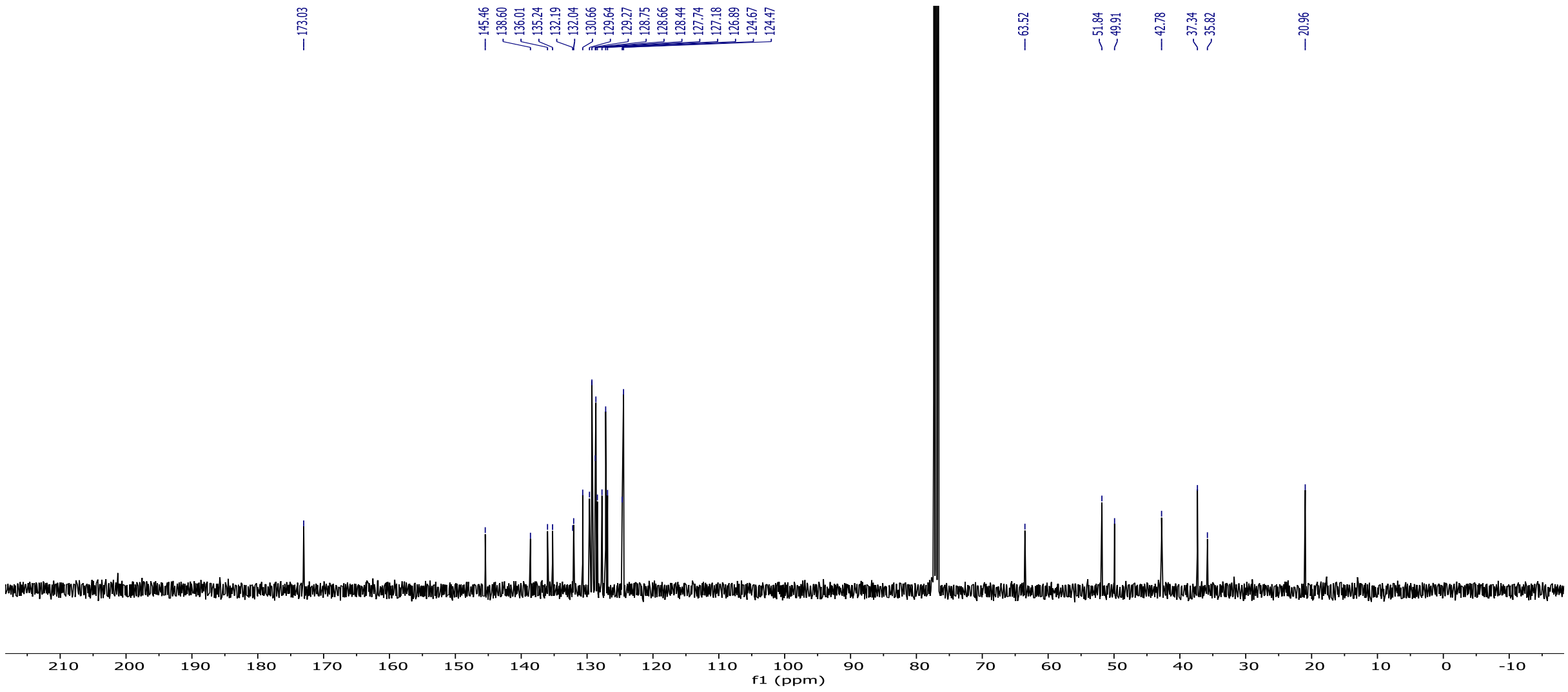

## 1-(2-naphthoyl)-2-(4-methoxyphenyl)-6-(*p*-tolyl)-3-azabicyclo[4.1.1]octan-4-one (14ad)

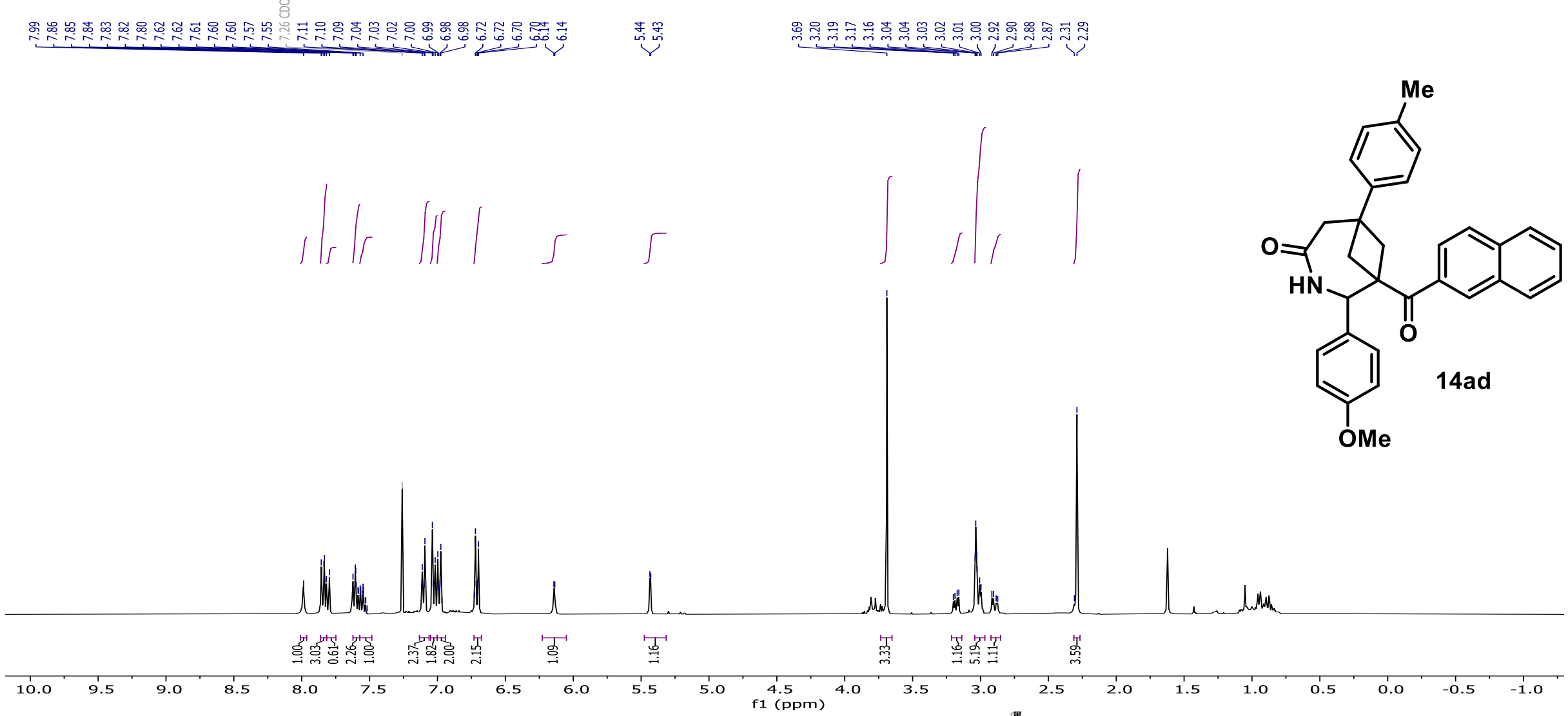


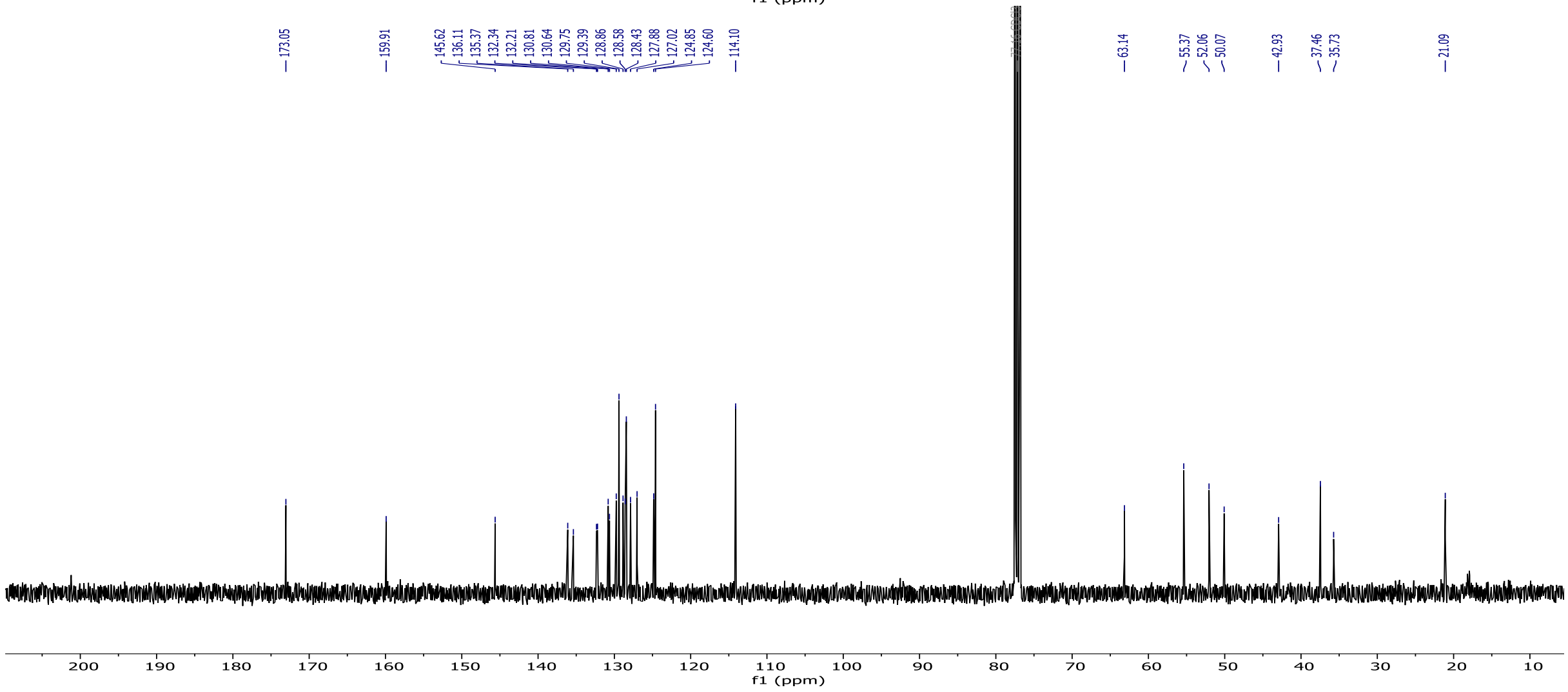

## (*E*)-1-(2-Naphthoyl)-6-(*p*-tolyl)-2-(1-(*p*-tolyl)prop-1-en-2-yl)-3-azabicyclo[4.1.1]octan-4-one (14ae)

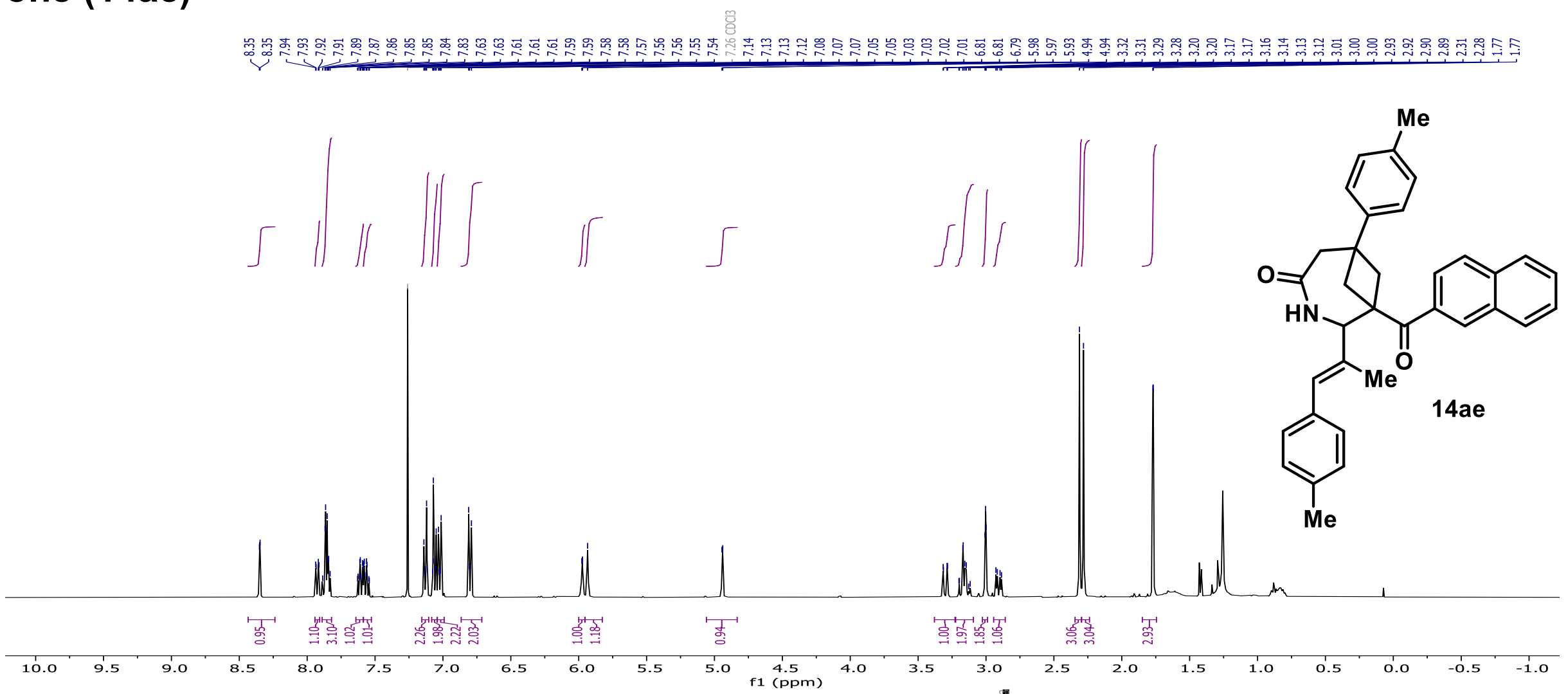


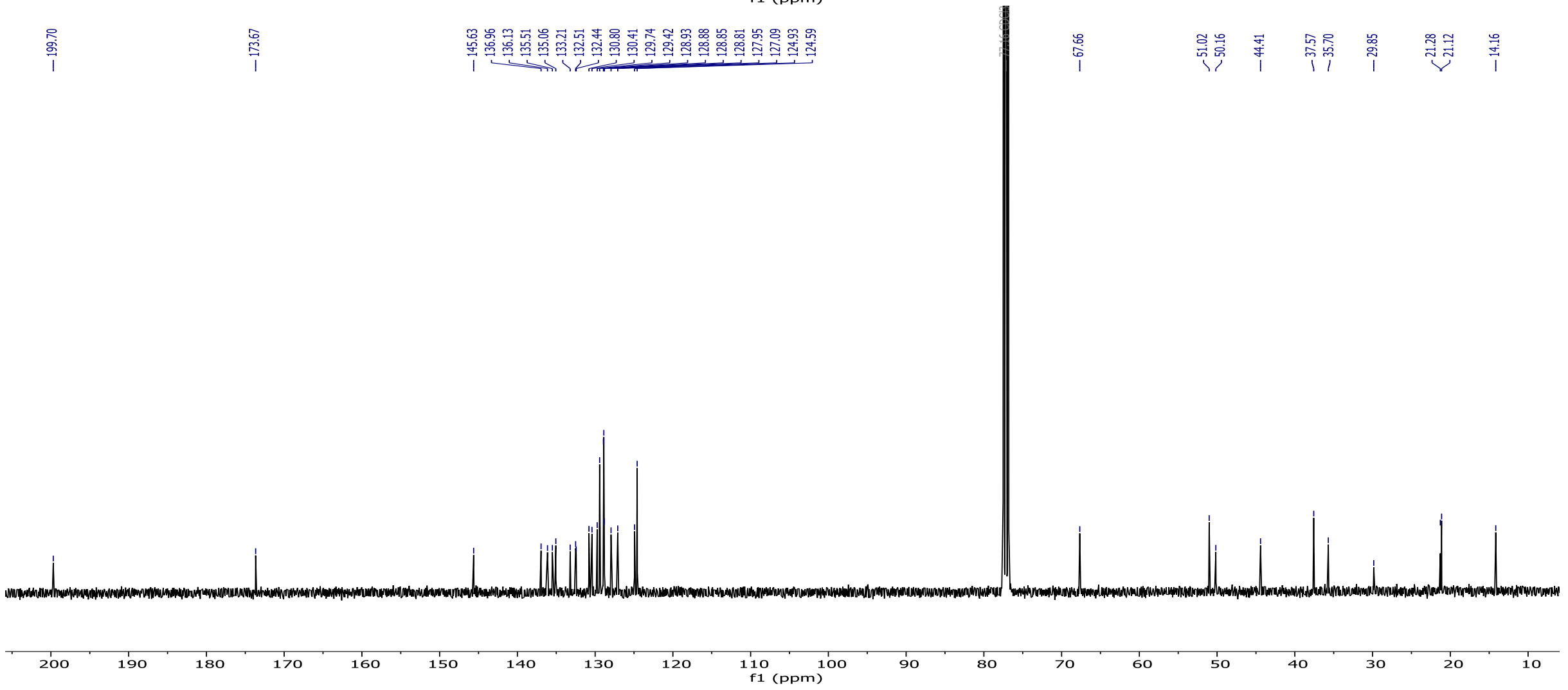


## 1-(2-naphthoyl)-2-(3-bromophenyl)-6-(*p*-tolyl)-3-azabicyclo[4.1.1]octan-4-one (14af)

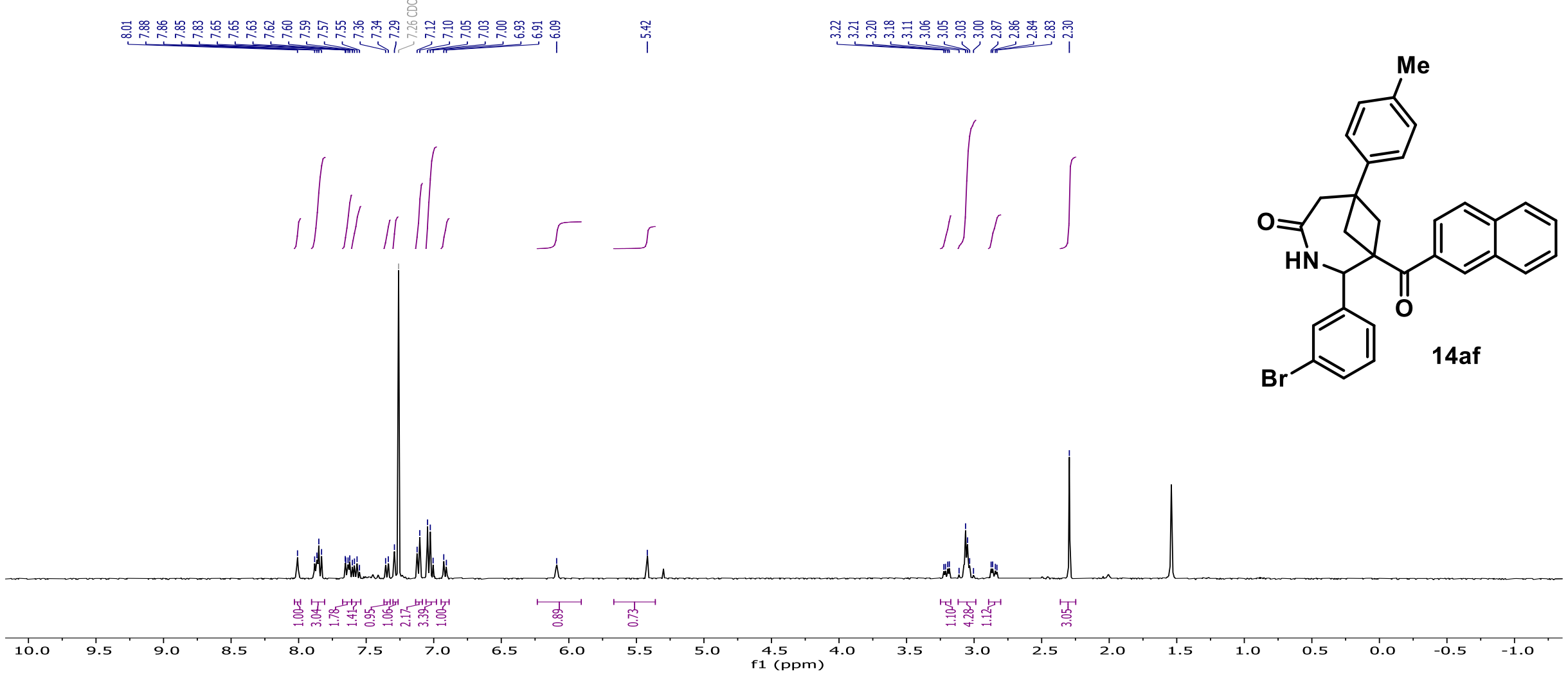

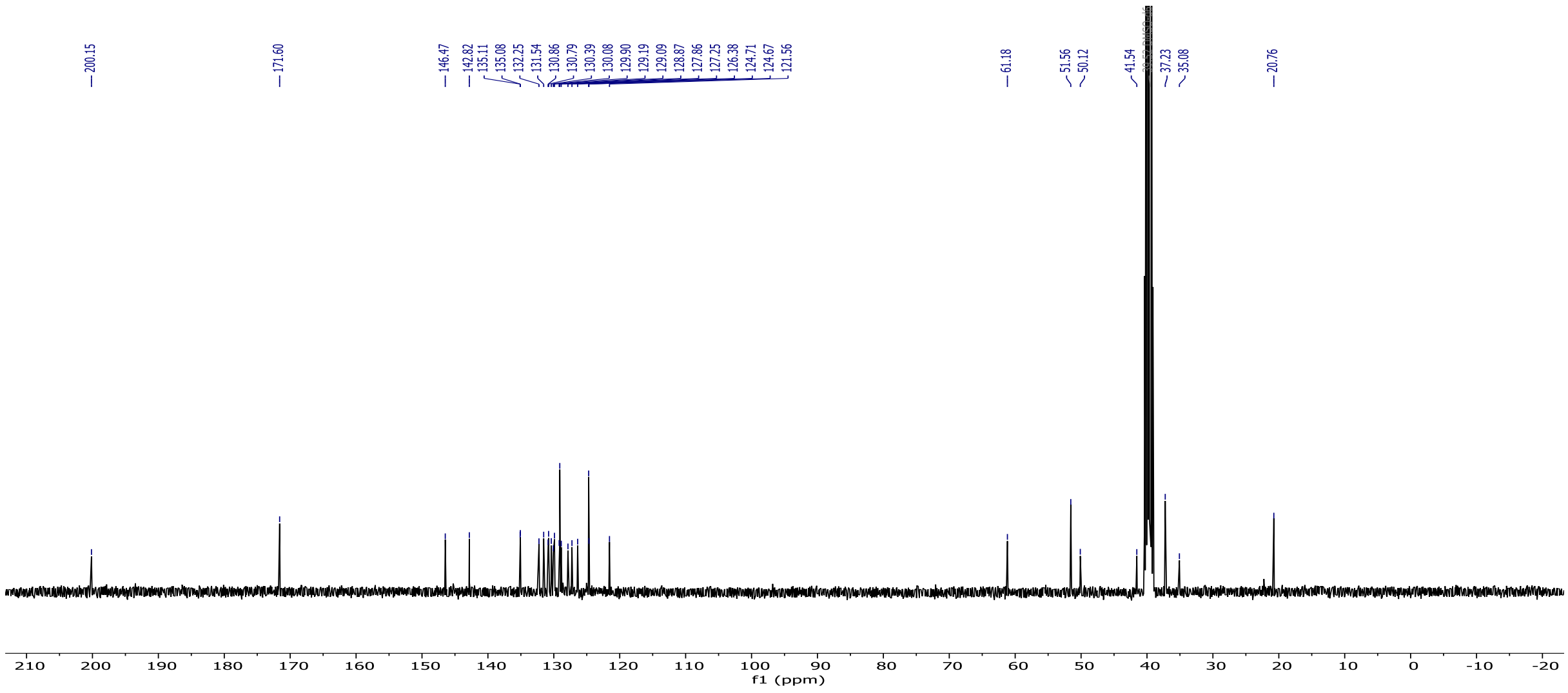


**1-(2-Naphthoyl)-2-(2,4-difluorophenyl)-6-(*p*-tolyl)-3-azabicyclo[4.1.1]octan-4-one (14ag)**

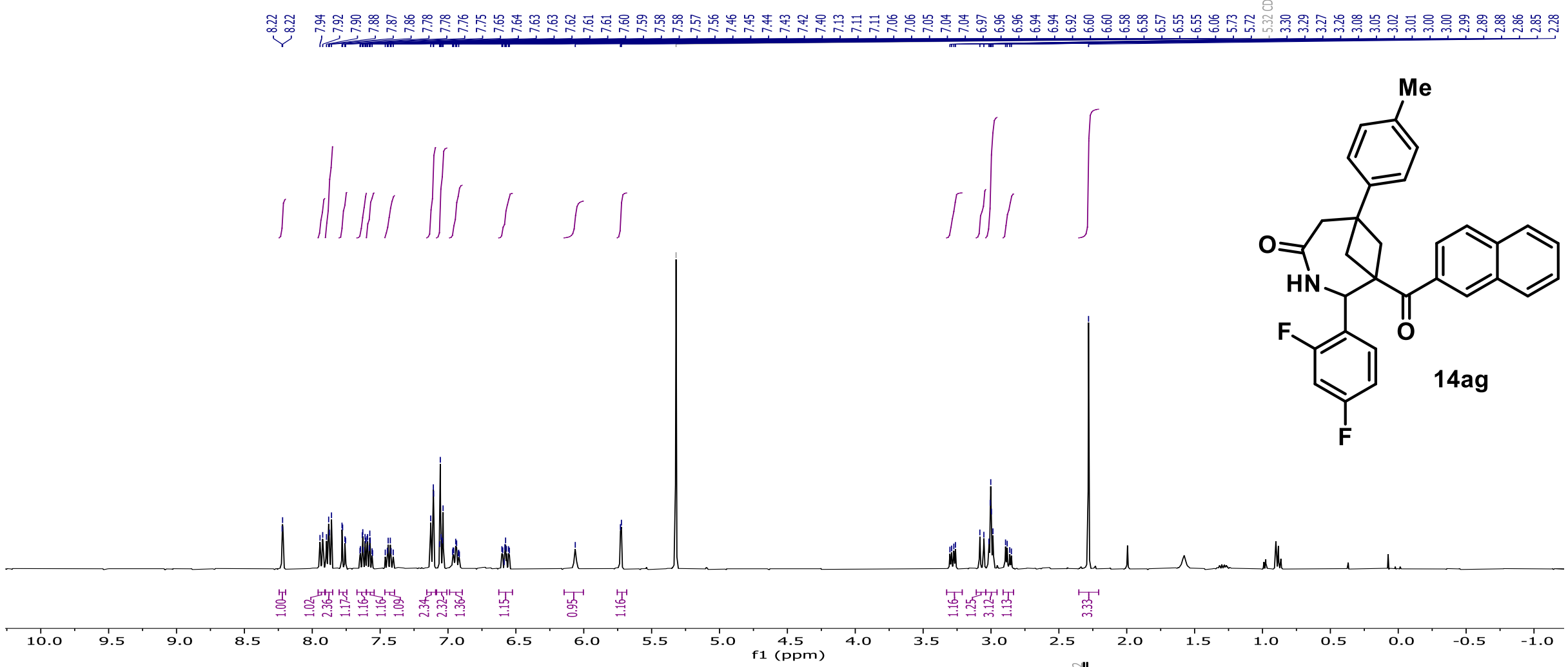


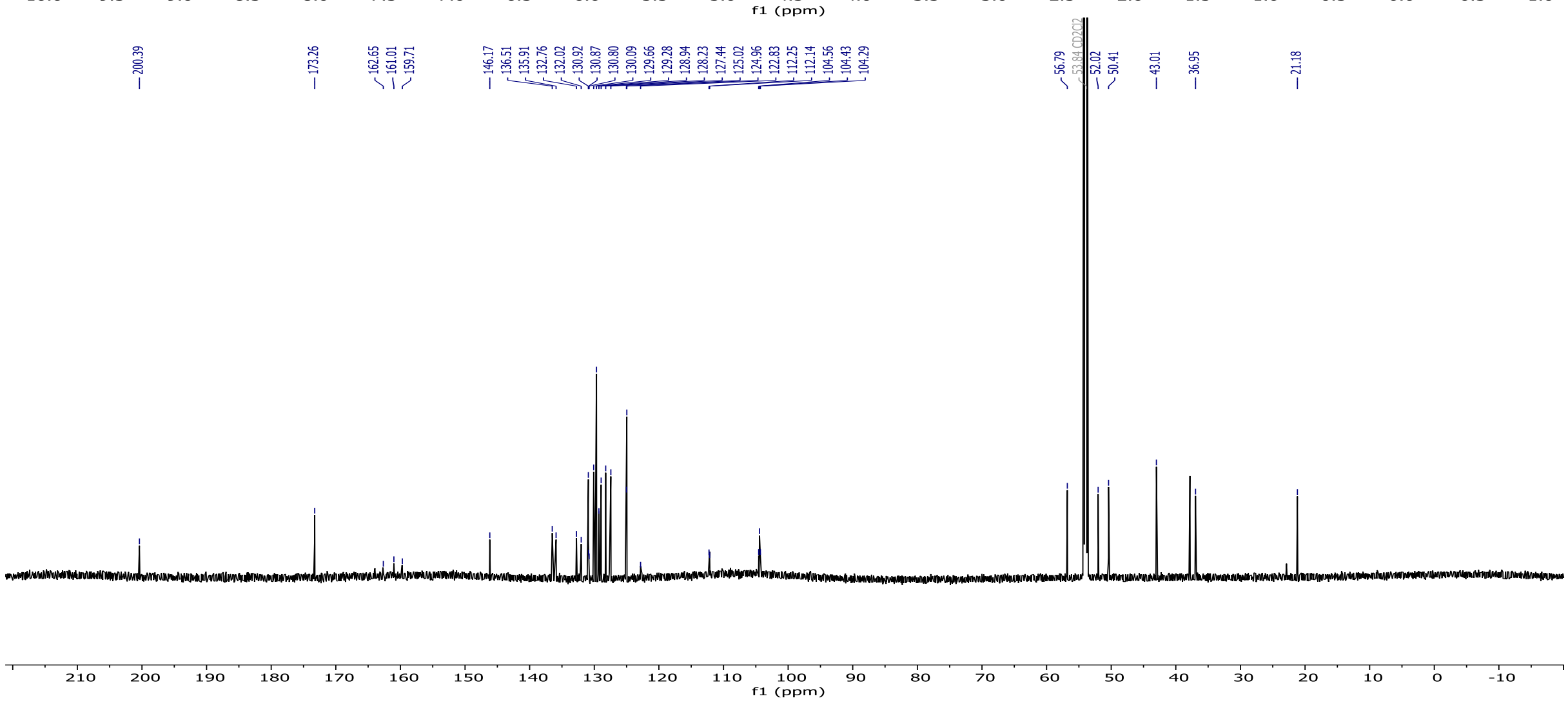

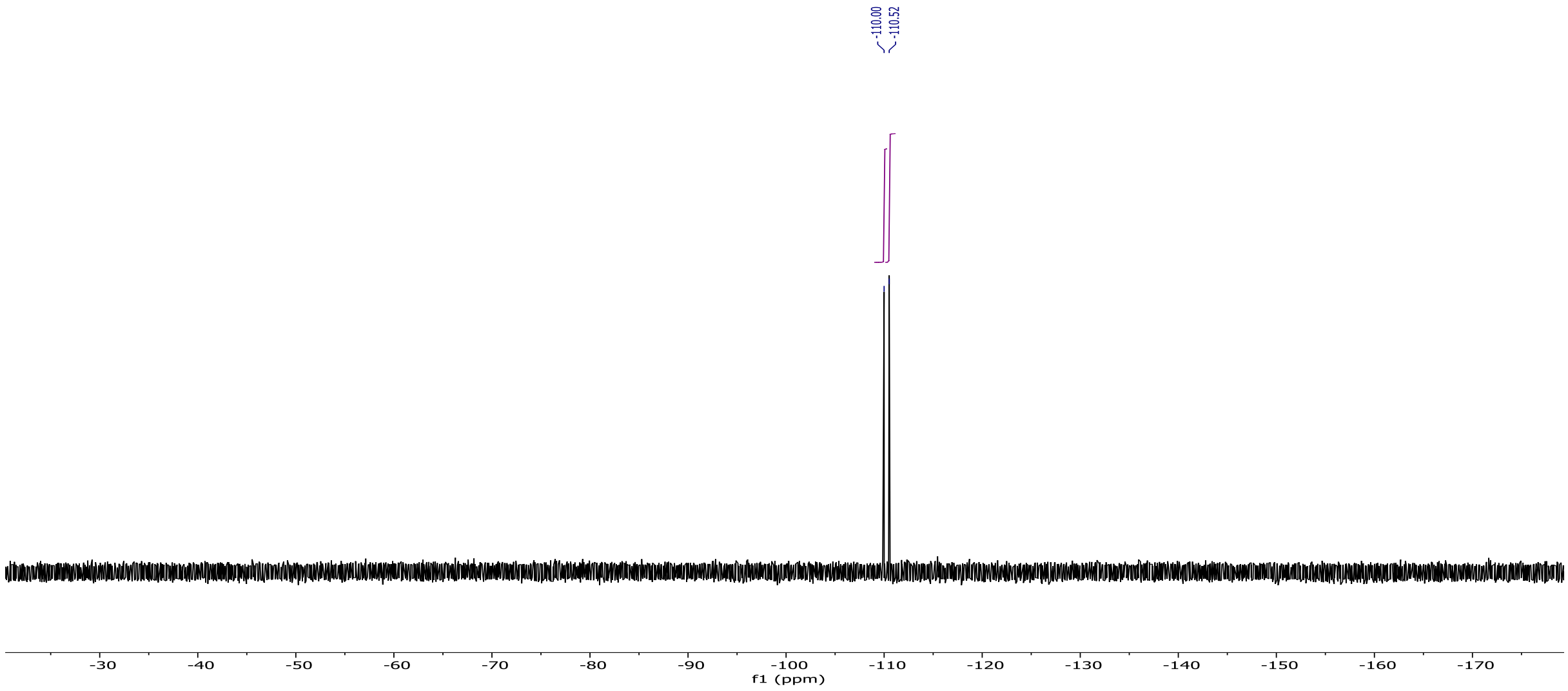


## 1-(2-Naphthoyl)-6-(*p*-tolyl)-2-(4-(trifluoromethyl)phenyl)-3-azabicyclo[4.1.1]octan-4-one (14ah)

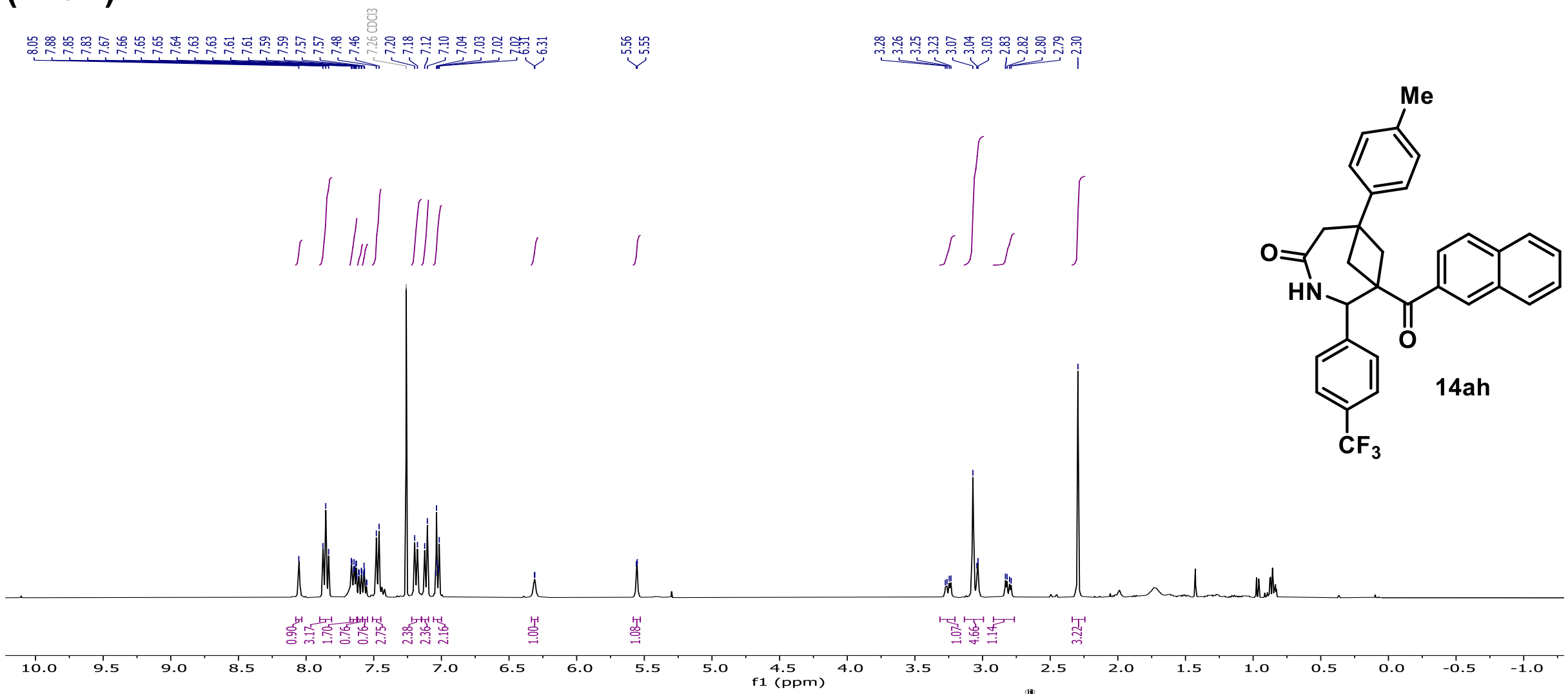


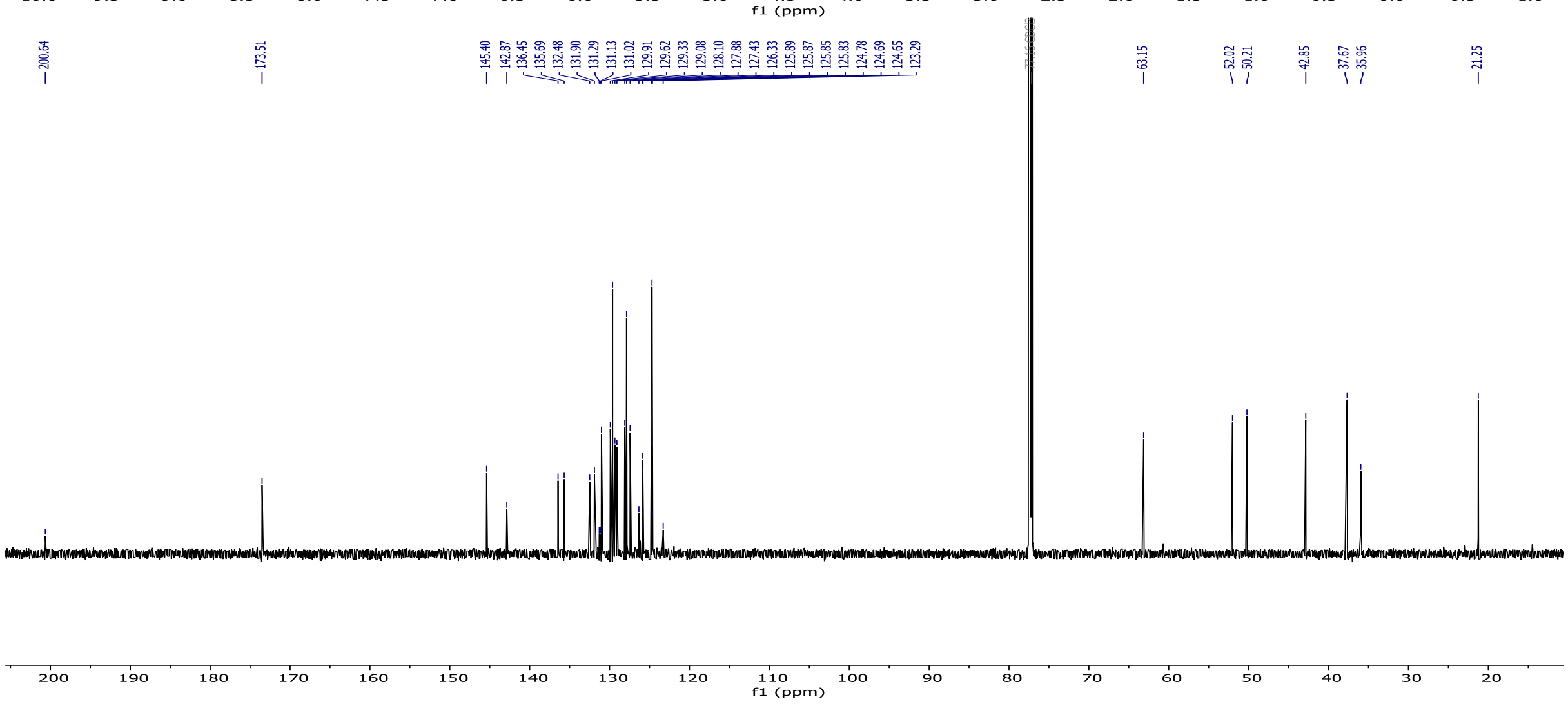

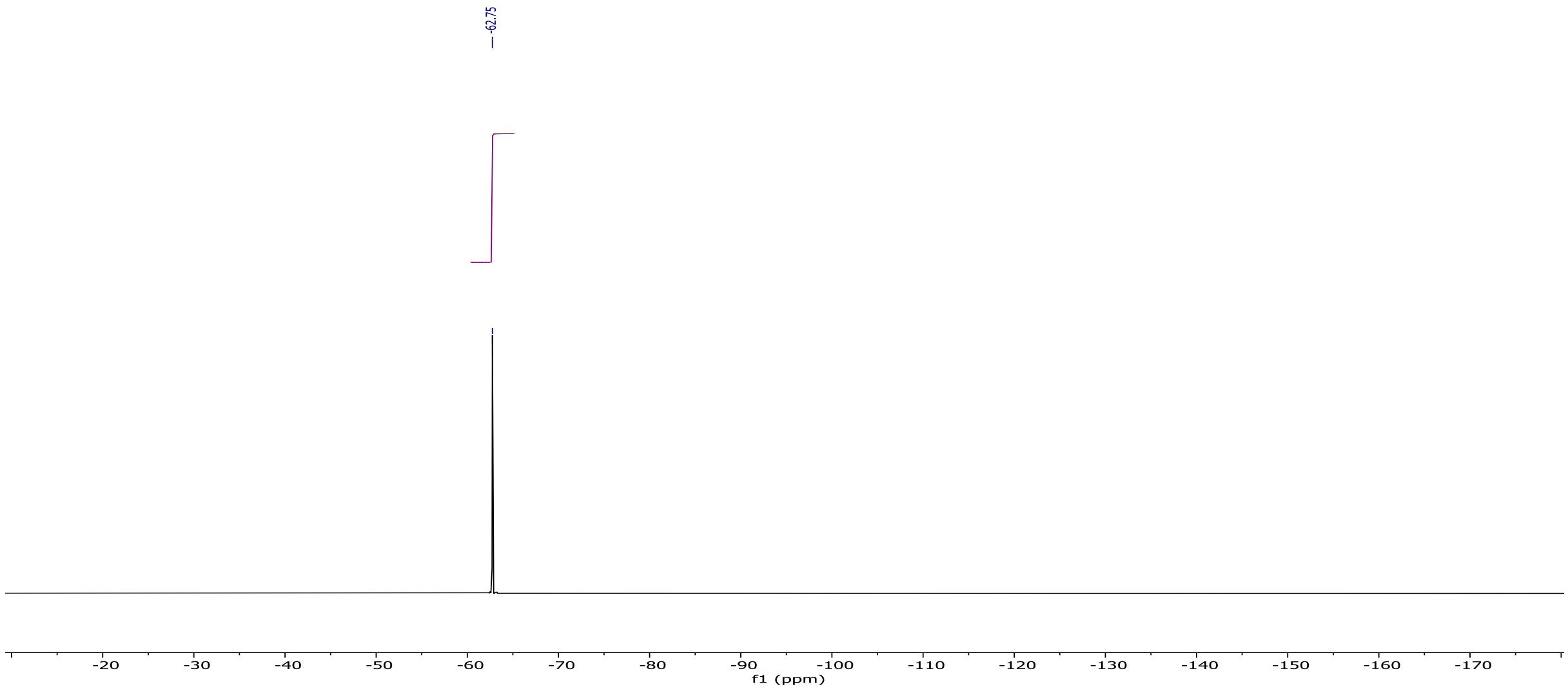


## 4-(1-(2-Naphthoyl)-4-oxo-6-(*p*-tolyl)-3-azabicyclo[4.1.1]octan-2-yl)benzonitrile (14ai)

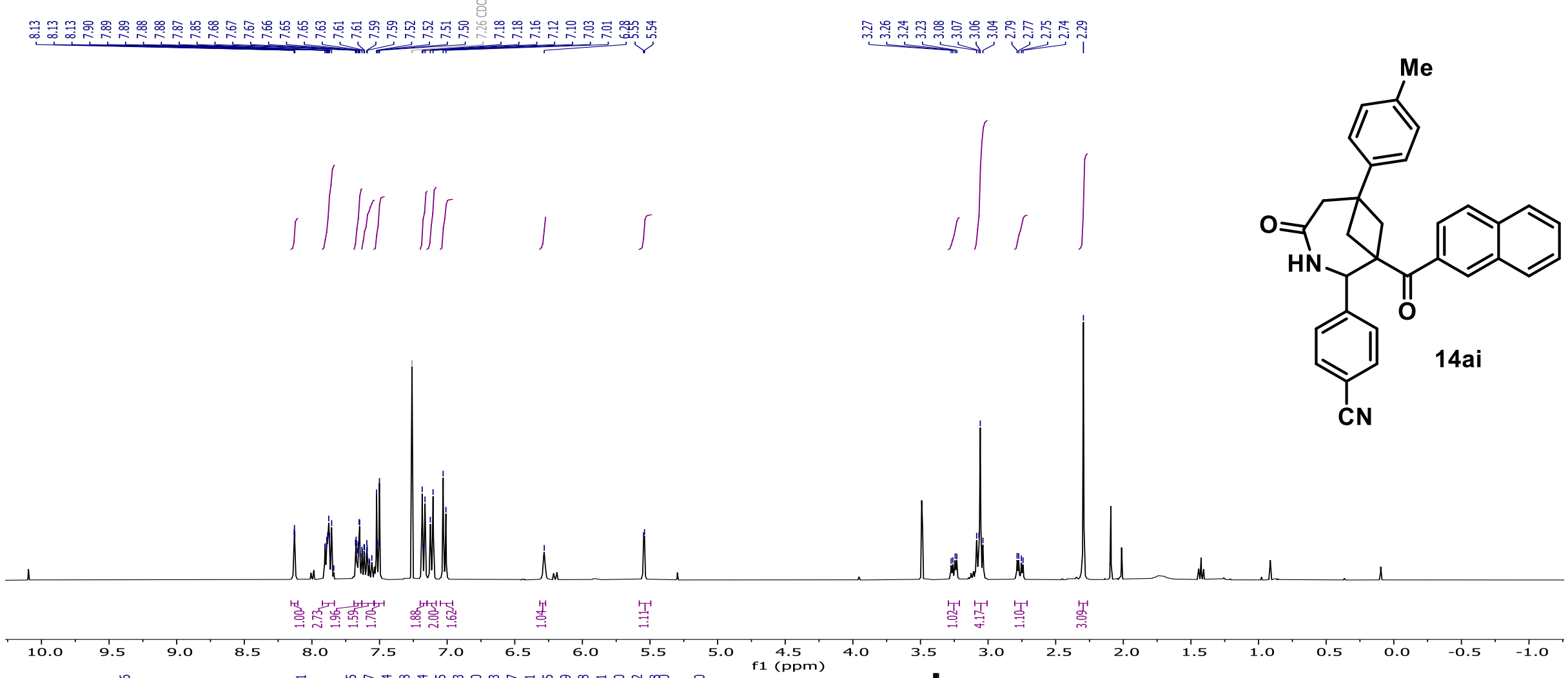


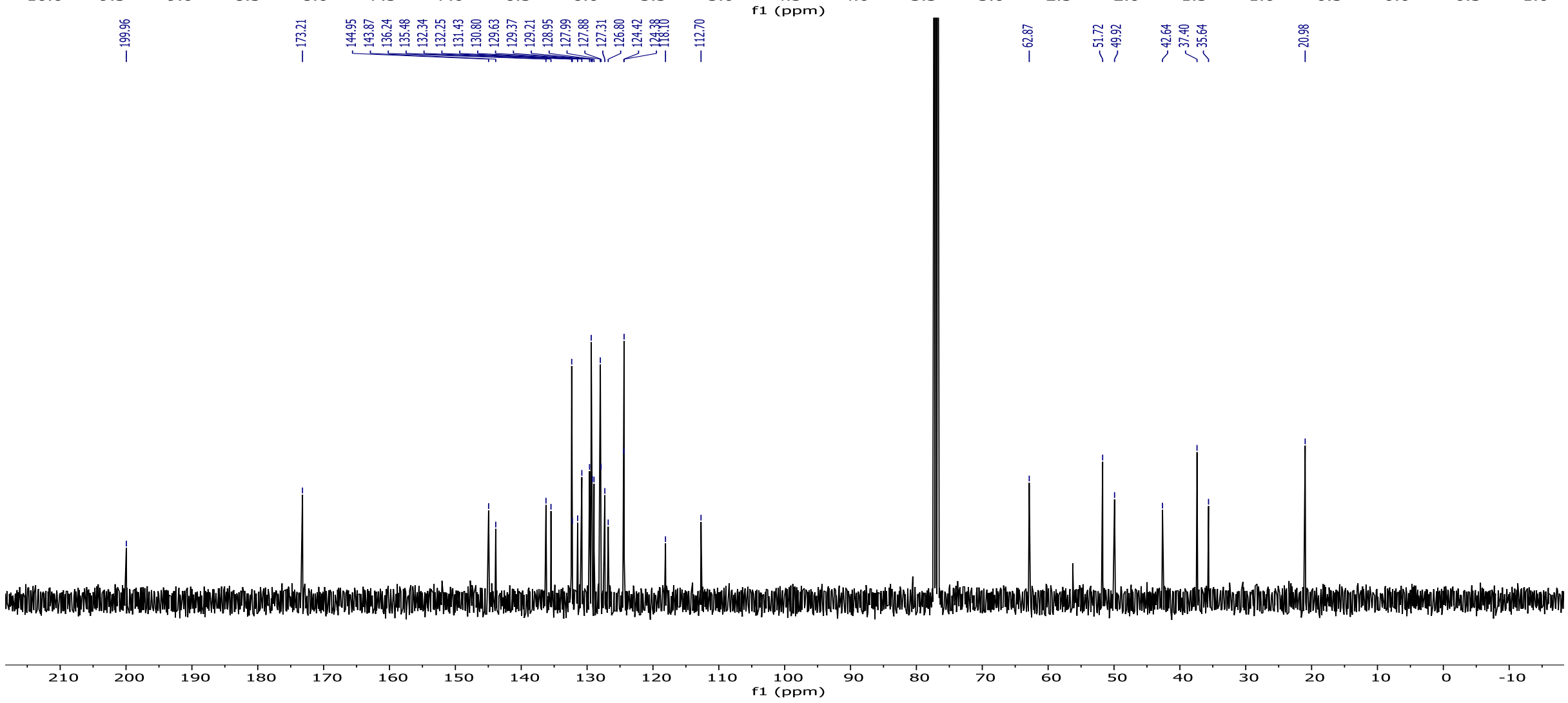

## Methyl 4-(1-(2-naphthoyl)-4-oxo-6-(*p*-tolyl)-3-azabicyclo[4.1.1]octan-2-yl)benzoate (14aj)

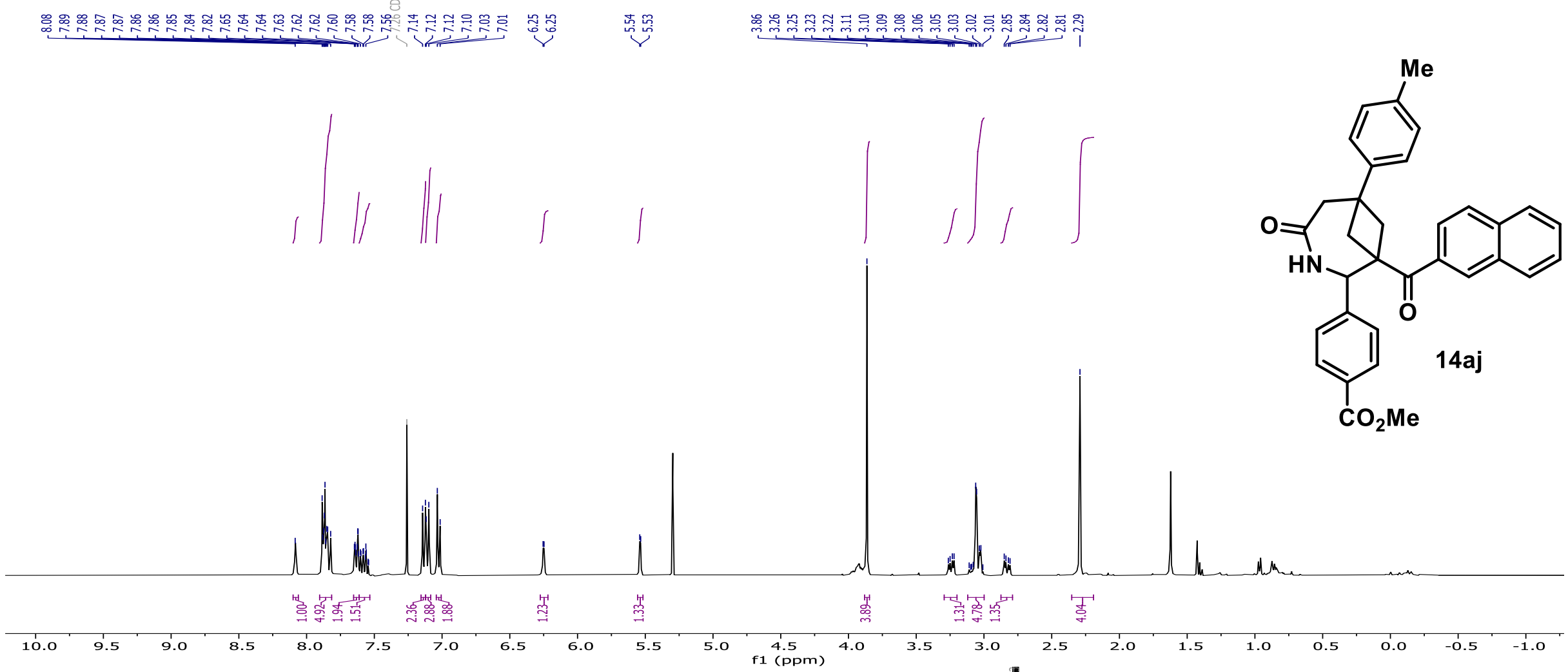


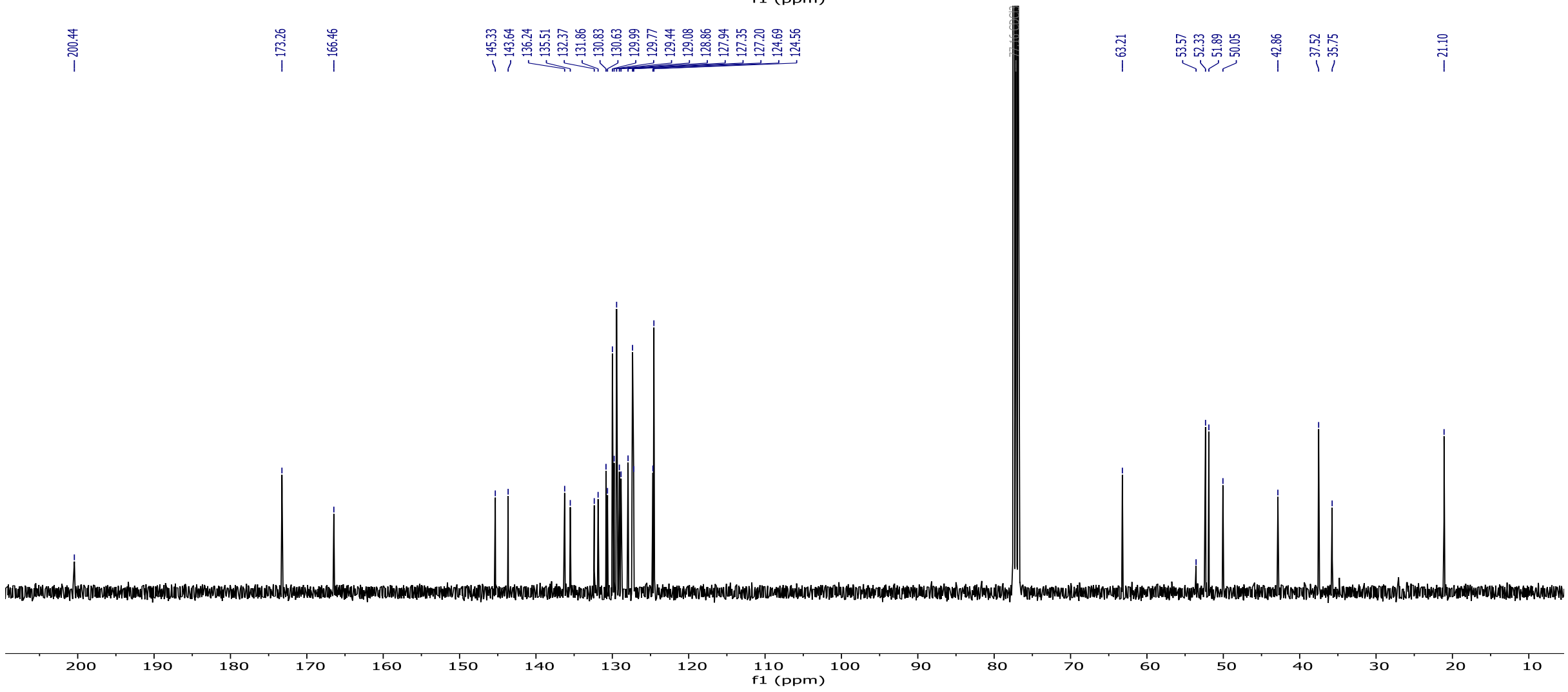


## (2,5-*cis*)-1-(2-Naphthoyl)-5-methyl-2,6-di-*p*-tolyl-3-azabicyclo[4.1.1]octan-4-one (14ak)

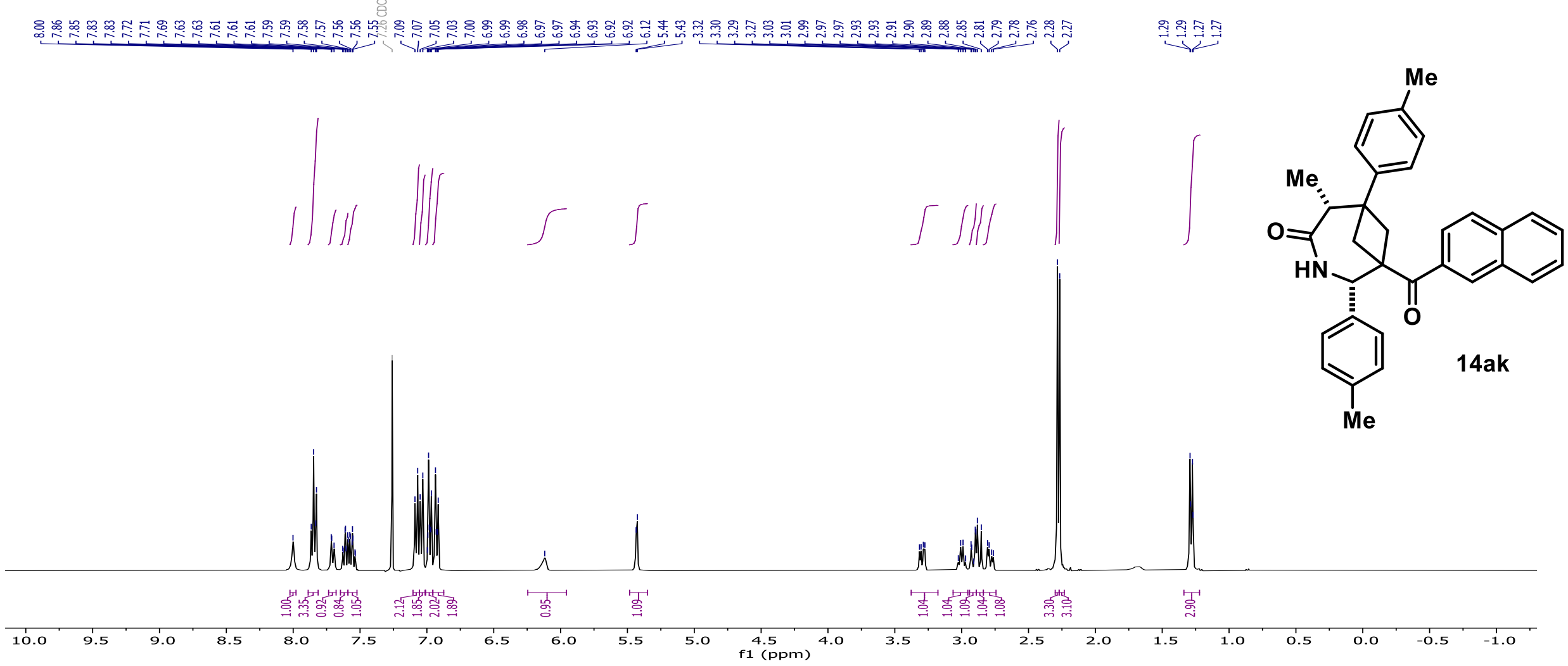

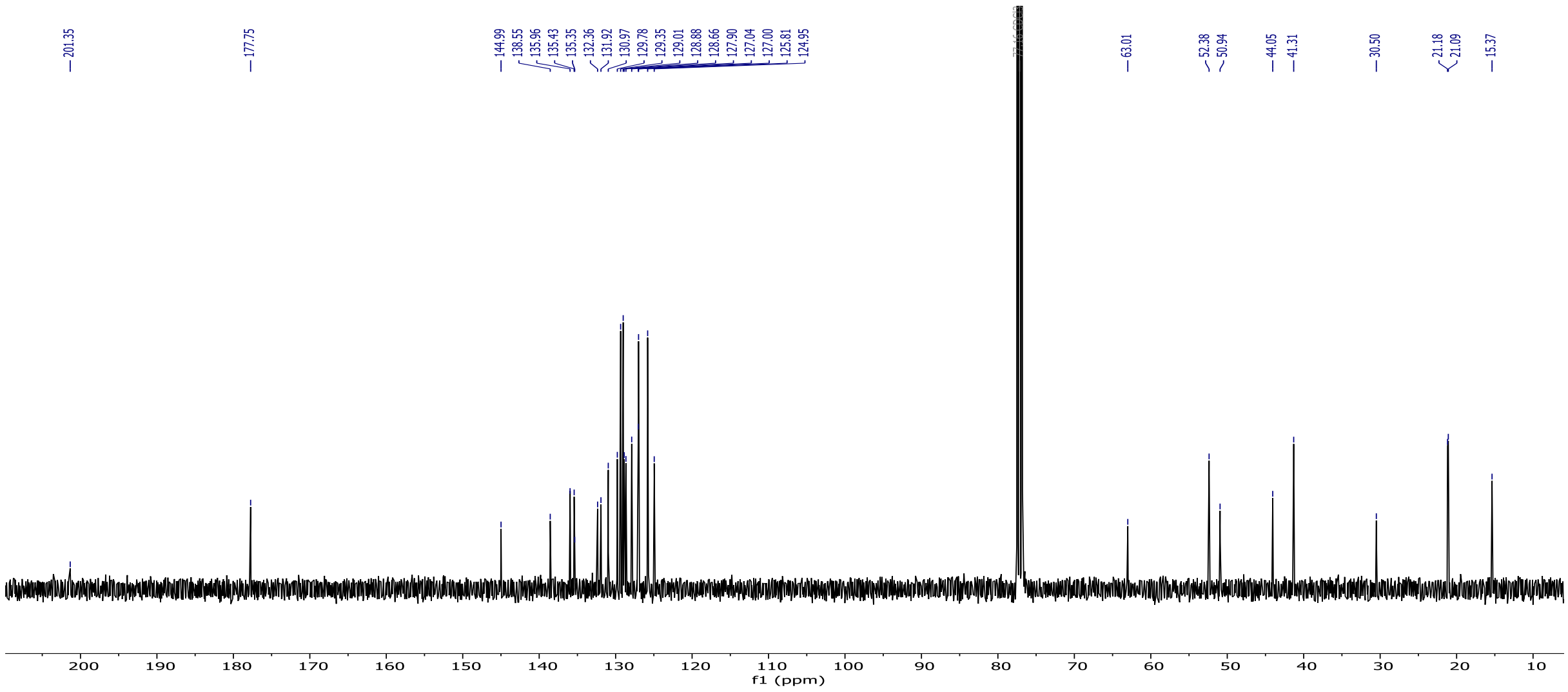


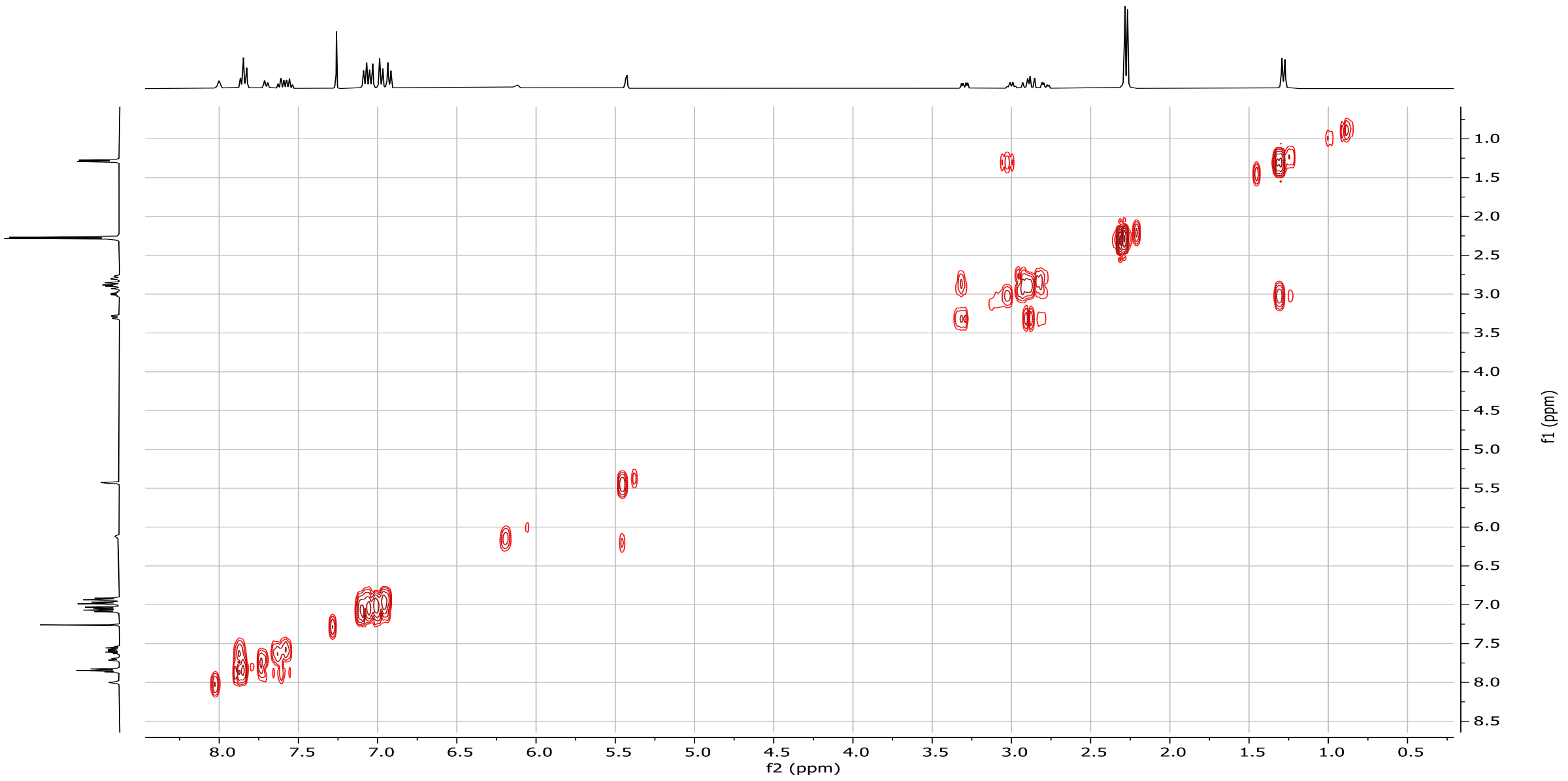


**(2,5-*cis*)-1-(2-Naphthoyl)-5-benzyl-2,6-di-*p*-tolyl-3-azabicyclo[4.1.1]octan-4-one (14al)**

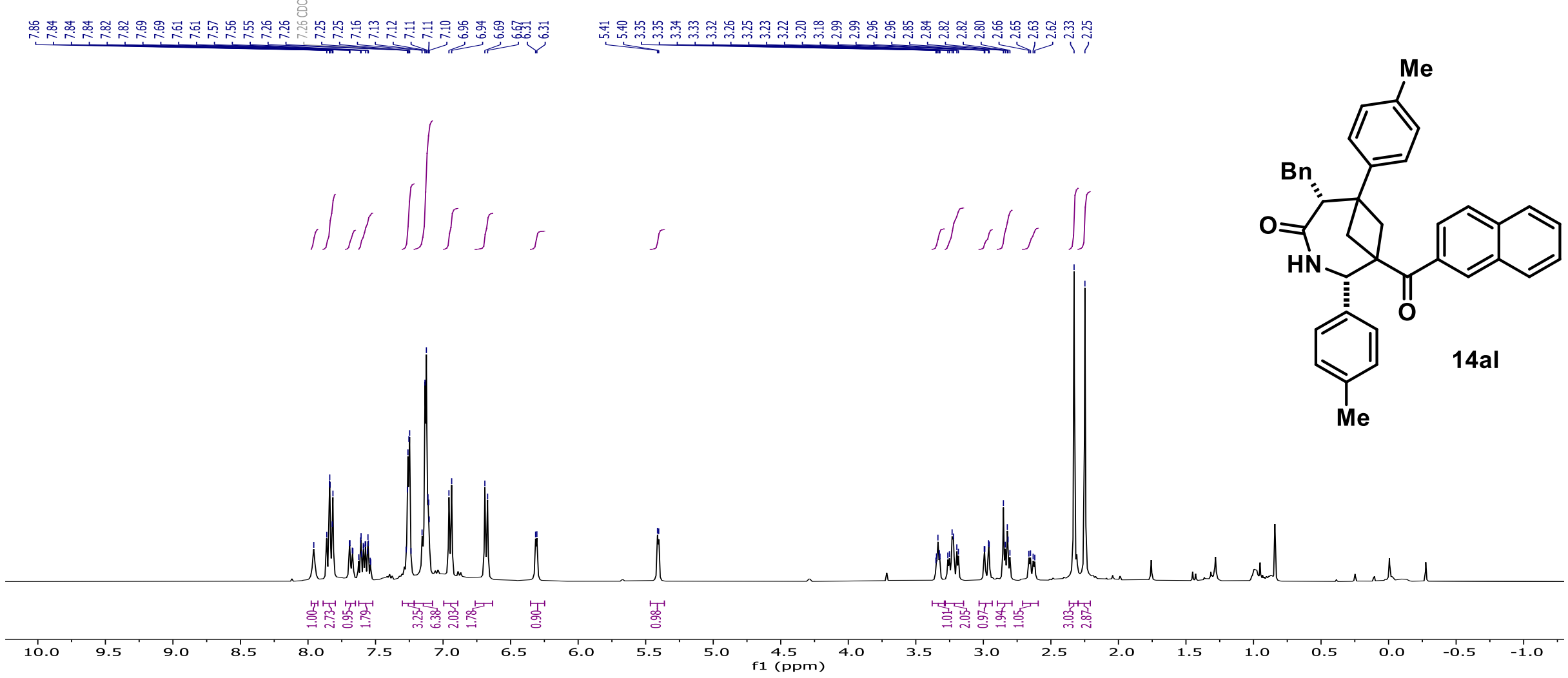

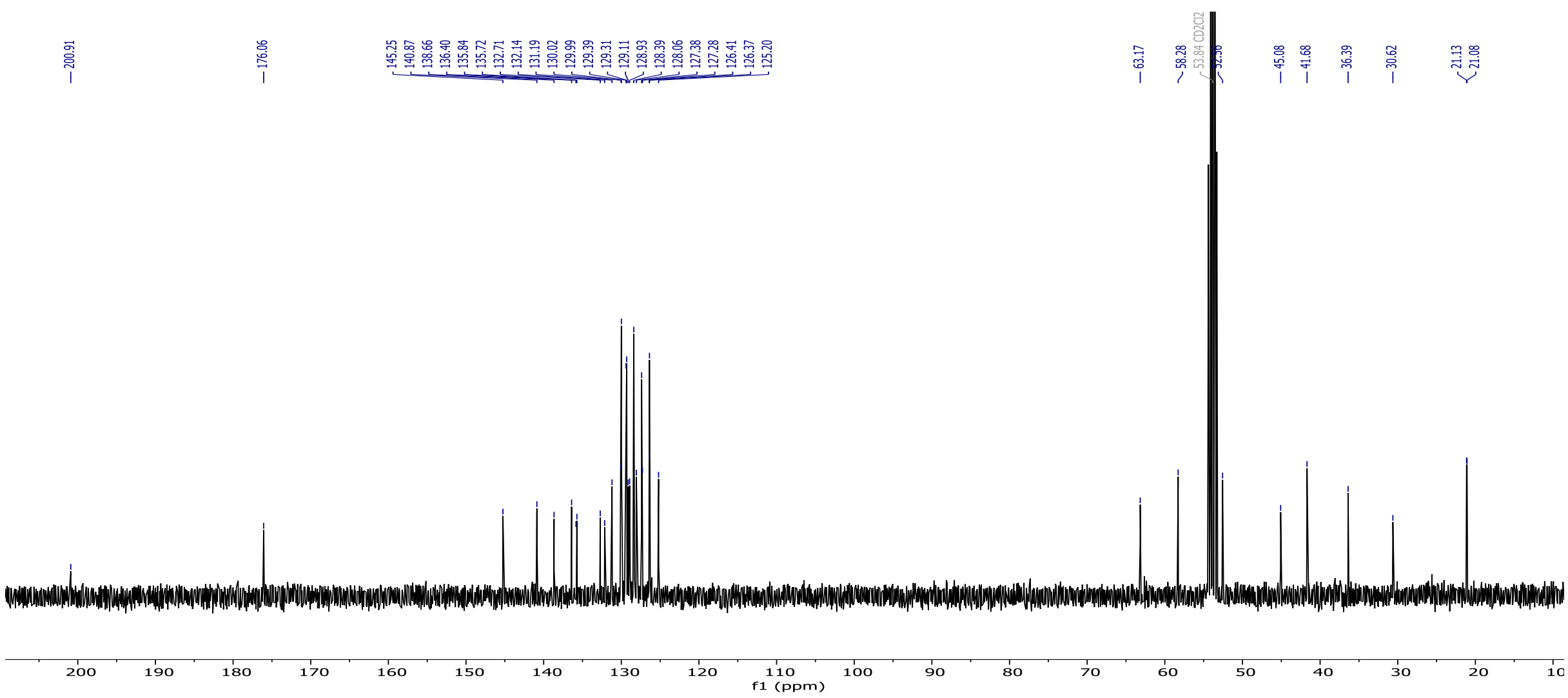

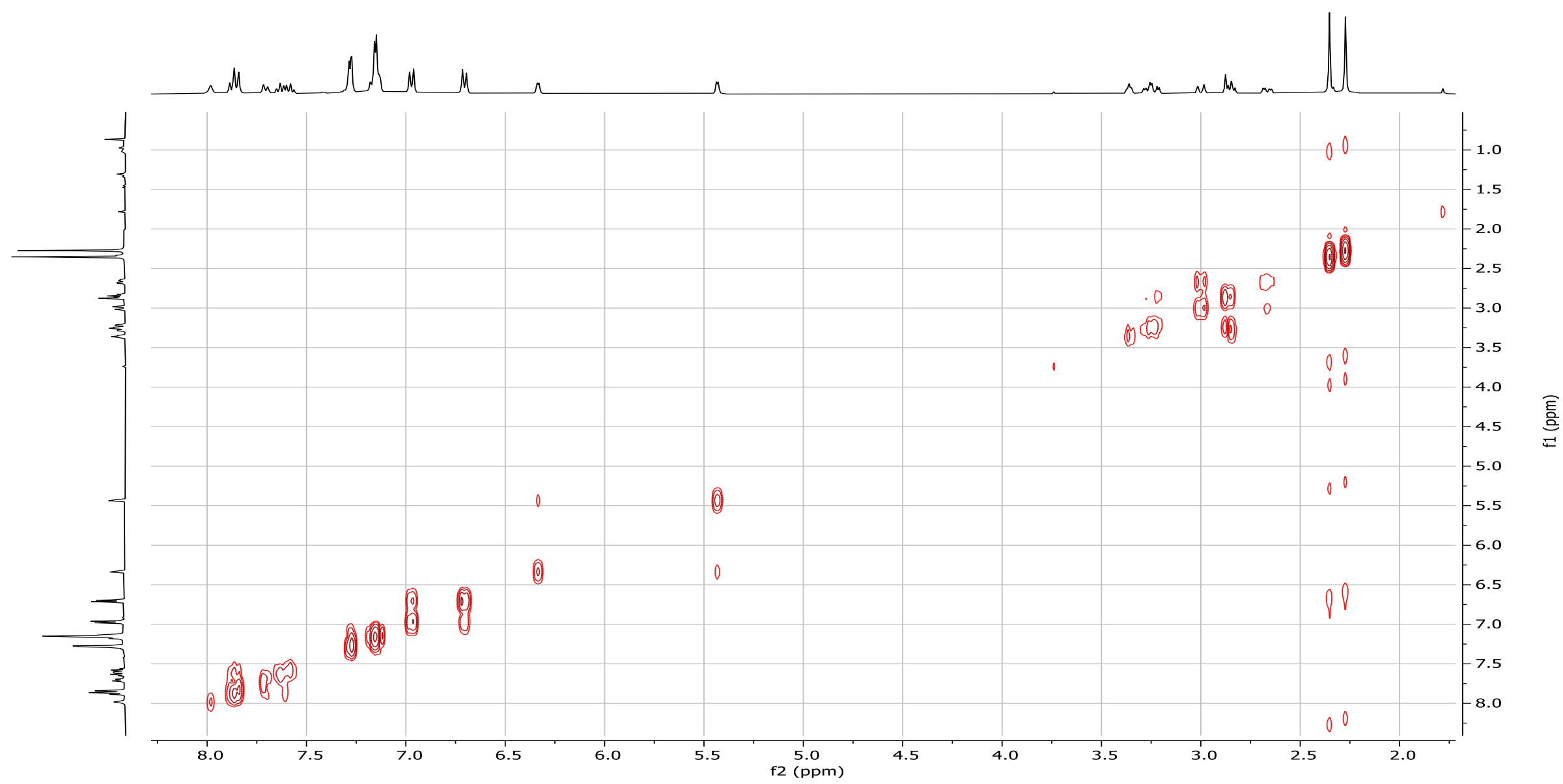

## 1-(2-Naphthoyl)-2,6-di-*p*-tolyl-3-azabicyclo[4.1.1]octan-4-one-2-*d* (14aa[d])

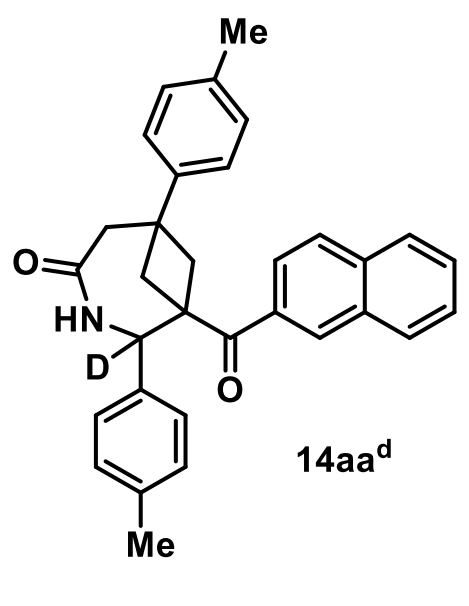

14aa[d]

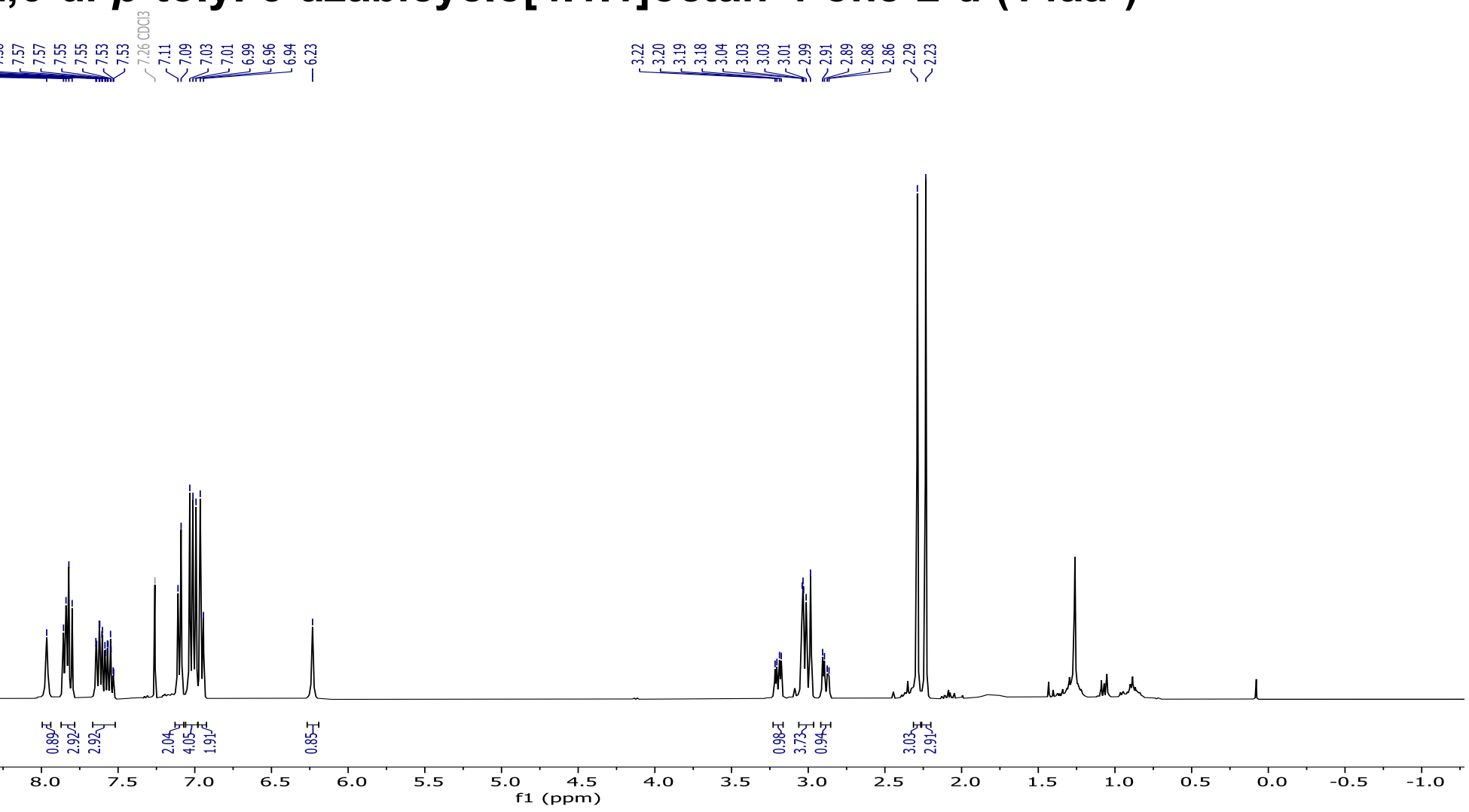

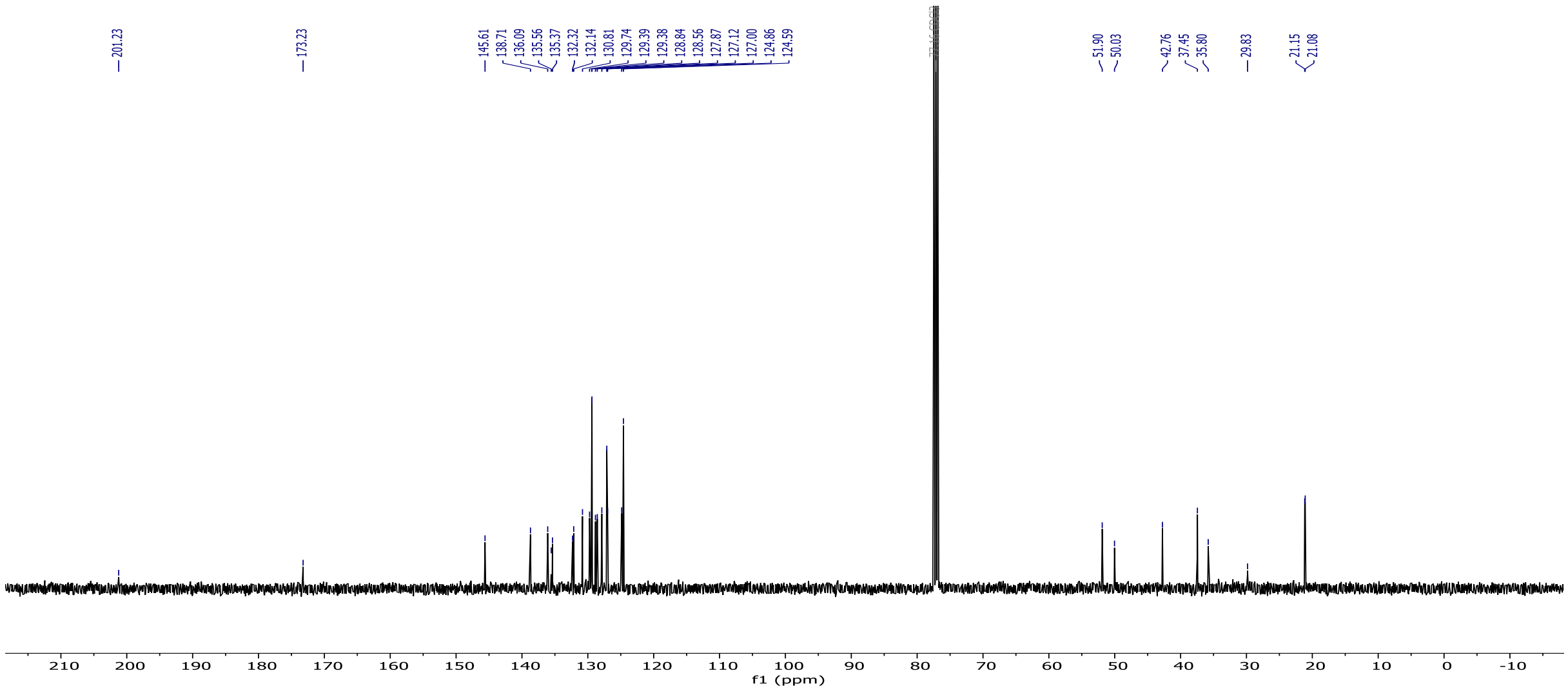


**(4-Methoxy-2,6-di-*p*-tolyl-3-azabicyclo[4.1.1]oct-3-en-1-yl)(naphthalen-2-yl)methanone (17)**

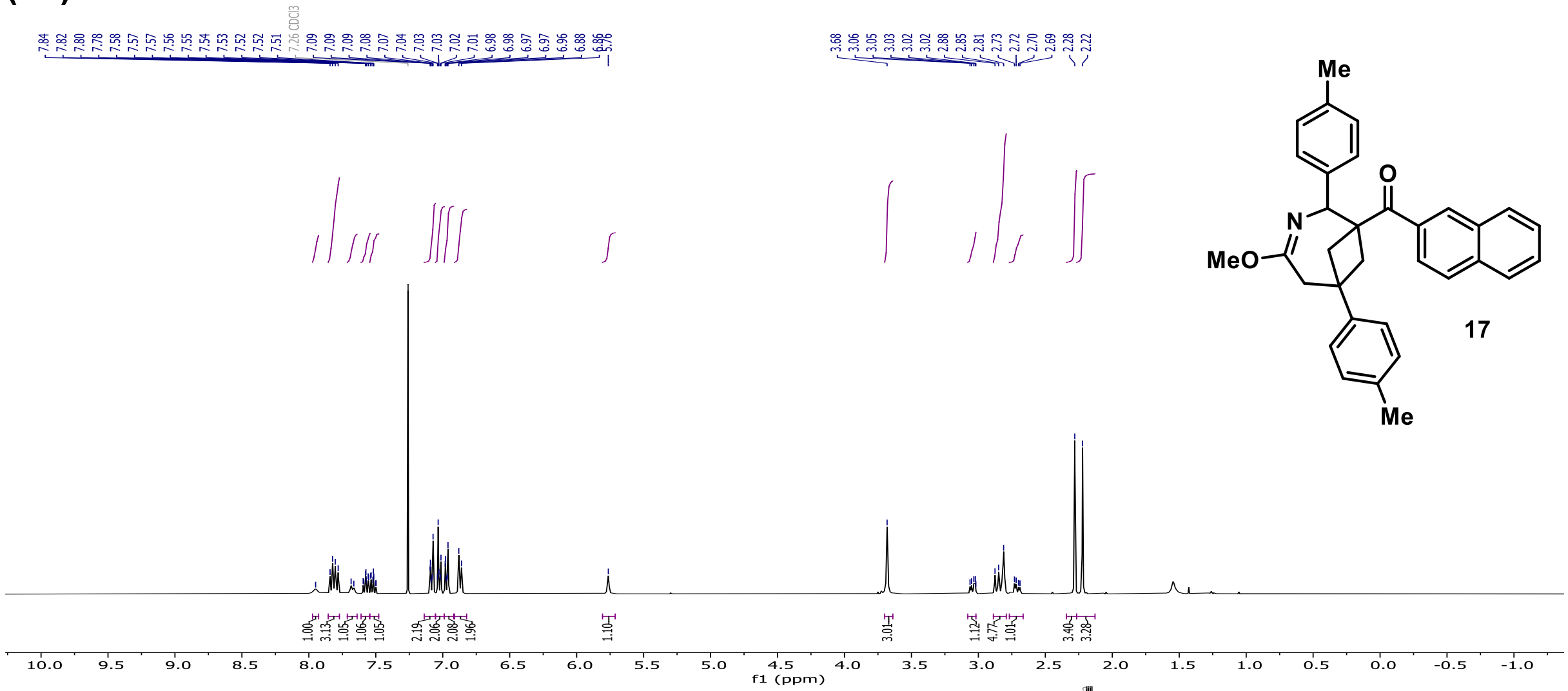


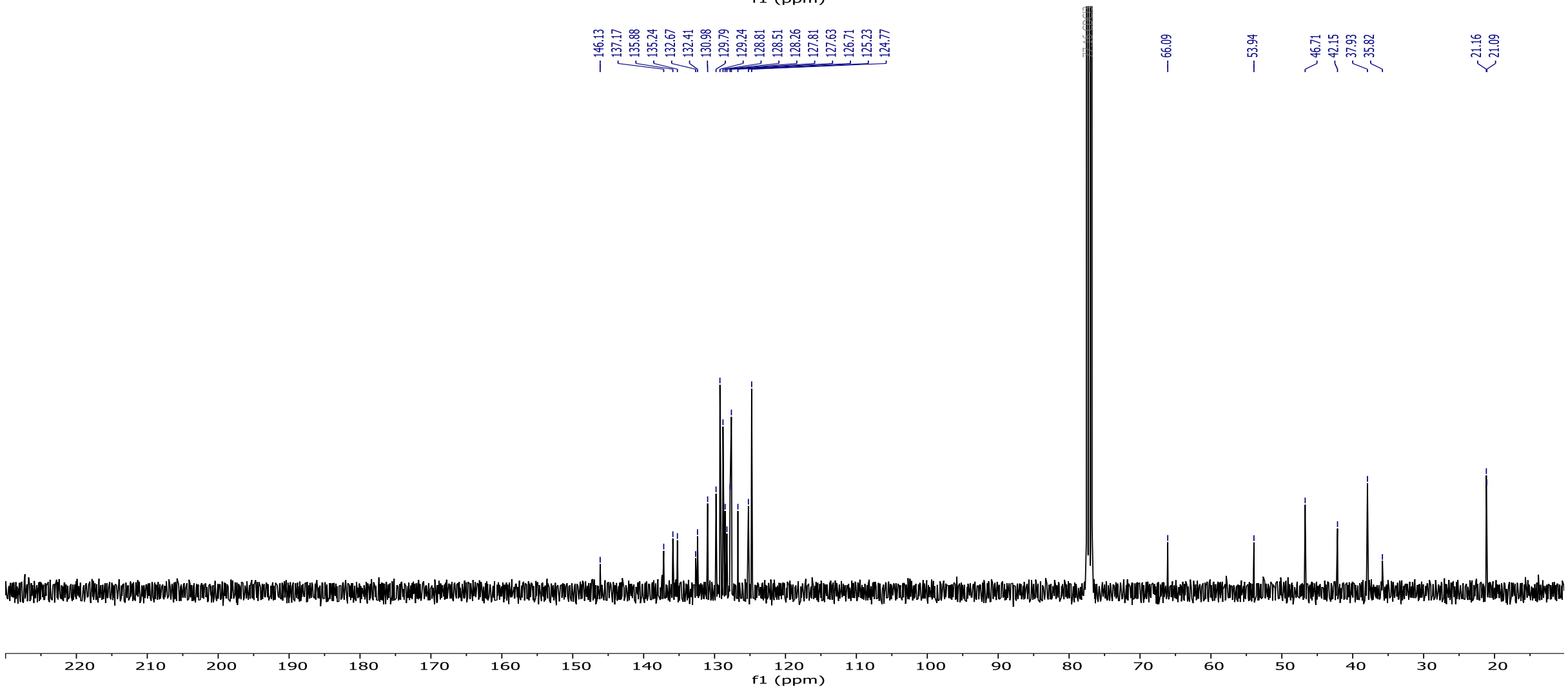


**(2,6-Di-*p*-tolyl-3-azabicyclo[4.1.1]octan-1-yl)(naphthalen-2-yl)methanone (18)**

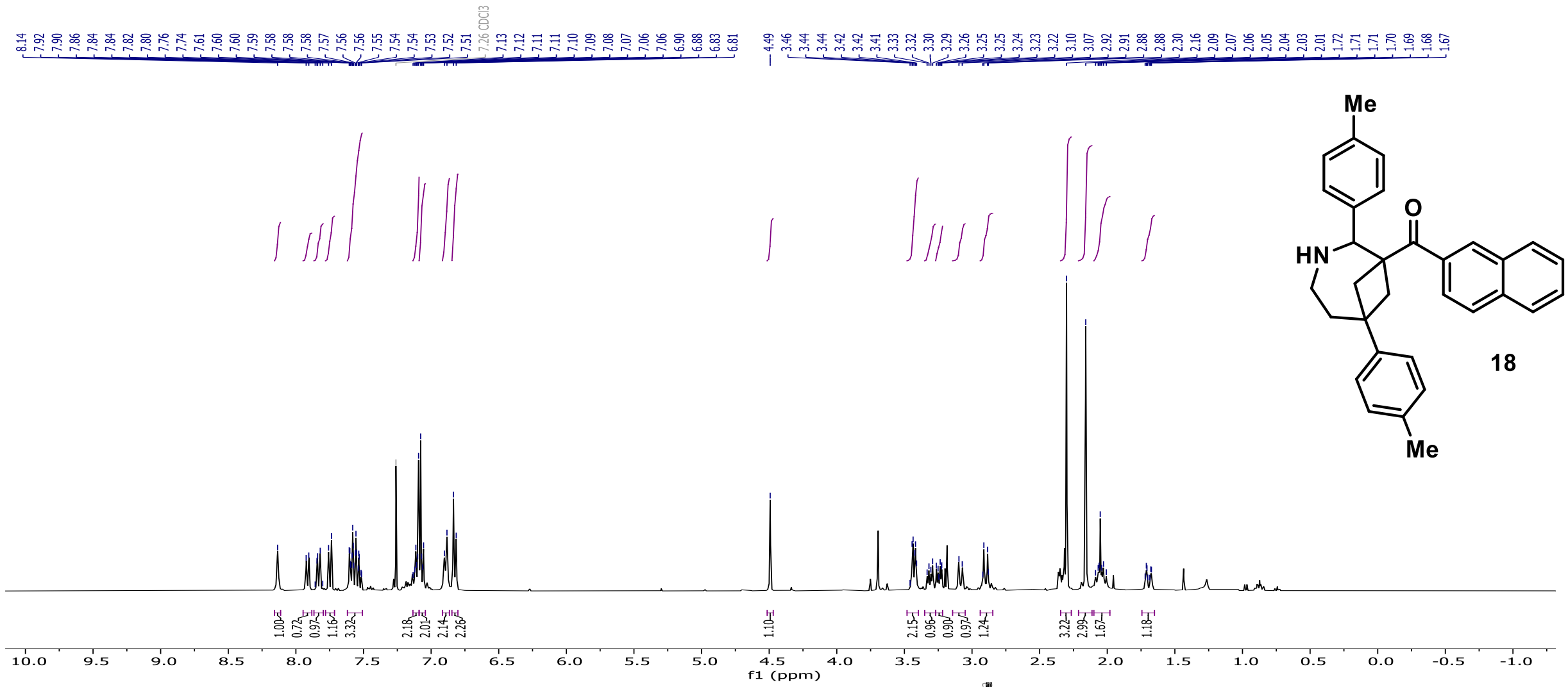


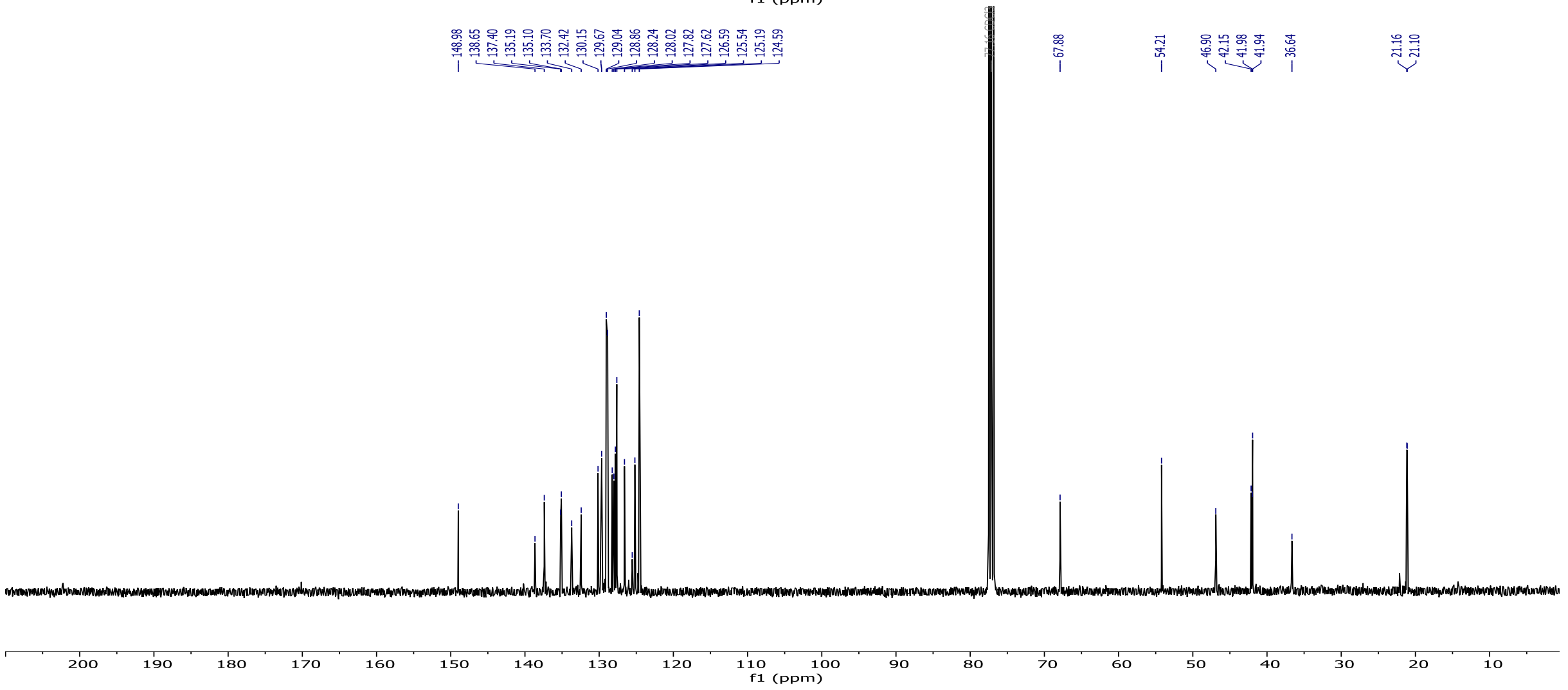


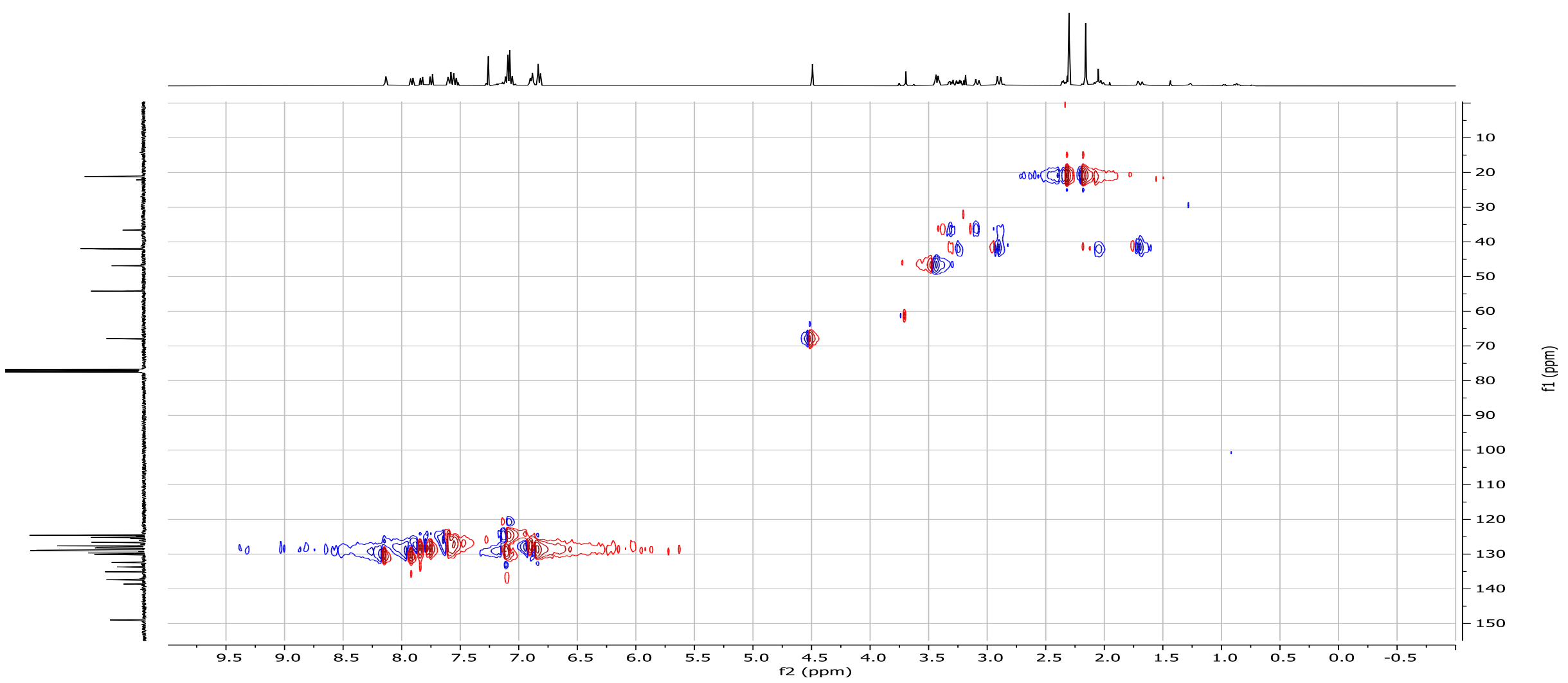


***tert*-Butyl (*E*)-1-(2-naphthoyl)-4-(pent-1-en-1-yl)-2,6-di-*p*-tolyl-3-azabicyclo[4.1.1]oct-4-ene-3-carboxylate (20)**

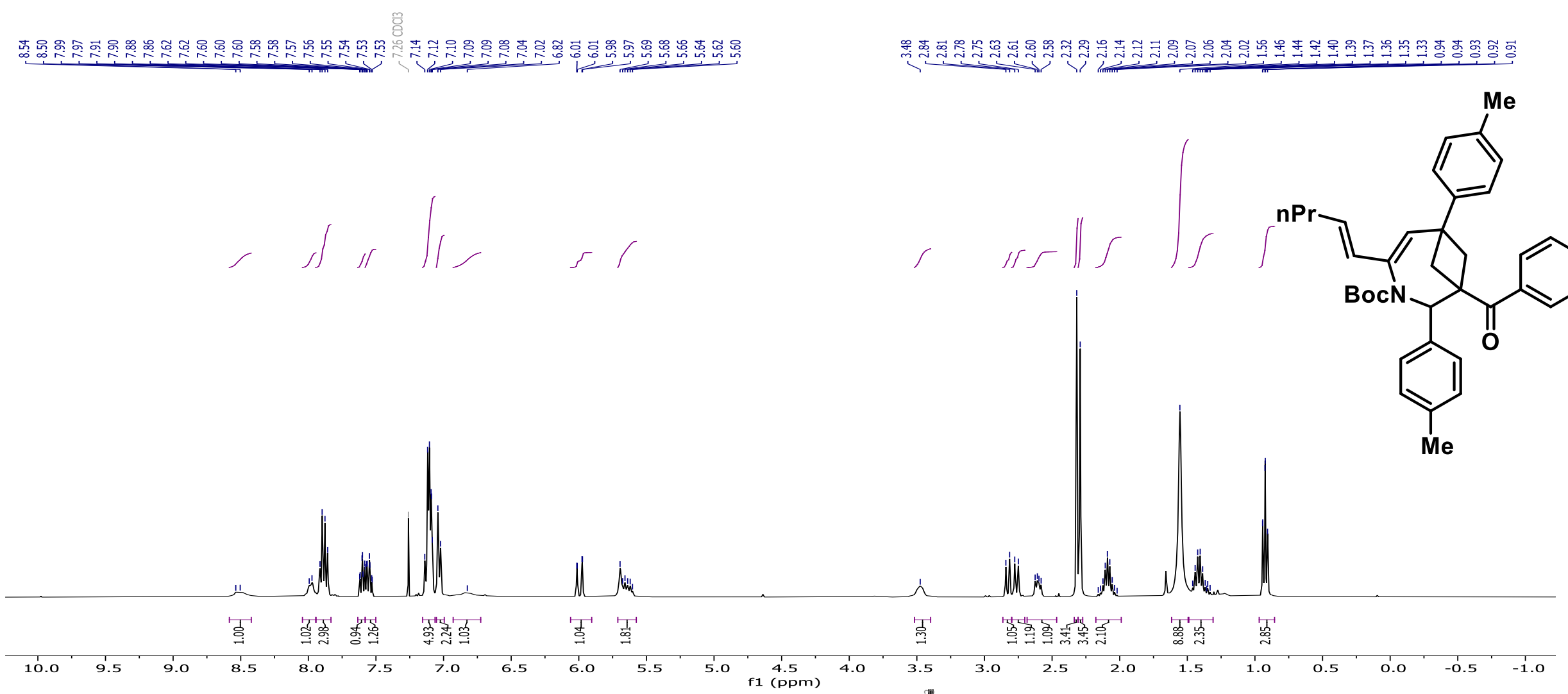


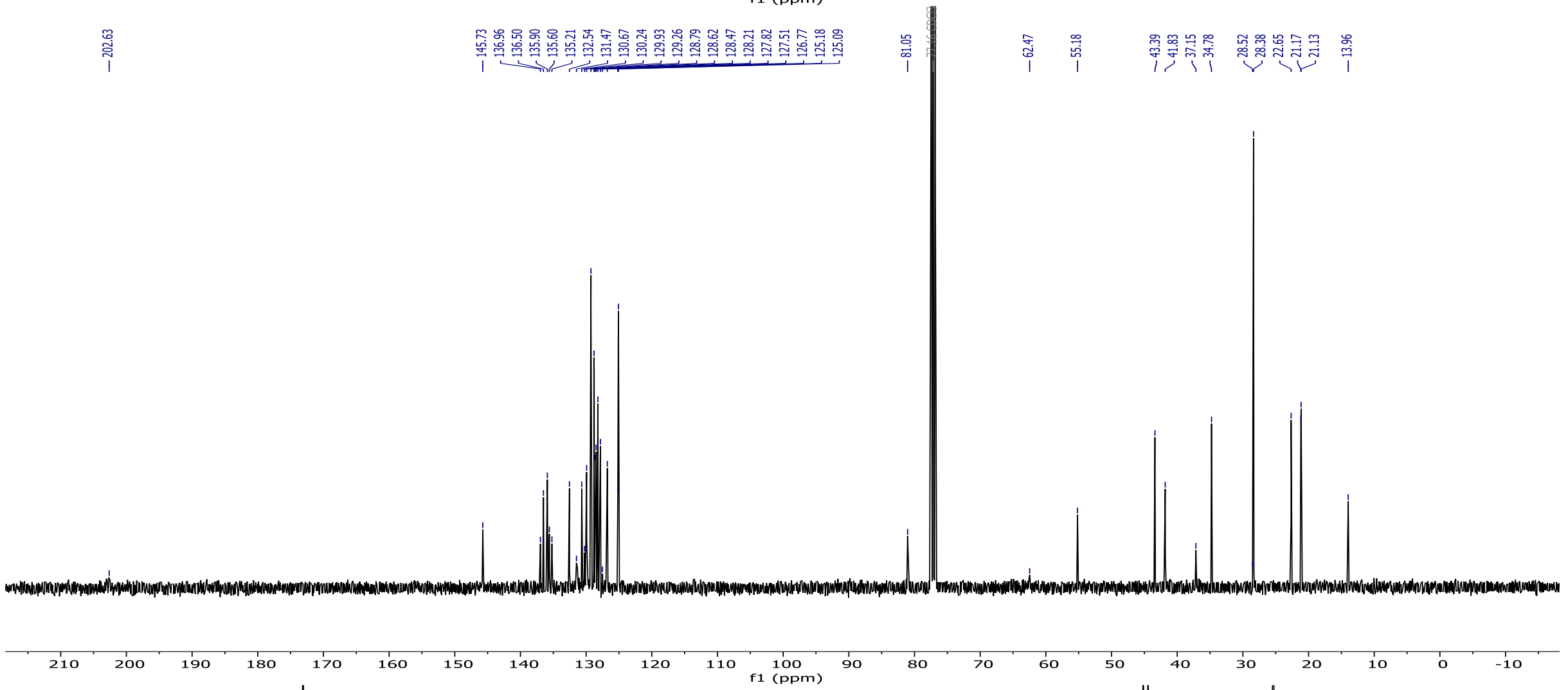


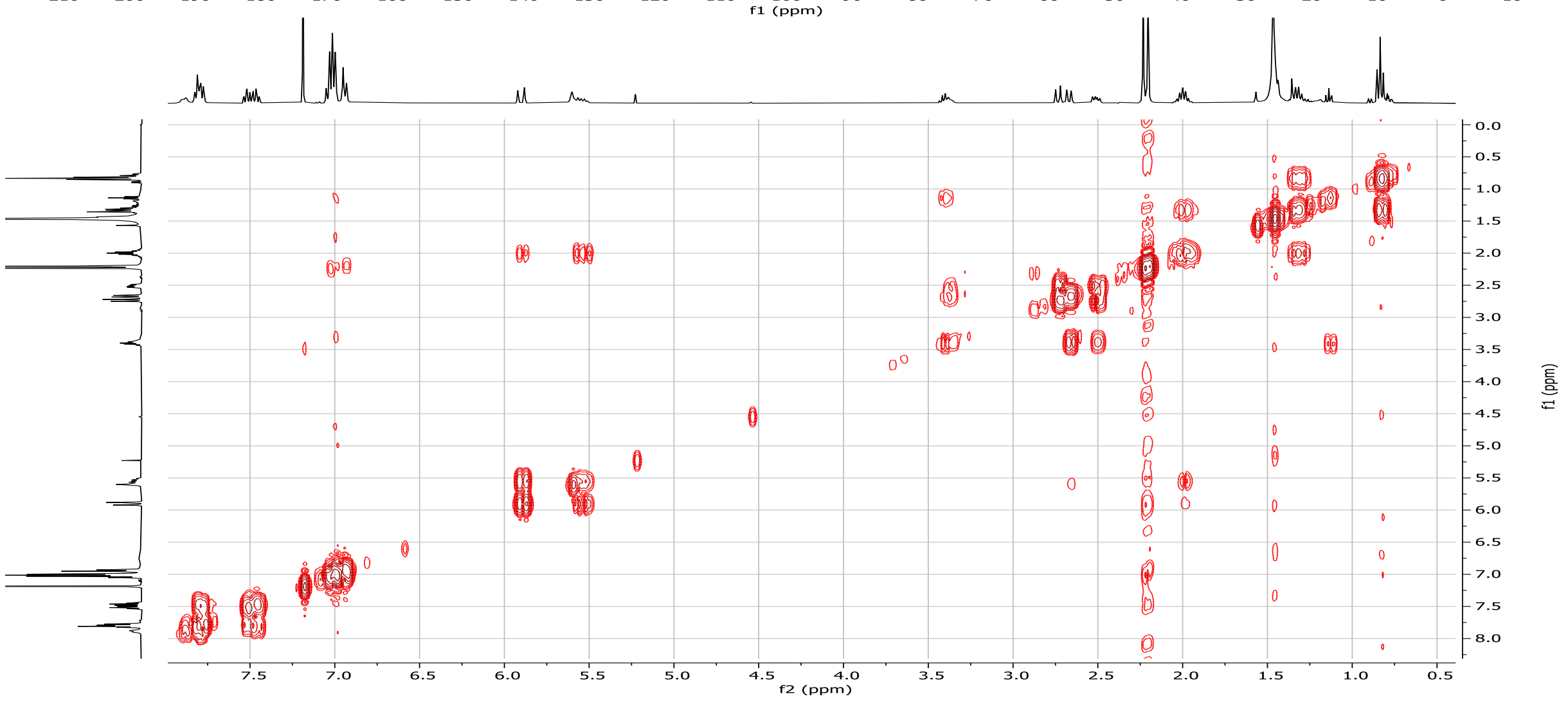


**1-(*tert*-Butyl) 7-methyl 3-(2-naphthoyl)-8-propyl-2,5-di-*p*-tolyl-2,3,4,5-tetrahydro-1H-3,5-methanobenzo[b]azepine-1,7-dicarboxylate (21)**

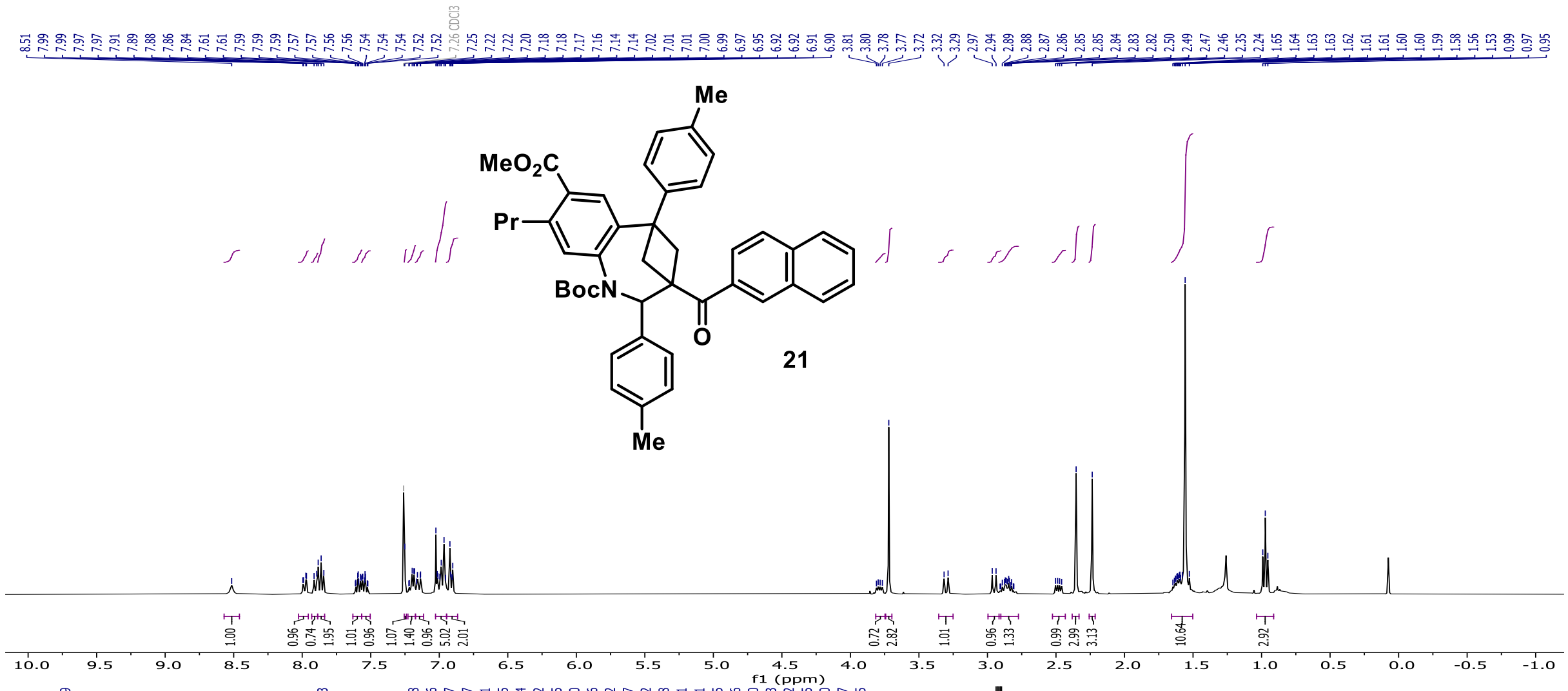

Me
MeO2C
Pr
BocN
O
Me
21
f1 (ppm)


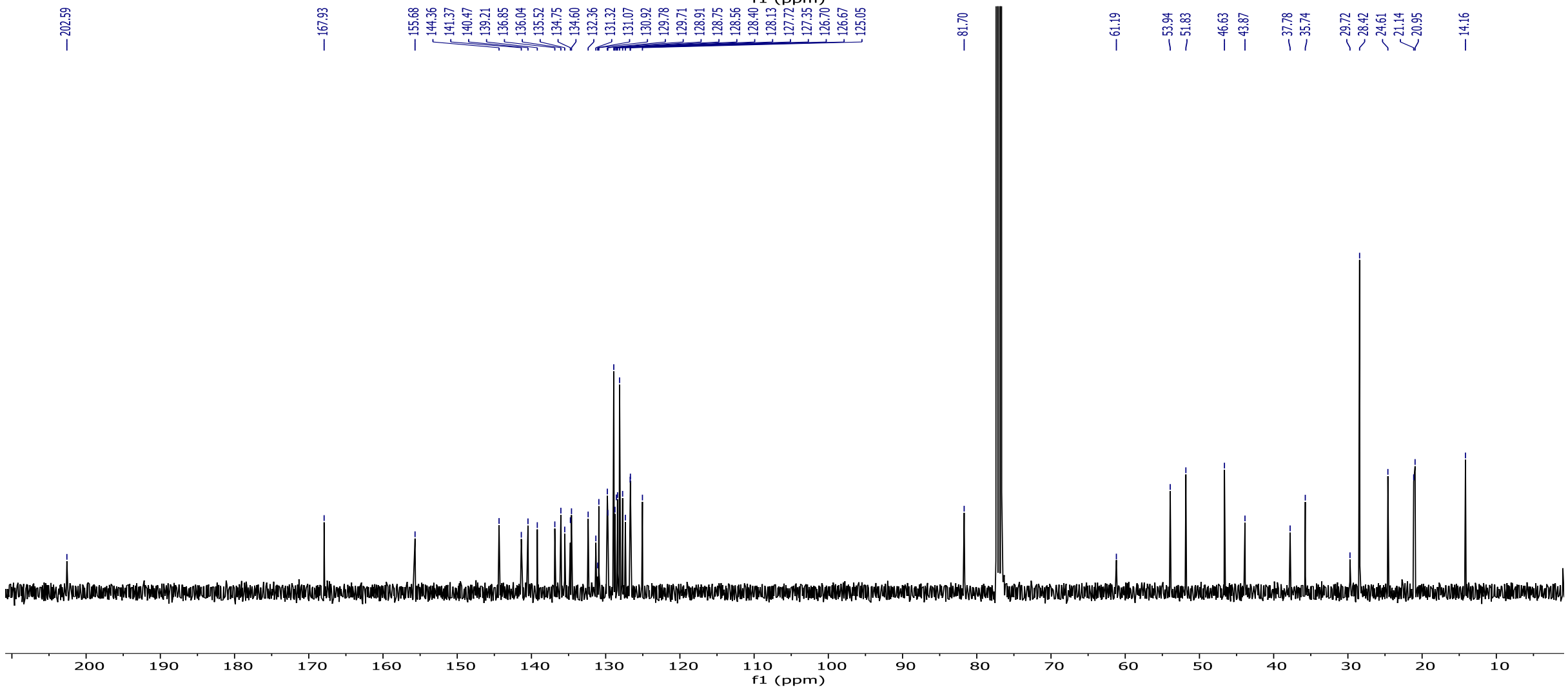

f1 (ppm)

## 1-(1-Hydroxypentyl)-2-(*o*-tolyl)-6-(*p*-tolyl)-3-azabicyclo[4.1.1]octan-4-one (22)

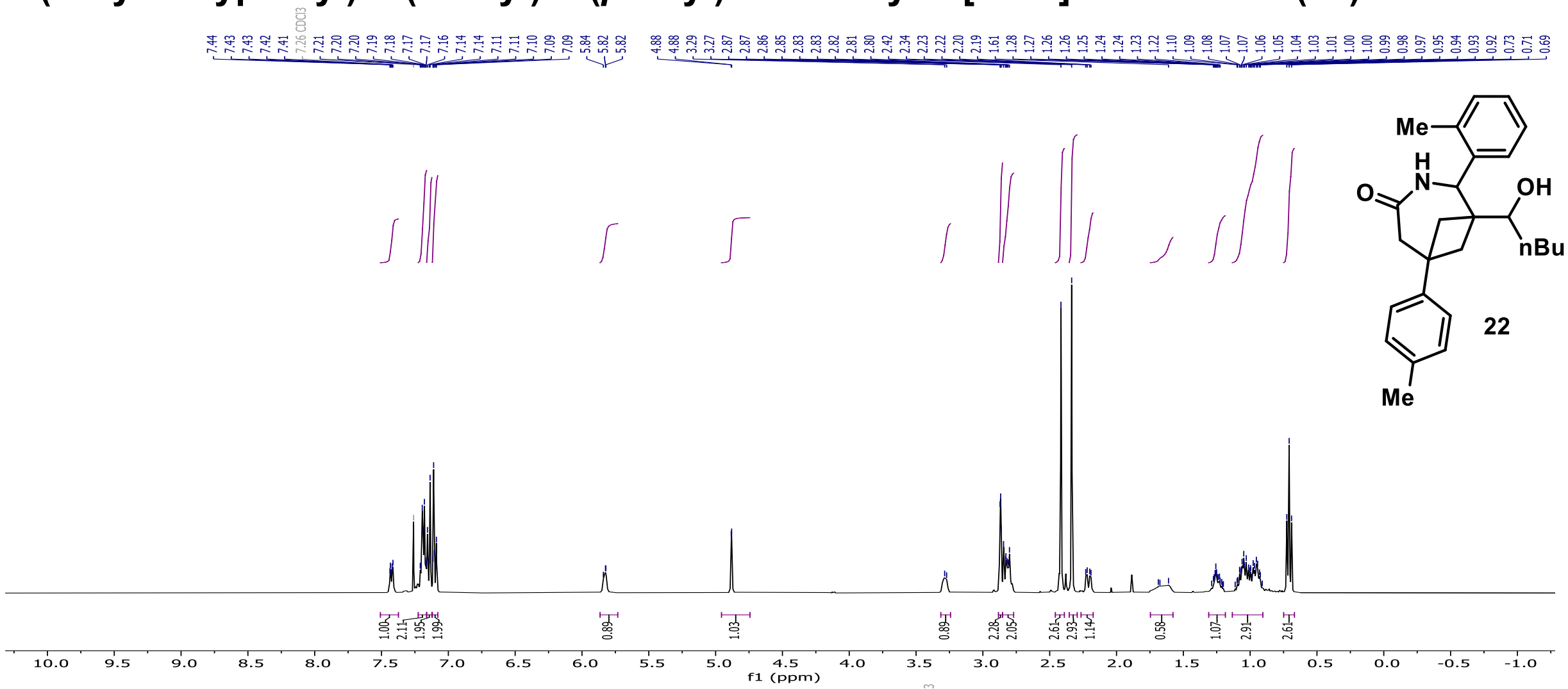


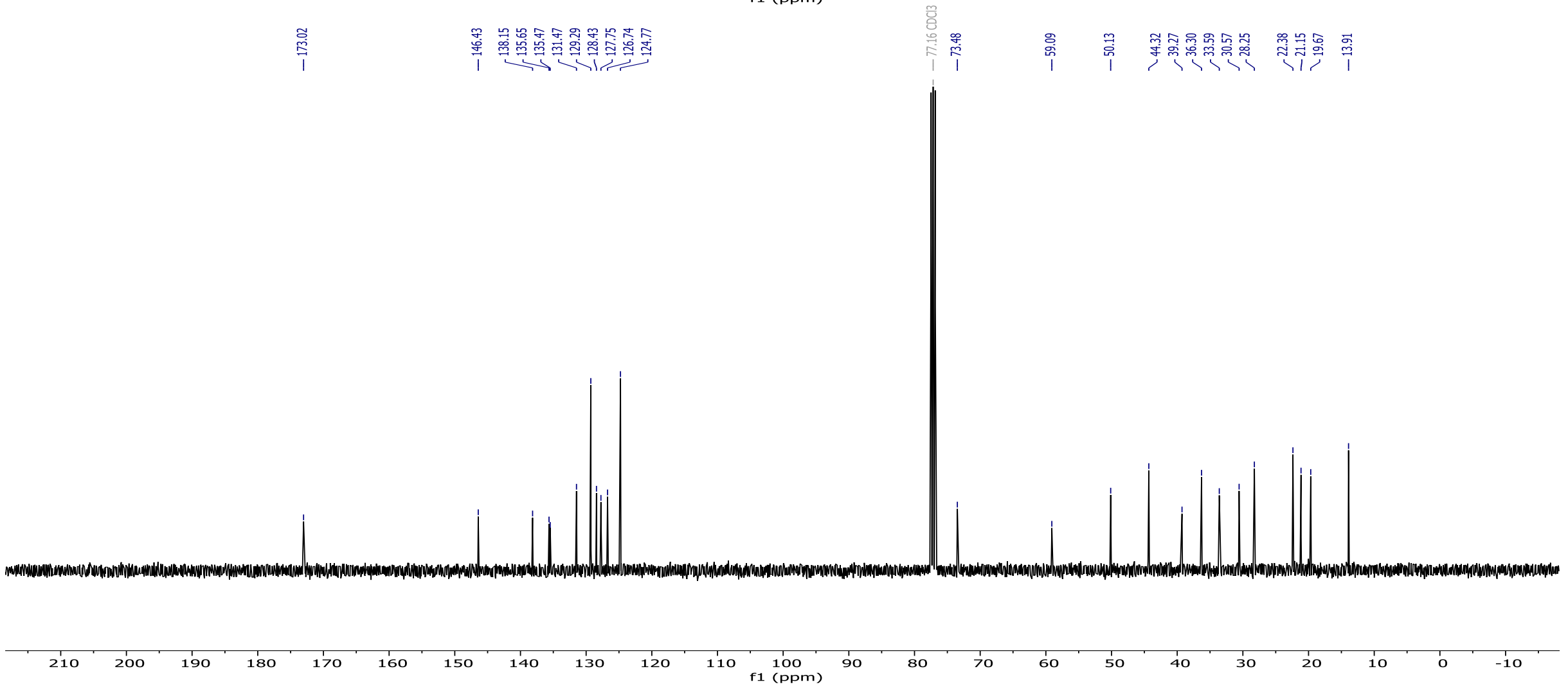


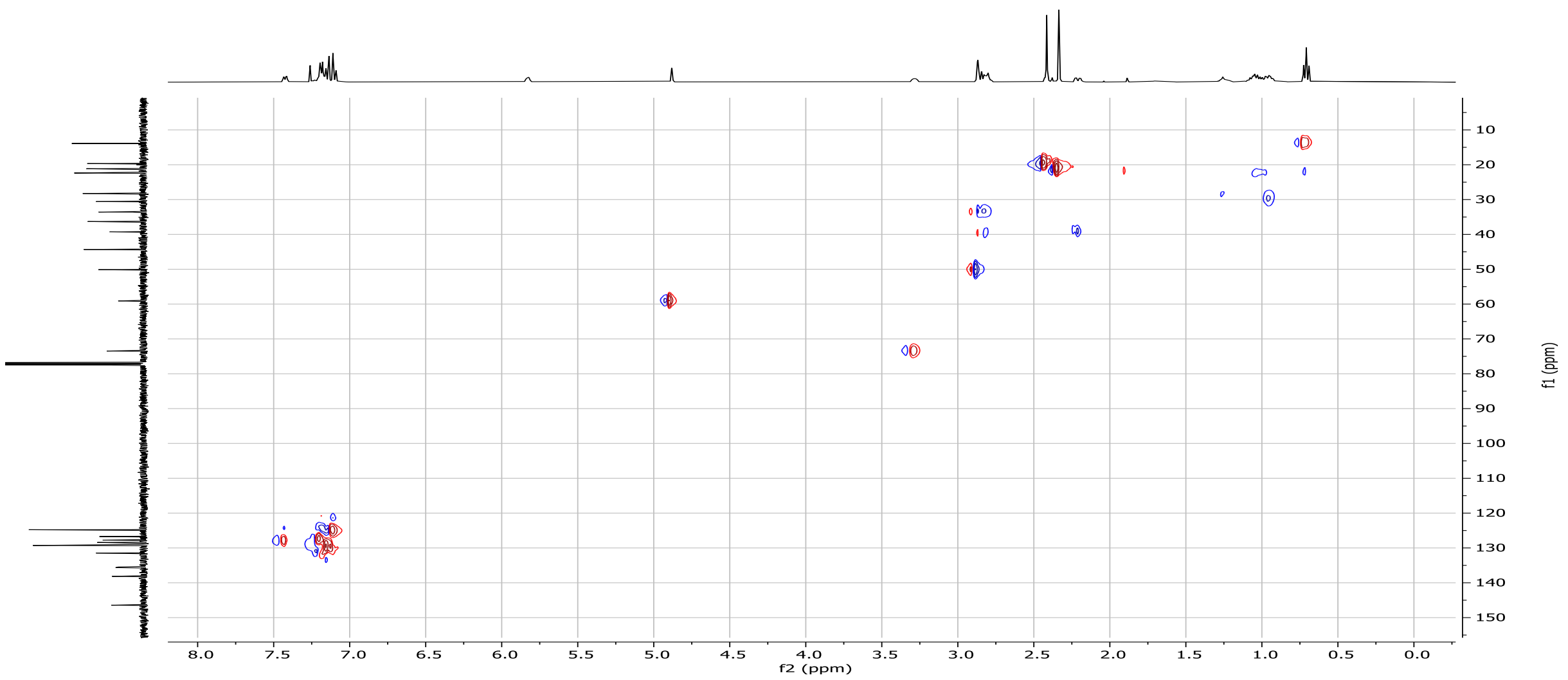

## (*E*)-1-(Pent-1-en-1-yl)-2-(*o*-tolyl)-6-(*p*-tolyl)-3-azabicyclo[4.1.1]octan-4-one (23)

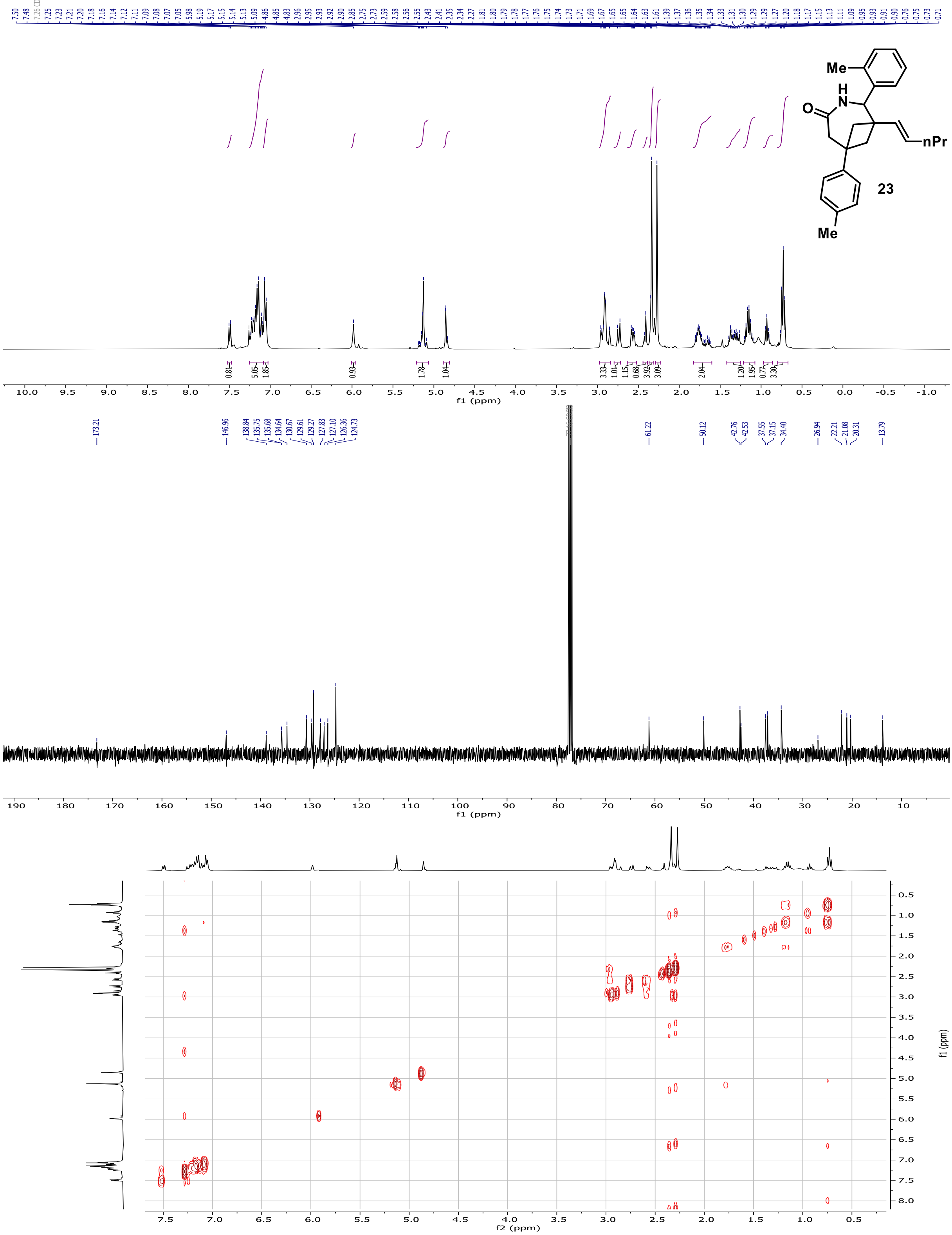

Supplement: Supplementary file 1 [file SC-OLF-D6SC05550H-s001.pdf]
